# Genotypic Expansion Within the Population Structure of Classical *Brucella* Species Revealed by MLVA16 Typing of 1404 *Brucella* Isolates From Different Animal and Geographic Origins, 1974–2006

**DOI:** 10.3389/fmicb.2018.01545

**Published:** 2018-07-12

**Authors:** Gilles Vergnaud, Yolande Hauck, David Christiany, Brendan Daoud, Christine Pourcel, Isabelle Jacques, Axel Cloeckaert, Michel S. Zygmunt

**Affiliations:** ^1^Institute for Integrative Biology of the Cell, CEA, CNRS, Univ. Paris-Sud, Université Paris-Saclay, Gif-sur-Yvette, France; ^2^ISP, INRA, Université François Rabelais de Tours, UMR 1282, Nouzilly, France; ^3^IUT de Tours, Tours, France

**Keywords:** *Brucella*, MLVA, population structure, genotyping, animal, human

## Abstract

Previous studies have shown the usefulness of MLVA16 as a rapid molecular identification and classification method for *Brucella* species and biovars including recently described novel *Brucella* species from wildlife. Most studies were conducted on a limited number of strains from limited geographic/host origins. The objective of this study was to assess genetic diversity of *Brucella* spp. by MLVA16 on a larger scale. Thus, 1404 animal or human isolates collected from all parts of the world over a period of 32 years (1974-2006) were investigated. Selection of the 1404 strains was done among the approximately 4000 strains collection of the BCCN (*Brucella* Culture Collection Nouzilly), based on classical biotyping and on the animal/human/geographic origin over the time period considered. MLVA16 was performed on extracted DNAs using high throughput capillary electrophoresis. The 16 loci were amplified in four multiplex PCR reactions. This large scale study firstly confirmed the accuracy of MLVA16 typing for *Brucella* species and biovar identification and its congruence with the recently described Extended Multilocus Sequence Analysis. In addition, it allowed identifying novel MLVA11 (based upon 11 slowly evolving VNTRs) genotypes representing an increase of 15% relative to the previously known *Brucella* MLVA11 genotypes. Cluster analysis showed that among the MLVA16 genotypes some were genetically more distant from the major classical clades. For example new major clusters of *B. abortus* biovar 3 isolated from cattle in Sub-Saharan Africa were identified. For other classical species and biovars this study indicated also genotypic expansion within the population structure of classical *Brucella* species. MLVA proves to be a powerful tool to rapidly assess genetic diversity of bacterial populations on a large scale, as here on a large collection of strains of the genomically homogeneous genus *Brucella*. The highly discriminatory power of MLVA appears of particular interest as a first step for selection of *Brucella* strains for whole-genome sequencing. The MLVA data of this study were added to the public *Brucella* MLVA database at http://microbesgenotyping.i2bc.paris-saclay.fr. Current version *Brucella*_4_3 comprises typing data from more than 5000 strains including *in silico* data analysis of public whole genome sequence datasets.

## Introduction

*Brucellae* are Gram-negative, facultative intracellular bacteria that can infect many species of animals and man. Until the 1990s six species were classically recognized within the genus *Brucella*: *B. abortus*, *B. melitensis*, *B. suis*, *B. ovis*, *B. canis*, and *B. neotomae* (Corbel and Brinley Morgan, 1984; Moreno et al., 2002; Godfroid et al., 2011). This classification was mainly based on differences in pathogenicity, host preference, and phenotypic characteristics (Alton et al., 1988). With the advent of modern molecular typing methods and whole genome sequencing a number of new species representing mostly wildlife isolates have been validly published. In chronological order it concerns the species (i) *B. ceti* and *B. pinnipedialis* isolated from marine mammals, with cetaceans (dolphin, porpoise, and whale species) and pinnipeds (various seal species) as preferred hosts respectively (Foster et al., 2007); (ii) *B. microti* isolated initially from the common vole but found later also in red foxes and in soil (Scholz et al., 2008a,b, 2009); (iii) *B. inopinata* isolated from human (Scholz et al., 2010); (iv) *B. papionis* isolated from baboons (Whatmore et al., 2014); and (v) the latest *B. vulpis* species isolated from red foxes (Scholz et al., 2016b). Novel *Brucella* strains representing potentially novel species have also been isolated from Australian rodents (Tiller et al., 2010a), a wide variety of frog species (Eisenberg et al., 2012; Fischer et al., 2012; Scholz et al., 2016a; Soler-Lloréns et al., 2016; Al Dahouk et al., 2017; Kimura et al., 2017; Mühldorfer et al., 2017), and surprisingly also from fish namely from a bluespotted ribbontail ray (*Taeniura lymma*) (Eisenberg et al., 2017). The genus *Brucella* nowadays is thus not restricted to mammal species. Particular attention is required to survey and study those novel isolates which may represent a potential risk to human health with possible associated difficulty of diagnosis and consecutive treatment. A good example to emphasize this problem is the novel *Brucella* sp. strain BO2, isolated from a patient with chronic destructive pneumonia (Tiller et al., 2010b), for which the animal or environmental reservoir has not been identified yet.

During the 1980s the six classical species were found by DNA–DNA hybridization to be highly genetically related (more than 90% DNA relatedness) which was later confirmed by whole genome sequencing (Paulsen et al., 2002). Consequently, from a strict genomic point of view, *Brucella* could be considered as a monospecific genus (Verger et al., 1985). However, for both medical and historical reasons the multispecies concept has been kept. Indeed the different lineages induce specific pathologies in farm animals as well as different risks of transmission and long-lasting disease in humans if antibiotic treatment is not applied adequately.

The species *B. melitensis*, *B. abortus*, and *B. suis* are further subdivided into biovars based on phenotypic characterization, such as serotyping, phage typing, sensitivity to dyes, or metabolic profiles (Alton et al., 1988). These classical phenotyping techniques have a limited discriminatory power and are mostly available in reference laboratories only. Some of them are time-consuming, require manipulating the living agent, and due to a lack of standardization of the typing reagents, they sometimes raise difficulties in the interpretation of the results. Therefore, several molecular typing methods have been developed since the end of the 1990s, especially when *Brucella* genome sequences became available. The most commonly used today are Multilocus Sequence Typing (MLST) and Multiple Loci VNTR (Variable Number of Tandem Repeats) Analysis (MLVA) (Le Flèche et al., 2006; Whatmore et al., 2007). Both methodologies are sufficiently highly discriminatory and provide a clustering of strains that is globally in accordance with the currently recognized *Brucella* species and biovars. Moreover, they have allowed identifying subtypes within each species or biovar based on geographic origin or host specificity. A good example for this are the marine mammal brucellae consisting currently of the species *B. ceti* and *B. pinnipedialis.* No biovars have been defined for these species, but nevertheless both species constitute a diverse set of distinct genotypes in both MLST and MLVA that are in clear congruence with the marine mammal host from which they were isolated (Groussaud et al., 2007; Maquart et al., 2009). Based on the latest published extended MLST scheme with 21 loci, over 100 sequence types (STs) were identified for the whole *Brucella* population structure, including 16 STs for marine mammal brucellae (Whatmore et al., 2016). From numerous studies both MLVA and MLST have proved to be useful to assess genetic diversity of *Brucella* strains and to identify and classify newly emerging or atypical isolates as novel species within the genus *Brucella* (Scholz and Vergnaud, 2013), which was not possible based on phenotypic characterization alone. In addition, both MLVA and MLST are robust and accurate and their implementation as rapid diagnostic assays may likely replace in the future the classical phenotyping scheme of *Brucella* species and biovar.

Since 10 years, numerous studies have been published independently using MLVA technology but mostly on a limited number of *Brucella* strains (less than 300) from specific geographic origins or hosts. The objective of the present study was to assess genetic diversity of *Brucella* spp. by MLVA on a larger scale. Thus, 1404 animal or human isolates covering all parts of the world over a period of 32 years (1974–2006), hosted by INRA in the *Brucella* Culture Collection Nouzilly (BCCN), were investigated. Data were compared for congruency with those of MLST when available, to try to determine the advantages and complementarity of each methodology. This was facilitated by taking advantage of the availability of whole genome sequence data from which both MLVA and MLST genotypes could be deduced by *in silico* analysis.

## Materials and Methods

### Bacterial Strains

Selection of the 1404 strains of this study was done among the approximately 4000 strains collection of the BCCN (*Brucella* Culture Collection Nouzilly), based on classical biotyping, and covering all geographic origins and hosts of the world over the period 1974–2006. Information on these strains is provided in Supplementary Figure S1 and Supplementary Table S1. Culture, DNA extraction, and PCR were performed using standard methods.

### MLVA

MLVA16 [Multiple Loci VNTR (Variable Number of Tandem Repeats) Analysis (MLVA) using 16 chromosomal loci] was performed as described previously (Scholz and Vergnaud, 2013), using high throughput capillary electrophoresis on a Beckman CEQ8000 machine. The 16 loci were amplified in four multiplex PCR reactions. The composition of each multiplex is indicated in Supplementary Table S2.

### MLST and MLVA *in Silico* Analysis of Available Genome Sequence Data

Multilocus Sequence Typing codes of complete genomes or *de novo* assemblies from reads archives were deduced *in silico* using the BioNumerics (Applied-Maths) version 7.6.2 tools. The scheme for MLST coding was recovered from the *Brucella* MLST database at https://pubmlst.org/brucella/.

The scripts used to deduce MLVA codes of complete genomes or draft assemblies are deposited at https://github.com/dpchris/MLVA. In order to estimate the read length necessary for a correct reconstruction of tandem repeats length from read archives, artificial reads data were produced from complete genome sequences using artificial fastq generator (Frampton and Houlston, 2012). The reads were then assembled using SPAdes version 3.9 (Bankevich et al., 2012).

Supplementary Table S3 indicates that reads longer than 200 bp can be used to reconstruct all VNTRs with a reasonable success rate, whereas only some VNTRs can be confidently reconstructed with shorter reads.

### Whole Genome SNP Analysis

Sequencing reads were mapped on a reference genome using BioNumerics version 7.6.2. The *B. melitensis* 16M assembly GCA_000740415.1 was used as reference after concatenating chromosome I accession number CP007763.1 and chromosome II accession number CP007762.1 in a single file. SNPs were called within BioNumerics using the strict closed dataset option. Minimum spanning trees were produced with the hypothetical missing links option.

## Results and Discussion

### Global Clustering Analysis of MLVA Data

**FIGURE 1:**
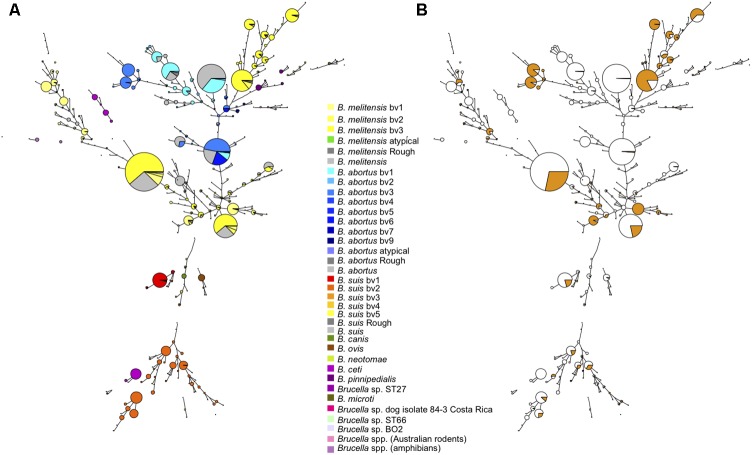
Global view of the genetic diversity of *Brucella* spp. provided by MLVA11. Entries with a full MLVA11 dataset were used to produce a minimum spanning tree based upon 4998 entries, allowing hypothetical missing links. This includes 1404 *Brucella* strains from the present study, and 375 *in silico* deduced data (from 48 assemblies and 327 sequence reads archives). 377 MLVA11 genotypes are defined. **(A)** Strains are color coded according to species and biovar as indicated. **(B)** The proportion of strains from the present study is reflected by the colored sector in each circle.

**Figure 1** shows the MLVA11 genotype distribution of 4971 *Brucella* strains which include previously published strains and the 1404 strains of this study listed in Supplementary Table S1. In total 377 MLVA11 genotypes were defined, of which 63 new ones from this study. At the species level the largest contribution in this study was *B. melitensis*, with 1049 strains analyzed from diverse geographic origins and hosts (see Supplementary Figures S1, S2). Previously, three major clusters within *B. melitensis* were defined, based on MLVA data, in agreement with a preferred geographic location, and called “Americas,” “West Mediterranean,” and “East Mediterranean” (Le Flèche et al., 2006). This clustering was subsequently confirmed by MLST (Whatmore et al., 2016). As can be seen in **Figure 1B** most of the 1050 *B. melitensis* strains studied are uniformly distributed within the East Mediterranean or West Mediterranean clades, of which some are new for the East Mediterranean clade. A lower proportion of strains were distributed in the Americas clade. The 213 *B. abortus* strains of this study were predominantly found in the MLST21-defined clade called *B. abortus* B. The remaining 142 strains of this study, classified within a species, were mainly *B. suis* biovar 2 (*n* = 92), followed by *B. suis* biovar 1 (*n* = 30) and a few strains of *B. canis* (*n* = 1), *B. ovis* (*n* = 2), and *B. neotomae* (*n* = 7). Most of them clustered with previously described MLVA11 genotypes. These different species/biovars were clearly separated from each other and from the other major *Brucella* species. Recently reported strains isolated from Australian rodents representing a potential novel *Brucella* species were also included in this study, and MLVA11 confirmed them as a specific separate *Brucella* lineage (Tiller et al., 2010a). A large number of marine mammal *Brucella* strains (*n* = 295), of the species *B. ceti* and *B. pinnipedialis*, has been previously characterized by MLVA (Maquart et al., 2009), and are included in **Figure 1**. Species *B. ceti* and *B. pinnipedialis* form distinct MLVA11 clades from the other *Brucella* species. Species *B. microti*, and *B. suis* biovars 4 and 5 are similarly shown and form also distinct clades. *B. suis* biovar 5 represents a distant clade relative to the species *B. suis*, that does not cluster with any of the other biovars including *B. canis*, known to be closely genetically related to *B. suis*.

Taking all MLVA11 data together (*n* = 4971), and in agreement with global animal or human brucellosis epidemiology, since the 1970s *B. melitensis* is the predominant species followed by *B. abortus* and *B. suis*.

### B. melitensis

**FIGURE 2:**
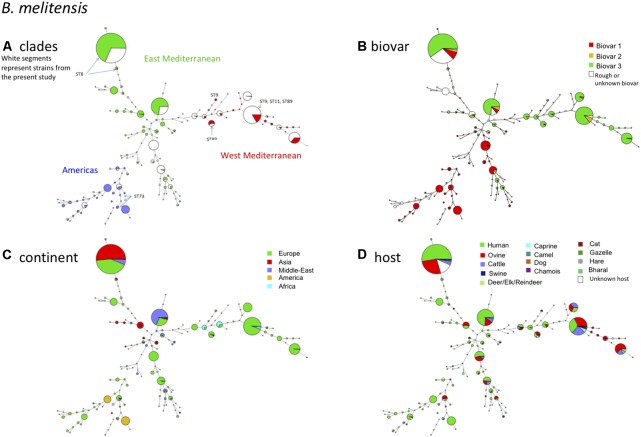
*B. melitensis* intraspecies diversity view provided by MLVA11. 2320 among the 4971 entries with a full MLVA11 dataset belong to *B. melitensis.* 1049 entries are from this study, 17 are *in silico* entries (nine complete genomes and eight entries based on sequence reads archives) and 1254 are compiled from MLVA publications. The same MLVA11 minimum spanning tree was color-coded according to different characteristics. **(A)** Color coding according to assignment to East Mediterranean, West Mediterranean, or Americas groups except for strains typed in the course of the present study (white). The MLST21 genotypes deduced from whole genome sequence data are indicated. **(B)** Color-coding according to biovar (white when unknown). **(C)** Color-coding according to geographic origin defined at continent level. **(D)** Color-coding according to host (white when unknown).

If we take a closer look at the largest panel of strains of this study belonging to the species *B. melitensis*, in the MLVA11 based minimum spanning tree shown in **Figure 2**, we confirm a distribution reflecting geographic origins previously defined by MLVA (Le Flèche et al., 2006), i.e., East Mediterranean, West Mediterranean, and the Americas. Most of the strains belonged to the East Mediterranean group which actually comprises strains from Europe, the Middle-East, and Asia (**Figure 2C**). A more precise geographic origin is indicated in Supplementary Figure S1. It must be noted that for Europe most strains were from Spain, France, or Greece. The West Mediterranean group consisted mostly of strains from Europe (Mediterranean countries) and to a lesser extent of strains from Africa, while the Americas group was mainly composed of strains from the Americas and from Europe (**Figure 2C**).

As observed before and also in agreement with MLST (Whatmore et al., 2016) (corresponding ST types of MLVA clades are indicated in **Figure 2A**), there was no clear relationship between genotype and biovar of *B. melitensis* (**Figure 2B**). In summary the following observations are fully congruent with MLST data (i) strains of the “Americas” clades are essentially of biovar 1, (ii) the “West Mediterranean” clades are mainly composed of biovar 3 strains and some contain a minority of biovar 2 strains, (iii) the “East Mediterranean” clades are more heterogeneous and comprise strains belonging to all three biovars, but biovar 2 remains minor and is likely a minor biovar within species *B. melitensis*. *B. melitensis* biovars are solely classified based on serological reaction using polyclonal antibodies directed against the main surface antigen consisting of the O chain of the outer membrane lipopolysaccharide (LPS). Structurally and antigenically this O chain is not highly diverse among the *Brucella* species and biovars, and therefore strains are classified by use of established anti-A or anti-M monospecific serum only within three serotypes, namely A+ (or A-dominant), M+ (or M-dominant), and A+M+ strains (Alton et al., 1988; Zygmunt et al., 2015). Those that do not react with these monospecific sera usually lack the O chain and are classified as rough type (R). Each of these serotypes exists also within the major species *B. abortus* and *B. suis*, but unlike *B. melitensis*, these species comprise other biovar markers than the serotype (Alton et al., 1988). As suggested in the MLVA study of Le Flèche et al. (2006) and confirmed by the MLST study of Whatmore et al. (2016), the biovar concept in the case of *B. melitensis* appears of limited epidemiological value and MLVA or MLST may better fulfill questions regarding epidemiology and tracking epidemic strains.

Regarding the host distribution of *B. melitensis*, the main hosts were as expected ovine and human, followed by cattle and caprine (**Figure 2D**). Strains from these hosts were quite uniformly distributed within the Americas, East or West Mediterranean MLVA clades. *B. melitensis* was also encountered in less frequent or unexpected hosts such as camel (mostly dromedary), cat, dog, swine including wildlife hosts such as chamois, bharal, elk, hare, gazelle, or reindeer.

### B. abortus

**FIGURE 3:**
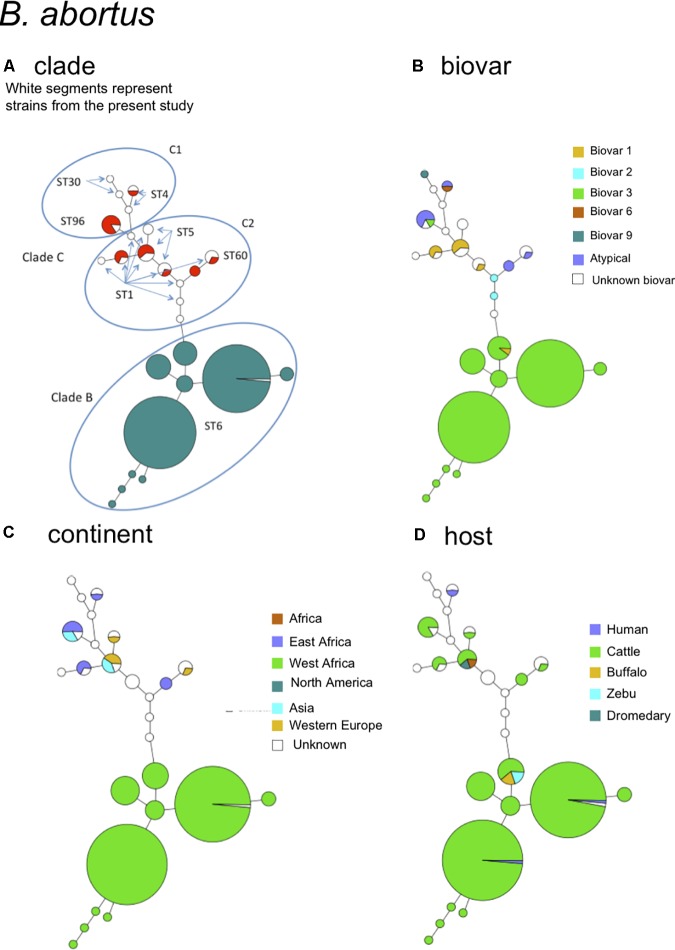
*B. abortus* intraspecies diversity view provided by MLVA11. 232 entries are used including 213 *B. abortus* strains from this study and 19 representative entries from *in silico* data. The same MLVA11 minimum spanning tree was color-coded according to different characteristics. **(A)** Color coding according to assignment to *B. abortus* clade C or *B. abortus* clade B. Uncolored (white) entries correspond to *in silico* data. The MLST21 ST genotype for the white entries is indicated and was used to deduce the A, B, or C clade assignment defined by Whatmore et al. (2016). A tentative assignment of clades C1 and C2 is also proposed. **(B)** Color coding according to biovar (white when unknown). **(C)** Color-coding according to geographic origin defined at continent level (white when unknown). **(D)** Color-coding according to host (white when unknown).

The species *B. abortus* could be subdivided by MLVA in three clades congruent with the clades B, C1, and C2 of MLST (Whatmore et al., 2016). The correspondence with MLST STs is indicated in **Figure 3A**. Most strains of this study were of clade B and originated from Africa (Sub-Saharan, see Supplemental Figure S1 for details), mostly isolated from cattle and some from dromedary, zebu, or human (**Figures 3C,D**). Clade B strains predominantly belonged to biovar 3 (**Figure 3B**). Clade C strains, although more limited in number in this study, appeared more widely distributed over the other continents America, Asia, and Europe (**Figure 3C**) and comprised other *B. abortus* biovars such as biovar 1, 2, 6, and 9 (**Figure 3B**). They were mostly isolated from cattle but one C2 clade (corresponding to ST5 in MLST) comprised also buffalo strains.

### *B. suis* – *B. canis*

**FIGURE 4:**
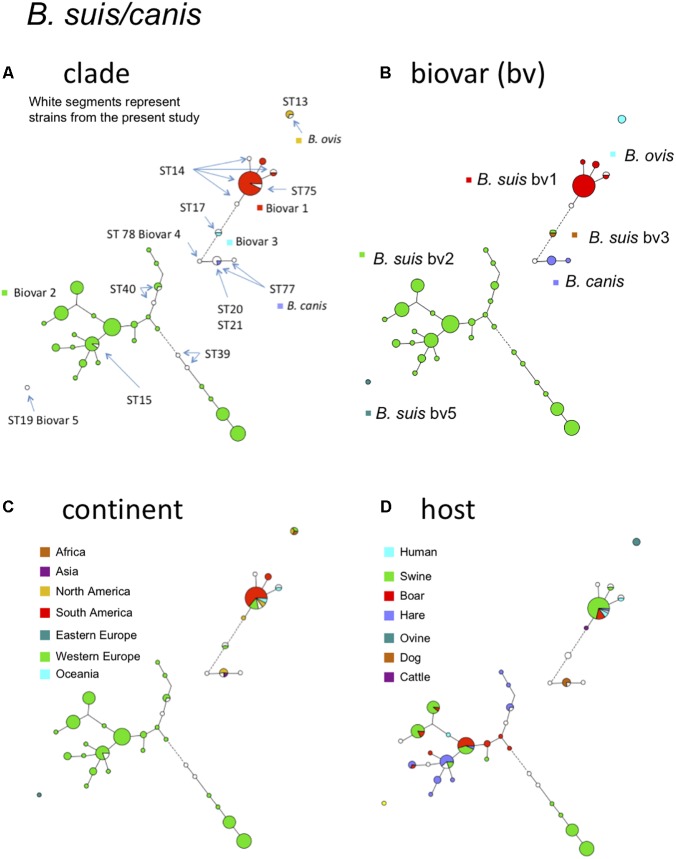
*B. suis, B. canis*, and *B. ovis* intraspecies diversity according to MLVA11. The minimum spanning tree was drawn with data from 138 entries including 120 strains from this study and 18 representative entries from *in silico* data. **(A)** Color coding according to species and biovar, *B. suis* bv1, bv2, bv3, *B. canis*, or *B. ovis*. Uncolored (white) entries correspond to *in silico* data. The MLST21 ST genotypes for the white entries are indicated. **(B)** Color coding according to biovar (white when unknown). **(C)** Color-coding according to geographic origin defined at continent level (white when unknown). **(D)** Color-coding according to host (white when unknown).

**Figure 4** shows the *B. suis* and *B. canis* MLVA clades distribution with the corresponding MLST STs. *B. suis* biovar 4 and *B. canis* are known to be phylogenetically closely related and this is also supported by the MLVA or MLST data. On the other hand, *B. suis* biovar 5 appears far more distant. Most strains of this study belonged to *B. suis* biovar 1 or biovar 2. The large proportion of biovar 2 strains from Western Europe correspond to an emergence of this biovar in European countries. A change in agricultural practices namely rearing in open fields of swine bringing them in close contact with wildlife reservoirs, mainly wild boar and hare might be responsible for this emergence (Godfroid et al., 2011). Some MLVA clades comprised strains isolated from the three predominant hosts (swine, wild boar, hare) (**Figure 4D**). Some more distant clades consisted exclusively of swine or hare isolates. *B. suis* biovar 1 strains formed distinct clades from biovar 2 with a major one corresponding to MLST21 ST75 (**Figure 4A**). Those strains were more widely distributed with a majority from South America, followed by Europe, North America, and Oceania (**Figure 4C**). The major host for this biovar is swine, but the clade corresponding to ST75 comprised also wild boar, hare, and human isolates (**Figure 4D**).

### *Brucella* spp. From Rodents and Human Isolate BO2

**FIGURE 5:**
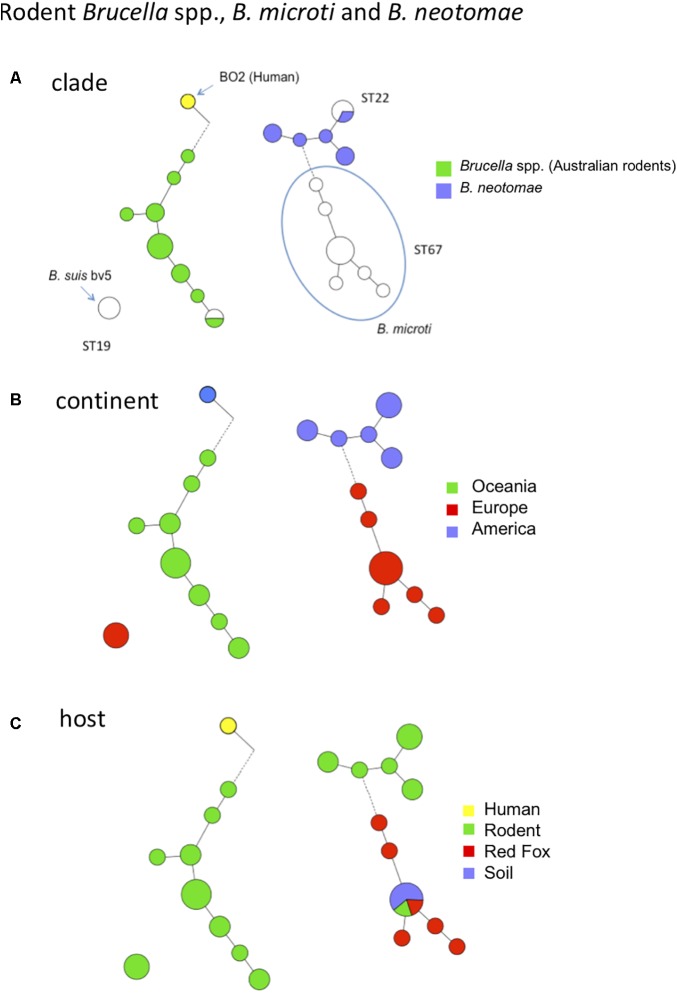
MLVA11 diversity of rodent *Brucella* spp., *B. microti*, *B. neotomae* and human *Brucella* sp. isolate BO2. Thirty-seven entries are used including 21 strains from this study (*B. neotomae*, *Brucella* spp. from Australian rodents, and human *Brucella* sp. isolate BO2), six representative entries from *in silico* data and data from 10 previously reported strains including *B. microti*. **(A)** Color coding according to species and biovar. Uncolored (white) entries correspond to *in silico* or previously published data. The MLST21 ST genotype is indicated. **(B)** Color-coding according to geographic origin defined at continent level. **(C)** Color-coding according to host.

Over the past decade there has been growing interest for *Brucella* spp. isolated from rodents because they may constitute a reservoir for novel *Brucella* species that may be potential novel zoonotic pathogens. Some of them have been evaluated in mouse or cellular models of infection (Jiménez de Bagüés et al., 2010, 2014). Among these novel species, *B. microti* has firstly been isolated from the common vole in the Czech Republic (Hubálek et al., 2007; Scholz et al., 2008b), but was later also identified in the red fox from Austria (Scholz et al., 2009), and most recently in wild boar from Hungary (Rónai et al., 2015). Of interest is that *B. microti* has also been isolated from soil 7 years after its first isolation from common voles at the same location, suggesting that this species persists in soil (Scholz et al., 2008a). The diversity of *B. microti* strains has been previously assessed by MLVA (Al Dahouk et al., 2012). In this study we analyzed other strains from rodents, namely a set of strains belonging to the species *B. neotomae*, two strains of *B. suis* biovar 5, and a set of strains isolated from Australian rodents and the human *Brucella* sp. strain BO2 isolated from a lung biopsy from a patient presenting chronic destructive pneumonia (Tiller et al., 2010b). The latter strains (from Australian rodents and BO2) represent potential novel species phylogenetically closer to *B. inopinata* than the classical *Brucella* species (Tiller et al., 2010a). **Figure 5** shows MLVA cluster analysis of these strains and the corresponding MLST ST in comparison with the *B. microti* strains previously published (Al Dahouk et al., 2012). The four groups of rodent strains and human isolate BO2 are clearly separated with no apparent epidemiological link. As a consequence, from these and previous molecular data provide no clue regarding the animal or environmental reservoir for the human *Brucella* sp. isolate BO2. Although only a limited number of rodent strains are currently available (*n* = 37 in **Figure 5**), intra-species diversity is observed by MLVA within each species represented, except *B. suis* biovar 5. Regarding host diversity only *B. microti* appears not restricted to rodents (**Figure 5C**).

### Taking Advantage of Available Whole Genome Sequence Data to Link MLVA Clusters Onto MLSA-Based Phylogeny

MLVA is an efficient clustering tool but with moderate phylogenetic value on its own. Phylogeny can be recovered indirectly by anchoring MLVA genotypes on a phylogenetic framework, as provided by whole or partial (as in MLST) genome analysis. This can be done if a representative set of strains has been typed with both methods as described by Riehm et al. (2012). In theory, both MLVA and MLST data can be deduced from whole genome sequence data. We recovered fifty-four complete *Brucella* genomes from the EBI-ENA public databases. Seventeen correspond to 14 reference strains (strains *B. abortus* 870, *B. melitensis* 16M, and *B. suis* 1330 were sequenced twice independently), which we have also typed *in vitro* by MLVA via electrophoresis of PCR amplification products. The *in vitro* and *in silico* deduced MLVA11 genotypes were identical with three exceptions. *B. abortus* C68 full genome is *in silico* coded 2 at locus Bruce06 whereas it is *in vitro* coded 3. The duplicate full genome sequences of strains 870 and 16M differ respectively at locus Bruce06 and Bruce42 by a one repeat unit difference. One of the two genome sequences is in agreement with *in vitro* typing data. These two loci are fairly stable so that the most likely explanation for these discrepancies is an incorrect assembly of the corresponding tandem repeat in the generation of one of the full genome sequences. Consequently, most complete genome assemblies appear to correctly reconstruct the tandem repeat loci used in the MLVA11 assay. Because more than 1000 *Brucella* sequence reads archives are currently accessible in public depositories, we have evaluated the read length which allows reconstruction of a given tandem repeat with a moderate error rate. For this purpose we have produced artificial sequence reads of different length from each of the complete genomes.

We then reassembled the genomes using Spades version 3.9 and *in silico* deduced the resulting MLVA genotype. Supplementary Table S3 shows that all alleles which could be assembled from 250 bp long reads have a correct size. In contrast, shorter reads are often incorrectly assembled. Loci Bruce06, Bruce42, Bruce55, and Bruce21 are the most challenging in terms of reassembly at least when using Spades 3.9 and reads up to 200 bp long. When an *in silico* MLVA reconstruction corresponds to an unknown MLVA11 genotype, we suggest to confirm it by PCR amplification and sequencing the loci that are responsible for the new genotype.

**FIGURE 6:**
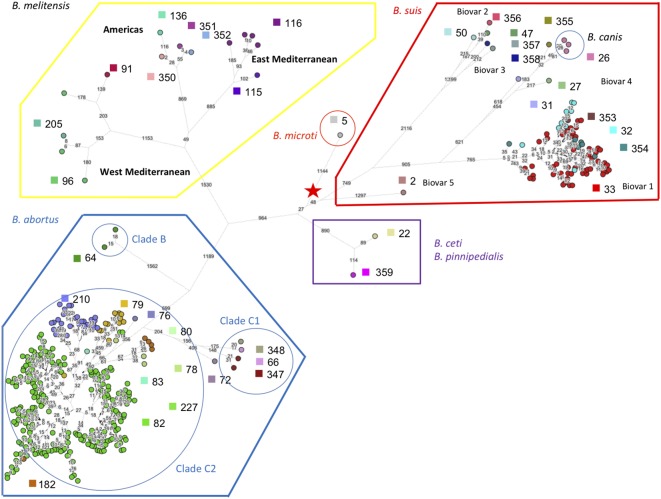
Superposition of whole genome phylogeny and MLVA11 genotypes of phylogeny deduced from whole genome SNP analysis of 355 sequence reads archives and complete genomes compatible with robust *in silico* MLVA genotypes reconstruction. Three hundred and fifty-five whole genome sequence datasets fulfilled the required criteria for a robust *in silico* reconstruction of MLVA genotypes, i.e., sufficiently long reads. The datasets were also used for a whole genome SNP analysis as well as for *in silico* MLST21 genotypes assignment. The color code and associated numbers indicate MLVA11 genotypes. *B. abortus* B, C1, and C2 MLST clades assignments are indicated. The numbers on branches are the number of SNPs. The length of branches is the square root of the SNP number.

Next we assembled public sequence reads archives with reads of appropriate length according to the previous simulation. For example, Illumina MiSeq 250 bp paired ends files allow to confidently reconstitute all VNTR loci, whereas reads archives with shorter reads are used to reconstruct only some loci as deduced from Supplementary Table S3. From these assemblies, MLVA and MLST data were extracted *in silico*. Full MLVA11 and MLST9 (according to Whatmore et al., 2007) genotypes could be recovered for 355 datasets including the complete genome sequences. **Figure 6** shows a minimum spanning tree deduced from whole genome SNP analysis. The tree is in agreement with the MLSA clade assignment. The three clades within *B. melitensis*, previously identified by MLVA and MLST, were well-resolved. Within *B. suis*, the clusters initially defined by biotyping (biovars 1 to 5) and further supported by MLVA and MLST, were also well-preserved. The *B. suis* biovar 5 lineage defines the first split within the *B. suis* species. From the split, 1297 SNPs define the branch leading to biovar 5. In contrast, the speed of branch expansion is twice relative to *B. suis* biovar 2 (up to 2648 SNPs). According to the figure, the rate of expansion of the different lineages appears generally variable. More than 4,000 SNPs occur from the root indicated by the red star in the figure to the tip within the *B. melitensis* West Mediterranean clade. *B. microti* defines the shortest branch with approximately 1000 SNPs from root to tip. **Figure 6** thus illustrates how both MLST and MLVA can be used to assign a strain to a position on the phylogenetic tree produced by whole genome SNP analysis.

## Conclusion

Both MLVA and MLST constitute valuable complementary tools to investigate genetic diversity and molecular epidemiology of *Brucella* species and biovars. In addition to their ability to identify rapidly pathogenic *Brucella* species or to their recognized contribution to identify novel *Brucella* species, they both are able to discriminate below the biovar level, down to a strain level representative of a geographic origin or particular host.

Multilocus Sequence Typing as classically done, i.e., via sequencing of nine (MLST9) or 21 (MLST21) PCR amplification products might soon become too expensive compared to the running of draft whole genome sequencing, which has much greater added value. Nevertheless, MLST genotypes are a very valuable way of naming lineages, and can be readily deduced from whole genome sequences as used in this study.

MLVA is a convenient first line assay for outbreak investigations, fast quality check of strain identity in a collection, and identification of outliers, i.e., strains which should be sequenced in priority as they may represent new lineages. With more than 5,000 strains in the current version of the online database, it is likely that most of the existing genotype diversity has now been uncovered and that the discovery of new MLVA11 genotypes will be limited.

From a practical point of view, MLVA can be run with a variety of equipment including regular agarose gels (Le Flèche et al., 2006), monoplex capillary electrophoresis (De Santis et al., 2013), or multiplex PCR multicolor capillary electrophoresis (Garofolo et al., 2013). Target loci can be selected to suit a specific epidemiological background. In a given area, only few MLVA11 genotypes will be encountered. It will not be necessary to type all 11 loci, and conversely it will be very valuable to include additional, more discriminatory VNTRs not included in the MLVA16 assay. When MLVA was initially developed, a subset of loci was selected for practical reasons among a large collection of potentially interesting markers (Bricker et al., 2003; Le Flèche et al., 2006; Whatmore et al., 2006). Owing to the accessibility of whole genome sequencing, tailored optimized MLVA assays can now be developed. For this purpose, one only needs to draft sequence a few local representative strains, deduce the MLVA profile at all known *Brucella* VNTR loci, identify the relevant VNTRs, and use these in a new assay. We have shown here that sequencing reads with a length of 250 bp or more will provide accurate assemblies. The advent of sequencing machines providing long reads may further simplify the process. *In silico* MLVA typing requires a correct determination of the length of the tandem repeat, but is not very demanding in terms of internal sequence accuracy.

## Author Contributions

GV, CP, AC, and MZ conceived and designed the study. GV, AC, and MZ analyzed the data and drafted the manuscript. YH, GV, and CP performed experimental work and data management and control. DC performed *in silico* data analyses. BD upgraded the MicrobesGenotyping web site and underlying database. IJ oversaw strains provision, DNA preparation, and biotyping.

## Conflict of Interest Statement

The authors declare that the research was conducted in the absence of any commercial or financial relationships that could be construed as a potential conflict of interest.
