# Supplementary material for: Genotypic Expansion Within the Population Structure of Classical Brucella Species Revealed by MLVA16 Typing of 1404 Brucella Isolates From Different Animal and Geographic Origins, 1974–2006
Source: Front Microbiol. 2018 Jul 12;9:1545. doi: 10.3389/fmicb.2018.01545 (PMC6052141; doi:10.3389/fmicb.2018.01545)
Supplement: Supplementary file 4 [file Image_1.PDF]

Figure S1. MLVA16 clustering analysis of data from this report or compiled from the literature. Data from 4971 entries with a full MLVA16 data set are shown. ‘Key’ refers to the source publication (year-first author-strain index in publication) as listed below, except for the 2018Vergnaud#XXXX keys which refer to the present report. ‘Speciesbiovar’ is the biotyping information when known. The next three columns indicate the MLVA8, MLVA11 and, when known, the MLST21 genotype. Strain, year of isolation, host and geographic origin were recovered from the publications. Clustering was done using the categorical distance coefficient and UPGMA clustering method.

The corresponding data can be queried or downloaded via the web site at http://microbesgenotyping.i2bc.paris-saclay.fr

Data source references:

Aftab, H., Dargis, R., Christensen, J.J., Le Flèche, P., and Kemp, M. (2011). Imported brucellosis in Denmark: molecular identification and multiple-locus variable number tandem repeat analysis (MLVA) genotyping of the bacteria. *Scandinavian journal of infectious diseases* 43**,** 536-538.

Al Dahouk, S., Hofer, E., Tomaso, H., Vergnaud, G., Le Flèche, P., Cloeckaert, A., Koylass, M.S., Whatmore, A.M., Nöckler, K., and Scholz, H.C. (2012). Intraspecies biodiversity of the genetically homologous species *Brucella microti*. *Appl Environ Microbiol* 78**,** 1534-1543.

Al Dahouk, S., Kohler, S., Occhialini, A., Jimenez De Bagues, M.P., Hammerl, J.A., Eisenberg, T., Vergnaud, G., Cloeckaert, A., Zygmunt, M.S., Whatmore, A.M., Melzer, F., Drees, K.P., Foster, J.T., Wattam, A.R., and Scholz, H.C. (2017). *Brucella* spp. of amphibians comprise genomically diverse motile strains competent for replication in macrophages and survival in mammalian hosts. *Sci Rep* 7**,** 44420.

Al Dahouk, S., Le Flèche, P., Nöckler, K., Jacques, I., Grayon, M., Scholz, H.C., Tomaso, H., Vergnaud, G., and Neubauer, H. (2007). Evaluation of *Brucella* MLVA typing for human brucellosis. *Journal of microbiological methods* 69**,** 137-145.

Campbell, J.I., Lan, N.P.H., Phuong, P.M., Chau, L.B., Trung Pham, D., Guzman-Verri, C., Ruiz-Villalobos, N., Minh, T.P.T., Munoz Alvaro, P.M., Moreno, E., Thwaites, G.E., Rabaa, M.A., Chau, N.V.V., and Baker, S. (2017). Human *Brucella melitensis* infections in southern Vietnam. *Clin Microbiol Infect* 23**,** 788-790.

Dorneles, E.M., Freire, G.N., Dasso, M.G., Poester, F.P., and Lage, A.P. (2014). Genetic diversity of *Brucella ovis* isolates from Rio Grande do Sul, Brazil, by MLVA16. *BMC Res Notes* 7**,** 447.

Ferreira, A.C., Chambel, L., Tenreiro, T., Cardoso, R., Flor, L., Dias, I.T., Pacheco, T., Garin-Bastuji, B., Le Flèche, P., Vergnaud, G., Tenreiro, R., and De Sá, M.I. (2012). MLVA16 typing of Portuguese human and animal *Brucella melitensis* and *Brucella abortus* isolates. *PloS one* 7**,** e42514.

Ferreira, A.C., Correa De Sa, M.I., Dias, R., and Tenreiro, R. (2017). MLVA-16 typing of *Brucella suis* biovar 2 strains circulating in Europe. *Vet Microbiol* 210**,** 77-82.

García-Yoldi, D., Le Flèche, P., De Miguel, M.J., Muñoz, P.M., Blasco, J.M., Cvetnic, Z., Marín, C.M., Vergnaud, G., and López-Goñi, I. (2007). Comparison of multiple-locus variable-number tandem-repeat analysis with other PCR-based methods for typing *Brucella suis* isolates. *J Clin Microbiol* 45**,** 4070-4072.

Garofolo, G., Ancora, M., and Di Giannatale, E. (2013a). MLVA-16 loci panel on *Brucella* spp. using multiplex PCR and multicolor capillary electrophoresis. *Journal of microbiological methods* 92**,** 103-107.

Garofolo, G., Di Giannatale, E., De Massis, F., Zilli, K., Ancora, M., Camma, C., Calistri, P., and Foster, J.T. (2013b). Investigating genetic diversity of *Brucella abortus* and *Brucella melitensis* in Italy with MLVA-16. *Infection, genetics and evolution : journal of molecular epidemiology and evolutionary genetics in infectious diseases* 19**,** 59-70.

Gyuranecz, M., Wernery, U., Kreizinger, Z., Juhász, J., Felde, O., and Nagy, P. (2016). Genotyping of *Brucella melitensis* strains from dromedary camels (Camelus dromedarius) from the United Arab Emirates with multiple-locus variable-number tandem repeat analysis. *Veterinary microbiology* 186**,** 8-12.

Hanot Mambres, D., Boarbi, S., Michel, P., Bouker, N., Escobar-Calle, L., Desqueper, D., Fancello, T., Van Esbroeck, M., Godfroid, J., Fretin, D., and Mori, M. (2017). Imported human brucellosis in Belgium: Bio and molecular typing of bacterial isolates, 1996-2015. *PLoS One* 12**,** e0174756.

Her, M., Kang, S.I., Cho, D.H., Cho, Y.S., Hwang, I.Y., Heo, Y.R., Jung, S.C., and Yoo, H.S. (2009). Application and evaluation of the MLVA typing assay for the *Brucella abortus* strains isolated in Korea. *BMC microbiology* 9**,** 230.

Her, M., Kang, S.I., Kim, J.W., Kim, J.Y., Hwang, I.Y., Jung, S.C., Park, S.H., Park, M.Y., and Yoo, H. (2010). A genetic comparison of *Brucella abortus* isolates from animals and humans by using an MLVA assay. *Journal of microbiology and biotechnology* 20**,** 1750-1755.

Hernandez-Mora, G., Ruiz-Villalobos, N., Bonilla-Montoya, R., Romero-Zuniga, J.J., Jimenez-Arias, J., Gonzalez-Barrientos, R., Barquero-Calvo, E., Chacon-Diaz, C., Rojas, N., Chaves-Olarte, E., Guzman-Verri, C., and Moreno, E. (2017). Epidemiology of bovine brucellosis in Costa Rica: Lessons learned from failures in the control of the disease. *PLoS One* 12**,** e0182380.

Isidoro-Ayza, M., Ruiz-Villalobos, N., Pérez, L., Guzmán-Verri, C., Muñoz, P.M., Alegre, F., Barberán, M., Chacón-Díaz, C., Chaves-Olarte, E., González-Barrientos, R., Moreno, E., Blasco, J.M., and Domingo, M. (2014). *Brucella ceti* infection in dolphins from the Western Mediterranean sea. *BMC Vet Res* 10**,** 206.

Jiang, H., Fan, M., Chen, J., Mi, J., Yu, R., Zhao, H., Piao, D., Ke, C., Deng, X., Tian, G., and Cui, B. (2011). MLVA genotyping of Chinese human *Brucella melitensis* biovar 1, 2 and 3 isolates. *BMC microbiology* 11**,** 256.

Jiang, H., Wang, H., Xu, L., Hu, G., Ma, J., Xiao, P., Fan, W., Di, D., Tian, G., Fan, M., Mi, J., Yu, R., Song, L., Zhao, H., Piao, D., and Cui, B. (2013). MLVA genotyping of *Brucella melitensis* and *Brucella abortus* isolates from different animal species and humans and identification of *Brucella suis* vaccine strain S2 from cattle in China. *PloS one* 8**,** e76332.

Kattar, M.M., Jaafar, R.F., Araj, G.F., Le Fleche, P., Matar, G.M., Abi Rached, R., Khalife, S., and Vergnaud, G. (2008). Evaluation of a multilocus variable-number tandem-repeat analysis scheme for typing human *Brucella* isolates in a region of brucellosis endemicity. *J Clin Microbiol* 46**,** 3935-3940.

Kiliç, S., Ivanov, I.N., Durmaz, R., Bayraktar, M.R., Ayaslioglu, E., Uyanik, M.H., Aliskan, H., Yasar, E., Bayramoglu, G., Arslantürk, A., Vergnaud, G., and Kantardjiev, T.V. (2011). Multiple-locus variable-number tandem-repeat analysis genotyping of human *Brucella* isolates from Turkey. *Journal of clinical microbiology* 49**,** 3276-3283.
Kreizinger, Z., Foster, J.T., Rónai, Z., Sulyok, K.M., Wehmann, E., Jánosi, S., and Gyuranecz, M. (2014). Genetic relatedness of *Brucella suis* biovar 2 isolates from hares, wild boars and domestic pigs. *Veterinary microbiology* 172**,** 492-498.
Le Flèche, P., Jacques, I., Grayon, M., Al Dahouk, S., Bouchon, P., Denoeud, F., Nöckler, K., Neubauer, H., Guilloteau, L.A., and Vergnaud, G. (2006). Evaluation and selection of tandem repeat loci for a *Brucella* MLVA typing assay. *BMC Microbiol* 6**,** 9.
Liu, Z.G., Di, D.D., Wang, M., Liu, R.H., Zhao, H.Y., Piao, D.R., Tian, G.Z., Fan, W.X., Jiang, H., Cui, B.Y., and Xia, X.Z. (2017). MLVA Genotyping Characteristics of Human *Brucella melitensis* Isolated from Ulanqab of Inner Mongolia, China. *Frontiers in microbiology* 8**,** 6.
Maquart, M., Le Flèche, P., Foster, G., Tryland, M., Ramisse, F., Djonne, B., Al Dahouk, S., Jacques, I., Neubauer, H., Walravens, K., Godfroid, J., Cloeckaert, A., and Vergnaud, G. (2009). MLVA-16 typing of 295 marine mammal *Brucella* isolates from different animal and geographic origins identifies 7 major groups within *Brucella ceti* and *Brucella pinnipedialis*. *BMC Microbiol* 9**,** 145.
Marianelli, C., Graziani, C., Santangelo, C., Xibilia, M.T., Imbriani, A., Amato, R., Neri, D., Cuccia, M., Rinnone, S., Di Marco, V., and Ciuchini, F. (2007). Molecular epidemiological and antibiotic susceptibility characterization of *Brucella* isolates from humans in Sicily, Italy. *Journal of clinical microbiology* 45**,** 2923-2928.
Minharro, S., Silva Mol, J.P., Dorneles, E.M., Pauletti, R.B., Neubauer, H., Melzer, F., Poester, F.P., Dasso, M.G., Pinheiro, E.S., Soares Filho, P.M., Santos, R.L., Heinemann, M.B., and Lage, A.P. (2013). Biotyping and genotyping (MLVA16) of *Brucella abortus* isolated from cattle in Brazil, 1977 to 2008. *PloS one* 8**,** e81152.
Muendo, E.N., Mbatha, P.M., Macharia, J., Abdoel, T.H., Janszen, P.V., Pastoor, R., and Smits, H.L. (2012). Infection of cattle in Kenya with *Brucella abortus* biovar 3 and *Brucella melitensis* biovar 1 genotypes. *Tropical animal health and production* 44**,** 17-20.
Nöckler, K., Maves, R., Cepeda, D., Draeger, A., Mayer-Scholl, A., Chacaltana, J., Castañeda, M., Espinosa, B., Castillo, R., Hall, E., Al Dahouk, S., Gilman, R.H., Cabeza, F., and Smits, H.L. (2009). Molecular epidemiology of *Brucella* genotypes in patients at a major hospital in central Peru. *Journal of clinical microbiology* 47**,** 3147-3155.
Rónai, Z., Kreizinger, Z., Dán, A., Drees, K., Foster, J.T., Bányai, K., Marton, S., Szeredi, L., Jánosi, S., and Gyuranecz, M. (2015). First isolation and characterization of *Brucella microti* from wild boar. *BMC veterinary research* 11**,** 147.
Scholz, H.C., Hubalek, Z., Nesvadbova, J., Tomaso, H., Vergnaud, G., Le Fleche, P., Whatmore, A.M., Al Dahouk, S., Kruger, M., Lodri, C., and Pfeffer, M. (2008). Isolation of *Brucella microti* from soil. *Emerg Infect Dis* 14**,** 1316-1317.
Scholz, H.C., Nöckler, K., Göllner, C., Bahn, P., Vergnaud, G., Tomaso, H., Al Dahouk, S., Kämpfer, P., Cloeckaert, A., Maquart, M., Zygmunt, M.S., Whatmore, A.M., Pfeffer, M., Huber, B., Busse, H.J., and De, B.K. (2010). *Brucella inopinata* sp. nov., isolated from a breast implant infection. *International journal of systematic and evolutionary microbiology* 60**,** 801-808.
Shevtsov, A., Ramanculov, E., Shevtsova, E., Kairzhanova, A., Tarlykov, P., Filipenko, M., Dymova, M., Abisheva, G., Jailbekova, A., Kamalova, D., Chsherbakov, A., Tulegenov,

S., Akhmetova, A., Sytnik, I., Karibaev, T., and Mukanov, K. (2015). Genetic diversity of *Brucella abortus* and *Brucella melitensis* in Kazakhstan using MLVA-16. *Infection, genetics and evolution : journal of molecular epidemiology and evolutionary genetics in infectious diseases* 34**,** 173-180.

Shevtsova, E., Shevtsov, A., Mukanov, K., Filipenko, M., Kamalova, D., Sytnik, I., Syzdykov, M., Kuznetsov, A., Akhmetova, A., Zharova, M., Karibaev, T., Tarlykov, P., and Ramanculov, E. (2016). Epidemiology of Brucellosis and Genetic Diversity of *Brucella abortus* in Kazakhstan. *PloS one* 11**,** e0167496.

Smits, H.L., Espinosa, B., Castillo, R., Hall, E., Guillen, A., Zevaleta, M., Gilman, R.H., Melendez, P., Guerra, C., Draeger, A., Broglia, A., and Nöckler, K. (2009). MLVA genotyping of human *Brucella* isolates from Peru. *Transactions of the Royal Society of Tropical Medicine and Hygiene* 103**,** 399-402.

Suarez-Esquivel, M., Baker, K.S., Ruiz-Villalobos, N., Hernandez-Mora, G., Barquero-Calvo, E., Gonzalez-Barrientos, R., Castillo-Zeledon, A., Jimenez-Rojas, C., Chacon-Diaz, C., Cloeckaert, A., Chaves-Olarte, E., Thomson, N.R., Moreno, E., and Guzman-Verri, C. (2017a). *Brucella* Genetic Variability in Wildlife Marine Mammals Populations Relates to Host Preference and Ocean Distribution. *Genome Biol Evol* 9**,** 1901-1912.

Suarez-Esquivel, M., Ruiz-Villalobos, N., Jimenez-Rojas, C., Barquero-Calvo, E., Chacon-Diaz, C., Viquez-Ruiz, E., Rojas-Campos, N., Baker, K.S., Oviedo-Sanchez, G., Amuy, E., Chaves-Olarte, E., Thomson, N.R., Moreno, E., and Guzman-Verri, C. (2017b). *Brucella neotomae* Infection in Humans, Costa Rica. *Emerg Infect Dis* 23**,** 997-1000.

Valdezate, S., Navarro, A., Villalón, P., Carrasco, G., and Saéz-Nieto, J.A. (2010). Epidemiological and phylogenetic analysis of Spanish human *Brucella melitensis* strains by multiple-locus variable-number tandem-repeat typing, hypervariable octameric oligonucleotide fingerprinting, and rpoB typing. *Journal of clinical microbiology* 48**,** 2734-2740.

Whatmore, A.M., Davison, N., Cloeckaert, A., Al Dahouk, S., Zygmunt, M.S., Brew, S.D., Perrett, L.L., Koylass, M.S., Vergnaud, G., Quance, C., Scholz, H.C., Dick, E.J., Jr., Hubbard, G., and Schlabritz-Loutsevitch, N.E. (2014). *Brucella papionis* sp. nov., isolated from baboons (*Papio* spp.). *International journal of systematic and evolutionary microbiology* 64**,** 4120-4128.

mlva (<All Characters>)

mlva

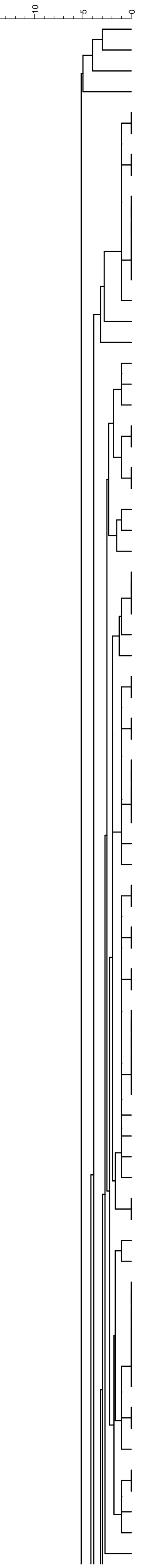

Bruce06	Bruce08	Bruce11	Bruce12	Bruce42	Bruce43	Bruce45	Bruce55	Bruce18	Bruce19	Bruce21	Bruce04	Bruce07	Bruce09	Bruce16	Bruce30	Key	speciesbiovar	MLVA8	MLVA11	MLST21	strain	host	year	isolated_in
1	5	3	13	3	2	3	2	4	43	8	8	4	8	7	3	2006LeFleche#132	B. melitensis bv2	43	122		BfR 50	Ovine	1990	Aleppo, Syria
1	5	3	13	3	2	3	2	4	43	8	9	4	3	7	6	2007AlDahouk#094	B. melitensis bv3	43	122		BfR 71	Human	2000	Greece
1	5	3	13	2	2	3	2	3	43	8	4	4	3	7	6	2007AlDahouk#092	B. melitensis bv3	42	107		BfR 68	Human	1999	Urlaub, Tyrol, Germany
1	5	3	13	3	2	3	2	4	45	8	10	4	10	4	6	2008Kattar#011	B. melitensis	43	123		AUB BRUP-S21	Human		Lebanon
1	5	3	13	3	2	3	2	4	9	8	5	4	4	4	3	2018Vergnaud#1184	B. melitensis bv1	43	292		BCCN#86-52	Ovine	1986	Saragosse, Spain
1	5	3	13	3	2	3	2	4	9	8	5	4	4	4	3	2018Vergnaud#1185	B. melitensis bv1	43	292		BCCN#86-53	Ovine	1986	Saragosse, Spain
1	5	3	13	3	2	3	2	4	9	8	5	4	4	4	5	2018Vergnaud#0731	B. melitensis bv1	43	292		BCCN#86-27	Ovine	1986	Saragosse, Spain
1	5	3	13	3	2	3	2	4	9	8	5	4	4	4	5	2018Vergnaud#0917	B. melitensis bv1	43	292		BCCN#86-47	Ovine	1986	Saragosse, Spain
1	5	3	13	3	2	3	2	4	9	8	5	4	4	4	7	2018Vergnaud#0446	B. melitensis bv1	43	292		BCCN#86-26	Ovine	1986	Saragosse, Spain
1	5	3	13	3	2	3	2	4	9	8	5	4	4	4	7	2018Vergnaud#0916	B. melitensis bv1	43	292		BCCN#86-46	Ovine	1986	Saragosse, Spain
1	5	3	13	3	2	3	2	4	9	8	5	4	4	4	7	2018Vergnaud#0934	B. melitensis bv1	43	292		BCCN#86-28	Ovine	1986	Saragosse, Spain
1	5	3	13	3	2	3	2	4	9	8	5	4	4	4	7	2018Vergnaud#0953	B. melitensis bv1	43	292		BCCN#86-48	Ovine	1986	Saragosse, Spain
1	5	3	13	3	2	3	2	4	9	8	5	4	4	4	7	2018Vergnaud#0954	B. melitensis bv1	43	292		BCCN#86-49	Ovine	1986	Saragosse, Spain
1	5	3	13	3	2	3	2	4	9	8	5	4	4	4	6	2018Vergnaud#1107	B. melitensis bv1	43	292		BCCN#86-51	Ovine	1986	Saragosse, Spain
1	5	3	13	3	2	3	2	4	49	8	5	4	5	4	5	2008Kattar#012	B. melitensis	43	124		AUB BRUP-S22	Human		Lebanon
1	5	3	13	3	2	3	2	4	9	8	5	6	4	7	8	2018Vergnaud#0447	B. melitensis bv1	43	292		BCCN#86-44	Ovine	1986	Saragosse, Spain
1	5	3	13	3	2	3	2	5	9	8	4	4	3	4	6	2018Vergnaud#0774	B. melitensis bv1	43	105		BCCN#92-11	Ovine	1992	Somme, France
1	5	3	13	3	2	3	2	5	9	8	7	4	3	4	6	2018Vergnaud#1108	B. melitensis bv1	43	105		BCCN#94-58	Human	1994	Loire-Atlantique, France
1	5	3	13	3	2	3	2	5	9	8	5	4	3	4	6	2018Vergnaud#0877	B. melitensis bv1	43	105		BCCN#77-144	Cattle	1977	Navarre, Spain
1	5	3	13	3	2	3	2	5	9	8	3	4	3	5	6	2018Vergnaud#0768	B. melitensis bv1	43	105		BCCN#82-54	Human	1982	Paris, France
1	5	3	13	3	2	3	2	5	9	8	3	4	3	5	6	2018Vergnaud#0804	B. melitensis bv1	43	105		BCCN#82-55	Human	1982	Paris, France
1	5	3	13	3	2	3	2	5	9	8	4	4	3	5	6	2018Vergnaud#0472	B. melitensis bv1	43	105		BCCN#91-288	Cattle	1991	Somme, France
1	5	3	13	3	2	3	2	5	9	8	4	4	3	5	6	2018Vergnaud#0775	B. melitensis bv1	43	105		BCCN#92-19	Cattle	1992	Somme, France
1	5	3	13	3	2	3	2	4	9	8	4	4	3	5	8	2018Vergnaud#0093	B. melitensis bv1	43	292		BCCN#78-146	Ovine	1978	Haute-Vienne, France
1	5	3	13	3	2	3	2	5	9	8	4	4	3	5	8	2018Vergnaud#0467	B. melitensis bv1	43	105		BCCN#91-263	Cattle	1991	Somme, France
1	5	3	13	3	2	3	2	5	9	8	4	4	3	5	9	2018Vergnaud#0456	B. melitensis bv1	43	105		BCCN#89-70	Cattle	1989	Somme, France
1	5	3	13	3	2	3	2	5	9	8	6	4	3	4	5	2018Vergnaud#0041	B. melitensis bv1	43	105		BCCN#80-163	Human	1980	Spain
1	5	3	13	3	2	3	2	5	9	8	6	4	3	4	5	2018Vergnaud#0087	B. melitensis bv1	43	105		BCCN#77-94	Human	1977	Navarre, Spain
1	5	3	13	3	2	3	2	5	9	8	6	4	3	4	5	2018Vergnaud#0454	B. melitensis bv1	43	105		BCCN#89-136	Ovine	1989	Saragosse, Spain
1	5	3	13	3	2	3	2	5	9	8	6	4	3	4	4	2018Vergnaud#0733	B. melitensis bv1	43	105		BCCN#92-89	Human	1992	Navarre, Spain
1	5	3	13	3	2	3	2	5	9	8	6	4	3	5	5	2018Vergnaud#0089	B. melitensis bv1	43	105		BCCN#77-152	Cattle	1977	Navarre, Spain
1	5	3	13	3	2	3	2	5	9	8	5	4	3	6	5	2018Vergnaud#0088	B. melitensis bv1	43	105		BCCN#77-146	Human	1977	Navarre, Spain
1	5	3	13	3	2	3	2	5	9	8	5	4	3	6	5	2018Vergnaud#0915	B. melitensis bv1	43	105		BCCN#77-4	Human	1977	Pampelune, Spain
1	5	3	13	3	2	3	2	5	9	8	5	4	3	4	5	2018Vergnaud#0076	B. melitensis bv1	43	105		BCCN#74-266	Human	1974	Navarre, Spain
1	5	3	13	3	2	3	2	5	9	8	5	4	3	4	5	2018Vergnaud#1282	B. melitensis bv1	43	105		BCCN#94-66	Human	1994	Hautes-Pyrénées, France
1	5	3	13	3	2	3	2	5	9	8	5	4	3	5	5	2018Vergnaud#0026	B. melitensis bv1	43	105		BCCN#82-91	Ovine	1982	France
1	5	3	13	3	2	3	2	5	9	8	5	4	3	5	5	2018Vergnaud#0211	B. melitensis R.	43	105		BCCN#88-3	Human	1988	Pampelune, Spain
1	5	3	13	3	2	3	2	5	9	8	5	4	3	5	5	2018Vergnaud#0212	B. melitensis R.	43	105		BCCN#88-4	Human	1988	Pampelune, Spain
1	5	3	13	3	2	3	2	5	9	8	5	4	3	5	5	2018Vergnaud#0452	B. melitensis bv1	43	105		BCCN#88-5	Human	1988	Pampelune, Spain
1	5	3	13	3	2	3	2	5	9	8	5	4	3	9	5	2018Vergnaud#0349	B. melitensis R.	43	105		BCCN#76-325	Human	1976	Strasbourg ,Bas-Rhin, .
1	5	3	13	3	2	3	2	5	9	8	5	4	3	7	5	2018Vergnaud#0474	B. melitensis bv1	43	105		BCCN#92-84	Ovine	1992	Murcie ,Spain
1	5	3	13	3	2	3	2	5	9	8	4	4	3	11	5	2018Vergnaud#1373	B. melitensis bv1	43	105		BCCN#95-40	Human	1995	Haut-Rhin, France
1	5	3	13	3	2	3	2	5	9	8	4	4	3	11	5	2018Vergnaud#1403	B. melitensis bv1	43	105		BCCN#96-133	Human	1996	Ain, France
1	5	3	13	3	2	3	2	5	9	8	4	4	3	10	5	2018Vergnaud#0890	B. melitensis bv1	43	105		BCCN#95-52	Human	1995	Haut-Rhin, France
1	5	3	13	3	2	3	2	5	9	8	4	4	3	10	5	2018Vergnaud#1375	B. melitensis bv1	43	105		BCCN#96-18	Human	1996	Bas-Rhin, France
1	5	3	13	3	2	3	2	5	9	8	4	4	3	6	5	2018Vergnaud#0449	B. melitensis bv1	43	105		BCCN#86-92	Ovine	1986	Saragosse, Spain
1	5	3	13	3	2	3	2	5	9	8	4	4	3	6	5	2018Vergnaud#0450	B. melitensis bv1	43	105		BCCN#86-93	Ovine	1986	Saragosse, Spain
1	5	3	13	3	2	3	2	5	9	8	4	4	3	4	5	2006LeFleche#085	B. melitensis bv2	43	105		BCCN#92-87	Ovine	1992	Aragon, Spain
1	5	3	13	3	2	3	2	5	9	8	4	4	3	4	5	2018Vergnaud#0347	B. melitensis bv2	43	105		BCCN#93-91	Ovine	1993	Saragosse ,Spain
1	5	3	13	3	2	3	2	5	9	8	4	4	3	4	5	2018Vergnaud#0348	B. melitensis bv2	43	105		BCCN#93-92	Ovine	1993	Spain
1	5	3	13	3	2	3	2	5	9	8	4	4	3	4	5	2018Vergnaud#0350	B. melitensis R.	43	105		BCCN#76-334	Human	1976	Clermont-Ferrand ,Puy.
1	5	3	13	3	2	3	2	5	9	8	4	4	3	4	5	2018Vergnaud#1028	B. melitensis bv1	43	105		BCCN#74-260	Human	1974	Paris, France
1	5	3	13	3	2	3	2	5	9	8	4	4	3	9	5	2018Vergnaud#0351	B. melitensis R.	43	105		BCCN#76-365	Human	1976	Strasbourg ,Bas-Rhin, .
1	5	3	13	3	2	3	2	5	9	8	4	4	3	8	5	2018Vergnaud#0732	B. melitensis bv1	43	105		BCCN#92-48	Cattle	1992	Meuse, France
1	5	3	13	3	2	3	2	5	9	8	4	4	3	5	5	2018Vergnaud#1368	B. melitensis bv1	43	105		BCCN#85-22	Human	1985	Nouans le Fuselier ,Loir.
1	5	3	13	3	2	3	2	5	9	8	4	4	3	12	5	2018Vergnaud#1374	B. melitensis bv1	43	105		BCCN#95-9	Human	1995	Bas-Rhin, France
1	5	3	13	3	2	3	2	5	9	8	4	5	3	4	5	2018Vergnaud#0957	B. melitensis bv1	43	105		BCCN#95-57	Cattle	1995	Navarre, Spain
1	5	3	13	3	2	3	2	5	9	8	4	5	3	4	5	2018Vergnaud#0958	B. melitensis bv1	43	105		BCCN#95-58	Cattle	1995	Navarre, Spain
1	5	3	13	3	2	3	2	5	9	8	3	4	3	5	7	2018Vergnaud#0056	B. melitensis bv1	43	105		BCCN#80-276	Human	1980	Somme, France
1	5	3	13	3	2	3	2	5	9	8	3	4	3	8	7	2018Vergnaud#0453	B. melitensis bv1	43	105		BCCN#88-52	Cattle	1988	Haute-Marne, France
1	5	3	13	3	2	3	2	5	9	8	3	4	3	5	5	2007AlDahouk#023	B. melitensis	43	105		BCCN#94-62	Human	1994	Agen, France
1	5	3	13	3	2	3	2	5	9	8	3	4	3	5	5	2018Vergnaud#0431	B. melitensis bv1	43	105		BCCN#75-204	Ovine	1975	Alpes-Maritimes, France
1	5	3	13	3	2	3	2	5	9	8	3	4	3	5	5	2018Vergnaud#0771	B. melitensis bv1	43	105		BCCN#87-22	Cattle	1987	Woustviller ,Moselle, F.
1	5	3	13	3	2	3	2	5	9	8	3	4	3	5	5	2018Vergnaud#0886	B. melitensis bv1	43	105		BCCN#75-1	Human	1975	France
1	5	3	13	3	2	3	2	5	9	8	3	4	3	5	5	2018Vergnaud#0935	B. melitensis bv1	43	105		BCCN#87-18	Cattle	1987	Woustviller ,Moselle, F.
1	5	3	13	3	2	3	2	5	9	8	3	4	3	5	5	2018Vergnaud#1032	B. melitensis bv1	43	105		BCCN#87-21	Cattle	1987	Woustviller ,Moselle, F.
1	5	3	13	3	2	3	2	5	9	8	3	4	3	7	5	2006LeFleche#129	B. melitensis bv2	43	105		BfR 43	unknown	1972	
1	5	3	13	3	2	3	2	5	9	8	3	4	3	7	5	2018Vergnaud#0057	B. melitensis bv1	43	105		BCCN#81-283	Ovine	1981	Argenton Chateau ,Deu.
1	5	3	13	3	2	3	2	5	9	8	3	4	3	4	5	2018Vergnaud#0029	B. melitensis bv1	43	105		BCCN#83-218	Human	1983	France
1	5	3	13	3	2	3	2	5	9	8	3	4	3	6	4	2018Vergnaud#0047	B. melitensis bv1	43	105		BCCN#86-6	Ovine	1986	Somme, France
1	5	3	13	3	2	3	2	5	9	8	3	4	3	6	4	2018Vergnaud#1030	B. melitensis bv1	43	105		BCCN#82-10	Human	1982	Somme, France
1	5	3	13	3	2	3	2	5	9	8	3	4	3	6	5	2006LeFleche#130	B. melitensis bv2	43	105		BfR 44	unknown	1972	
1	5	3	13	3	2	3	2	5	9	8	3	4	3	6	6	2018Vergnaud#0805	B. melitensis bv1	43	105		BCCN#91-159	Ovine	1991	Somme, France
1	5	3	14	3	2	3	2	5	9	8	3	4	3	4	5	2018Vergnaud#0872	B. melitensis bv1	61	287		BCCN#76-337	Human	1976	Montpellier ,Hérault, Fr.

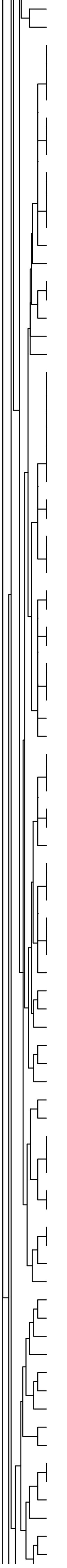

1	5	3	13	3	2	3	2	5	9	8	4	4	3	6	2	2018Vergnaud#0451	B. melitensis bv1	43	105	BCCN#86-94	Ovine	1986	Saragosse, Spain
1	5	3	13	3	2	3	2	5	9	8	4	6	3	6	7	2018Vergnaud#0477	B. melitensis bv1	43	105	BCCN#95-56	Cattle	1995	Navarre, Spain
1	5	3	13	3	2	3	2	4	9	8	8	4	3	5	5	2018Vergnaud#0457	B. melitensis bv1	43	292	BCCN#89-73	Ovine	1989	Pyrénées-Atlantiques, .
1	5	3	13	3	2	3	2	4	9	8	8	4	3	5	5	2018Vergnaud#0920	B. melitensis bv1	43	292	BCCN#97-53	Human	1997	Pyrénées-Atlantiques, .
1	5	3	13	3	2	3	2	4	9	8	8	4	3	5	5	2018Vergnaud#1370	B. melitensis bv1	43	292	BCCN#94-1	Human	1994	Loiret, France
1	5	3	13	3	2	3	2	4	9	8	8	4	3	5	5	2018Vergnaud#1371	B. melitensis bv1	43	292	BCCN#95-14	Human	1995	Pyrénées-Atlantiques, .
1	5	3	13	3	2	3	2	4	9	8	4	4	3	5	5	2018Vergnaud#0077	B. melitensis bv1	43	292	BCCN#74-267	Human	1974	Navarre, Spain
1	5	3	13	3	2	3	2	4	9	8	4	4	3	5	5	2018Vergnaud#0595	B. melitensis bv3	43	292	BCCN#84-4	Human	1984	Spain
1	5	3	13	3	2	3	2	4	9	8	4	4	3	5	5	2018Vergnaud#0801	B. melitensis bv1	43	292	BCCN#76-307	Human	1976	Navarre, Spain
1	5	3	13	3	2	3	2	4	9	8	5	4	3	5	5	2018Vergnaud#0071	B. melitensis bv1	43	292	BCCN#74-237	Human	1974	Paris, France
1	5	3	13	3	2	3	2	4	9	8	5	4	3	5	5	2018Vergnaud#0073	B. melitensis bv1	43	292	BCCN#74-239	Human	1974	Paris, France
1	5	3	13	3	2	3	2	4	9	8	5	4	3	5	5	2018Vergnaud#0081	B. melitensis bv1	43	292	BCCN#74-271	Human	1974	Navarre, Spain
1	5	3	13	3	2	3	2	4	9	8	5	4	3	5	5	2018Vergnaud#0765	B. melitensis bv1	43	292	BCCN#75-3	Human	1975	Navarre, France
1	5	3	13	3	2	3	2	4	9	8	6	4	3	5	5	2018Vergnaud#0045	B. melitensis bv1	43	292	BCCN#84-11	Caprine	1984	Spain
1	5	3	13	3	2	3	2	4	36	8	5	4	3	5	5	2011Kilic#150	B. melitensis bv3	43	121	BRU-S150	Human	2005	Malatya, Turkey
1	5	3	13	3	2	3	2	4	9	8	4	4	3	5	7	2018Vergnaud#0043	B. melitensis bv1	43	292	BCCN#80-86	Ovine	1980	Haute-Vienne, France
1	5	3	13	3	2	3	2	4	9	8	4	4	3	5	7	2018Vergnaud#0439	B. melitensis bv1	43	292	BCCN#78-158	Ovine	1978	Haute-Vienne, France
1	5	3	13	3	2	3	2	4	9	8	5	4	3	5	7	2018Vergnaud#0012	B. melitensis bv2	43	292	BCCN#76-407	Human	1976	Navarre, Spain
1	5	3	14	3	2	3	2	4	9	8	4	4	3	5	5	2018Vergnaud#0475	B. melitensis bv1	61	288	BCCN#92-88	Human	1992	Navarre, Spain
1	5	3	13	3	2	3	2	4	9	8	4	5	3	5	5	2018Vergnaud#0875	B. melitensis bv1	43	292	BCCN#76-408	Human	1976	Navarre, Spain
1	5	3	13	3	2	3	2	4	9	8	7	4	3	4	5	2018Vergnaud#0485	B. melitensis bv1	43	292	BCCN#97-118	Caprine	1997	Hauts-de-Seine, France
1	5	3	13	3	2	3	2	4	9	8	7	4	3	4	5	2018Vergnaud#0921	B. melitensis bv1	43	292	BCCN#97-54	Human	1997	Gironde, France
1	5	3	13	3	2	3	2	4	9	8	7	4	3	4	5	2018Vergnaud#0992	B. melitensis bv1	43	292	BCCN#97-47	Human	1997	Indre-et-Loire, France
1	5	3	13	3	2	3	2	4	9	8	7	4	3	4	5	2018Vergnaud#0993	B. melitensis bv1	43	292	BCCN#97-48	Human	1997	Pessac ,Gironde, France
1	5	3	13	3	2	3	2	4	9	8	7	4	3	4	5	2018Vergnaud#1147	B. melitensis bv1	43	292	BCCN#97-55	Human	1997	St Denis de Vil ,Gironde.
1	5	3	13	3	2	3	2	4	9	8	7	4	3	4	5	2018Vergnaud#1148	B. melitensis bv1	43	292	BCCN#97-56	Human	1997	Parthenay ,Deux-Sèvres.
1	5	3	13	3	2	3	2	4	9	8	7	4	3	4	5	2018Vergnaud#1376	B. melitensis bv1	43	292	BCCN#97-39	Human	1997	Maine-et-Loire, France
1	5	3	13	3	2	3	2	4	9	8	4	4	3	4	5	2018Vergnaud#0458	B. melitensis bv1	43	292	BCCN#89-84	Ovine	1989	Saragosse, Spain
1	5	3	13	3	2	3	2	4	9	8	4	4	3	4	5	2018Vergnaud#0952	B. melitensis bv1	43	292	BCCN#77-148	Human	1977	Navarre, Spain
1	5	3	13	3	2	3	2	4	9	8	5	4	3	4	5	2018Vergnaud#0055	B. melitensis bv1	43	292	BCCN#80-229	Human	1980	Haute-Garonne, France
1	5	3	13	3	2	3	2	4	9	8	5	4	3	4	5	2018Vergnaud#0061	B. melitensis bv1	43	292	BCCN#74-264	Human	1974	Navarre, Spain
1	5	3	13	3	2	3	2	4	9	8	5	4	3	4	5	2018Vergnaud#0991	B. melitensis bv1	43	292	BCCN#91-62	Ovine	1991	Huesca, Spain
1	5	3	13	3	2	3	2	4	9	8	3	4	3	4	5	2018Vergnaud#0937	B. melitensis bv1	43	292	BCCN#92-3	Cattle	1992	Navarre, Spain
1	5	3	13	3	2	3	2	4	9	8	3	4	3	4	5	2018Vergnaud#1038	B. melitensis bv1	43	292	BCCN#98-15	Human	1998	Navarre, Spain
1	5	3	13	3	2	3	2	4	9	8	3	4	3	8	5	2018Vergnaud#0880	B. melitensis bv1	43	292	BCCN#95-26	Cattle	1995	Navarre, Spain
1	5	3	13	3	2	3	2	4	9	8	3	4	3	8	5	2018Vergnaud#0881	B. melitensis bv1	43	292	BCCN#95-27	Cattle	1995	Navarre, Spain
1	5	3	13	3	2	3	2	4	9	8	3	4	3	7	5	2018Vergnaud#0466	B. melitensis bv1	43	292	BCCN#91-234	Ovine	1991	Navarre, Spain
1	5	3	13	3	2	3	2	4	9	8	3	4	3	7	5	2018Vergnaud#0806	B. melitensis bv1	43	292	BCCN#91-235	Ovine	1991	Navarre, Spain
1	5	3	13	3	2	3	2	4	9	8	3	4	3	7	5	2018Vergnaud#0878	B. melitensis bv1	43	292	BCCN#91-236	Ovine	1991	Navarre, Spain
1	5	3	13	3	2	3	2	4	9	8	3	4	3	9	5	2018Vergnaud#0108	B. melitensis bv1	43	292	BCCN#94-54	Cattle	1994	Pyrénées-Atlantiques, .
1	5	3	13	3	2	3	2	4	9	8	3	4	3	5	5	2018Vergnaud#0773	B. melitensis bv1	43	292	BCCN#91-52	Ovine	1991	Saragosse, Spain
1	5	3	13	3	2	3	2	4	9	8	5	4	3	5	6	2018Vergnaud#0039	B. melitensis bv1	43	292	BCCN#75-77	Human	1975	Navarre, Spain
1	5	3	13	3	2	3	2	4	9	8	5	4	3	5	6	2018Vergnaud#0802	B. melitensis bv1	43	292	BCCN#76-309	Human	1976	Navarre, Spain
1	5	3	13	3	2	3	2	4	9	8	5	4	3	5	6	2018Vergnaud#1164	B. melitensis bv1	43	292	BCCN#75-209	Human	1975	Navarre, Spain
1	5	3	13	3	2	3	2	4	9	8	5	4	3	8	6	2018Vergnaud#0037	B. melitensis bv1	43	292	BCCN#75-24	Human	1975	Navarre, Spain
1	5	3	13	3	2	3	2	4	9	8	5	4	3	8	6	2018Vergnaud#0083	B. melitensis bv1	43	292	BCCN#75-208	Human	1975	Navarre, Spain
1	5	3	13	3	2	3	2	4	9	8	5	4	3	6	6	2018Vergnaud#0990	B. melitensis bv1	43	292	BCCN#91-61	Ovine	1991	Huesca, Spain
1	5	3	13	3	2	3	2	4	9	8	4	4	3	4	6	2018Vergnaud#0933	B. melitensis bv1	43	292	BCCN#75-5	Human	1975	Navarre, Spain
1	5	3	13	3	2	3	2	4	9	8	4	4	3	4	6	2018Vergnaud#0955	B. melitensis bv1	43	292	BCCN#91-35	Ovine	1991	Saragosse, Spain
1	5	3	13	3	2	3	2	4	9	8	4	4	3	4	6	2018Vergnaud#0956	B. melitensis bv1	43	292	BCCN#91-36	Ovine	1991	Saragosse, Spain
1	5	3	13	3	2	3	2	4	9	8	5	4	3	4	6	2018Vergnaud#0914	B. melitensis bv1	43	292	BCCN#77-2	Human	1977	Pampelune, Spain
1	5	3	13	3	2	3	2	4	9	8	5	4	3	4	6	2018Vergnaud#0951	B. melitensis bv1	43	292	BCCN#77-147	Human	1977	Navarre, Spain
1	5	3	13	3	2	3	2	4	9	8	5	4	3	4	6	2018Vergnaud#1365	B. melitensis bv1	43	292	BCCN#76-420	Human	1976	Navarre, Spain
1	5	3	13	3	2	3	2	4	9	8	6	4	3	4	6	2018Vergnaud#0455	B. melitensis bv1	43	292	BCCN#89-42	Cattle	1989	Pyrénées-Atlantiques, .
1	5	3	13	3	2	3	2	4	9	8	6	4	3	7	6	2018Vergnaud#0463	B. melitensis bv1	43	292	BCCN#91-227	Ovine	1991	Saragosse, Spain
1	5	3	13	3	2	3	2	4	9	8	4	4	3	7	6	2018Vergnaud#0936	B. melitensis bv1	43	292	BCCN#90-13	Ovine	1990	Saragosse, Spain
1	5	3	13	3	2	3	2	4	9	8	4	4	3	8	6	2018Vergnaud#0769	B. melitensis bv1	43	292	BCCN#82-57	Human	1982	Madrid ,Spain
1	5	3	13	3	2	3	2	4	9	8	5	4	3	4	8	2018Vergnaud#0079	B. melitensis bv1	43	292	BCCN#74-269	Human	1974	Navarre, Spain
1	5	3	13	3	2	3	2	4	9	8	5	4	3	4	7	2018Vergnaud#1106	B. melitensis bv1	43	292	BCCN#86-50	Ovine	1986	Saragosse, Spain
1	5	3	13	3	2	3	2	4	9	8	5	4	3	6	8	2018Vergnaud#0473	B. melitensis bv1	43	292	BCCN#91-29	Ovine	1991	Saragosse, Spain
1	5	3	13	3	2	3	2	4	9	8	7	4	3	6	5	2018Vergnaud#1034	B. melitensis bv1	43	292	BCCN#91-240	Ovine	1991	Saragosse, Spain
1	5	3	13	3	2	3	2	4	9	8	7	4	3	6	7	2018Vergnaud#1366	B. melitensis bv1	43	292	BCCN#78-75	Human	1978	Navarre, Spain
1	5	3	13	3	2	3	2	4	9	8	4	4	3	6	5	2018Vergnaud#0040	B. melitensis bv1	43	292	BCCN#75-127	Human	1975	Navarre, Spain
1	5	3	13	3	2	3	2	4	9	8	4	4	3	6	5	2018Vergnaud#0091	B. melitensis bv1	43	292	BCCN#78-76	Human	1978	Navarre, Spain
1	5	3	13	3	2	3	2	4	9	8	4	4	3	6	5	2018Vergnaud#0210	B. melitensis R.	43	292	BCCN#82-71	Human	1982	Barcelone ,Spain
1	5	3	13	3	2	3	2	4	9	8	4	4	4	6	5	2018Vergnaud#0038	B. melitensis bv1	43	292	BCCN#75-75	Human	1975	Navarre, Spain
1	5	3	13	3	2	3	2	4	9	8	4	4	4	6	5	2018Vergnaud#0766	B. melitensis bv1	43	292	BCCN#76-310	Human	1976	Navarre, Spain
1	5	3	13	3	2	3	2	4	9	8	5	3	3	6	5	2018Vergnaud#0448	B. melitensis bv1	43	292	BCCN#86-84	Ovine	1986	Saragosse, Spain
1	5	3	13	3	2	3	2	4	9	8	5	3	3	6	5	2018Vergnaud#0471	B. melitensis bv1	43	292	BCCN#86-85	Ovine	1986	Saragosse, Spain
1	5	3	13	3	2	3	2	4	9	8	4	3	3	6	5	2018Vergnaud#0479	B. melitensis bv1	43	292	BCCN#96-117	Cattle	1996	Navarre, Spain
1	5	3	13	3	2	3	2	4	9	8	4	3	3	6	6	2018Vergnaud#0036	B. melitensis bv1	43	292	BCCN#75-23	Human	1975	Navarre, Spain
1	5	3	13	3	2	2	2	5	9	8	4	4	3	4	5	2018Vergnaud#0046	B. melitensis bv1	60	286	BCCN#85-7	Human	1985	Portugal
1	5	3	13	3	2	2	2	6	9	8	4	4	3	4	5	2018Vergnaud#1105	B. melitensis bv1	60	99	BCCN#75-292	Human	1975	Castille, Spain
1	5	3	13	3	2	3	2	6	9	8	4	4	3	4	5	2018Vergnaud#0812	B. melitensis bv1	43	283	BCCN#76-330	Human	1976	Le Puy ,Haute-Loire, Fr.
1	5	3	13	3	2	2	2	5	9	8	4	4	3	5	5	2018Vergnaud#0873	B. melitensis bv1	60	286	BCCN#76-338	Human	1976	Montpellier ,Hérault, Fr.
1	5	3	13	3	2	2	2	6	9	8	4	4	3	6	6	2007AlDahouk#010	B. melitensis bv2	60	99	BCCN#96-3	Human	1996	Tarbes, France
1	5	3	13	3	2	2	2	6	9	8	4	4	3	6	5	2007AlDahouk#062	B. melitensis bv1	60	99	BfR 28	Human	2000	Bern, Switzerland
1	5	3	13	3	2	2	2	6	9	8	4	4	21	6	5	2018Vergnaud#0353	B. melitensis R.	60	99	BCCN#83-21	Human	1983	France
1	5	3	13	3	2	2	2	6	9	8	5	5	3	4	5	2018Vergnaud#0025	B. melitensis bv1	60	99	BCCN#81-23	Human	1981	Versailles ,Yvelines, Fr.
1	5	3	13	3	2	2	2	6	9	8	5	4	3	4	5	2018Vergnaud#0084	B. melitensis bv1	60	99	BCCN#75-309	Human	1975	Castille, Spain
1	5	3	13	3	2	2	2	6	9	8	7	4	3	5	6	2007AlDahouk#005	B. melitensis bv1	60	99	BCCN#93-2	Human	1993	Poitiers, France
1	5	3	13	3	2	2	2	6	9	8	7	4	3	5	6	2018Vergnaud#1035	B. melitensis bv1	60	99	BCCN#93-11	Human	1993	Vienne, France
1	5	3	13	3	2	2	2	6	9	8	5	4	3	5	6	2018Vergnaud#1257	B. melitensis bv1	60	99	BCCN#75-296	Human	1975	Castille, Spain
1	5	3	13	3	2	2	2	6	9	8	6	4	3	5	5	2018Vergnaud#0879	B. melitensis bv1	60	99	BCCN#91-239	Ovine	1991	Leon, Spain
1	5	3	13	3	2	2	2	6	9	8	6	4	3	4	6	2018Vergnaud#0030	B. melitensis bv1	60	99	BCCN#84-6	Human	1984	Portugal
1	5	3	13	3	2	2	2	6	9	8	6	4	3	4	7	2018Vergnaud#0918	B. melitensis bv1	60	99	BCCN#91-31	Ovine	1991	Saragosse, Spain

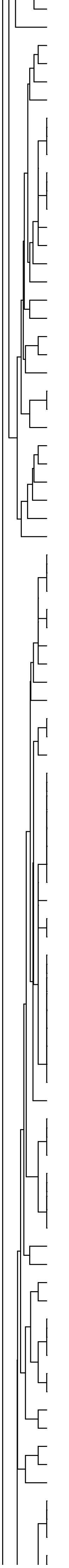

1	5	3	13	3	2	2	2	6	9	8	7	4	3	4	7	2018Vergnaud#0919	B. melitensis bv1	60	99		BCCN#91-32	Ovine	1991	Saragosse, Spain
1	5	3	13	3	2	2	2	4	9	8	5	4	3	5	4	2018Vergnaud#1031	B. melitensis bv1	60	285		BCCN#86-45	Ovine	1986	Saragosse, Spain
1	5	3	13	3	2	3	2	5	36	8	4	4	3	5	6	2007AlDahouk#031	B. melitensis bv2	43	104		BfR 6	Human	2002	Turkey
1	5	3	13	3	2	3	2	5	36	8	4	4	3	5	4	2007AlDahouk#069	B. melitensis bv2	43	104		BfR 36	Human	2000	Berlin, Germany
1	5	3	13	3	2	3	2	5	36	8	4	4	3	6	6	2007AlDahouk#102	B. melitensis bv3	43	104		BfR 81	Human	1998	
1	5	3	13	3	2	3	2	5	36	8	5	4	3	5	4	SRR4436618	B. melitensis	43	104		BwIM_BGR_21	Human	2017	Bulgaria
1	5	3	13	3	2	3	2	5	36	8	5	4	3	6	6	2018Vergnaud#0400	B. melitensis bv3	43	104		BCCN#91-100	Cattle	1991	Salonique ,Greece
1	5	3	13	3	2	3	2	5	36	8	5	4	3	6	6	2018Vergnaud#0617	B. melitensis bv3	43	104		BCCN#91-99	Cattle	1991	Salonique ,Greece
1	5	3	13	3	2	3	2	5	36	8	5	4	3	6	6	2018Vergnaud#0835	B. melitensis bv3	43	104		BCCN#91-111	Cattle	1991	Salonique ,Greece
1	5	3	13	3	2	3	2	5	36	8	5	4	3	7	6	2011Kilic#052	B. melitensis bv3	43	104		BRU-S052	Human	2008	Kutahya, Turkey
1	5	3	13	3	2	3	2	5	36	8	5	4	3	7	6	2018Vergnaud#0536	B. melitensis bv3	43	104		BCCN#83-191	Caprine	1983	Greece
1	5	3	13	3	2	3	2	5	36	8	5	4	3	7	6	2018Vergnaud#0565	B. melitensis bv3	43	104		BCCN#83-192	Caprine	1983	Greece
1	5	3	13	3	2	3	2	5	36	8	5	4	3	13	6	2011Aftab#007	B. melitensis	43	104		BD31509-00	Human		Turkey
1	5	3	13	3	2	3	2	5	36	8	5	4	3	8	6	2017Hanot-Mamb.	B. melitensis bv3	43	104		L3/10	Human	1997	Belgium
1	5	3	13	3	2	3	2	5	36	8	5	4	3	6	5	2018Vergnaud#0137	B. melitensis bv3	43	104		BCCN#04-2	Human	2004	Turkey
1	5	3	13	3	2	3	2	5	36	8	6	4	3	8	6	2011Aftab#008	B. melitensis	43	104		BD33436-99	Human		Turkey
1	5	3	13	3	2	3	2	4	36	8	5	4	3	3	6	2011Aftab#014	B. melitensis	43	121		H80446	Human		Turkey
1	5	3	13	3	2	3	2	4	36	8	6	4	3	6	6	2011Kilic#154	B. melitensis bv3	43	121		BRU-S154	Human	2003	Malatya, Turkey
1	5	3	13	3	2	3	2	5	36	8	5	4	3	4	8	2011Kilic#055	B. melitensis bv3	43	104		BRU-S055	Human	2008	Kutahya, Turkey
1	5	3	13	3	2	3	2	5	36	8	6	4	3	4	8	SRR4436597	B. melitensis	43	104		BwIM_TUR_52	Human	2017	Turkey
1	5	3	13	3	2	3	2	5	36	8	6	4	3	9	4	2011Kilic#092	B. melitensis bv3	43	104		BRU-S092	Human	2008	Tirebolu, Turkey
1	5	3	13	3	2	3	2	5	36	8	9	4	3	5	5	2011Kilic#051	B. melitensis bv3	43	104		BRU-S051	Human	2008	Kutahya, Turkey
1	5	3	13	3	2	3	2	5	36	8	9	4	3	5	5	2011Kilic#053	B. melitensis bv3	43	104		BRU-S053	Human	2008	Kutahya, Turkey
1	5	3	13	3	2	3	2	5	36	8	5	4	6	5	5	2018Vergnaud#0377	B. melitensis bv3	43	104		BCCN#04-4	Human	2004	Turkey
1	5	3	14	3	2	3	2	5	36	8	6	4	3	5	6	2007AlDahouk#037	B. melitensis bv2	61	103		BfR 13	Human	2002	Turkey
1	5	3	14	3	2	3	2	5	36	8	7	4	3	5	6	2011Kilic#071	B. melitensis bv3	61	103		BRU-S071	Human	2004	Kirikkale, Turkey
1	5	3	14	3	2	3	2	5	36	8	6	4	3	6	6	2011Kilic#086	B. melitensis bv3	61	103		BRU-S086	Human	2007	Trabzon, Turkey
1	5	3	14	3	2	3	2	5	36	8	7	4	3	7	6	2011Kilic#087	B. melitensis bv3	61	103		BRU-S087	Human	2007	Bayburt, Turkey
1	5	3	14	3	2	3	2	4	36	8	7	4	3	6	6	2011Kilic#084	B. melitensis bv3	61	102		BRU-S084	Human	2006	Trabzon, Turkey
1	5	3	14	3	2	3	2	5	36	8	8	4	3	7	7	2011Kilic#088	B. melitensis bv3	61	103		BRU-S088	Human	2007	Gumushane, Turkey
1	5	3	13	2	3	3	2	4	41	8	6	4	3	3	5	2017Liu#007	B. melitensis	63	111		WS006	Human	2012	Shangdu, China
1	5	3	13	2	3	3	2	4	41	8	6	4	3	3	5	2017Liu#053	B. melitensis	63	111		WS052	Human	2015	Fengzhen, China
1	5	3	13	2	3	3	2	4	41	8	6	4	3	3	5	2017Liu#121	B. melitensis bv3	63	111		QY1		2016	
1	5	3	13	2	3	3	2	4	41	8	5	4	3	3	5	2013Jiang#041	B. melitensis bv1	63	111		NM96-1004	Ovine	1996	Inner Mongolia, China
1	5	3	13	2	3	3	2	4	41	8	5	4	3	3	5	2017Liu#024	B. melitensis	63	111		WS023	Human	2015	Fengzhen, China
1	5	3	13	2	3	3	2	4	41	8	4	4	3	3	5	2017Liu#009	B. melitensis	63	111		WS008	Human	2014	Fengzhen, China
1	5	3	13	2	3	3	2	4	41	8	7	4	3	3	5	2017Liu#044	B. melitensis	63	111		WS043	Ovine	2015	Siziwangqi, China
1	5	3	13	2	3	3	2	4	41	8	6	4	8	3	5	2013Jiang#012	B. melitensis bv3	63	111		NM06-8	Ovine	2006	Inner Mongolia, China
1	5	3	13	2	3	3	2	4	41	8	5	4	3	3	6	2011Jiang#099	B. melitensis bv3	63	111		NM97-09	Human	1997	Inner Mongolia, China
1	5	3	13	2	3	3	2	4	41	8	4	4	3	6	5	2011Aftab#009	B. melitensis	63	111		AIII3cd2	Human		Turkey
1	5	3	13	2	3	3	2	4	41	8	4	4	3	6	5	2013Jiang#060	B. melitensis bv3	63	111		XJ113	Ovine	2011	Xinjiang, China
1	5	3	13	2	3	3	2	4	41	8	6	4	3	6	5	2013Jiang#068	B. melitensis bv1	63	111		XJ157	Ovine	2011	Xinjiang, China
1	5	3	13	2	3	3	2	4	41	8	5	4	3	8	5	2015Shevtsov#138	B. melitensis bv3	63	111		002124	Dog	2009	Almaty, Almaty, Almaty,.
1	5	3	13	2	3	3	2	4	41	8	5	4	3	8	5	2015Shevtsov#139	B. melitensis	63	111		57is	Ovine	2013	Zhambyl,Moiynkum,Zh.
1	5	3	13	2	3	3	2	4	41	8	5	4	3	8	5	2015Shevtsov#140	B. melitensis	63	111		58is	Ovine	2013	Zhambyl,Moiynkum,Zh.
1	5	3	13	2	3	3	2	4	41	8	5	4	3	8	5	2015Shevtsov#141	B. melitensis	63	111		59is	Ovine	2013	Zhambyl,Moiynkum,Zh.
1	5	3	13	2	3	3	2	4	41	8	5	4	3	8	5	2015Shevtsov#142	B. melitensis	63	111		62is	Ovine	2013	Zhambyl,Moiynkum,Zh.
1	5	3	13	2	3	3	2	4	41	8	5	4	3	8	5	2015Shevtsov#143	B. melitensis	63	111		64is	Ovine	2013	Kenes,Moiynkum,Zha.
1	5	3	13	2	3	3	2	4	41	8	5	4	3	8	5	2015Shevtsov#160	B. melitensis bv3	63	111		011207	Dog	2009	Almaty, Almaty, Almaty,.
1	5	3	13	2	3	3	2	4	41	8	5	4	3	5	5	2017Liu#120	B. melitensis bv3	63	111		NM-15		2016	
1	5	3	13	2	3	3	2	4	41	8	5	4	3	7	5	2011Jiang#033	B. melitensis bv3	63	111		NM60-25	Human	1960	Inner Mongolia, China
1	5	3	13	2	3	3	2	4	41	8	5	4	3	7	5	2015Shevtsov#210	B. melitensis bv1	63	111		154ZH	Ovine	2008	Zhanaturminskiy,Bayza.
1	5	3	13	2	3	3	2	4	41	8	5	4	3	6	5	2011Jiang#030	B. melitensis bv3	63	111		NM57-264	Human	1957	Inner Mongolia, China
1	5	3	13	2	3	3	2	4	41	8	5	4	3	6	5	2015Shevtsov#152	B. melitensis	63	111		103is	Ovine	2013	Kylshybay,Moiynkum,Z.
1	5	3	13	2	3	3	2	4	41	8	5	4	3	6	5	2015Shevtsov#153	B. melitensis	63	111		104is	Ovine	2013	Kylshybay,Moiynkum,Z.
1	5	3	13	2	3	3	2	4	41	8	5	4	3	6	5	2015Shevtsov#154	B. melitensis	63	111		105is	Ovine	2013	Kylshybay,Moiynkum,Z.
1	5	3	13	2	3	3	2	4	41	8	5	4	3	6	5	2015Shevtsov#155	B. melitensis	63	111		106is	Ovine	2013	ZhanaZhol,Shu,Zhamby.
1	5	3	13	2	3	3	2	4	41	8	5	4	3	6	5	2015Shevtsov#156	B. melitensis	63	111		107is	Ovine	2013	ZhanaZhol,Shu,Zhamby.
1	5	3	13	2	3	3	2	4	41	8	5	4	3	6	5	2015Shevtsov#157	B. melitensis	63	111		108is	Ovine	2013	ZhanaZhol,Shu,Zhamby.
1	5	3	13	2	3	3	2	4	41	8	5	4	3	6	5	2015Shevtsov#158	B. melitensis	63	111		109is	Ovine	2013	ZhanaZhol,Shu,Zhamby.
1	5	3	13	2	3	3	2	4	49	8	5	4	3	6	5	2011Jiang#065	B. melitensis bv1	63	293		SH07-33	Human	2007	Shanghai, China
1	5	3	13	2	2	3	2	4	41	8	7	4	3	7	5	2015Shevtsov#169	B. melitensis	42	116		29Zh	Ovine	2010	Zhualy,Zhambyl,Kazakh.
1	5	3	13	2	2	3	2	4	41	8	7	4	3	7	5	2015Shevtsov#219	B. melitensis	42	116		39is	Ovine	2013	Nogaybay,Korday,Zha.
1	5	3	13	2	2	3	2	4	41	8	7	4	3	7	5	2015Shevtsov#222	B. melitensis	42	116		41is	Ovine	2013	Nogaybay,Korday,Zha.
1	5	3	13	2	3	3	2	4	41	8	7	4	3	7	5	2015Shevtsov#134	B. melitensis	63	111		46is	Ovine	2013	Akkum,Talas,Zhambyl,.
1	5	3	13	2	3	3	2	4	41	8	7	4	3	7	5	2015Shevtsov#135	B. melitensis	63	111		50is	Ovine	2013	Akkum,Talas,Zhambyl,.
1	5	3	13	2	3	3	2	4	41	8	7	4	3	7	5	2015Shevtsov#136	B. melitensis	63	111		51is	Ovine	2013	Akkum,Talas,Zhambyl,.
1	5	3	13	2	3	3	2	4	41	8	7	4	3	7	5	2015Shevtsov#137	B. melitensis	63	111		56is	Ovine	2013	Tamdy,Talas,Zhambyl,.
1	5	3	13	2	3	3	2	4	41	9	7	4	3	3	5	2017Liu#004	B. melitensis	63	343		WS003	Human	2012	Ulanqab, China
1	5	3	13	2	3	3	2	4	41	8	7	4	3	3	4	2017Liu#037	B. melitensis	63	111		WS036	Human	2015	Qianqi, China
1	5	3	13	2	2	3	2	4	41	8	3	4	3	4	4	2011Jiang#084	B. melitensis bv3	42	116		LB10-13	Human	2010	Guangdong, China
1	5	3	13	2	2	3	2	4	41	8	3	4	3	4	5	2017Hanot-Mamb.	B. melitensis bv1	42	116		L3/298	Human	2015	Belgium
1	5	3	13	2	1	3	2	4	41	8	3	4	3	4	5	2017Liu#043	B. melitensis	114	291		WS042	Human	2015	Ulanqab, China
1	5	3	13	2	1	3	2	4	41	8	3	4	3	4	5	2017Liu#058	B. melitensis	114	291		WS057	Human	2015	Houqi, China
1	5	3	13	2	1	3	2	4	41	8	3	4	3	4	5	2017Liu#108	B. melitensis	114	291		WS107	Human	2015	Qianqi, China
1	5	3	13	2	1	3	2	4	41	8	3	4	3	7	5	2013Jiang#014	B. melitensis bv3	114	291		SHD01-2	Ovine	2001	Shandong, China
1	5	3	13	2	1	3	2	4	41	8	3	4	3	7	5	2013Jiang#015	B. melitensis bv3	114	291		SHD01-3	Ovine	2001	Shandong, China
1	5	3	13	2	1	3	2	4	41	8	5	4	3	7	4	2011Jiang#015	B. melitensis bv3	114	291		GD08-7	Human	2008	Guangdong, China
1	5	3	13	2	1	3	2	4	41	8	5	4	3	7	5	2011Jiang#018	B. melitensis bv3	114	291		GD08-10	Human	2008	Guangdong, China
1	5	3	13	2	1	3	2	4	41	8	9	4	3	6	6	2017Liu#012	B. melitensis	114	291		WS011	Human	2014	Shangdu, China
1	5	3	13	2	1	3	2	4	41	8	5	4	3	6	6	2017Liu#087	B. melitensis	114	291		WS086	Human	2015	Qianqi, China
1	5	3	14	2	3	3	2	4	41	8	5	4	3	6	6	SRR4436571	B. melitensis	194	375	8	BwIM_SYR_47	Human	2017	Syria
1	5	3	13	2	2	3	2	4	41	8	4	4	3	6	8	2013Jiang#062	B. melitensis bv3	42	116		XJ150	Ovine	2011	Xinjiang, China
1	5	3	13	2	2	3	2	4	41	8	4	4	3	6	8	2013Jiang#063	B. melitensis bv3	42	116		XJ151	Caprine	2011	Xinjiang, China
1	5	3	13	2	2	3	2	4	41	8	4	4	3	6	8	2013Jiang#071	B. melitensis bv3	42	116		XJ160	Ovine	2011	Xinjiang, China
1	5	3	13	2	2	3	2	4	41	8	4	4	3	6	7	2013Jiang#004	B. melitensis bv3	42	116		QH86-39	Bharal	1986	Qinghai, China

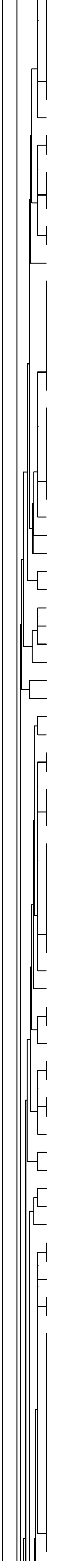

1	5	3	13	2	2	3	2	4	41	8	4	4	3	6	7	2013Jiang#072	B. melitensis bv3	42	116	XJ161	Ovine	2011	Xinjiang, China
1	5	3	13	2	2	3	2	4	41	8	4	4	3	6	7	2015Shevtsov#171	B. melitensis bv1	42	116	314Zh	Ovine	2011	Zhualy,Zhambyl,Kazakh.
1	5	3	13	2	2	3	2	4	41	8	4	4	3	6	7	2015Shevtsov#175	B. melitensis bv1	42	116	319Zh	Ovine	2011	Zhualy,Zhambyl,Kazakh.
1	5	3	13	2	2	3	2	4	41	8	4	4	3	6	7	2015Shevtsov#176	B. melitensis bv1	42	116	320Zh	Ovine	2011	Merki,Zhambyl,Kazakhs.
1	5	3	13	2	2	3	2	4	41	8	4	4	3	6	7	2015Shevtsov#221	B. melitensis bv3	42	116	208	Ovine	2008	Enbekshikazakh,Almaty.
1	5	3	13	2	2	3	2	4	41	8	4	4	3	6	7	2017Liu#045	B. melitensis	42	116	WS044	Human	2015	Suniteyouqi, China
1	5	3	13	2	2	3	2	4	41	8	4	4	3	6	4	2017Liu#011	B. melitensis	42	116	WS010	Human	2014	Huade, China
1	5	3	13	2	2	3	2	4	41	8	4	8	3	6	5	2018Vergnaud#1121	B. melitensis bv3	42	116	BCCN#75-172	Human	1975	Castille, Spain
1	5	3	13	2	2	3	2	4	41	8	4	8	3	6	5	2018Vergnaud#1150	B. melitensis bv3	42	116	BCCN#75-147	Human	1975	Castille, Spain
1	5	3	13	2	2	3	2	4	41	8	4	7	3	6	5	2018Vergnaud#0817	B. melitensis bv3	42	116	BCCN#75-281	Human	1975	Castille, Spain
1	5	3	13	2	2	3	2	4	41	8	4	7	3	6	5	2018Vergnaud#0946	B. melitensis bv3	42	116	BCCN#88-69	Human	1988	Bry-Sur-Marne ,Val-de.
1	5	3	13	2	2	3	2	4	41	8	4	7	3	6	5	2018Vergnaud#1258	B. melitensis bv3	42	116	BCCN#75-297	Human	1975	Castille, Spain
1	5	3	13	2	2	3	2	4	41	8	4	4	3	6	5	2007AlDahouk#103	B. melitensis bv3	42	116	BfR 82	Human	1999	Kazakhstan
1	5	3	13	2	2	3	2	4	41	8	4	4	3	6	5	2013Jiang#003	B. melitensis bv3	42	116	QH87-41	Bharal	1987	Qinghai, China
1	5	3	13	2	2	3	2	4	41	8	4	4	2	6	5	2017Liu#132	B. melitensis bv3	42	116	NM-3	Ovine	2016	
1	5	3	13	2	2	3	2	4	41	8	7	4	3	6	7	2011Jiang#066	B. melitensis bv2	42	116	NM55-013	Human	1955	Inner Mongolia, China
1	5	3	13	2	2	3	2	4	41	8	7	4	3	6	7	2011Jiang#068	B. melitensis bv2	42	116	NM65-039	Human	1965	Inner Mongolia, China
1	5	3	13	2	2	3	2	4	41	8	7	4	3	6	7	2011Jiang#071	B. melitensis bv2	42	116	SD73-009	Human	1973	Shandong, China
1	5	3	13	2	2	3	2	4	41	8	7	4	3	6	7	2013Jiang#022	B. melitensis bv3	42	116	SHD91-1	Ovine	1991	Shandong, China
1	5	3	13	2	2	3	2	4	41	8	7	4	3	6	7	2013Jiang#030	B. melitensis bv2	42	116	NM63-006	Ovine	1963	Inner Mongolia, China
1	5	3	13	2	2	3	2	4	41	8	7	4	3	6	7	2013Jiang#031	B. melitensis bv2	42	116	SH64-027	Cattle		Shanghai, China
1	5	3	13	2	2	3	2	4	41	8	7	4	3	6	7	2013Jiang#034	B. melitensis bv2	42	116	NM73-026	Ovine	1973	Inner Mongolia, China
1	5	3	13	2	2	3	2	4	41	8	7	4	3	6	5	2011Jiang#052	B. melitensis bv3	42	116	ZHJ05-43	Human	2005	Zhejiang, China
1	5	3	13	2	2	3	2	4	41	8	7	4	3	6	5	2011Kilic#048	B. melitensis bv3	42	116	BRU-S048	Human	2008	Erzurum, Turkey
1	5	3	13	2	2	3	2	4	41	8	7	4	3	6	5	2015Shevtsov#145	B. melitensis	42	116	101is	Ovine	2013	Karaoy,Ile,Almaty,Kaza.
1	5	3	13	2	2	3	2	4	41	8	7	4	3	6	5	2015Shevtsov#252	B. melitensis	42	116	88is	Ovine	2013	Bakanas, Balkhash,Alm.
1	5	3	13	2	2	3	2	4	41	8	7	4	3	6	5	2017Liu#016	B. melitensis	42	116	WS015	Human	2014	Qianqi, China
1	5	3	13	2	2	3	2	4	41	8	7	4	3	6	5	2017Liu#020	B. melitensis	42	116	WS019	Human	2014	Huade, China
1	5	3	13	2	2	3	2	4	41	8	7	4	3	6	11	2017Liu#081	B. melitensis	42	116	WS080	Human	2015	Liangcheng, China
1	5	3	13	2	2	3	2	4	41	8	7	8	3	6	7	2012Ferreira#026	B. melitensis bv3	42	116	AFSSA-03/2230	unknown	2003	Portugal
1	5	3	13	2	2	3	2	4	41	8	7	1	3	6	5	2017Liu#018	B. melitensis	42	116	WS017	Human	2014	Shangdu, China
1	5	3	13	2	2	3	2	4	41	8	6	4	3	6	3	2013Jiang#019	B. melitensis bv3	42	116	SHX79-3	Ovine	1979	Shanxi, China
1	5	3	13	2	2	3	2	4	41	8	9	4	3	6	3	2018Vergnaud#0572	B. melitensis bv3	42	116	BCCN#87-51	Ovine	1987	Murcie ,Spain
1	5	3	13	2	2	3	2	4	41	8	5	6	3	6	7	2007AlDahouk#064	B. melitensis bv2	42	116	BfR 30	Human	1999	Turkey
1	5	3	13	2	2	3	2	4	41	8	6	6	3	6	7	2018Vergnaud#0631	B. melitensis bv3	42	116	BCCN#94-69	Human	1994	Turkey
1	5	3	13	2	2	3	2	4	41	8	4	6	3	6	7	2011Kilic#034	B. melitensis bv3	42	116	BRU-S034	Human	2007	Igdir, Turkey
1	5	3	13	2	2	3	2	4	41	8	5	6	3	4	7	2012Ferreira#118	B. melitensis bv3	42	116	LNIV-766Bm3-06	unknown	2006	Entre Douro e Minho, P.
1	5	3	12	2	2	3	2	4	41	8	5	4	3	6	7	2011Jiang#047	B. melitensis bv3	45	115	SHANXI07-1	Human	2007	Shanxi, China
1	5	3	12	2	2	3	2	4	41	8	4	5	3	6	7	2017Hanot-Mamb.	B. melitensis bv3	45	115	L3/139	Human	2004	Belgium
1	5	3	13	2	2	3	2	4	41	8	8	4	3	7	7	2013Jiang#024	B. melitensis bv3	42	116	HN03-1	Ovine	2003	Henan, China
1	5	3	13	2	2	3	2	4	41	8	7	4	3	7	7	2017Liu#074	B. melitensis	42	116	WS073	Human	2015	Xinghe, China
1	5	3	13	2	2	3	2	4	41	8	5	4	3	8	7	2011Jiang#022	B. melitensis bv3	42	116	GD07-4	Human	2007	Guangdong, China
1	5	3	13	2	2	3	2	4	41	8	5	4	3	8	7	2017Liu#080	B. melitensis	42	116	WS079	Human	2015	Zhongqi, China
1	5	3	13	2	2	3	2	4	41	8	5	4	3	6	7	2011Jiang#016	B. melitensis bv3	42	116	GD08-8	Human	2008	Guangdong, China
1	5	3	13	2	2	3	2	4	41	8	5	4	3	6	7	2017Liu#040	B. melitensis	42	116	WS039	Human	2015	Qianqi, China
1	5	3	13	2	2	3	2	4	41	8	5	4	3	6	7	2017Liu#088	B. melitensis	42	116	WS087	Human	2015	Zhongqi, China
1	5	3	13	2	2	3	2	4	41	8	5	4	3	7	7	2011Jiang#011	B. melitensis bv3	42	116	GD08-3	Human	2008	Guangdong, China
1	5	3	13	2	2	3	2	4	41	8	5	4	3	7	7	2011Jiang#088	B. melitensis bv1	42	116	NM94-0705	Human	1994	Inner Mongolia, China
1	5	3	13	2	2	3	2	4	41	8	5	4	3	7	7	2011Jiang#091	B. melitensis bv1	42	116	NM95-1462	Human	1995	Inner Mongolia, China
1	5	3	13	2	2	3	2	4	41	8	5	4	3	7	7	2013Jiang#037	B. melitensis bv1	42	116	NM95-1212	Ovine	1995	Inner Mongolia, China
1	5	3	13	2	2	3	2	4	41	8	5	4	3	7	7	2013Jiang#039	B. melitensis bv1	42	116	NM95-1250	Ovine	1995	Inner Mongolia, China
1	5	3	13	2	2	3	2	4	41	8	5	4	3	7	7	2013Jiang#040	B. melitensis bv1	42	116	NM95-1453	Ovine	1995	Inner Mongolia, China
1	5	3	13	2	2	3	2	4	41	8	5	4	3	7	7	2017Liu#086	B. melitensis	42	116	WS085	Human	2015	Shangdu, China
1	5	3	13	2	2	3	2	4	41	8	5	4	3	3	7	2011Jiang#104	B. melitensis bv1	42	116	NM93-0824	Human	1993	Inner Mongolia, China
1	5	3	13	2	2	3	2	4	41	8	5	5	3	7	7	2011Jiang#003	B. melitensis bv3	42	116	SHX08-14	Human	2008	Shan\'xi, China
1	5	3	13	2	2	3	2	4	41	8	5	4	3	7	3	2011Jiang#092	B. melitensis bv1	42	116	NM95-1463	Human	1995	Inner Mongolia, China
1	5	3	13	2	2	3	2	4	41	8	5	4	3	7	3	2011Jiang#094	B. melitensis bv1	42	116	NM95-1465	Human	1995	Inner Mongolia, China
1	5	3	13	2	2	3	2	4	41	8	5	4	3	8	3	2011Jiang#093	B. melitensis bv1	42	116	NM95-1464	Human	1995	Inner Mongolia, China
1	5	3	13	2	2	3	2	4	41	8	6	4	3	8	7	2018Vergnaud#0279	B. melitensis bv1	42	116	BCCN#02-41	Human	2002	Mongolia
1	5	3	13	2	2	3	2	4	41	8	6	4	3	8	7	2018Vergnaud#0428	B. melitensis bv1	42	116	BCCN#02-13	Human	2002	Mongolia
1	5	3	13	2	2	3	2	4	41	8	6	4	3	7	7	2017Liu#030	B. melitensis	42	116	WS029	Human	2015	Fengzhen, China
1	5	3	13	2	2	3	2	4	41	8	6	4	3	7	7	2018Vergnaud#0525	B. melitensis bv1	42	116	BCCN#02-17	Human	2002	Mongolia
1	5	3	13	2	2	3	2	4	41	8	6	4	3	10	7	2013Jiang#038	B. melitensis bv1	42	116	NM95-1232	Ovine	1995	Inner Mongolia, China
1	5	3	13	2	2	3	2	4	41	8	5	4	1	7	7	2011Jiang#090	B. melitensis bv1	42	116	NM95-1461	Human	1995	Inner Mongolia, China
1	5	3	13	2	2	3	2	4	41	8	5	4	1	7	6	2011Jiang#096	B. melitensis bv1	42	116	NM96-0618	Human	1996	Inner Mongolia, China
1	5	3	13	2	2	3	2	4	41	8	6	4	3	2	6	2013Jiang#048	B. melitensis bv1	42	116	NM91-0510	Ovine	1991	Inner Mongolia, China
1	5	3	13	2	2	3	2	4	41	8	6	4	3	3	6	SRR4436578	B. melitensis	42	116	BwIM_IRN_28	Human	2017	Iran
1	5	3	13	2	2	3	2	4	41	8	8	4	3	3	6	2013Jiang#074	B. melitensis bv3	42	116	XJ164	Cattle	2011	Xinjiang, China
1	5	3	13	2	2	3	2	4	41	8	9	4	3	7	6	2017Liu#027	B. melitensis	42	116	WS026	Human	2015	Fengzhen, China
1	5	3	13	2	2	3	2	4	41	8	9	4	3	7	6	2017Liu#028	B. melitensis	42	116	WS027	Human	2015	Fengzhen, China
1	5	3	13	2	2	3	2	4	41	8	11	4	3	7	6	2015Shevtsov#220	B. melitensis	42	116	40is	Ovine	2013	Nogaybay,Korday,Zha.
1	5	3	13	2	2	3	2	4	41	8	6	4	3	7	6	2013Jiang#036	B. melitensis bv1	42	116	NM93-0907	Ovine	1993	Inner Mongolia, China
1	5	3	13	2	2	3	2	4	41	8	6	4	3	7	6	2017Liu#047	B. melitensis	42	116	WS046	Human	2015	Liangcheng, China
1	5	3	13	2	2	3	2	4	41	8	5	4	3	7	6	2011Jiang#029	B. melitensis bv3	42	116	NM57-55	Human	1957	Inner Mongolia, China
1	5	3	13	2	2	3	2	4	41	8	5	4	3	7	6	2011Jiang#031	B. melitensis bv3	42	116	NM58-2	Human	1958	Inner Mongolia, China
1	5	3	13	2	2	3	2	4	41	8	5	4	3	7	6	2011Jiang#038	B. melitensis bv3	42	116	GD09-2	Human	2009	Guangdong, China
1	5	3	13	2	2	3	2	4	41	8	5	4	3	7	6	2011Jiang#046	B. melitensis bv3	42	116	SHANXI09-17	Human	2009	Shanxi, China
1	5	3	13	2	2	3	2	4	41	8	5	4	3	7	6	2011Jiang#083	B. melitensis bv3	42	116	LB10-12	Human	2010	Guangdong, China
1	5	3	13	2	2	3	2	4	41	8	5	4	3	7	6	2011Jiang#098	B. melitensis bv3	42	116	NM96-0620	Human	1996	Inner Mongolia, China
1	5	3	13	2	2	3	2	4	41	8	5	4	3	7	6	2013Jiang#001	B. melitensis bv3	42	116	HB07-1	Ovine	2007	Hebei, China
1	5	3	13	2	2	3	2	4	41	8	5	4	3	7	6	2013Jiang#002	B. melitensis bv3	42	116	HB07-2	Ovine	2007	Hebei, China
1	5	3	13	2	2	3	2	4	41	8	5	4	3	7	6	2013Jiang#010	B. melitensis bv3	42	116	NM06-6	Ovine	2006	Inner Mongolia, China
1	5	3	13	2	2	3	2	4	41	8	5	4	3	7	6	2013Jiang#047	B. melitensis bv1	42	116	NM91-0509	Ovine	1991	Inner Mongolia, China
1	5	3	13	2	2	3	2	4	41	8	5	4	3	7	6	2013Jiang#054	B. melitensis bv1	42	116	NM92-0614	Ovine	1992	Inner Mongolia, China
1	5	3	13	2	2	3	2	4	41	8	5	4	3	7	6	2013Jiang#056	B. melitensis bv1	42	116	NM92-0616	Ovine	1992	Inner Mongolia, China
1	5	3	13	2	2	3	2	4	41	8	5	4	3	7	6	2017Liu#098	B. melitensis	42	116	WS097	Human	2015	Shangdu, China

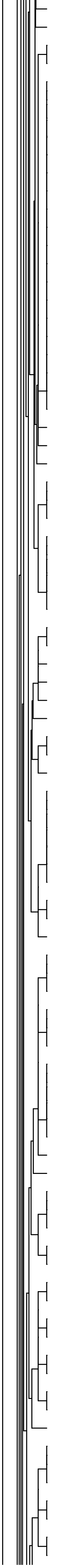

1	5	3	13	2	2	3	2	4	41	8	5	8	3	7	6	2018Vergnaud#1299	B. melitensis bv3	42	116		BCCN#75-274	Human	1975	Castille, Spain
1	5	3	12	2	2	3	2	4	41	8	5	4	3	7	6	2011Jiang#049	B. melitensis bv3	45	115		SHANXI07-3	Human	2007	Shanxi, China
1	5	3	13	2	2	3	2	4	41	8	5	4	3	9	6	2011Jiang#087	B. melitensis bv3	42	116		LB10-17	Human	2010	Guangdong, China
1	5	3	13	2	2	3	2	4	41	8	5	4	3	9	6	2013Jiang#082	B. melitensis bv3	42	116		LY009	Human	2011	Zhejiang, China
1	5	3	13	2	2	3	2	4	41	8	5	4	3	5	6	2011Jiang#060	B. melitensis bv3	42	116		LN05-2	Human	2005	Liaoning, China
1	5	3	13	2	2	3	2	4	41	8	5	4	3	5	6	2015Shevtsov#131	B. melitensis	42	116		93is	Ovine	2013	Koktal,Balkhash,Almaty.
1	5	3	13	2	2	3	2	4	41	8	5	4	3	5	6	2015Shevtsov#132	B. melitensis	42	116		94is	Ovine	2013	Koktal,Balkhash,Almaty.
1	5	3	13	2	2	3	2	4	41	8	5	4	3	5	6	2015Shevtsov#133	B. melitensis	42	116		95is	Ovine	2013	Ushkonyr,Karasay,Alma.
1	5	3	13	2	2	3	2	4	41	8	5	4	3	5	6	2015Shevtsov#144	B. melitensis	42	116		100is	Ovine	2013	Karaoy,Ile,Almaty,Kaza.
1	5	3	13	2	2	3	2	4	41	8	5	4	3	5	6	2015Shevtsov#146	B. melitensis	42	116		102is	Ovine	2013	Karaoy,Ile,Almaty,Kaza.
1	5	3	13	2	2	3	2	4	41	8	5	4	3	5	6	2015Shevtsov#147	B. melitensis	42	116		95is	Ovine	2013	Ushkonyr,Karasay,Alma.
1	5	3	13	2	2	3	2	4	41	8	5	4	3	5	6	2015Shevtsov#148	B. melitensis	42	116		96is	Ovine	2013	Ushkonyr,Karasay,Alma.
1	5	3	13	2	2	3	2	4	41	8	5	4	3	5	6	2015Shevtsov#149	B. melitensis	42	116		97is	Ovine	2013	Karaoy,Ile,Almaty,Kaza.
1	5	3	13	2	2	3	2	4	41	8	5	4	3	5	6	2015Shevtsov#150	B. melitensis	42	116		98is	Ovine	2013	Karaoy,Ile,Almaty,Kaza.
1	5	3	13	2	2	3	2	4	41	8	5	4	3	5	6	2015Shevtsov#151	B. melitensis	42	116		99	Ovine	2013	Karaoy,Ile,Almaty,Kaza.
1	5	3	13	2	2	3	2	4	41	8	5	4	3	5	6	2015Shevtsov#167	B. melitensis bv1	42	116		33Zh	Ovine	2009	Zhualy,Zhambyl,Kazakh.
1	5	3	13	2	2	3	2	4	41	8	5	4	3	5	6	2015Shevtsov#182	B. melitensis	42	116		1is	Ovine	2013	Kasyk, Korday, Zhamby.
1	5	3	13	2	2	3	2	4	41	8	5	4	3	5	6	2015Shevtsov#183	B. melitensis	42	116		2is	Ovine	2013	Kasyk, Korday,Zhambyl.
1	5	3	13	2	2	3	2	4	41	8	5	4	3	5	6	2015Shevtsov#184	B. melitensis	42	116		3is	Ovine	2013	Kasyk, Korday,Zhambyl.
1	5	3	13	2	2	3	2	4	41	8	5	4	3	5	6	2015Shevtsov#185	B. melitensis	42	116		4is	Ovine	2013	Kasyk, Korday,Zhambyl.
1	5	3	13	2	2	3	2	4	41	8	5	4	3	5	6	2015Shevtsov#190	B. melitensis	42	116		9is	Ovine	2013	Karasay,Korday,Zhamby.
1	5	3	13	2	2	3	2	4	41	8	5	4	3	5	6	2015Shevtsov#191	B. melitensis	42	116		10is	Ovine	2013	Karasay,Korday,Zhamby.
1	5	3	13	2	2	3	2	4	41	8	5	4	3	5	6	2017Liu#116	B. melitensis	42	116		WS115	Human	2015	Qianqi, China
1	5	3	13	2	2	3	2	4	41	8	5	4	3	4	6	2011Jiang#014	B. melitensis bv3	42	116		GD08-6	Human	2008	Guangdong, China
1	5	3	13	2	2	3	2	4	41	8	5	4	3	10	6	2011Jiang#043	B. melitensis bv3	42	116		SHANXI09-14	Human	2009	Shanxi, China
1	5	3	13	2	2	3	2	4	41	8	5	4	3	5	8	2011Jiang#050	B. melitensis bv3	42	116		GD06-14	Human	2006	Guangdong, China
1	5	3	13	2	2	3	2	4	41	8	7	4	3	8	6	2015Shevtsov#178	B. melitensis	42	116		03040031	Ovine	2012	Enbekshikazakh,Almaty.
1	5	3	13	2	2	3	2	4	41	8	7	4	3	8	6	2015Shevtsov#179	B. melitensis	42	116		03040032	Ovine	2012	Enbekshikazakh,Almaty.
1	5	3	13	2	2	3	2	4	41	8	7	4	3	8	6	2015Shevtsov#180	B. melitensis	42	116		03040045	Ovine	2012	Enbekshikazakh,Almaty.
1	5	3	13	2	2	3	2	4	41	8	5	4	3	8	6	2011Jiang#004	B. melitensis bv3	42	116		SHX08-15	Human	2008	Shan\'xi, China
1	5	3	13	2	2	3	2	4	41	8	5	4	3	8	6	2011Jiang#019	B. melitensis bv3	42	116		GD07-1	Human	2007	Guangdong, China
1	5	3	13	2	2	3	2	4	41	8	5	4	3	8	6	2011Jiang#024	B. melitensis bv3	42	116		GD07-6	Human	2007	Guangdong, China
1	5	3	13	2	2	3	2	4	41	8	5	4	3	8	6	2011Jiang#040	B. melitensis bv3	42	116		GD09-4	Human	2009	Guangdong, China
1	5	3	13	2	2	3	2	4	41	8	5	4	3	8	6	2011Jiang#089	B. melitensis bv1	42	116		NM94-0806	Human	1994	Inner Mongolia, China
1	5	3	13	2	2	3	2	4	41	8	5	7	3	6	6	2007AlDahouk#051	B. melitensis bv2	42	116		BfR 4	Human	2000	Bern, Switzerland
1	5	3	13	2	2	3	2	4	41	8	5	7	3	6	6	2018Vergnaud#0994	B. melitensis bv3	42	116		BCCN#75-310	Human	1975	Castille, Spain
1	5	3	13	2	2	3	2	4	41	8	5	8	3	6	6	2018Vergnaud#0359	B. melitensis R.	42	116		BCCN#92-85	Ovine	1992	Murcie ,Spain
1	5	3	13	2	2	3	2	4	41	8	5	5	3	6	6	2011Jiang#020	B. melitensis bv3	42	116		GD07-2	Human	2007	Guangdong, China
1	5	3	13	2	2	3	2	4	41	8	5	10	3	6	6	2012Ferreira#098	B. melitensis bv3	42	116		LNIV-416Bm3-05	unknown	2005	Alentejo, Portugal
1	5	3	13	2	2	3	2	4	41	8	9	7	3	6	6	2012Ferreira#111	B. melitensis bv3	42	116		LNIV-311Bm3-04	unknown	2004	Algarve, Portugal
1	5	3	13	2	2	3	2	4	41	8	7	6	3	6	6	2011Kilic#080	B. melitensis bv3	42	116		BRU-S080	Human	2003	Kirikkale, Turkey
1	5	3	13	2	2	3	2	4	41	8	7	6	3	6	6	2011Kilic#081	B. melitensis bv3	42	116		BRU-S081	Human	2003	Kirikkale, Turkey
1	5	3	13	2	2	3	2	4	41	8	5	6	3	6	6	2017Hanot-Mamb.	B. melitensis bv3	42	116		L3/131	Human	2002	Belgium
1	5	3	13	2	2	3	2	4	41	8	3	4	3	6	6	2017Liu#054	B. melitensis	42	116		WS053	Human	2015	Fengzhen, China
1	5	3	13	2	2	3	2	4	41	8	3	4	3	6	6	2017Liu#056	B. melitensis	42	116		WS055	Human	2015	Liangcheng, China
1	5	3	13	2	2	3	2	4	41	8	3	4	3	6	6	2017Liu#059	B. melitensis	42	116		WS058	Human	2015	Shangyi, China
1	5	3	13	2	2	3	2	4	41	8	3	4	3	6	6	2017Liu#079	B. melitensis	42	116		WS078	Human	2015	Fengzhen, China
1	5	3	13	2	2	3	2	4	41	8	3	4	3	6	6	2017Liu#091	B. melitensis	42	116		WS090	Human	2015	Fengzhen, China
1	5	3	13	2	2	3	2	4	41	8	3	4	3	6	6	2017Liu#113	B. melitensis	42	116		WS112	Human	2015	Fengzhen, China
1	5	3	13	2	2	3	2	4	41	8	5	4	3	6	6	2011Jiang#025	B. melitensis bv3	42	116		GD07-7	Human	2007	Guangdong, China
1	5	3	13	2	2	3	2	4	41	8	5	4	3	6	6	2017Liu#057	B. melitensis	42	116		WS056	Human	2015	Liangcheng, China
1	5	3	13	2	2	3	2	4	41	8	8	4	3	6	6	SRR4436639	B. melitensis	42	116	8	BwIM_AFG_44	Human	2017	Afghanistan
1	5	3	13	2	2	3	2	4	41	8	4	4	3	3	6	2018Vergnaud#0317	B. melitensis aty.	42	116		BCCN#02-16	Human	2002	Mongolia
1	5	3	13	2	2	3	2	4	41	8	4	4	3	3	6	2018Vergnaud#0366	B. melitensis R.	42	116		BCCN#00-2	Human	2000	Mongolia
1	5	3	13	2	2	3	2	4	41	8	4	4	3	3	6	2018Vergnaud#0367	B. melitensis R.	42	116		BCCN#02-15	Human	2002	Mongolia
1	5	3	13	2	2	3	2	4	41	8	4	4	3	7	6	2015Shevtsov#246	B. melitensis	42	116		81is	Ovine	2013	Kaynar,Raiymbek,Almat.
1	5	3	13	2	2	3	2	4	41	8	4	4	3	7	6	2015Shevtsov#251	B. melitensis	42	116		87is	Ovine	2013	Kakpakty, Raiymbek,A.
1	5	3	13	2	2	3	2	4	41	8	4	4	3	7	6	SRR4436629	B. melitensis	42	116	8	BwIM_SAU_09	Human	2017	Saudi Arabia
1	5	3	13	2	2	3	2	4	41	8	4	4	3	4	6	2011Jiang#056	B. melitensis bv3	42	116		FJ09-4	Human	2009	Fujian, China
1	5	3	13	2	2	3	2	4	41	8	4	4	3	4	6	2015Shevtsov#128	B. melitensis	42	116		90is	Ovine	2013	Bereke,Balkhash,Almat.
1	5	3	13	2	2	3	2	4	41	8	4	4	3	4	6	2015Shevtsov#129	B. melitensis	42	116		91is	Ovine	2013	Bereke,Balkhash,Almat.
1	5	3	13	2	2	3	2	4	41	8	4	4	3	4	6	2015Shevtsov#130	B. melitensis	42	116		92is	Ovine	2013	Bereke,Balkhash,Almat.
1	5	3	13	2	2	3	2	4	41	8	4	4	3	4	6	2015Shevtsov#232	B. melitensis bv2	42	116		280	Ovine	2008	Enbekshikazakh,Almaty.
1	5	3	13	2	2	3	2	4	41	8	4	4	3	6	6	2011Jiang#053	B. melitensis bv3	42	116		HZ09-1	Human	2009	Zhejiang, China
1	5	3	13	2	3	3	2	4	41	8	4	4	3	4	6	2007AlDahouk#004	B. melitensis bv1	63	111		BCCN#92-70	Human	1992	Paris, France
1	5	3	13	2	2	3	2	4	41	8	4	4	3	7	5	2015Shevtsov#199	B. melitensis bv1	42	116		141ZH	Ovine	2008	Karasazskiy,Zhualy,Zh.
1	5	3	13	2	2	3	2	4	41	8	4	4	3	7	5	2015Shevtsov#248	B. melitensis	42	116		83is	Ovine	2013	Kaynar,Raiymbek,Almat.
1	5	3	13	2	2	3	2	4	41	8	4	4	3	7	5	2015Shevtsov#250	B. melitensis	42	116		86is	Ovine	2013	Kaynar,Raiymbek,Almat.
1	5	3	13	2	2	3	2	4	41	8	4	4	3	7	7	2015Shevtsov#170	B. melitensis bv1	42	116		311Zh	Ovine	2011	Bayzak,Zhambyl,Kazak.
1	5	3	13	2	2	3	2	4	41	8	4	4	3	7	7	2015Shevtsov#173	B. melitensis bv1	42	116		317Zh	Ovine	2011	Zhualy,Zhambyl,Kazakh.
1	5	3	13	2	2	3	2	4	41	8	4	9	3	5	6	2018Vergnaud#1140	B. melitensis bv3	42	116		BCCN#91-221	Ovine	1991	Leon, Spain
1	5	3	13	2	2	3	2	4	41	8	4	9	3	5	6	2018Vergnaud#1141	B. melitensis bv3	42	116		BCCN#91-222	Ovine	1991	Leon, Spain
1	5	3	13	2	2	3	2	4	41	8	4	7	3	5	6	2018Vergnaud#0882	B. melitensis bv1	42	116		BCCN#95-28	Human	1995	Navarre, Spain
1	5	3	13	2	2	3	2	4	41	8	4	7	3	5	6	2018Vergnaud#1297	B. melitensis bv3	42	116		BCCN#75-177	Human	1975	Castille, Spain
1	5	3	13	2	2	3	2	4	41	8	4	6	3	5	6	2018Vergnaud#0697	B. melitensis bv3	42	116		BCCN#99-91	Human	1999	Baliros ,Pyrénées-Atlant.
1	5	3	13	2	2	3	2	4	41	8	4	6	3	5	6	2018Vergnaud#1067	B. melitensis bv3	42	116		BCCN#00-27	Human	2000	Haute-Garonne, France
1	5	3	13	2	2	3	2	4	41	8	4	4	3	5	6	2011Jiang#067	B. melitensis bv2	42	116		SX62-020	Human	1962	Shan\'xi, China
1	5	3	13	2	2	3	2	4	41	8	4	4	3	5	6	2013Jiang#020	B. melitensis bv3	42	116		QH80-347	Ovine	1980	Qinghai, China
1	5	3	13	2	2	3	2	4	41	8	4	6	3	8	6	2018Vergnaud#0627	B. melitensis bv3	42	116		BCCN#93-99	Human	1993	Ille-et-Vilaine, France
1	5	3	13	2	2	3	2	4	41	8	4	4	3	8	4	2017Liu#095	B. melitensis	42	116		WS094	Human	2015	Xinghe, China
1	5	3	13	2	2	3	2	4	41	8	4	4	3	8	4	2017Liu#100	B. melitensis	42	116		WS099	Human	2015	Houqi, China
1	5	3	13	2	2	3	2	4	41	8	4	4	3	8	4	2017Liu#107	B. melitensis	42	116		WS106	Human	2015	Zhongqi, China
1	5	3	13	2	2	3	2	4	41	8	4	4	3	8	8	2013Jiang#069	B. melitensis bv3	42	116		XJ158	Ovine	2011	Xinjiang, China
1	5	3	13	2	2	3	2	4	41	8	4	4	3	8	8	GCA_000227645	B. melitensis	42	116	8	NI	Cattle	2011	China
1	5	3	13	2	2	3	2	4	41	8	4	4	3	8	5	2011Jiang#072	B. melitensis bv3	42	116		TJ-01	Human	2010	Tianjin, China
1	5	3	13	2	2	3	2	4	41	8	4	4	3	8	5	2011Kilic#143	B. melitensis bv3	42	116		BRU-S143	Human	2004	Malatya, Turkey

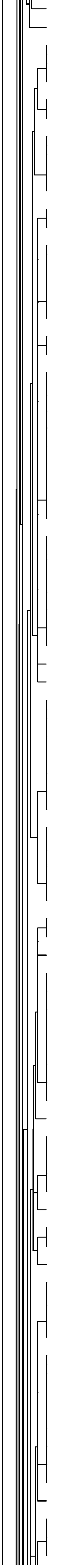

1	5	3	13	2	2	3	2	4	41	8	4	8	3	8	5	2007AlDahouk#032	B. melitensis bv2	42	116		BfR 7	Human	2002	Turkey
1	5	3	13	2	2	3	2	4	41	8	4	7	3	8	7	2018Vergnaud#0672	B. melitensis bv3	42	116		BCCN#99-1	Human	1999	Finistère, France
1	5	3	13	2	3	3	2	4	41	8	5	4	3	8	4	SRR4436589	B. melitensis	63	111		BwIM_AFG_29b	Human		Afghanistan
1	5	3	13	2	3	3	2	4	41	8	5	4	3	8	4	SRR4436592	B. melitensis	63	111		BwIM_AFG_29a	Human		Afghanistan
1	5	3	13	2	3	3	2	4	41	8	5	4	3	8	4	SRR4436592_SR.	B. melitensis	63	111	22			2017	
1	5	3	13	2	2	3	2	4	41	8	5	4	3	8	4	2018Vergnaud#0698	B. melitensis aty.	42	116		BCCN#02-30	unknown	2002	India
1	5	3	13	2	2	3	2	4	41	8	5	4	3	8	4	GCA_001431745	B. melitensis	42	116	8	20236	unknown		
1	5	3	13	2	3	3	2	4	41	8	5	4	3	5	4	2011Jiang#008	B. melitensis bv3	63	111		SHANXI09-3	Human	2009	Shanxi, China
1	5	3	13	2	3	3	2	4	41	8	5	4	3	5	4	2011Jiang#077	B. melitensis bv3	63	111		LB10-06	Human	2010	Guangdong, China
1	5	3	13	2	3	3	2	4	41	8	5	4	3	5	4	2017Liu#046	B. melitensis	63	111		WS045	Human	2015	Houqi, China
1	5	3	13	2	3	3	2	4	41	8	5	4	3	5	4	2017Liu#069	B. melitensis	63	111		WS068	Human	2015	Zhuozi, China
1	5	3	13	2	2	3	2	4	41	8	5	4	3	15	4	2017Liu#025	B. melitensis	42	116		WS024	Human	2015	Liangcheng, China
1	5	3	13	2	2	3	2	4	41	8	5	4	3	15	4	2017Liu#026	B. melitensis	42	116		WS025	Human	2015	Liangcheng, China
1	5	3	13	2	2	3	2	4	41	8	5	4	3	7	4	2015Shevtsov#159	B. melitensis bv3	42	116		011206	Dog	2009	Almaty, Almaty, Almaty,.
1	5	3	13	2	2	3	2	4	41	8	5	4	3	7	4	2017Liu#085	B. melitensis	42	116		WS084	Human	2015	Liangcheng, China
1	5	3	13	2	2	3	2	4	41	8	5	4	3	7	4	2017Liu#103	B. melitensis	42	116		WS102	Human	2015	Fengzhen, China
1	5	3	13	2	2	3	2	4	41	8	5	4	3	7	4	2018Vergnaud#0316	B. melitensis aty.	42	116		BCCN#02-11	Ovine	2002	India
1	5	3	13	2	2	3	2	4	41	8	5	4	3	7	4	2018Vergnaud#0728	B. melitensis bv1	42	116		BCCN#00-20	Ovine	2000	India
1	5	3	13	2	2	3	2	4	41	8	5	4	3	3	4	2013Jiang#066	B. melitensis bv3	42	116		XJ155	Ovine	2011	Xinjiang, China
1	5	3	13	2	2	3	2	4	41	8	5	4	3	3	4	2013Jiang#067	B. melitensis bv3	42	116		XJ156	Ovine	2011	Xinjiang, China
1	5	3	13	2	2	3	2	4	41	8	5	4	3	5	4	2011Jiang#002	B. melitensis bv3	42	116		SHX08-6	Human	2008	Shan\'xi, China
1	5	3	13	2	2	3	2	4	41	8	5	4	3	5	4	2011Jiang#006	B. melitensis bv3	42	116		SHANXI09-4	Human	2009	Shanxi, China
1	5	3	13	2	2	3	2	4	41	8	5	4	3	5	4	2011Jiang#010	B. melitensis bv3	42	116		GD08-2	Human	2008	Guangdong, China
1	5	3	13	2	2	3	2	4	41	8	5	4	3	5	4	2011Jiang#012	B. melitensis bv3	42	116		GD08-4	Human	2008	Guangdong, China
1	5	3	13	2	2	3	2	4	41	8	5	4	3	5	4	2017Liu#021	B. melitensis	42	116		WS020	Human	2014	Qianqi, China
1	5	3	13	2	2	3	2	4	41	8	5	4	3	5	4	2017Liu#033	B. melitensis	42	116		WS032	Human	2015	Liangcheng, China
1	5	3	13	2	2	3	2	4	41	8	5	4	3	5	4	2018Vergnaud#0342	B. melitensis aty.	42	116		BCCN#02-32	Ovine	2002	India
1	5	3	13	2	2	3	2	4	41	8	5	4	3	5	4	2018Vergnaud#0343	B. melitensis aty.	42	116		BCCN#02-33	Ovine	2002	India
1	5	3	13	2	2	3	2	4	41	8	5	4	3	5	4	2018Vergnaud#0344	B. melitensis aty.	42	116		BCCN#02-35	Human	2002	India
1	5	3	13	2	2	3	2	4	41	8	5	4	3	6	4	2006LeFleche#100	B. melitensis	42	116		BCCN#94-49	Ovine	1994	India
1	5	3	13	2	2	3	2	4	41	8	5	4	3	6	4	2011Jiang#009	B. melitensis bv3	42	116		SJZ09-1	Human	2009	Hebei, China
1	5	3	13	2	2	3	2	4	41	8	5	4	3	6	4	2017Liu#031	B. melitensis	42	116		WS030	Human	2015	Liangcheng, China
1	5	3	13	2	2	3	2	4	41	8	5	4	3	6	4	2018Vergnaud#0310	B. melitensis bv1	42	116		BCCN#00-18	Ovine	2000	India
1	5	3	13	2	2	3	2	4	41	8	5	4	3	6	4	2018Vergnaud#0313	B. melitensis aty.	42	116		BCCN#94-48	Ovine	1994	India
1	5	3	13	2	2	3	2	4	41	8	5	4	3	6	4	2018Vergnaud#0314	B. melitensis aty.	42	116		BCCN#02-9	Ovine	2002	India
1	5	3	13	2	2	3	2	4	41	8	5	4	3	6	4	2018Vergnaud#0318	B. melitensis aty.	42	116		BCCN#02-31	Ovine	2002	India
1	5	3	13	2	2	3	2	4	41	8	5	4	3	4	4	2011Jiang#070	B. melitensis bv2	42	116		JL71-020	Human	1971	Jilin, China
1	5	3	13	2	2	3	2	4	41	8	5	4	3	13	4	2017Liu#035	B. melitensis	42	116		WS034	Human	2015	Liangcheng, China
1	5	3	13	2	2	3	2	4	41	8	5	7	3	6	4	2018Vergnaud#0493	B. melitensis bv3	42	116		BCCN#01-20	Human	2001	Portugal
1	5	3	13	2	2	3	2	4	41	8	5	7	3	6	4	2018Vergnaud#0494	B. melitensis bv3	42	116		BCCN#01-21	Human	2001	France
1	5	3	13	2	2	3	2	4	41	8	5	7	3	6	4	2018Vergnaud#0495	B. melitensis bv3	42	116		BCCN#01-22	Human	2001	Portugal
1	5	3	13	2	2	3	2	4	41	8	5	7	3	6	4	2018Vergnaud#0496	B. melitensis bv3	42	116		BCCN#01-23	Human	2001	Portugal
1	5	3	13	2	2	3	2	4	41	8	5	7	3	6	4	2018Vergnaud#0885	B. melitensis bv3	42	116		BCCN#00-9	Human	2000	Paris, France
1	5	3	13	2	2	3	2	4	41	8	5	7	3	6	4	2018Vergnaud#1112	B. melitensis bv3	42	116		BCCN#00-15	Human	2000	Essonne, France
1	5	3	13	2	2	3	2	4	41	8	5	7	3	6	4	2018Vergnaud#1113	B. melitensis bv3	42	116		BCCN#00-16	Human	2000	Essonne, France
1	5	3	13	2	2	3	2	4	41	8	5	7	3	5	4	2012Ferreira#037	B. melitensis bv3	42	116		AFSSA-06/0444	unknown	2005	Portugal
1	5	3	13	2	2	3	2	4	41	8	5	7	3	5	4	2012Ferreira#125	B. melitensis bv3	42	116		LNIV-917Bm3-11	unknown	2011	Ribatejo e Oeste, Portu.
1	5	3	13	2	2	3	2	4	41	8	5	7	3	5	4	2012Ferreira#131	B. melitensis bv3	42	116		LNIV-923Bm3-11	unknown	2011	Ribatejo e Oeste, Portu.
1	5	3	13	2	2	3	2	4	41	8	5	7	3	5	4	2018Vergnaud#0610	B. melitensis bv3	42	116		BCCN#92-122	Human	1992	Portugal
1	5	3	13	2	2	3	2	4	41	8	5	7	3	5	4	2018Vergnaud#1208	B. melitensis bv3	42	116		BCCN#93-53	Human	1993	Val-de-Marne, France
1	5	3	13	2	2	3	2	4	41	8	4	4	3	5	8	2013Jiang#070	B. melitensis bv3	42	116		XJ159	Ovine	2011	Xinjiang, China
1	5	3	13	2	2	3	2	4	41	8	4	4	3	5	8	2013Jiang#073	B. melitensis bv3	42	116		XJ162	Cattle	2011	Xinjiang, China
1	5	3	13	2	2	3	2	4	41	8	4	4	3	5	10	2015Shevtsov#174	B. melitensis bv1	42	116		318Zh	Ovine	2011	Zhualy,Zhambyl,Kazakh.
1	5	3	13	2	2	3	2	4	41	8	4	4	3	5	4	2011Aftab#004	B. melitensis	42	116		REF 3413-04	Human		Turkey
1	5	3	13	2	2	3	2	4	41	8	4	4	3	5	4	2013Jiang#065	B. melitensis bv1	42	116		XJ153	Ovine	2011	Xinjiang, China
1	5	3	13	2	2	3	2	4	41	8	4	4	3	5	4	2016Gyuranecz#001	B. melitensis	42	116		C1	Camel	2006	Saudi Arabia
1	5	3	13	2	2	3	2	4	41	8	4	4	3	5	4	2016Gyuranecz#002	B. melitensis	42	116		C2	Camel	2006	Saudi Arabia
1	5	3	13	2	2	3	2	4	41	8	4	4	3	5	4	2016Gyuranecz#004	B. melitensis	42	116		C4	Camel	2006	Saudi Arabia
1	5	3	13	2	2	3	2	4	41	8	4	4	3	5	4	2017Hanot-Mamb.	B. melitensis bv3	42	116		L3/201	Human	2011	Belgium
1	5	3	13	2	2	3	2	4	41	8	4	4	3	5	4	2017Liu#110	B. melitensis	42	116		WS109	Human	2015	Shangyi, China
1	5	3	13	2	2	3	2	4	41	8	4	4	3	5	4	2017Liu#111	B. melitensis	42	116		WS110	Human	2015	Shangyi, China
1	5	3	13	2	2	3	2	4	41	8	4	4	3	11	4	2017Liu#042	B. melitensis	42	116		WS041	Human	2015	Qianqi, China
1	5	3	13	2	2	3	2	4	41	8	9	4	3	5	4	2011Jiang#051	B. melitensis bv3	42	116		BJ06-10	Human	2006	Beijing, China
1	5	3	13	2	2	3	2	4	41	8	9	4	3	5	4	2011Jiang#062	B. melitensis bv3	42	116		NM06-11	Human	2006	Inner Mongolia, China
1	5	3	13	2	2	3	2	4	41	8	9	4	3	5	4	2017Liu#032	B. melitensis	42	116		WS031	Human	2015	Qianqi, China
1	5	3	13	2	2	3	2	4	41	8	9	4	3	5	4	2017Liu#038	B. melitensis	42	116		WS037	Human	2015	Xinghe, China
1	5	3	13	2	2	3	2	4	41	8	9	4	3	8	4	2017Liu#041	B. melitensis	42	116		WS040	Human	2015	Fengzhen, China
1	5	3	13	2	2	3	2	4	41	8	10	4	3	5	4	2017Liu#029	B. melitensis	42	116		WS028	Human	2015	Liangcheng, China
1	5	3	13	2	2	3	2	4	41	8	10	4	3	5	4	2017Liu#051	B. melitensis	42	116		WS050	Human	2015	Liangcheng, China
1	5	3	13	2	2	3	2	4	41	8	10	4	3	5	6	2017Liu#078	B. melitensis	42	116		WS077	Human	2015	Jining, China
1	5	3	13	2	2	3	2	4	41	8	6	4	3	6	4	2017Liu#013	B. melitensis	42	116		WS012	Human	2014	Shangdu, China
1	5	3	13	2	2	3	2	4	41	8	6	4	3	6	4	2018Vergnaud#0315	B. melitensis aty.	42	116		BCCN#02-10	Ovine	2002	India
1	5	3	13	2	2	3	2	4	41	8	6	4	3	6	4	2018Vergnaud#0727	B. melitensis bv1	42	116		BCCN#00-19	Ovine	2000	India
1	5	3	13	2	2	3	2	4	41	8	6	4	3	6	4	SRR4436623	B. melitensis	42	116		BwIM_TKM_56	Human	2017	Turkmenistan
1	5	3	13	2	2	3	2	4	41	8	6	4	3	4	4	2013Jiang#075	B. melitensis bv3	42	116		LY001	Human	2011	Zhejiang, China
1	5	3	13	2	2	3	2	4	41	8	6	4	3	4	4	2013Jiang#076	B. melitensis bv3	42	116		LY002	Human	2011	Zhejiang, China
1	5	3	13	2	2	3	2	4	41	8	6	4	3	4	4	2013Jiang#077	B. melitensis bv3	42	116		LY003	Human	2011	Zhejiang, China
1	5	3	13	2	2	3	2	4	41	8	6	4	3	4	4	2013Jiang#078	B. melitensis bv3	42	116		LY004	Human	2011	Zhejiang, China
1	5	3	13	2	2	3	2	4	41	8	6	4	3	4	4	2013Jiang#079	B. melitensis bv3	42	116		LY005	Caprine	2011	Zhejiang, China
1	5	3	13	2	2	3	2	4	41	8	6	4	3	4	4	2013Jiang#080	B. melitensis bv3	42	116		LY006	Caprine	2011	Zhejiang, China
1	5	3	13	2	2	3	2	4	41	8	6	4	3	4	4	2016Gyuranecz#003	B. melitensis	42	116		C3	Camel	2006	Saudi Arabia
1	5	3	13	2	2	3	2	4	41	8	6	4	3	4	4	2016Gyuranecz#005	B. melitensis	42	116		C5	Camel	2006	Saudi Arabia
1	5	3	13	2	2	3	2	4	41	8	6	4	3	8	4	SRR4436579	B. melitensis	42	116	8	BwIM_SYR_01	Human	2017	Syria
1	5	3	13	2	2	3	2	4	41	8	6	4	3	5	6	2017Liu#049	B. melitensis	42	116		WS048	Human	2015	Fengzhen, China
1	5	3	13	2	2	3	2	4	41	8	6	4	3	5	6	2017Liu#101	B. melitensis	42	116		WS100	Human	2015	Fengzhen, China
1	5	3	13	2	2	3	2	4	41	8	6	4	3	5	6	2017Liu#115	B. melitensis	42	116		WS114	Human	2015	Houqi, China

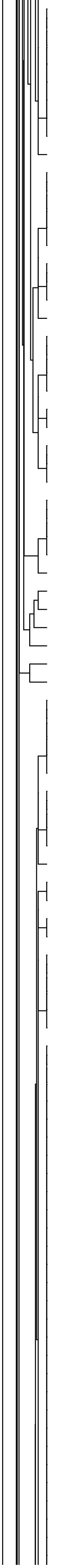

1	5	3	13	2	2	3	2	4	41	8	6	4	3	5	4	2017Liu#023	B. melitensis	42	116		WS022	Human	2015	Liangcheng, China
1	5	3	13	2	2	3	2	4	41	8	6	4	3	5	4	2017Liu#034	B. melitensis	42	116		WS033	Human	2015	Liangcheng, China
1	5	3	13	2	2	3	2	4	41	8	6	4	3	5	4	2017Liu#090	B. melitensis	42	116		WS089	Human	2015	Xinghe, China
1	5	3	13	2	2	3	2	4	41	8	6	4	3	5	4	2017Liu#092	B. melitensis	42	116		WS091	Human	2015	Liangcheng, China
1	5	3	13	2	2	3	2	4	41	8	6	4	3	5	4	2017Liu#093	B. melitensis	42	116		WS092	Human	2015	Liangcheng, China
1	5	3	13	2	2	3	2	4	41	8	6	4	3	5	4	2017Liu#099	B. melitensis	42	116		WS098	Human	2015	Qianqi, China
1	5	3	13	2	2	3	2	4	41	8	6	4	3	5	4	2017Liu#102	B. melitensis	42	116		WS101	Human	2015	Liangcheng, China
1	5	3	13	2	2	3	2	4	41	8	6	4	3	5	4	2017Liu#105	B. melitensis	42	116		WS104	Human	2015	Liangcheng, China
1	5	3	13	2	2	3	2	4	41	8	6	4	3	5	11	2017Liu#015	B. melitensis	42	116		WS014	Human	2014	Liangcheng, China
1	5	3	13	2	2	3	2	4	41	8	8	4	3	6	4	2017Liu#060	B. melitensis	42	116		WS059	Human	2015	Zhongqi, China
1	5	3	13	2	2	3	2	4	41	8	8	4	3	6	4	2017Liu#064	B. melitensis	42	116		WS063	Human	2015	Zhongqi, China
1	5	3	13	2	2	3	2	4	41	8	8	4	3	6	4	2017Liu#070	B. melitensis	42	116		WS069	Human	2015	Jining, China
1	5	3	13	2	2	3	2	4	41	8	8	4	3	6	4	2017Liu#076	B. melitensis	42	116		WS075	Human	2015	Jining, China
1	5	3	13	2	2	3	2	4	41	8	8	4	3	6	4	2017Liu#084	B. melitensis	42	116		WS083	Human	2015	Jining, China
1	5	3	13	2	2	3	2	4	41	8	8	4	3	5	4	2017Liu#050	B. melitensis	42	116		WS049	Human	2015	Liangcheng, China
1	5	3	13	2	2	3	2	4	41	8	8	4	3	5	4	2017Liu#096	B. melitensis	42	116		WS095	Human	2015	Fengzhen, China
1	5	3	13	2	2	3	2	4	41	8	8	4	3	5	4	2017Liu#112	B. melitensis	42	116		WS111	Human	2015	Fengzhen, China
1	5	3	13	2	2	3	2	4	41	8	8	4	3	7	4	2017Liu#083	B. melitensis	42	116		WS082	Human	2015	Qianqi, China
1	5	3	13	2	2	3	2	4	41	8	7	4	3	6	4	2017Liu#073	B. melitensis	42	116		WS072	Human	2015	Qianqi, China
1	5	3	13	2	2	3	2	4	41	8	7	4	3	6	4	2017Liu#077	B. melitensis	42	116		WS076	Human	2015	Qianqi, China
1	5	3	13	2	2	3	2	4	41	8	7	4	3	6	4	2017Liu#106	B. melitensis	42	116		WS105	Human	2015	Qianqi, China
1	5	3	13	2	2	3	2	4	41	8	7	4	3	6	4	2017Liu#114	B. melitensis	42	116		WS113	Human	2015	Qianqi, China
1	5	3	13	2	2	3	2	4	41	8	7	4	3	7	4	2017Liu#067	B. melitensis	42	116		WS066	Human	2015	Qianqi, China
1	5	3	13	2	2	3	2	4	41	8	7	4	3	7	4	2017Liu#075	B. melitensis	42	116		WS074	Human	2015	Datong, China
1	5	3	13	2	2	3	2	4	41	8	7	4	3	5	4	2017Liu#039	B. melitensis	42	116		WS038	Human	2015	Qianqi, China
1	5	3	13	2	2	3	2	4	41	8	7	4	3	5	4	2017Liu#055	B. melitensis	42	116		WS054	Human	2015	Xinghe, China
1	5	3	13	2	2	3	2	4	41	8	7	4	3	5	4	2017Liu#063	B. melitensis	42	116		WS062	Human	2015	Shangdu, China
1	5	3	12	2	2	3	2	4	41	8	7	4	3	5	4	2006LeFleche#010	B. melitensis bv2	45	115		REF 63/9	Caprine	1963	India
1	5	3	12	2	2	3	2	4	41	8	7	4	3	5	4	2012Ferreira#002	B. melitensis bv2	45	115		REF 63/9	Caprine	1963	India
1	5	3	12	2	2	3	2	4	41	8	7	4	3	5	4	2018Vergnaud#0002	B. melitensis bv2	45	115		BCCN#R2	Caprine		Turkey
1	5	3	12	2	2	3	2	4	41	8	7	4	3	5	4	SRR011160	B. melitensis bv2	45	115	8	REF 63/9	Caprine	1963	India
1	5	3	12	2	2	3	2	4	41	8	8	4	3	5	4	GCA_000022625	B. melitensis	45	115	8	REF 63/9 (ATC.	Caprine	1963	India
1	5	3	13	2	2	3	2	4	41	8	5	6	3	5	4	2012Ferreira#030	B. melitensis bv3	42	116		AFSSA-05/3515	unknown	2005	Portugal
1	5	3	13	2	2	3	2	4	41	8	5	8	3	5	4	2013Jiang#033	B. melitensis bv2	42	116		HN72-003	Ovine	1972	Henan, China
1	5	3	13	2	2	3	2	4	41	8	5	8	3	5	7	2018Vergnaud#0491	B. melitensis bv3	42	116		BCCN#00-5	Human	2000	Navarre, Spain
1	5	3	13	2	2	3	2	4	41	8	5	9	3	5	3	2018Vergnaud#0492	B. melitensis bv3	42	116		BCCN#01-19	Human	2001	Portugal
1	5	3	12	2	2	3	2	4	41	8	5	4	3	9	8	2011Jiang#048	B. melitensis bv3	45	115		SHANXI07-2	Human	2007	Shanxi, China
1	5	3	13	2	2	3	2	4	41	8	5	4	3	9	9	2011Jiang#097	B. melitensis bv1	42	116		NM96-0619	Human	1996	Inner Mongolia, China
1	5	3	13	2	2	3	2	4	41	8	6	7	3	5	5	2018Vergnaud#0052	B. melitensis bv3	42	116		BCCN#92-83	Ovine	1992	Murcie ,Spain
1	5	3	13	2	2	3	2	4	41	8	6	7	3	5	5	2018Vergnaud#0117	B. melitensis bv3	42	116		BCCN#83-209	Human	1983	France
1	5	3	13	2	2	3	2	4	41	8	6	7	3	5	5	2018Vergnaud#0641	B. melitensis bv3	42	116		BCCN#96-165	Human	1996	Pampelune, Spain
1	5	3	13	2	2	3	2	4	41	8	6	7	3	5	5	2018Vergnaud#1243	B. melitensis bv3	42	116		BCCN#94-29	Human	1994	Paris, France
1	5	3	13	2	2	3	2	4	41	8	6	7	3	5	5	2018Vergnaud#1323	B. melitensis bv3	42	116		BCCN#92-27	Human	1992	Val-de-Marne, France
1	5	3	13	2	2	3	2	4	41	8	6	7	3	7	5	2012Ferreira#075	B. melitensis bv3	42	116		LNIV-142Bm3-03	unknown	2003	Beira Interior, Portugal
1	5	3	13	2	2	3	2	4	41	8	6	7	3	7	5	2018Vergnaud#1190	B. melitensis bv3	42	116		BCCN#75-268	Human	1975	Castille, Spain
1	5	3	13	2	2	3	2	4	41	8	6	7	3	7	5	2018Vergnaud#1206	B. melitensis bv3	42	116		BCCN#91-232	Ovine	1991	Zamora, Spain
1	5	3	13	2	2	3	2	4	41	8	6	7	3	7	5	2018Vergnaud#1392	B. melitensis bv3	42	116		BCCN#91-24	Human	1991	Paris, France
1	5	3	13	2	2	3	2	4	41	8	6	7	3	8	5	2018Vergnaud#1117	B. melitensis bv3	42	116		BCCN#75-150	Human	1975	Castille, Spain
1	5	3	13	2	2	3	2	4	41	8	6	9	3	4	5	2018Vergnaud#0540	B. melitensis bv3	42	116		BCCN#83-207	Human	1983	Spain
1	5	3	13	2	2	3	2	4	41	8	6	9	3	4	5	2018Vergnaud#1262	B. melitensis bv3	42	116		BCCN#83-204	Human	1983	Spain
1	5	3	13	2	2	3	2	4	41	8	6	4	3	4	5	2017Liu#006	B. melitensis	42	116		WS005	Human	2012	Qianqi, China
1	5	3	13	2	2	3	2	4	41	8	6	4	3	4	5	2017Liu#173	B. melitensis bv3	42	116		326-8		2015	
1	5	3	13	2	2	3	2	4	41	8	6	8	3	4	5	2012Ferreira#028	B. melitensis bv3	42	116		AFSSA-03/480.	unknown	2003	Portugal
1	5	3	13	2	2	3	2	4	41	8	6	8	3	4	5	2012Ferreira#066	B. melitensis bv3	42	116		LNIV-649Bm3-07	unknown	2007	Beira Litoral, Portugal
1	5	3	13	2	2	3	2	4	41	8	6	8	3	4	5	2012Ferreira#072	B. melitensis bv3	42	116		LNIV-73Bm3-01	unknown	2001	Beira Interior, Portugal
1	5	3	13	2	2	3	2	4	41	8	6	8	3	4	5	2012Ferreira#082	B. melitensis bv3	42	116		LNIV-45Bm3-03	unknown	2003	Ribatejo e Oeste, Portu.
1	5	3	13	2	2	3	2	4	41	8	6	8	3	4	5	2018Vergnaud#1267	B. melitensis bv3	42	116		BCCN#89-134	Ovine	1989	Saragosse, Spain
1	5	3	13	2	2	3	2	4	41	8	6	7	3	4	5	2012Ferreira#005	B. melitensis bv3	42	116		LNIV-912Bm3-09	unknown	2009	Ribatejo e Oeste, Portu.
1	5	3	13	2	2	3	2	4	41	8	6	7	3	4	5	2012Ferreira#062	B. melitensis bv3	42	116		LNIV-126Bm3-03	unknown	2003	Beira Litoral, Portugal
1	5	3	13	2	2	3	2	4	41	8	6	7	3	4	5	2012Ferreira#069	B. melitensis bv3	42	116		LNIV-721Bm3-07	unknown	2007	Beira Litoral, Portugal
1	5	3	13	2	2	3	2	4	41	8	6	7	3	4	5	2012Ferreira#074	B. melitensis bv3	42	116		LNIV-101Bm3-03	unknown	2003	Beira Interior, Portugal
1	5	3	13	2	2	3	2	4	41	8	6	7	3	4	5	2012Ferreira#093	B. melitensis bv3	42	116		LNIV-194Bm3-03	unknown	2003	Alentejo, Portugal
1	5	3	13	2	2	3	2	4	41	8	6	7	3	4	5	2018Vergnaud#0352	B. melitensis R.	42	116		BCCN#82-61	Human	1982	Asturies, Spain
1	5	3	13	2	2	3	2	4	41	8	6	7	3	4	5	2018Vergnaud#0382	B. melitensis bv3	42	116		BCCN#82-87	Human	1982	Paris, France
1	5	3	13	2	2	3	2	4	41	8	6	7	3	4	5	2018Vergnaud#0393	B. melitensis bv3	42	116		BCCN#85-12	Human	1985	Portugal
1	5	3	13	2	2	3	2	4	41	8	6	7	3	4	5	2018Vergnaud#0413	B. melitensis bv3	42	116		BCCN#94-36	Human	1994	Portugal
1	5	3	13	2	2	3	2	4	41	8	6	7	3	4	5	2018Vergnaud#0596	B. melitensis bv3	42	116		BCCN#85-13	Human	1985	Clamart ,Paris, France
1	5	3	13	2	2	3	2	4	41	8	6	7	3	4	5	2018Vergnaud#0602	B. melitensis bv3	42	116		BCCN#91-219	Ovine	1991	Teruel, Spain
1	5	3	13	2	2	3	2	4	41	8	6	7	3	4	5	2018Vergnaud#0668	B. melitensis bv3	42	116		BCCN#98-52	Human	1998	Vaucluse, France
1	5	3	13	2	2	3	2	4	41	8	6	7	3	4	5	2018Vergnaud#0671	B. melitensis bv3	42	116		BCCN#98-98	Human	1998	Paris, France
1	5	3	13	2	2	3	2	4	41	8	6	7	3	4	5	2018Vergnaud#0738	B. melitensis bv3	42	116		BCCN#87-28	Cattle	1987	Saragosse, Spain
1	5	3	13	2	2	3	2	4	41	8	6	7	3	4	5	2018Vergnaud#0845	B. melitensis bv3	42	116		BCCN#92-91	Cattle	1992	Navarre, Spain
1	5	3	13	2	2	3	2	4	41	8	6	7	3	4	5	2018Vergnaud#0855	B. melitensis bv3	42	116		BCCN#97-1	Human	1997	Seine-et-Marne, France
1	5	3	13	2	2	3	2	4	41	8	6	7	3	4	5	2018Vergnaud#0896	B. melitensis bv3	42	116		BCCN#79-185	Human	1979	Spain
1	5	3	13	2	2	3	2	4	41	8	6	7	3	4	5	2018Vergnaud#0903	B. melitensis bv3	42	116		BCCN#86-86	Ovine	1986	Saragosse, Spain
1	5	3	13	2	2	3	2	4	41	8	6	7	3	4	5	2018Vergnaud#0904	B. melitensis bv3	42	116		BCCN#86-87	Ovine	1986	Saragosse, Spain
1	5	3	13	2	2	3	2	4	41	8	6	7	3	4	5	2018Vergnaud#0906	B. melitensis bv3	42	116		BCCN#87-37	Cattle	1987	Saragosse, Spain
1	5	3	13	2	2	3	2	4	41	8	6	7	3	4	5	2018Vergnaud#0923	B. melitensis bv3	42	116		BCCN#75-144	Human	1975	Castille, Spain
1	5	3	13	2	2	3	2	4	41	8	6	7	3	4	5	2018Vergnaud#0962	B. melitensis bv3	42	116		BCCN#02-7	Human	2002	France
1	5	3	13	2	2	3	2	4	41	8	6	7	3	4	5	2018Vergnaud#1008	B. melitensis bv3	42	116		BCCN#92-78	Ovine	1992	Aragon, Spain
1	5	3	13	2	2	3	2	4	41	8	6	7	3	4	5	2018Vergnaud#1041	B. melitensis bv3	42	116		BCCN#80-88	Human	1980	Pampelune, Spain
1	5	3	13	2	2	3	2	4	41	8	6	7	3	4	5	2018Vergnaud#1042	B. melitensis bv3	42	116		BCCN#82-70	Human	1982	Barcelone,Spain
1	5	3	13	2	2	3	2	4	41	8	6	7	3	4	5	2018Vergnaud#1045	B. melitensis bv3	42	116		BCCN#86-88	Ovine	1986	Saragosse, Spain
1	5	3	13	2	2	3	2	4	41	8	6	7	3	4	5	2018Vergnaud#1091	B. melitensis bv3	42	116		BCCN#97-109	Human	1997	Hauts-de-Seine, France
1	5	3	13	2	2	3	2	4	41	8	6	7	3	4	5	2018Vergnaud#1114	B. melitensis bv3	42	116		BCCN#75-132	Human	1975	Castille, Spain
1	5	3	13	2	2	3	2	4	41	8	6	7	3	4	5	2018Vergnaud#1127	B. melitensis bv3	42	116		BCCN#75-282	Human	1975	Castille, Spain

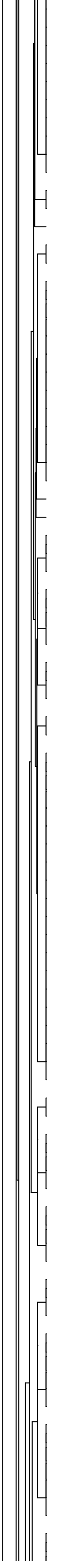

1	5	3	13	2	2	3	2	4	41	8	6	7	3	4	5	2018Vergnaud#1154	B. melitensis bv3	42	116	BCCN#87-30	Cattle	1987	Saragosse, Spain
1	5	3	13	2	2	3	2	4	41	8	6	7	3	4	5	2018Vergnaud#1155	B. melitensis bv3	42	116	BCCN#87-31	Cattle	1987	Saragosse, Spain
1	5	3	13	2	2	3	2	4	41	8	6	7	3	4	5	2018Vergnaud#1173	B. melitensis bv3	42	116	BCCN#91-38	Ovine	1991	Saragosse, Spain
1	5	3	13	2	2	3	2	4	41	8	6	7	3	4	5	2018Vergnaud#1188	B. melitensis bv3	42	116	BCCN#75-157	Human	1975	Castille, Spain
1	5	3	13	2	2	3	2	4	41	8	6	7	3	4	5	2018Vergnaud#1192	B. melitensis bv3	42	116	BCCN#87-32	Cattle	1987	Saragosse, Spain
1	5	3	13	2	2	3	2	4	41	8	6	7	3	4	5	2018Vergnaud#1193	B. melitensis bv3	42	116	BCCN#87-33	Cattle	1987	Saragosse, Spain
1	5	3	13	2	2	3	2	4	41	8	6	7	3	4	5	2018Vergnaud#1194	B. melitensis bv3	42	116	BCCN#89-137	Ovine	1989	Saragosse, Spain
1	5	3	13	2	2	3	2	4	41	8	6	7	3	4	5	2018Vergnaud#1197	B. melitensis bv3	42	116	BCCN#75-258	Human	1975	Castille, Spain
1	5	3	13	2	2	3	2	4	41	8	6	7	3	4	5	2018Vergnaud#1205	B. melitensis bv3	42	116	BCCN#91-231	Ovine	1991	Zamora, Spain
1	5	3	13	2	2	3	2	4	41	8	6	7	3	4	5	2018Vergnaud#1383	B. melitensis bv3	42	116	BCCN#87-29	Cattle	1987	Saragosse, Spain
1	5	3	13	2	2	3	2	4	41	8	6	7	3	4	7	2012Ferreira#071	B. melitensis bv3	42	116	LNIV-47Bm3-01	unknown	2001	Beira Interior, Portugal
1	5	3	13	2	2	3	2	4	41	8	6	7	3	4	7	2018Vergnaud#0499	B. melitensis bv3	42	116	BCCN#76-419	Human	1976	Navarre, Spain
1	5	3	13	2	2	3	2	5	41	8	6	7	3	4	5	2012Ferreira#063	B. melitensis bv3	42	180	LNIV-128Bm3-03	unknown	2003	Beira Litoral, Portugal
1	5	3	13	2	2	3	2	5	41	8	7	7	3	4	5	2018Vergnaud#1094	B. melitensis bv3	42	180	BCCN#97-96	Human	1997	Navarre, Spain
1	5	3	13	2	2	3	2	5	41	8	7	7	3	4	5	2018Vergnaud#1095	B. melitensis bv3	42	180	BCCN#97-97	Human	1997	Navarre, Spain
1	5	3	13	2	2	3	2	4	41	8	7	7	3	4	5	2012Ferreira#022	B. melitensis	42	116	AFSSA-02/6425	unknown	2002	Portugal
1	5	3	13	2	2	3	2	4	41	8	7	7	3	4	5	2012Ferreira#025	B. melitensis bv3	42	116	AFSSA-03/0346	unknown	2003	Portugal
1	5	3	13	2	2	3	2	4	41	8	7	7	3	4	5	2012Ferreira#047	B. melitensis bv3	42	116	LNIV-489Bm3-05	unknown	2005	Tras-os-Montes, Portugal
1	5	3	13	2	2	3	2	4	41	8	7	7	3	4	5	2012Ferreira#070	B. melitensis bv3	42	116	LNIV-722Bm3-07	unknown	2007	Beira Litoral, Portugal
1	5	3	13	2	2	3	2	4	41	8	7	7	3	4	5	2012Ferreira#073	B. melitensis bv3	42	116	LNIV-9Bm3-02	unknown	2002	Beira Interior, Portugal
1	5	3	13	2	2	3	2	4	41	8	7	7	3	4	5	2012Ferreira#080	B. melitensis bv3	42	116	LNIV-610Bm3-06	unknown	2006	Beira Interior, Portugal
1	5	3	13	2	2	3	2	4	41	8	7	7	3	4	5	2018Vergnaud#0131	B. melitensis bv3	42	116	BCCN#77-145	Cattle	1977	Navarre, Spain
1	5	3	13	2	2	3	2	4	41	8	7	7	3	4	5	2018Vergnaud#0665	B. melitensis bv3	42	116	BCCN#98-19	Human	1998	Loire-Atlantique, France
1	5	3	13	2	2	3	2	4	41	8	7	7	3	4	5	2018Vergnaud#0961	B. melitensis bv3	42	116	BCCN#02-39	Human	2002	France
1	5	3	13	2	2	3	2	4	41	8	7	7	3	4	5	2018Vergnaud#1003	B. melitensis bv3	42	116	BCCN#91-241	Human	1991	France
1	5	3	13	2	2	3	2	4	41	8	7	7	3	4	5	2018Vergnaud#1103	B. melitensis bv3	42	116	BCCN#99-97	Human	1999	Ariège, France
1	5	3	13	2	2	3	2	4	41	8	7	7	3	4	5	2018Vergnaud#1183	B. melitensis bv3	42	116	BCCN#93-64	Human	1993	Pyrénées-Atlantiques, .
1	5	3	13	2	2	3	2	4	41	8	7	8	3	4	5	2018Vergnaud#1007	B. melitensis bv3	42	116	BCCN#92-22	Human	1992	Paris, France
1	5	3	13	2	2	3	2	4	41	8	7	7	11	4	5	2018Vergnaud#0369	B. melitensis R.	42	116	BCCN#02-46	Human	2002	Seine-et-Marne, France
1	5	3	13	2	2	3	2	4	41	8	9	7	3	4	5	2012Ferreira#061	B. melitensis bv3	42	116	LNIV-51Bm3-01	unknown	2001	Beira Litoral, Portugal
1	5	3	13	2	2	3	2	4	41	8	9	7	3	4	5	2012Ferreira#076	B. melitensis bv3	42	116	LNIV-265Bm3-04	unknown	2004	Beira Interior, Portugal
1	5	3	13	2	2	3	2	4	41	8	9	7	3	4	5	2012Ferreira#083	B. melitensis bv3	42	116	LNIV-104Bm3-03	unknown	2003	Ribatejo e Oeste, Portu.
1	5	3	13	2	2	3	2	4	41	8	3	7	3	4	5	2012Ferreira#060	B. melitensis bv3	42	116	LNIV-531Bm3-06	unknown	2006	Tras-os-Montes, Portugal
1	5	3	13	2	2	3	2	4	41	8	3	7	3	4	5	2018Vergnaud#0979	B. melitensis bv3	42	116	BCCN#91-33	Ovine	1991	Saragosse, Spain
1	5	3	13	2	2	3	2	4	41	8	3	7	3	4	5	2018Vergnaud#0980	B. melitensis bv3	42	116	BCCN#91-34	Ovine	1991	Saragosse, Spain
1	5	3	13	2	2	3	2	4	41	8	3	7	3	4	5	2018Vergnaud#1142	B. melitensis bv3	42	116	BCCN#91-223	Ovine	1991	Huesca, Spain
1	5	3	13	2	2	3	2	4	41	8	8	7	3	4	5	2007AlDahouk#109	B. melitensis bv3	42	116	BfR 88	Human	1998	Leipzig, Germany
1	5	3	13	2	2	3	2	4	41	8	8	7	3	4	5	2018Vergnaud#0490	B. melitensis bv3	42	116	BCCN#00-14	Human	2000	Seine-et-Marne, France
1	5	3	13	2	2	3	2	4	41	8	8	7	3	4	5	2018Vergnaud#1126	B. melitensis bv3	42	116	BCCN#75-264	Human	1975	Castille, Spain
1	5	3	13	2	2	3	2	4	41	6	5	7	3	4	5	2018Vergnaud#1265	B. melitensis bv3	42	294	BCCN#89-132	Ovine	1989	Saragosse, Spain
1	5	3	13	2	2	3	2	4	41	6	5	7	3	4	5	2018Vergnaud#1266	B. melitensis bv3	42	294	BCCN#89-133	Ovine	1989	Saragosse, Spain
1	5	3	13	2	2	3	2	4	41	8	5	7	3	4	5	2007AlDahouk#014	B. melitensis bv3	42	116	BCCN#96-142	Human	1996	Lyon, France
1	5	3	13	2	2	3	2	4	41	8	5	7	3	4	5	2012Ferreira#112	B. melitensis bv3	42	116	LNIV-384Bm3-05	unknown	2005	Algarve, Portugal
1	5	3	13	2	2	3	2	4	41	8	5	7	3	4	5	2012Ferreira#126	B. melitensis bv3	42	116	LNIV-918Bm3-11	unknown	2011	Beira Litoral, Portugal
1	5	3	13	2	2	3	2	4	41	8	5	7	3	4	5	2012Ferreira#128	B. melitensis bv3	42	116	LNIV-920Bm3-11	unknown	2011	Beira Litoral, Portugal
1	5	3	13	2	2	3	2	4	41	8	5	7	3	4	5	2012Ferreira#129	B. melitensis bv3	42	116	LNIV-921Bm3-11	unknown	2011	Beira Litoral, Portugal
1	5	3	13	2	2	3	2	4	41	8	5	7	3	4	5	2012Ferreira#130	B. melitensis bv3	42	116	LNIV-922Bm3-11	unknown	2011	Beira Litoral, Portugal
1	5	3	13	2	2	3	2	4	41	8	5	7	3	4	5	2012Ferreira#132	B. melitensis bv3	42	116	LNIV-924Bm3-11	unknown	2011	Beira Litoral, Portugal
1	5	3	13	2	2	3	2	4	41	8	5	7	3	4	5	2018Vergnaud#0636	B. melitensis bv3	42	116	BCCN#96-13	Human	1996	Portugal
1	5	3	13	2	2	3	2	4	41	8	5	7	3	4	5	2018Vergnaud#0637	B. melitensis bv3	42	116	BCCN#96-14	Human	1996	Portugal
1	5	3	13	2	2	3	2	4	41	8	5	7	3	4	5	2018Vergnaud#0670	B. melitensis bv3	42	116	BCCN#98-7	Human	1998	Pamplona, Spain
1	5	3	13	2	2	3	2	4	41	8	5	7	3	4	5	2018Vergnaud#0819	B. melitensis bv3	42	116	BCCN#75-301	Human	1975	Castille, Spain
1	5	3	13	2	2	3	2	4	41	8	5	7	3	4	5	2018Vergnaud#0848	B. melitensis bv3	42	116	BCCN#96-155	Human	1996	Pampelune, Spain
1	5	3	13	2	2	3	2	4	41	8	5	7	3	4	5	2018Vergnaud#0905	B. melitensis bv3	42	116	BCCN#87-36	Cattle	1987	Saragosse, Spain
1	5	3	13	2	2	3	2	4	41	8	5	7	3	4	5	2018Vergnaud#0982	B. melitensis bv3	42	116	BCCN#97-93	Human	1997	Navarre, Spain
1	5	3	13	2	2	3	2	4	41	8	5	7	3	4	5	2018Vergnaud#0983	B. melitensis bv3	42	116	BCCN#97-94	Human	1997	Navarre, Spain
1	5	3	13	2	2	3	2	4	41	8	5	7	3	4	5	2018Vergnaud#1119	B. melitensis bv3	42	116	BCCN#75-152	Human	1975	Castille, Spain
1	5	3	13	2	2	3	2	4	41	8	5	7	3	4	5	2018Vergnaud#1241	B. melitensis bv3	42	116	BCCN#94-26	Human	1994	Correze, France
1	5	3	13	2	2	3	2	4	41	8	5	7	3	4	5	2018Vergnaud#1277	B. melitensis bv3	42	116	BCCN#93-67	Human	1993	Tarn-et-Garonne, France
1	5	3	13	2	2	3	2	4	41	8	5	7	3	4	5	2018Vergnaud#1298	B. melitensis bv3	42	116	BCCN#75-273	Human	1975	Castille, Spain
1	5	3	13	2	2	3	2	4	41	8	4	4	3	4	5	2013Jiang#021	B. melitensis bv3	42	116	SHX84-1	Ovine	1984	Shanxi, China
1	5	3	13	2	2	3	2	4	41	8	4	4	3	4	5	2015Shevtsov#181	B. melitensis	42	116	040068	Ovine	2012	Enbekshikazakh,Almaty.
1	5	3	13	2	2	3	2	4	41	8	4	6	3	4	5	2012Ferreira#056	B. melitensis bv3	42	116	LNIV-519Bm3-06	unknown	2006	Tras-os-Montes, Portugal
1	5	3	13	2	2	3	2	4	41	8	4	6	3	4	5	2012Ferreira#068	B. melitensis bv3	42	116	LNIV-719Bm3-07	unknown	2007	Beira Litoral, Portugal
1	5	3	13	2	2	3	2	4	41	8	4	6	3	4	5	2012Ferreira#081	B. melitensis bv3	42	116	LNIV-744Bm3-07	unknown	2007	Beira Interior, Portugal
1	5	3	13	2	2	3	2	4	41	8	4	6	3	4	5	2018Vergnaud#0547	B. melitensis bv3	42	116	BCCN#00-4	Human	2000	Portugal
1	5	3	13	2	2	3	2	4	41	8	4	7	3	4	5	2012Ferreira#004	B. melitensis bv3	42	116	LNIV-913Bm3-10	unknown	2010	Ribatejo e Oeste, Portu.
1	5	3	13	2	2	3	2	4	41	8	4	7	3	4	5	2012Ferreira#104	B. melitensis bv3	42	116	LNIV-202Bm3-03	unknown	2003	Algarve, Portugal
1	5	3	13	2	2	3	2	4	41	8	4	7	3	4	5	2018Vergnaud#0497	B. melitensis bv3	42	116	BCCN#01-24	Human	2001	France
1	5	3	13	2	2	3	2	4	41	8	4	7	3	4	5	2018Vergnaud#1087	B. melitensis bv3	42	116	BCCN#96-156	Human	1996	St Gaudens ,Haute-Ga.
1	5	3	13	2	2	3	2	4	41	8	4	7	3	5	5	2011Kilic#102	B. melitensis bv3	42	116	BRU-S102	Human	2008	Istanbul, Turkey
1	5	3	13	2	2	3	2	4	41	8	4	7	3	5	5	2012Ferreira#018	B. melitensis bv3	42	116	LNIV-520Bm3-06	unknown	2006	Alentejo, Portugal
1	5	3	13	2	2	3	2	4	41	8	4	7	3	5	5	2018Vergnaud#0995	B. melitensis bv3	42	116	BCCN#75-311	Human	1975	Castille, Spain
1	5	3	13	2	2	3	2	4	41	8	7	7	3	5	5	2011Kilic#061	B. melitensis bv3	42	116	BRU-S061	Human	2002	Kirikkale, Turkey
1	5	3	13	2	2	3	2	4	41	8	7	7	3	5	5	2011Kilic#076	B. melitensis bv3	42	116	BRU-S076	Human	2002	Kirikkale, Turkey
1	5	3	13	2	2	3	2	4	41	8	7	7	3	5	5	2012Ferreira#077	B. melitensis bv3	42	116	LNIV-321Bm3-04	unknown	2004	Beira Interior, Portugal
1	5	3	13	2	2	3	2	4	41	8	7	7	3	5	5	2018Vergnaud#0859	B. melitensis bv3	42	116	BCCN#99-89	Human	1999	Ille-et-Vilaine, France
1	5	3	13	2	2	3	2	4	41	8	7	7	3	5	5	2018Vergnaud#1102	B. melitensis bv3	42	116	BCCN#99-96	Human	1999	Rennes ,Ille-et-Vilaine, .
1	5	3	13	2	2	3	2	4	41	8	5	7	3	5	5	2007AlDahouk#020	B. melitensis bv3	42	116	BCCN#03-1	Human	2003	Paris, France
1	5	3	13	2	2	3	2	4	41	8	5	7	3	5	5	2012Ferreira#020	B. melitensis bv3	42	116	AFSSA-02/4222	unknown	2002	Portugal
1	5	3	13	2	2	3	2	4	41	8	5	7	3	5	5	2012Ferreira#023	B. melitensis bv3	42	116	AFSSA-02/7384	unknown	2002	Portugal
1	5	3	13	2	2	3	2	4	41	8	5	7	3	5	5	2017Hanot-Mamb.	B. melitensis bv3	42	116	L3/14	Human	1997	Belgium
1	5	3	13	2	2	3	2	4	41	8	5	7	3	5	5	2018Vergnaud#0116	B. melitensis bv3	42	116	BCCN#83-197	Human	1983	Spain
1	5	3	13	2	2	3	2	4	41	8	5	7	3	5	5	2018Vergnaud#1186	B. melitensis bv3	42	116	BCCN#75-148	Human	1975	Castille, Spain
1	5	3	13	2	2	3	2	4	41	8	5	7	3	8	5	2012Ferreira#100	B. melitensis bv3	42	116	LNIV-595Bm3-06	unknown	2006	Alentejo, Portugal
1	5	3	13	2	2	3	2	4	41	8	5	7	3	8	5	2012Ferreira#101	B. melitensis bv3	42	116	LNIV-596Bm3-06	unknown	2006	Alentejo, Portugal

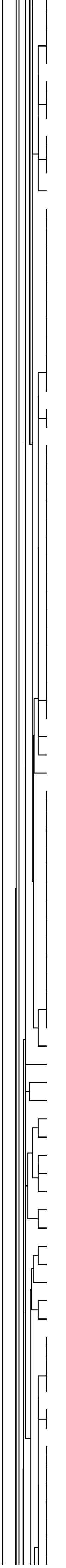

1	5	3	13	2	2	3	2	4	41	8	5	7	3	8	5	2012Ferreira#110	B. melitensis bv3	42	116	LNIV-302Bm3-04	unknown	2004	Algarve, Portugal
1	5	3	13	2	2	3	2	4	41	8	5	7	3	8	5	2017Hanot-Mamb.	B. melitensis bv3	42	116	L3/137	Human	2004	Belgium
1	5	3	13	2	2	3	2	4	41	8	5	7	3	8	5	2018Vergnaud#1129	B. melitensis bv3	42	116	BCCN#75-284	Human	1975	Castille, Spain
1	5	3	13	2	2	3	2	4	41	8	5	7	3	8	5	2018Vergnaud#1189	B. melitensis bv3	42	116	BCCN#75-158	Human	1975	Castille, Spain
1	5	3	13	2	2	3	2	4	41	8	7	7	3	8	5	2012Ferreira#024	B. melitensis bv3	42	116	AFSSA-03/0342	unknown	2003	Portugal
1	5	3	13	2	2	3	2	4	41	8	7	7	3	8	5	2018Vergnaud#0374	B. melitensis bv3	42	116	BCCN#03-18	Human	2003	Portugal
1	5	3	13	2	2	3	2	4	41	8	7	7	3	8	5	2018Vergnaud#0814	B. melitensis bv3	42	116	BCCN#75-181	Human	1975	Castille, Spain
1	5	3	13	2	2	3	2	4	41	8	4	7	3	8	5	2011Kilic#157	B. melitensis bv3	42	116	BRU-S157	Human	2005	Malatya, Turkey
1	5	3	13	2	2	3	2	4	41	8	4	7	3	8	5	2011Kilic#158	B. melitensis bv3	42	116	BRU-S158	Human	2005	Malatya, Turkey
1	5	3	13	2	2	3	2	4	41	8	4	7	3	8	5	2011Kilic#159	B. melitensis bv3	42	116	BRU-S159	Human	2005	Malatya, Turkey
1	5	3	13	2	2	3	2	4	41	8	9	7	3	8	5	2012Ferreira#103	B. melitensis bv3	42	116	LNIV-122Bm3-03	unknown	2003	Algarve, Portugal
1	5	3	13	2	2	3	2	4	41	8	6	7	3	6	5	2018Vergnaud#0537	B. melitensis bv3	42	116	BCCN#83-201	Human	1983	Spain
1	5	3	13	2	2	3	2	4	41	8	6	7	3	6	5	2018Vergnaud#0538	B. melitensis bv3	42	116	BCCN#83-202	Human	1983	Spain
1	5	3	13	2	2	3	2	4	41	8	6	7	3	6	5	2018Vergnaud#0539	B. melitensis bv3	42	116	BCCN#83-203	Human	1983	Spain
1	5	3	13	2	2	3	2	4	41	8	6	7	3	6	5	2018Vergnaud#0813	B. melitensis bv3	42	116	BCCN#75-131	Human	1975	Castille, Spain
1	5	3	13	2	2	3	2	4	41	8	6	7	3	6	5	2018Vergnaud#0893	B. melitensis bv3	42	116	BCCN#75-142	Human	1975	Castille, Spain
1	5	3	13	2	2	3	2	4	41	8	6	7	3	6	5	2018Vergnaud#0894	B. melitensis bv3	42	116	BCCN#75-261	Human	1975	Castille, Spain
1	5	3	13	2	2	3	2	4	41	8	6	7	3	6	5	2018Vergnaud#1115	B. melitensis bv3	42	116	BCCN#75-133	Human	1975	Castille, Spain
1	5	3	13	2	2	3	2	4	41	8	6	7	3	6	5	2018Vergnaud#1123	B. melitensis bv3	42	116	BCCN#75-260	Human	1975	Castille, Spain
1	5	3	13	2	2	3	2	4	41	8	6	7	3	6	5	2018Vergnaud#1300	B. melitensis bv3	42	116	BCCN#75-291	Human	1975	Castille, Spain
1	5	3	13	2	2	3	2	4	41	8	6	7	3	6	5	2018Vergnaud#1382	B. melitensis bv3	42	116	BCCN#83-206	Human	1983	Spain
1	5	3	13	2	2	3	2	4	41	8	6	7	3	6	5	2018Vergnaud#1394	B. melitensis bv3	42	116	BCCN#94-43	Human	1994	Haute-Garonne, France
1	5	3	13	2	2	3	2	4	41	8	7	7	3	6	5	2018Vergnaud#0066	B. melitensis bv3	42	116	BCCN#94-65	Human	1994	Hérault, France
1	5	3	13	2	2	3	2	4	41	8	7	7	3	6	5	2018Vergnaud#1187	B. melitensis bv3	42	116	BCCN#75-149	Human	1975	Castille, Spain
1	5	3	13	2	2	3	2	4	41	8	5	7	3	6	5	2007AlDahouk#012	B. melitensis bv3	42	116	BCCN#83-198	Human	1983	Spain
1	5	3	13	2	2	3	2	4	41	8	5	7	3	6	5	2012Ferreira#057	B. melitensis bv3	42	116	LNIV-522Bm3-06	unknown	2006	Tras-os-Montes, Portugal
1	5	3	13	2	2	3	2	4	41	8	5	7	3	6	5	2018Vergnaud#0122	B. melitensis bv3	42	116	BCCN#75-313	Human	1975	Castille, Spain
1	5	3	13	2	2	3	2	4	41	8	5	7	3	6	5	2018Vergnaud#0526	B. melitensis bv3	42	116	BCCN#00-1	Human	2000	Navarre, Spain
1	5	3	13	2	2	3	2	4	41	8	5	7	3	6	5	2018Vergnaud#0666	B. melitensis bv3	42	116	BCCN#98-3	Human	1998	Pampelune, Spain
1	5	3	13	2	2	3	2	4	41	8	5	7	3	6	5	2018Vergnaud#0695	B. melitensis bv3	42	116	BCCN#99-86	Human	1999	Navarre, Spain
1	5	3	13	2	2	3	2	4	41	8	5	7	3	6	5	2018Vergnaud#0740	B. melitensis bv3	42	116	BCCN#88-34	Human	1988	Yvelines, France
1	5	3	13	2	2	3	2	4	41	8	5	7	3	6	5	2018Vergnaud#0892	B. melitensis bv3	42	116	BCCN#75-141	Human	1975	Castille, Spain
1	5	3	13	2	2	3	2	4	41	8	5	7	3	6	5	2018Vergnaud#0924	B. melitensis bv3	42	116	BCCN#75-259	Human	1975	Castille, Spain
1	5	3	13	2	2	3	2	4	41	8	5	7	3	6	5	2018Vergnaud#0941	B. melitensis bv3	42	116	BCCN#75-126	Human	1975	Navarre, Spain
1	5	3	13	2	2	3	2	4	41	8	5	7	3	6	5	2018Vergnaud#1120	B. melitensis bv3	42	116	BCCN#75-171	Human	1975	Castille, Spain
1	5	3	13	2	2	3	2	4	41	8	5	7	3	6	5	2018Vergnaud#1122	B. melitensis bv3	42	116	BCCN#75-173	Human	1975	Castille, Spain
1	5	3	13	2	2	3	2	4	41	8	5	7	3	6	5	2018Vergnaud#1125	B. melitensis bv3	42	116	BCCN#75-263	Human	1975	Castille, Spain
1	5	3	13	2	2	3	2	4	41	8	5	7	3	6	5	2018Vergnaud#1191	B. melitensis bv3	42	116	BCCN#75-269	Human	1975	Castille, Spain
1	5	3	13	2	2	3	2	4	41	8	5	7	3	6	5	2018Vergnaud#1301	B. melitensis bv3	42	116	BCCN#75-294	Human	1975	Castille, Spain
1	5	3	13	2	2	3	2	4	41	8	5	7	3	6	5	2018Vergnaud#1379	B. melitensis bv3	42	116	BCCN#80-253	Human	1980	Spain
1	5	3	13	2	2	3	2	4	41	8	2	7	3	6	5	2008Kattar#007	B. melitensis	42	116	AUB BRUP-S15	Human		Lebanon
1	5	3	13	2	2	3	2	4	41	8	8	7	3	6	5	2011Kilic#014	B. melitensis bv3	42	116	BRU-S014	Human	2007	Antalya, Turkey
1	5	3	13	2	2	3	2	4	43	8	5	7	3	6	5	2007AlDahouk#099	B. melitensis bv3	42	108	BfR 77	Human	1998	Spain
1	5	3	13	2	2	3	2	4	41	8	5	7	3	7	5	2012Ferreira#007	B. melitensis bv3	42	116	LNIV-90Bm3-03	unknown	2003	Entre Douro e Minho, P.
1	5	3	13	2	2	3	2	4	41	8	5	7	3	7	5	2012Ferreira#027	B. melitensis bv3	42	116	AFSSA-03/3515	unknown	2003	Portugal
1	5	3	13	2	2	3	2	4	41	8	5	7	3	7	5	2012Ferreira#086	B. melitensis bv3	42	116	LNIV-255Bm3-04	unknown	2004	Ribatejo e Oeste, Portu.
1	5	3	13	2	2	3	2	4	41	8	5	7	3	7	5	2012Ferreira#095	B. melitensis bv3	42	116	LNIV-262Bm3-04	unknown	2004	Alentejo, Portugal
1	5	3	13	2	2	3	2	4	41	8	5	7	3	7	5	2012Ferreira#097	B. melitensis bv3	42	116	LNIV-415Bm3-05	unknown	2005	Alentejo, Portugal
1	5	3	13	2	2	3	2	4	41	8	5	7	3	7	5	2018Vergnaud#0468	B. melitensis bv3	42	116	BCCN#94-34	Human	1994	Portugal
1	5	3	13	2	2	3	2	4	41	8	5	7	3	7	5	2018Vergnaud#0737	B. melitensis bv3	42	116	BCCN#87-16	Human	1987	France
1	5	3	13	2	2	3	2	4	41	8	5	7	3	7	5	2018Vergnaud#0897	B. melitensis bv3	42	116	BCCN#79-187	Human	1979	Spain
1	5	3	13	2	2	3	2	4	41	8	5	7	3	7	5	2018Vergnaud#0922	B. melitensis bv3	42	116	BCCN#75-143	Human	1975	Castille, Spain
1	5	3	13	2	2	3	2	4	41	8	5	7	3	7	5	2018Vergnaud#1018	B. melitensis bv3	42	116	BCCN#98-17	Human	1998	Vienne, France
1	5	3	13	2	2	3	2	4	41	8	5	7	3	7	5	2018Vergnaud#1027	B. melitensis bv3	42	116	BCCN#75-312	Human	1975	Castille, Spain
1	5	3	13	2	2	3	2	4	41	8	5	7	3	7	5	2018Vergnaud#1093	B. melitensis bv3	42	116	BCCN#97-95	Human	1997	Navarre, Spain
1	5	3	13	2	2	3	2	4	41	8	5	7	3	7	5	2018Vergnaud#1272	B. melitensis bv3	42	116	BCCN#90-186	Human	1990	Burgos ,Spain
1	5	3	13	2	2	3	2	4	41	8	5	7	3	7	5	2018Vergnaud#1296	B. melitensis bv3	42	116	BCCN#75-176	Human	1975	Castille, Spain
1	5	3	13	2	2	3	2	4	41	8	5	8	3	7	5	2018Vergnaud#1198	B. melitensis bv3	42	116	BCCN#82-60	Human	1982	Madrid ,Spain
1	5	3	13	2	2	3	2	4	41	8	2	1	3	4	5	2017Liu#166	B. melitensis bv3	42	116	T05	Ovine	2016	
1	5	3	13	2	2	3	2	4	41	8	8	7	3	4	3	2018Vergnaud#0421	B. melitensis bv3	42	116	BCCN#97-57	Human	1997	Aragon, Spain
1	5	3	13	2	3	3	2	4	41	8	8	7	3	4	5	2018Vergnaud#0895	B. melitensis bv3	63	111	BCCN#75-262	Human	1975	Castille, Spain
1	5	3	13	2	2	3	2	4	41	8	4	7	3	7	6	2017Hanot-Mamb.	B. melitensis bv3	42	116	L3/289	Human	2015	Belgium
1	5	3	13	2	2	3	2	4	41	8	6	7	3	7	6	2018Vergnaud#0546	B. melitensis bv3	42	116	BCCN#00-11	Cattle	2000	Navarre, Spain
1	5	3	13	2	2	3	2	4	41	8	7	7	3	7	5	2012Ferreira#092	B. melitensis bv3	42	116	LNIV-32Bm3-02	unknown	2002	Alentejo, Portugal
1	5	3	13	2	2	3	2	4	41	8	7	7	3	7	6	2018Vergnaud#0573	B. melitensis bv3	42	116	BCCN#87-53	Ovine	1987	Murcie ,Spain
1	5	3	13	2	2	3	2	4	41	8	7	7	3	7	4	2012Ferreira#115	B. melitensis bv3	42	116	LNIV-763Bm3-04	unknown	2004	Entre Douro e Minho, P.
1	5	3	13	2	2	3	2	4	41	8	7	7	3	4	4	2012Ferreira#121	B. melitensis bv3	42	116	LNIV-769Bm3-07	unknown	2007	Entre Douro e Minho, P.
1	5	3	13	2	2	3	2	4	41	8	7	7	3	3	4	2012Ferreira#123	B. melitensis bv3	42	116	LNIV-771Bm3-07	unknown	2007	Entre Douro e Minho, P.
1	5	3	13	2	2	3	2	4	41	8	4	3	3	4	6	2018Vergnaud#0401	B. melitensis bv3	42	116	BCCN#91-112	Cattle	1991	Palampur ,India
1	5	3	13	2	2	3	2	4	41	8	4	5	3	4	6	2018Vergnaud#0404	B. melitensis bv3	42	116	BCCN#91-115	Cattle	1991	Palampur ,India
1	5	3	13	2	2	3	2	4	41	8	5	3	3	4	6	2018Vergnaud#0403	B. melitensis bv3	42	116	BCCN#91-114	Cattle	1991	Palampur ,India
1	5	3	13	2	2	3	2	4	41	8	7	5	3	4	6	2012Ferreira#064	B. melitensis bv3	42	116	LNIV-281Bm3-04	unknown	2004	Beira Litoral, Portugal
1	5	3	13	2	2	3	2	4	41	8	7	8	3	4	6	2018Vergnaud#0667	B. melitensis bv3	42	116	BCCN#98-4	Human	1998	Pampelune, Spain
1	5	3	13	2	2	3	2	4	41	8	5	7	3	4	6	2018Vergnaud#1081	B. melitensis bv3	42	116	BCCN#93-94	Cattle	1993	Pyrénées Espagnoles, .
1	5	3	13	2	2	3	2	4	41	8	5	7	3	4	6	2018Vergnaud#1082	B. melitensis bv3	42	116	BCCN#93-95	Cattle	1993	Pyrénées Espagnoles, .
1	5	3	13	2	2	3	2	4	41	8	5	7	3	4	6	2018Vergnaud#1083	B. melitensis bv3	42	116	BCCN#93-96	Cattle	1993	Pyrénées Espagnoles, .
1	5	3	13	2	2	3	2	4	41	8	5	7	3	4	6	2018Vergnaud#1084	B. melitensis bv3	42	116	BCCN#93-97	Cattle	1993	Pyrénées Espagnoles, .
1	5	3	13	2	2	3	2	4	41	8	7	7	3	4	6	2012Ferreira#124	B. melitensis bv3	42	116	LNIV-914Bm3-01	unknown	2001	Beira Litoral, Portugal
1	5	3	13	2	2	3	2	4	41	8	7	7	3	4	6	2018Vergnaud#0986	B. melitensis bv3	42	116	BCCN#99-93	Human	1999	Pampelune, Spain
1	5	3	13	2	2	3	2	4	41	8	6	7	3	4	6	2012Ferreira#019	B. melitensis bv3	42	116	LNIV-563Bm3-06	unknown	2006	Beira Litoral, Portugal
1	5	3	13	2	2	3	2	4	41	8	6	7	3	4	6	2012Ferreira#085	B. melitensis bv3	42	116	LNIV-173Bm3-03	unknown	2003	Ribatejo e Oeste, Portu.
1	5	3	13	2	2	3	2	4	41	8	6	7	3	4	6	2012Ferreira#087	B. melitensis bv3	42	116	LNIV-381Bm3-05	unknown	2005	Beira Litoral, Portugal
1	5	3	13	2	2	3	2	4	41	8	6	7	3	4	6	2018Vergnaud#0626	B. melitensis bv3	42	116	BCCN#93-98	Cattle	1993	Pyrénées Espagnoles, .
1	5	3	13	2	2	3	2	4	41	8	6	7	3	4	6	2018Vergnaud#0858	B. melitensis bv3	42	116	BCCN#99-87	Human	1999	Navarre, Spain
1	5	3	13	2	2	3	2	4	41	8	6	7	3	4	6	2018Vergnaud#1013	B. melitensis bv3	42	116	BCCN#95-41	Human	1995	Briare ,Loiret, France
1	5	3	13	2	2	3	2	4	41	8	6	7	3	4	6	2018Vergnaud#1014	B. melitensis bv3	42	116	BCCN#95-42	Human	1995	Briare ,Loiret, France

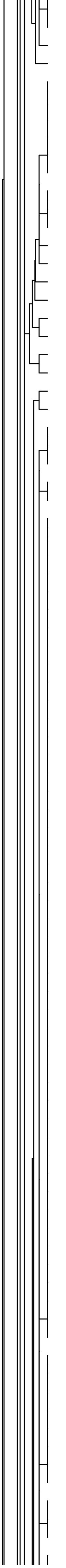

1	5	3	13	2	2	3	2	4	41	8	6	7	3	4	6	2018Vergnaud#1015	B. melitensis bv3	42	116		BCCN#95-43	Human	1995	Briare ,Loiret, France
1	5	3	13	2	2	3	2	4	41	8	6	7	3	4	6	2018Vergnaud#1172	B. melitensis bv3	42	116		BCCN#91-37	Ovine	1991	Saragosse, Spain
1	5	3	13	2	2	3	2	4	41	8	4	7	3	4	6	2006LeFleche#095	B. melitensis bv3	42	116		BCCN#92-80	Ovine	1992	Basque country, Spain
1	5	3	13	2	2	3	2	4	41	8	6	6	3	4	6	2018Vergnaud#1128	B. melitensis bv3	42	116		BCCN#75-283	Human	1975	Castille, Spain
1	5	3	13	2	2	3	2	4	41	8	7	6	3	4	5	2012Ferreira#006	B. melitensis bv3	42	116		LNIV-66Bm3-01	unknown	2001	Entre Douro e Minho, P.
1	5	3	13	2	2	3	2	4	41	8	7	6	3	4	5	2012Ferreira#021	B. melitensis bv3	42	116		AFSSA-02/5258	unknown	2002	Portugal
1	5	3	13	2	2	3	2	4	41	8	7	6	3	4	5	2012Ferreira#099	B. melitensis bv3	42	116		LNIV-581Bm3-06	unknown	2006	Alentejo, Portugal
1	5	3	13	2	2	3	2	4	41	8	7	6	3	4	5	2018Vergnaud#0119	B. melitensis bv3	42	116		BCCN#75-188	Human	1975	Castille, Spain
1	5	3	13	2	2	3	2	4	41	8	7	6	3	4	5	2018Vergnaud#0615	B. melitensis bv3	42	116		BCCN#93-17	Human	1993	Vienne, France
1	5	3	13	2	2	3	2	4	41	8	7	6	3	4	5	2018Vergnaud#1039	B. melitensis bv3	42	116		BCCN#00-3	Human	2000	Rhone, France
1	5	3	13	2	2	3	2	4	41	8	7	6	3	5	5	2007AlDahouk#027	B. melitensis bv2	42	116		BfR 2	Human	2002	Turkey
1	5	3	13	2	2	3	2	4	41	8	7	6	3	5	5	2011Kilic#064	B. melitensis bv3	42	116		BRU-S064	Human	2004	Kirikkale, Turkey
1	5	3	13	2	2	3	2	4	41	8	7	6	3	5	5	2018Vergnaud#0392	B. melitensis bv3	42	116		BCCN#83-35	Human	1983	Ankara ,Turkey
1	5	3	13	2	2	3	2	4	41	8	7	6	3	6	5	2011Kilic#067	B. melitensis bv3	42	116		BRU-S067	Human	2002	Kirikkale, Turkey
1	5	3	13	2	2	3	2	4	41	8	7	6	3	7	5	2018Vergnaud#0815	B. melitensis bv3	42	116		BCCN#75-182	Human	1975	Castille, Spain
1	5	3	14	2	2	3	2	4	41	8	7	6	3	4	5	2018Vergnaud#1399	B. melitensis bv3	83	298		BCCN#97-99	Human	1997	Navarre, Spain
1	5	3	13	2	2	3	2	4	41	8	7	6	6	4	5	2018Vergnaud#1295	B. melitensis bv3	42	116		BCCN#75-162	Human	1975	Castille, Spain
1	5	3	13	2	2	3	2	4	41	8	8	6	3	4	5	2018Vergnaud#1118	B. melitensis bv3	42	116		BCCN#75-151	Human	1975	Castille, Spain
1	5	3	13	2	2	3	2	4	41	8	8	6	3	6	5	2018Vergnaud#1259	B. melitensis bv3	42	116		BCCN#75-298	Human	1975	Castille, Spain
1	5	3	13	2	2	3	2	4	43	8	7	6	3	4	5	2007AlDahouk#071	B. melitensis bv3	42	108		BfR 38	Human	1996	Berlin, Germany
1	5	3	13	2	2	3	2	4	43	8	7	7	3	4	5	2007AlDahouk#072	B. melitensis bv2	42	108		BfR 39	Human	2002	Portugal
1	5	3	13	2	2	3	2	4	41	8	5	8	3	4	5	2018Vergnaud#0059	B. melitensis bv2	42	116		BCCN#74-259	Human	1974	Paris, France
1	5	3	13	2	2	3	2	4	41	8	5	8	3	6	5	2018Vergnaud#1294	B. melitensis bv3	42	116		BCCN#75-161	Human	1975	Castille, Spain
1	5	3	13	2	2	3	2	4	41	8	5	4	3	3	5	2017Liu#052	B. melitensis	42	116		WS051	Human	2015	Fengzhen, China
1	5	3	13	2	2	3	2	4	41	8	5	4	3	3	5	2017Liu#065	B. melitensis	42	116		WS064	Human	2015	Zhongqi, China
1	5	3	13	2	2	3	2	4	41	8	5	4	3	3	5	2017Liu#097	B. melitensis	42	116		WS096	Human	2015	Zhuozi, China
1	5	3	13	2	2	3	2	4	41	8	5	4	3	10	5	2017Liu#008	B. melitensis	42	116		WS007	Human	2014	Liangcheng, China
1	5	3	13	2	2	3	2	4	41	8	5	4	3	10	5	SRR4436595	B. melitensis	42	116	8	BwIM_KWT_42	Human	2017	Kuwait
1	5	3	13	2	2	3	2	4	41	8	5	4	3	6	5	2013Jiang#005	B. melitensis bv3	42	116		SHANXI79-04	Ovine	1979	Shanxi, China
1	5	3	13	2	2	3	2	4	41	8	5	4	3	6	5	2013Jiang#006	B. melitensis bv3	42	116		NM06-1	Ovine	2006	Inner Mongolia, China
1	5	3	13	2	2	3	2	4	41	8	5	4	3	6	5	2013Jiang#016	B. melitensis bv3	42	116		SHD01-8	Ovine	2001	Shandong, China
1	5	3	13	2	2	3	2	4	41	8	5	4	3	6	5	2013Jiang#017	B. melitensis bv3	42	116		SC63-13	Ovine	1963	Sichuan, China
1	5	3	13	2	2	3	2	4	41	8	5	4	3	6	5	2013Jiang#018	B. melitensis bv3	42	116		NM65-38	Ovine	1965	Inner Mongolia, China
1	5	3	13	2	2	3	2	4	41	8	5	4	3	6	5	2013Jiang#023	B. melitensis bv3	42	116		SHD01-1	Ovine	2001	Shandong, China
1	5	3	13	2	2	3	2	4	41	8	5	4	3	6	5	2013Jiang#025	B. melitensis bv1	42	116		XJ05-29	Ovine	2005	Xinjiang, China
1	5	3	13	2	2	3	2	4	41	8	5	4	3	6	5	2015Shevtsov#127	B. melitensis	42	116		Zh-158	Cattle	2008	Jambyl,Zhambyl,Kazak.
1	5	3	13	2	2	3	2	4	41	8	5	4	3	6	5	2015Shevtsov#186	B. melitensis	42	116		5is	Ovine	2013	Kasyk, Korday,Zhambyl.
1	5	3	13	2	2	3	2	4	41	8	5	4	3	6	5	2015Shevtsov#187	B. melitensis	42	116		6is	Ovine	2013	Kasyk, Korday,Zhambyl.
1	5	3	13	2	2	3	2	4	41	8	5	4	3	6	5	2015Shevtsov#188	B. melitensis	42	116		strain ZHB-154	Ovine	2008	Zhanaturminskiy,Bayza.
1	5	3	13	2	2	3	2	4	41	8	5	4	3	6	5	2015Shevtsov#189	B. melitensis	42	116		7is	Ovine	2013	Kasyk, Korday,Zhambyl.
1	5	3	13	2	2	3	2	4	41	8	5	4	3	6	5	2015Shevtsov#192	B. melitensis	42	116		11is	Ovine	2013	Karasay,Korday,Zhamby.
1	5	3	13	2	2	3	2	4	41	8	5	4	3	6	5	2015Shevtsov#194	B. melitensis	42	116		13is	Ovine	2013	Karasay,Korday,Zhamby.
1	5	3	13	2	2	3	2	4	41	8	5	4	3	6	5	2015Shevtsov#195	B. melitensis	42	116		14is	Ovine	2013	Karasay,Korday,Zhamby.
1	5	3	13	2	2	3	2	4	41	8	5	4	3	6	5	2015Shevtsov#196	B. melitensis	42	116		15is	Ovine	2013	Karasay,Korday,Zhamby.
1	5	3	13	2	2	3	2	4	41	8	5	4	3	6	5	2015Shevtsov#198	B. melitensis	42	116		17is	Ovine	2013	Karasay,Korday,Zhamby.
1	5	3	13	2	2	3	2	4	41	8	5	4	3	6	5	2015Shevtsov#200	B. melitensis	42	116		18is	Ovine	2013	Karasay,Korday,Zhamby.
1	5	3	13	2	2	3	2	4	41	8	5	4	3	6	5	2015Shevtsov#202	B. melitensis	42	116		20is	Ovine	2013	Karasay,Korday,Zhamby.
1	5	3	13	2	2	3	2	4	41	8	5	4	3	6	5	2015Shevtsov#203	B. melitensis	42	116		21is	Ovine	2013	Karasay,Korday,Zhamby.
1	5	3	13	2	2	3	2	4	41	8	5	4	3	6	5	2015Shevtsov#204	B. melitensis	42	116		22is	Ovine	2013	Karasay,Korday,Zhamby.
1	5	3	13	2	2	3	2	4	41	8	5	4	3	6	5	2015Shevtsov#205	B. melitensis	42	116		23is	Ovine	2013	Karasay,Korday,Zhamby.
1	5	3	13	2	2	3	2	4	41	8	5	4	3	6	5	2015Shevtsov#207	B. melitensis	42	116		26is	Ovine	2013	Karasay,Korday,Zhamby.
1	5	3	13	2	2	3	2	4	41	8	5	4	3	6	5	2015Shevtsov#208	B. melitensis	42	116		27is	Ovine	2013	Karasay,Korday,Zhamby.
1	5	3	13	2	2	3	2	4	41	8	5	4	3	6	5	2015Shevtsov#209	B. melitensis	42	116		28is	Ovine	2013	Karasay,Korday,Zhamby.
1	5	3	13	2	2	3	2	4	41	8	5	4	3	6	5	2015Shevtsov#212	B. melitensis	42	116		30is	Ovine	2013	Karasay,Korday,Zhamby.
1	5	3	13	2	2	3	2	4	41	8	5	4	3	6	5	2015Shevtsov#213	B. melitensis	42	116		31is	Ovine	2013	Korday,Korday,Zhamby.
1	5	3	13	2	2	3	2	4	41	8	5	4	3	6	5	2015Shevtsov#215	B. melitensis	42	116		34is	Ovine	2013	Korday,Korday,Zhamby.
1	5	3	13	2	2	3	2	4	41	8	5	4	3	6	5	2015Shevtsov#216	B. melitensis	42	116		35is	Ovine	2013	Otar,Korday,Zhambyl,K.
1	5	3	13	2	2	3	2	4	41	8	5	4	3	6	5	2015Shevtsov#217	B. melitensis	42	116		36is	Ovine	2013	Otar,Korday,Zhambyl,K.
1	5	3	13	2	2	3	2	4	41	8	5	4	3	6	5	2015Shevtsov#218	B. melitensis	42	116		38is	Ovine	2013	Nogaybay,Korday,Zha.
1	5	3	13	2	2	3	2	4	41	8	5	4	3	6	5	2015Shevtsov#224	B. melitensis	42	116		47is	Ovine	2013	Akkum,Talas,Zhambyl,.
1	5	3	13	2	2	3	2	4	41	8	5	4	3	6	5	2015Shevtsov#226	B. melitensis	42	116		53is	Ovine	2013	Bostandyk,Talas,Zhamb.
1	5	3	13	2	2	3	2	4	41	8	5	4	3	6	5	2015Shevtsov#227	B. melitensis	42	116		54is	Ovine	2013	Bostandyk,Talas,Zhamb.
1	5	3	13	2	2	3	2	4	41	8	5	4	3	6	5	2015Shevtsov#235	B. melitensis	42	116		71is	Ovine	2013	Navasel,Turar Ryskulov.
1	5	3	13	2	2	3	2	4	41	8	5	4	3	6	5	2015Shevtsov#236	B. melitensis	42	116		72is	Ovine	2013	Navasel,Turar Ryskulov.
1	5	3	13	2	2	3	2	4	41	8	5	4	3	6	5	2015Shevtsov#239	B. melitensis	42	116		75is	Ovine	2013	Kumarlyk,Turar Ryskulo.
1	5	3	13	2	2	3	2	4	41	8	5	4	3	6	5	2015Shevtsov#240	B. melitensis	42	116		76is	Ovine	2013	Kumarlyk,Turar Ryskulo.
1	5	3	13	2	2	3	2	4	41	8	5	4	3	6	5	2015Shevtsov#241	B. melitensis	42	116		77is	Ovine	2013	Kumarlyk,Turar Ryskulo.
1	5	3	13	2	2	3	2	4	41	8	5	4	3	6	5	2015Shevtsov#242	B. melitensis	42	116		78is	Ovine	2013	Kumarlyk,Turar Ryskulo.
1	5	3	13	2	2	3	2	4	41	8	5	4	3	6	5	2015Shevtsov#243	B. melitensis	42	116		Zh-134	Ovine	2008	Karasazskiy,Zhualy,Zh.
1	5	3	13	2	2	3	2	4	41	8	5	4	3	6	5	2015Shevtsov#244	B. melitensis	42	116		79is	Ovine	2013	Kaynar,Raiymbek,Almat.
1	5	3	13	2	2	3	2	4	41	8	5	4	3	6	5	2015Shevtsov#245	B. melitensis	42	116		80is	Ovine	2013	Kaynar,Raiymbek,Almat.
1	5	3	13	2	2	3	2	4	41	8	5	4	3	6	5	2015Shevtsov#247	B. melitensis	42	116		82is	Ovine	2013	Kaynar,Raiymbek,Almat.
1	5	3	13	2	2	3	2	4	41	8	5	4	3	6	5	2015Shevtsov#249	B. melitensis	42	116		85is	Ovine	2013	Kaynar,Raiymbek,Almat.
1	5	3	13	2	2	3	2	4	41	8	5	4	3	6	5	SRR4436640	B. melitensis	42	116	8	BwIM_TUR_51	Human	2017	Turkey
1	5	3	13	2	2	3	2	4	41	8	5	4	3	5	5	2011Jiang#061	B. melitensis bv3	42	116		LN05-4	Human	2005	Liaoning, China
1	5	3	13	2	2	3	2	4	41	8	5	4	3	5	5	2011Jiang#095	B. melitensis bv1	42	116		NM96-1006	Human	1996	Inner Mongolia, China
1	5	3	13	2	2	3	2	4	41	8	5	4	3	5	5	2013Jiang#064	B. melitensis bv1	42	116		XJ152	Ovine	2011	Xinjiang, China
1	5	3	13	2	2	3	2	4	41	8	5	4	3	5	5	2015Shevtsov#162	B. melitensis bv3	42	116		011210	Dog	2009	Almaty, Almaty, Almaty,.
1	5	3	13	2	2	3	2	4	41	8	5	4	3	5	5	2015Shevtsov#165	B. melitensis bv1	42	116		25Zh	Ovine	2009	Zhualy,Zhambyl,Kazakh.
1	5	3	13	2	2	3	2	4	41	8	5	4	3	5	5	2015Shevtsov#237	B. melitensis	42	116		73is	Ovine	2013	Navasel,Turar Ryskulov.
1	5	3	13	2	2	3	2	4	41	8	5	4	3	5	5	2018Vergnaud#0278	B. melitensis bv1	42	116		BCCN#02-12	Human	2002	Mongolia
1	5	3	13	2	2	3	2	4	41	8	5	4	3	5	5	2018Vergnaud#0429	B. melitensis bv1	42	116		BCCN#02-14	Human	2002	Mongolia
1	5	3	13	2	2	3	2	4	41	8	5	4	3	9	5	2011Jiang#044	B. melitensis bv3	42	116		SHANXI09-13	Human	2009	Shanxi, China
1	5	3	13	2	2	3	2	4	41	8	5	4	3	9	5	2011Jiang#069	B. melitensis bv2	42	116		QH65-063	Human	1965	Qinghai, China
1	5	3	13	2	2	3	2	4	41	8	5	4	3	9	5	2015Shevtsov#172	B. melitensis bv1	42	116		316Zh	Ovine	2011	Zhualy,Zhambyl,Kazakh.
1	5	3	13	2	2	3	2	4	41	8	5	4	3	7	5	2011Jiang#021	B. melitensis bv3	42	116		GD07-3	Human	2007	Guangdong, China

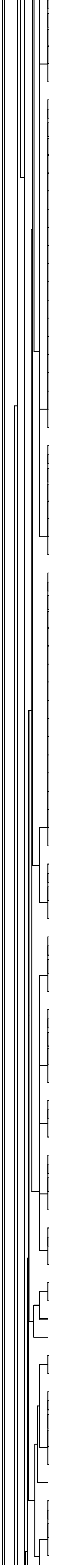

1	5	3	13	2	2	3	2	4	41	8	5	4	3	7	5	2011Jiang#023	B. melitensis bv3	42	116	GD07-5	Human	2007	Guangdong, China
1	5	3	13	2	2	3	2	4	41	8	5	4	3	7	5	2011Jiang#026	B. melitensis bv3	42	116	GD07-8	Human	2007	Guangdong, China
1	5	3	13	2	2	3	2	4	41	8	5	4	3	7	5	2011Jiang#037	B. melitensis bv3	42	116	GD09-1	Human	2009	Guangdong, China
1	5	3	13	2	2	3	2	4	41	8	5	4	3	7	5	2011Jiang#041	B. melitensis bv3	42	116	GD09-5	Human	2009	Guangdong, China
1	5	3	13	2	2	3	2	4	41	8	5	4	3	7	5	2017Liu#071	B. melitensis	42	116	WS070	Human	2015	Zhongqi, China
1	5	3	13	2	2	3	2	4	41	8	5	4	3	4	5	2011Jiang#013	B. melitensis bv3	42	116	GD08-5	Human	2008	Guangdong, China
1	5	3	13	2	2	3	2	4	41	8	5	4	3	4	5	2011Jiang#017	B. melitensis bv3	42	116	GD08-9	Human	2008	Guangdong, China
1	5	3	13	2	2	3	2	4	41	8	5	4	3	4	5	2015Shevtsov#161	B. melitensis bv3	42	116	011208	Dog	2009	Almaty, Almaty, Almaty,.
1	5	3	13	2	2	3	2	4	41	8	5	4	3	4	5	2015Shevtsov#193	B. melitensis	42	116	12is	Ovine	2013	Karasay,Korday,Zhamby.
1	5	3	13	2	2	3	2	4	41	8	5	4	3	4	5	2015Shevtsov#197	B. melitensis	42	116	16is	Ovine	2013	Karasay,Korday,Zhamby.
1	5	3	13	2	2	3	2	4	41	8	5	4	3	4	5	2015Shevtsov#201	B. melitensis	42	116	19is	Ovine	2013	Karasay,Korday,Zhamby.
1	5	3	13	2	2	3	2	4	41	8	5	4	3	4	5	2015Shevtsov#206	B. melitensis	42	116	24is	Ovine	2013	Karasay,Korday,Zhamby.
1	5	3	13	2	2	3	2	4	41	8	5	4	3	4	5	2015Shevtsov#211	B. melitensis	42	116	29is	Ovine	2013	Karasay,Korday,Zhamby.
1	5	3	13	2	2	3	2	4	41	8	5	4	3	4	5	2015Shevtsov#214	B. melitensis	42	116	32is	Ovine	2013	Korday,Korday,Zhamby.
1	5	3	13	2	2	3	2	4	41	8	5	4	3	4	5	2015Shevtsov#223	B. melitensis	42	116	45is	Ovine	2013	Akkum,Talas,Zhambyl,.
1	5	3	13	2	2	3	2	4	41	8	5	4	3	4	5	2015Shevtsov#225	B. melitensis	42	116	49is	Ovine	2013	Akkum,Talas,Zhambyl,.
1	5	3	13	2	2	3	2	4	41	8	5	4	3	4	5	2015Shevtsov#228	B. melitensis	42	116	61is	Ovine	2013	Zhambyl,Moiynkum,Zh.
1	5	3	13	2	2	3	2	4	41	8	5	4	3	4	5	2015Shevtsov#229	B. melitensis	42	116	65is	Ovine	2013	Kenes,Moiynkum,Zha.
1	5	3	13	2	2	3	2	4	41	8	5	4	3	4	5	2015Shevtsov#230	B. melitensis	42	116	66is	Ovine	2013	Kenes,Moiynkum,Zha.
1	5	3	13	2	2	3	2	4	41	8	5	4	3	4	5	2015Shevtsov#231	B. melitensis	42	116	67is	Ovine	2013	Kenes,Moiynkum,Zha.
1	5	3	13	2	2	3	2	4	41	8	5	4	3	4	5	2015Shevtsov#233	B. melitensis	42	116	69is	Ovine	2013	Birlik,Moiynkum,Zhamby.
1	5	3	13	2	2	3	2	4	41	8	5	4	3	4	5	2015Shevtsov#234	B. melitensis	42	116	70is	Ovine	2013	Navasel,Turar Ryskulov.
1	5	3	13	2	2	3	2	4	41	8	5	4	3	4	5	2015Shevtsov#238	B. melitensis	42	116	74is	Ovine	2013	Kumarlyk,Turar Ryskulo.
1	5	3	13	2	2	3	2	4	41	8	5	4	3	4	5	2015Shevtsov#253	B. melitensis	42	116	89is	Ovine	2013	Bakanas, Balkhash,Alm.
1	5	3	13	2	2	3	2	4	41	8	5	4	3	8	5	2011Jiang#001	B. melitensis bv3	42	116	SHX08-3	Human	2008	Shan\'xi, China
1	5	3	13	2	2	3	2	4	41	8	5	4	3	8	5	2011Jiang#027	B. melitensis bv3	42	116	GD07-9	Human	2007	Guangdong, China
1	5	3	13	2	2	3	2	4	41	8	5	4	3	8	5	2011Jiang#028	B. melitensis bv3	42	116	NM57-9	Human	1957	Inner Mongolia, China
1	5	3	13	2	2	3	2	4	41	8	5	4	3	8	5	2011Jiang#036	B. melitensis bv3	42	116	HN82-13	Human	1982	Henan, China
1	5	3	13	2	2	3	2	4	41	8	5	4	3	8	5	2013Jiang#042	B. melitensis bv1	42	116	NM96-1005	Ovine	1996	Inner Mongolia, China
1	5	3	13	2	2	3	2	4	41	8	5	4	3	8	5	2015Shevtsov#163	B. melitensis bv1	42	116	20Zh	Ovine	2009	Bayzak,Zhambyl,Kazak.
1	5	3	13	2	2	3	2	4	41	8	5	4	3	8	5	2015Shevtsov#164	B. melitensis bv1	42	116	21Zh	Ovine	2009	Bayzak,Zhambyl,Kazak.
1	5	3	13	2	2	3	2	4	41	8	5	9	3	8	5	2012Ferreira#008	B. melitensis bv3	42	116	LNIV-479Bm3-05	unknown	2005	Beira Litoral, Portugal
1	5	3	13	2	2	3	2	4	41	8	5	9	3	8	5	2012Ferreira#009	B. melitensis bv3	42	116	LNIV-480Bm3-05	unknown	2005	Beira Litoral, Portugal
1	5	3	13	2	2	3	2	4	41	8	5	9	3	8	5	2012Ferreira#010	B. melitensis bv3	42	116	LNIV-481Bm3-05	unknown	2005	Beira Litoral, Portugal
1	5	3	13	2	2	3	2	4	41	8	5	9	3	8	5	2012Ferreira#011	B. melitensis bv3	42	116	LNIV-482Bm3-05	unknown	2005	Beira Litoral, Portugal
1	5	3	13	2	2	3	2	4	41	8	5	9	3	8	5	2012Ferreira#012	B. melitensis bv3	42	116	LNIV-483Bm3-05	unknown	2005	Beira Litoral, Portugal
1	5	3	13	2	2	3	2	4	41	8	5	9	3	8	5	2012Ferreira#013	B. melitensis bv3	42	116	LNIV-484Bm3-05	unknown	2005	Beira Litoral, Portugal
1	5	3	13	2	2	3	2	4	41	8	5	9	3	8	5	2012Ferreira#014	B. melitensis bv3	42	116	LNIV-485Bm3-05	unknown	2005	Beira Litoral, Portugal
1	5	3	13	2	2	3	2	4	41	8	5	9	3	8	5	2012Ferreira#015	B. melitensis bv3	42	116	LNIV-486Bm3-05	unknown	2005	Beira Litoral, Portugal
1	5	3	13	2	2	3	2	4	41	8	5	9	3	8	5	2012Ferreira#016	B. melitensis bv3	42	116	LNIV-490Bm3-05	unknown	2005	Beira Litoral, Portugal
1	5	3	13	2	2	3	2	4	41	8	5	9	3	8	5	2012Ferreira#017	B. melitensis bv3	42	116	LNIV-505Bm3-05	unknown	2005	Beira Litoral, Portugal
1	5	3	13	2	2	3	2	4	41	8	5	9	3	8	5	2012Ferreira#031	B. melitensis bv3	42	116	AFSSA-05/3565	unknown	2005	Portugal
1	5	3	13	2	2	3	2	4	41	8	5	9	3	8	5	2012Ferreira#032	B. melitensis bv3	42	116	AFSSA-05/4465	unknown	2005	Portugal
1	5	3	13	2	2	3	2	4	41	8	5	9	3	8	5	2012Ferreira#033	B. melitensis bv3	42	116	AFSSA-05/3111	unknown	2005	Portugal
1	5	3	13	2	2	3	2	4	41	8	5	9	3	8	5	2012Ferreira#034	B. melitensis bv3	42	116	AFSSA-05/3592	unknown	2005	Portugal
1	5	3	13	2	2	3	2	4	41	8	5	9	3	8	5	2012Ferreira#035	B. melitensis bv3	42	116	AFSSA-05/3731	unknown	2005	Portugal
1	5	3	13	2	2	3	2	4	41	8	5	9	3	8	5	2012Ferreira#036	B. melitensis bv3	42	116	AFSSA-05/3819	unknown	2005	Portugal
1	5	3	13	2	2	3	2	4	41	8	5	9	3	6	5	2007AlDahouk#030	B. melitensis bv2	42	116	BfR 5	Human	2002	Turkey
1	5	3	13	2	2	3	2	4	41	8	5	9	3	6	5	2012Ferreira#029	B. melitensis bv3	42	116	AFSSA-05/3818	unknown	2005	Portugal
1	5	3	13	2	2	3	2	4	41	8	5	9	3	6	5	2012Ferreira#038	B. melitensis bv3	42	116	AFSSA-06/1576	unknown	2006	Portugal
1	5	3	13	2	2	3	2	4	41	8	5	9	3	6	5	2012Ferreira#039	B. melitensis bv3	42	116	LNIV-630Bm3-06	unknown	2006	Beira Litoral, Portugal
1	5	3	13	2	2	3	2	4	41	8	5	6	3	9	5	2018Vergnaud#0840	B. melitensis bv3	42	116	BCCN#91-63	Caprine	1991	Saragosse, Spain
1	5	3	13	2	2	3	2	4	41	8	5	6	3	9	5	2018Vergnaud#0841	B. melitensis bv3	42	116	BCCN#91-64	Caprine	1991	Saragosse, Spain
1	5	3	13	2	2	3	2	4	41	8	5	6	3	9	5	2018Vergnaud#1174	B. melitensis bv3	42	116	BCCN#91-48	Ovine	1991	Saragosse, Spain
1	5	3	13	2	2	3	2	4	41	8	5	6	3	9	5	2018Vergnaud#1274	B. melitensis bv3	42	116	BCCN#91-42	Ovine	1991	Saragosse, Spain
1	5	3	13	2	2	3	2	4	41	8	5	6	3	4	5	2018Vergnaud#0589	B. melitensis bv3	42	116	BCCN#90-9	Ovine	1990	Saragosse, Spain
1	5	3	13	2	2	3	2	4	41	8	5	6	3	4	5	2018Vergnaud#0816	B. melitensis bv3	42	116	BCCN#75-280	Human	1975	Castille, Spain
1	5	3	13	2	2	3	2	4	41	8	5	6	3	4	5	2018Vergnaud#0925	B. melitensis bv3	42	116	BCCN#75-303	Human	1975	Castille, Spain
1	5	3	13	2	2	3	2	4	41	8	5	6	3	4	5	2018Vergnaud#0960	B. melitensis bv3	42	116	BCCN#02-1	Human	2002	Indre-et-Loire, France
1	5	3	13	2	2	3	2	4	41	8	5	6	3	4	5	2018Vergnaud#1260	B. melitensis bv3	42	116	BCCN#75-299	Human	1975	Castille, Spain
1	5	3	13	2	2	3	2	4	41	8	5	6	3	7	5	2012Ferreira#096	B. melitensis bv3	42	116	LNIV-310Bm3-04	unknown	2004	Alentejo, Portugal
1	5	3	13	2	2	3	2	4	41	8	5	6	3	7	5	2012Ferreira#106	B. melitensis bv3	42	116	LNIV-241Bm3-03	unknown	2003	Algarve, Portugal
1	5	3	13	2	2	3	2	4	41	8	5	6	3	7	5	2012Ferreira#107	B. melitensis bv3	42	116	LNIV-272Bm3-04	unknown	2004	Algarve, Portugal
1	5	3	13	2	2	3	2	4	41	8	5	6	3	3	5	2012Ferreira#049	B. melitensis bv3	42	116	LNIV-511Bm3-05	unknown	2005	Tras-os-Montes, Portugal
1	5	3	13	2	2	3	2	4	41	8	5	6	3	3	5	2012Ferreira#050	B. melitensis bv3	42	116	LNIV-512Bm3-05	unknown	2005	Tras-os-Montes, Portugal
1	5	3	13	2	2	3	2	4	41	8	5	6	3	3	5	2012Ferreira#051	B. melitensis bv3	42	116	LNIV-513Bm3-05	unknown	2005	Tras-os-Montes, Portugal
1	5	3	13	2	2	3	2	4	41	8	5	6	3	3	5	2012Ferreira#052	B. melitensis bv3	42	116	LNIV-514Bm3-06	unknown	2006	Tras-os-Montes, Portugal
1	5	3	13	2	2	3	2	4	41	8	5	6	3	5	5	2011Kilic#063	B. melitensis bv3	42	116	BRU-S063	Human	2004	Kirikkale, Turkey
1	5	3	13	2	2	3	2	4	41	8	5	6	3	5	5	2011Kilic#072	B. melitensis bv3	42	116	BRU-S072	Human	2004	Kirikkale, Turkey
1	5	3	13	2	2	3	2	4	41	8	5	6	3	5	5	2012Ferreira#108	B. melitensis bv3	42	116	LNIV-273Bm3-04	unknown	2004	Algarve, Portugal
1	5	3	13	2	2	3	2	4	41	8	8	4	3	8	5	2011Jiang#085	B. melitensis bv3	42	116	LB10-15	Human	2010	Guangdong, China
1	5	3	13	2	2	3	2	4	41	8	8	4	3	8	5	2015Shevtsov#168	B. melitensis bv1	42	116	44Zh	Ovine	2009	Zhualy,Zhambyl,Kazakh.
1	5	3	13	2	2	3	2	4	41	8	8	4	3	6	5	2017Liu#022	B. melitensis	42	116	WS021	Human	2014	Ulanqab, China
1	5	3	13	2	2	3	2	4	41	9	8	4	3	6	5	2017Liu#003	B. melitensis	42	345	WS002	Human	2012	Qianqi, China
1	5	3	13	2	2	3	2	4	46	8	5	7	3	5	5	2018Vergnaud#0425	B. melitensis bv3	42	297	BCCN#98-18	Human	1998	Nador ,Morocco
1	5	3	13	2	2	3	2	4	46	8	5	7	3	5	5	2018Vergnaud#0669	B. melitensis bv3	42	297	BCCN#98-6	Human	1998	Vedene ,Vaucluse, Fra.
1	5	3	13	2	2	3	2	4	46	8	5	4	3	5	5	2011Jiang#063	B. melitensis bv1	42	297	SD05-34	Human	2005	Shandong, China
1	5	3	13	2	2	3	2	4	46	8	5	4	3	5	5	2011Jiang#064	B. melitensis bv1	42	297	SD05-40	Human	2005	Shandong, China
1	5	3	13	2	2	3	2	4	46	8	5	4	3	5	5	2011Jiang#075	B. melitensis bv3	42	297	LB10-03	Human	2010	Guangdong, China
1	5	3	13	2	2	3	2	4	46	8	5	4	3	5	5	2013Jiang#007	B. melitensis bv3	42	297	NM06-2	Ovine	2006	Inner Mongolia, China
1	5	3	13	2	2	3	2	4	46	8	5	4	3	5	5	2013Jiang#008	B. melitensis bv3	42	297	NM06-3	Ovine	2006	Inner Mongolia, China
1	5	3	13	2	2	3	2	4	46	8	5	4	3	6	5	2013Jiang#009	B. melitensis bv3	42	297	NM06-4	Ovine	2006	Inner Mongolia, China
1	5	3	13	2	2	3	2	4	45	8	5	4	3	5	5	2011Jiang#102	B. melitensis bv1	42	179	NM91-0513	Human	1991	Inner Mongolia, China
1	5	3	13	2	2	3	2	4	45	8	5	4	3	5	5	2011Jiang#103	B. melitensis bv1	42	179	NM91-0514	Human	1991	Inner Mongolia, China
1	5	3	13	2	2	3	2	4	45	8	5	4	3	5	5	2013Jiang#049	B. melitensis bv1	42	179	NM91-0511	Ovine	1991	Inner Mongolia, China
1	5	3	13	2	2	3	2	4	45	8	5	4	3	5	5	2013Jiang#057	B. melitensis bv1	42	179	NM92-0617	Ovine	1992	Inner Mongolia, China

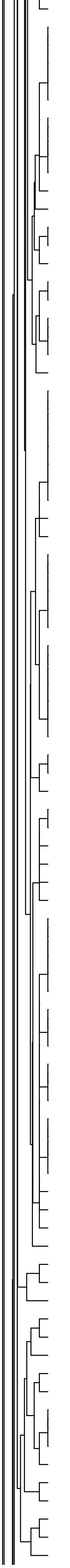

1	5	3	13	2	2	3	2	4	45	8	5	4	3	9	5	2013Jiang#044	B. melitensis bv1	42	179		NM89-0906	Ovine	1989	Inner Mongolia, China
1	5	3	13	2	2	3	2	4	41	8	4	4	3	9	5	2011Jiang#101	B. melitensis bv1	42	116		NM88-0906	Human	1988	Inner Mongolia, China
1	5	3	13	2	2	3	2	4	41	8	4	4	3	9	5	2013Jiang#046	B. melitensis bv1	42	116		NM91-0508	Cattle	1991	Inner Mongolia, China
1	5	3	13	2	2	3	2	4	41	8	4	4	3	9	5	2013Jiang#050	B. melitensis bv1	42	116		NM91-0512	Ovine	1991	Inner Mongolia, China
1	5	3	13	2	2	3	2	4	41	8	4	4	3	9	5	2013Jiang#051	B. melitensis bv1	42	116		NM92-0611	Ovine	1992	Inner Mongolia, China
1	5	3	13	2	2	3	2	4	41	8	4	4	3	9	5	2013Jiang#052	B. melitensis bv1	42	116		NM92-0612	Ovine	1992	Inner Mongolia, China
1	5	3	13	2	2	3	2	4	41	8	7	4	3	9	5	2011Jiang#079	B. melitensis bv3	42	116		LB10-08	Human	2010	Guangdong, China
1	5	3	13	2	2	3	2	4	41	8	7	4	3	9	5	2011Jiang#080	B. melitensis bv3	42	116		LB10-09	Human	2010	Guangdong, China
1	5	3	13	2	2	3	2	4	41	8	7	4	3	9	5	2011Jiang#081	B. melitensis bv3	42	116		LB10-10	Human	2010	Guangdong, China
1	5	3	13	2	2	3	2	4	41	8	7	4	3	9	5	2011Jiang#082	B. melitensis bv3	42	116		LB10-11	Human	2010	Guangdong, China
1	5	3	13	2	2	3	2	4	41	8	6	4	3	9	5	2017Liu#002	B. melitensis	42	116		WS001	Human	2012	Huade, China
1	5	3	13	2	2	3	2	4	41	8	4	7	3	9	5	2018Vergnaud#0694	B. melitensis bv3	42	116		BCCN#99-85	Human	1999	Navarre, Spain
1	5	3	13	2	2	3	2	4	41	8	4	4	3	3	5	2013Jiang#045	B. melitensis bv1	42	116		NM89-0907	Ovine	1989	Inner Mongolia, China
1	5	3	13	2	2	3	2	4	41	8	4	4	3	3	5	2018Vergnaud#0368	B. melitensis R.	42	116		BCCN#02-40	Human	2002	Mongolia
1	5	3	13	2	2	3	2	4	41	8	7	4	3	3	5	2017Liu#104	B. melitensis	42	116		WS103	Human	2015	Xinghe, China
1	5	3	13	2	2	3	2	4	9	8	4	4	3	5	5	2018Vergnaud#0086	B. melitensis bv1	42	295		BCCN#77-3	Human	1977	Pampelune, Spain
1	5	3	13	2	2	3	2	4	9	8	4	4	3	5	5	2018Vergnaud#0803	B. melitensis bv1	42	295		BCCN#76-423	Human	1976	Navarre, Spain
1	5	3	13	2	2	3	2	4	41	8	4	4	3	5	5	2013Jiang#032	B. melitensis bv2	42	116		NM66-148	Camel		Inner Mongolia, China
1	5	3	13	2	2	3	2	4	41	8	4	4	3	5	5	2013Jiang#058	B. melitensis bv1	42	116		NM93-0907	Ovine	1993	Inner Mongolia, China
1	5	3	13	2	2	3	2	4	41	8	4	4	3	5	5	SRR4436599	B. melitensis	42	116	8	BwIM_IND_58	Human	2017	India
1	5	3	13	2	2	3	2	4	41	8	7	4	3	5	5	GCA_000192725	B. melitensis	42	116	8	M28	Ovine	2011	China
1	5	3	13	2	2	3	2	4	41	8	6	4	3	7	5	2011Jiang#074	B. melitensis bv1	42	116		LB10-01	Human	2010	Guangdong, China
1	5	3	13	2	2	3	2	4	41	8	6	4	3	7	5	2011Jiang#105	B. melitensis bv1	42	116		M5	Ovine	1962	Heilongjiang, China
1	5	3	13	2	2	3	2	4	41	8	6	4	3	7	5	2013Jiang#059	B. melitensis bv1	42	116		M5	Ovine	1962	Heilongjiang, China
1	5	3	13	2	2	3	2	4	41	8	6	4	3	7	5	2017Liu#170	B. melitensis bv3	42	116		326-4		2015	
1	5	3	13	2	2	3	2	4	41	8	6	4	3	7	5	2017Liu#171	B. melitensis bv3	42	116		326-5		2015	
1	5	3	13	2	2	3	2	4	41	8	6	4	3	7	5	2017Liu#172	B. melitensis bv3	42	116		326-7		2015	
1	5	3	13	2	2	3	2	4	41	8	6	4	3	7	5	2017Liu#174	B. melitensis bv3	42	116		326-9		2015	
1	5	3	13	2	2	3	2	4	41	8	6	4	3	11	5	2017Liu#010	B. melitensis	42	116		WS009	Human	2014	Houqi, China
1	5	3	13	2	2	3	2	4	41	8	6	4	3	8	5	2011Jiang#073	B. melitensis bv3	42	116		LB10-02	Human	2010	Guangdong, China
1	5	3	13	2	2	3	2	4	46	8	6	4	3	5	5	2017Liu#061	B. melitensis	42	297		WS060	Human	2015	Zhongqi, China
1	5	3	13	2	2	3	2	4	46	8	6	4	3	5	5	2017Liu#062	B. melitensis	42	297		WS061	Human	2015	Ulanqab, China
1	5	3	13	2	2	3	2	4	46	8	6	4	3	5	5	2017Liu#066	B. melitensis	42	297		WS065	Human	2015	Zhuozi, China
1	5	3	13	2	2	3	2	4	46	8	6	4	3	5	5	2017Liu#068	B. melitensis	42	297		WS067	Human	2015	Zhongqi, China
1	5	3	13	2	2	3	2	4	46	8	6	4	3	5	5	2017Liu#089	B. melitensis	42	297		WS088	Human	2015	Qianqi, China
1	5	3	13	2	2	3	2	4	41	8	6	4	3	5	5	2011Jiang#100	B. melitensis bv1	42	116		NM97-12	Human	1997	Inner Mongolia, China
1	5	3	13	2	2	3	2	4	41	8	6	4	3	5	5	2013Jiang#055	B. melitensis bv1	42	116		NM92-0615	Cattle	1992	Inner Mongolia, China
1	5	3	13	2	2	3	2	4	41	8	6	4	3	5	5	2013Jiang#081	B. melitensis bv3	42	116		LY007	Human	2011	Zhejiang, China
1	5	3	13	2	2	3	2	4	41	8	6	4	3	5	5	2015Shevtsov#166	B. melitensis	42	116		A 1/3	Ovine	2007	Ashibulakskiy,Ile,Almat.
1	5	3	13	2	2	3	2	4	41	8	6	4	3	5	5	2017Liu#005	B. melitensis	42	116		WS004	Human	2012	Ulanqab, China
1	5	3	13	2	2	3	2	4	41	8	6	4	3	5	5	GCA_000192885	B. melitensis	42	116	8	M5-90	Ovine	2011	China
1	5	3	13	2	2	3	2	4	41	8	6	12	3	5	5	2011Kilic#074	B. melitensis bv3	42	116		BRU-S074	Human	2002	Kirikkale, Turkey
1	5	3	13	2	2	3	2	4	41	8	6	12	3	5	5	2011Kilic#077	B. melitensis bv3	42	116		BRU-S077	Human	2002	Kirikkale, Turkey
1	5	3	13	2	3	3	2	4	41	8	6	12	3	5	5	2011Kilic#068	B. melitensis bv3	63	111		BRU-S068	Human	2002	Kirikkale, Turkey
1	5	3	13	2	2	3	2	4	41	8	6	4	3	6	5	2013Jiang#061	B. melitensis bv3	42	116		XJ130	Ovine	2011	Xinjiang, China
1	5	3	13	2	2	3	2	4	41	8	6	4	3	6	5	2017Liu#072	B. melitensis	42	116		WS071	Human	2015	Huade, China
1	5	3	13	2	2	3	2	4	41	8	6	5	3	6	5	2018Vergnaud#0394	B. melitensis bv3	42	116		BCCN#85-37	Human	1985	Portugal
1	5	3	13	2	2	3	2	4	41	8	6	9	3	6	5	2007AlDahouk#063	B. melitensis bv2	42	116		BfR 29	Human	1999	
1	5	3	13	2	2	3	2	4	41	8	6	8	3	6	5	2012Ferreira#079	B. melitensis bv3	42	116		LNIV-536Bm3-06	unknown	2006	Beira Interior, Portugal
1	5	3	13	2	2	3	2	4	41	8	6	10	3	6	5	2017Hanot-Mamb.	B. melitensis bv3	42	116		L3/138	Human	2004	Belgium
1	5	3	13	2	2	3	2	4	41	8	6	6	3	5	5	2018Vergnaud#1306	B. melitensis bv3	42	116		BCCN#91-226	Ovine	1991	Saragosse ,Spain
1	5	3	13	2	2	3	2	4	41	8	6	6	3	5	5	SRR4436601	B. melitensis	42	116	8	BwIM_TUR_61a	Human		Turkey
1	5	3	13	2	2	3	2	4	41	8	6	6	3	5	5	SRR4436631	B. melitensis	42	116	8	BwIM_TUR_61c	Human		Turkey
1	5	3	13	2	2	3	2	4	41	8	6	6	3	5	5	SRR4436633	B. melitensis	42	116	8	BwIM_TUR_61b	Human		Turkey
1	5	3	13	2	2	3	2	4	41	8	6	6	3	5	5	SRR4436633_SR.	B. melitensis	42	116	8			2017	
1	5	3	13	2	2	3	2	4	41	8	6	6	3	11	5	2018Vergnaud#1199	B. melitensis bv3	42	116		BCCN#90-182	Cattle	1990	Navarre, Spain
1	5	3	13	2	2	3	2	4	41	8	6	6	3	11	5	2018Vergnaud#1270	B. melitensis bv3	42	116		BCCN#90-184	Cattle	1990	Navarre, Spain
1	5	3	13	2	2	3	2	4	41	8	6	6	3	11	5	2018Vergnaud#1271	B. melitensis bv3	42	116		BCCN#90-185	Cattle	1990	Navarre, Spain
1	5	3	13	2	2	3	2	4	41	8	6	6	3	4	5	2012Ferreira#067	B. melitensis bv3	42	116		LNIV-718Bm3-07	unknown	2007	Beira Litoral, Portugal
1	5	3	13	2	2	3	2	4	41	8	6	6	3	4	5	2012Ferreira#120	B. melitensis bv3	42	116		LNIV-768Bm3-07	unknown	2007	Entre Douro e Minho, P.
1	5	3	13	2	2	3	2	4	41	8	6	6	3	4	5	2018Vergnaud#0818	B. melitensis bv3	42	116		BCCN#75-300	Human	1975	Castille, Spain
1	5	3	13	2	2	3	2	4	41	8	6	6	3	6	5	2011Kilic#066	B. melitensis bv3	42	116		BRU-S066	Human	2003	Kirikkale, Turkey
1	5	3	13	2	2	3	2	4	41	8	6	6	3	6	5	2011Kilic#075	B. melitensis bv3	42	116		BRU-S075	Human	2003	Kirikkale, Turkey
1	5	3	13	2	2	3	2	4	41	8	6	6	3	6	5	2011Kilic#078	B. melitensis bv3	42	116		BRU-S078	Human	2003	Kirikkale, Turkey
1	5	3	13	2	2	3	2	4	41	8	6	6	3	6	5	2018Vergnaud#0926	B. melitensis bv3	42	116		BCCN#75-304	Human	1975	Castille, Spain
1	5	3	13	2	2	3	2	4	41	8	6	6	3	8	5	2011Kilic#073	B. melitensis bv3	42	116		BRU-S073	Human	2002	Kirikkale, Turkey
1	5	3	13	2	2	3	2	4	41	8	6	6	3	7	5	2018Vergnaud#1116	B. melitensis bv3	42	116		BCCN#75-134	Human	1975	Castille, Spain
1	5	3	13	2	2	3	2	4	41	8	6	6	3	12	5	2018Vergnaud#1158	B. melitensis bv3	42	116		BCCN#90-181	Cattle	1990	Navarre, Spain
1	5	3	13	2	2	3	2	4	41	8	6	6	5	6	5	2018Vergnaud#1149	B. melitensis bv3	42	116		BCCN#75-145	Human	1975	Castille, Spain
1	5	3	13	2	2	3	2	4	41	8	3	5	3	6	5	2017Liu#017	B. melitensis	42	116		WS016	Human	2014	Shangdu, China
1	5	3	13	2	2	3	2	4	41	8	3	5	3	7	5	2017Liu#129	B. melitensis bv3	42	116		M67		2016	
1	5	3	13	2	3	3	2	4	41	8	2	5	3	7	5	2017Liu#124	B. melitensis bv3	63	111		M68		2016	
1	5	3	13	2	2	3	2	4	41	8	4	4	7	4	4	2011Jiang#057	B. melitensis bv3	42	116		FJ09-5	Human	2009	Fujian, China
1	5	3	13	2	2	3	2	4	41	8	4	4	3	4	4	2017Liu#036	B. melitensis	42	116		WS035	Human	2015	Liangcheng, China
1	5	3	13	2	2	3	2	4	41	8	4	4	6	6	4	2013Jiang#035	B. melitensis bv2	42	116		XJ78-037	Ovine	1978	Xinjiang, China
1	5	3	13	2	2	3	2	4	41	8	7	6	3	6	4	2011Kilic#083	B. melitensis bv3	42	116		BRU-S083	Human	2003	Kirikkale, Turkey
1	5	3	13	2	2	3	2	4	41	8	7	6	3	4	4	2012Ferreira#094	B. melitensis bv3	42	116		LNIV-239Bm3-03	unknown	2003	Alentejo, Portugal
1	5	3	13	2	2	3	2	4	41	8	4	6	3	4	4	2012Ferreira#053	B. melitensis bv3	42	116		LNIV-515Bm3-06	unknown	2006	Tras-os-Montes, Portugal
1	5	3	13	2	2	3	2	4	41	8	4	6	3	4	4	2012Ferreira#054	B. melitensis bv3	42	116		LNIV-516Bm3-06	unknown	2006	Tras-os-Montes, Portugal
1	5	3	13	2	2	3	2	4	41	8	4	6	3	4	4	2012Ferreira#055	B. melitensis bv3	42	116		LNIV-518Bm3-06	unknown	2006	Tras-os-Montes, Portugal
1	5	3	13	2	2	3	2	4	41	8	4	6	3	8	4	2010Valdezate#064	B. melitensis	42	116		#20091052	Human	2009	Avila, Spain
1	5	3	13	2	2	3	2	4	41	8	4	8	3	4	3	2018Vergnaud#0973	B. melitensis bv3	42	116		BCCN#90-14	Ovine	1990	Saragosse, Spain
1	5	3	13	2	2	3	2	4	41	8	4	8	3	4	7	2018Vergnaud#0974	B. melitensis bv3	42	116		BCCN#90-15	Ovine	1990	Saragosse, Spain
1	5	3	13	2	2	3	2	4	41	8	4	4	9	4	5	2011Jiang#054	B. melitensis bv3	42	116		HZ09-2	Human	2009	Zhejiang, China
1	5	3	13	2	2	3	2	4	41	8	4	4	8	4	5	2013Jiang#013	B. melitensis bv3	42	116		XJ07-2	Ovine	2007	Xinjiang, China
1	5	3	13	2	2	3	2	4	41	8	4	5	7	4	5	2018Vergnaud#0402	B. melitensis bv3	42	116		BCCN#91-113	Cattle	1991	Palampur ,India

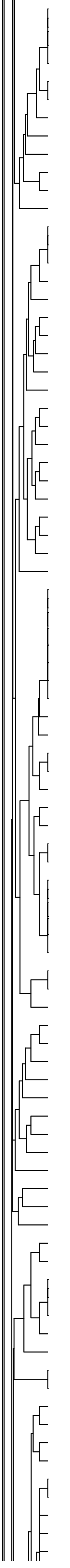

1	5	3	13	2	2	3	2	4	41	8	8	6	3	7	6	2011Kilic#082	B. melitensis bv3	42	116		BRU-S082	Human	2003	Kirikkale, Turkey
1	5	3	13	2	2	3	2	4	41	8	8	6	3	7	6	2018Vergnaud#1273	B. melitensis bv3	42	116		BCCN#91-41	Ovine	1991	Saragosse, Spain
1	5	3	13	2	2	3	2	4	41	8	8	6	3	7	6	2018Vergnaud#1275	B. melitensis bv3	42	116		BCCN#91-43	Ovine	1991	Saragosse, Spain
1	5	3	13	2	2	3	2	4	41	8	8	6	3	7	6	2018Vergnaud#1276	B. melitensis bv3	42	116		BCCN#91-44	Ovine	1991	Saragosse, Spain
1	5	3	13	2	2	3	2	4	41	8	7	6	3	7	6	2011Kilic#065	B. melitensis bv3	42	116		BRU-S065	Human	2003	Kirikkale, Turkey
1	5	3	13	2	2	3	2	4	41	8	7	6	3	7	6	2011Kilic#079	B. melitensis bv3	42	116		BRU-S079	Human	2003	Kirikkale, Turkey
1	5	3	13	2	2	3	2	4	41	8	8	6	3	7	4	2018Vergnaud#1175	B. melitensis bv3	42	116		BCCN#91-49	Ovine	1991	Saragosse, Spain
1	5	3	13	2	2	3	2	4	41	8	8	1	3	10	6	2017Liu#019	B. melitensis	42	116		WS018	Human	2014	Zhuozi, China
1	5	3	13	2	2	3	2	4	42	8	8	6	11	7	6	2017Hanot-Mamb.	B. melitensis bv3	42	367		L3/278	Human	2014	Belgium
1	5	3	13	2	2	3	2	4	43	8	5	6	3	7	6	2007AlDahouk#096	B. melitensis bv3	42	108		BfR 73	Human	2000	Turkey
1	5	3	13	2	2	3	2	4	43	8	5	6	3	7	5	2011Aftab#013	B. melitensis	42	108		M48086	Human		Turkey
1	5	3	13	2	2	3	2	4	41	8	7	6	4	5	6	2011Kilic#069	B. melitensis bv3	42	116		BRU-S069	Human	2002	Kirikkale, Turkey
1	5	3	13	2	2	3	2	4	41	8	7	4	2	3	6	2017Liu#137	B. melitensis bv3	42	116		NM-9		2016	
1	5	3	13	2	2	3	2	4	41	8	7	4	2	3	6	2017Liu#140	B. melitensis bv3	42	116		NM-13		2016	
1	5	3	13	2	2	3	2	4	41	8	7	4	2	3	6	2017Liu#141	B. melitensis bv3	42	116		NM-16		2016	
1	5	3	13	2	2	3	2	4	41	8	7	4	2	5	6	2017Liu#131	B. melitensis bv3	42	116		NM-2		2016	
1	5	3	13	2	2	3	2	4	41	8	7	4	5	4	6	2017Liu#094	B. melitensis	42	116		WS093	Human	2015	Houqi, China
1	5	3	13	2	2	3	2	4	41	8	3	4	2	6	6	2017Liu#134	B. melitensis bv3	42	116		NM-5		2016	
1	5	3	13	2	2	3	2	4	41	8	3	4	2	5	6	2017Liu#136	B. melitensis bv3	42	116		NM-7		2016	
1	5	3	13	2	2	3	2	4	41	8	6	4	2	5	6	2017Liu#144	B. melitensis bv3	42	116		NM-20		2016	
1	5	3	13	2	2	3	2	7	41	8	3	4	2	5	6	2017Liu#181	B. melitensis bv3	42	369		NM-8		2016	
1	5	3	13	2	2	3	2	4	41	8	3	4	2	3	5	2017Liu#135	B. melitensis bv3	42	116		NM-6		2016	
1	5	3	13	2	2	3	2	4	41	8	5	4	2	4	5	2017Liu#138	B. melitensis bv3	42	116		NM-11		2016	
1	5	3	13	2	2	3	2	4	41	8	5	4	2	2	5	2017Liu#147	B. melitensis bv3	42	116		NM-24		2016	
1	5	3	13	2	2	3	2	4	41	8	6	4	2	4	5	2017Liu#146	B. melitensis bv3	42	116		NM-23		2016	
1	5	3	13	2	2	3	2	4	41	8	5	4	2	4	4	2017Liu#139	B. melitensis bv3	42	116		NM-12		2016	
1	5	3	13	2	2	3	2	4	41	8	12	4	2	4	4	2017Liu#145	B. melitensis bv3	42	116		NM-21		2016	
1	5	3	13	2	2	3	2	4	41	8	5	4	2	4	6	2017Liu#142	B. melitensis bv3	42	116		NM-17		2016	
1	5	3	13	2	2	3	2	4	41	8	8	4	2	5	4	2017Liu#130	B. melitensis bv3	42	116		NM-1		2016	
1	5	3	13	2	2	3	2	4	41	8	5	4	2	5	4	2017Liu#143	B. melitensis bv3	42	116		NM-18		2016	
1	5	3	13	2	2	3	2	4	41	8	8	4	2	3	4	2017Liu#133	B. melitensis bv3	42	116		NM-4		2016	
1	5	3	13	2	2	3	2	4	41	8	8	4	8	8	6	2013Jiang#011	B. melitensis bv3	42	116		NM06-7	Ovine	2006	Inner Mongolia, China
1	5	3	13	2	2	3	2	4	41	8	6	3	2	7	5	2017Liu#155	B. melitensis bv3	42	116		XB1		2016	
1	5	3	13	2	2	3	2	4	41	8	6	3	2	7	5	2017Liu#157	B. melitensis bv3	42	116		XB3		2016	
1	5	3	13	2	2	3	2	4	41	8	6	3	2	7	5	2017Liu#158	B. melitensis bv3	42	116		XB4		2016	
1	5	3	13	2	2	3	2	4	41	8	6	3	2	7	5	2017Liu#159	B. melitensis bv3	42	116		XB5		2016	
1	5	3	13	2	2	3	2	4	41	8	6	3	2	7	5	2017Liu#160	B. melitensis bv3	42	116		M644/93/1 (alia.		2016	
1	5	3	13	2	2	3	2	4	41	8	6	3	2	7	5	2017Liu#161	B. melitensis bv3	42	116		XB8		2016	
1	5	3	13	2	2	3	2	4	41	8	6	3	2	7	5	2017Liu#163	B. melitensis bv3	42	116		XB10		2016	
1	5	3	13	2	2	3	2	4	41	8	6	3	3	7	5	2017Liu#169	B. melitensis bv3	42	116		Y08		2016	
1	5	3	13	2	2	3	2	4	41	8	8	3	2	7	5	2017Liu#162	B. melitensis bv3	42	116		XB9		2016	
1	5	3	13	2	2	3	2	4	41	8	7	3	2	3	5	2017Liu#164	B. melitensis bv3	42	116		M3		2016	
1	5	3	13	2	2	3	2	4	41	8	7	3	2	3	5	2017Liu#165	B. melitensis bv3	42	116		M5		2016	
1	5	3	13	2	2	3	2	4	41	8	7	3	2	7	5	2017Liu#156	B. melitensis bv3	42	116		XB2		2016	
1	5	3	13	2	3	3	2	4	41	8	6	3	2	2	5	2017Liu#122	B. melitensis bv3	63	111		QY2		2016	
1	5	3	13	2	3	3	2	4	41	8	5	3	2	2	5	2017Liu#123	B. melitensis bv3	63	111		QY3		2016	
1	5	3	13	2	2	3	2	4	41	8	4	3	2	2	5	2017Liu#151	B. melitensis bv3	42	116		JN4		2016	
1	5	3	13	2	2	3	2	4	41	8	4	3	2	2	5	2017Liu#152	B. melitensis bv3	42	116		JN5		2016	
1	5	3	13	2	2	3	2	4	41	8	5	3	2	2	5	2017Liu#148	B. melitensis bv3	42	116		JN1		2016	
1	5	3	13	2	2	3	2	4	41	8	5	3	2	2	5	2017Liu#149	B. melitensis bv3	42	116		JN2		2016	
1	5	3	13	2	2	3	2	4	41	8	5	3	2	2	5	2017Liu#150	B. melitensis bv3	42	116		JN3		2016	
1	5	3	13	2	2	3	2	4	41	8	5	3	2	2	5	2017Liu#153	B. melitensis bv3	42	116		JN6		2016	
1	5	3	13	2	2	3	2	4	41	8	5	3	2	2	5	2017Liu#154	B. melitensis bv3	42	116		JN7		2016	
1	5	3	13	2	2	3	2	4	41	8	4	3	2	5	6	2017Liu#167	B. melitensis bv3	42	116		T06		2016	
1	5	3	13	2	2	3	2	4	41	8	4	3	2	5	6	2017Liu#168	B. melitensis bv3	42	116		T08		2016	
1	5	3	13	2	2	3	2	5	41	8	5	3	2	5	6	2017Liu#182	B. melitensis bv3	42	180		XB7		2016	
1	5	3	13	2	2	3	2	4	41	8	6	5	3	8	6	2007AlDahouk#029	B. melitensis bv2	42	116		BfR 4	Human	2002	Turkey
1	5	3	13	2	2	3	2	4	41	8	6	5	3	5	6	2017Liu#048	B. melitensis	42	116		WS047	Human	2015	Fengzhen, China
1	5	3	13	2	2	3	2	4	41	8	5	5	3	9	6	2011Jiang#032	B. melitensis bv3	42	116		NM58-27	Human	1958	Inner Mongolia, China
1	5	3	13	2	2	3	2	4	41	8	8	5	3	9	4	SRR4436585	B. melitensis	42	116	8	BwIM_TUR_38	Human	2017	Turkey
1	5	3	14	2	2	3	2	4	41	8	6	5	3	9	9	2017Hanot-Mamb.	B. melitensis bv3	83	298		L3/200	Human	2011	Belgium
1	5	3	13	2	3	3	2	4	41	8	5	5	3	8	6	2011Jiang#045	B. melitensis bv3	63	111		SHANXI09-12	Human	2009	Shanxi, China
1	5	3	13	2	3	3	2	4	41	8	5	5	3	4	4	2017Liu#119	B. melitensis bv3	63	111		NM-14		2016	
1	5	3	13	2	3	3	2	4	41	8	6	5	3	5	4	2011Jiang#078	B. melitensis bv3	63	111		LB10-07	Human	2010	Guangdong, China
1	5	3	13	3	1	3	2	4	41	8	5	5	3	9	6	2011Jiang#034	B. melitensis bv3	58	120		GX61-5	Human	1961	Guangxi, China
1	5	3	13	2	2	3	2	4	49	8	6	3	3	4	5	2011Jiang#076	B. melitensis bv3	42	296		LB10-05	Human	2010	Guangdong, China
1	5	3	13	2	2	3	2	4	45	8	6	8	3	4	6	2012Ferreira#048	B. melitensis bv3	42	179		LNIV-499Bm3-05	unknown	2005	Tras-os-Montes, Portugal
1	5	3	13	2	2	3	2	4	9	8	3	4	3	4	6	2018Vergnaud#0807	B. melitensis bv1	42	295		BCCN#93-45	Human	1993	Navarre, Spain
1	4	3	13	3	2	3	2	4	41	8	5	4	3	4	5	2006LeFleche#090	B. melitensis bv3	44	110		BCCN#96-31	Ovine	1996	Israel
1	4	3	13	3	2	3	2	4	41	8	6	4	3	4	5	2006LeFleche#091	B. melitensis bv3	44	110		BCCN#96-32	Ovine	1996	Israel
1	4	3	13	3	2	3	2	4	41	8	8	4	3	7	5	2017Hanot-Mamb.	B. melitensis bv3	44	110		L3/135	Human	2004	Belgium
1	4	3	13	3	2	3	2	4	41	8	8	4	3	7	5	2017Hanot-Mamb.	B. melitensis bv3	44	110		L3/136	Human	2004	Belgium
1	4	3	13	3	2	3	2	4	41	8	8	4	3	7	5	2018Vergnaud#0987	B. melitensis bv1	44	110		BCCN#00-29	Human	2000	Paris, France
1	4	3	13	3	2	3	2	4	41	8	8	4	3	8	5	2008Kattar#023	B. melitensis	44	110		AUB BRUP-S34	Human		Lebanon
1	4	3	13	3	2	3	2	4	43	8	6	4	3	6	5	2018Vergnaud#0851	B. melitensis bv1	44	280		BCCN#76-424	Human	1976	Indre-et-Loire, France
1	4	3	13	3	2	3	2	4	46	8	4	4	3	5	4	2011Kilic#098	B. melitensis bv3	44	109		BRU-S098	Human	2008	Istanbul, Turkey
1	4	3	13	3	2	3	2	4	46	8	4	4	3	5	4	2011Kilic#131	B. melitensis bv3	44	109		BRU-S131	Human	1998	Malatya, Turkey
1	5	3	13	3	2	3	2	4	41	8	8	4	3	6	6	2011Kilic#056	B. melitensis bv3	43	125		BRU-S056	Human	2008	Kutahya, Turkey
1	5	3	13	3	2	3	2	4	41	8	9	4	3	6	6	2017Campbell#002	B. melitensis	43	125		#2	Human	2017	Vietnam
1	5	3	13	3	2	3	2	4	41	8	3	4	3	7	6	2008Kattar#003	B. melitensis	43	125		AUB BRUP-S11	Human		Lebanon
1	5	3	13	3	2	3	2	4	41	8	8	4	3	7	6	2017Campbell#007	B. melitensis	43	125		#7	Human	2017	Vietnam
1	5	3	13	3	2	3	2	4	41	8	4	4	3	5	6	2007AlDahouk#054	B. melitensis bv1	43	125		BfR 13	Human	1999	Turkey
1	5	3	13	3	2	3	2	4	41	8	4	4	3	5	6	2011Kilic#112	B. melitensis bv3	43	125		BRU-S112	Human	2005	Diyarbakir, Turkey
1	5	3	13	3	2	3	2	4	41	8	6	4	3	5	6	2018Vergnaud#0363	B. melitensis R.	43	125		BCCN#93-40	Human	1993	Palestine Cis Jordanie,.
1	5	3	13	3	2	3	2	4	41	8	11	4	3	5	6	2017Campbell#003	B. melitensis	43	125		#3	Human	2017	Vietnam
1	5	3	13	3	2	3	2	4	41	8	10	4	3	5	6	2017Campbell#005	B. melitensis	43	125		#5	Human	2017	Vietnam

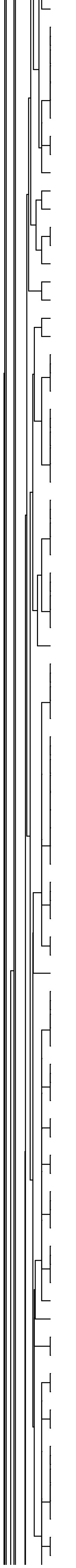

1	5	3	13	3	2	3	2	4	41	8	9	4	3	5	6	2017Campbell#009	B. melitensis	43	125		#9	Human	2017	Vietnam
1	5	3	13	3	2	3	2	4	41	8	5	4	3	5	6	2011Jiang#005	B. melitensis bv3	43	125		CHS08-1	Human	2008	Hunan, China
1	5	3	13	3	2	3	2	4	41	8	5	4	3	5	6	2011Kilic#024	B. melitensis bv3	43	125		BRU-S024	Human	2005	Erzurum, Turkey
1	5	3	13	3	2	3	2	4	41	8	5	4	3	5	6	2011Kilic#106	B. melitensis bv3	43	125		BRU-S106	Human	2006	Diyarbakir, Turkey
1	5	3	13	3	2	3	2	4	41	8	5	4	3	5	6	2011Kilic#109	B. melitensis bv3	43	125		BRU-S109	Human	2005	Diyarbakir, Turkey
1	5	3	13	3	2	3	2	4	41	8	5	4	3	5	6	2011Kilic#136	B. melitensis bv3	43	125		BRU-S136	Human	2001	Malatya, Turkey
1	5	3	13	3	2	3	2	4	41	8	5	4	3	5	6	SRR4436582	B. melitensis	43	125	8	BwIM_TUR_27	Human	2017	Turkey
1	5	3	13	3	2	3	2	4	41	8	5	4	3	9	6	2008Kattar#027	B. melitensis	43	125		AUB BRUP-S41	Human		Lebanon
1	5	3	13	3	2	3	2	4	41	8	5	4	3	9	6	2011Kilic#021	B. melitensis bv3	43	125		BRU-S021	Human	2007	Erzurum, Turkey
1	5	3	13	3	2	3	2	4	41	8	5	4	3	8	6	2011Kilic#049	B. melitensis bv3	43	125		BRU-S049	Human	2003	Izmir, Turkey
1	5	3	13	3	2	3	2	4	41	8	7	4	3	8	6	2018Vergnaud#0445	B. melitensis bv1	43	125		BCCN#83-194	Caprine	1983	Greece
1	5	3	13	3	2	3	2	4	41	8	9	4	3	8	6	2018Vergnaud#1381	B. melitensis bv3	43	125		BCCN#83-188	Ovine	1983	Greece
1	5	3	13	3	2	3	2	4	41	8	3	4	3	8	5	2018Vergnaud#0069	B. melitensis bv1	43	125		BCCN#06-57	Human	2006	France
1	5	3	13	3	2	3	2	4	41	8	3	4	3	8	5	2018Vergnaud#0070	B. melitensis bv1	43	125		BCCN#06-59	Human	2006	France
1	5	3	13	3	2	3	2	4	41	8	3	4	3	8	6	2008Kattar#016	B. melitensis	43	125		AUB BRUP-S27	Human		Lebanon
1	5	3	13	2	2	3	2	4	41	8	7	4	3	10	6	2011Jiang#086	B. melitensis bv3	42	116		LB10-16	Human	2010	Guangdong, China
1	5	3	13	3	2	3	2	4	41	8	7	4	3	10	6	SRR4436606	B. melitensis	43	125		BwIM_TUR_15	Human	2017	Turkey
1	5	3	13	3	2	3	2	4	41	8	4	4	3	11	5	2008Kattar#017	B. melitensis	43	125		AUB BRUP-S28	Human		Lebanon
1	5	3	13	2	2	3	2	4	41	8	4	4	3	11	5	2017Liu#082	B. melitensis	42	116		WS081	Human	2015	Shangdu, China
1	5	3	13	3	2	3	2	4	41	8	4	5	3	4	5	2008Kattar#001	B. melitensis	43	125		AUB BRUP-S1	Human		Lebanon
1	5	3	13	3	2	3	2	4	41	8	4	5	3	4	5	2011Aftab#003	B. melitensis	43	125		REF 3181-03	Human		Turkey
1	5	3	13	3	2	3	2	4	41	8	4	5	3	4	5	2011Aftab#005	B. melitensis	43	125		REF 3353-04	Human		Turkey
1	5	3	13	3	2	3	2	4	41	8	4	4	3	4	5	2007AlDahouk#086	B. melitensis bv3	43	125		BfR 62	Human	2001	Irak
1	5	3	13	3	2	3	2	4	41	8	4	4	3	4	5	2011Kilic#054	B. melitensis bv3	43	125		BRU-S054	Human	2008	Kutahya, Turkey
1	5	3	13	3	2	3	2	4	41	8	4	4	3	4	5	2011Kilic#120	B. melitensis bv3	43	125		BRU-S120	Human	2007	Sirnak, Turkey
1	5	3	13	3	2	3	2	4	41	8	4	4	3	4	5	2018Vergnaud#0568	B. melitensis bv3	43	125		BCCN#86-9	Human	1986	France
1	5	3	13	3	2	3	2	4	41	8	4	4	3	4	5	SRR4436607	B. melitensis	43	125	38	BwIM_TUR_19	Human	2017	Turkey
1	5	3	13	3	2	3	2	4	9	8	4	4	3	8	5	2018Vergnaud#0035	B. melitensis bv1	43	292		BCCN#75-4	Human	1975	Navarre, Spain
1	5	3	13	3	2	3	2	4	9	8	4	4	3	8	5	2018Vergnaud#0075	B. melitensis bv1	43	292		BCCN#74-263	Human	1974	Navarre, Spain
1	5	3	13	3	2	3	2	4	9	8	4	4	3	8	5	2018Vergnaud#0078	B. melitensis bv1	43	292		BCCN#74-268	Human	1974	Navarre, Spain
1	5	3	13	3	2	3	2	4	9	8	4	4	3	8	5	2018Vergnaud#1219	B. melitensis bv1	43	292		BCCN#74-265	Human	1974	Navarre, Spain
1	5	3	13	3	2	3	2	4	41	8	4	4	3	8	5	2008Kattar#013	B. melitensis	43	125		AUB BRUP-S23	Human		Lebanon
1	5	3	13	3	2	3	2	4	41	8	4	4	3	8	5	SRR4436593	B. melitensis	43	125	38	BwIM_IRQ_16a	Human		Iraq
1	5	3	13	3	2	3	2	4	41	8	4	4	3	8	5	SRR4436593_SR.	B. melitensis	43	125	22			2017	
1	5	3	13	3	2	3	2	4	41	8	4	4	3	8	5	SRR4436594	B. melitensis	43	125	38	BwIM_IRQ_16b	Human		Iraq
1	5	3	13	3	2	3	2	4	9	8	6	4	3	8	5	2018Vergnaud#0874	B. melitensis bv1	43	292		BCCN#76-405	Human	1976	Navarre, Spain
1	5	3	13	3	2	3	2	4	41	8	7	4	3	12	5	2018Vergnaud#0824	B. melitensis bv3	43	125		BCCN#83-172	Ovine	1983	Greece
1	5	3	13	3	2	3	2	4	41	8	7	4	3	12	5	2018Vergnaud#0825	B. melitensis bv3	43	125		BCCN#83-173	Ovine	1983	Greece
1	5	3	13	3	2	3	2	4	41	8	7	4	3	12	5	2018Vergnaud#0950	B. melitensis bv3	43	125		BCCN#83-190	Ovine	1983	Greece
1	5	3	13	3	2	3	2	4	41	8	7	4	3	12	5	2018Vergnaud#0966	B. melitensis bv3	43	125		BCCN#83-176	Ovine	1983	Greece
1	5	3	13	3	2	3	2	4	41	8	7	4	3	6	5	2018Vergnaud#0432	B. melitensis bv3	43	125		BCCN#91-101	Cattle	1991	Salonique ,Greece
1	5	3	13	3	2	3	2	4	41	8	7	4	3	6	5	2018Vergnaud#0592	B. melitensis bv3	43	125		BCCN#91-102	Cattle	1991	Salonique ,Greece
1	5	3	13	3	2	3	2	4	41	8	7	4	3	6	5	2018Vergnaud#0833	B. melitensis bv3	43	125		BCCN#91-104	Cattle	1991	Salonique ,Greece
1	5	3	13	3	2	3	2	4	41	8	7	4	3	6	5	2018Vergnaud#1137	B. melitensis bv3	43	125		BCCN#91-105	Cattle	1991	Salonique ,Greece
1	5	3	13	3	2	3	2	4	41	8	7	4	3	6	5	2018Vergnaud#1138	B. melitensis bv3	43	125		BCCN#91-106	Cattle	1991	Salonique ,Greece
1	5	3	13	3	2	3	2	4	41	8	7	4	3	6	5	2018Vergnaud#1139	B. melitensis bv3	43	125		BCCN#91-107	Cattle	1991	Salonique ,Greece
1	5	3	13	3	2	3	2	4	41	8	7	4	3	6	5	2018Vergnaud#1161	B. melitensis bv3	43	125		BCCN#91-108	Cattle	1991	Salonique ,Greece
1	5	3	13	3	2	3	2	4	41	8	7	4	3	6	5	2018Vergnaud#1162	B. melitensis bv3	43	125		BCCN#91-109	Cattle	1991	Salonique ,Greece
1	5	3	13	3	2	3	2	4	41	8	7	4	3	4	5	2011Kilic#141	B. melitensis bv3	43	125		BRU-S141	Human	2004	Malatya, Turkey
1	5	3	13	3	2	3	2	4	41	8	7	4	3	4	5	2018Vergnaud#0129	B. melitensis bv3	43	125		BCCN#90-128	Cattle	1990	Salonique ,Greece
1	5	3	13	3	2	3	2	4	41	8	7	4	3	4	5	SRR4436574	B. melitensis	43	125	38	BwIM_SYR_14	Human	2017	Syria
1	5	3	13	3	2	3	2	4	41	8	7	4	3	7	5	2011Kilic#015	B. melitensis bv3	43	125		BRU-S015	Human	2005	Antalya, Turkey
1	5	3	13	3	2	3	2	4	41	8	7	4	3	7	5	2011Kilic#016	B. melitensis bv3	43	125		BRU-S016	Human	2005	Antalya, Turkey
1	5	3	13	3	2	3	2	4	41	8	7	4	3	5	9	2017Campbell#008	B. melitensis	43	125		#8	Human	2017	Vietnam
1	5	3	13	3	2	3	2	4	41	8	5	4	3	7	5	SRR4436572	B. melitensis	43	125	8	BwIM_TUR_35c	Human		Turkey
1	5	3	13	3	2	3	2	4	41	8	5	4	3	7	5	SRR4436603	B. melitensis	43	125	8	BwIM_TUR_35a	Human		Turkey
1	5	3	13	3	2	3	2	4	41	8	5	4	3	7	5	SRR4436632	B. melitensis	43	125	8	BwIM_TUR_35b	Human		Turkey
1	5	3	13	3	2	3	2	4	41	8	5	4	3	7	5	SRR4436632_SR.	B. melitensis	43	125	8			2017	
1	5	3	13	3	2	3	2	4	41	8	5	4	3	9	5	2018Vergnaud#0734	B. melitensis bv3	43	125		BCCN#83-180	Ovine	1983	Greece
1	5	3	13	3	2	3	2	4	41	8	5	4	3	9	5	2018Vergnaud#0735	B. melitensis bv3	43	125		BCCN#83-181	Ovine	1983	Greece
1	5	3	13	3	2	3	2	4	41	8	5	4	3	9	5	2018Vergnaud#1380	B. melitensis bv3	43	125		BCCN#83-182	Ovine	1983	Greece
1	5	3	13	3	2	3	2	4	41	8	5	4	3	6	5	2013Garofolo_19.	B. melitensis bv3	43	125		19513	Human	2011	Ferrara,Italy
1	5	3	13	3	2	3	2	4	41	8	5	4	3	6	5	2017Hanot-Mamb.	B. melitensis bv3	43	125		L3/134	Human	2002	Belgium
1	5	3	13	3	2	3	2	4	41	8	5	4	3	8	5	2011Jiang#039	B. melitensis bv3	43	125		GD09-3	Human	2009	Guangdong, China
1	5	3	13	3	2	3	2	4	41	8	5	4	3	8	5	2011Kilic#124	B. melitensis bv3	43	125		BRU-S124	Human	2007	Sirnak, Turkey
1	5	3	13	3	2	3	2	4	41	8	5	4	3	5	5	2008Kattar#004	B. melitensis	43	125		AUB BRUP-S12	Human		Lebanon
1	5	3	13	3	2	3	2	4	41	8	5	4	3	5	5	2011Kilic#032	B. melitensis bv3	43	125		BRU-S032	Human	2007	Erzurum, Turkey
1	5	3	13	3	2	3	2	4	41	8	5	4	3	5	5	2011Kilic#050	B. melitensis bv3	43	125		BRU-S050	Human	2008	Kutahya, Turkey
1	5	3	13	3	2	3	2	4	41	8	5	4	3	4	5	2007AlDahouk#038	B. melitensis bv1	43	125		BfR 15	Human	2002	Turkey
1	5	3	13	3	2	3	2	4	41	8	5	4	3	4	5	2011Kilic#039	B. melitensis bv3	43	125		BRU-S039	Human	2008	Ardahan, Turkey
1	5	3	13	3	2	3	2	4	41	8	5	4	3	4	5	2011Kilic#134	B. melitensis bv3	43	125		BRU-S134	Human	2002	Malatya, Turkey
1	5	3	13	3	2	3	2	4	41	8	5	4	3	10	5	2017Hanot-Mamb.	B. melitensis bv3	43	125		L3/141	Human	2004	Belgium
1	5	3	13	3	2	3	2	4	41	8	5	5	3	7	5	2007AlDahouk#042	B. melitensis bv2	43	125		BfR III	Human	2003	Turkey
1	5	3	13	3	3	3	2	4	41	8	5	4	3	5	5	2011Kilic#104	B. melitensis bv3	57	112		BRU-S104	Human	2004	Gaziantep, Turkey
1	5	3	13	3	3	3	2	4	41	8	5	4	3	5	5	2011Kilic#105	B. melitensis bv3	57	112		BRU-S105	Human	2004	Gaziantep, Turkey
1	5	3	13	3	2	3	2	4	41	8	6	4	3	5	5	2011Kilic#019	B. melitensis bv3	43	125		BRU-S019	Human	2005	Agri, Turkey
1	5	3	13	3	2	3	2	4	41	8	6	4	3	5	5	2011Kilic#026	B. melitensis bv3	43	125		BRU-S026	Human	2005	Erzurum, Turkey
1	5	3	13	3	2	3	2	4	41	8	8	4	3	5	5	2011Kilic#009	B. melitensis bv3	43	125		BRU-S009	Human	2004	Adana, Turkey
1	5	3	13	3	2	3	2	4	41	8	8	4	3	5	5	2017Campbell#001	B. melitensis	43	125		#1	Human	2016	Vietnam
1	5	3	13	3	2	3	2	4	41	8	4	4	3	5	5	2007AlDahouk#045	B. melitensis bv2	43	125		BfR IX	Human	2003	Turkey
1	5	3	13	3	2	3	2	4	41	8	4	4	3	5	5	2011Kilic#145	B. melitensis bv3	43	125		BRU-S145	Human	2004	Malatya, Turkey
1	5	3	13	3	2	3	2	4	41	8	4	4	3	5	5	2011Kilic#146	B. melitensis bv3	43	125		BRU-S146	Human	2004	Malatya, Turkey
1	5	3	13	3	2	3	2	4	41	8	4	4	3	5	5	SRR4436580	B. melitensis	43	125	38	BwIM_IRQ_43	Human	2017	Iraq
1	5	3	13	3	2	3	2	4	41	8	4	4	3	5	5	SRR4436581	B. melitensis	43	125	38	BwIM_SYR_34	Human	2017	Syria
1	5	3	13	3	2	3	2	4	41	8	3	4	3	5	5	2007AlDahouk#033	B. melitensis bv1	43	125		BfR 8	Human	2002	Turkey
1	5	3	13	3	2	3	2	4	41	8	3	4	3	5	5	2011Kilic#003	B. melitensis bv3	43	125		BRU-S003	Human	2004	Hatay, Turkey

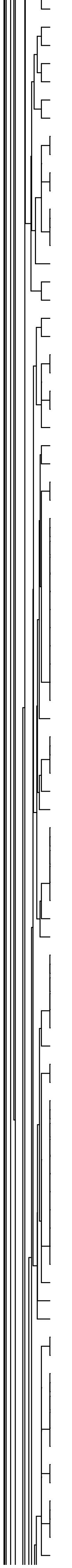

1	5	3	13	3	2	3	2	4	41	8	9	4	3	5	5	2011Kilic#001	B. melitensis bv3	43	125		BRU-S001	Human	2004	Adana, Turkey
1	5	3	13	3	2	3	2	4	41	8	5	4	3	5	7	2011Kilic#090	B. melitensis bv3	43	125		BRU-S090	Human	2007	Trabzon, Turkey
1	5	3	13	3	2	3	2	4	41	8	9	4	3	5	7	2018Vergnaud#0395	B. melitensis bv3	43	125		BCCN#90-134	Cattle	1990	Salonique ,Greece
1	5	3	13	2	2	3	2	4	41	8	8	4	3	5	7	2007AlDahouk#041	B. melitensis bv1	42	116		BfR II	Human	2003	
1	5	3	13	3	2	3	2	4	41	8	8	4	3	5	7	2011Kilic#031	B. melitensis bv3	43	125		BRU-S031	Human	2007	Bayburt, Turkey
1	5	3	13	3	2	3	2	4	41	8	5	4	3	7	7	2011Jiang#042	B. melitensis bv3	43	125		SHANXI09-15	Human	2009	Shanxi, China
1	5	3	13	3	2	3	2	4	41	8	8	4	3	7	7	2011Kilic#110	B. melitensis bv3	43	125		BRU-S110	Human	2003	Diyarbakir, Turkey
1	5	3	13	3	2	3	2	4	41	8	7	4	3	6	7	2017Campbell#006	B. melitensis	43	125		#6	Human	2017	Vietnam
1	5	3	13	3	2	3	2	4	41	8	7	4	3	6	7	2018Vergnaud#0776	B. melitensis bv3	43	125		BCCN#83-148	Ovine	1983	Greece
1	5	3	13	3	2	3	2	4	41	8	6	4	3	6	7	2011Kilic#103	B. melitensis bv3	43	125		BRU-S103	Human	2008	Istanbul, Turkey
1	5	3	13	3	2	3	2	4	41	8	6	4	3	6	7	SRR4436622	B. melitensis	43	125	8	BwIM_ALB_46	Human	2017	Albania
1	5	3	13	3	2	3	2	4	41	8	5	4	3	6	7	2011Kilic#058	B. melitensis bv3	43	125		BRU-S058	Human	2008	Kutahya, Turkey
1	5	3	13	3	2	3	2	4	41	8	5	4	3	6	7	2011Kilic#059	B. melitensis bv3	43	125		BRU-S059	Human	2008	Kutahya, Turkey
1	5	3	13	3	2	3	2	4	41	8	5	4	3	6	7	2011Kilic#085	B. melitensis bv3	43	125		BRU-S085	Human	2006	Artvin, Turkey
1	5	3	13	3	2	3	2	4	41	8	7	7	3	6	7	2018Vergnaud#0387	B. melitensis bv3	43	125		BCCN#83-168	Caprine	1983	Greece
1	5	3	13	3	2	3	2	4	41	8	5	6	3	8	7	2011Jiang#035	B. melitensis bv3	43	125		HN63-15	Human	1963	Henan, China
1	5	3	13	3	2	3	2	4	41	8	5	4	3	8	7	2011Kilic#108	B. melitensis bv3	43	125		BRU-S108	Human	2003	Diyarbakir, Turkey
1	5	3	13	3	2	3	2	4	41	8	4	4	3	9	6	2011Kilic#025	B. melitensis bv3	43	125		BRU-S025	Human	2007	Erzurum, Turkey
1	5	3	13	3	2	3	2	4	41	8	4	4	3	9	5	2017Hanot-Mamb.	B. melitensis bv2	43	125		L3/148	Human	2007	Belgium
1	5	3	13	3	2	3	2	4	41	8	5	4	3	9	4	2011Kilic#128	B. melitensis bv3	43	125		BRU-S128	Human	2004	Adiyaman, Turkey
1	5	3	13	3	2	3	2	4	41	8	5	4	3	9	4	2011Kilic#142	B. melitensis bv3	43	125		BRU-S142	Human	2003	Malatya, Turkey
1	5	3	13	3	2	3	2	4	41	8	4	4	3	9	4	2011Kilic#013	B. melitensis bv3	43	125		BRU-S013	Human	2006	Adana, Turkey
1	5	3	13	3	2	3	2	4	41	8	4	4	3	9	4	2017Campbell#010	B. melitensis	43	125		#10	Human	2017	Vietnam
1	5	3	13	3	2	3	2	4	41	8	6	4	3	9	4	2016Gyuranecz#015	B. melitensis	43	125		C15	Camel	2015	Sudan
1	5	3	13	3	2	3	2	4	41	8	9	4	3	4	4	2011Kilic#020	B. melitensis bv3	43	125		BRU-S020	Human	2005	Agri, Turkey
1	5	3	13	3	2	3	2	4	41	8	8	4	3	4	4	2011Kilic#040	B. melitensis bv3	43	125		BRU-S040	Human	2008	Kars, Turkey
1	5	3	13	3	2	3	2	4	43	8	4	4	3	4	4	2011Aftab#010	B. melitensis	43	122		S34686	Human		Turkey
1	5	3	13	3	2	3	2	4	43	8	4	4	3	4	4	2011Aftab#011	B. melitensis	43	122		H13944	Human		Kurdistan or Turkey
1	5	3	13	3	2	3	2	4	41	8	4	4	3	4	4	2007AlDahouk#044	B. melitensis bv2	43	125		BfR VII	Human	2003	Syria
1	5	3	13	3	2	3	2	4	41	8	4	4	3	4	4	2011Kilic#002	B. melitensis bv3	43	125		BRU-S002	Human	2003	Hatay, Turkey
1	5	3	13	3	2	3	2	4	41	8	4	4	3	4	4	2011Kilic#022	B. melitensis bv3	43	125		BRU-S022	Human	2007	Bayburt, Turkey
1	5	3	13	3	2	3	2	4	41	8	4	4	3	4	4	2011Kilic#113	B. melitensis bv3	43	125		BRU-S113	Human	2004	Diyarbakir, Turkey
1	5	3	13	3	2	3	2	4	41	8	4	4	3	4	4	2011Kilic#130	B. melitensis bv3	43	125		BRU-S130	Human	2009	Elazig, Turkey
1	5	3	13	3	2	3	2	4	41	8	4	4	3	4	4	2011Kilic#132	B. melitensis bv3	43	125		BRU-S132	Human	2002	Malatya, Turkey
1	5	3	13	3	2	3	2	4	41	8	4	4	3	4	4	2011Kilic#133	B. melitensis bv3	43	125		BRU-S133	Human	2003	Malatya, Turkey
1	5	3	13	3	2	3	2	4	41	8	4	4	3	4	4	2011Kilic#135	B. melitensis bv3	43	125		BRU-S135	Human	2002	Malatya, Turkey
1	5	3	13	3	2	3	2	4	41	8	4	4	3	4	4	2011Kilic#148	B. melitensis bv3	43	125		BRU-S148	Human	2004	Malatya, Turkey
1	5	3	13	3	2	3	2	4	41	8	4	4	3	4	4	SRR4436619	B. melitensis	43	125	38	BwIM_TUR_02	Human	2017	Turkey
1	5	3	13	3	2	3	2	4	41	8	4	4	3	4	4	SRR4436630	B. melitensis	43	125	38	BwIM_SYR_04	Human	2017	Syria
1	5	3	13	3	3	3	2	4	41	8	4	4	3	4	4	SRR4436591	B. melitensis	57	112		BwIM_XXX_54	Human	2017	
1	5	3	13	3	2	3	2	4	41	8	4	4	3	7	4	2008Kattar#005	B. melitensis	43	125		AUB BRUP-S13	Human		Lebanon
1	5	3	13	3	2	3	2	4	41	8	4	4	3	7	4	2011Kilic#017	B. melitensis bv3	43	125		BRU-S017	Human	2008	Antalya, Turkey
1	5	3	13	3	2	3	2	4	41	8	4	4	3	7	4	2011Kilic#117	B. melitensis bv3	43	125		BRU-S117	Human	2007	Sirnak, Turkey
1	5	3	13	3	2	3	2	4	9	8	4	4	3	7	4	2018Vergnaud#0800	B. melitensis bv1	43	292		BCCN#75-2	Human	1975	France
1	5	3	13	3	2	3	2	4	41	8	4	4	3	8	4	2011Kilic#119	B. melitensis bv3	43	125		BRU-S119	Human	2007	Sirnak, Turkey
1	5	3	13	3	2	3	2	4	41	8	4	4	3	6	4	2007AlDahouk#043	B. melitensis bv2	43	125		BfR IV	Human	2003	Lebanon
1	5	3	13	3	2	3	2	4	41	8	4	4	3	6	4	SRR4436583	B. melitensis	43	125	38	BwIM_SYR_53	Human	2017	Syria
1	5	3	13	3	2	3	2	4	41	8	4	4	3	6	4	SRR4436614	B. melitensis	43	125	38	BwIM_SYR_10	Human	2017	Syria
1	5	3	13	3	2	3	2	4	41	8	4	4	3	6	4	SRR4436615	B. melitensis	43	125	38	BwIM_SYR_08	Human	2017	Syria
1	5	3	13	3	2	3	2	4	41	8	4	4	3	6	4	SRR4436625	B. melitensis	43	125	38	BwIM_SYR_26	Human	2017	Syria
1	5	3	13	3	2	3	2	4	41	8	4	4	6	6	4	SRR4436602	B. melitensis	43	125	38	BwIM_TUR_30	Human	2017	Turkey
1	4	3	13	3	2	3	2	4	41	8	4	4	3	6	4	2017Campbell#004	B. melitensis	44	110		#4	Human	2017	Vietnam
1	5	3	13	3	2	3	2	4	41	8	5	4	3	6	4	2007AlDahouk#067	B. melitensis bv2	43	125		BfR 34	Human	2000	Turkey
1	5	3	13	3	2	3	2	4	41	8	5	4	3	6	4	2011Aftab#002	B. melitensis	43	125		REF 1929-01	Human		Turkey
1	5	3	13	3	2	3	2	4	41	8	5	4	3	6	4	2011Kilic#035	B. melitensis bv3	43	125		BRU-S035	Human	2007	Agri, Turkey
1	5	3	13	3	2	3	2	4	41	8	5	4	3	6	4	2011Kilic#037	B. melitensis bv3	43	125		BRU-S037	Human	2007	Erzurum, Turkey
1	5	3	13	3	2	3	2	4	41	8	5	4	3	6	4	2011Kilic#099	B. melitensis bv3	43	125		BRU-S099	Human	2008	Istanbul, Turkey
1	5	3	13	3	2	3	2	4	41	8	5	4	3	7	4	2011Kilic#140	B. melitensis bv3	43	125		BRU-S140	Human	2003	Malatya, Turkey
1	5	3	13	3	2	3	2	4	41	8	5	4	3	4	7	2011Jiang#007	B. melitensis bv3	43	125		SHANXI09-2	Human	2009	Shanxi, China
1	5	3	13	3	2	3	2	4	41	8	5	4	3	4	7	2011Kilic#047	B. melitensis bv3	43	125		BRU-S047	Human	2008	Igdir, Turkey
1	5	3	13	3	2	3	2	4	41	8	5	4	3	4	4	2007AlDahouk#088	B. melitensis bv3	43	125		BfR 64	Human	1996	Turkey
1	5	3	13	3	2	3	2	4	41	8	5	4	3	4	4	2011Kilic#027	B. melitensis bv3	43	125		BRU-S027	Human	2005	Agri, Turkey
1	5	3	13	3	2	3	2	4	41	8	5	4	3	4	4	2011Kilic#033	B. melitensis bv3	43	125		BRU-S033	Human	2007	Agri, Turkey
1	5	3	13	3	2	3	2	4	41	8	5	4	3	4	4	2011Kilic#038	B. melitensis bv3	43	125		BRU-S038	Human	2007	Bingšl, Turkey
1	5	3	13	3	2	3	2	4	41	8	5	4	3	4	4	2011Kilic#042	B. melitensis bv3	43	125		BRU-S042	Human	2008	Kars, Turkey
1	5	3	13	3	2	3	2	4	41	8	5	4	3	4	4	2011Kilic#043	B. melitensis bv3	43	125		BRU-S043	Human	2008	Bingšl, Turkey
1	5	3	13	3	2	3	2	4	41	8	5	4	3	4	4	2011Kilic#121	B. melitensis bv3	43	125		BRU-S121	Human	2007	Sirnak, Turkey
1	5	3	13	3	2	3	2	4	41	8	5	4	3	4	4	2011Kilic#156	B. melitensis bv3	43	125		BRU-S156	Human	2004	Malatya, Turkey
1	5	3	13	3	2	3	2	4	41	8	5	4	3	4	4	SRR4436600	B. melitensis	43	125	38	BwIM_TUR_48	Human	2017	Turkey
1	5	3	13	3	2	3	2	4	41	8	5	4	3	4	4	SRR4436608	B. melitensis	43	125	38	BwIM_IRQ_32	Human	2017	Iraq
1	5	3	13	3	2	3	2	4	41	8	5	4	3	4	6	2011Kilic#023	B. melitensis bv3	43	125		BRU-S023	Human	2005	Erzurum, Turkey
1	5	3	13	3	2	3	2	5	41	8	5	4	3	4	4	2007AlDahouk#036	B. melitensis bv2	43	106		BfR 12	Human	2002	Greece
1	5	3	13	3	2	3	3	4	41	8	5	4	3	4	4	2017Hanot-Mamb.	B. melitensis bv3	187	365		L3/206	Human	2012	Belgium
1	5	3	13	3	2	3	2	4	41	8	6	4	3	7	4	2011Kilic#060	B. melitensis bv3	43	125		BRU-S060	Human	2002	Kirikkale, Turkey
1	5	3	13	3	2	3	2	4	41	8	6	4	3	7	4	2017Hanot-Mamb.	B. melitensis bv2	43	125		L3/202	Human	2011	Belgium
1	5	3	13	3	2	3	2	4	41	8	6	4	3	4	4	2011Kilic#028	B. melitensis bv3	43	125		BRU-S028	Human	2005	Agri, Turkey
1	5	3	13	3	2	3	2	4	41	8	6	4	3	4	4	2011Kilic#030	B. melitensis bv3	43	125		BRU-S030	Human	2007	Bingšl, Turkey
1	5	3	13	3	2	3	2	4	41	8	6	4	3	4	4	2011Kilic#046	B. melitensis bv3	43	125		BRU-S046	Human	2008	Igdir, Turkey
1	5	3	13	3	2	3	2	4	41	8	6	4	3	4	4	2011Kilic#118	B. melitensis bv3	43	125		BRU-S118	Human	2007	Sirnak, Turkey
1	5	3	13	3	2	3	2	4	41	8	6	4	3	4	4	2011Kilic#122	B. melitensis bv3	43	125		BRU-S122	Human	2007	Sirnak, Turkey
1	5	3	13	3	2	3	2	4	41	8	6	4	3	6	4	2007AlDahouk#059	B. melitensis bv1	43	125		BfR 19	Human	1995	Mannheim, Germany
1	5	3	13	3	2	3	2	4	41	8	6	4	3	6	4	2011Kilic#123	B. melitensis bv3	43	125		BRU-S123	Human	2007	Sirnak, Turkey
1	5	3	13	3	2	3	2	4	41	8	6	4	3	5	4	2007AlDahouk#028	B. melitensis bv2	43	125		BfR 3	Human	2002	Turkey
1	5	3	13	3	2	3	2	4	41	8	6	4	3	5	4	2011Kilic#149	B. melitensis bv3	43	125		BRU-S149	Human	2005	Malatya, Turkey
1	5	3	13	3	2	3	2	4	41	8	6	4	3	5	4	SRR4436612	B. melitensis	43	125	38	BwIM_SYR_41	Human	2017	Syria
1	5	3	13	3	2	3	2	4	41	8	6	4	3	3	4	2017Hanot-Mamb.	B. melitensis bv2	43	125		L3/150	Human	2007	Belgium

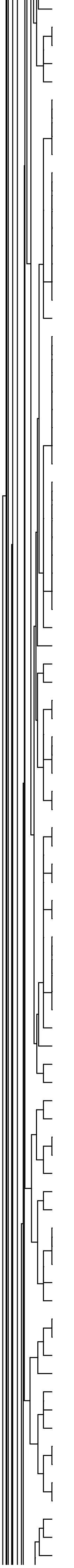

1	5	3	13	3	2	3	2	4	41	8	6	4	3	4	6	2016Gyuranecz#012	B. melitensis	43	125		C12	Caprine	2014	United Arab Emirates
1	5	3	13	3	2	3	2	4	41	8	6	4	3	7	5	2007AlDahouk#056	B. melitensis bv1	43	125		BfR 16	Human	2001	Stuttgart, Germany
1	5	3	13	3	2	3	2	4	41	8	6	4	3	7	5	SRR4436576	B. melitensis	43	125		BwIM_IRQ_11	Human	2017	Iraq
1	5	3	13	3	2	3	2	4	41	8	6	4	3	11	5	2011Kilic#155	B. melitensis bv3	43	125		BRU-S155	Human	2004	Malatya, Turkey
1	5	3	13	3	2	3	2	4	41	8	6	4	3	4	5	2011Kilic#045	B. melitensis bv3	43	125		BRU-S045	Human	2008	Igdir, Turkey
1	5	3	13	3	2	3	2	4	41	8	7	4	3	5	4	2008Kattar#008	B. melitensis	43	125		AUB BRUP-S17	Human		Lebanon
1	5	3	13	3	2	3	2	4	41	8	7	4	3	5	4	2011Kilic#010	B. melitensis bv3	43	125		BRU-S010	Human	2004	Adana, Turkey
1	5	3	13	3	2	3	2	4	41	8	7	4	3	5	4	2011Kilic#062	B. melitensis bv3	43	125		BRU-S062	Human	2003	Kirikkale, Turkey
1	5	3	13	3	2	3	2	4	41	8	7	4	3	5	4	SRR4436641	B. melitensis	43	125	38	BwIM_TUR_17	Human	2017	Turkey
1	5	3	13	3	2	3	2	4	41	8	5	4	3	5	4	2007AlDahouk#047	B. melitensis bv2	43	125		BfR XII	Human	2003	Turkey
1	5	3	13	3	2	3	2	4	41	8	5	4	3	5	4	2011Kilic#004	B. melitensis bv3	43	125		BRU-S004	Human	2003	Hatay, Turkey
1	5	3	13	3	2	3	2	4	41	8	5	4	3	5	4	2011Kilic#114	B. melitensis bv3	43	125		BRU-S114	Human	2003	Diyarbakir, Turkey
1	5	3	13	3	2	3	2	4	41	8	5	4	3	5	4	2011Kilic#115	B. melitensis bv3	43	125		BRU-S115	Human	2005	Diyarbakir, Turkey
1	5	3	13	3	2	3	2	4	41	8	5	4	3	5	4	2011Kilic#138	B. melitensis bv3	43	125		BRU-S138	Human	2003	Malatya, Turkey
1	5	3	13	3	2	3	2	4	41	8	5	4	3	5	4	2018Vergnaud#0427	B. melitensis bv3	43	125		BCCN#96-108	Human	1996	Iraq
1	5	3	13	3	2	3	2	4	41	8	5	4	3	5	4	SRR4436626	B. melitensis	43	125		BwIM_JOR_13	Human	2017	Jordan
1	5	3	13	3	2	3	2	4	41	8	5	4	3	5	4	SRR4436627	B. melitensis	43	125	38	BwIM_IRN_37	Human	2017	Iran
1	5	3	13	3	2	3	2	4	41	8	8	4	3	5	4	2011Kilic#057	B. melitensis bv3	43	125		BRU-S057	Human	2008	Kutahya, Turkey
1	5	3	13	3	2	3	2	4	41	8	4	5	3	5	4	2011Kilic#029	B. melitensis bv3	43	125		BRU-S029	Human	2007	Agri, Turkey
1	5	3	13	3	2	3	2	4	41	8	4	5	3	5	4	2011Kilic#116	B. melitensis bv3	43	125		BRU-S116	Human	2005	Diyarbakir, Turkey
1	5	3	13	3	2	3	2	4	41	8	4	5	3	5	4	2011Kilic#162	B. melitensis bv3	43	125		BRU-S162	Human	2001	Malatya, Turkey
1	5	3	13	3	2	3	2	4	41	8	4	5	3	5	4	2018Vergnaud#0042	B. melitensis bv1	43	125		BCCN#00-28	Human	2000	Bas-Rhin, France
1	5	3	13	3	2	3	2	4	41	8	4	5	3	5	4	2018Vergnaud#0050	B. melitensis bv1	43	125		BCCN#88-43	Human	1988	Israel
1	5	3	13	3	2	3	2	4	41	8	4	5	3	5	4	2018Vergnaud#0106	B. melitensis bv1	43	125		BCCN#88-39	Ovine	1988	Israel
1	5	3	13	3	2	3	2	4	41	8	4	5	3	5	4	2018Vergnaud#0437	B. melitensis bv1	43	125		BCCN#76-280	Cattle	1976	Israel
1	5	3	13	3	2	3	2	4	41	8	4	5	3	5	4	SRR4436570	B. melitensis	43	125		BwIM_SYR_06	Human	2017	Syria
1	5	3	13	3	2	3	2	4	41	8	4	4	3	5	4	2008Kattar#020	B. melitensis	43	125		AUB BRUP-S31	Human		Lebanon
1	5	3	13	3	2	3	2	4	41	8	4	4	3	5	4	2016Gyuranecz#013	B. melitensis	43	125		C13	Cattle	2015	United Arab Emirates
1	5	3	13	3	2	3	2	4	41	8	4	4	3	5	4	2016Gyuranecz#014	B. melitensis	43	125		C14	Cattle	2015	United Arab Emirates
1	5	3	13	3	2	3	2	4	41	8	4	4	3	5	4	2017Hanot-Mamb.	B. melitensis bv3	43	125		L3/149	Human	2007	Belgium
1	5	3	13	3	2	3	2	4	41	8	4	4	3	5	4	2018Vergnaud#0959	B. melitensis bv3	43	125		BCCN#01-55	Human	2001	France
1	5	3	13	3	2	3	2	4	41	8	4	4	3	5	4	SRR4436584	B. melitensis	43	125	38	BwIM_SYR_07	Human	2017	Syria
1	5	3	13	3	2	3	2	4	41	8	4	4	3	5	4	SRR4436611	B. melitensis	43	125	38	BwIM_TUR_40	Human	2017	Turkey
1	5	3	13	3	2	3	2	4	41	8	4	4	3	5	4	SRR4436624	B. melitensis	43	125	38	BwIM_XXX_22	Human	2017	
1	5	3	13	3	2	3	2	4	41	8	4	7	3	5	4	2007AlDahouk#107	B. melitensis bv3	43	125		BfR 86	Human	1998	Gelsenkirchen, Germany
1	5	3	13	3	2	2	2	4	41	8	4	4	3	5	4	2008Kattar#014	B. melitensis	60	118		AUB BRUP-S24	Human		Lebanon
1	5	3	13	3	2	3	2	4	41	8	8	3	3	5	4	2008Kattar#022	B. melitensis	43	125		AUB BRUP-S33	Human		Lebanon
1	5	3	13	3	2	3	2	4	41	8	5	3	3	5	4	2011Kilic#152	B. melitensis bv3	43	125		BRU-S152	Human	2005	Malatya, Turkey
1	5	3	13	3	2	3	2	4	41	8	6	6	3	5	4	2011Kilic#007	B. melitensis bv3	43	125		BRU-S007	Human	2004	Adana, Turkey
1	5	3	13	3	2	3	2	4	41	8	6	6	3	5	4	2011Kilic#160	B. melitensis bv3	43	125		BRU-S160	Human	2004	Malatya, Turkey
1	5	3	13	3	2	3	2	4	41	8	6	5	3	5	4	2007AlDahouk#087	B. melitensis bv3	43	125		BfR 63	Human	2001	Greece
1	5	3	13	3	2	3	2	4	41	8	6	5	3	5	4	2011Kilic#129	B. melitensis bv3	43	125		BRU-S129	Human	2004	Adiyaman, Turkey
1	5	3	13	3	2	3	2	4	41	8	6	5	3	5	4	2011Kilic#139	B. melitensis bv3	43	125		BRU-S139	Human	2003	Malatya, Turkey
1	5	3	13	3	2	3	2	4	41	8	6	3	3	5	4	2007AlDahouk#035	B. melitensis bv1	43	125		BfR 11	Human	2002	Turkey
1	5	3	13	3	2	3	2	4	41	8	6	3	3	5	4	2011Kilic#161	B. melitensis bv3	43	125		BRU-S161	Human	2001	Malatya, Turkey
1	5	3	13	3	2	3	2	4	41	8	5	5	3	8	4	SRR4436590	B. melitensis	43	125		BwIM_TUR_24	Human	2017	Turkey
1	5	3	13	3	2	3	2	4	41	8	5	5	3	8	4	SRR4436610	B. melitensis	43	125		BwIM_TUR_60	Human	2017	Turkey
1	5	3	13	3	2	3	2	4	41	8	5	5	3	6	4	SRR4436587	B. melitensis	43	125		BwIM_TUR_39	Human	2017	Turkey
1	5	3	13	3	2	3	2	4	41	8	5	5	3	6	4	SRR4436609	B. melitensis	43	125		BwIM_TUR_59	Human	2017	Turkey
1	5	3	13	3	2	3	2	4	41	8	5	5	3	3	4	2017Hanot-Mamb.	B. melitensis bv3	43	125		L3/203	Human	2011	Belgium
1	5	3	13	3	2	3	2	4	41	8	5	5	3	3	4	2017Hanot-Mamb.	B. melitensis bv3	43	125		L3/204	Human	2011	Belgium
1	5	3	13	3	2	3	2	4	41	8	5	5	3	5	4	2006LeFleche#080	B. melitensis bv1	43	125		BCCN#75-478	Ovine	1975	Israel
1	5	3	13	3	2	3	2	4	41	8	5	5	3	5	4	2011Kilic#126	B. melitensis bv3	43	125		BRU-S126	Human	2004	Adiyaman, Turkey
1	5	3	13	3	2	3	2	4	41	8	5	5	3	5	4	2011Kilic#127	B. melitensis bv3	43	125		BRU-S127	Human	2004	Adiyaman, Turkey
1	5	3	13	3	2	3	2	4	41	8	5	5	3	5	4	2018Vergnaud#0034	B. melitensis bv1	43	125		BCCN#88-40	Ovine	1988	Israel
1	5	3	13	3	2	3	2	4	41	8	5	5	3	5	4	2018Vergnaud#0438	B. melitensis bv1	43	125		BCCN#76-281	Cattle	1976	Israel
1	5	3	13	3	2	3	2	4	41	8	5	5	3	4	4	2007AlDahouk#026	B. melitensis bv2	43	125		BfR 1	Human	2002	Turkey
1	5	3	13	3	2	3	2	4	43	8	5	5	3	5	4	2007AlDahouk#091	B. melitensis bv3	43	122		BfR 67	Human	1998	Turkey
1	5	3	13	3	2	3	2	4	41	8	7	5	3	5	4	2008Kattar#015	B. melitensis	43	125		AUB BRUP-S26	Human		Lebanon
1	5	3	13	3	2	3	2	4	41	8	7	5	3	4	4	2011Kilic#125	B. melitensis bv3	43	125		BRU-S125	Human	2007	Sirnak, Turkey
1	5	3	13	3	2	3	2	4	36	8	7	4	3	8	8	2011Kilic#100	B. melitensis bv3	43	121		BRU-S100	Human	2008	Istanbul, Turkey
1	5	3	13	3	2	3	2	4	41	8	7	4	3	8	8	2018Vergnaud#0385	B. melitensis bv3	43	125		BCCN#83-165	Ovine	1983	Greece
1	5	3	13	3	2	3	2	4	41	8	7	4	3	7	8	2007AlDahouk#046	B. melitensis bv2	43	125		BfR X	Human	2003	Bosnia
1	5	3	13	3	2	3	2	4	41	8	7	4	3	7	8	2018Vergnaud#0386	B. melitensis bv3	43	125		BCCN#83-166	Ovine	1983	Greece
1	5	3	13	3	2	3	2	4	41	8	8	4	3	7	8	SRR4436575	B. melitensis	43	125	8	BwIM_XXX_12	Human	2017	
1	5	3	13	2	2	3	2	4	41	8	6	4	3	6	8	2017Liu#109	B. melitensis	42	116		WS108	Human	2015	Zhuozi, China
1	5	3	13	2	2	3	2	4	41	8	6	4	3	10	8	2018Vergnaud#1101	B. melitensis bv3	42	116		BCCN#99-95	Human	1999	Hérault, France
1	5	3	13	3	2	3	2	4	41	8	6	4	3	6	8	2011Kilic#018	B. melitensis bv3	43	125		BRU-S018	Human	2004	Erzurum, Turkey
1	5	3	13	3	2	3	2	4	41	8	6	4	3	6	8	2018Vergnaud#0535	B. melitensis bv3	43	125		BCCN#83-144	Ovine	1983	Greece
1	5	3	13	3	2	3	2	4	41	8	6	4	3	6	8	2018Vergnaud#1303	B. melitensis bv3	43	125		BCCN#83-187	Ovine	1983	Greece
1	5	3	13	3	2	3	2	4	41	8	6	4	3	8	8	2018Vergnaud#0580	B. melitensis bv3	43	125		BCCN#90-160	Cattle	1990	Salonique ,Greece
1	5	3	13	3	2	3	2	4	41	8	6	4	3	5	8	2011Kilic#107	B. melitensis bv3	43	125		BRU-S107	Human	2005	Diyarbakir, Turkey
1	5	3	13	3	2	3	2	4	41	8	6	5	3	5	5	2007AlDahouk#034	B. melitensis bv2	43	125		BfR 10	Human	2002	Turkey
1	5	3	13	3	2	3	2	4	41	8	6	5	3	5	5	SRR4436634	B. melitensis	43	125	8	BwIM_IRQ_49	Human	2017	Iraq
1	5	3	13	3	2	3	2	4	41	8	3	5	3	5	5	2007AlDahouk#066	B. melitensis bv2	43	125		BfR 33	Human	2001	Ulm, Germany
1	5	3	13	3	2	3	2	6	41	8	3	5	3	5	5	2018Vergnaud#0051	B. melitensis bv1	43	284		BCCN#88-44	Human	1988	Israel
1	5	3	13	3	2	3	2	4	41	8	4	5	3	6	5	2007AlDahouk#011	B. melitensis bv2	43	125		BCCN#96-30	Human	1996	Israel
1	5	3	13	3	2	3	2	4	41	8	7	5	3	6	5	2018Vergnaud#0405	B. melitensis bv3	43	125		BCCN#91-131	Cattle	1991	Salonique ,Greece
1	5	3	13	3	2	3	2	4	41	8	6	5	3	6	5	2011Kilic#012	B. melitensis bv3	43	125		BRU-S012	Human	2004	Mersin, Turkey
1	5	3	13	3	2	3	2	4	41	8	4	5	3	6	4	2015Shevtsov#126	B. melitensis	43	125		SH-3	Ovine	2006	Bozaryk,Sayram,South.
1	5	3	13	3	2	3	2	4	41	8	4	5	3	6	4	2015Shevtsov#177	B. melitensis	43	125		ZHT	Ovine	2007	Burnoye,Zhualy,Zhamby.
1	5	3	13	3	2	3	2	4	41	8	6	5	3	6	4	2011Kilic#147	B. melitensis bv3	43	125		BRU-S147	Human	2004	Malatya, Turkey
1	5	3	13	3	2	3	2	4	41	8	6	5	3	6	4	2011Kilic#153	B. melitensis bv3	43	125		BRU-S153	Human	2004	Malatya, Turkey
1	5	3	13	3	2	3	2	4	43	8	4	4	3	5	4	2006LeFleche#133	B. melitensis bv2	43	122		BfR 51	Ovine	1990	Meskane, Syria
1	5	3	13	3	2	3	2	4	43	8	4	5	3	5	4	2007AlDahouk#077	B. melitensis bv2	43	122		BfR 52	Human	2000	Bern, Switzerland
1	5	3	13	3	2	3	2	4	43	8	4	5	3	6	4	2007AlDahouk#079	B. melitensis bv2	43	122		BfR 54	Human	2001	Bern, Switzerland

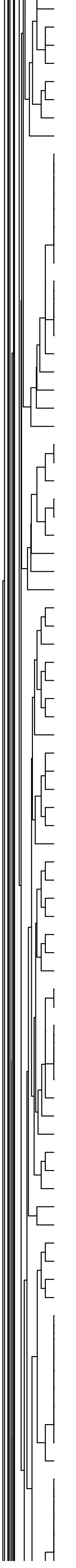

1	5	3	13	3	2	3	2	4	43	8	7	4	3	5	4	2007AlDahouk#090	B. melitensis bv3	43	122		BfR 66	Human	1997	Bonn, Germany
1	5	3	13	3	2	3	2	4	43	8	5	4	3	6	4	2007AlDahouk#074	B. melitensis bv2	43	122		BfR 42	Human	2002	
1	5	3	13	3	2	3	2	4	43	8	4	4	3	6	4	2007AlDahouk#100	B. melitensis bv3	43	122		BfR 79	Human	1998	Heidelberg, Germany
1	5	3	13	3	2	3	2	4	43	8	6	4	3	6	4	2007AlDahouk#098	B. melitensis bv3	43	122		BfR 75	Human	1998	Turkey
1	5	3	13	3	2	3	2	4	43	8	5	4	3	4	5	2007AlDahouk#073	B. melitensis bv2	43	122		BfR 40	Human	2002	Munchen, Germany
1	5	3	13	3	2	3	2	4	43	8	5	4	3	4	4	2007AlDahouk#101	B. melitensis bv3	43	122		BfR 80	Human	1998	Turkey
1	5	3	13	3	2	3	2	4	43	8	2	4	3	4	4	2007AlDahouk#075	B. melitensis bv2	43	122		BfR 45	Human	1999	
1	5	3	13	3	2	3	2	4	46	8	8	4	3	6	4	SRR4436620	B. melitensis	43	374	38	BwIM_SYR_33	Human	2017	Syria
1	5	3	13	3	2	3	2	5	41	8	4	4	3	4	5	2018Vergnaud#0115	B. melitensis bv3	43	106		BCCN#83-157	Ovine	1983	Greece
1	5	3	13	3	2	3	2	5	41	8	4	4	3	4	5	2018Vergnaud#0822	B. melitensis bv3	43	106		BCCN#83-149	Ovine	1983	Greece
1	5	3	13	3	2	3	2	5	41	8	4	4	3	4	5	2018Vergnaud#0899	B. melitensis bv3	43	106		BCCN#83-145	Ovine	1983	Greece
1	5	3	13	3	2	3	2	5	41	8	4	4	3	4	5	2018Vergnaud#0930	B. melitensis bv3	43	106		BCCN#83-152	Ovine	1983	Greece
1	5	3	13	3	2	3	2	5	41	8	4	4	3	4	5	2018Vergnaud#1151	B. melitensis bv3	43	106		BCCN#83-153	Ovine	1983	Greece
1	5	3	13	3	2	3	2	5	41	8	4	4	3	4	5	2018Vergnaud#1152	B. melitensis bv3	43	106		BCCN#83-155	Ovine	1983	Greece
1	5	3	13	3	2	3	2	5	41	8	4	4	3	4	5	2018Vergnaud#1153	B. melitensis bv3	43	106		BCCN#83-156	Ovine	1983	Greece
1	5	3	13	3	2	3	2	5	41	8	4	4	3	4	4	2011Kilic#036	B. melitensis bv3	43	106		BRU-S036	Human	2007	Bayburt, Turkey
1	5	3	13	3	2	3	2	5	41	8	4	4	3	4	4	2018Vergnaud#0384	B. melitensis bv3	43	106		BCCN#83-164	Caprine	1983	Greece
1	5	3	13	3	2	3	2	5	41	8	4	4	3	4	4	2018Vergnaud#0836	B. melitensis bv3	43	106		BCCN#91-140	Ovine	1991	Salonique ,Greece
1	5	3	13	3	2	3	2	5	41	8	4	4	3	4	4	2018Vergnaud#0976	B. melitensis bv3	43	106		BCCN#91-142	Ovine	1991	Salonique ,Greece
1	5	3	13	3	2	3	2	5	41	8	4	4	3	4	7	2018Vergnaud#0461	B. melitensis bv1	43	106		BCCN#90-84	Ovine	1990	Somme, France
1	5	3	13	3	2	3	2	5	41	8	4	6	3	4	4	2018Vergnaud#0406	B. melitensis bv3	43	106		BCCN#91-139	Ovine	1991	Salonique ,Greece
1	5	3	13	3	2	3	2	5	41	8	4	7	3	4	6	2018Vergnaud#0433	B. melitensis bv3	43	106		BCCN#91-152	Caprine	1991	Salonique ,Greece
1	5	3	13	3	2	3	2	5	41	8	4	4	6	4	6	2018Vergnaud#0376	B. melitensis bv3	43	106		BCCN#04-12	Human	2004	Turkey
1	5	3	13	3	2	3	2	5	41	8	8	4	3	8	5	2011Kilic#101	B. melitensis bv3	43	106		BRU-S101	Human	2008	Istanbul, Turkey
1	5	3	13	3	3	3	2	4	41	8	6	4	3	6	4	2007AlDahouk#085	B. melitensis bv3	57	112		BfR 61	Human	2001	Turkey
1	5	3	13	3	3	3	2	4	41	8	6	4	3	6	4	SRR4436586	B. melitensis	57	112	38	BwIM_SYR_31	Human	2017	Syria
1	5	3	13	2	3	3	2	4	41	8	6	4	3	6	4	SRR4436605	B. melitensis	63	111		BwIM_XXX_62	Human	2017	
1	5	3	13	3	3	3	2	4	41	8	5	4	3	8	4	2008Kattar#024	B. melitensis	57	112		AUB BRUP-S36	Human		Lebanon
1	5	3	13	3	3	3	2	4	41	8	5	4	3	8	4	SRR4436616	B. melitensis	57	112	38	BwIM_SYR_23	Human	2017	Syria
1	5	3	13	3	3	3	2	4	41	8	5	4	3	6	4	SRR4436577	B. melitensis	57	112	38	BwIM_SYR_05	Human	2017	Syria
1	5	3	13	3	3	3	2	4	41	8	5	4	3	7	8	2007AlDahouk#058	B. melitensis bv1	57	112		BfR 18	Human	2000	Turkey
1	5	3	13	3	3	3	2	4	41	8	5	7	3	5	4	2011Aftab#001	B. melitensis	57	112		REF 665-99	Human		Turkey
1	5	3	13	2	3	3	2	4	41	8	9	4	3	9	4	SRR4436613	B. melitensis	63	111	8	BwIM_AFG_63	Human	2017	Afghanistan
1	5	3	13	3	2	3	2	4	41	8	6	4	6	4	3	2006LeFleche#094	B. melitensis bv3	43	125		BCCN#90-112	Cattle	1990	Greece
1	5	3	13	3	2	3	2	4	41	8	6	4	6	5	3	2007AlDahouk#052	B. melitensis bv2	43	125		BfR 5	Human	2004	Dusseldorf, Germany
1	5	3	13	3	2	3	2	4	41	8	6	4	11	5	3	2018Vergnaud#0128	B. melitensis bv3	43	125		BCCN#90-127	Cattle	1990	Salonique ,Greece
1	5	3	13	3	2	3	2	4	41	8	7	4	8	5	3	2018Vergnaud#0534	B. melitensis bv3	43	125		BCCN#83-143	Ovine	1983	Greece
1	5	3	13	3	2	3	2	4	41	8	5	4	8	5	3	2018Vergnaud#0587	B. melitensis bv3	43	125		BCCN#90-37	Human	1990	Val-de-Marne, France
1	5	3	13	3	2	3	2	4	41	8	6	4	8	6	3	2007AlDahouk#108	B. melitensis bv3	43	125		BfR 87	Human	1998	Ulm, Germany
1	5	3	13	3	2	3	2	4	41	8	6	4	8	5	3	2011Kilic#005	B. melitensis bv3	43	125		BRU-S005	Human	2004	Hatay, Turkey
1	5	3	13	3	2	3	2	4	41	8	3	4	5	5	3	2011Kilic#006	B. melitensis bv3	43	125		BRU-S006	Human	2004	Osmaniye, Turkey
1	5	3	13	3	2	3	2	4	41	8	6	4	3	7	3	2008Kattar#021	B. melitensis	43	125		AUB BRUP-S32	Human		Lebanon
1	5	3	13	3	2	3	2	4	41	8	6	4	7	7	3	2018Vergnaud#0823	B. melitensis bv3	43	125		BCCN#83-150	Ovine	1983	Greece
1	5	3	13	3	2	3	2	4	41	8	6	4	9	7	3	2011Kilic#144	B. melitensis bv3	43	125		BRU-S144	Human	2004	Malatya, Turkey
1	5	3	13	3	2	3	2	4	41	8	6	4	4	7	3	2006LeFleche#131	B. melitensis bv2	43	125		BfR 46	unknown	1999	Bonn, Germany
1	5	3	13	3	2	3	2	4	43	8	6	4	4	7	3	2007AlDahouk#076	B. melitensis bv2	43	122		BfR 47	Human	1999	Turkey
1	5	3	13	3	2	3	2	4	41	8	6	4	14	9	3	2011Kilic#008	B. melitensis bv3	43	125		BRU-S008	Human	2004	Mersin, Turkey
1	5	3	13	3	2	3	2	4	41	8	5	4	6	9	3	2007AlDahouk#084	B. melitensis bv3	43	125		BfR 60	Human	2001	Turkey
1	5	3	13	3	2	3	2	4	41	8	5	4	6	5	3	2018Vergnaud#1302	B. melitensis bv3	43	125		BCCN#83-186	Ovine	1983	Greece
1	5	3	13	3	2	3	2	4	41	8	5	4	7	6	3	2007AlDahouk#048	B. melitensis bv2	43	125		BfR XIV	Human	2003	
1	5	3	13	3	2	3	2	4	41	8	5	4	6	6	3	2007AlDahouk#106	B. melitensis bv3	43	125		BfR 85	Human	1998	Turkey
1	5	3	13	3	2	3	2	4	41	8	6	4	7	4	3	2011Kilic#011	B. melitensis bv3	43	125		BRU-S011	Human	2004	Adana, Turkey
1	5	3	13	3	2	3	2	4	41	8	5	4	7	4	3	2017Hanot-Mamb.	B. melitensis bv3	43	125		L3/130	Human	2001	Belgium
1	5	3	13	3	2	3	2	4	41	8	5	4	7	5	3	2018Vergnaud#0778	B. melitensis bv3	43	125		BCCN#83-189	Ovine	1983	Greece
1	5	3	13	3	2	3	2	4	41	8	5	4	7	7	3	2018Vergnaud#0900	B. melitensis bv3	43	125		BCCN#83-147	Ovine	1983	Greece
1	5	3	13	3	2	3	2	4	41	8	5	4	7	7	3	2018Vergnaud#0929	B. melitensis bv3	43	125		BCCN#83-151	Ovine	1983	Greece
1	5	3	13	3	2	3	2	4	41	8	5	4	5	7	3	2011Kilic#094	B. melitensis bv3	43	125		BRU-S094	Human	2005	Zonguldak, Turkey
1	5	3	13	3	2	3	2	4	41	8	5	4	5	7	3	2011Kilic#095	B. melitensis bv3	43	125		BRU-S095	Human	2005	Zonguldak, Turkey
1	5	3	13	3	2	3	2	4	41	8	5	4	5	7	3	2011Kilic#096	B. melitensis bv3	43	125		BRU-S096	Human	2005	Zonguldak, Turkey
1	5	3	13	3	2	3	2	4	41	8	5	4	5	7	3	2011Kilic#097	B. melitensis bv3	43	125		BRU-S097	Human	2005	Zonguldak, Turkey
1	5	3	13	3	2	3	2	4	41	8	5	4	9	7	3	SRR4436637	B. melitensis	43	125	8	BwIM_TUR_03	Human	2017	Turkey
1	5	3	13	3	2	3	2	4	43	8	5	4	5	7	3	2007AlDahouk#095	B. melitensis bv3	43	122		BfR 72	Human	1999	Turkey
1	5	3	13	3	2	3	2	4	41	8	7	4	7	7	3	2018Vergnaud#0965	B. melitensis bv3	43	125		BCCN#83-175	Ovine	1983	Greece
1	5	3	13	3	2	3	2	4	41	8	5	4	9	11	3	2011Kilic#041	B. melitensis bv3	43	125		BRU-S041	Human	2008	Erzurum, Turkey
1	5	3	13	3	2	3	2	4	41	8	5	4	9	5	3	2018Vergnaud#0777	B. melitensis bv3	43	125		BCCN#83-174	Ovine	1983	Greece
1	5	3	13	3	2	3	2	4	41	8	4	4	9	5	3	2011Kilic#070	B. melitensis bv3	43	125		BRU-S070	Human	2003	Kirikkale, Turkey
1	5	3	13	3	2	3	2	4	41	8	4	4	6	7	3	2007AlDahouk#089	B. melitensis bv3	43	125		BfR 65	Human	1997	
1	5	3	13	1	2	3	2	4	41	8	4	4	3	7	3	2011Kilic#111	B. melitensis bv3	84	113		BRU-S111	Human	2003	Diyarbakir, Turkey
1	5	3	13	3	2	3	2	4	41	8	9	4	7	6	3	2018Vergnaud#0827	B. melitensis bv3	43	125		BCCN#83-179	Ovine	1983	Greece
1	5	3	13	3	2	3	2	4	41	8	9	4	10	6	3	2018Vergnaud#0967	B. melitensis bv3	43	125		BCCN#83-177	Ovine	1983	Greece
1	5	3	13	3	2	3	2	4	41	8	2	4	3	6	3	2008Kattar#010	B. melitensis	43	125		AUB BRUP-S20	Human		Lebanon
1	5	3	13	3	2	3	2	4	41	8	9	4	3	6	3	2018Vergnaud#0826	B. melitensis bv3	43	125		BCCN#83-178	Ovine	1983	Greece
1	5	3	13	3	2	3	2	4	41	8	7	4	9	6	3	2018Vergnaud#0837	B. melitensis bv3	43	125		BCCN#91-141	Ovine	1991	Salonique ,Greece
1	5	3	13	3	2	3	2	4	41	8	7	4	9	6	3	2018Vergnaud#0838	B. melitensis bv3	43	125		BCCN#91-149	Ovine	1991	Salonique ,Greece
1	5	3	13	3	2	3	2	4	41	8	7	4	9	6	3	2018Vergnaud#0839	B. melitensis bv3	43	125		BCCN#91-150	Ovine	1991	Salonique ,Greece
1	5	3	13	3	2	3	2	4	41	8	7	4	9	6	3	2018Vergnaud#0977	B. melitensis bv3	43	125		BCCN#91-144	Ovine	1991	Salonique ,Greece
1	5	3	13	3	2	3	2	4	41	8	7	4	9	6	3	2018Vergnaud#0978	B. melitensis bv3	43	125		BCCN#91-145	Ovine	1991	Salonique ,Greece
1	5	3	13	3	2	3	2	4	41	8	7	4	9	6	3	2018Vergnaud#1163	B. melitensis bv3	43	125		BCCN#91-146	Ovine	1991	Salonique ,Greece
1	5	3	13	3	2	3	2	4	41	8	7	4	9	6	3	2018Vergnaud#1165	B. melitensis bv3	43	125		BCCN#91-147	Ovine	1991	Salonique ,Greece
1	5	3	13	3	2	3	2	4	41	8	7	4	9	6	3	2018Vergnaud#1166	B. melitensis bv3	43	125		BCCN#91-148	Ovine	1991	Salonique ,Greece
1	5	3	13	3	2	3	2	4	41	8	7	4	10	6	3	2018Vergnaud#0594	B. melitensis bv3	43	125		BCCN#91-151	Ovine	1991	Salonique ,Greece
1	5	3	13	3	2	3	2	4	41	8	6	4	9	6	5	SRR4436568	B. melitensis	43	125	8	BwIM_TUR_20d	Human		Turkey
1	5	3	13	3	2	3	2	4	41	8	6	4	9	6	5	SRR4436569	B. melitensis	43	125	8	BwIM_TUR_20a	Human		Turkey
1	5	3	13	3	2	3	2	4	41	8	6	4	9	6	5	SRR4436573	B. melitensis	43	125	8	BwIM_TUR_20b	Human		Turkey
1	5	3	13	3	2	3	2	4	41	8	6	4	9	6	5	SRR4436628	B. melitensis	43	125	8	BwIM_TUR_20e	Human		Turkey
1	5	3	13	3	2	3	2	4	41	8	6	4	9	6	5	SRR4436638	B. melitensis	43	125	8	BwIM_TUR_20c	Human		Turkey

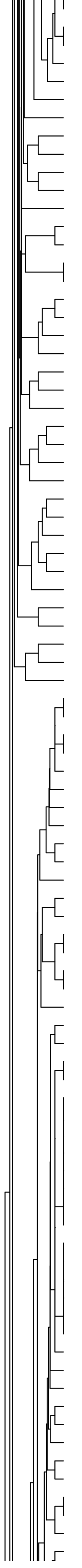

1	5	3	13	3	2	3	2	4	41	8	6	4	9	6	5	SRR4436638_SR.	B. melitensis	43	125	8			2017	
1	5	3	13	3	2	3	2	4	41	8	6	4	3	6	5	2008Kattar#019	B. melitensis	43	125		AUB BRUP-S30	Human		Lebanon
1	5	3	13	3	2	3	2	4	41	8	6	4	3	6	5	2018Vergnaud#0834	B. melitensis bv3	43	125		BCCN#91-110	Cattle	1991	Salonique ,Greece
1	5	3	13	3	2	3	2	4	41	8	9	4	9	6	5	2018Vergnaud#0390	B. melitensis bv2	43	125		BCCN#03-35	Human	2003	Turkey
1	5	3	13	3	2	3	2	4	41	8	6	3	3	6	5	2018Vergnaud#0831	B. melitensis bv3	43	125		BCCN#91-103	Cattle	1991	Salonique ,Greece
1	5	3	13	3	2	3	2	4	41	8	6	5	9	8	3	2011Kilic#151	B. melitensis bv3	43	125		BRU-S151	Human	2005	Malatya, Turkey
1	5	3	13	2	2	3	3	4	41	8	4	11	3	7	5	2017Hanot-Mamb.	B. melitensis bv3	184	362		L3/23	Human	1998	Belgium
1	5	3	13	2	2	3	2	5	41	8	5	7	9	5	5	2018Vergnaud#0373	B. melitensis bv3	42	180		BCCN#00-21	Human	2000	Switzerland
1	5	3	13	2	2	3	2	5	41	8	4	7	3	11	5	2018Vergnaud#0545	B. melitensis bv3	42	180		BCCN#00-10	Human	2000	Gironde, France
1	5	3	13	2	2	3	2	4	41	6	6	7	6	5	5	2018Vergnaud#0010	B. melitensis bv2	42	294		BCCN#76-356	Human	1976	Montpellier ,Hérault, Fr.
1	5	3	13	2	2	3	2	4	41	8	6	7	10	6	5	2018Vergnaud#0811	B. melitensis bv3	42	116		BCCN#75-130	Human	1975	Castille, Spain
1	5	3	13	2	1	3	2	4	46	8	8	4	3	5	5	2017Liu#014	B. melitensis	114	344		WS013	Human	2014	Xinghe, China
1	5	3	12	2	2	3	2	4	41	8	7	4	2	4	4	2017Liu#125	B. melitensis bv3	45	115		NM-22	Ovine	2016	
1	5	3	12	2	2	3	2	4	41	8	7	3	2	4	4	2017Liu#126	B. melitensis bv3	45	115		M2	Ovine	2016	
1	5	3	12	2	2	3	2	4	41	8	6	4	3	3	3	2017Liu#127	B. melitensis bv3	45	115		061-2	Ovine	2015	
1	5	3	12	2	2	3	2	4	41	8	6	4	3	3	3	2017Liu#128	B. melitensis bv3	45	115		061-3	Ovine	2015	
1	5	3	13	3	2	3	2	4	41	8	5	5	8	7	9	2008Kattar#026	B. melitensis	43	125		AUB BRUP-S40	Human		Lebanon
1	5	3	13	3	2	3	2	4	41	8	5	5	8	7	10	2018Vergnaud#0214	B. melitensis R.	43	125		BCCN#93-41	Human	1993	Palestine Cis Jordanie,.
1	5	3	13	3	2	3	2	4	41	8	5	4	8	6	9	2007AlDahouk#006	B. melitensis bv1	43	125		BCCN#94-37	Human	1994	Paris, France
1	5	3	13	3	2	3	2	4	41	8	5	5	9	6	6	2008Kattar#006	B. melitensis	43	125		AUB BRUP-S14	Human		Lebanon
1	5	3	13	2	2	3	2	4	43	8	8	7	3	3	5	2007AlDahouk#070	B. melitensis bv2	42	108		BfR 37	Human	1997	Turkey
1	5	3	13	2	2	3	2	4	40	8	9	7	3	7	5	2017Hanot-Mamb.	B. melitensis bv3	42	368		L3/311	Human	2015	Belgium
1	5	3	13	2	2	3	2	6	43	8	3	6	3	3	5	2013Jiang#053	B. melitensis bv1	42	279		NM92-0613	Ovine	1992	Inner Mongolia, China
1	3	3	13	2	2	3	2	4	41	8	4	5	3	4	4	2011Kilic#089	B. melitensis bv3	62	114		BRU-S089	Human	2007	Trabzon, Turkey
1	3	3	13	2	2	3	2	4	41	8	5	5	3	5	4	2011Kilic#091	B. melitensis bv3	62	114		BRU-S091	Human	2008	Bayburt, Turkey
1	3	3	13	2	2	3	2	4	41	8	7	5	3	6	7	2007AlDahouk#053	B. melitensis bv1	62	114		BfR 12	Human	1999	Turkey
1	3	3	13	3	2	3	3	4	41	8	5	5	3	3	4	2017Hanot-Mamb.	B. melitensis bv3	186	364		L3/205	Human	2012	Belgium
1	5	3	13	3	1	3	2	4	41	8	4	4	3	5	6	2007AlDahouk#040	B. melitensis bv1	58	120		BfR I	Human	2003	Turkey
1	5	3	13	3	4	3	2	4	41	8	4	4	3	8	6	2011Kilic#137	B. melitensis bv3	85	119		BRU-S137	Human	2004	Malatya, Turkey
1	5	3	13	3	1	3	2	4	41	8	4	4	9	4	6	2011Jiang#055	B. melitensis bv3	58	120		FJ09-3	Human	2009	Fujian, China
1	5	3	13	3	1	3	2	4	41	8	4	4	6	8	3	2011Jiang#058	B. melitensis bv3	58	120		YN09-H	Human	2009	Yunnan, China
1	5	3	13	3	1	3	2	4	41	8	4	4	8	5	3	2011Jiang#059	B. melitensis bv3	58	120		YN09-17	Human	2009	Yunnan, China
1	5	3	13	3	2	3	2	4	49	8	4	4	9	5	6	2008Kattar#002	B. melitensis	43	124		AUB BRUP-S10	Human		Lebanon
1	5	3	12	3	2	3	2	5	41	8	7	4	3	7	5	2007AlDahouk#105	B. melitensis bv3	64	101		BfR 84	Human	1998	Wurzburg, Germany
1	5	3	12	3	2	3	2	8	41	8	5	4	3	7	4	2016Gyuranecz#011	B. melitensis	64	223		C11	Camel	2013	United Arab Emirates
1	5	3	13	3	2	3	2	5	41	8	5	5	7	5	6	2008Kattar#025	B. melitensis	43	106		AUB BRUP-S38	Human		Lebanon
1	5	3	13	3	2	3	2	5	9	8	6	5	3	5	6	2018Vergnaud#0024	B. melitensis bv2	43	105		BCCN#82-68	Human	1982	Barcelone ,Spain
1	5	3	14	2	2	3	2	5	41	8	5	6	3	5	6	2011Kilic#044	B. melitensis bv3	83	100		BRU-S044	Human	2008	Kars, Turkey
1	5	3	13	2	2	3	2	5	41	7	4	6	3	5	3	2010Valdezate#051	B. melitensis	42	166		#20010204	Human	2010	Madrid, Spain
1	5	3	13	2	2	3	2	5	41	7	4	6	3	5	3	2010Valdezate#052	B. melitensis	42	166		#9900113	Human	1999	Caceres, Spain
1	5	3	13	2	2	3	2	5	41	7	4	6	3	3	3	2010Valdezate#035	B. melitensis	42	166		#20030228	Human	2003	Madrid, Spain
1	5	3	13	2	2	3	2	5	41	7	4	6	3	3	3	2010Valdezate#036	B. melitensis	42	166		#20030273	Human	2003	Madrid, Spain
1	5	3	13	2	2	3	2	5	41	7	4	6	3	4	3	2010Valdezate#049	B. melitensis	42	166		#20020342	Human	2002	Badajoz, Spain
1	5	3	13	2	2	3	2	5	41	6	4	6	3	3	3	2010Valdezate#057	B. melitensis	42	165		#20030359	Human	2003	Madrid, Spain
1	5	3	13	2	2	3	2	5	41	7	2	6	3	3	3	2010Valdezate#034	B. melitensis	42	166		#20030198	Human	2003	Madrid, Spain
1	5	3	13	2	2	3	2	5	41	7	4	5	3	3	3	2010Valdezate#038	B. melitensis	42	166		#20040050	Human	2004	Badajoz, Spain
1	5	3	13	2	3	3	2	5	41	7	4	6	3	3	3	2010Valdezate#055	B. melitensis	63	167		#20030237	Human	2003	Madrid, Spain
1	5	3	13	2	3	3	2	5	41	7	4	6	3	7	3	2010Valdezate#061	B. melitensis	63	167		#20040318	Human	2004	Orense, Spain
1	5	3	13	2	2	3	2	5	41	7	4	7	3	3	6	2010Valdezate#045	B. melitensis	42	166		#20060454	Human	2006	Caceres, Spain
1	5	3	13	2	2	3	2	5	41	7	5	6	3	5	5	2010Valdezate#050	B. melitensis	42	166		#20040087	Human	2004	Caceres, Spain
1	5	3	13	2	2	3	2	5	41	7	3	6	3	5	5	2010Valdezate#054	B. melitensis	42	166		#20060152	Human	2006	Lugo, Spain
1	5	3	13	2	2	3	2	5	41	7	4	5	3	5	4	2010Valdezate#043	B. melitensis	42	166		#20050366	Human	2005	Leon, Spain
1	5	3	13	2	2	3	2	5	41	7	4	5	3	5	4	2010Valdezate#046	B. melitensis	42	166		#20070723	Human	2007	Burgos, Spain
1	5	3	13	2	2	3	2	5	41	7	4	6	3	5	4	2010Valdezate#031	B. melitensis	42	166		#20051102	Human	2005	Coru–a, Spain
1	5	3	13	2	2	3	2	5	41	7	4	6	3	5	4	2010Valdezate#053	B. melitensis	42	166		#9900115	Human	1999	Cordoba, Spain
1	5	3	13	2	2	3	2	5	41	6	4	5	3	5	5	2010Valdezate#059	B. melitensis	42	165		#9900053	Human	1999	Caceres, Spain
1	5	3	13	2	2	3	2	5	41	7	5	6	3	8	4	2010Valdezate#040	B. melitensis	42	166		#20040329	Human	2004	Soria, Spain
1	5	3	13	2	2	3	2	5	41	7	5	6	3	5	4	2010Valdezate#047	B. melitensis	42	166		#20000237	Human	2000	Guadalajara, Spain
1	5	3	13	2	2	3	2	5	41	7	3	6	3	3	4	2010Valdezate#018	B. melitensis	42	166		#20040220	Human	2004	Caceres, Spain
1	5	3	13	2	2	3	2	5	41	7	3	6	3	3	4	2010Valdezate#027	B. melitensis	42	166		#20070044	Human	2007	Ciudad Real, Spain
1	5	3	13	2	2	3	2	5	41	7	5	6	3	3	4	2010Valdezate#011	B. melitensis	42	166		#20040213	Human	2004	Caceres, Spain
1	5	3	13	2	2	3	2	5	41	7	5	6	3	3	4	2010Valdezate#012	B. melitensis	42	166		#9700018	Human	1997	Guipuzcoa, Spain
1	5	3	13	2	2	3	2	5	41	7	5	6	3	3	4	2010Valdezate#016	B. melitensis	42	166		#20000197	Human	2000	Cordoba, Spain
1	5	3	13	2	2	3	2	5	41	7	5	6	3	3	4	2010Valdezate#017	B. melitensis	42	166		#20040219	Human	2004	Caceres, Spain
1	5	3	13	2	2	3	2	5	41	7	5	6	3	3	4	2010Valdezate#019	B. melitensis	42	166		#20040297	Human	2004	Caceres, Spain
1	5	3	13	2	2	3	2	5	41	7	5	6	3	3	4	2010Valdezate#020	B. melitensis	42	166		#20040302	Human	2004	Caceres, Spain
1	5	3	13	2	2	3	2	5	41	7	5	6	3	3	4	2010Valdezate#024	B. melitensis	42	166		#20050861	Human	2005	Caceres, Spain
1	5	3	13	2	2	3	2	5	41	7	5	6	3	3	4	2010Valdezate#030	B. melitensis	42	166		#20081443	Human	2008	Leon, Spain
1	5	3	13	2	2	3	2	5	41	7	6	6	3	3	4	2010Valdezate#002	B. melitensis	42	166		#9900120	Human	1999	Cordoba, Spain
1	5	3	13	2	2	3	2	5	41	7	6	6	3	3	4	2010Valdezate#003	B. melitensis	42	166		#9900124	Human	1999	Cordoba, Spain
1	5	3	13	2	2	3	2	5	41	7	6	6	3	3	4	2010Valdezate#004	B. melitensis	42	166		#9900125	Human	1999	Cordoba, Spain
1	5	3	13	2	2	3	2	5	41	7	6	6	3	3	4	2010Valdezate#005	B. melitensis	42	166		#9900224	Human	1999	Cordoba, Spain
1	5	3	13	2	2	3	2	5	41	7	6	6	3	3	4	2010Valdezate#006	B. melitensis	42	166		#9900356	Human	1999	Cordoba, Spain
1	5	3	13	2	2	3	2	5	41	7	6	6	3	3	4	2010Valdezate#007	B. melitensis	42	166		#9900357	Human	1999	Cordoba, Spain
1	5	3	13	2	2	3	2	5	41	7	4	6	3	3	4	2010Valdezate#013	B. melitensis	42	166		#9900001	Human	1999	Almeria, Spain
1	5	3	13	2	2	3	2	5	41	7	5	6	4	3	4	2010Valdezate#039	B. melitensis	42	166		#20040299	Human	2004	Caceres, Spain
1	5	3	13	2	3	3	2	5	41	7	5	6	3	3	4	2010Valdezate#041	B. melitensis	63	167		#20040346	Human	2004	Madrid, Spain
1	5	3	13	2	2	3	2	5	41	7	5	5	3	3	4	2010Valdezate#023	B. melitensis	42	166		#20050801	Human	2005	Alicante, Spain
1	5	3	13	2	2	3	2	5	41	7	3	5	3	3	4	2010Valdezate#029	B. melitensis	42	166		#20080817	Human	2008	Lleida, Spain
1	5	3	13	2	2	3	2	5	41	7	5	8	3	3	4	2010Valdezate#025	B. melitensis	42	166		#20051047	Human	2005	Caceres, Spain
1	5	3	13	2	2	3	2	5	41	7	3	6	3	3	6	2010Valdezate#032	B. melitensis	42	166		#20010109	Human	2010	Madrid, Spain
1	5	3	13	2	2	3	2	5	41	7	5	6	3	3	6	2010Valdezate#044	B. melitensis	42	166		#20050606	Human	2005	Caceres, Spain
1	5	3	13	2	2	3	2	5	41	7	6	6	3	3	5	2010Valdezate#014	B. melitensis	42	166		#9900139	Human	1999	Cordoba, Spain
1	5	3	13	2	2	3	2	5	41	7	6	6	3	3	5	2010Valdezate#015	B. melitensis	42	166		#20000194	Human	2000	Cordoba, Spain
1	5	3	13	2	2	3	2	5	41	7	6	6	3	3	3	2010Valdezate#022	B. melitensis	42	166		#20050522	Human	2005	Caceres, Spain
1	5	3	13	2	2	3	2	5	41	7	6	2	3	3	4	2010Valdezate#001	B. melitensis	42	166		#9700017	Human	1997	Jaen, Spain

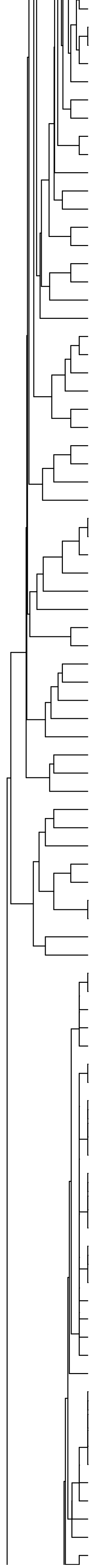

1	5	3	13	2	2	3	2	5	41	7	6	5	3	3	4	2010Valdezate#008	B. melitensis	42	166	#20000230	Human	2000	Madrid, Spain
1	5	3	13	2	2	3	2	5	41	7	6	4	3	3	4	2010Valdezate#009	B. melitensis	42	166	#20000472	Human	2000	Malaga, Spain
1	5	3	13	2	2	3	2	5	41	7	6	4	3	3	4	2010Valdezate#010	B. melitensis	42	166	#20040238	Human	2004	Teruel, Spain
1	5	3	13	2	2	3	2	5	41	7	6	4	3	7	4	2010Valdezate#021	B. melitensis	42	166	#20040337	Human	2004	Soria, Spain
1	5	3	13	2	2	3	2	3	41	7	6	5	3	3	4	2010Valdezate#028	B. melitensis	42	163	#20080676	Human	2008	Guadalajara, Spain
1	5	3	13	2	2	3	2	5	41	7	3	4	3	7	4	2010Valdezate#033	B. melitensis	42	166	#20030149	Human	2003	Malaga, Spain
1	5	3	13	2	2	3	2	5	41	7	3	4	3	3	5	2010Valdezate#042	B. melitensis	42	166	#20050201	Human	2005	Caceres, Spain
1	5	3	13	2	2	3	2	5	41	7	6	5	3	6	4	2010Valdezate#026	B. melitensis	42	166	#20061222	Human	2006	Madrid, Spain
1	5	3	13	2	2	3	2	5	41	7	6	5	12	6	4	2010Valdezate#037	B. melitensis	42	166	#20040036	Human	2004	Tarragona, Spain
1	5	3	13	2	2	3	2	6	41	7	6	4	3	3	5	2010Valdezate#048	B. melitensis	42	164	#20081716	Human	2008	Sevilla, Spain
1	5	3	13	2	2	3	2	5	41	6	4	6	3	6	6	2010Valdezate#060	B. melitensis	42	165	#9800003	Human	1998	Palencia, Spain
1	5	3	13	2	2	3	2	5	41	6	6	6	3	11	3	2018Vergnaud#1200	B. melitensis bv3	42	165	BCCN#90-183	Cattle	1990	Navarre, Spain
1	5	3	13	3	2	3	2	5	43	7	3	4	3	3	4	2010Valdezate#056	B. melitensis	43	170	#20070783	Human	2007	Valladolid, Spain
1	5	3	13	3	2	3	2	5	9	7	5	4	3	3	4	2010Valdezate#058	B. melitensis	43	172	#20000240	Human	2000	Guadalajara, Spain
1	5	3	13	3	2	3	2	5	9	7	4	3	3	4	4	2010Valdezate#062	B. melitensis	43	172	#20010227	Human	2010	Malaga, Spain
1	5	3	13	3	2	3	2	7	9	7	4	3	3	4	5	2010Valdezate#066	B. melitensis	43	171	#20051126	Human	2005	Toledo, Spain
1	5	3	13	5	2	3	2	4	41	7	4	6	3	4	4	2010Valdezate#063	B. melitensis	100	169	#20080920	Human	2008	Vizcaya, Spain
1	5	3	13	2	3	3	2	5	43	8	3	6	3	7	4	2010Valdezate#065	B. melitensis	63	173	#20090995	Human	2009	Ciudad Real, Spain
1	5	2	13	3	2	3	2	4	41	8	8	3	6	4	5	2018Vergnaud#0027	B. melitensis bv1	59	117	BCCN#83-142	Caprine	1983	Greece
1	5	2	13	3	2	3	2	4	41	8	6	3	6	4	5	2018Vergnaud#0443	B. melitensis bv1	59	117	BCCN#83-154	Ovine	1983	Greece
1	5	2	13	3	2	3	2	4	41	8	5	3	6	5	5	2007AlDahouk#055	B. melitensis bv1	59	117	BfR 14	Human	2000	Munchen, Germany
1	5	2	13	3	2	3	2	4	41	8	6	3	8	8	5	2018Vergnaud#0444	B. melitensis bv1	59	117	BCCN#83-162	Human	1983	Greece
1	5	2	13	3	2	3	2	4	41	8	4	3	4	8	4	2018Vergnaud#0280	B. melitensis bv1	59	117	BCCN#91-137	Cattle	1991	Salonique ,Greece
1	5	2	13	3	2	3	2	6	41	8	1	3	4	8	4	2018Vergnaud#0281	B. melitensis bv1	59	290	BCCN#91-138	Cattle	1991	Salonique ,Greece
1	4	3	13	2	2	3	2	6	43	8	4	5	7	3	3	2013Jiang#026	B. melitensis bv1	115	278	JL85-02	Deer/El.	1985	Jilin, China
1	4	3	13	2	2	3	2	6	43	8	4	6	4	3	3	2013Jiang#027	B. melitensis bv1	115	278	NX79-104	Ovine	1979	Ningxia, China
1	5	3	13	2	2	3	2	4	43	8	4	6	7	6	3	2013Jiang#043	B. melitensis bv1	42	108	NM88-0907	Ovine	1988	Inner Mongolia, China
1	5	3	13	2	2	3	2	4	36	8	4	5	7	9	5	2017Liu#001	B. melitensis	42	342	WS000	Human	2011	Shangdu, China
3	5	3	13	4	2	3	4	4	41	8	6	4	3	7	4	2016Gyuranecz#007	B. melitensis	121	224	C7	Camel	2009	Sudan
3	5	3	13	4	2	3	4	4	41	8	6	4	3	7	4	2016Gyuranecz#009	B. melitensis	121	224	C9	Camel	2010	Sudan
3	5	3	13	4	2	3	4	6	41	8	6	4	3	7	4	2016Gyuranecz#008	B. melitensis	121	226	C8	Camel	2010	Sudan
3	5	3	13	4	2	3	4	5	41	8	4	4	3	5	4	2016Gyuranecz#006	B. melitensis	121	225	C6	Camel	2009	Sudan
2	5	3	13	4	2	3	4	7	36	8	5	4	8	7	4	2007AlDahouk#061	B. melitensis bv1	66	84	BfR 27	Human	2002	
2	5	8	13	1	2	3	2	4	41	8	6	4	3	5	4	2018Vergnaud#0251	B. abortus bv1_.	131	277	BCCN#83-36er.	Cattle_?	198.	La Réunion, France_?
3	5	3	13	3	2	3	3	6	41	8	7	4	5	5	3	2007AlDahouk#060	B. melitensis bv1	65	98	BfR 20	Human	1998	Pakistan
3	5	3	13	3	2	3	3	6	41	8	6	4	6	5	3	2011Aftab#006	B. melitensis	65	98	NV5593-63	Human		Turkey
1	5	3	14	3	1	3	3	4	43	8	4	4	3	4	5	2018Vergnaud#0459	B. melitensis bv1	128	303	BCCN#89-85	Ovine	1989	Saragosse, Spain
1	5	3	13	3	1	3	3	4	43	8	7	4	3	6	5	2018Vergnaud#0460	B. melitensis bv1	135	302	BCCN#90-12	Ovine	1990	Saragosse, Spain
1	5	3	13	3	1	3	3	4	45	8	3	4	3	7	5	2018Vergnaud#0465	B. melitensis bv1	135	301	BCCN#91-233	Ovine	1991	Navarre, Spain
1	5	3	13	3	1	2	3	6	43	8	4	4	3	8	5	2018Vergnaud#0464	B. melitensis bv1	127	299	BCCN#91-228	Ovine	1991	Saragosse, Spain
1	5	3	13	3	1	3	3	5	49	8	3	4	3	6	6	2018Vergnaud#0462	B. melitensis bv1	135	300	BCCN#91-158	Ovine	1991	Somme, France
1	5	3	13	1	2	2	2	6	49	8	9	5	6	4	5	2018Vergnaud#0578	B. melitensis bv3	149	281	BCCN#89-30	Ovine	1989	Hautes-Pyrénées, France
1	5	3	13	1	2	3	2	6	9	8	9	4	11	4	5	2018Vergnaud#0579	B. melitensis bv3	84	282	BCCN#90-153	Cattle	1990	Salonique ,Greece
1	5	3	13	1	2	3	2	6	9	8	7	5	9	5	5	2018Vergnaud#0581	B. melitensis bv3	84	282	BCCN#90-166	Ovine	1990	Pyrénées-Orientales, F.
3	5	3	13	4	2	3	5	4	36	8	5	4	4	5	6	2016Gyuranecz#010	B. melitensis	120	222	C10	Gazelle	2013	United Arab Emirates
3	5	3	13	4	2	3	5	6	36	8	5	7	4	4	5	2018Vergnaud#0285	B. melitensis bv1	132	276	BCCN#96-99	Caprine	1996	South Africa
2	5	3	13	4	2	3	5	6	36	8	5	8	5	3	5	2018Vergnaud#0470	B. melitensis bv1	140	275	BCCN#02-38	Human	2002	France
3	5	3	12	4	2	3	5	7	36	8	5	4	7	6	5	2006LeFleche#127	B. melitensis bv1	41	85	BfR 23	Caprine		United Arab Emirates
3	5	3	12	4	2	3	5	7	36	8	4	4	6	6	5	2006LeFleche#128	B. melitensis bv1	41	85	BfR 25	Caprine		United Arab Emirates
3	5	3	12	4	2	3	5	7	36	8	5	5	6	5	6	2018Vergnaud#0371	B. melitensis R.	41	85	BCCN#04-6	Caprine	2004	Oman
3	5	3	12	4	2	3	5	7	36	8	5	5	6	5	6	2018Vergnaud#0372	B. melitensis R.	41	85	BCCN#04-7	Caprine	2004	Oman
2	5	3	13	3	2	3	5	4	36	8	5	5	7	7	6	2011Aftab#012	B. melitensis	94	178	W14744	Human		Somalia
2	5	3	13	4	2	3	5	5	36	8	4	5	7	8	5	2017Hanot-Mamb.	B. melitensis bv1	140	361	L3/09	Human	1996	Belgium
3	5	3	12	2	2	3	3	7	45	8	13	4	3	3	3	2013Minharro#039	B. abortus bv6	40	324	165	Cattle	2008	Cumaru do Norte, 683.
3	5	3	12	2	2	3	3	7	45	8	13	4	3	3	3	2013Minharro#073	B. abortus bv6	40	324	218	Cattle	2008	Sao Geraldo, 68570000.
3	5	3	12	2	2	3	3	7	45	8	4	4	3	3	3	2013Minharro#035	B. abortus bv6	40	324	161	Cattle	2008	Redencao, 68550010, .
3	5	3	12	2	2	3	3	7	45	8	8	4	3	3	3	2013Minharro#036	B. abortus bv3	40	324	162	Cattle	2008	Conceicao do Araguaia,.
3	5	3	12	2	2	3	3	7	45	8	10	4	3	3	3	2013Minharro#038	B. abortus bv3	40	324	164	Cattle	2008	Conceicao do Araguaia,.
3	5	3	12	2	2	3	3	7	43	8	14	4	3	3	3	2013Minharro#070	B. abortus bv3	40	66	211	Cattle	2008	Araguaina, 7780902, B.
3	5	3	12	2	2	3	3	7	43	8	14	4	3	3	3	2013Minharro#071	B. abortus bv3	40	66	216	Cattle	2008	Conceicao do Araguaia,.
3	5	3	12	2	2	3	3	7	43	8	6	4	3	3	3	2013Minharro#045	B. abortus bv6	40	66	172	Cattle	2008	Conceicao do Araguaia,.
3	5	3	12	2	2	3	3	7	43	8	6	4	3	3	3	2013Minharro#046	B. abortus bv6	40	66	173	Cattle	2008	Cumaru do Norte, 683.
3	5	3	12	2	2	3	3	7	43	8	6	4	3	3	3	2013Minharro#067	B. abortus bv3	40	66	207	Cattle	2008	Maraba, 68500005, Brazil
3	5	3	12	2	2	3	3	7	43	8	6	4	3	3	3	2013Minharro#068	B. abortus bv3	40	66	208	Cattle	2008	Juarina, 77753000, Brazil
3	5	3	12	2	2	3	3	7	43	8	5	4	3	3	3	2013Minharro#040	B. abortus bv6	40	66	166	Cattle	2008	Redencao, 68550010, .
3	5	3	12	2	2	3	3	7	43	8	5	4	3	3	3	2013Minharro#042	B. abortus bv6	40	66	168	Cattle	2008	Redencao, 68550010, .
3	5	3	12	2	2	3	3	7	43	8	5	4	3	3	3	2013Minharro#047	B. abortus bv3	40	66	174	Cattle	2008	Cumaru do Norte, 683.
3	5	3	12	2	2	3	3	7	43	8	5	4	3	3	3	2018Vergnaud#0110	B. abortus atypi.	40	66	BCCN#77-79A	Human	1977	Paris, France
3	5	3	12	2	2	3	3	7	43	8	7	4	3	3	3	2013Minharro#025	B. abortus bv3	40	66	62	Cattle	2008	Cabo Verde, 37880000.
3	5	3	12	2	2	3	3	7	43	8	7	4	3	3	3	2013Minharro#043	B. abortus bv6	40	66	169	Cattle	2008	Couto Magalhaes, 777.
3	5	3	12	2	2	3	3	7	43	8	7	4	3	3	3	2013Minharro#044	B. abortus bv6	40	66	170	Cattle	2008	Floresta do Araguaia, 6.
3	5	3	12	2	2	3	3	7	43	8	9	4	3	3	3	2013Minharro#026	B. abortus bv3	40	66	66	Cattle	2008	Cabo Verde, 37880000.
3	5	3	12	2	2	3	3	7	43	8	12	4	3	3	3	2013Minharro#041	B. abortus bv6	40	66	167	Cattle	2008	Redencao, 68550010, .
3	5	3	12	2	2	3	3	7	43	8	13	4	3	3	3	2013Minharro#066	B. abortus bv3	40	66	206	Cattle	2008	Floresta do Araguaia, 6.
3	5	3	12	2	2	3	3	7	43	8	4	4	3	3	3	2013Minharro#074	B. abortus bv3	40	66	219	Cattle	2008	Conceicao do Araguaia,.
3	5	3	12	2	2	3	3	6	43	8	8	4	3	3	3	2013Minharro#108	B. abortus bv6	40	329	SP 3			Sao Paulo, 01036970, .
3	5	3	12	2	2	3	3	7	43	9	13	4	3	3	3	2013Minharro#050	B. abortus bv3	40	322	182	Cattle	2008	Maraba, 68500005, Brazil
3	5	3	12	2	2	3	3	7	43	9	13	4	3	3	3	2013Minharro#051	B. abortus bv3	40	322	187	Cattle	2008	Muricilandia, 77848000.
3	5	3	12	2	2	3	3	7	43	9	13	4	3	3	3	2013Minharro#052	B. abortus bv3	40	322	188	Cattle	2008	Augustinopolis, 779699.
3	5	3	12	2	2	3	3	7	43	9	13	4	3	3	3	2013Minharro#054	B. abortus bv6	40	322	190	Cattle	2008	Conceicao do Araguaia,.
3	5	3	12	2	2	3	3	7	43	9	13	4	3	3	3	2013Minharro#055	B. abortus bv3	40	322	192	Cattle	2008	Maraba, 68500005, Brazil
3	5	3	12	2	2	3	3	7	43	9	4	4	3	3	3	2013Minharro#053	B. abortus bv3	40	322	189	Cattle	2008	Palestina do Para, 685.
3	5	3	12	2	2	3	3	7	43	9	8	4	3	3	3	2013Minharro#136	B. abortus bv6	40	322	Jr 24	Cattle	2008	Sao Paulo, 01036970, .
3	5	3	12	2	2	3	3	7	43	9	8	4	3	3	4	2013Minharro#135	B. abortus bv3	40	322	Jr 31	Cattle	2008	Sao Paulo, 01036970, .
3	5	4	12	2	2	3	3	7	43	9	8	4	3	3	3	2013Minharro#137	B. abortus bv1	33	321	Jr 05	Cattle	2008	Sao Paulo, 01036970, .
3	5	3	12	2	2	3	3	7	43	9	7	5	3	3	3	2013Minharro#089	B. abortus bv6	40	322	17a/02	Cattle	2002	Porto Alegre, 90570020.

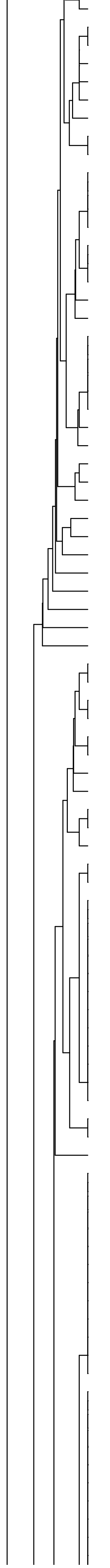

3	5	3	12	2	2	3	3	7	43	8	7	5	3	3	3	2013Minharro#090	B. abortus bv6	40	66		17b/02	Cattle	2002	Porto Alegre, 90570020.
3	5	3	12	2	2	3	3	7	43	8	13	4	3	4	3	2013Minharro#059	B. abortus bv3	40	66		198	Cattle	2008	Conceicao do Araguaia,.
3	5	3	12	2	2	3	3	7	43	8	13	4	3	4	3	2013Minharro#060	B. abortus bv3	40	66		199	Cattle	2008	Juarina, 68540000, Brazil
3	5	3	12	2	2	3	3	7	43	8	5	4	3	4	3	2013Minharro#057	B. abortus bv3	40	66		195	Cattle	2008	Maraba, 68500005, Brazil
3	5	3	12	2	2	3	3	7	43	8	14	4	3	4	3	2013Minharro#061	B. abortus bv3	40	66		200	Cattle	2008	Conceicao do Araguaia,.
3	5	3	12	2	2	3	3	7	43	8	4	4	3	4	3	2013Minharro#062	B. abortus bv3	40	66		201	Cattle	2008	Bandeirantes, 77783000.
3	5	3	12	2	2	3	3	7	43	8	5	2	3	4	3	2013Minharro#058	B. abortus bv3	40	66		196	Cattle	2008	Bandeirantes, 77783000.
4	5	3	12	2	2	3	3	7	43	8	6	4	3	4	3	2013Jiang#118	B. abortus bv3	112	326		NM1102	Cattle	1985	Inner Mongolia, China
4	5	3	12	2	2	3	3	7	43	8	6	4	3	4	3	2013Jiang#134	B. abortus bv3	112	326		NM1161	Cattle	1989	Inner Mongolia, China
3	5	3	12	2	2	2	3	7	43	8	6	6	3	3	3	2006LeFleche#007	B. abortus bv9	39	67		REF C68	Cattle		England
3	5	3	12	2	2	2	3	7	43	8	6	6	3	3	3	2009Her#007	B. abortus bv9	39	67		KRef08	Cattle		England
3	5	3	12	2	2	2	3	7	43	8	6	6	3	3	3	2012Ferreira#134	B. abortus bv9	39	67		REF C68	Cattle		
3	5	3	12	2	2	2	3	7	43	8	6	6	3	3	3	SRR032599	B. abortus bv9	39	67	30	REF C68			
3	5	3	12	2	2	2	3	7	43	8	6	7	3	3	3	2006LeFleche#006	B. abortus bv5	39	67		REF B3196	Cattle		England
3	5	3	12	2	2	2	3	7	43	8	6	7	3	3	3	2009Her#005	B. abortus bv5	39	67		KRef05	Cattle		England
3	5	3	12	2	2	2	3	7	43	8	6	7	3	3	3	2012Ferreira#133	B. abortus bv5	39	67		REF B3196	Cattle		
3	5	3	12	2	2	2	3	7	43	8	4	6	3	3	3	2006LeFleche#118	B. abortus bv9	39	67		BCCN#87-46	Cattle	1987	Tarbes, France
2	5	3	12	2	2	2	3	7	43	8	6	6	3	3	3	GCA_000740195	B. abortus bv9	179	347	30	REF C68	Cattle		
3	5	3	12	2	2	3	3	7	43	8	3	6	3	3	3	2006LeFleche#009	B. abortus bv6	40	66		REF 870	Cattle		Africa
3	5	3	12	2	2	3	3	7	43	8	3	6	3	3	3	2009Her#006	B. abortus bv6	40	66		KRef06	Cattle		Africa
3	5	3	12	2	2	3	3	7	43	8	3	6	3	3	3	2012Ferreira#135	B. abortus bv6	40	66		REF 870	Cattle		
3	5	3	12	2	2	3	3	7	43	8	3	6	3	3	3	GCA_000740215	B. abortus bv6	40	66	4	REF 870 (NCT.	Cattle		
3	5	3	12	2	2	3	3	7	43	8	3	6	3	3	3	SRR032629	B. abortus bv6	40	66		REF 870	Cattle		
3	5	3	12	2	2	3	3	7	43	8	8	6	3	3	3	2013Minharro#027	B. abortus bv6	40	66		70	Cattle	2007	Cabo Verde, 37880000.
2	5	3	12	2	2	3	3	7	43	8	3	6	3	3	3	GCA_000740175	B. abortus	178	348	4	REF 870 (NCT.	Cattle		
3	5	3	12	2	2	3	3	7	49	8	9	4	3	3	4	2013Minharro#001	B. abortus bv3	40	323		A1	Cattle	2004	Araguaina, 7780902, B.
3	5	3	12	2	2	3	3	7	49	8	9	4	3	3	8	2013Minharro#002	B. abortus bv3	40	323		A4	Cattle	2004	Conceicao do Araguaia,.
3	5	3	12	2	2	3	3	7	43	8	9	4	3	3	4	2013Minharro#010	B. abortus bv6	40	66		37	Cattle	2006	Governador Valadares,.
3	5	3	12	2	2	3	3	7	45	7	7	4	3	3	3	2013Minharro#024	B. abortus bv6	40	320		61	Cattle	2005	Cabo Verde, 37880000.
3	5	3	12	2	2	3	1	7	45	8	7	4	3	3	3	2013Minharro#037	B. abortus bv3	167	319		163	Cattle	2008	Guarai, 77700000, Brazil
3	5	3	12	2	2	3	3	7	45	8	9	4	7	3	3	2013Minharro#003	B. abortus bv3	40	324		A6	Cattle	2004	Conceicao do Araguaia,.
4	4	3	12	2	2	3	3	7	43	8	13	4	3	3	3	2013Minharro#072	B. abortus bv3	168	325		217	Cattle	2008	Conceicao do Araguaia,.
3	5	3	12	2	2	2	3	7	43	8	4	5	3	4	3	2006LeFleche#117	B. abortus bv9	39	67		BCCN#80-133	Dog	1980	Belgium
3	5	3	12	2	2	3	3	9	43	3	5	4	3	8	3	2013Minharro#064	B. abortus bv6	40	318		203	Cattle	2008	Itupiranga, 68580000, .
2	5	3	12	2	2	2	3	7	43	8	5	8	6	3	3	GCA_000740295	B. abortus	179	347	30	63 75			
4	5	4	12	2	2	3	3	7	45	8	7	4	3	4	3	2013Minharro#017	B. abortus bv1	28	335		45	Cattle	2006	Governador Valadares,.
4	5	3	12	2	2	3	3	6	43	8	5	4	3	3	5	2013Jiang#148	B. abortus bv3	112	210		NM1210	Cattle	1993	Inner Mongolia, China
4	5	3	12	2	2	3	3	6	43	8	5	4	3	3	5	2013Jiang#149	B. abortus bv3	112	210		NM1211	Cattle	1993	Inner Mongolia, China
4	5	3	12	2	2	3	3	6	43	8	5	4	3	3	3	2013Jiang#110	B. abortus bv3	112	210		NM1071	Cattle	1985	Inner Mongolia, China
4	5	3	12	2	2	3	3	6	43	8	5	4	3	3	3	2013Jiang#129	B. abortus bv3	112	210		NM1149	Cattle	1988	Inner Mongolia, China
4	5	3	12	2	2	3	3	7	43	8	5	4	3	3	3	2013Jiang#111	B. abortus bv3	112	326		NM1072	Cattle	1985	Inner Mongolia, China
4	5	3	12	2	2	3	3	7	43	8	5	4	3	3	3	2013Jiang#112	B. abortus bv3	112	326		NM1074	Cattle	1985	Inner Mongolia, China
4	5	3	12	2	2	3	3	6	43	8	5	4	7	3	3	2013Jiang#104	B. abortus bv3	112	210		NM1061	Cattle	1984	Inner Mongolia, China
4	5	3	12	2	2	3	2	6	43	8	5	4	3	3	3	2013Jiang#127	B. abortus bv3	117	328		NM1147	Cattle	1988	Inner Mongolia, China
4	5	3	12	2	2	3	3	6	42	8	6	4	3	3	3	2017Liu#177	B. abortus bv3	112	210		057-2		2015	
4	5	3	12	2	2	3	3	6	42	8	6	4	3	3	3	2017Liu#178	B. abortus bv3	112	210		061-1		2015	
4	5	3	12	2	2	3	3	6	42	8	5	4	3	3	3	2017Liu#176	B. abortus bv3	112	210		057-1		2015	
4	5	2	12	2	2	3	3	6	43	8	4	5	3	3	3	2013Garofolo_18.	B. abortus bv3	108	208		18080	Cattle	2011	Salerno,Italy
4	5	2	12	2	2	3	3	6	43	8	4	5	3	3	3	2013Garofolo_8486	B. abortus bv3	108	208		8486	Cattle	2011	Giffoni Valle Piana,Italy
4	5	3	12	2	2	3	3	6	43	8	4	5	3	3	3	2013Garofolo_1046	B. abortus bv3	112	210		1046	Cattle	2011	Roccaspinalveti,Italy
4	5	3	12	2	2	3	3	6	43	8	4	5	3	3	3	2013Garofolo_1366	B. abortus bv3	112	210		1366	Cattle	2011	Vastogirardi,Italy
4	5	3	12	2	2	3	3	6	43	8	4	5	3	3	3	2013Garofolo_1368	B. abortus bv3	112	210		1368	Cattle	2011	Agnone,Italy
4	5	3	12	2	2	3	3	6	43	8	4	5	3	3	3	2013Garofolo_1369	B. abortus bv3	112	210		1369	Cattle	2011	Pescopennataro,Italy
4	5	3	12	2	2	3	3	6	43	8	4	5	3	3	3	2013Garofolo_15.	B. abortus bv3	112	210		15073	Cattle	2011	San Marco in Lamis,Italy
4	5	3	12	2	2	3	3	6	43	8	4	5	3	3	3	2013Garofolo_15.	B. abortus bv3	112	210		15076	Cattle	2011	San Marco in Lamis,Italy
4	5	3	12	2	2	3	3	6	43	8	4	5	3	3	3	2013Garofolo_1600	B. abortus bv3	112	210		1600	Cattle	2011	Agnone,Italy
4	5	3	12	2	2	3	3	6	43	8	4	5	3	3	3	2013Garofolo_20.	B. abortus bv3	112	210		20016	Cattle	2011	Apricena,Italy
4	5	3	12	2	2	3	3	6	43	8	4	5	3	3	3	2013Garofolo_2078	B. abortus bv3	112	210		2078	Cattle	2011	Pescopennataro,Italy
4	5	3	12	2	2	3	3	6	43	8	4	5	3	3	3	2013Garofolo_2705	B. abortus bv3	112	210		2705	Cattle	2011	Pescara,Italy
4	5	3	12	2	2	3	3	6	43	8	4	5	3	3	3	2013Garofolo_3915	B. abortus bv3	112	210		3915	Cattle	2011	Pescopennataro,Italy
4	5	3	12	2	2	3	3	6	43	8	4	5	3	3	3	2013Garofolo_8982	B. abortus bv3	112	210		8982	Cattle	2011	San Marci in Lamis,Italy
4	5	3	12	2	2	3	3	6	43	8	7	6	3	3	3	2013Garofolo_18.	B. abortus bv3	112	210		18081	Cattle	2011	Apricena,Italy
4	5	3	12	2	2	3	3	6	43	8	7	6	3	3	3	2013Garofolo_3272	B. abortus bv3	112	210		3272	Cattle	2011	Apricena,Italy
4	5	3	12	2	2	3	2	8	43	8	5	6	3	3	3	2013Jiang#113	B. abortus bv3	117	327		NM1075	Cattle	1985	Inner Mongolia, China
4	5	3	12	2	2	3	1	6	43	8	4	7	8	3	3	2013Garofolo_12.	B. abortus bv3	36	72		12184	Cattle	2011	Sanza,Italy
4	5	3	12	2	2	3	1	6	43	8	4	7	8	3	3	2013Jiang#096	B. abortus bv3	36	72			Cattle	2010	Helongjiang, China
4	5	3	12	2	2	3	1	6	43	8	4	7	8	3	3	2013Jiang#097	B. abortus bv3	36	72			Cattle	2010	Helongjiang, China
4	5	3	12	2	2	3	1	6	43	8	4	7	8	3	3	2013Jiang#098	B. abortus bv3	36	72			Cattle	2010	Helongjiang, China
4	5	3	12	2	2	3	1	6	43	8	4	7	8	3	3	2013Jiang#099	B. abortus bv3	36	72			Cattle	2010	Helongjiang, China
4	5	3	12	2	2	3	1	6	43	8	4	7	8	3	3	2016Shevtsova#047	B. abortus	36	72		109-15-G (5)	Cattle	2015	Tasty, Celinogradski, .
4	5	3	12	2	2	3	1	6	43	8	4	7	8	3	3	2016Shevtsova#054	B. abortus	36	72		160-15-G (13)	Cattle	2015	Tasty, Celinogradski, .
4	5	3	12	2	2	3	1	6	43	8	4	7	8	3	3	2016Shevtsova#061	B. abortus	36	72		116/2-15-G (25)	Cattle	2015	Mereyskiy, Taskalinskiy.
4	5	3	12	2	2	3	1	6	43	8	4	7	8	3	3	2016Shevtsova#062	B. abortus	36	72		116/2-15-G (24)	Cattle	2015	Budarinskiy, Akzhaikskiy.
4	5	3	12	2	2	3	1	6	43	8	4	7	8	3	3	2016Shevtsova#063	B. abortus	36	72		116/2-15-G (22)	Cattle	2015	Kyzyloba, Zhangalinskiy.
4	5	3	12	2	2	3	1	6	43	8	4	7	8	3	3	2016Shevtsova#073	B. abortus	36	72		116/2-15-G (13)	Cattle	2015	Kyzyloba, Zhangalinskiy.
4	5	3	12	2	2	3	1	6	43	8	4	7	8	3	3	2016Shevtsova#082	B. abortus	36	72		116/2-15-G (3)	Cattle	2015	Sholakankaty, Syrymsky.
4	5	3	12	2	2	3	1	6	43	8	4	6	8	3	3	2013Garofolo_12.	B. abortus bv3	36	72		12178	Cattle	2011	Sala Consilina,Italy
4	5	3	12	2	2	3	1	6	43	8	4	6	8	3	3	2013Garofolo_20.	B. abortus bv3	36	72		20825	Cattle	2011	San Rufo,Italy
4	5	3	12	2	2	3	1	6	43	8	4	6	8	3	3	2013Garofolo_2683	B. abortus bv3	36	72		2683	Buffalo	2011	Capaccio,Italy
4	5	3	12	2	2	3	1	6	43	8	4	6	8	3	3	2013Garofolo_2685	B. abortus bv3	36	72		2685	Buffalo	2011	Eboli,Italy
4	5	3	12	2	2	3	1	6	43	8	4	6	8	3	3	2013Garofolo_2686	B. abortus bv3	36	72		2686	Cattle	2011	Campana,Italy
4	5	3	12	2	2	3	1	6	43	8	4	6	8	3	3	2013Garofolo_3634	B. abortus bv3	36	72		3634	Buffalo	2011	Capaccio,Italy
4	5	3	12	2	2	3	1	6	43	8	4	6	8	3	3	2013Garofolo_5359	B. abortus bv3	36	72		5359	Buffalo	2011	Capaccio,Italy
4	5	3	12	2	2	3	1	6	43	8	4	6	8	3	3	2013Garofolo_5360	B. abortus bv3	36	72		5360	Buffalo	2011	Capaccio,Italy
4	5	3	12	2	2	3	1	6	43	8	4	6	8	3	3	2016Shevtsova#001	B. abortus	36	72		228-15-B/G (6)	Cattle	2015	Tasty, Celinogradski, .
4	5	3	12	2	2	3	1	6	43	8	4	6	8	3	3	2016Shevtsova#002	B. abortus	36	72		228-15-B/G (2)	Cattle	2015	Tasty, Celinogradski, .

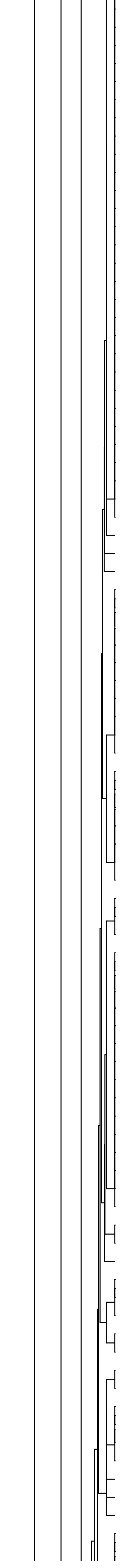

4	5	3	12	2	2	3	1	6	43	8	4	6	8	3	3	2016Shevtsova#003	B. abortus	36	72	228-15-B/G (11)	Cattle	2015	Tasty, Celinogradski, .
4	5	3	12	2	2	3	1	6	43	8	4	6	8	3	3	2016Shevtsova#004	B. abortus	36	72	228-15-B/G (5)	Cattle	2015	Tasty, Celinogradski, .
4	5	3	12	2	2	3	1	6	43	8	4	6	8	3	3	2016Shevtsova#005	B. abortus	36	72	228-15-B/G (18)	Cattle	2015	Tasty, Celinogradski, .
4	5	3	12	2	2	3	1	6	43	8	4	6	8	3	3	2016Shevtsova#006	B. abortus	36	72	228-15-B/G (7)	Cattle	2015	Tasty, Celinogradski, .
4	5	3	12	2	2	3	1	6	43	8	4	6	8	3	3	2016Shevtsova#007	B. abortus	36	72	228-15-B/G (8)	Cattle	2015	Tasty, Celinogradski, .
4	5	3	12	2	2	3	1	6	43	8	4	6	8	3	3	2016Shevtsova#008	B. abortus	36	72	228-15-B/G (19)	Cattle	2015	Tasty, Celinogradski, .
4	5	3	12	2	2	3	1	6	43	8	4	6	8	3	3	2016Shevtsova#009	B. abortus	36	72	228-15-B/G (12)	Cattle	2015	Tasty, Celinogradski, .
4	5	3	12	2	2	3	1	6	43	8	4	6	8	3	3	2016Shevtsova#010	B. abortus	36	72	228-15-B/G (17)	Cattle	2015	Tasty, Celinogradski, .
4	5	3	12	2	2	3	1	6	43	8	4	6	8	3	3	2016Shevtsova#011	B. abortus	36	72	228-15-B/G (20)	Cattle	2015	Tasty, Celinogradski, .
4	5	3	12	2	2	3	1	6	43	8	4	6	8	3	3	2016Shevtsova#012	B. abortus	36	72	228-15-B/G (3)	Cattle	2015	Tasty, Celinogradski, .
4	5	3	12	2	2	3	1	6	43	8	4	6	8	3	3	2016Shevtsova#013	B. abortus	36	72	228-15-B/G (13)	Cattle	2015	Tasty, Celinogradski, .
4	5	3	12	2	2	3	1	6	43	8	4	6	8	3	3	2016Shevtsova#014	B. abortus	36	72	228-15-B/G (16)	Cattle	2015	Tasty, Celinogradski, .
4	5	3	12	2	2	3	1	6	43	8	4	6	8	3	3	2016Shevtsova#015	B. abortus	36	72	228-15-B/G (9)	Cattle	2015	Tasty, Celinogradski, .
4	5	3	12	2	2	3	1	6	43	8	4	6	8	3	3	2016Shevtsova#016	B. abortus	36	72	228-15-B/G (10)	Cattle	2015	Tasty, Celinogradski, .
4	5	3	12	2	2	3	1	6	43	8	4	6	8	3	3	2016Shevtsova#017	B. abortus	36	72	228-15-B/G (4)	Cattle	2015	Tasty, Celinogradski, .
4	5	3	12	2	2	3	1	6	43	8	4	6	8	3	3	2016Shevtsova#019	B. abortus	36	72	228-15-B/G (14)	Cattle	2015	Tasty, Celinogradski, .
4	5	3	12	2	2	3	1	6	43	8	4	6	8	3	3	2016Shevtsova#048	B. abortus	36	72	160-15-G (20)	Cattle	2015	Tasty, Celinogradski, .
4	5	3	12	2	2	3	1	6	43	8	4	6	8	3	3	2016Shevtsova#049	B. abortus	36	72	160-15-G (19)	Cattle	2015	Tasty, Celinogradski, .
4	5	3	12	2	2	3	1	6	43	8	4	6	8	3	3	2016Shevtsova#050	B. abortus	36	72	160-15-G (18)	Cattle	2015	Tasty, Celinogradski, .
4	5	3	12	2	2	3	1	6	43	8	4	6	8	3	3	2016Shevtsova#051	B. abortus	36	72	160-15-G (17)	Cattle	2015	Tasty, Celinogradski, .
4	5	3	12	2	2	3	1	6	43	8	4	6	8	3	3	2016Shevtsova#052	B. abortus	36	72	160-15-G (16)	Cattle	2015	Tasty, Celinogradski, .
4	5	3	12	2	2	3	1	6	43	8	4	6	8	3	3	2016Shevtsova#055	B. abortus	36	72	160-15-G (11)	Cattle	2015	Tasty, Celinogradski, .
4	5	3	12	2	2	3	1	6	43	8	4	6	8	3	3	2016Shevtsova#056	B. abortus	36	72	160-15-G (10)	Cattle	2015	Tasty, Celinogradski, .
4	5	3	12	2	2	3	1	6	43	8	4	6	8	3	3	2016Shevtsova#057	B. abortus	36	72	160-15-G (9)	Cattle	2015	Tasty, Celinogradski, .
4	5	3	12	2	2	3	1	6	43	8	4	6	8	3	3	2016Shevtsova#058	B. abortus	36	72	160-15-G (6)	Cattle	2015	Tasty, Celinogradski, .
4	5	3	12	2	2	3	1	6	43	8	4	6	8	3	3	2016Shevtsova#060	B. abortus	36	72	160-15-G (1)	Cattle	2015	Tasty, Celinogradski, .
4	5	3	12	2	2	3	1	6	43	8	4	6	8	3	3	2016Shevtsova#085	B. abortus	36	72	155-15-G (7)	Cattle	2015	Tasty, Celinogradski, .
4	5	3	12	2	2	3	1	6	43	8	4	6	8	3	3	2016Shevtsova#087	B. abortus	36	72	155-15-G (2)	Cattle	2015	Tasty, Celinogradski, .
4	5	3	12	2	2	3	1	6	43	8	4	6	8	3	3	2016Shevtsova#088	B. abortus	36	72	150-15-G (13)	Cattle	2015	Tasty, Celinogradski, .
4	5	3	12	2	2	3	1	6	43	8	4	5	8	3	3	2016Shevtsova#053	B. abortus	36	72	160-15-G (15)	Cattle	2015	Tasty, Celinogradski, .
4	5	3	12	2	2	3	1	6	43	8	4	6	5	3	3	2013Jiang#083	B. abortus bv3	36	72		Cattle	2011	Xinjiang, China
4	5	3	12	2	2	3	1	6	43	8	4	6	8	3	2	2013Garofolo_3273	B. abortus bv3	36	72	3273	Cattle	2011	Felitto,Italy
4	5	3	12	2	2	3	1	6	43	8	4	6	6	3	3	2012Ferreira#152	B. abortus bv3	36	72	LNIV-109Ba3-03	Cattle	2003	Beira Interior, Portugal
4	5	3	12	2	2	3	1	6	43	8	4	6	6	3	3	2012Ferreira#153	B. abortus bv3	36	72	LNIV-120Ba3-03	Cattle	2003	Beira Interior, Portugal
4	5	3	12	2	2	3	1	6	43	8	4	6	6	3	3	2012Ferreira#154	B. abortus bv3	36	72	LNIV-141Ba3-04	Cattle	2004	Beira Interior, Portugal
4	5	3	12	2	2	3	1	6	43	8	4	6	6	3	3	2012Ferreira#164	B. abortus bv3	36	72	LNIV-130Ba3-03	Cattle	2003	Entre Douro e Minho, P.
4	5	3	12	2	2	3	1	6	43	8	4	6	6	3	3	2013Garofolo_3921	B. abortus bv3	36	72	3921	Cattle	2011	San Gregorio Magno,Italy
4	5	3	12	2	2	3	1	6	43	8	4	6	6	3	3	2013Garofolo_5007	B. abortus bv3	36	72	5007	Cattle	2011	San Gregorio Magno,Italy
4	5	3	12	2	2	3	1	6	43	8	4	6	6	3	3	2013Garofolo_6861	B. abortus bv3	36	72	6861	Cattle	2011	Agropoli,Italy
4	5	3	12	2	2	3	1	6	43	8	4	6	6	3	3	2013Garofolo_6863	B. abortus bv3	36	72	6863	Cattle	2011	San Gregorio Magno,Italy
4	5	3	12	2	2	3	1	6	43	8	4	6	6	3	3	2013Garofolo_8055	B. abortus bv3	36	72	8055	Cattle	2011	Calvello,Italy
4	5	3	12	2	2	3	1	6	43	8	4	6	6	3	3	2016Shevtsova#059	B. abortus	36	72	160-15-G (5)	Cattle	2015	Tasty, Celinogradski, .
4	5	3	12	2	2	3	1	6	43	8	4	7	6	3	3	2012Ferreira#149	B. abortus bv3	36	72	LNIV-320Ba3-06	Cattle	2006	Alentejo, Portugal
4	5	3	12	2	2	3	1	6	43	8	4	7	6	3	3	2012Ferreira#150	B. abortus bv3	36	72	LNIV-375Ba3-07	Cattle	2007	Alentejo, Portugal
4	5	3	12	2	2	3	1	6	43	8	4	7	6	3	3	2012Ferreira#151	B. abortus bv3	36	72	LNIV-376Ba3-07	Cattle	2007	Alentejo, Portugal
4	5	3	12	2	2	3	1	6	43	8	4	7	6	3	3	2016Shevtsova#027	B. abortus	36	72	1106	Cattle	1961	Esik, Enbekshikazakh, .
4	5	3	12	2	2	3	1	6	43	8	4	7	6	3	3	2016Shevtsova#036	B. abortus	36	72	67Al	Cattle	1967	Kaskelen, Almaty, Kara.
4	5	3	12	2	2	3	1	6	43	8	4	7	6	3	3	2016Shevtsova#038	B. abortus	36	72	1105	Cattle	1961	Esik, Enbekshikazakh, .
4	5	3	12	2	2	3	1	6	43	8	4	7	6	3	3	2016Shevtsova#076	B. abortus	36	72	116/2-15-G (10)	Cattle	2015	Masteksayskiy, Zhangal.
4	5	3	12	2	2	3	1	6	43	8	5	6	7	3	3	2013Garofolo_9263	B. abortus bv3	36	72	9263	Buffalo	2011	Albanella,Italy
4	5	3	12	2	2	3	1	6	43	8	5	6	7	3	3	2016Shevtsova#020	B. abortus	36	72	304/2-15-G (1)	Cattle	2015	Ekpinskiy, Tarbagatays.
4	5	3	12	2	2	3	1	6	43	8	5	6	7	3	3	2016Shevtsova#026	B. abortus	36	72	69AtCam	Camel	1969	Balkuduk, Kurmangazy,.
4	5	3	12	2	2	3	1	6	43	8	4	6	7	3	3	2013Garofolo_12.	B. abortus bv3	36	72	12183	Cattle	2011	Corleto Monforte,Italy
4	5	3	12	2	2	3	1	6	43	8	4	6	7	3	3	2013Garofolo_12.	B. abortus bv3	36	72	12185	Cattle	2011	San Rufo,Italy
4	5	3	12	2	2	3	1	6	43	8	4	6	7	3	3	2013Garofolo_21.	B. abortus bv3	36	72	21571	Cattle	2011	Monte San Giacomo,Italy
4	5	3	12	2	2	3	1	6	43	8	4	6	7	3	3	2013Garofolo_21.	B. abortus bv3	36	72	21675	Cattle	2011	Monte San Giacomo,Italy
4	5	3	12	2	2	3	1	6	43	8	4	6	7	3	3	2013Garofolo_22.	B. abortus bv3	36	72	22839	Buffalo	2011	Albanella,Italy
4	5	3	12	2	2	3	1	6	43	8	4	6	7	3	3	2013Garofolo_22.	B. abortus bv3	36	72	22842	Cattle	2011	Teggiano,Italy
4	5	3	12	2	2	3	1	6	43	8	4	6	7	3	3	2013Garofolo_3636	B. abortus bv3	36	72	3636	Cattle	2011	Monte San Giacomo,Italy
4	5	3	12	2	2	3	1	6	43	8	4	6	7	3	3	2013Garofolo_3916	B. abortus bv3	36	72	3916	Cattle	2011	Monte San Giacomo,Italy
4	5	3	12	2	2	3	1	6	43	8	4	6	7	3	3	2013Garofolo_3920	B. abortus bv3	36	72	3920	Cattle	2011	Teggiano,Italy
4	5	3	12	2	2	3	1	6	43	8	4	6	7	3	3	2013Garofolo_4363	B. abortus bv3	36	72	4363	Cattle	2011	Laurenzana,Italy
4	5	3	12	2	2	3	1	6	43	8	4	6	7	3	3	2013Garofolo_5362	B. abortus bv3	36	72	5362	Buffalo	2011	Monte San Giacomo,Italy
4	5	3	12	2	2	3	1	6	43	8	4	6	7	3	3	2013Garofolo_8980	B. abortus bv3	36	72	8980	Cattle	2011	Teggiano,Italy
4	5	3	12	2	2	3	1	6	43	8	4	6	7	3	3	2013Garofolo_8984	B. abortus bv3	36	72	8984	Cattle	2011	Sassano,Italy
4	5	3	12	2	2	3	1	6	43	8	4	6	7	3	3	2013Jiang#093	B. abortus bv3	36	72	2011166\'	Human	2011	Chongqing, China
4	5	3	12	2	2	3	1	6	43	8	4	6	7	3	3	2013Jiang#094	B. abortus bv3	36	72	YLQ	Human	2006	Zhejiang, China
4	5	3	12	1	2	3	1	6	43	8	4	6	7	3	3	2013Garofolo_5361	B. abortus bv3	113	211	5361	Buffalo	2011	Giffoni Valle Piana,Italy
4	5	3	12	1	2	3	1	6	43	8	4	6	7	3	3	2013Garofolo_6906	B. abortus bv3	113	211	6906	Cattle	2011	Albanella,Italy
4	5	3	12	2	2	3	1	6	43	8	4	6	7	5	3	2013Garofolo_4360	B. abortus bv3	36	72	4360	Cattle	2011	Anzi,Italy
4	5	3	12	2	2	3	1	6	43	8	4	7	5	3	3	2012Ferreira#147	B. abortus bv3	36	72	LNIV-316Ba3-06	Cattle	2006	Alentejo, Portugal
4	5	3	12	2	2	3	1	6	43	8	4	7	5	3	3	2012Ferreira#148	B. abortus bv3	36	72	LNIV-318Ba3-06	Cattle	2006	Alentejo, Portugal
4	5	3	12	2	2	3	1	6	43	8	4	7	5	3	3	2012Ferreira#159	B. abortus bv3	36	72	LNIV-371Ba3-07	Cattle	2007	Beira Interior, Portugal
4	5	3	12	2	2	3	1	6	43	8	4	7	7	3	3	2012Ferreira#146	B. abortus bv3	36	72	LNIV-328Ba3-06	Cattle	2006	Alentejo, Portugal
4	5	3	12	2	2	3	1	6	43	8	4	7	7	3	3	2013Jiang#092	B. abortus bv3	36	72	2011165\	Human	2011	Chongqing, China
4	5	3	12	2	2	3	1	6	43	8	4	8	6	3	3	2016Shevtsova#074	B. abortus	36	72	116/2-15-G (12)	Cattle	2015	Mereyskiy, Taskalinskiy.
4	5	3	12	2	2	3	1	6	43	8	4	8	6	3	3	2016Shevtsova#081	B. abortus	36	72	116/2-15-G (4)	Cattle	2015	Kyzyloba, Zhangalinskiy.
4	5	3	12	2	2	3	1	6	43	8	4	8	13	3	3	2015Shevtsov#026	B. abortus	36	72	B. abortus is1a	Cattle	2013	Aktubek,Alakol,Almaty,.
4	5	3	12	2	2	3	1	6	43	8	4	8	13	3	3	2015Shevtsov#027	B. abortus	36	72	B. abortus is4a	Cattle	2013	Dostyk,Enbekshikazak.
4	5	3	12	2	2	3	1	6	43	8	4	8	13	3	3	2015Shevtsov#028	B. abortus	36	72	B. abortus is2a	Cattle	2013	Zaypak,Alakol,Almaty,.
4	5	3	12	2	2	3	1	6	43	8	4	8	13	3	3	2015Shevtsov#029	B. abortus	36	72	B. abortus is3a	Cattle	2013	Karaturik,Enbekshikaz.
4	5	3	12	2	2	3	1	6	43	8	4	8	7	3	3	2016Shevtsova#018	B. abortus	36	72	82 SR	vaccine		
4	5	3	12	2	2	3	1	6	43	8	4	8	8	3	3	2016Shevtsova#040	B. abortus	36	72	66Al	Cattle	1966	Almaty, Kazakhstan
4	5	3	12	2	2	3	1	6	43	8	4	8	11	3	3	2016Shevtsova#086	B. abortus	36	72	040-15-G	Cattle	2015	Lesnoe, Zhetyzhar, Be.
4	5	3	12	2	2	3	1	6	43	8	4	12	10	3	3	2015Shevtsov#005	B. abortus bv1	36	72	B. abortus 674	Cattle	2011	Fyodorov,Qostanay, Ka.
4	5	3	12	2	2	3	1	6	43	8	4	12	10	3	3	2015Shevtsov#023	B. abortus bv6	36	72	B. abortus 9460	Cattle	2011	t. Rudnyi,Qostanay, Ka.

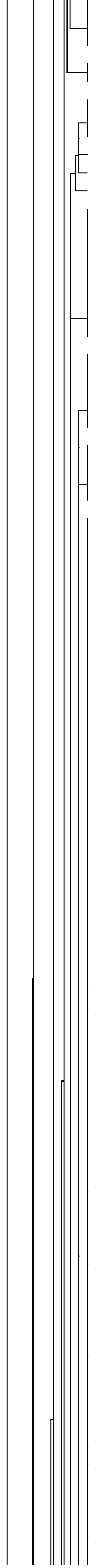

4	5	3	12	2	2	3	1	6	43	8	4	12	10	3	3	2015Shevtsov#025	B. abortus bv6	36	72	B. abortus 96	Cattle	2011	t. Rudnyi,Qostanay, Ka.
4	5	3	12	2	2	3	1	6	43	8	4	12	10	3	3	2015Shevtsov#107	B. abortus bv1	36	72	B. abortus 1169	Cattle	2010	Fyodorov,Qostanay, Ka.
4	5	3	12	2	2	3	1	6	43	8	4	12	10	3	3	2015Shevtsov#124	B. abortus bv6	36	72	B. abortus 1247	Cattle	2011	t. Rudnyi,Qostanay, Ka.
3	5	3	12	2	2	3	1	6	43	8	4	6	4	3	3	2016Shevtsova#089	B. abortus	167	338	113-15-G (10)	Cattle	2015	Karamende bi, Aktogay.
3	5	3	12	2	2	3	1	6	43	8	4	6	4	3	3	2016Shevtsova#090	B. abortus	167	338	113-15-G (1)	Cattle	2015	Zarechnoe, Nurinskyi, .
4	5	3	12	2	2	3	1	6	43	8	5	6	4	3	3	2006LeFleche#119	B. abortus bv7	36	72	BCCN#99-98	Cattle	1999	Mongolia
4	5	3	12	2	2	3	1	6	43	8	5	6	4	3	3	2018Vergnaud#0111	B. abortus bv7	36	72	BCCN#99-98	Cattle	1999	Mongolia
4	5	3	12	2	2	3	1	6	43	8	5	6	4	3	3	2018Vergnaud#0135	B. abortus bv7	36	72	BCCN#99-99	Cattle	1999	Mongolia
4	5	3	12	2	2	3	1	6	43	8	5	5	4	3	3	2006LeFleche#144	B. abortus bv6	36	72	BfR 123	unknown	1972	
4	5	3	12	2	2	3	1	6	43	8	5	13	4	3	3	2016Shevtsova#066	B. abortus	36	72	116/2-15-G (19)	Cattle	2015	Masteksayskiy, Zhangal.
4	5	3	12	2	2	3	1	6	43	8	5	6	3	3	3	2016Shevtsova#042	B. abortus	36	72	70Al	Cattle	1970	Alakol, Almaty, Kazakh.
4	5	3	12	2	2	3	1	6	43	8	5	9	11	3	3	2016Shevtsova#064	B. abortus	36	72	116/2-15-G (21)	Cattle	2015	Koskolskiy, Karatubinsk.
4	5	3	12	2	2	3	1	6	43	8	5	9	11	3	3	2016Shevtsova#068	B. abortus	36	72	116/2-15-G (18)	Cattle	2015	Akzhaikskiy, Terektinski.
4	5	3	12	2	2	3	1	6	43	8	5	9	11	3	3	2016Shevtsova#070	B. abortus	36	72	116/2-15-G (16)	Cattle	2015	Koskolskiy, Karatubinsk.
4	5	3	12	2	2	3	1	6	43	8	5	9	11	3	3	2016Shevtsova#071	B. abortus	36	72	116/2-15-G (15)	Cattle	2015	Koskolskiy, Karatubinsk.
4	5	3	12	2	2	3	1	6	43	8	5	9	11	3	3	2016Shevtsova#072	B. abortus	36	72	116/2-15-G (14)	Cattle	2015	Koskolskiy, Karatubinsk.
4	5	3	12	2	2	3	1	6	43	8	5	9	11	3	3	2016Shevtsova#075	B. abortus	36	72	116/2-15-G (11)	Cattle	2015	Koskolskiy, Karatubinsk.
4	5	3	12	2	2	3	1	6	43	8	5	9	11	3	3	2016Shevtsova#077	B. abortus	36	72	116/2-15-G (8)	Cattle	2015	Mereyskiy, Taskalinskiy.
4	5	3	12	2	2	3	1	6	43	8	5	9	11	3	3	2016Shevtsova#083	B. abortus	36	72	116/2-15-G (2)	Cattle	2015	Koskolskiy, Karatubinsk.
4	5	3	12	2	2	3	1	6	43	8	5	7	6	3	3	2015Shevtsov#016	B. abortus bv1	36	72	B. abortus 706	Cattle	2011	Fyodorov,Qostanay, Ka.
4	5	3	12	2	2	3	1	6	43	8	5	7	6	3	3	2016Shevtsova#033	B. abortus	36	72	65Ak	Cattle	1965	Akmola, Kazakhstan
4	5	3	12	2	2	3	1	6	43	8	5	7	6	3	3	2016Shevtsova#034	B. abortus	36	72	70Ak	Cattle	1970	Celinogradski, Akmola,.
4	5	3	12	2	2	3	1	6	43	8	5	7	6	3	3	2016Shevtsova#045	B. abortus	36	72	48WKR	Cattle	1948	West Kazakhstan Regi.
4	5	3	12	2	2	3	1	6	43	8	5	7	6	3	3	2016Shevtsova#046	B. abortus	36	72	69WKR	Cattle	1969	Terekti, West Kazakhst.
4	5	3	12	2	2	3	1	6	43	8	5	7	8	3	3	2013Jiang#132	B. abortus bv3	36	72	NM1020	Cattle	1989	Inner Mongolia, China
4	5	3	12	2	2	3	1	6	43	8	5	7	8	3	3	2013Jiang#133	B. abortus bv3	36	72	NM1159	Cattle	1989	Inner Mongolia, China
4	5	3	12	2	2	3	1	6	43	8	5	7	8	3	3	2013Jiang#143	B. abortus bv3	36	72	NM1178	Cattle	1990	Inner Mongolia, China
4	5	3	12	2	2	3	1	6	43	8	5	7	8	3	3	2013Jiang#144	B. abortus bv3	36	72	NM1179	Cattle	1990	Inner Mongolia, China
4	5	3	12	2	2	3	1	6	43	8	5	7	10	3	3	2013Jiang#117	B. abortus bv3	36	72	NM1091	Cattle	1985	Inner Mongolia, China
4	5	3	12	2	2	3	1	6	43	8	5	7	10	3	3	2015Shevtsov#001	B. abortus bv3	36	72	B. abortus 64 1	Cattle	2007	Sarybulakskiy,Kerbulak.
4	5	3	12	2	2	3	1	6	43	8	5	7	10	3	3	2015Shevtsov#002	B. abortus bv6	36	72	B. abortus K 4	Cattle	2008	Sosnovka,Auleikol,Qos.
4	5	3	12	2	2	3	1	6	43	8	5	7	10	3	3	2015Shevtsov#003	B. abortus bv1	36	72	B. abortus 671	Cattle	2011	Fyodorov,Qostanay, Ka.
4	5	3	12	2	2	3	1	6	43	8	5	7	10	3	3	2015Shevtsov#004	B. abortus bv1	36	72	B. abortus 672	Cattle	2011	Fyodorov,Qostanay, Ka.
4	5	3	12	2	2	3	1	6	43	8	5	7	10	3	3	2015Shevtsov#006	B. abortus bv1	36	72	B. abortus 679	Cattle	2011	Fyodorov,Qostanay, Ka.
4	5	3	12	2	2	3	1	6	43	8	5	7	10	3	3	2015Shevtsov#007	B. abortus bv1	36	72	B. abortus 683	Cattle	2011	Fyodorov,Qostanay, Ka.
4	5	3	12	2	2	3	1	6	43	8	5	7	10	3	3	2015Shevtsov#008	B. abortus bv1	36	72	B. abortus 687	Cattle	2011	Fyodorov,Qostanay, Ka.
4	5	3	12	2	2	3	1	6	43	8	5	7	10	3	3	2015Shevtsov#009	B. abortus bv1	36	72	B. abortus 687	Cattle	2011	Fyodorov,Qostanay, Ka.
4	5	3	12	2	2	3	1	6	43	8	5	7	10	3	3	2015Shevtsov#010	B. abortus bv1	36	72	B. abortus 688	Cattle	2011	Fyodorov,Qostanay, Ka.
4	5	3	12	2	2	3	1	6	43	8	5	7	10	3	3	2015Shevtsov#011	B. abortus bv1	36	72	B. abortus 690	Cattle	2011	Fyodorov,Qostanay, Ka.
4	5	3	12	2	2	3	1	6	43	8	5	7	10	3	3	2015Shevtsov#012	B. abortus bv1	36	72	B. abortus 691	Cattle	2011	Fyodorov,Qostanay, Ka.
4	5	3	12	2	2	3	1	6	43	8	5	7	10	3	3	2015Shevtsov#013	B. abortus bv6	36	72	B. abortus K 7	Cattle	2008	Sosnovka,Auleikol,Qos.
4	5	3	12	2	2	3	1	6	43	8	5	7	10	3	3	2015Shevtsov#014	B. abortus bv1	36	72	B. abortus 693	Cattle	2011	Fyodorov,Qostanay, Ka.
4	5	3	12	2	2	3	1	6	43	8	5	7	10	3	3	2015Shevtsov#015	B. abortus bv1	36	72	B. abortus 694	Cattle	2011	Fyodorov,Qostanay, Ka.
4	5	3	12	2	2	3	1	6	43	8	5	7	10	3	3	2015Shevtsov#017	B. abortus bv1	36	72	B. abortus 708	Cattle	2011	Fyodorov,Qostanay, Ka.
4	5	3	12	2	2	3	1	6	43	8	5	7	10	3	3	2015Shevtsov#018	B. abortus bv1	36	72	B. abortus 710	Cattle	2011	Fyodorov,Qostanay, Ka.
4	5	3	12	2	2	3	1	6	43	8	5	7	10	3	3	2015Shevtsov#019	B. abortus bv1	36	72	B. abortus 711	Cattle	2011	Fyodorov,Qostanay, Ka.
4	5	3	12	2	2	3	1	6	43	8	5	7	10	3	3	2015Shevtsov#020	B. abortus bv1	36	72	B. abortus 712	Cattle	2011	Fyodorov,Qostanay, Ka.
4	5	3	12	2	2	3	1	6	43	8	5	7	10	3	3	2015Shevtsov#021	B. abortus bv6	36	72	B. abortus 8213	Cattle	2011	t. Rudnyi,Qostanay, Ka.
4	5	3	12	2	2	3	1	6	43	8	5	7	10	3	3	2015Shevtsov#022	B. abortus bv6	36	72	B. abortus 8392	Cattle	2011	t. Rudnyi,Qostanay, Ka.
4	5	3	12	2	2	3	1	6	43	8	5	7	10	3	3	2015Shevtsov#031	B. abortus bv6	36	72	B. abortus K 9	Cattle	2008	Sosnovka,Auleikol,Qos.
4	5	3	12	2	2	3	1	6	43	8	5	7	10	3	3	2015Shevtsov#033	B. abortus bv6	36	72	B. abortus K 27	Cattle	2008	Denisov,Qostanay, Kaz.
4	5	3	12	2	2	3	1	6	43	8	5	7	10	3	3	2015Shevtsov#034	B. abortus bv6	36	72	B. abortus K 41	Cattle	2008	Denisov,Qostanay, Kaz.
4	5	3	12	2	2	3	1	6	43	8	5	7	10	3	3	2015Shevtsov#035	B. abortus bv6	36	72	B. abortus K 50	Cattle	2008	Auleikol,Qostanay, Kaz.
4	5	3	12	2	2	3	1	6	43	8	5	7	10	3	3	2015Shevtsov#037	B. abortus bv6	36	72	B. abortus K 69	Cattle	2008	Auleikol,Qostanay, Kaz.
4	5	3	12	2	2	3	1	6	43	8	5	7	10	3	3	2015Shevtsov#038	B. abortus bv6	36	72	B. abortus K 11	Cattle	2008	Sosnovka,Auleikol,Qos.
4	5	3	12	2	2	3	1	6	43	8	5	7	10	3	3	2015Shevtsov#040	B. abortus bv6	36	72	B. abortus K 71	Cattle	2008	Auleikol,Qostanay,Kaz.
4	5	3	12	2	2	3	1	6	43	8	5	7	10	3	3	2015Shevtsov#041	B. abortus bv6	36	72	B. abortus K 72	Cattle	2008	Auleikol,Qostanay, Kaz.
4	5	3	12	2	2	3	1	6	43	8	5	7	10	3	3	2015Shevtsov#043	B. abortus bv6	36	72	B. abortus K 74	Cattle	2008	Auleikol,Qostanay,Kaz.
4	5	3	12	2	2	3	1	6	43	8	5	7	10	3	3	2015Shevtsov#044	B. abortus bv6	36	72	B. abortus K 75	Cattle	2008	Auleikol,Qostanay, Kaz.
4	5	3	12	2	2	3	1	6	43	8	5	7	10	3	3	2015Shevtsov#046	B. abortus bv6	36	72	B. abortus K 77	Cattle	2008	Denisov,Qostanay, Kaz.
4	5	3	12	2	2	3	1	6	43	8	5	7	10	3	3	2015Shevtsov#047	B. abortus bv6	36	72	B. abortus K 79	Cattle	2008	Denisov,Qostanay, Kaz.
4	5	3	12	2	2	3	1	6	43	8	5	7	10	3	3	2015Shevtsov#049	B. abortus bv6	36	72	B. abortus K 12	Cattle	2008	Sosnovka,Auleikol,Qos.
4	5	3	12	2	2	3	1	6	43	8	5	7	10	3	3	2015Shevtsov#050	B. abortus bv6	36	72	B. abortus K 83	Cattle	2008	Denisov,Qostanay,Kaz.
4	5	3	12	2	2	3	1	6	43	8	5	7	10	3	3	2015Shevtsov#051	B. abortus bv6	36	72	B. abortus K 84	Cattle	2008	Denisov,Qostanay, Kaz.
4	5	3	12	2	2	3	1	6	43	8	5	7	10	3	3	2015Shevtsov#052	B. abortus bv6	36	72	B. abortus K 85	Cattle	2008	Denisov,Qostanay,Kaz.
4	5	3	12	2	2	3	1	6	43	8	5	7	10	3	3	2015Shevtsov#053	B. abortus bv6	36	72	B. abortus K 86	Cattle	2008	Denisov,Qostanay, Kaz.
4	5	3	12	2	2	3	1	6	43	8	5	7	10	3	3	2015Shevtsov#055	B. abortus bv6	36	72	B. abortus K 24	Cattle	2008	Denisov,Qostanay,Kaz.
4	5	3	12	2	2	3	1	6	43	8	5	7	10	3	3	2015Shevtsov#056	B. abortus bv6	36	72	B. abortus K 25	Cattle	2008	Denisov,Qostanay,Kaz.
4	5	3	12	2	2	3	1	6	43	8	5	7	10	3	3	2015Shevtsov#058	B. abortus bv6	36	72	B. abortus K 28	Cattle	2008	Denisov,Qostanay,Kaz.
4	5	3	12	2	2	3	1	6	43	8	5	7	10	3	3	2015Shevtsov#059	B. abortus bv6	36	72	B. abortus K 29	Cattle	2008	Denisov,Qostanay,Kaz.
4	5	3	12	2	2	3	1	6	43	8	5	7	10	3	3	2015Shevtsov#063	B. abortus bv6	36	72	B. abortus K 33	Cattle	2008	Denisov,Qostanay,Kaz.
4	5	3	12	2	2	3	1	6	43	8	5	7	10	3	3	2015Shevtsov#065	B. abortus bv6	36	72	B. abortus K 35	Cattle	2008	Kostanay,Qostanay,Ka.
4	5	3	12	2	2	3	1	6	43	8	5	7	10	3	3	2015Shevtsov#066	B. abortus bv6	36	72	B. abortus K 36	Cattle	2008	Kostanay,Qostanay,Ka.
4	5	3	12	2	2	3	1	6	43	8	5	7	10	3	3	2015Shevtsov#067	B. abortus bv6	36	72	B. abortus K 37	Cattle	2008	Kostanay,Qostanay,Ka.
4	5	3	12	2	2	3	1	6	43	8	5	7	10	3	3	2015Shevtsov#070	B. abortus bv6	36	72	B. abortus K 40	Cattle	2008	Kostanay,Qostanay,Ka.
4	5	3	12	2	2	3	1	6	43	8	5	7	10	3	3	2015Shevtsov#074	B. abortus bv6	36	72	B. abortus K 44	Cattle	2008	Kostanay,Qostanay,Ka.
4	5	3	12	2	2	3	1	6	43	8	5	7	10	3	3	2015Shevtsov#078	B. abortus bv6	36	72	B. abortus K 48	Cattle	2008	Kostanay,Qostanay,Ka.
4	5	3	12	2	2	3	1	6	43	8	5	7	10	3	3	2015Shevtsov#079	B. abortus bv6	36	72	B. abortus K 49	Cattle	2008	Kostanay,Qostanay,Ka.
4	5	3	12	2	2	3	1	6	43	8	5	7	10	3	3	2015Shevtsov#080	B. abortus bv6	36	72	B. abortus K 51	Cattle	2008	Kostanay,Qostanay,Ka.
4	5	3	12	2	2	3	1	6	43	8	5	7	10	3	3	2015Shevtsov#081	B. abortus bv6	36	72	B. abortus K 53	Cattle	2008	Kostanay,Qostanay,Ka.
4	5	3	12	2	2	3	1	6	43	8	5	7	10	3	3	2015Shevtsov#082	B. abortus bv6	36	72	B. abortus K 19	Cattle	2008	Sosnovka,Auleikol,Qos.
4	5	3	12	2	2	3	1	6	43	8	5	7	10	3	3	2015Shevtsov#084	B. abortus bv6	36	72	B. abortus K 55	Cattle	2008	Kostanay,Qostanay,Ka.
4	5	3	12	2	2	3	1	6	43	8	5	7	10	3	3	2015Shevtsov#091	B. abortus bv6	36	72	B. abortus K 63	Cattle	2008	Karasu,Qostanay,Kaza.
4	5	3	12	2	2	3	1	6	43	8	5	7	10	3	3	2015Shevtsov#093	B. abortus bv6	36	72	B. abortus K 2	Cattle	2008	Sosnovka,Auleikol,Qos.
4	5	3	12	2	2	3	1	6	43	8	5	7	10	3	3	2015Shevtsov#095	B. abortus bv6	36	72	B. abortus K 67	Cattle	2008	Karasu,Qostanay,Kaza.
4	5	3	12	2	2	3	1	6	43	8	5	7	10	3	3	2015Shevtsov#096	B. abortus bv6	36	72	B. abortus K 68	Cattle	2008	Karasu,Qostanay,Kaza.

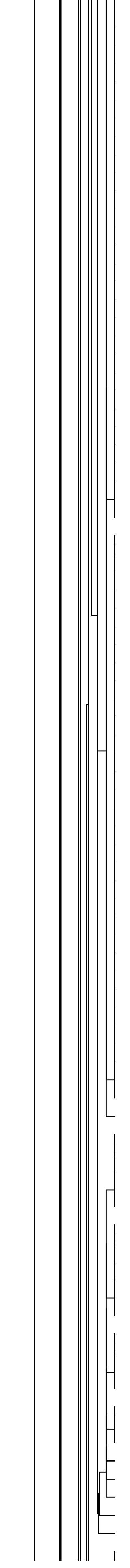

4	5	3	12	2	2	3	1	6	43	8	5	7	10	3	3	2015Shevtsov#097	B. abortus bv6	36	72	B. abortus K 6	Cattle	2009	Sosnovka,Auleikol,Qos.
4	5	3	12	2	2	3	1	6	43	8	5	7	10	3	3	2015Shevtsov#098	B. abortus	36	72	B. abortus Kos.	Cattle	2009	Denisov,Qostanay,Kaz.
4	5	3	12	2	2	3	1	6	43	8	5	7	10	3	3	2015Shevtsov#099	B. abortus	36	72	B. abortus Kos.	Cattle	2009	Denisov,Qostanay,Kaz.
4	5	3	12	2	2	3	1	6	43	8	5	7	10	3	3	2015Shevtsov#100	B. abortus bv1	36	72	B. abortus 1126	Cattle	2009	Fyodorov,Qostanay,Ka.
4	5	3	12	2	2	3	1	6	43	8	5	7	10	3	3	2015Shevtsov#101	B. abortus	36	72	B. abortus Kos.	Cattle	2009	Denisov,Qostanay, Kaz.
4	5	3	12	2	2	3	1	6	43	8	5	7	10	3	3	2015Shevtsov#102	B. abortus	36	72	B. abortus Kos.	Cattle	2009	Denisov,Qostanay, Kaz.
4	5	3	12	2	2	3	1	6	43	8	5	7	10	3	3	2015Shevtsov#103	B. abortus	36	72	B. abortus Kos.	Cattle	2009	Denisov,Qostanay,Kaz.
4	5	3	12	2	2	3	1	6	43	8	5	7	10	3	3	2015Shevtsov#105	B. abortus bv1	36	72	B. abortus 1058	Cattle	2010	Fyodorov,Qostanay, Ka.
4	5	3	12	2	2	3	1	6	43	8	5	7	10	3	3	2015Shevtsov#106	B. abortus bv1	36	72	B. abortus 1160	Cattle	2010	Fyodorov,Qostanay, Ka.
4	5	3	12	2	2	3	1	6	43	8	5	7	10	3	3	2015Shevtsov#108	B. abortus bv1	36	72	B. abortus 1950	Cattle	2010	Fyodorov,Qostanay, Ka.
4	5	3	12	2	2	3	1	6	43	8	5	7	10	3	3	2015Shevtsov#109	B. abortus bv1	36	72	B. abortus 38	Cattle	2010	Fyodorov,Qostanay, Ka.
4	5	3	12	2	2	3	1	6	43	8	5	7	10	3	3	2015Shevtsov#110	B. abortus bv1	36	72	B. abortus 38 42	Cattle	2010	Fyodorov,Qostanay, Ka.
4	5	3	12	2	2	3	1	6	43	8	5	7	10	3	3	2015Shevtsov#111	B. abortus bv1	36	72	B. abortus 4253	Cattle	2010	Fyodorov,Qostanay, Ka.
4	5	3	12	2	2	3	1	6	43	8	5	7	10	3	3	2015Shevtsov#112	B. abortus bv1	36	72	B. abortus 43 62	Cattle	2010	Fyodorov,Qostanay, Ka.
4	5	3	12	2	2	3	1	6	43	8	5	7	10	3	3	2015Shevtsov#113	B. abortus bv1	36	72	B. abortus 1230	Cattle	2011	Fyodorov,Qostanay, Ka.
4	5	3	12	2	2	3	1	6	43	8	5	7	10	3	3	2015Shevtsov#114	B. abortus bv1	36	72	B. abortus 1238	Cattle	2011	Fyodorov,Qostanay, Ka.
4	5	3	12	2	2	3	1	6	43	8	5	7	10	3	3	2015Shevtsov#115	B. abortus bv6	36	72	B. abortus K 3	Cattle	2008	Sosnovka,Auleikol,Qos.
4	5	3	12	2	2	3	1	6	43	8	5	7	10	3	3	2015Shevtsov#116	B. abortus bv1	36	72	B. abortus 1057	Cattle	2011	Fyodorov,Qostanay, Ka.
4	5	3	12	2	2	3	1	6	43	8	5	7	10	3	3	2015Shevtsov#117	B. abortus bv1	36	72	B. abortus 1073	Cattle	2011	Fyodorov,Qostanay, Ka.
4	5	3	12	2	2	3	1	6	43	8	5	7	10	3	3	2015Shevtsov#118	B. abortus bv1	36	72	B. abortus 1077	Cattle	2011	Fyodorov,Qostanay, Ka.
4	5	3	12	2	2	3	1	6	43	8	5	7	10	3	3	2015Shevtsov#119	B. abortus bv1	36	72	B. abortus 1087	Cattle	2011	Fyodorov,Qostanay, Ka.
4	5	3	12	2	2	3	1	6	43	8	5	7	10	3	3	2015Shevtsov#120	B. abortus bv1	36	72	B. abortus 1097	Cattle	2011	Fyodorov,Qostanay, Ka.
4	5	3	12	2	2	3	1	6	43	8	5	7	10	3	3	2015Shevtsov#121	B. abortus bv6	36	72	B. abortus 1170	Cattle	2011	t. Rudnyi,Qostanay, Ka.
4	5	3	12	2	2	3	1	6	43	8	5	7	10	3	3	2015Shevtsov#122	B. abortus bv1	36	72	B. abortus 1229	Cattle	2011	Fyodorov,Qostanay, Ka.
4	5	3	12	2	2	3	1	6	43	8	5	7	10	3	3	2015Shevtsov#123	B. abortus bv1	36	72	B. abortus 1242	Cattle	2011	Fyodorov,Qostanay, Ka.
4	5	3	12	2	2	3	1	6	43	8	5	7	10	3	3	2015Shevtsov#125	B. abortus bv1	36	72	B. abortus 670	Cattle	2011	Fyodorov,Qostanay, Ka.
4	5	3	12	2	2	3	1	6	43	8	5	7	10	3	3	2016Shevtsova#023	B. abortus	36	72	52Al	Cattle	1952	Eskeldi, Almaty, Kazakh.
4	5	3	12	2	2	3	1	6	43	8	5	7	10	3	3	2016Shevtsova#025	B. abortus	36	72	69At	Cattle	1969	Balyqshy, Kurmangazy,.
4	5	3	12	2	2	3	1	6	43	8	5	7	10	3	3	2016Shevtsova#041	B. abortus	36	72	57Al	Cattle	1957	Almaty, Kazakhstan
4	5	3	12	2	2	3	1	6	43	8	5	7	9	3	3	2013Jiang#109	B. abortus bv3	36	72	NM1070	Ovine	1985	Inner Mongolia, China
4	5	3	12	2	2	3	1	6	43	8	5	7	9	3	3	2015Shevtsov#024	B. abortus bv6	36	72	B. abortus K 8	Cattle	2008	Sosnovka,Auleikol,Qos.
4	5	3	12	2	2	3	1	6	43	8	5	7	9	3	3	2015Shevtsov#032	B. abortus bv6	36	72	B. abortus K 23	Cattle	2008	Denisov,Qostanay,Kaz.
4	5	3	12	2	2	3	1	6	43	8	5	7	9	3	3	2015Shevtsov#036	B. abortus bv6	36	72	B. abortus K 65	Cattle	2008	Auleikol,Qostanay, Kaz.
4	5	3	12	2	2	3	1	6	43	8	5	7	9	3	3	2015Shevtsov#039	B. abortus bv6	36	72	B. abortus K 70	Cattle	2008	Auleikol,Qostanay, Kaz.
4	5	3	12	2	2	3	1	6	43	8	5	7	9	3	3	2015Shevtsov#042	B. abortus bv6	36	72	B. abortus K 73	Cattle	2008	Auleikol,Qostanay, Kaz.
4	5	3	12	2	2	3	1	6	43	8	5	7	9	3	3	2015Shevtsov#045	B. abortus bv6	36	72	B. abortus K 76	Cattle	2008	Auleikol,Qostanay,Kaz.
4	5	3	12	2	2	3	1	6	43	8	5	7	9	3	3	2015Shevtsov#048	B. abortus bv6	36	72	B. abortus K 80	Cattle	2008	Denisov,Qostanay, Kaz.
4	5	3	12	2	2	3	1	6	43	8	5	7	9	3	3	2015Shevtsov#054	B. abortus bv6	36	72	B. abortus K 87	Cattle	2008	Denisov,Qostanay, Kaz.
4	5	3	12	2	2	3	1	6	43	8	5	7	9	3	3	2015Shevtsov#057	B. abortus bv6	36	72	B. abortus K 26	Cattle	2008	Denisov,Qostanay,Kaz.
4	5	3	12	2	2	3	1	6	43	8	5	7	9	3	3	2015Shevtsov#060	B. abortus bv6	36	72	B. abortus K 15	Cattle	2008	Sosnovka,Auleikol,Qos.
4	5	3	12	2	2	3	1	6	43	8	5	7	9	3	3	2015Shevtsov#061	B. abortus bv6	36	72	B. abortus K 31	Cattle	2008	Denisov,Qostanay,Kaz.
4	5	3	12	2	2	3	1	6	43	8	5	7	9	3	3	2015Shevtsov#062	B. abortus bv6	36	72	B. abortus K 32	Cattle	2008	Denisov,Qostanay,Kaz.
4	5	3	12	2	2	3	1	6	43	8	5	7	9	3	3	2015Shevtsov#064	B. abortus bv6	36	72	B. abortus K 34	Cattle	2008	Denisov,Qostanay,Kaz.
4	5	3	12	2	2	3	1	6	43	8	5	7	9	3	3	2015Shevtsov#068	B. abortus bv6	36	72	B. abortus K 38	Cattle	2008	Kostanay,Qostanay,Ka.
4	5	3	12	2	2	3	1	6	43	8	5	7	9	3	3	2015Shevtsov#069	B. abortus bv6	36	72	B. abortus K 39	Cattle	2008	Kostanay,Qostanay,Ka.
4	5	3	12	2	2	3	1	6	43	8	5	7	9	3	3	2015Shevtsov#071	B. abortus bv6	36	72	B. abortus K 17	Cattle	2008	Sosnovka,Auleikol,Qos.
4	5	3	12	2	2	3	1	6	43	8	5	7	9	3	3	2015Shevtsov#072	B. abortus bv6	36	72	B. abortus K 42	Cattle	2008	Kostanay,Qostanay,Ka.
4	5	3	12	2	2	3	1	6	43	8	5	7	9	3	3	2015Shevtsov#073	B. abortus bv6	36	72	B. abortus K 43	Cattle	2008	Kostanay,Qostanay,Ka.
4	5	3	12	2	2	3	1	6	43	8	5	7	9	3	3	2015Shevtsov#075	B. abortus bv6	36	72	B. abortus K 45	Cattle	2008	Kostanay,Qostanay,Ka.
4	5	3	12	2	2	3	1	6	43	8	5	7	9	3	3	2015Shevtsov#076	B. abortus bv6	36	72	B. abortus K 46	Cattle	2008	Kostanay,Qostanay,Ka.
4	5	3	12	2	2	3	1	6	43	8	5	7	9	3	3	2015Shevtsov#077	B. abortus bv6	36	72	B. abortus K 47	Cattle	2008	Kostanay,Qostanay,Ka.
4	5	3	12	2	2	3	1	6	43	8	5	7	9	3	3	2015Shevtsov#083	B. abortus bv6	36	72	B. abortus K 54	Cattle	2008	Kostanay,Qostanay,Ka.
4	5	3	12	2	2	3	1	6	43	8	5	7	9	3	3	2015Shevtsov#085	B. abortus bv6	36	72	B. abortus K 56	Cattle	2008	Kostanay,Qostanay,Ka.
4	5	3	12	2	2	3	1	6	43	8	5	7	9	3	3	2015Shevtsov#086	B. abortus bv6	36	72	B. abortus K 58	Cattle	2008	Kostanay,Qostanay,Ka.
4	5	3	12	2	2	3	1	6	43	8	5	7	9	3	3	2015Shevtsov#087	B. abortus bv6	36	72	B. abortus K 59	Cattle	2008	Kostanay,Qostanay,Ka.
4	5	3	12	2	2	3	1	6	43	8	5	7	9	3	3	2015Shevtsov#088	B. abortus bv6	36	72	B. abortus K 60	Cattle	2008	Karasu,Qostanay,Kaza.
4	5	3	12	2	2	3	1	6	43	8	5	7	9	3	3	2015Shevtsov#089	B. abortus bv6	36	72	B. abortus K 61	Cattle	2008	Karasu,Qostanay,Kaza.
4	5	3	12	2	2	3	1	6	43	8	5	7	9	3	3	2015Shevtsov#090	B. abortus bv6	36	72	B. abortus K 62	Cattle	2008	Karasu,Qostanay,Kaza.
4	5	3	12	2	2	3	1	6	43	8	5	7	9	3	3	2015Shevtsov#092	B. abortus bv6	36	72	B. abortus K 64	Cattle	2008	Karasu,Qostanay,Kaza.
4	5	3	12	2	2	3	1	6	43	8	5	7	9	3	3	2015Shevtsov#094	B. abortus bv6	36	72	B. abortus K 66	Cattle	2008	Karasu,Qostanay,Kaza.
4	5	3	12	2	2	3	1	6	43	8	5	7	9	3	3	2015Shevtsov#104	B. abortus bv6	36	72	B. abortus K 20	Cattle	2008	Sosnovka,Auleikol,Qos.
4	5	3	12	2	2	3	1	6	43	8	5	7	5	3	3	2016Shevtsova#044	B. abortus	36	72	57Al	Cattle	1957	Almaty, Kazakhstan
4	5	3	12	2	2	3	1	6	43	8	5	4	5	3	3	2013Jiang#136	B. abortus bv3	36	72	NM1170	Cattle	1989	Inner Mongolia, China
4	5	3	12	2	2	3	1	6	43	8	5	4	5	3	3	2013Jiang#137	B. abortus bv3	36	72	NM1171	Cattle	1989	Inner Mongolia, China
4	5	3	12	2	2	3	1	6	43	8	5	4	5	3	3	2013Jiang#138	B. abortus bv3	36	72	NM1172	Cattle	1989	Inner Mongolia, China
4	5	3	12	2	2	3	1	6	43	8	5	4	5	3	3	2013Jiang#139	B. abortus bv3	36	72	NM1173	Cattle	1989	Inner Mongolia, China
4	5	3	12	2	2	3	1	6	43	8	5	4	5	3	3	2016Shevtsova#021	B. abortus	36	72	243-15-G	Cattle	2015	Ushtobe, BucharZhyrau.
4	5	3	12	2	2	3	1	6	43	8	5	4	7	3	3	2013Jiang#125	B. abortus bv3	36	72	NM1140	Cattle	1988	Inner Mongolia, China
4	5	3	12	2	2	3	1	6	43	8	5	4	7	3	3	2013Jiang#126	B. abortus bv3	36	72	NM1146	Cattle	1988	Inner Mongolia, China
4	5	3	12	2	2	3	1	6	43	8	5	4	7	3	3	2013Jiang#128	B. abortus bv3	36	72	NM1148	Cattle	1988	Inner Mongolia, China
4	5	3	12	2	2	3	1	6	43	8	5	4	7	3	3	2013Jiang#141	B. abortus bv3	36	72	NM1176	Cattle	1990	Inner Mongolia, China
4	5	3	12	2	2	3	1	6	43	8	5	4	7	3	3	2013Jiang#150	B. abortus bv3	36	72	NM1215	Cattle	1994	Inner Mongolia, China
4	5	3	12	2	2	3	1	6	43	8	5	4	7	3	3	2013Jiang#151	B. abortus bv3	36	72	NM1218	Cattle	1995	Inner Mongolia, China
4	5	3	12	2	2	3	1	6	43	8	5	4	6	3	3	2013Jiang#121	B. abortus bv3	36	72	NM1134	Cattle	1988	Inner Mongolia, China
4	5	3	12	2	2	3	1	6	43	8	5	4	6	3	3	2013Jiang#122	B. abortus bv3	36	72	NM1135	Cattle	1988	Inner Mongolia, China
4	5	3	12	2	2	3	1	6	43	8	5	4	6	3	3	2013Jiang#123	B. abortus bv3	36	72	NM1136	Cattle	1988	Inner Mongolia, China
4	5	3	12	2	2	3	1	6	43	8	5	4	6	3	3	2013Jiang#124	B. abortus bv3	36	72	NM1137	Cattle	1988	Inner Mongolia, China
4	5	3	12	2	2	3	1	6	43	8	5	4	8	3	3	2013Jiang#107	B. abortus bv3	36	72	NM1067	Cattle	1985	Inner Mongolia, China
4	5	3	12	2	2	3	1	6	43	8	5	4	8	3	3	2013Jiang#120	B. abortus bv3	36	72	NM1133	Cattle	1988	Inner Mongolia, China
4	5	3	12	2	2	3	1	6	43	8	5	4	8	3	3	2013Jiang#142	B. abortus bv3	36	72	NM1177	Cattle	1990	Inner Mongolia, China
4	5	3	12	2	2	3	1	6	43	8	5	4	4	3	3	2013Jiang#106	B. abortus bv3	36	72	NM1066	Cattle	1985	Inner Mongolia, China
4	5	3	12	2	2	3	1	6	43	8	5	4	10	3	3	2013Jiang#145	B. abortus bv3	36	72	NM1181	Cattle	1990	Inner Mongolia, China
4	5	3	12	2	2	3	1	6	43	8	5	4	12	3	3	2013Jiang#147	B. abortus bv3	36	72	NM1200	Cattle	1992	Inner Mongolia, China
4	5	3	12	2	2	3	1	6	43	9	5	4	6	3	3	2013Jiang#135	B. abortus bv3	36	330	NM1169	Cattle	1989	Inner Mongolia, China
4	5	2	12	2	2	3	1	6	43	8	5	4	8	3	3	2013Jiang#101	B. abortus bv3	116	331	NM1055	Cattle	1984	Inner Mongolia, China
4	5	3	12	2	2	3	1	6	43	8	3	3	7	3	3	2016Shevtsova#029	B. abortus	36	72	59Al	Cattle	1959	Almaty, Kazakhstan

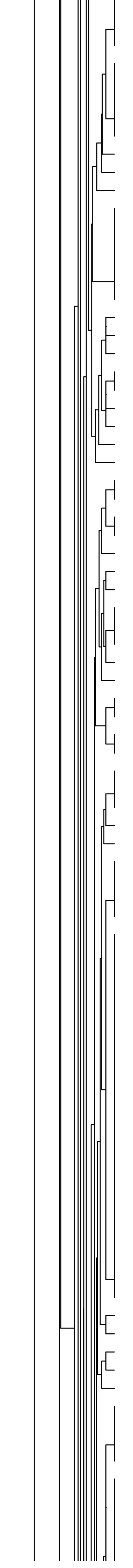

4	5	3	12	2	2	3	1	6	43	8	3	3	7	3	3	2016Shevtsova#032	B. abortus	36	72		69WKR	Cattle	1969	Terekti, West Kazakhst.
4	5	3	12	2	2	3	1	6	43	8	3	3	7	3	3	2016Shevtsova#039	B. abortus	36	72		68AI	Cattle	1968	Enbekshikazakh, Almaty.
4	5	3	12	2	2	3	1	6	43	8	3	3	7	3	3	2016Shevtsova#043	B. abortus	36	72		67WKR	Cattle	1967	West Kazakhstan Regi.
4	5	3	12	2	2	3	1	6	43	8	3	5	7	3	3	2013Garofolo_11.	B. abortus bv3	36	72		11786	Cattle	2011	Priolo Gargallo,Italy
4	5	3	12	2	2	3	1	6	43	8	3	5	7	3	3	2013Garofolo_11.	B. abortus bv3	36	72		11795	Cattle	2011	Ragusa,Italy
4	5	3	12	2	2	3	1	6	43	8	3	5	7	3	3	2013Garofolo_11.	B. abortus bv3	36	72		11796	Cattle	2011	Tusa,Italy
4	5	3	12	2	2	3	1	6	43	8	3	5	7	3	3	2013Garofolo_11.	B. abortus bv3	36	72		11807	Cattle	2011	Pettineo,Italy
4	5	3	12	2	2	3	1	6	43	8	3	5	7	3	3	2013Garofolo_11.	B. abortus bv3	36	72		11809	Cattle	2011	Ucria,Italy
4	5	3	12	2	2	3	1	6	43	8	5	5	7	3	3	2013Jiang#152	B. abortus bv3	36	72		NM1219	Cattle	1995	Inner Mongolia, China
4	5	3	12	2	2	3	1	6	43	8	3	5	8	3	3	2006LeFleche#115	B. abortus bv3	36	72		BCCN#95-34	Cattle	1995	Sicilia, Italia
4	5	3	12	2	2	3	1	6	43	8	3	6	9	3	3	2013Garofolo_13.	B. abortus bv3	36	72		13544	Ovine	2011	Campo Tindaro,Italy
4	5	3	12	2	2	3	1	7	43	8	6	5	7	3	3	2016Shevtsova#065	B. abortus	36	70		116/2-15-G (20)	Cattle	2015	Budarinskiy, Akzhaikskiy.
4	5	3	12	2	2	3	1	7	43	8	6	5	7	3	3	2016Shevtsova#069	B. abortus	36	70		116/2-15-G (17)	Cattle	2015	Mereyskiy, Taskalinskiy.
4	5	3	12	2	2	3	1	7	43	8	6	5	7	3	3	2016Shevtsova#078	B. abortus	36	70		116/2-15-G (7)	Cattle	2015	Saychinskiy, Bokeiordi.
4	5	3	12	2	2	3	1	7	43	8	6	5	7	3	3	2016Shevtsova#079	B. abortus	36	70		116/2-15-G (6)	Cattle	2015	Mereyskiy, Taskalinskiy.
4	5	3	12	2	2	3	1	7	43	8	6	5	7	3	3	2016Shevtsova#080	B. abortus	36	70		116/2-15-G (5)	Cattle	2015	Mereyskiy, Taskalinskiy.
4	5	3	12	2	2	3	1	7	43	8	6	5	7	3	3	2016Shevtsova#084	B. abortus	36	70		116/2-15-G (1)	Cattle	2015	Mereyskiy, Taskalinskiy.
4	5	3	12	2	2	3	1	6	43	8	6	4	8	3	3	2013Jiang#100	B. abortus bv3	36	72		NM1051	Cattle	1984	Inner Mongolia, China
4	5	3	12	2	2	3	1	6	43	8	6	4	5	3	3	2013Jiang#146	B. abortus bv3	36	72		NM1185	Cattle	1990	Inner Mongolia, China
4	5	3	12	2	2	3	1	6	43	8	6	4	4	3	3	2013Jiang#130	B. abortus bv3	36	72		NM1156	Ovine	1988	Inner Mongolia, China
4	5	3	12	2	2	3	1	6	43	8	6	4	7	3	3	2013Jiang#131	B. abortus bv3	36	72		NM1158	Cattle	1988	Inner Mongolia, China
4	5	3	12	2	2	3	1	6	43	8	6	4	7	3	3	2013Jiang#140	B. abortus bv3	36	72		NM1175	Cattle	1990	Inner Mongolia, China
4	5	3	12	2	2	3	1	6	43	8	6	6	7	3	3	GCA_001043295	B. abortus	36	72	96	BAB8416			
4	5	3	12	2	2	3	1	6	43	8	6	7	7	3	3	2013Jiang#108	B. abortus bv3	36	72		NM1068	Cattle	1985	Inner Mongolia, China
4	5	3	12	2	2	3	1	6	43	8	6	7	6	3	3	2006LeFleche#203	B. abortus bv7	36	72		BCCN#04-1	unknown	2004	Turkey
4	5	3	12	2	2	3	2	6	43	8	6	4	4	3	3	2013Jiang#105	B. abortus bv3	117	328		NM1065	Cattle	1985	Inner Mongolia, China
4	5	3	12	2	2	3	1	6	43	8	4	5	6	3	3	2013Jiang#087	B. abortus bv3	36	72			Cattle	2011	Hebei, China
4	5	3	12	2	2	3	1	6	43	8	4	5	6	3	3	2013Jiang#088	B. abortus bv3	36	72			Cattle	2011	Hebei, China
4	5	3	12	2	2	3	1	6	43	8	3	5	6	3	3	2013Garofolo_11.	B. abortus bv3	36	72		11783	Cattle	2011	San Mauro Castel verde.
4	5	3	12	2	2	3	1	6	43	8	3	5	6	3	3	2013Garofolo_11.	B. abortus bv3	36	72		11813	Cattle	2011	Catania,Italy
4	5	3	12	2	2	3	1	6	36	8	4	5	6	3	3	2013Jiang#085	B. abortus bv3	36	332			Cattle	2011	Hebei, China
4	5	3	12	2	2	3	1	6	43	8	4	5	4	3	3	2006LeFleche#108	B. abortus bv3	36	72		BCCN#91-90	Cattle	1991	Greece
4	5	3	12	2	2	3	1	6	43	8	4	5	11	3	3	2013Jiang#084	B. abortus bv3	36	72			Cattle	2011	Xinjiang, China
4	5	3	12	2	2	3	1	6	43	8	4	5	5	3	3	2006LeFleche#114	B. abortus bv3	36	72		BCCN#95-7	Cattle	1995	Limoges, France
4	5	3	12	2	2	3	1	6	43	8	4	5	5	3	3	2013Jiang#086	B. abortus bv3	36	72			Cattle	2011	Hebei, China
4	5	3	12	2	2	3	1	6	43	8	4	5	5	3	3	2018Vergnaud#0112	B. abortus atypi.	36	72		BCCN#R26	Cattle		Germany
4	5	3	12	2	2	3	1	6	43	8	7	5	5	3	3	2016Shevtsova#067	B. abortus	36	72		052/1-15-G	Cattle	2015	Rasvet, Kyzyljarskii, No.
4	5	3	12	2	2	3	1	4	43	8	4	5	5	3	3	2016Shevtsova#031	B. abortus	36	340		1947	Cattle	1947	Kaskelen, Almaty, Kara.
4	5	3	12	2	2	3	1	6	43	8	5	5	5	3	3	2013Jiang#091	B. abortus bv3	36	72		2011163\'	Cattle	2011	Xinjiang, China
4	5	3	12	2	2	3	1	6	43	8	5	5	5	3	3	2018Vergnaud#0736	B. abortus bv3	36	72		BCCN#87-15	Cattle	1987	Savoie, France
4	5	3	12	2	2	3	1	6	43	8	5	5	5	4	3	2013Garofolo_1161	B. abortus bv3	36	72		1161	Cattle	2011	Mesoraca,Italy
4	5	3	12	2	2	3	1	6	43	8	5	5	5	4	3	2013Garofolo_6463	B. abortus bv3	36	72		6463	Cattle	2011	Umbriatico,Italy
4	5	3	12	2	2	3	1	6	43	8	4	5	5	4	3	2006LeFleche#109	B. abortus bv3	36	72		BCCN#92-25	Cattle	1992	La Roche-sur-Yon, Fra.
4	5	3	12	2	2	3	1	6	43	8	4	5	5	4	3	2012Ferreira#163	B. abortus bv3	36	72		LNIV-122Ba3-03	Cattle	2003	Entre Douro e Minho, P.
4	5	3	12	2	2	3	1	6	43	8	4	5	5	4	3	2013Garofolo_1965	B. abortus bv3	36	72		1965	Cattle	2011	Roccaraso,Italy
4	5	3	12	2	2	3	1	6	43	8	4	5	5	4	4	2018Vergnaud#0884	B. abortus atypi.	36	72		BCCN#00-6	Cattle	2000	Navarre, Spain
4	5	3	12	2	2	3	1	6	43	8	4	4	5	4	3	2013Garofolo_6060	B. abortus bv3	36	72		6060	Cattle	2011	San Marco Argentano,It.
4	5	3	12	2	2	3	1	6	43	8	5	5	6	4	3	2013Garofolo_12.	B. abortus bv3	36	72		12855	Cattle	2011	Campana,Italy
4	5	3	12	2	2	3	1	6	43	8	5	5	6	4	3	2013Garofolo_6902	B. abortus bv3	36	72		6902	Cattle	2011	Cagnano Varano,Italy
4	5	3	12	2	2	3	1	6	43	8	5	5	6	4	3	2013Garofolo_6903	B. abortus bv3	36	72		6903	Cattle	2011	Vico del Gargano,Italy
4	5	3	12	2	2	3	1	6	43	8	5	5	6	4	3	2013Garofolo_6905	B. abortus bv3	36	72		6905	Cattle	2011	Campana,Italy
4	5	3	12	2	2	3	1	6	43	8	4	5	6	4	3	2006LeFleche#110	B. abortus bv3	36	72		BCCN#93-15	Cattle	1993	Pamplona, Spain
4	5	3	12	2	2	3	1	6	43	8	4	5	6	4	3	2012Ferreira#155	B. abortus bv3	36	72		LNIV-181Ba3-04	Cattle	2004	Beira Interior, Portugal
4	5	3	12	2	2	3	1	6	43	8	4	5	6	4	3	2012Ferreira#156	B. abortus bv3	36	72		LNIV-329Ba3-07	Cattle	2007	Beira Interior, Portugal
4	5	3	12	2	2	3	1	6	43	8	4	5	6	4	3	2012Ferreira#157	B. abortus bv3	36	72		LNIV-77Ba3-03	Cattle	2003	Beira Interior, Portugal
4	5	3	12	2	2	3	1	6	43	8	4	5	6	4	3	2012Ferreira#162	B. abortus bv3	36	72		LNIV-P24Ba3/04	Cattle	2004	Beira Litoral, Portugal
4	5	3	12	2	2	3	1	6	43	8	4	5	6	4	3	2012Ferreira#165	B. abortus bv3	36	72		LNIV-P25Ba3/04	Cattle	2004	Entre Douro e Minho, P.
4	5	3	12	2	2	3	1	6	43	8	4	5	6	4	3	2012Ferreira#166	B. abortus bv3	36	72		LNIV-P26Ba3/04	Cattle	2004	Entre Douro e Minho, P.
4	5	3	12	2	2	3	1	6	43	8	4	5	6	4	3	2012Ferreira#170	B. abortus bv3	36	72		LNIV-P30Ba3/04	Cattle	2004	Tras-os-Montes, Portugal
4	5	3	12	2	2	3	1	6	43	8	4	5	6	4	3	2012Ferreira#171	B. abortus bv3	36	72		LNIV-P31Ba3/03	Cattle	2003	Tras-os-Montes, Portugal
4	5	3	12	2	2	3	1	6	43	8	4	5	6	4	3	2012Ferreira#172	B. abortus bv3	36	72		LNIV-P32Ba3/03	Cattle	2003	Tras-os-Montes, Portugal
4	5	3	12	2	2	3	1	6	43	8	4	5	6	4	3	2012Ferreira#173	B. abortus bv3	36	72		LNIV-P35Ba3/04	Cattle	2004	Tras-os-Montes, Portugal
4	5	3	12	2	2	3	1	6	43	8	4	5	6	4	3	2013Garofolo_1114	B. abortus bv3	36	72		1114	Cattle	2011	Agnone,Italy
4	5	3	12	2	2	3	1	6	43	8	4	5	6	4	3	2013Garofolo_1115	B. abortus bv3	36	72		1115	Cattle	2011	Agnone,Italy
4	5	3	12	2	2	3	1	6	43	8	4	5	6	4	3	2013Garofolo_1365	B. abortus bv3	36	72		1365	Cattle	2011	Carovilli,Italy
4	5	3	12	2	2	3	1	6	43	8	4	5	6	4	3	2013Garofolo_1565	B. abortus bv3	36	72		1565	Cattle	2011	Agnone,Italy
4	5	3	12	2	2	3	1	6	43	8	4	5	6	4	3	2013Garofolo_2081	B. abortus bv3	36	72		2081	Cattle	2011	Agnone,Italy
4	5	3	12	2	2	3	1	6	43	8	4	5	6	4	3	2013Garofolo_2873	B. abortus bv3	36	72		2873	Cattle	2011	Agnone,Italy
4	5	3	12	2	2	3	1	6	43	8	4	5	6	4	3	2013Garofolo_6899	B. abortus bv3	36	72		6899	Cattle	2011	Carpino,Italy
4	5	3	12	2	2	3	1	6	43	8	4	5	6	4	3	2013Garofolo_6900	B. abortus bv3	36	72		6900	Cattle	2011	San Nicandro Garganico.
4	5	3	12	2	2	3	1	6	43	8	4	5	6	4	3	2013Garofolo_6904	B. abortus bv3	36	72		6904	Cattle	2011	Ariola,Italy
4	5	3	12	2	2	3	1	6	43	8	4	5	6	4	3	2013Garofolo_8985	B. abortus bv3	36	72		8985	Cattle	2011	San Nicandro Garganico.
4	5	3	12	2	2	3	1	6	43	8	4	5	12	4	3	2012Ferreira#168	B. abortus bv3	36	72		LNIV-2Ba3-01	Cattle	2001	Tras-os-Montes, Portugal
4	5	3	12	2	2	3	1	6	43	8	4	7	12	4	3	2012Ferreira#169	B. abortus bv3	36	72		LNIV-3Ba3-01	Cattle	2001	Tras-os-Montes, Portugal
4	5	3	12	2	2	3	1	7	43	8	4	5	6	4	3	2006LeFleche#141	B. abortus bv3	36	70		BfR 101	Cattle	1998	Rostock, Germany
4	5	3	12	2	2	3	1	7	43	8	4	5	7	4	3	2006LeFleche#143	B. abortus bv3	36	70		BfR 107	unknown	1998	Frankfurt/O., Germany
4	5	3	12	2	2	3	1	7	43	8	4	6	6	4	3	2006LeFleche#142	B. abortus bv3	36	70		BfR 106	Cattle	1998	Frankfurt/O., Germany
4	5	3	12	2	2	3	1	6	43	8	4	5	8	5	3	2012Ferreira#161	B. abortus bv3	36	72		LNIV-P23Ba3/03	Cattle	2003	Beira Litoral, Portugal
4	5	3	12	2	2	3	1	6	43	8	4	5	8	5	3	2012Ferreira#167	B. abortus bv3	36	72		LNIV-P27Ba3/03	Cattle	2003	Entre Douro e Minho, P.
4	5	3	12	2	2	3	1	6	43	8	4	5	8	5	3	2013Garofolo_6901	B. abortus bv3	36	72		6901	Cattle	2011	Campana,Italy
4	5	3	12	2	2	3	1	6	43	8	4	5	8	5	3	2013Garofolo_9061	B. abortus bv3	36	72		9061	Cattle	2011	Campana,Italy
4	5	3	12	2	2	3	1	6	43	8	4	5	6	5	3	2012Ferreira#158	B. abortus bv3	36	72		LNIV-370Ba3-07	Cattle	2007	Beira Interior, Portugal
4	5	3	12	2	2	3	1	6	43	8	4	5	6	5	3	2013Garofolo_11.	B. abortus bv3	36	72		11393	Cattle	2011	Castrovillari,Italy
4	5	3	12	2	2	3	1	6	43	8	4	5	6	5	3	2013Garofolo_15.	B. abortus bv3	36	72		15074	Cattle	2011	San Marco in Lamis,Italy
4	5	3	12	2	2	3	1	6	43	8	4	5	6	5	3	2013Garofolo_15.	B. abortus bv3	36	72		15081	Cattle	2011	Cagnano Varano,Italy
4	5	3	12	2	2	3	1	6	43	8	4	5	6	5	3	2013Garofolo_19.	B. abortus bv3	36	72		19433	Cattle	2011	Campana,Italy

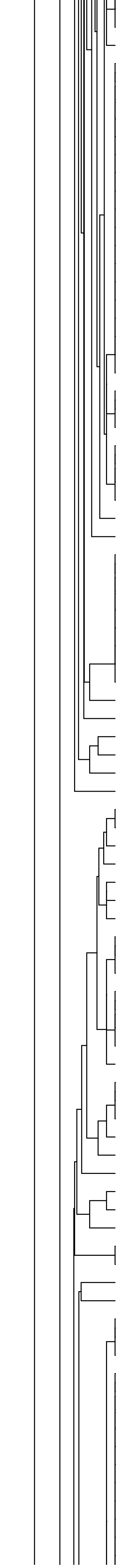

4	5	3	12	2	2	3	1	6	43	8	4	5	6	5	3	2013Garofolo_19.	B. abortus bv3	36	72		19434	Cattle	2011	Morano Calabro,Italy
4	5	3	12	2	2	3	1	6	43	8	4	5	6	5	3	2013Garofolo_21.	B. abortus bv3	36	72		21677	Cattle	2011	Vico Equense,Italy
4	5	3	12	2	2	3	1	6	43	8	4	5	0	5	3	2012Ferreira#160	B. abortus bv3	36	72		LNIV-P22Ba3/03	Cattle	2003	Beira Litoral, Portugal
4	5	3	12	2	2	3	1	6	43	8	4	5	7	5	3	2013Garofolo_12.	B. abortus bv3	36	72		12368	Cattle	2011	Campana,Italy
4	5	3	12	2	2	3	1	6	43	8	4	5	7	5	3	2013Garofolo_12.	B. abortus bv3	36	72		12837	Cattle	2011	Verzino,Italy
4	5	3	12	2	2	3	1	6	43	8	4	5	7	5	3	2013Garofolo_12.	B. abortus bv3	36	72		12847	Cattle	2011	Verzino,Italy
4	5	3	12	2	2	3	1	6	43	8	4	5	7	5	3	2013Garofolo_12.	B. abortus bv3	36	72		12848	Cattle	2011	Umbriatico,Italy
4	5	3	12	2	2	3	1	6	43	8	4	5	7	5	3	2013Garofolo_17.	B. abortus bv3	36	72		17473	Cattle	2011	Campana,Italy
4	5	3	12	2	2	3	1	6	43	8	4	5	7	5	3	2013Garofolo_18.	B. abortus bv3	36	72		18948	Cattle	2011	Verzino,Italy
4	5	3	12	2	2	3	1	6	43	8	4	5	7	5	3	2013Garofolo_2216	B. abortus bv3	36	72		2216	Cattle	2011	Verzino,Italy
4	5	3	12	2	2	3	1	6	43	8	4	5	7	5	3	2013Garofolo_2222	B. abortus bv3	36	72		2222	Cattle	2011	Verzino,Italy
4	5	3	12	2	2	3	1	6	43	8	4	5	7	5	3	2013Garofolo_3270	B. abortus bv3	36	72		3270	Cattle	2011	Verzino,Italy
4	5	3	12	2	2	3	1	6	43	8	4	5	7	5	3	2013Garofolo_3821	B. abortus bv3	36	72		3821	Cattle	2011	Verzino,Italy
4	5	3	12	2	2	3	1	6	43	8	4	5	7	5	3	2013Garofolo_5585	B. abortus bv3	36	72		5585	Cattle	2011	Caccuri,Italy
4	5	3	12	2	2	3	1	6	43	8	4	5	7	5	3	2013Garofolo_5586	B. abortus bv3	36	72		5586	Cattle	2011	Pallagorio,Italy
4	5	3	12	2	2	3	1	6	43	8	4	5	7	5	3	2013Garofolo_6053	B. abortus bv3	36	72		6053	Cattle	2011	Campana,Italy
4	5	3	12	2	2	3	1	6	43	8	4	5	7	5	3	2013Garofolo_6054	B. abortus bv3	36	72		6054	Cattle	2011	Cariati,Italy
4	5	3	12	2	2	3	1	6	43	8	4	5	7	5	3	2013Garofolo_6064	B. abortus bv3	36	72		6064	Cattle	2011	Verzino,Italy
4	5	3	12	2	2	3	1	6	43	8	4	5	7	5	3	2013Garofolo_6461	B. abortus bv3	36	72		6461	Cattle	2011	Verzino,Italy
4	5	3	12	2	2	3	1	6	43	8	4	5	7	5	3	2013Garofolo_7742	B. abortus bv3	36	72		7742	Cattle	2011	Pallagorio,Italy
4	5	3	12	2	2	3	1	6	43	8	4	5	7	5	3	2013Garofolo_9056	B. abortus bv3	36	72		9056	Cattle	2011	Verzino,Italy
4	5	3	12	2	2	3	1	6	43	8	4	5	7	6	3	2013Garofolo_1159	B. abortus bv3	36	72		1159	Cattle	2011	Pietrapaola,Italy
4	5	3	12	2	2	3	1	6	43	8	4	5	7	6	3	2013Garofolo_1166	B. abortus bv3	36	72		1166	Cattle	2011	San Sosti,Italy
4	5	3	12	2	2	3	1	6	43	8	4	5	7	6	3	2013Garofolo_20.	B. abortus bv3	36	72		20826	Cattle	2011	Longobucco,Italy
4	5	3	12	2	2	3	1	6	43	8	4	5	7	3	3	2006LeFleche#112	B. abortus bv3	36	72		BCCN#94-18	Cattle	1994	Limoges, France
4	5	3	12	2	2	3	1	6	43	8	4	5	7	3	3	2006LeFleche#113	B. abortus bv3	36	72		BCCN#94-19	Cattle	1994	Limoges, France
4	5	3	12	2	2	3	1	6	43	8	4	5	7	3	3	2013Jiang#089	B. abortus bv3	36	72			Cattle	2011	Hebei, China
4	5	3	12	2	2	3	1	6	43	8	4	5	7	3	3	2013Jiang#090	B. abortus bv3	36	72			Cattle	2011	Hebei, China
4	5	3	12	2	2	3	1	6	43	8	4	5	8	6	3	2013Garofolo_7747	B. abortus bv3	36	72		7747	Cattle	2011	Pietrapaola,Italy
4	5	3	12	2	2	3	1	6	43	8	4	3	3	4	3	2013Garofolo_1567	B. abortus bv3	36	72		1567	Cattle	2011	Agnone,Italy
4	5	3	12	2	2	3	1	6	43	8	6	6	5	4	3	2013Garofolo_12.	B. abortus bv3	36	72		12839	Cattle	2011	Umbriatico,Italy
4	5	3	12	2	2	3	1	6	43	8	6	6	5	4	3	2013Garofolo_17.	B. abortus bv3	36	72		17472	Cattle	2011	Scala Coeli,Italy
4	5	3	12	2	2	3	1	6	43	8	6	6	5	4	3	2013Garofolo_17.	B. abortus bv3	36	72		17476	Cattle	2011	Umbriatico,Italy
4	5	3	12	2	2	3	1	6	43	8	6	6	5	4	3	2013Garofolo_18.	B. abortus bv3	36	72		18936	Cattle	2011	Casabona,Italy
4	5	3	12	2	2	3	1	6	43	8	6	6	5	4	3	2013Garofolo_6047	B. abortus bv3	36	72		6047	Cattle	2011	Scala Coeli,Italy
4	5	3	12	2	2	3	1	6	43	8	6	6	5	4	3	2013Garofolo_6459	B. abortus bv3	36	72		6459	Cattle	2011	Umbriatico,Italy
4	5	3	12	2	2	3	1	6	43	8	6	6	5	4	3	2013Garofolo_9059	B. abortus bv3	36	72		9059	Cattle	2011	Umbriatico,Italy
4	5	3	12	2	2	3	1	6	43	8	6	6	5	4	3	2013Garofolo_9060	B. abortus bv3	36	72		9060	Cattle	2011	Scala Coeli,Italy
1	5	3	12	2	2	3	1	6	43	8	6	6	6	3	3	2011Kilic#093	B. abortus bv3	86	69		BRU-S093	Human	2006	Trabzon, Turkey
4	5	3	12	2	2	3	1	6	43	8	5	4	4	3	5	2013Jiang#119	B. abortus bv3	36	72		NM1132	Cattle	1988	Inner Mongolia, China
2	5	3	12	2	2	3	1	6	45	8	4	9	6	3	3	2006LeFleche#145	B. abortus bv6	38	68		BfR 124	Cattle	1993	Syria
2	5	3	12	2	2	3	1	7	43	8	4	9	6	3	3	2016Shevtsova#037	B. abortus	38	341		60AI	Cattle	1960	Almaty, Kazakhstan
2	5	3	12	2	2	3	1	6	43	8	4	8	5	3	3	2016Shevtsova#030	B. abortus	38	339		53AI	Cattle	1953	Eskeldi, Almaty, Kazakh.
4	5	4	12	2	2	3	2	6	43	8	6	6	7	3	3	2013Jiang#095	B. abortus bv3	30	78			Human	2012	Inner Mongolia, China
3	5	4	12	1	2	3	3	6	43	8	5	4	3	5	4	2012Ferreira#138	B. abortus bv1	98	183		LNIV-P2Ba1/03	Cattle	2003	Beira Litoral, Portugal
3	5	4	12	1	2	3	3	6	43	8	5	4	3	5	4	2012Ferreira#143	B. abortus bv1	98	183		LNIV-P9Ba1/03	Cattle	2003	Entre Douro e Minho, P.
3	5	4	12	1	2	3	3	6	43	8	5	4	3	5	5	2012Ferreira#139	B. abortus bv1	98	183		LNIV-P38Ba1/04	Cattle	2004	Beira Litoral, Portugal
3	5	4	12	1	2	3	3	6	43	8	5	4	3	6	4	2012Ferreira#141	B. abortus bv1	98	183		LNIV-P7Ba1/04	Cattle	2004	Entre Douro e Minho, P.
3	5	4	12	1	2	3	3	6	43	8	4	4	3	5	4	2012Ferreira#140	B. abortus bv1	98	183		LNIV-P6Ba1/04	Cattle	2004	Entre Douro e Minho, P.
3	5	4	12	1	2	3	3	6	43	8	4	4	3	8	4	2012Ferreira#144	B. abortus bv1	98	183		LNIV-4Ba1-01	Cattle	2001	Tras-os-Montes, Portugal
3	5	4	12	1	2	3	3	6	43	8	4	4	3	6	4	2012Ferreira#142	B. abortus bv1	98	183		LNIV-P8Ba1/04	Cattle	2004	Entre Douro e Minho, P.
4	5	4	12	1	2	3	3	6	43	8	5	4	3	5	4	2012Ferreira#181	B. abortus bv1	32	182		LNIV-P5Ba1/04	Cattle	2004	Entre Douro e Minho, P.
4	5	4	12	1	2	3	3	6	43	8	5	4	3	5	4	2012Ferreira#209	B. abortus bv1	32	182		LNIV-P12Ba1/04	Cattle	2004	Tras-os-Montes, Portugal
4	5	4	12	1	2	3	3	6	43	8	5	4	3	5	4	2012Ferreira#210	B. abortus bv1	32	182		LNIV-P13Ba1/04	Cattle	2004	Tras-os-Montes, Portugal
4	5	4	12	1	2	3	3	6	43	8	4	4	3	5	4	2012Ferreira#180	B. abortus bv1	32	182		LNIV-P4Ba1/04	Cattle	2004	Entre Douro e Minho, P.
4	5	4	12	1	2	3	3	6	43	8	4	4	3	5	4	2012Ferreira#182	B. abortus bv1	32	182		LNIV-P10Ba1/04	Cattle	2004	Entre Douro e Minho, P.
4	5	4	12	1	2	3	3	6	43	8	4	4	3	5	4	2012Ferreira#183	B. abortus bv1	32	182		LNIV-P11Ba1/03	Cattle	2003	Entre Douro e Minho, P.
4	5	4	12	1	2	3	3	6	43	8	4	4	3	5	4	2012Ferreira#211	B. abortus bv1	32	182		LNIV-P14Ba1/03	Cattle	2003	Tras-os-Montes, Portugal
4	5	4	12	1	2	3	3	6	43	8	1	4	3	5	4	2018Vergnaud#0290	B. abortus atypi.	32	182		BCCN#87-56	Cattle	1987	Canada
4	5	4	12	1	2	3	3	6	43	8	1	4	3	6	6	SRR1371371	B. abortus	32	182	1	B94-0500	Cattle		United States - FL
4	5	4	12	1	2	3	3	6	43	8	1	4	3	6	6	SRR1371380	B. abortus	32	182	1	B96-0242	Cattle		United States - GA
4	5	4	12	1	2	3	3	6	43	8	1	4	3	6	6	SRR1371384	B. abortus	32	182	60	B98-0822	Cattle		United States - TX
4	5	4	12	1	2	3	3	6	43	8	1	4	3	5	6	SRR1371369	B. abortus	32	182	60	B93-0824	Cattle		United States - FL
4	5	4	12	1	2	3	3	6	45	8	1	4	3	4	6	2013Minharro#088	B. abortus bv4	32	337		16_fev	Cattle	2002	Porto Alegre, 90570020.
4	5	4	12	1	2	3	3	6	43	6	5	4	3	4	5	2006LeFleche#140	B. abortus bv1	32	73		BfR 100	unknown	1972	Musterkappeln, Germany
3	5	4	12	2	2	3	3	6	43	8	5	6	3	4	4	2006LeFleche#101	B. abortus bv1	33	76		BCCN#92-73	Cattle	1992	Limoges, France
3	5	4	12	2	2	3	3	6	43	8	5	7	3	4	4	2006LeFleche#102	B. abortus bv1	33	76		BCCN#95-11	Cattle	1995	Clermont-Ferrand, Fra.
3	5	4	12	2	2	3	3	6	43	8	5	4	3	8	7	2013Minharro#048	B. abortus bv1	33	76		175	Cattle	2008	Redencao, 68550010, .
4	5	4	12	1	1	3	3	6	43	8	2	5	3	4	4	2018Vergnaud#0249	B. abortus atypi.	133	245		BCCN#82-98A	Cattle	1982	Le Bourget du Lac ,Sav.
4	5	4	12	1	1	3	3	6	43	8	2	5	3	4	4	2018Vergnaud#0250	B. abortus atypi.	133	245		BCCN#82-98B	Cattle	1982	Le Bourget du Lac ,Sav.
4	5	4	12	2	2	3	3	6	43	8	3	5	12	5	4	2013Minharro#082	B. abortus bv1	28	82		11_jun	Cattle	2006	Porto Alegre, 90570020.
4	5	4	12	2	2	3	3	6	43	8	3	1	6	3	6	2017Hanot-Mamb.	B. abortus bv1	28	82		L3/129	Human	2000	Belgium
4	5	4	12	2	3	3	3	6	43	8	4	4	3	3	7	2009Her#142	B. abortus bv1	27	79		KBa131	Cattle	2005	Gyeonggi, South Korea
4	5	4	12	2	3	3	3	6	43	8	4	4	3	3	7	2009Her#184	B. abortus bv1	27	79		KBa173	Cattle	2008	Jeonnam, South Korea
4	5	4	12	2	3	3	3	6	43	8	4	4	3	3	7	2010Her#18	B. abortus	27	79		#40	Human	2005	Gyeongbuk, South Korea
4	5	4	12	2	3	3	3	6	43	8	4	4	3	3	5	2009Her#063	B. abortus bv1	27	79		KBa052	Cattle	2007	Gyeonggi, South Korea
4	5	4	12	2	3	3	3	6	43	8	4	4	3	3	5	2009Her#064	B. abortus bv1	27	79		KBa053	Cattle	2007	Gyeonggi, South Korea
4	5	4	12	2	3	3	3	6	43	8	4	4	3	3	5	2009Her#065	B. abortus bv1	27	79		KBa054	Cattle	2007	Gyeonggi, South Korea
4	5	4	12	2	3	3	3	6	43	8	4	4	3	3	5	2009Her#066	B. abortus bv1	27	79		KBa055	Cattle	2007	Gyeonggi, South Korea
4	5	4	12	2	3	3	3	6	43	8	4	4	3	3	5	2009Her#067	B. abortus bv1	27	79		KBa056	Cattle	2007	Gyeonggi, South Korea
4	5	4	12	2	3	3	3	6	43	8	4	4	3	3	5	2009Her#068	B. abortus bv1	27	79		KBa057	Cattle	2007	Gyeonggi, South Korea
4	5	4	12	2	3	3	3	6	43	8	4	4	3	3	5	2009Her#073	B. abortus bv1	27	79		KBa062	Cattle	2007	Chungbuk, South Korea
4	5	4	12	2	3	3	3	6	43	8	4	4	3	3	5	2009Her#074	B. abortus bv1	27	79		KBa063	Cattle	2007	Chungbuk, South Korea
4	5	4	12	2	3	3	3	6	43	8	4	4	3	3	5	2009Her#075	B. abortus bv1	27	79		KBa064	Cattle	2007	Chungbuk, South Korea
4	5	4	12	2	3	3	3	6	43	8	4	4	3	3	5	2009Her#105	B. abortus bv1	27	79		KBa094	Cattle	2008	Gyeonggi, South Korea
4	5	4	12	2	3	3	3	6	43	8	4	4	3	3	5	2009Her#110	B. abortus bv1	27	79		KBa099	Cattle	1999	Gyeonggi, South Korea

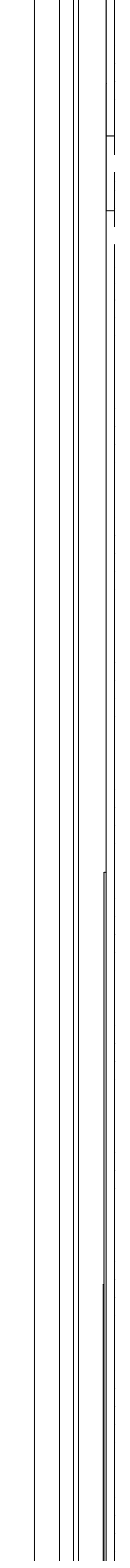

4	5	4	12	2	3	3	3	6	43	8	4	4	3	3	5	2009Her#115	B. abortus bv1	27	79	KBa104	Cattle	2002	Gyeongbuk, South Korea
4	5	4	12	2	3	3	3	6	43	8	4	4	3	3	5	2009Her#116	B. abortus bv1	27	79	KBa105	Cattle	2003	Gyeongbuk, South Korea
4	5	4	12	2	3	3	3	6	43	8	4	4	3	3	5	2009Her#125	B. abortus bv1	27	79	KBa114	Cattle	2004	Gyeonggi, South Korea
4	5	4	12	2	3	3	3	6	43	8	4	4	3	3	5	2009Her#130	B. abortus bv1	27	79	KBa119	Cattle	2004	Jeonbuk, South Korea
4	5	4	12	2	3	3	3	6	43	8	4	4	3	3	5	2009Her#134	B. abortus bv1	27	79	KBa123	Cattle	2005	Kangwon, South Korea
4	5	4	12	2	3	3	3	6	43	8	4	4	3	3	5	2009Her#135	B. abortus bv1	27	79	KBa124	Cattle	2005	Kangwon, South Korea
4	5	4	12	2	3	3	3	6	43	8	4	4	3	3	5	2009Her#139	B. abortus bv1	27	79	KBa128	Cattle	2005	Kangwon, South Korea
4	5	4	12	2	3	3	3	6	43	8	4	4	3	3	5	2009Her#141	B. abortus bv1	27	79	KBa130	Cattle	2005	Kangwon, South Korea
4	5	4	12	2	3	3	3	6	43	8	4	4	3	3	5	2010Her#38	B. abortus	27	79	#91	Human	2007	Jeonbuk, South Korea
4	5	4	12	2	3	3	3	6	43	8	4	4	3	3	4	2009Her#028	B. abortus bv1	27	79	KBa017	Cattle	1996	Jeju, South Korea
4	5	4	12	2	3	3	3	6	43	8	4	4	3	3	4	2009Her#121	B. abortus bv1	27	79	KBa110	Cattle	2004	Gyeonggi, South Korea
4	5	4	12	2	3	3	3	6	43	8	4	4	3	3	4	2009Her#137	B. abortus bv1	27	79	KBa126	Cattle	2005	Kangwon, South Korea
4	5	4	12	2	3	3	3	6	43	8	4	4	3	3	4	2009Her#138	B. abortus bv1	27	79	KBa127	Cattle	2005	Kangwon, South Korea
4	5	4	12	2	3	3	3	6	43	8	4	4	3	3	6	2009Her#019	B. abortus bv1	27	79	KBa008	Cattle	2004	Jeju, South Korea
4	5	4	12	2	3	3	3	6	43	8	4	4	3	3	6	2009Her#020	B. abortus bv1	27	79	KBa009	Cattle	2004	Jeju, South Korea
4	5	4	12	2	3	3	3	6	43	8	4	4	3	3	6	2009Her#021	B. abortus bv1	27	79	KBa010	Cattle	2004	Jeju, South Korea
4	5	4	12	2	3	3	3	6	43	8	4	4	3	3	6	2009Her#022	B. abortus bv1	27	79	KBa011	Cattle	1996	Chungnam, South Korea
4	5	4	12	2	3	3	3	6	43	8	4	4	3	3	6	2009Her#023	B. abortus bv1	27	79	KBa012	Cattle	1996	Chungnam, South Korea
4	5	4	12	2	3	3	3	6	43	8	4	4	3	3	6	2009Her#024	B. abortus bv1	27	79	KBa013	Cattle	1996	Chungnam, South Korea
4	5	4	12	2	3	3	3	6	43	8	4	4	3	3	6	2009Her#025	B. abortus bv1	27	79	KBa014	Cattle	1996	Chungnam, South Korea
4	5	4	12	2	3	3	3	6	43	8	4	4	3	3	6	2009Her#026	B. abortus bv1	27	79	KBa015	Cattle	1996	Chungnam, South Korea
4	5	4	12	2	3	3	3	6	43	8	4	4	3	3	6	2009Her#027	B. abortus bv1	27	79	KBa016	Cattle	1996	Chungnam, South Korea
4	5	4	12	2	3	3	3	6	43	8	4	4	3	3	6	2009Her#029	B. abortus bv1	27	79	KBa018	Cattle	2002	Jeonbuk, South Korea
4	5	4	12	2	3	3	3	6	43	8	4	4	3	3	6	2009Her#031	B. abortus bv1	27	79	KBa020	Cattle	2000	Gyeonggi, South Korea
4	5	4	12	2	3	3	3	6	43	8	4	4	3	3	6	2009Her#045	B. abortus bv1	27	79	KBa034	Cattle	1998	Gyeongbuk, South Korea
4	5	4	12	2	3	3	3	6	43	8	4	4	3	3	6	2009Her#046	B. abortus bv1	27	79	KBa035	Cattle	2006	Gyeongbuk, South Korea
4	5	4	12	2	3	3	3	6	43	8	4	4	3	3	6	2009Her#070	B. abortus bv1	27	79	KBa059	Cattle	2007	Kangwon, South Korea
4	5	4	12	2	3	3	3	6	43	8	4	4	3	3	6	2009Her#071	B. abortus bv1	27	79	KBa060	Cattle	2007	Kangwon, South Korea
4	5	4	12	2	3	3	3	6	43	8	4	4	3	3	6	2009Her#072	B. abortus bv1	27	79	KBa061	Cattle	2007	Kangwon, South Korea
4	5	4	12	2	3	3	3	6	43	8	4	4	3	3	6	2009Her#076	B. abortus bv1	27	79	KBa065	Cattle	2007	Kangwon, South Korea
4	5	4	12	2	3	3	3	6	43	8	4	4	3	3	6	2009Her#081	B. abortus bv1	27	79	KBa070	Cattle	2007	Kangwon, South Korea
4	5	4	12	2	3	3	3	6	43	8	4	4	3	3	6	2009Her#082	B. abortus bv1	27	79	KBa071	Cattle	2007	Kangwon, South Korea
4	5	4	12	2	3	3	3	6	43	8	4	4	3	3	6	2009Her#083	B. abortus bv1	27	79	KBa072	Cattle	2007	Gyeonggi, South Korea
4	5	4	12	2	3	3	3	6	43	8	4	4	3	3	6	2009Her#084	B. abortus bv1	27	79	KBa073	Cattle	2007	Gyeonggi, South Korea
4	5	4	12	2	3	3	3	6	43	8	4	4	3	3	6	2009Her#085	B. abortus bv1	27	79	KBa074	Cattle	2007	Kangwon, South Korea
4	5	4	12	2	3	3	3	6	43	8	4	4	3	3	6	2009Her#086	B. abortus bv1	27	79	KBa075	Cattle	2007	Kangwon, South Korea
4	5	4	12	2	3	3	3	6	43	8	4	4	3	3	6	2009Her#087	B. abortus bv1	27	79	KBa076	Cattle	2007	Kangwon, South Korea
4	5	4	12	2	3	3	3	6	43	8	4	4	3	3	6	2009Her#088	B. abortus bv1	27	79	KBa077	Cattle	2007	Kangwon, South Korea
4	5	4	12	2	3	3	3	6	43	8	4	4	3	3	6	2009Her#089	B. abortus bv1	27	79	KBa078	Cattle	2007	Chungbuk, South Korea
4	5	4	12	2	3	3	3	6	43	8	4	4	3	3	6	2009Her#106	B. abortus bv1	27	79	KBa095	Deer/El.	2008	Jeonnam, South Korea
4	5	4	12	2	3	3	3	6	43	8	4	4	3	3	6	2009Her#107	B. abortus bv1	27	79	KBa096	Deer/El.	2008	Jeonnam, South Korea
4	5	4	12	2	3	3	3	6	43	8	4	4	3	3	6	2009Her#108	B. abortus bv1	27	79	KBa097	Deer/El.	2008	Jeonnam, South Korea
4	5	4	12	2	3	3	3	6	43	8	4	4	3	3	6	2009Her#109	B. abortus bv1	27	79	KBa098	Cattle	1996	Chungnam, South Korea
4	5	4	12	2	3	3	3	6	43	8	4	4	3	3	6	2009Her#111	B. abortus bv1	27	79	KBa100	Cattle	1999	Chungnam, South Korea
4	5	4	12	2	3	3	3	6	43	8	4	4	3	3	6	2009Her#113	B. abortus bv1	27	79	KBa102	Cattle	2000	Chungbuk, South Korea
4	5	4	12	2	3	3	3	6	43	8	4	4	3	3	6	2009Her#114	B. abortus bv1	27	79	KBa103	Cattle	2001	Gyeonggi, South Korea
4	5	4	12	2	3	3	3	6	43	8	4	4	3	3	6	2009Her#117	B. abortus bv1	27	79	KBa106	Cattle	2003	Chungnam, South Korea
4	5	4	12	2	3	3	3	6	43	8	4	4	3	3	6	2009Her#119	B. abortus bv1	27	79	KBa108	Cattle	2004	Gyeonggi, South Korea
4	5	4	12	2	3	3	3	6	43	8	4	4	3	3	6	2009Her#123	B. abortus bv1	27	79	KBa112	Cattle	2004	Gyeonggi, South Korea
4	5	4	12	2	3	3	3	6	43	8	4	4	3	3	6	2009Her#127	B. abortus bv1	27	79	KBa116	Cattle	2004	Gyeonggi, South Korea
4	5	4	12	2	3	3	3	6	43	8	4	4	3	3	6	2009Her#128	B. abortus bv1	27	79	KBa117	Cattle	2004	Jeonbuk, South Korea
4	5	4	12	2	3	3	3	6	43	8	4	4	3	3	6	2009Her#129	B. abortus bv1	27	79	KBa118	Cattle	2004	Jeonbuk, South Korea
4	5	4	12	2	3	3	3	6	43	8	4	4	3	3	6	2009Her#131	B. abortus bv1	27	79	KBa120	Cattle	2004	Jeonbuk, South Korea
4	5	4	12	2	3	3	3	6	43	8	4	4	3	3	6	2009Her#132	B. abortus bv1	27	79	KBa121	Cattle	2005	Kangwon, South Korea
4	5	4	12	2	3	3	3	6	43	8	4	4	3	3	6	2009Her#136	B. abortus bv1	27	79	KBa125	Cattle	2005	Kangwon, South Korea
4	5	4	12	2	3	3	3	6	43	8	4	4	3	3	6	2009Her#140	B. abortus bv1	27	79	KBa129	Cattle	2005	Kangwon, South Korea
4	5	4	12	2	3	3	3	6	43	8	4	4	3	3	6	2009Her#143	B. abortus bv1	27	79	KBa132	Cattle	2005	Gyeonggi, South Korea
4	5	4	12	2	3	3	3	6	43	8	4	4	3	3	6	2009Her#147	B. abortus bv1	27	79	KBa136	Cattle	2005	Gyeongnam, South Ko.
4	5	4	12	2	3	3	3	6	43	8	4	4	3	3	6	2009Her#151	B. abortus bv1	27	79	KBa140	Cattle	2005	Gyeongbuk, South Korea
4	5	4	12	2	3	3	3	6	43	8	4	4	3	3	6	2009Her#153	B. abortus bv1	27	79	KBa142	Cattle	2005	Jeonbuk, South Korea
4	5	4	12	2	3	3	3	6	43	8	4	4	3	3	6	2009Her#154	B. abortus bv1	27	79	KBa143	Cattle	2005	Jeonbuk, South Korea
4	5	4	12	2	3	3	3	6	43	8	4	4	3	3	6	2009Her#155	B. abortus bv1	27	79	KBa144	Cattle	2005	Jeonbuk, South Korea
4	5	4	12	2	3	3	3	6	43	8	4	4	3	3	6	2009Her#159	B. abortus bv1	27	79	KBa148	Cattle	2005	Chungnam, South Korea
4	5	4	12	2	3	3	3	6	43	8	4	4	3	3	6	2009Her#163	B. abortus bv1	27	79	KBa152	Cattle	2005	Chungnam, South Korea
4	5	4	12	2	3	3	3	6	43	8	4	4	3	3	6	2009Her#164	B. abortus bv1	27	79	KBa153	Cattle	2005	Chungbuk, South Korea
4	5	4	12	2	3	3	3	6	43	8	4	4	3	3	6	2009Her#166	B. abortus bv1	27	79	KBa155	Cattle	2006	Kangwon, South Korea
4	5	4	12	2	3	3	3	6	43	8	4	4	3	3	6	2009Her#170	B. abortus bv1	27	79	KBa159	Cattle	2002	Gyeongbuk, South Korea
4	5	4	12	2	3	3	3	6	43	8	4	4	3	3	6	2009Her#172	B. abortus bv1	27	79	KBa161	Cattle	2003	Gyeongbuk, South Korea
4	5	4	12	2	3	3	3	6	43	8	4	4	3	3	6	2009Her#173	B. abortus bv1	27	79	KBa162	Cattle	2003	Gyeongbuk, South Korea
4	5	4	12	2	3	3	3	6	43	8	4	4	3	3	6	2009Her#178	B. abortus bv1	27	79	KBa167	Cattle	2008	Gyeonggi, South Korea
4	5	4	12	2	3	3	3	6	43	8	4	4	3	3	6	2009Her#179	B. abortus bv1	27	79	KBa168	Cattle	2008	Gyeonggi, South Korea
4	5	4	12	2	3	3	3	6	43	8	4	4	3	3	6	2009Her#180	B. abortus bv1	27	79	KBa169	Cattle	2008	Gyeonggi, South Korea
4	5	4	12	2	3	3	3	6	43	8	4	4	3	3	6	2009Her#181	B. abortus bv2	27	79	KBa170	Cattle	2008	Jeonnam, South Korea
4	5	4	12	2	3	3	3	6	43	8	4	4	3	3	6	2009Her#185	B. abortus bv2	27	79	KBa174	Cattle	2008	Jeonnam, South Korea
4	5	4	12	2	3	3	3	6	43	8	4	4	3	3	6	2009Her#186	B. abortus bv2	27	79	KBa175	Cattle	2008	Jeonnam, South Korea
4	5	4	12	2	3	3	3	6	43	8	4	4	3	3	6	2009Her#187	B. abortus bv2	27	79	KBa176	Cattle	2008	Jeonnam, South Korea
4	5	4	12	2	3	3	3	6	43	8	4	4	3	3	6	2009Her#188	B. abortus bv2	27	79	KBa177	Cattle	2008	Jeonnam, South Korea
4	5	4	12	2	3	3	3	6	43	8	4	4	3	3	6	2010Her#01	B. abortus	27	79	#2	Human	2003	Gangwon, South Korea
4	5	4	12	2	3	3	3	6	43	8	4	4	3	3	6	2010Her#02	B. abortus	27	79	#3	Human	2003	Gangwon, South Korea
4	5	4	12	2	3	3	3	6	43	8	4	4	3	3	6	2010Her#04	B. abortus	27	79	#6	Human	2004	Chungbuk, South Korea
4	5	4	12	2	3	3	3	6	43	8	4	4	3	3	6	2010Her#05	B. abortus	27	79	#8	Human	2004	Gyeongnam, South Ko.
4	5	4	12	2	3	3	3	6	43	8	4	4	3	3	6	2010Her#09	B. abortus	27	79	#17	Human	2004	Jeonbuk, South Korea
4	5	4	12	2	3	3	3	6	43	8	4	4	3	3	6	2010Her#10	B. abortus	27	79	#20	Human	2005	Jeonbuk, South Korea
4	5	4	12	2	3	3	3	6	43	8	4	4	3	3	6	2010Her#11	B. abortus	27	79	#21	Human	2005	Ulsan, South Korea
4	5	4	12	2	3	3	3	6	43	8	4	4	3	3	6	2010Her#12	B. abortus	27	79	#22	Human	2005	Gyeongnam, South Ko.
4	5	4	12	2	3	3	3	6	43	8	4	4	3	3	6	2010Her#16	B. abortus	27	79	#36	Human	2005	Chungnam, South Korea

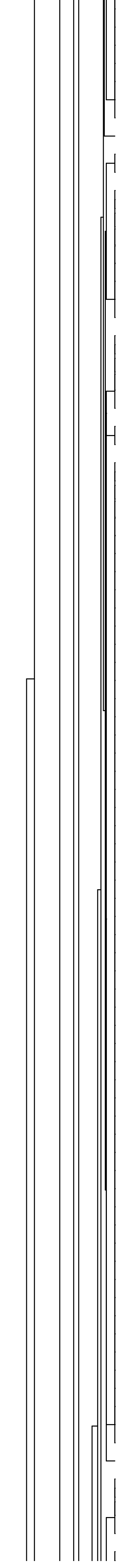

4	5	4	12	2	3	3	3	6	43	8	4	4	3	3	6	2010Her#21	B. abortus	27	79		#49	Human	2006	Gyeongnam, South Ko.
4	5	4	12	2	3	3	3	6	43	8	4	4	3	3	6	2010Her#22	B. abortus	27	79		#50	Human	2006	Chungnam, South Korea
4	5	4	12	2	3	3	3	6	43	8	4	4	3	3	6	2010Her#23	B. abortus	27	79		#53	Human	2006	Gyeonggi, South Korea
4	5	4	12	2	3	3	3	6	43	8	4	4	3	3	6	2010Her#26	B. abortus	27	79		#61	Human	2006	Gyeongbuk, South Korea
4	5	4	12	2	3	3	3	6	43	8	4	4	3	3	6	2010Her#27	B. abortus	27	79		#63	Human	2006	Gwangju, South Korea
4	5	4	12	2	3	3	3	6	43	8	4	4	3	3	6	2010Her#34	B. abortus	27	79		#87	Human	2007	Gwangju, South Korea
4	5	4	12	2	3	3	3	6	43	8	4	4	3	3	6	GCA_000238175	B. abortus	27	79	5	A13334	Cattle	2011	Gyeonggi, Yangpyeong.
4	5	4	12	2	3	3	3	6	43	8	4	4	3	6	6	SRR1371383	B. abortus	27	79	1	B98-0688	Cattle		United States - TX
4	5	4	12	2	4	3	3	6	43	8	4	4	3	3	8	2009Her#042	B. abortus bv1	91	176		KBa031	Cattle	2006	Gyeonggi, South Korea
4	5	4	12	2	4	3	3	6	43	8	4	4	3	3	8	2009Her#044	B. abortus bv1	91	176		KBa033	Cattle	2006	Gyeonggi, South Korea
4	5	4	12	2	4	3	3	6	43	8	4	4	3	3	7	2009Her#040	B. abortus bv1	91	176		KBa029	Cattle	2006	Gyeonggi, South Korea
4	5	4	12	2	4	3	3	6	43	8	4	4	3	3	7	2009Her#041	B. abortus bv1	91	176		KBa030	Cattle	2006	Gyeonggi, South Korea
4	5	4	12	2	4	3	3	6	43	8	4	4	3	3	7	2009Her#043	B. abortus bv1	91	176		KBa032	Cattle	2006	Gyeonggi, South Korea
4	5	4	12	2	4	3	3	6	43	8	4	4	3	3	7	2009Her#102	B. abortus bv1	91	176		KBa091	Cattle	2008	Gyeonggi, South Korea
4	5	4	12	2	4	3	3	6	43	8	4	4	3	3	7	2009Her#103	B. abortus bv1	91	176		KBa092	Cattle	2008	Gyeonggi, South Korea
4	5	4	12	2	4	3	3	6	43	8	4	4	3	3	7	2009Her#104	B. abortus bv1	91	176		KBa093	Cattle	2008	Gyeonggi, South Korea
4	5	4	12	2	4	3	3	6	43	8	4	4	3	3	7	2009Her#167	B. abortus bv1	91	176		KBa156	Cattle	2006	Gyeonggi, South Korea
4	5	4	12	2	4	3	3	6	43	8	4	4	3	3	7	2010Her#28	B. abortus	91	176		#68	Human	2006	Gyeongbuk, South Korea
4	5	4	12	2	5	3	3	6	43	8	4	4	3	3	6	2009Her#094	B. abortus bv1	89	174		KBa083	Cattle	2008	Gyeongnam, South Ko.
4	5	4	12	2	5	3	3	6	43	8	4	4	3	3	6	2009Her#095	B. abortus bv1	89	174		KBa084	Cattle	2008	Gyeongnam, South Ko.
4	5	4	12	2	5	3	3	6	43	8	4	4	3	3	6	2009Her#096	B. abortus bv1	89	174		KBa085	Cattle	2008	Gyeongnam, South Ko.
4	5	4	12	2	5	3	3	6	43	8	4	4	3	3	6	2009Her#097	B. abortus bv1	89	174		KBa086	Cattle	2008	Gyeongnam, South Ko.
4	5	4	12	2	5	3	3	6	43	8	4	4	3	3	6	2010Her#37	B. abortus	89	174		#90	Human	2007	Daejeon, South Korea
4	5	4	12	2	6	3	3	6	43	8	4	4	3	3	6	2009Her#033	B. abortus bv1	90	175		KBa022	Cattle	2005	Gyeongnam, South Ko.
4	5	4	12	2	6	3	3	6	43	8	4	4	3	3	6	2009Her#036	B. abortus bv1	90	175		KBa025	Cattle	2005	Gyeongnam, South Ko.
4	5	4	12	2	4	3	3	6	43	8	4	4	3	3	6	2009Her#012	B. abortus bv1	91	176		KBa001	Cattle	2004	Jeonbuk, South Korea
4	5	4	12	2	4	3	3	6	43	8	4	4	3	3	6	2009Her#013	B. abortus bv1	91	176		KBa002	Cattle	2004	Jeonbuk, South Korea
4	5	4	12	2	4	3	3	6	43	8	4	4	3	3	6	2009Her#014	B. abortus bv1	91	176		KBa003	Cattle	2004	Jeonbuk, South Korea
4	5	4	12	2	4	3	3	6	43	8	4	4	3	3	6	2009Her#015	B. abortus bv1	91	176		KBa004	Cattle	2004	Jeonbuk, South Korea
4	5	4	12	2	4	3	3	6	43	8	4	4	3	3	6	2009Her#016	B. abortus bv1	91	176		KBa005	Cattle	2004	Jeonbuk, South Korea
4	5	4	12	2	4	3	3	6	43	8	4	4	3	3	6	2009Her#030	B. abortus bv1	91	176		KBa019	Cattle	2003	Jeonnam, South Korea
4	5	4	12	2	4	3	3	6	43	8	4	4	3	3	6	2009Her#032	B. abortus bv1	91	176		KBa021	Cattle	2005	Kangwon, South Korea
4	5	4	12	2	4	3	3	6	43	8	4	4	3	3	6	2009Her#037	B. abortus bv1	91	176		KBa026	Cattle	2005	Jeonnam, South Korea
4	5	4	12	2	4	3	3	6	43	8	4	4	3	3	6	2009Her#038	B. abortus bv1	91	176		KBa027	Cattle	2005	Jeonnam, South Korea
4	5	4	12	2	4	3	3	6	43	8	4	4	3	3	6	2009Her#039	B. abortus bv1	91	176		KBa028	Cattle	2005	Jeonnam, South Korea
4	5	4	12	2	4	3	3	6	43	8	4	4	3	3	6	2009Her#047	B. abortus bv1	91	176		KBa036	Cattle	2006	Gyeongbuk, South Korea
4	5	4	12	2	4	3	3	6	43	8	4	4	3	3	6	2009Her#048	B. abortus bv1	91	176		KBa037	Cattle	2006	Gyeongbuk, South Korea
4	5	4	12	2	4	3	3	6	43	8	4	4	3	3	6	2009Her#049	B. abortus bv1	91	176		KBa038	Cattle	2006	Gyeongbuk, South Korea
4	5	4	12	2	4	3	3	6	43	8	4	4	3	3	6	2009Her#050	B. abortus bv1	91	176		KBa039	Cattle	2006	Gyeongbuk, South Korea
4	5	4	12	2	4	3	3	6	43	8	4	4	3	3	6	2009Her#051	B. abortus bv1	91	176		KBa040	Cattle	2006	Gyeongbuk, South Korea
4	5	4	12	2	4	3	3	6	43	8	4	4	3	3	6	2009Her#052	B. abortus bv1	91	176		KBa041	Cattle	2006	Gyeongbuk, South Korea
4	5	4	12	2	4	3	3	6	43	8	4	4	3	3	6	2009Her#053	B. abortus bv1	91	176		KBa042	Cattle	2006	Gyeongbuk, South Korea
4	5	4	12	2	4	3	3	6	43	8	4	4	3	3	6	2009Her#054	B. abortus bv1	91	176		KBa043	Cattle	2006	Gyeongbuk, South Korea
4	5	4	12	2	4	3	3	6	43	8	4	4	3	3	6	2009Her#055	B. abortus bv1	91	176		KBa044	Cattle	2006	Gyeongbuk, South Korea
4	5	4	12	2	4	3	3	6	43	8	4	4	3	3	6	2009Her#056	B. abortus bv1	91	176		KBa045	Cattle	2006	Gyeongbuk, South Korea
4	5	4	12	2	4	3	3	6	43	8	4	4	3	3	6	2009Her#057	B. abortus bv1	91	176		KBa046	Cattle	2006	Gyeongbuk, South Korea
4	5	4	12	2	4	3	3	6	43	8	4	4	3	3	6	2009Her#060	B. abortus bv1	91	176		KBa049	Cattle	2006	Gyeongbuk, South Korea
4	5	4	12	2	4	3	3	6	43	8	4	4	3	3	6	2009Her#061	B. abortus bv1	91	176		KBa050	Cattle	2006	Gyeongbuk, South Korea
4	5	4	12	2	4	3	3	6	43	8	4	4	3	3	6	2009Her#077	B. abortus bv1	91	176		KBa066	Cattle	2007	Chungbuk, South Korea
4	5	4	12	2	4	3	3	6	43	8	4	4	3	3	6	2009Her#078	B. abortus bv1	91	176		KBa067	Cattle	2007	Chungbuk, South Korea
4	5	4	12	2	4	3	3	6	43	8	4	4	3	3	6	2009Her#079	B. abortus bv1	91	176		KBa068	Cattle	2007	Chungbuk, South Korea
4	5	4	12	2	4	3	3	6	43	8	4	4	3	3	6	2009Her#118	B. abortus bv1	91	176		KBa107	Cattle	2004	Kangwon, South Korea
4	5	4	12	2	4	3	3	6	43	8	4	4	3	3	6	2009Her#126	B. abortus bv1	91	176		KBa115	Cattle	2004	Gyeonggi, South Korea
4	5	4	12	2	4	3	3	6	43	8	4	4	3	3	6	2009Her#145	B. abortus bv1	91	176		KBa134	Cattle	2005	Gyeongnam, South Ko.
4	5	4	12	2	4	3	3	6	43	8	4	4	3	3	6	2009Her#146	B. abortus bv1	91	176		KBa135	Cattle	2005	Gyeongnam, South Ko.
4	5	4	12	2	4	3	3	6	43	8	4	4	3	3	6	2009Her#148	B. abortus bv1	91	176		KBa137	Cattle	2005	Gyeongnam, South Ko.
4	5	4	12	2	4	3	3	6	43	8	4	4	3	3	6	2009Her#149	B. abortus bv1	91	176		KBa138	Cattle	2005	Gyeongnam, South Ko.
4	5	4	12	2	4	3	3	6	43	8	4	4	3	3	6	2009Her#150	B. abortus bv1	91	176		KBa139	Cattle	2005	Gyeongnam, South Ko.
4	5	4	12	2	4	3	3	6	43	8	4	4	3	3	6	2009Her#152	B. abortus bv1	91	176		KBa141	Cattle	2005	Jeonnam, South Korea
4	5	4	12	2	4	3	3	6	43	8	4	4	3	3	6	2009Her#156	B. abortus bv1	91	176		KBa145	Cattle	2005	Jeonbuk, South Korea
4	5	4	12	2	4	3	3	6	43	8	4	4	3	3	6	2009Her#157	B. abortus bv1	91	176		KBa146	Cattle	2005	Jeonbuk, South Korea
4	5	4	12	2	4	3	3	6	43	8	4	4	3	3	6	2009Her#158	B. abortus bv1	91	176		KBa147	Cattle	2005	Jeonbuk, South Korea
4	5	4	12	2	4	3	3	6	43	8	4	4	3	3	6	2009Her#165	B. abortus bv1	91	176		KBa154	Cattle	2005	Chungbuk, South Korea
4	5	4	12	2	4	3	3	6	43	8	4	4	3	3	6	2009Her#174	B. abortus bv1	91	176		KBa163	Cattle	2006	Gyeongbuk, South Korea
4	5	4	12	2	4	3	3	6	43	8	4	4	3	3	6	2009Her#175	B. abortus bv1	91	176		KBa164	Cattle	2006	Gyeongbuk, South Korea
4	5	4	12	2	4	3	3	6	43	8	4	4	3	3	6	2009Her#176	B. abortus bv1	91	176		KBa165	Cattle	2006	Gyeongbuk, South Korea
4	5	4	12	2	4	3	3	6	43	8	4	4	3	3	6	2009Her#177	B. abortus bv1	91	176		KBa166	Cattle	2006	Gyeongbuk, South Korea
4	5	4	12	2	4	3	3	6	43	8	4	4	3	3	6	2010Her#06	B. abortus	91	176		#11	Human	2004	Gyeongbuk, South Korea
4	5	4	12	2	4	3	3	6	43	8	4	4	3	3	6	2010Her#07	B. abortus	91	176		#12	Human	2004	Gyeongbuk, South Korea
4	5	4	12	2	4	3	3	6	43	8	4	4	3	3	6	2010Her#08	B. abortus	91	176		#13	Human	2004	Gyeongbuk, South Korea
4	5	4	12	2	4	3	3	6	43	8	4	4	3	3	6	2010Her#13	B. abortus	91	176		#23	Human	2005	Chungnam, South Korea
4	5	4	12	2	4	3	3	6	43	8	4	4	3	3	6	2010Her#15	B. abortus	91	176		#31	Human	2005	Daegu, South Korea
4	5	4	12	2	4	3	3	6	43	8	4	4	3	3	6	2010Her#17	B. abortus	91	176		#37	Human	2005	Daegu, South Korea
4	5	4	12	2	4	3	3	6	43	8	4	4	3	3	6	2010Her#19	B. abortus	91	176		#42	Human	2005	Gyeongbuk, South Korea
4	5	4	12	2	4	3	3	6	43	8	4	4	3	3	6	2010Her#20	B. abortus	91	176		#48	Human	2006	Gyeongbuk, South Korea
4	5	4	12	2	4	3	3	6	43	8	4	4	3	3	6	2010Her#25	B. abortus	91	176		#60	Human	2006	Gyeongbuk, South Korea
4	5	4	12	2	4	3	3	6	43	8	4	4	3	3	6	2010Her#30	B. abortus	91	176		#74	Human	2006	Gyeonggi, South Korea
4	5	4	12	2	4	3	3	6	43	8	4	4	3	3	6	2010Her#31	B. abortus	91	176		#79	Human	2006	Gyeongbuk, South Korea
4	5	4	12	2	4	3	3	6	43	8	4	4	3	3	6	2010Her#35	B. abortus	91	176		#88	Human	2007	Gyeongbuk, South Korea
4	5	4	12	2	4	3	3	6	43	8	4	4	3	3	6	2010Her#36	B. abortus	91	176		#89	Human	2007	Gyeongbuk, South Korea
4	5	4	12	2	7	3	3	6	43	8	4	4	3	3	6	2009Her#034	B. abortus bv1	92	177		KBa023	Cattle	2005	Gyeongnam, South Ko.
4	5	4	12	1	2	3	3	6	43	8	4	4	3	3	6	2012Ferreira#174	B. abortus bv1	32	182		LNIV-15Ba1-01	Cattle	2001	Azores, Portugal
4	5	4	12	1	2	3	3	6	43	8	4	4	3	3	6	2012Ferreira#175	B. abortus bv1	32	182		LNIV-16Ba1-01	Cattle	2001	Azores, Portugal
4	5	4	12	1	2	3	3	6	43	8	4	4	3	3	6	2012Ferreira#176	B. abortus bv1	32	182		LNIV-17Ba1-01	Cattle	2001	Azores, Portugal
4	5	4	12	1	2	3	3	6	43	8	4	4	3	3	6	2012Ferreira#177	B. abortus bv1	32	182		LNIV-19Ba1-01	Cattle	2001	Azores, Portugal
4	5	4	12	2	2	3	3	6	43	8	4	4	3	3	6	2009Her#017	B. abortus bv1	28	82		KBa006	Cattle	2004	Jeju, South Korea

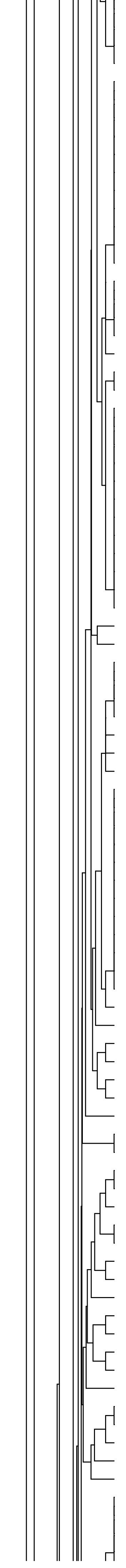

4	5	4	12	2	2	3	3	6	43	8	4	4	3	3	6	2009Her#018	B. abortus bv1	28	82		KBa007	Cattle	2004	Jeju, South Korea
4	5	4	12	2	2	3	3	6	43	8	4	4	3	3	6	2009Her#144	B. abortus bv1	28	82		KBa133	Cattle	2005	Gyeonggi, South Korea
4	5	4	12	2	2	3	3	6	43	8	4	4	3	3	6	2010Her#03	B. abortus	28	82		#4	Human	2003	Jeonbuk, South Korea
4	5	4	12	2	2	3	3	6	43	8	4	4	3	3	6	2017Hernández-M.	B. abortus	28	82		baboCR143	Cattle	2016	Costa Rica
4	5	4	12	2	3	3	3	6	43	8	5	4	3	3	5	2009Her#058	B. abortus bv1	27	79		KBa047	Cattle	2006	Gyeongbuk, South Korea
4	5	4	12	2	3	3	3	6	43	8	5	4	3	3	5	2009Her#098	B. abortus bv1	27	79		KBa087	Cattle	2008	Gyeonggi, South Korea
4	5	4	12	2	3	3	3	6	43	8	5	4	3	3	5	2009Her#099	B. abortus bv1	27	79		KBa088	Cattle	2008	Gyeonggi, South Korea
4	5	4	12	2	3	3	3	6	43	8	5	4	3	3	5	2009Her#100	B. abortus bv1	27	79		KBa089	Cattle	2008	Gyeonggi, South Korea
4	5	4	12	2	3	3	3	6	43	8	5	4	3	3	5	2009Her#101	B. abortus bv1	27	79		KBa090	Cattle	2008	Gyeonggi, South Korea
4	5	4	12	2	3	3	3	6	43	8	5	4	3	3	5	2009Her#120	B. abortus bv1	27	79		KBa109	Cattle	2004	Gyeonggi, South Korea
4	5	4	12	2	3	3	3	6	43	8	5	4	3	3	5	2009Her#122	B. abortus bv1	27	79		KBa111	Cattle	2004	Gyeonggi, South Korea
4	5	4	12	2	3	3	3	6	43	8	5	4	3	3	5	2009Her#124	B. abortus bv1	27	79		KBa113	Cattle	2004	Gyeonggi, South Korea
4	5	4	12	2	3	3	3	6	43	8	5	4	3	3	5	2009Her#168	B. abortus bv1	27	79		KBa157	Cattle	2006	Gyeonggi, South Korea
4	5	4	12	2	3	3	3	6	43	8	5	4	3	3	5	2009Her#182	B. abortus bv1	27	79		KBa171	Cattle	2008	Gyeonggi, South Korea
4	5	4	12	2	3	3	3	6	43	8	5	4	3	3	5	2009Her#183	B. abortus bv1	27	79		KBa172	Cattle	2008	Gyeonggi, South Korea
4	5	4	12	2	3	3	3	6	43	8	3	4	3	3	5	2006LeFleche#134	B. abortus bv1	27	79		BfR 91	Cattle	1996	Bern, Switzerland
4	5	4	12	2	3	3	3	6	43	8	3	4	3	3	5	2017Hernández-M.	B. abortus	27	79		baboCR52	Cattle	2005	Costa Rica
4	5	4	12	2	3	3	3	6	43	8	3	4	3	3	5	2017Hernández-M.	B. abortus	27	79		baboCR75	Cattle	2014	Costa Rica
4	5	4	12	2	3	3	3	6	43	8	3	4	3	3	5	2017Hernández-M.	B. abortus	27	79		baboCR90	Cattle	2015	Costa Rica
4	5	4	12	2	3	3	3	6	43	8	7	4	3	3	5	2017Hernández-M.	B. abortus	27	79		baboCR74	Cattle	2014	Costa Rica
4	5	4	12	2	4	3	3	6	43	8	5	4	3	3	6	2010Her#24	B. abortus	91	176		#56	Human	2006	Gyeongnam, South Ko.
4	5	4	12	2	4	3	3	6	43	8	5	4	3	3	6	2010Her#29	B. abortus	91	176		#72	Human	2006	Gyeonggi, South Korea
4	5	4	12	2	3	3	3	6	43	8	5	4	3	3	6	2009Her#080	B. abortus bv1	27	79		KBa069	Cattle	2007	Chungbuk, South Korea
4	5	4	12	2	3	3	3	6	43	8	5	4	3	3	6	2009Her#091	B. abortus bv1	27	79		KBa080	Cattle	2008	Gyeonggi, South Korea
4	5	4	12	2	3	3	3	6	43	8	5	4	3	3	6	2009Her#092	B. abortus bv1	27	79		KBa081	Cattle	2008	Gyeonggi, South Korea
4	5	4	12	2	3	3	3	6	43	8	5	4	3	3	6	2009Her#093	B. abortus bv1	27	79		KBa082	Cattle	2008	Gyeonggi, South Korea
4	5	4	12	2	3	3	3	6	43	8	5	4	3	3	6	2009Her#160	B. abortus bv1	27	79		KBa149	Cattle	2005	Chungnam, South Korea
4	5	4	12	2	3	3	3	6	43	8	5	4	3	3	6	2009Her#161	B. abortus bv1	27	79		KBa150	Cattle	2005	Chungnam, South Korea
4	5	4	12	2	3	3	3	6	43	8	5	4	3	3	6	2009Her#162	B. abortus bv1	27	79		KBa151	Cattle	2005	Chungnam, South Korea
4	5	4	12	2	3	3	3	6	43	8	5	4	3	3	6	2009Her#169	B. abortus bv1	27	79		KBa158	Cattle	2006	Kangwon, South Korea
4	5	4	12	2	3	3	3	6	43	8	5	4	3	3	6	2009Her#171	B. abortus bv1	27	79		KBa160	Cattle	2002	Gyeongbuk, South Korea
4	5	4	12	2	3	3	3	6	43	8	5	4	3	3	6	2010Her#14	B. abortus	27	79		#28	Human	2005	Gyeonggi, South Korea
4	5	4	12	2	3	3	3	6	43	8	5	4	3	3	6	2010Her#32	B. abortus	27	79		#84	Human	2007	Gyeongnam, South Ko.
4	5	4	12	2	3	3	3	6	43	8	5	4	3	3	6	2010Her#33	B. abortus	27	79		#85	Human	2007	Gyeongbuk, South Korea
4	5	4	12	2	4	3	3	6	43	8	5	5	3	3	6	2009Her#062	B. abortus bv1	91	176		KBa051	Cattle	2006	Gyeongbuk, South Korea
4	5	4	12	2	4	3	3	6	43	8	5	4	3	3	7	2009Her#069	B. abortus bv1	91	176		KBa058	Cattle	2007	Kangwon, South Korea
4	5	4	12	2	2	3	3	6	43	8	4	4	3	3	5	2009Her#133	B. abortus bv1	28	82		KBa122	Cattle	2005	Kangwon, South Korea
4	5	4	12	2	2	3	3	6	43	8	4	4	3	3	5	2017Hernández-M.	B. abortus	28	82		baboCR137	Cattle	2016	Costa Rica
4	5	4	12	2	2	3	3	6	43	8	4	4	3	3	5	2017Hernández-M.	B. abortus	28	82		baboCR139	Cattle	2016	Costa Rica
4	5	4	12	2	2	3	3	6	43	8	4	4	3	3	5	2017Hernández-M.	B. abortus	28	82		baboCR167	Cattle	2015	Costa Rica
4	5	4	12	2	5	3	3	6	43	8	4	4	3	3	5	2009Her#090	B. abortus bv1	89	174		KBa079	Cattle	2007	Gyeonggi, South Korea
4	5	4	12	2	6	3	3	6	43	8	4	4	3	3	5	2009Her#035	B. abortus bv1	90	175		KBa024	Cattle	2005	Gyeongnam, South Ko.
4	5	4	12	2	4	3	3	6	43	8	4	4	3	3	5	2009Her#059	B. abortus bv1	91	176		KBa048	Cattle	2006	Gyeongbuk, South Korea
4	5	4	12	2	2	3	3	6	43	8	4	3	3	3	5	2017Hernández-M.	B. abortus	28	82		baboCR111	Cattle	2015	Costa Rica
4	5	4	12	2	2	3	3	6	43	8	4	3	3	3	5	2017Hernández-M.	B. abortus	28	82		baboCR146	Cattle	2016	Costa Rica
4	5	4	12	2	2	3	3	6	43	8	4	3	3	3	5	2017Hernández-M.	B. abortus	28	82		baboCR147	Cattle	2016	Costa Rica
4	5	4	12	2	2	3	3	6	43	8	4	3	3	3	5	2017Hernández-M.	B. abortus	28	82		baboCR148	Cattle	2016	Costa Rica
4	5	4	12	2	2	3	3	6	43	8	4	3	3	3	5	2017Hernández-M.	B. abortus	28	82		baboCR149	Cattle	2016	Costa Rica
4	5	4	12	2	2	3	3	6	43	8	4	3	3	3	5	2017Hernández-M.	B. abortus	28	82		baboCR150	Cattle	2016	Costa Rica
4	5	4	12	2	2	3	3	6	43	8	4	3	3	3	5	2017Hernández-M.	B. abortus	28	82		baboCR162	Cattle	2016	Costa Rica
4	5	4	12	2	2	3	3	6	43	8	4	3	3	3	5	2017Hernández-M.	B. abortus	28	82		baboCR164	Cattle	2016	Costa Rica
4	5	4	12	2	2	3	3	6	43	8	4	3	3	3	5	2017Hernández-M.	B. abortus	28	82		baboCR93	Cattle	2015	Costa Rica
4	5	4	12	2	2	3	3	6	43	8	4	3	3	3	5	2017Hernández-M.	B. abortus	28	82		baboCR109	Cattle	2015	Costa Rica
4	5	4	12	2	2	3	3	6	43	8	4	3	3	3	5	2017Hernández-M.	B. abortus	28	82		baboCR110	Cattle	2015	Costa Rica
4	5	4	12	2	2	3	3	6	43	8	4	3	3	3	5	2017Hernández-M.	B. abortus	28	82		baboCR151	Cattle	2016	Costa Rica
4	5	4	12	2	2	3	3	5	43	8	4	3	3	3	5	2017Hernández-M.	B. abortus	28	83		baboCR114	Cattle	2015	Costa Rica
4	5	4	12	2	2	3	3	6	45	8	4	5	3	3	5	2013Minharro#032	B. abortus bv1	28	75		138	Cattle	2007	Esmeraldas, 35740000,.
4	5	4	12	2	2	3	3	6	43	8	4	7	3	3	4	2013Garofolo_20.	B. abortus bv1	28	82		20001	Buffalo	2011	Cancello ed Arnone,Italy
4	5	4	12	2	2	3	3	6	43	8	3	7	3	3	4	2013Garofolo_20.	B. abortus bv1	28	82		20823	Buffalo	2011	Grazzanise,Italy
4	5	4	12	2	2	3	3	6	43	8	4	4	3	3	4	2006LeFleche#103	B. abortus bv1	28	82		BCCN#95-19	Chamois	1995	Chambery, France
4	5	4	12	2	2	3	3	4	43	8	4	4	3	3	4	2006LeFleche#106	B. abortus bv1	28	81		BCCN#96-62	Cattle	1996	Sicilia, Italia
4	5	4	12	2	2	3	3	5	43	8	4	4	3	4	6	2013Minharro#094	B. abortus bv1	28	83		577	Cattle	1977	Porto Alegre, 90570020.
4	5	4	12	2	3	3	3	6	42	8	6	5	3	3	6	2017Hernández-M.	B. abortus	27	371		baboCR60	Cattle	2011	Costa Rica
4	5	4	12	2	3	3	3	6	42	8	6	5	3	3	6	2017Hernández-M.	B. abortus	27	371		baboCR59	Cattle	2010	Costa Rica
4	5	4	12	2	2	3	3	6	43	9	4	4	3	5	7	2013Minharro#087	B. abortus bv1	28	235		15_mar	Cattle	2003	Porto Alegre, 90570020.
4	5	4	12	2	2	3	3	6	43	9	4	4	3	5	7	2013Minharro#133	B. abortus bv2	28	235		SC4	Cattle		Florianopolis, 88010970.
4	5	4	12	2	2	3	3	6	43	8	4	4	3	5	7	2013Minharro#083	B. abortus bv1	28	82		13_mar	Cattle	2003	Porto Alegre, 90570020.
4	5	4	12	2	2	3	3	6	43	8	4	5	3	5	7	2013Garofolo_6055	B. abortus bv1	28	82		6055	Cattle	2011	Papasidero,Italy
4	5	4	12	2	2	3	3	6	43	8	4	5	3	5	7	2013Garofolo_9261	B. abortus bv1	28	82		9261	Cattle	2011	Papasidero,Italy
4	5	4	12	2	2	3	3	6	43	8	4	4	3	5	8	2013Minharro#065	B. abortus bv1	28	82		205	Cattle	2008	Floresta do Araguaia, 6.
4	5	4	12	2	2	3	3	6	43	8	3	4	3	5	8	2013Minharro#121	B. abortus bv1	28	82		1_1	Cattle		Florianopolis, 88010970.
3	5	4	12	2	2	3	3	6	43	8	4	4	3	5	6	2013Minharro#069	B. abortus bv2	33	76		209	Cattle	2008	Maraba, 68500005, Brazil
4	5	4	12	2	2	3	3	6	43	9	4	4	3	5	4	2013Minharro#101	B. abortus bv1	28	235		RS 6	Cattle	2006	Porto Alegre, 90570020.
4	5	4	12	2	2	3	3	6	43	9	4	4	3	6	4	2013Minharro#104	B. abortus bv1	28	235		RS 9	Cattle	2007	Porto Alegre, 90570020.
4	5	4	12	2	2	3	3	6	45	9	4	4	3	4	6	2013Minharro#084	B. abortus bv1	28	333		13a/02	Cattle	2002	Porto Alegre, 90570020.
4	5	4	12	2	2	3	3	6	43	9	4	4	3	4	6	2013Minharro#102	B. abortus bv1	28	235		RS 7	Cattle	2007	Porto Alegre, 90570020.
4	5	4	12	2	2	3	3	6	43	9	3	4	3	3	7	2013Minharro#063	B. abortus bv1	28	235		202	Cattle	2008	Itaguatins, 77920000, B.
4	5	4	12	2	2	3	3	6	43	8	4	6	3	6	4	2013Garofolo_13.	B. abortus bv1	28	82		13928	Cattle	2011	Sasso di Castalda,Italy
4	5	4	12	2	2	3	3	6	43	8	4	6	3	6	4	SRR1371372	B. abortus	28	82	1	B94-0609	Cattle		United States - MO
4	5	4	12	2	2	3	3	6	43	8	4	6	3	7	4	2013Garofolo_2647	B. abortus bv3	28	82		2647	Cattle	2011	Cirigliano,Italy
4	5	4	12	2	2	3	3	6	43	8	5	4	3	6	4	2006LeFleche#138	B. abortus bv1	28	82		BfR 98	unknown	1972	Lagesbuttel, Germany
4	5	3	12	2	2	3	3	6	43	8	4	4	3	7	4	SRR1371306	B. abortus	112	210	1	B02-0040	Cattle		United States - TX
4	5	4	12	1	2	3	3	6	43	8	3	4	3	5	5	2012Ferreira#189	B. abortus bv1	32	182		LNIV-395Ba1-06	Cattle	2006	S. Jorge, Azores, Portu.
4	5	4	12	1	2	3	3	6	43	8	3	4	3	5	5	2012Ferreira#190	B. abortus bv1	32	182		LNIV-396Ba1-06	Cattle	2006	S. Jorge, Azores, Portu.
4	5	4	12	1	2	3	3	6	43	8	3	4	3	5	5	2012Ferreira#195	B. abortus bv1	32	182		LNIV-402Ba1-96	Cattle	2006	S. Jorge, Azores, Portu.
4	5	4	12	1	2	3	3	6	43	8	3	4	3	5	5	2012Ferreira#205	B. abortus bv1	32	182		LNIV-414Ba1-07	Cattle	2007	S. Miguel, Azores, Port.

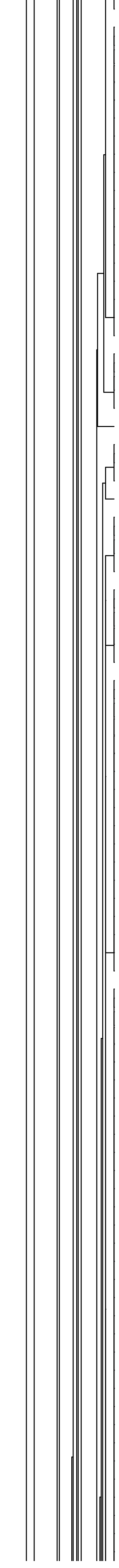

4	5	4	12	1	2	3	3	6	43	8	3	4	3	5	5	2012Ferreira#206	B. abortus bv1	32	182		LNIV-415Ba1-07	Cattle	2007	S. Miguel, Azores, Port.
4	5	4	12	1	2	3	3	6	43	8	3	4	3	4	5	2012Ferreira#178	B. abortus bv1	32	182		LNIV-362Ba1-07	Cattle	2007	Alentejo, Portugal
4	5	4	12	1	2	3	3	6	43	8	3	4	3	4	5	2012Ferreira#179	B. abortus bv1	32	182		LNIV-373Ba1-07	Cattle	2007	Alentejo, Portugal
4	5	4	12	1	2	3	3	6	43	8	3	4	3	4	5	2012Ferreira#187	B. abortus bv1	32	182		LNIV-393Ba1-06	Cattle	2006	S. Jorge, Azores, Portu.
4	5	4	12	1	2	3	3	6	43	8	3	4	3	4	5	2012Ferreira#188	B. abortus bv1	32	182		LNIV-394Ba1-06	Cattle	2006	S. Jorge, Azores, Portu.
4	5	4	12	1	2	3	3	6	43	8	3	4	3	4	5	2012Ferreira#191	B. abortus bv1	32	182		LNIV-397Ba1-06	Cattle	2006	S. Jorge, Azores, Portu.
4	5	4	12	1	2	3	3	6	43	8	3	4	3	4	5	2012Ferreira#192	B. abortus bv1	32	182		LNIV-398Ba1-06	Cattle	2006	S. Jorge, Azores, Portu.
4	5	4	12	1	2	3	3	6	43	8	3	4	3	4	5	2012Ferreira#193	B. abortus bv1	32	182		LNIV-400Ba1-06	Cattle	2006	S. Jorge, Azores, Portu.
4	5	4	12	1	2	3	3	6	43	8	3	4	3	4	5	2012Ferreira#194	B. abortus bv1	32	182		LNIV-401Ba1-06	Cattle	2006	S. Jorge, Azores, Portu.
4	5	4	12	1	2	3	3	6	43	8	3	4	3	4	5	2012Ferreira#197	B. abortus bv1	32	182		LNIV-404Ba1-06	Cattle	2006	S. Jorge, Azores, Portu.
4	5	4	12	1	2	3	3	6	43	8	3	4	3	4	5	2012Ferreira#198	B. abortus bv1	32	182		LNIV-405Ba1-06	Cattle	2006	S. Jorge, Azores, Portu.
4	5	4	12	1	2	3	3	6	43	8	3	4	3	4	5	2012Ferreira#199	B. abortus bv1	32	182		LNIV-406Ba1-06	Cattle	2006	S. Jorge, Azores, Portu.
4	5	4	12	1	2	3	3	6	43	8	3	4	3	4	5	2012Ferreira#200	B. abortus bv1	32	182		LNIV-407Ba1-06	Cattle	2006	S. Jorge, Azores, Portu.
4	5	4	12	1	2	3	3	6	43	8	3	4	3	4	5	2012Ferreira#201	B. abortus bv1	32	182		LNIV-408Ba1-07	Cattle	2007	S. Jorge, Azores, Portu.
4	5	4	12	1	2	3	3	6	43	8	3	4	3	4	5	2012Ferreira#202	B. abortus bv1	32	182		LNIV-409Ba1-07	Cattle	2007	S. Jorge, Azores, Portu.
4	5	4	12	1	2	3	3	6	43	8	3	4	3	4	5	2012Ferreira#203	B. abortus bv1	32	182		LNIV-431Ba1-07	Cattle	2007	S. Jorge, Azores, Portu.
4	5	4	12	1	2	3	3	6	43	8	3	4	3	4	5	2012Ferreira#204	B. abortus bv1	32	182		LNIV-435Ba1-07	Cattle	2007	S. Jorge, Azores, Portu.
4	5	4	12	1	2	3	3	6	43	8	3	4	3	4	5	2012Ferreira#207	B. abortus bv1	32	182		LNIV-421Ba1-07	Cattle	2007	S. Miguel, Azores, Port.
4	5	4	12	1	2	3	3	6	43	8	3	4	3	4	5	2012Ferreira#208	B. abortus bv1	32	182		LNIV-410Ba1-07	Cattle	2007	Terceira, Azores, Portu.
4	5	4	12	1	2	3	3	6	43	8	3	4	3	4	6	2012Ferreira#184	B. abortus bv1	32	182		LNIV-390Ba1-06	Cattle	2006	S. Jorge, Azores, Portu.
4	5	4	12	1	2	3	3	6	43	8	3	4	3	4	6	2012Ferreira#185	B. abortus bv1	32	182		LNIV-391Ba1-06	Cattle	2006	S. Jorge, Azores, Portu.
4	5	4	12	1	2	3	3	6	43	8	3	4	3	4	6	2012Ferreira#186	B. abortus bv1	32	182		LNIV-392Ba1-06	Cattle	2006	S. Jorge, Azores, Portu.
4	5	4	12	1	2	3	3	6	43	8	3	4	3	4	6	2012Ferreira#196	B. abortus bv1	32	182		LNIV-403Ba1-06	Cattle	2006	S. Jorge, Azores, Portu.
4	5	4	12	1	2	3	3	6	43	8	3	5	3	5	5	SRR3096436	B. abortus	32	182		B11-0259	Cattle		USA: Wyoming
4	5	4	12	2	2	3	3	6	43	8	3	5	3	4	6	2006LeFleche#139	B. abortus bv1	28	82		BfR 99	unknown	1972	Bern, Switzerland
4	5	4	12	2	2	3	3	6	43	8	3	5	3	4	6	2012Ferreira#296	B. abortus bv1	28	82		REF 99W	Cattle		UK
4	5	4	12	2	2	3	3	6	43	8	3	5	3	4	6	SRR1371386	B. abortus	28	82	1	B99-0074	Cattle		United States - MO
4	5	4	12	2	2	3	3	6	43	8	3	3	3	4	6	SRR1371359	B. abortus	28	82	1	B12-0429	Bison		United States - WY
4	5	4	12	2	2	3	3	6	43	8	3	4	3	3	6	2013Minharro#111	B. abortus bv1	28	82		SP 6	Cattle		Sao Paulo, 01036970, .
4	5	4	12	2	2	3	3	6	43	8	3	4	3	3	6	2017Hernández-M.	B. abortus	28	82		baboCR158	Cattle	2015	Costa Rica
4	5	4	12	2	2	3	3	6	43	8	3	4	3	3	6	2017Hernández-M.	B. abortus	28	82		baboCR159	Cattle	2015	Costa Rica
4	5	4	12	2	2	3	3	6	43	8	3	4	3	3	6	2018Vergnaud#0227	B. abortus Rough	28	82		BCCN#02-45	Buffalo	2002	India
4	5	4	12	2	2	3	3	6	43	8	3	4	3	5	6	2013Minharro#110	B. abortus bv1	28	82		SP 5	Cattle		Sao Paulo, 01036970, .
4	5	4	12	2	2	3	3	6	43	8	3	4	3	5	6	2013Minharro#113	B. abortus bv1	28	82		SP 8	Cattle		Sao Paulo, 01036970, .
4	5	4	12	2	2	3	3	6	43	8	3	4	3	5	6	2013Minharro#124	B. abortus bv2	28	82		5_3	Cattle		Belo Horizonte, 30001.
4	5	4	12	2	2	3	3	6	43	8	3	4	3	5	6	SRR3096409	B. abortus	28	82	1	B-102110002TD	Deer/El.		USA: Wyoming
4	5	4	12	2	2	3	3	6	43	8	3	4	3	5	6	SRR3096456	B. abortus	28	82	1	B-11121001	Deer/El.		USA: Wyoming
4	5	4	12	2	2	3	3	6	43	8	3	4	3	6	6	2012Ferreira#266	B. abortus bv1	28	82		LNIV-412Ba1-07	Cattle	2007	S. Miguel, Azores, Port.
4	5	4	12	2	2	3	3	6	43	8	3	4	3	6	6	GCA_000008145	B. abortus bv1	28	82	1	9-941	Cattle	2005	Wyoming, USA
4	5	4	12	2	2	3	3	6	43	8	3	4	3	6	6	SRR1371328	B. abortus	28	82	1	B11-0208	Cattle		United States - WY
4	5	4	12	2	2	3	3	6	43	8	3	4	3	6	6	SRR1371329	B. abortus	28	82	1	B11-0210	Cattle		United States - WY
4	5	4	12	2	2	3	3	6	43	8	3	4	3	6	6	SRR1371330	B. abortus	28	82	1	B11-0245	Cattle		United States - WY
4	5	4	12	2	2	3	3	6	43	8	3	4	3	6	6	SRR1371332	B. abortus	28	82	1	B12-0002	Cattle		United States - WY
4	5	4	12	2	2	3	3	6	43	8	3	4	3	6	6	SRR1371333	B. abortus	28	82	1	B12-0025	Cattle		United States - WY
4	5	4	12	2	2	3	3	6	43	8	3	4	3	6	6	SRR1371364	B. abortus	28	82	1	B89-0941	Cattle		United States - WY
4	5	4	12	2	2	3	3	6	43	8	3	4	3	6	6	SRR1371365	B. abortus	28	82	1	B89-0944	Cattle		United States - WY
4	5	4	12	2	2	3	3	6	43	8	3	4	3	6	6	SRR3096334	B. abortus	28	82	1	B02-0204	Cattle		USA: Idaho
4	5	4	12	2	2	3	3	6	43	8	3	4	3	6	6	SRR3096335	B. abortus	28	82	1	B02-0235	Cattle		USA: Idaho
4	5	4	12	2	2	3	3	6	43	8	3	4	3	6	6	SRR3096336	B. abortus	28	82	1	B02-0236	Cattle		USA: Idaho
4	5	4	12	2	2	3	3	6	43	8	3	4	3	6	6	SRR3096337	B. abortus	28	82	1	B02-0237	Cattle		USA: Idaho
4	5	4	12	2	2	3	3	6	43	8	3	4	3	6	6	SRR3096338	B. abortus	28	82	1	B02-0239	Cattle		USA: Idaho
4	5	4	12	2	2	3	3	6	43	8	3	4	3	6	6	SRR3096339	B. abortus	28	82	1	B02-0241	Cattle		USA: Idaho
4	5	4	12	2	2	3	3	6	43	8	3	4	3	6	6	SRR3096340	B. abortus	28	82	1	B02-0242	Cattle		USA: Idaho
4	5	4	12	2	2	3	3	6	43	8	3	4	3	6	6	SRR3096443	B. abortus	28	82		B11-0273	Deer/El.		USA: Wyoming
4	5	4	12	2	2	3	3	6	43	8	3	4	3	4	6	2012Ferreira#241	B. abortus bv1	28	82		LNIV-372Ba1-07	Cattle	2007	Alentejo, Portugal
4	5	4	12	2	2	3	3	6	43	8	3	4	3	4	6	2012Ferreira#245	B. abortus bv1	28	82		LNIV-378Ba1-07	Cattle	2007	Alentejo, Portugal
4	5	4	12	2	2	3	3	6	43	8	3	4	3	4	6	2012Ferreira#248	B. abortus bv1	28	82		LNIV-243Ba1-05	Cattle	2005	Beira Interior, Portugal
4	5	4	12	2	2	3	3	6	43	8	3	4	3	4	6	2012Ferreira#249	B. abortus bv1	28	82		LNIV-246Ba1-05	Cattle	2005	Beira Interior, Portugal
4	5	4	12	2	2	3	3	6	43	8	3	4	3	4	6	2012Ferreira#251	B. abortus bv1	28	82		LNIV-DNA181.	Cattle	2008	Beira Interior, Portugal
4	5	4	12	2	2	3	3	6	43	8	3	4	3	4	6	2012Ferreira#252	B. abortus bv1	28	82		LNIV-DNA182.	Cattle	2008	Beira Interior, Portugal
4	5	4	12	2	2	3	3	6	43	8	3	4	3	4	6	2012Ferreira#253	B. abortus bv1	28	82		LNIV-DNA183.	Cattle	2008	Beira Interior, Portugal
4	5	4	12	2	2	3	3	6	43	8	3	4	3	4	6	2012Ferreira#254	B. abortus bv1	28	82		LNIV-DNA184.	Cattle	2008	Beira Interior, Portugal
4	5	4	12	2	2	3	3	6	43	8	3	4	3	4	6	2012Ferreira#270	B. abortus bv1	28	82		LNIV-418Ba1-07	Cattle	2007	S. Miguel, Azores, Port.
4	5	4	12	2	2	3	3	6	43	8	3	4	3	4	6	2012Ferreira#280	B. abortus bv1	28	82		LNIV-429Ba1-07	Cattle	2007	S. Miguel, Azores, Port.
4	5	4	12	2	2	3	3	6	43	8	3	4	3	4	6	2012Ferreira#281	B. abortus bv1	28	82		LNIV-430Ba1-07	Cattle	2007	S. Miguel, Azores, Port.
4	5	4	12	2	2	3	3	6	43	8	3	4	3	4	6	2012Ferreira#283	B. abortus bv1	28	82		LNIV-433Ba1-07	Cattle	2007	S. Miguel, Azores, Port.
4	5	4	12	2	2	3	3	6	43	8	3	4	3	4	6	2012Ferreira#285	B. abortus bv1	28	82		LNIV-440Ba1-07	Cattle	2007	S. Miguel, Azores, Port.
4	5	4	12	2	2	3	3	6	43	8	3	4	3	4	6	2012Ferreira#292	B. abortus bv1	28	82		LNIV-443Ba1-07	Cattle	2007	S. Miguel, Azores, Port.
4	5	4	12	2	2	3	3	6	43	8	3	4	3	4	6	2013Minharro#119	B. abortus bv2	28	82		Paraí	Cattle		Belo Horizonte, 30001.
4	5	4	12	2	2	3	3	6	43	8	3	4	3	4	6	2013Minharro#128	B. abortus bv1	28	82		393-11	Cattle		Belo Horizonte, 30001.
4	5	4	12	2	2	3	3	6	43	8	3	4	3	4	6	2013Minharro#129	B. abortus bv2	28	82		Flor	Cattle		Belo Horizonte, 30001.
4	5	4	12	2	2	3	3	6	43	8	3	4	3	4	6	SRR1371310	B. abortus	28	82	1	B02-0219	Deer/El.		United States - ID
4	5	4	12	2	2	3	3	6	43	8	3	4	3	4	6	SRR1371326	B. abortus	28	82	1	B11-0053	Bison		United States - WY
4	5	4	12	2	2	3	3	6	43	8	3	4	3	4	6	SRR1371335	B. abortus	28	82	1	B12-0219	Bison		United States - WY
4	5	4	12	2	2	3	3	6	43	8	3	4	3	4	6	SRR1371336	B. abortus	28	82	1	B12-0228	Bison		United States - WY
4	5	4	12	2	2	3	3	6	43	8	3	4	3	4	6	SRR1371337	B. abortus	28	82	1	B12-0230	Bison		United States - WY
4	5	4	12	2	2	3	3	6	43	8	3	4	3	4	6	SRR1371338	B. abortus	28	82	1	B12-0232	Bison		United States - WY
4	5	4	12	2	2	3	3	6	43	8	3	4	3	4	6	SRR1371339	B. abortus	28	82	1	B12-0233	Bison		United States - WY
4	5	4	12	2	2	3	3	6	43	8	3	4	3	4	6	SRR1371340	B. abortus	28	82	1	B12-0234	Bison		United States - WY
4	5	4	12	2	2	3	3	6	43	8	3	4	3	4	6	SRR1371341	B. abortus	28	82	1	B12-0235	Bison		United States - WY
4	5	4	12	2	2	3	3	6	43	8	3	4	3	4	6	SRR1371342	B. abortus	28	82	1	B12-0238	Bison		United States - WY
4	5	4	12	2	2	3	3	6	43	8	3	4	3	4	6	SRR1371344	B. abortus	28	82	1	B12-0241	Bison		United States - WY
4	5	4	12	2	2	3	3	6	43	8	3	4	3	4	6	SRR1371345	B. abortus	28	82	1	B12-0242	Bison		United States - WY
4	5	4	12	2	2	3	3	6	43	8	3	4	3	4	6	SRR1371346	B. abortus	28	82	1	B12-0243	Bison		United States - WY
4	5	4	12	2	2	3	3	6	43	8	3	4	3	4	6	SRR1371347	B. abortus	28	82	1	B12-0250	Bison		United States - WY
4	5	4	12	2	2	3	3	6	43	8	3	4	3	4	6	SRR1371348	B. abortus	28	82	1	B12-0252	Bison		United States - WY

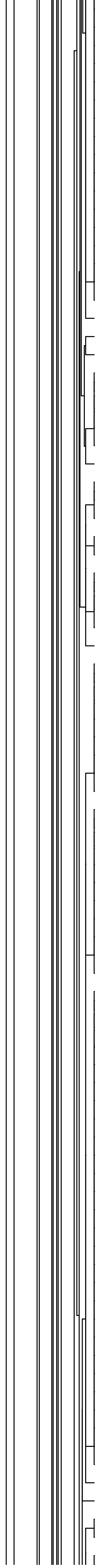

4	5	4	12	2	2	3	3	6	43	8	3	4	3	4	6	SRR1371349	B. abortus	28	82	1	B12-0256	Bison		United States - WY
4	5	4	12	2	2	3	3	6	43	8	3	4	3	4	6	SRR1371350	B. abortus	28	82	1	B12-0266	Bison		United States - WY
4	5	4	12	2	2	3	3	6	43	8	3	4	3	4	6	SRR1371351	B. abortus	28	82	1	B12-0268	Bison		United States - WY
4	5	4	12	2	2	3	3	6	43	8	3	4	3	4	6	SRR1371352	B. abortus	28	82	1	B12-0270	Bison		United States - WY
4	5	4	12	2	2	3	3	6	43	8	3	4	3	4	6	SRR1371353	B. abortus	28	82	1	B12-0271	Bison		United States - WY
4	5	4	12	2	2	3	3	6	43	8	3	4	3	4	6	SRR1371354	B. abortus	28	82	1	B12-0275	Bison		United States - WY
4	5	4	12	2	2	3	3	6	43	8	3	4	3	4	6	SRR1371355	B. abortus	28	82	1	B12-0276	Bison		United States - WY
4	5	4	12	2	2	3	3	6	43	8	3	4	3	4	6	SRR1371356	B. abortus	28	82	1	B12-0277	Bison		United States - WY
4	5	4	12	2	2	3	3	6	43	8	3	4	3	4	6	SRR1371358	B. abortus	28	82	1	B12-0425	Bison		United States - WY
4	5	4	12	2	2	3	3	6	43	8	3	4	3	4	6	SRR1371360	B. abortus	28	82	1	B12-0431	Bison		United States - WY
4	5	4	12	2	2	3	3	6	43	8	3	4	3	4	6	SRR3096345	B. abortus	28	82	1	B03-0202	Deer/El.		USA: Idaho
4	5	4	12	2	2	3	3	6	43	8	3	4	3	4	6	SRR3096363	B. abortus	28	82	1	B06-0031	Cattle		USA: Idaho
4	5	4	12	2	2	3	3	6	43	8	3	4	3	4	6	SRR3096441	B. abortus	28	82		B11-0270	Deer/El.		USA: Wyoming
4	5	4	12	2	2	3	3	6	43	8	3	4	3	4	6	SRR3096442	B. abortus	28	82	1	B11-0271	Deer/El.		USA: Wyoming
4	5	4	12	2	2	3	3	6	43	8	3	4	3	4	6	SRR3096472	B. abortus	28	82	1	B12-0407	Deer/El.		USA: Wyoming
4	5	4	12	2	2	3	3	6	43	8	3	4	3	4	6	SRR3096473	B. abortus	28	82	1	B12-0408	Deer/El.		USA: Wyoming
4	5	4	12	2	2	3	3	6	43	8	3	4	3	4	6	SRR3096475	B. abortus	28	82	1	B12-0410	Deer/El.		USA: Wyoming
4	5	4	12	2	2	3	3	6	43	8	3	4	3	7	6	SRR3096404	B. abortus	28	82	1	B-10061002	Deer/El.		USA: Wyoming
4	5	4	12	2	2	3	3	6	43	8	3	4	3	5	4	2012Ferreira#286	B. abortus bv1	28	82		LNIV-441Ba1-07	Cattle	2007	S. Miguel, Azores, Port.
4	5	4	12	2	2	3	3	6	43	8	3	4	3	3	4	2013Garofolo_8979	B. abortus bv1	28	82		8979	Buffalo	2011	Cancello ed Arnone,Italy
4	5	4	12	2	2	3	3	6	43	8	3	4	3	4	4	2013Minharro#014	B. abortus bv1	28	82		42	Cattle	2006	Governador Valadares,.
4	5	4	12	2	2	3	3	6	43	8	3	4	3	4	4	2013Minharro#015	B. abortus bv1	28	82		43	Cattle	2006	Governador Valadares,.
4	5	4	12	2	2	3	3	6	43	8	3	4	3	4	4	2013Minharro#016	B. abortus bv1	28	82		44	Cattle	2006	Governador Valadares,.
4	5	4	12	2	2	3	3	6	43	8	3	4	3	4	4	2013Minharro#018	B. abortus bv1	28	82		46	Cattle	2006	Governador Valadares,.
4	5	4	12	2	2	3	3	6	43	8	3	4	3	4	4	2013Minharro#019	B. abortus bv2	28	82		47	Cattle	2006	Governador Valadares,.
4	5	4	12	2	2	3	3	6	43	8	3	4	3	4	8	SRR1371388	B. abortus	28	82	1	B99-1033	Cattle		United States - TX
4	5	4	12	2	2	3	3	6	43	8	3	4	3	6	7	SRR1371309	B. abortus	28	82	1	B02-0187	Cattle		United States - ID
4	5	4	12	2	2	3	3	6	43	8	3	4	3	6	7	SRR1371323	B. abortus	28	82	1	B11-0015	Cattle		United States - WY
4	5	4	12	2	2	3	3	6	43	8	3	4	3	6	7	SRR1371325	B. abortus	28	82	1	B11-0038	Cattle		United States - WY
4	5	4	12	2	2	3	3	6	43	8	3	4	3	5	7	2013Minharro#125	B. abortus bv1	28	82		6_1	Cattle		Belo Horizonte, 30001.
4	5	4	12	2	2	3	3	6	43	8	3	4	3	5	7	2013Minharro#130	B. abortus bv1	28	82		SC1	Cattle		Florianopolis, 88010970.
4	5	4	12	2	2	3	3	6	43	8	3	4	3	4	7	2012Ferreira#250	B. abortus bv1	28	82		LNIV-385Ba1-07	Cattle	2007	Beira Interior, Portugal
4	5	4	12	2	2	3	3	6	43	8	3	4	3	4	7	2013Minharro#096	B. abortus bv1	28	82		Ba96	Cattle	1977	Porto Alegre, 90570020.
4	5	4	12	2	2	3	3	6	43	8	3	4	3	4	7	SRR1371305	B. abortus	28	82	1	B01-0650	Cattle		United States - AR
4	5	4	12	2	2	3	3	6	43	8	3	4	3	4	7	SRR1371343	B. abortus	28	82		B12-0240	Bison		United States - WY
4	5	4	12	2	2	3	3	6	43	8	3	4	3	3	7	2013Minharro#056	B. abortus bv1	28	82		194	Cattle	2008	Conceicao do Araguaia,.
4	5	4	12	2	2	3	3	6	43	8	3	5	3	5	5	SRR3096414	B. abortus	28	82	1	B11-0143	Deer/El.		USA: Wyoming
4	5	4	12	2	2	3	3	6	43	8	3	5	3	5	5	SRR3096416	B. abortus	28	82	1	B11-0145	Deer/El.		USA: Wyoming
4	5	4	12	2	2	3	3	6	43	8	3	5	3	5	5	SRR3096431	B. abortus	28	82	1	B11-0254	Bison		USA: Wyoming
4	5	4	12	2	2	3	3	6	43	8	3	5	3	5	5	SRR3096435	B. abortus	28	82	1	B11-0258	Bison		USA: Wyoming
4	5	4	12	2	2	3	3	6	43	8	3	5	3	5	5	SRR3096437	B. abortus	28	82	1	B11-0260	Bison		USA: Wyoming
4	5	4	12	2	2	3	3	6	43	8	3	5	3	5	5	SRR3096439	B. abortus	28	82	1	B11-0262	Bison		USA: Wyoming
4	5	4	12	2	2	3	3	6	43	8	3	5	3	5	5	SRR3096484	B. abortus	28	82	1	B12-0419	Bison		USA: Wyoming
4	5	4	12	2	2	3	3	6	43	8	3	5	3	5	5	SRR3096497	B. abortus	28	82	1	B13-0090	Bison		USA: Montana
4	5	4	12	2	2	3	3	6	43	8	3	6	3	5	5	SRR3096329	B. abortus	28	82	1	B01-0505	Bison		USA: Wyoming
4	5	4	12	2	2	3	3	6	43	8	3	6	3	5	5	SRR3096383	B. abortus	28	82	1	B09-0286	Deer/El.		USA: Montana
4	5	4	12	2	2	3	3	6	43	8	3	6	3	5	5	SRR3096450	B. abortus	28	82		B11-0488	Bison		USA: Montana
4	5	4	12	2	2	3	3	6	43	8	3	6	3	5	5	SRR3096451	B. abortus	28	82		B11-0574	Bison		USA: Montana
4	5	4	12	2	2	3	3	6	43	8	3	6	3	5	5	SRR3096485	B. abortus	28	82		B12-0460	Bison		USA: Montana
4	5	4	12	2	2	3	3	6	43	8	3	6	3	5	5	SRR3096493	B. abortus	28	82	1	B13-0061	Bison		USA: Montana
4	5	4	12	2	2	3	3	6	43	8	3	6	3	5	5	SRR3096494	B. abortus	28	82	1	B13-0083	Bison		USA: Montana
4	5	4	12	2	2	3	3	6	43	8	3	6	3	5	5	SRR3096495	B. abortus	28	82	1	B13-0086	Bison		USA: Montana
4	5	4	12	2	2	3	3	6	43	8	3	6	3	5	5	SRR3096498	B. abortus	28	82	1	B13-0091	Bison		USA: Montana
4	5	4	12	2	2	3	3	6	43	8	3	6	3	5	5	SRR3096500	B. abortus	28	82	1	B13-0097	Bison		USA: Montana
4	5	4	12	2	2	3	3	6	43	8	3	4	3	5	5	2013Minharro#075	B. abortus bv1	28	82		223	Cattle	2008	Ituitaba, 38300001, Brazil
4	5	4	12	2	2	3	3	6	43	8	3	4	3	5	5	2013Minharro#076	B. abortus bv1	28	82		224	Cattle	2008	Ituitaba, 38300001, Brazil
4	5	4	12	2	2	3	3	6	43	8	3	4	3	5	5	2013Minharro#112	B. abortus bv1	28	82		SP 7	Cattle		Sao Paulo, 01036970, .
4	5	4	12	2	2	3	3	6	43	8	3	4	3	5	5	2013Minharro#116	B. abortus bv1	28	82		Botucatu	Cattle	2007	Sao Paulo, 01036970, .
4	5	4	12	2	2	3	3	6	43	8	3	4	3	5	5	2013Minharro#117	B. abortus bv1	28	82		GM lanag	Cattle	2007	Florianopolis, 88010970.
4	5	4	12	2	2	3	3	6	43	8	3	4	3	5	5	2013Minharro#118	B. abortus bv1	28	82		Pintadinha	Cattle		Florianopolis, 88010970.
4	5	4	12	2	2	3	3	6	43	8	3	4	3	5	5	2013Minharro#120	B. abortus bv1	28	82		Bawla	Cattle		Florianopolis, 88010970.
4	5	4	12	2	2	3	3	6	43	8	3	4	3	5	5	2013Minharro#122	B. abortus bv1	28	82		5_1	Cattle		Belo Horizonte, 30001.
4	5	4	12	2	2	3	3	6	43	8	3	4	3	5	5	2013Minharro#123	B. abortus bv2	28	82		5_2	Cattle		Belo Horizonte, 30001.
4	5	4	12	2	2	3	3	6	43	8	3	4	3	5	5	SRR1371322	B. abortus	28	82	1	B11-0011	Bison		United States - MT
4	5	4	12	2	2	3	3	6	43	8	3	4	3	5	5	SRR3096343	B. abortus	28	82	1	B-02W2693	Deer/El.		USA: Wyoming
4	5	4	12	2	2	3	3	6	43	8	3	4	3	5	5	SRR3096351	B. abortus	28	82	1	B04-0224	Deer/El.		USA: Wyoming
4	5	4	12	2	2	3	3	6	43	8	3	4	3	5	5	SRR3096352	B. abortus	28	82	1	B04-0225	Deer/El.		USA: Wyoming
4	5	4	12	2	2	3	3	6	43	8	3	4	3	5	5	SRR3096384	B. abortus	28	82	1	B09-0295	Deer/El.		USA: Montana
4	5	4	12	2	2	3	3	6	43	8	3	4	3	5	5	SRR3096385	B. abortus	28	82	1	B09-0443	Deer/El.		USA: Montana
4	5	4	12	2	2	3	3	6	43	8	3	4	3	5	5	SRR3096389	B. abortus	28	82	1	B10-0091	Deer/El.		USA: Montana
4	5	4	12	2	2	3	3	6	43	8	3	4	3	5	5	SRR3096399	B. abortus	28	82	1	B10-0514	Deer/El.		USA: Montana
4	5	4	12	2	2	3	3	6	43	8	3	4	3	5	5	SRR3096410	B. abortus	28	82		B11-0131	Deer/El.		USA: Montana
4	5	4	12	2	2	3	3	6	43	8	3	4	3	5	5	SRR3096418	B. abortus	28	82	1	B11-0147	Deer/El.		USA: Wyoming
4	5	4	12	2	2	3	3	6	43	8	3	4	3	5	5	SRR3096447	B. abortus	28	82	1	B11-0469	Bison		USA: Montana
4	5	4	12	2	2	3	3	6	43	8	3	4	3	5	5	SRR3096448	B. abortus	28	82		B11-0473	Bison		USA: Montana
4	5	4	12	2	2	3	3	6	43	8	3	4	3	5	5	SRR3096487	B. abortus	28	82	1	B12-0578	Bison		USA: Montana
4	5	4	12	2	2	3	3	6	43	8	3	4	3	5	5	SRR3096488	B. abortus	28	82	1	B12-0583	Bison		USA: Montana
4	5	4	12	2	2	3	3	6	43	8	3	4	3	5	5	SRR3096489	B. abortus	28	82	1	B12-0585	Bison		USA: Montana
4	5	4	12	2	2	3	3	6	43	8	3	4	3	5	5	SRR3096490	B. abortus	28	82	1	B12-0587	Bison		USA: Montana
4	5	4	12	2	2	3	3	6	43	8	3	4	3	5	5	SRR3096492	B. abortus	28	82	1	B12-0602	Bison		USA: Montana
4	5	4	12	2	2	3	3	6	43	8	3	4	3	5	5	SRR3096496	B. abortus	28	82	1	B13-0088	Bison		USA: Montana
4	5	4	12	2	2	3	3	6	43	8	3	7	3	5	5	SRR1371307	B. abortus	28	82		B02-0120	Cattle		United States - OK
4	5	4	12	2	2	3	3	6	43	9	3	4	3	5	5	2013Minharro#049	B. abortus bv2	28	235		181	Cattle	2008	Pequizeiro, 77730000, .
4	5	4	12	2	2	3	3	6	43	8	3	4	3	9	5	SRR3096412	B. abortus	28	82	1	B11-0141	Bison		USA: Wyoming
4	5	4	12	2	2	3	3	6	43	8	3	4	3	9	5	SRR3096425	B. abortus	28	82	1	B11-0248	Bison		USA: Wyoming
4	5	4	12	2	2	3	3	6	43	8	3	4	3	7	5	SRR1371367	B. abortus	28	82	1	B93-0310	Cattle		United States - MO

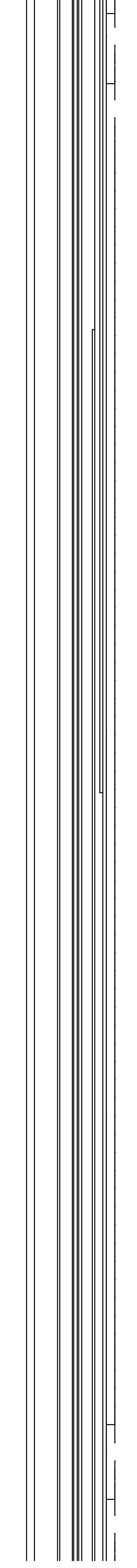

4	5	4	12	2	2	3	3	6	43	8	3	4	3	7	5	SRR3096354	B. abortus	28	82	1	B05-0018	Bison		USA: Wyoming
4	5	4	12	2	2	3	3	6	43	8	3	4	3	7	5	SRR3096478	B. abortus	28	82	1	B12-0413	Deer/El.		USA: Wyoming
4	5	4	12	2	2	3	3	6	43	8	3	4	3	8	5	SRR1371334	B. abortus	28	82	1	B12-0028	Bison		United States - MT
4	5	4	12	2	2	3	3	6	43	8	3	4	3	8	5	SRR3096355	B. abortus	28	82	1	B05-0019	Bison		USA: Wyoming
4	5	4	12	2	2	3	3	6	43	8	3	4	3	8	5	SRR3096394	B. abortus	28	82	1	B10-0252	Deer/El.		USA: Montana
4	5	4	12	2	2	3	3	6	43	8	3	4	3	8	5	SRR3096402	B. abortus	28	82	1	B10-0566	Deer/El.		USA: Montana
4	5	4	12	2	2	3	3	6	43	8	3	4	3	4	5	2012Ferreira#225	B. abortus bv1	28	82		LNIV-293Ba1-05	Cattle	2005	Alentejo, Portugal
4	5	4	12	2	2	3	3	6	43	8	3	4	3	4	5	2012Ferreira#226	B. abortus bv1	28	82		LNIV-294Ba1-05	Cattle	2005	Alentejo, Portugal
4	5	4	12	2	2	3	3	6	43	8	3	4	3	4	5	2012Ferreira#227	B. abortus bv1	28	82		LNIV-295Ba1-05	Cattle	2005	Alentejo, Portugal
4	5	4	12	2	2	3	3	6	43	8	3	4	3	4	5	2012Ferreira#228	B. abortus bv1	28	82		LNIV-94Ba1-03	Cattle	2003	Alentejo, Portugal
4	5	4	12	2	2	3	3	6	43	8	3	4	3	4	5	2012Ferreira#229	B. abortus bv1	28	82		LNIV-212Ba1-04	Cattle	2004	Alentejo, Portugal
4	5	4	12	2	2	3	3	6	43	8	3	4	3	4	5	2012Ferreira#230	B. abortus bv1	28	82		LNIV-352Ba1-07	Cattle	2007	Alentejo, Portugal
4	5	4	12	2	2	3	3	6	43	8	3	4	3	4	5	2012Ferreira#231	B. abortus bv1	28	82		LNIV-353Ba1-07	Cattle	2007	Alentejo, Portugal
4	5	4	12	2	2	3	3	6	43	8	3	4	3	4	5	2012Ferreira#232	B. abortus bv1	28	82		LNIV-235Ba1-05	Cattle	2005	Alentejo, Portugal
4	5	4	12	2	2	3	3	6	43	8	3	4	3	4	5	2012Ferreira#233	B. abortus bv1	28	82		LNIV-359Ba1-07	Cattle	2007	Alentejo, Portugal
4	5	4	12	2	2	3	3	6	43	8	3	4	3	4	5	2012Ferreira#234	B. abortus bv1	28	82		LNIV-360Ba1-07	Cattle	2007	Alentejo, Portugal
4	5	4	12	2	2	3	3	6	43	8	3	4	3	4	5	2012Ferreira#235	B. abortus bv1	28	82		LNIV-361Ba1-07	Cattle	2007	Alentejo, Portugal
4	5	4	12	2	2	3	3	6	43	8	3	4	3	4	5	2012Ferreira#236	B. abortus bv1	28	82		LNIV-364Ba1-07	Cattle	2007	Alentejo, Portugal
4	5	4	12	2	2	3	3	6	43	8	3	4	3	4	5	2012Ferreira#237	B. abortus bv1	28	82		LNIV-365Ba1-07	Cattle	2007	Alentejo, Portugal
4	5	4	12	2	2	3	3	6	43	8	3	4	3	4	5	2012Ferreira#238	B. abortus bv1	28	82		LNIV-366Ba1-07	Cattle	2007	Alentejo, Portugal
4	5	4	12	2	2	3	3	6	43	8	3	4	3	4	5	2012Ferreira#239	B. abortus bv1	28	82		LNIV-367Ba1-07	Cattle	2007	Alentejo, Portugal
4	5	4	12	2	2	3	3	6	43	8	3	4	3	4	5	2012Ferreira#240	B. abortus bv1	28	82		LNIV-368Ba1-07	Cattle	2007	Alentejo, Portugal
4	5	4	12	2	2	3	3	6	43	8	3	4	3	4	5	2012Ferreira#242	B. abortus bv1	28	82		LNIV-374Ba1-07	Cattle	2007	Alentejo, Portugal
4	5	4	12	2	2	3	3	6	43	8	3	4	3	4	5	2012Ferreira#243	B. abortus bv1	28	82		LNIV-380Ba1-07	Cattle	2007	Alentejo, Portugal
4	5	4	12	2	2	3	3	6	43	8	3	4	3	4	5	2012Ferreira#244	B. abortus bv1	28	82		LNIV-382Ba1-07	Cattle	2007	Alentejo, Portugal
4	5	4	12	2	2	3	3	6	43	8	3	4	3	4	5	2012Ferreira#246	B. abortus bv1	28	82		LNIV-379Ba1-07	Cattle	2007	Alentejo, Portugal
4	5	4	12	2	2	3	3	6	43	8	3	4	3	4	5	2012Ferreira#247	B. abortus bv1	28	82		LNIV-389Ba1-07	Cattle	2007	Alentejo, Portugal
4	5	4	12	2	2	3	3	6	43	8	3	4	3	4	5	2012Ferreira#255	B. abortus bv1	28	82		LNIV-P3Ba1/03	Cattle	2003	Beira Litoral, Portugal
4	5	4	12	2	2	3	3	6	43	8	3	4	3	4	5	2012Ferreira#256	B. abortus bv1	28	82		LNIV-240Ba1-05	Cattle	2005	Ribatejo e Oeste, Portu.
4	5	4	12	2	2	3	3	6	43	8	3	4	3	4	5	2012Ferreira#257	B. abortus bv1	28	82		LNIV-358Ba1-07	Cattle	2007	Ribatejo e Oeste, Portu.
4	5	4	12	2	2	3	3	6	43	8	3	4	3	4	5	2012Ferreira#258	B. abortus bv1	28	82		LNIV-363Ba1-07	Cattle	2007	Ribatejo e Oeste, Portu.
4	5	4	12	2	2	3	3	6	43	8	3	4	3	4	5	2012Ferreira#259	B. abortus bv1	28	82		LNIV-369Ba1-07	Cattle	2007	Ribatejo e Oeste, Portu.
4	5	4	12	2	2	3	3	6	43	8	3	4	3	4	5	2012Ferreira#260	B. abortus bv1	28	82		LNIV-381Ba1-07	Cattle	2007	Ribatejo e Oeste, Portu.
4	5	4	12	2	2	3	3	6	43	8	3	4	3	4	5	2012Ferreira#261	B. abortus bv1	28	82		LNIV-357Ba1-07	Cattle	2007	Ribatejo e Oeste, Portu.
4	5	4	12	2	2	3	3	6	43	8	3	4	3	4	5	2012Ferreira#262	B. abortus bv1	28	82		LNIV-384Ba1-07	Cattle	2007	Ribatejo e Oeste, Portu.
4	5	4	12	2	2	3	3	6	43	8	3	4	3	4	5	2012Ferreira#263	B. abortus bv1	28	82		LNIV-28Ba1-02	Cattle	2002	Ribatejo e Oeste, Portu.
4	5	4	12	2	2	3	3	6	43	8	3	4	3	4	5	2012Ferreira#264	B. abortus bv1	28	82		LNIV-140Ba1-04	Cattle	2004	S. Miguel, Azores, Port.
4	5	4	12	2	2	3	3	6	43	8	3	4	3	4	5	2012Ferreira#265	B. abortus bv1	28	82		LNIV-411Ba1-07	Cattle	2007	S. Miguel, Azores, Port.
4	5	4	12	2	2	3	3	6	43	8	3	4	3	4	5	2012Ferreira#269	B. abortus bv1	28	82		LNIV-417Ba1-07	Cattle	2007	S. Miguel, Azores, Port.
4	5	4	12	2	2	3	3	6	43	8	3	4	3	4	5	2012Ferreira#271	B. abortus bv1	28	82		LNIV-419Ba1-07	Cattle	2007	S. Miguel, Azores, Port.
4	5	4	12	2	2	3	3	6	43	8	3	4	3	4	5	2012Ferreira#272	B. abortus bv1	28	82		LNIV-420Ba1-07	Cattle	2007	S. Miguel, Azores, Port.
4	5	4	12	2	2	3	3	6	43	8	3	4	3	4	5	2012Ferreira#275	B. abortus bv1	28	82		LNIV-424Ba1-07	Cattle	2007	S. Miguel, Azores, Port.
4	5	4	12	2	2	3	3	6	43	8	3	4	3	4	5	2012Ferreira#276	B. abortus bv1	28	82		LNIV-425Ba1-07	Cattle	2007	S. Miguel, Azores, Port.
4	5	4	12	2	2	3	3	6	43	8	3	4	3	4	5	2012Ferreira#279	B. abortus bv1	28	82		LNIV-428Ba1-07	Cattle	2007	S. Miguel, Azores, Port.
4	5	4	12	2	2	3	3	6	43	8	3	4	3	4	5	2012Ferreira#289	B. abortus bv1	28	82		LNIV-438Ba1-07	Cattle	2007	S. Miguel, Azores, Port.
4	5	4	12	2	2	3	3	6	43	8	3	4	3	4	5	2012Ferreira#290	B. abortus bv1	28	82		LNIV-439Ba1-07	Cattle	2007	S. Miguel, Azores, Port.
4	5	4	12	2	2	3	3	6	43	8	3	4	3	4	5	2012Ferreira#293	B. abortus bv1	28	82		LNIV-445Ba1-07	Cattle	2007	S. Miguel, Azores, Port.
4	5	4	12	2	2	3	3	6	43	8	3	4	3	4	5	2013Minharro#004	B. abortus bv1	28	82		30	Cattle	2006	Curvelo, 35790971, Brazil
4	5	4	12	2	2	3	3	6	43	8	3	4	3	4	5	2013Minharro#006	B. abortus bv1	28	82		33	Cattle	2006	Governador Valadares,.
4	5	4	12	2	2	3	3	6	43	8	3	4	3	4	5	2013Minharro#007	B. abortus bv1	28	82		34	Cattle	2006	Governador Valadares,.
4	5	4	12	2	2	3	3	6	43	8	3	4	3	4	5	2013Minharro#012	B. abortus bv1	28	82		39	Cattle	2006	Governador Valadares,.
4	5	4	12	2	2	3	3	6	43	8	3	4	3	4	5	2013Minharro#013	B. abortus bv1	28	82		40	Cattle	2006	Governador Valadares,.
4	5	4	12	2	2	3	3	6	43	8	3	4	3	4	5	2013Minharro#077	B. abortus bv1	28	82		01_jun	Cattle	2006	Porto Alegre, 90570020.
4	5	4	12	2	2	3	3	6	43	8	3	4	3	4	5	2013Minharro#126	B. abortus bv1	28	82		388-01	Cattle		Belo Horizonte, 30001.
4	5	4	12	2	2	3	3	6	43	8	3	4	3	4	5	2013Minharro#127	B. abortus bv1	28	82		147-6	Cattle		Belo Horizonte, 30001.
4	5	4	12	2	2	3	3	6	43	8	3	4	3	4	5	SRR1371317	B. abortus	28	82	1	B06-0410	Cattle		United States - ID
4	5	4	12	2	2	3	3	6	43	8	3	4	3	4	5	SRR1371321	B. abortus	28	82	1	B10-0035	Cattle		United States - ID
4	5	4	12	2	2	3	3	6	43	8	3	4	3	4	5	SRR3096361	B. abortus	28	82		B06-0027	Cattle		USA: Idaho
4	5	4	12	2	2	3	3	6	43	8	3	4	3	4	5	SRR3096362	B. abortus	28	82	1	B06-0028	Cattle		USA: Idaho
4	5	4	12	2	2	3	3	6	43	8	3	4	3	4	5	SRR3096364	B. abortus	28	82	1	B-07-3128	Deer/El.		USA: Wyoming
4	5	4	12	2	2	3	3	6	43	8	3	4	3	4	5	SRR3096365	B. abortus	28	82	1	B08-0056	Cattle		USA: Idaho
4	5	4	12	2	2	3	3	6	43	8	3	4	3	4	5	SRR3096387	B. abortus	28	82	1	B10-0018	Cattle		USA: Idaho
4	5	4	12	2	2	3	3	6	43	8	3	4	3	4	5	SRR3096388	B. abortus	28	82	1	B10-0028	Cattle		USA: Idaho
4	5	4	12	2	2	3	3	6	43	8	3	4	3	4	5	SRR3096391	B. abortus	28	82	1	B10-0173	Cattle		USA: Idaho
4	5	4	12	2	2	3	3	6	43	8	3	4	3	4	5	SRR3096397	B. abortus	28	82	1	B10-0407	Cattle		USA: Idaho
4	5	4	12	2	2	3	3	6	43	8	3	4	3	4	5	SRR3096398	B. abortus	28	82	1	B10-0474	Cattle		USA: Idaho
4	5	4	12	2	2	3	3	6	43	8	3	4	3	4	5	SRR3096405	B. abortus	28	82	1	B10-0654	Cattle		USA: Idaho
4	5	4	12	2	2	3	3	6	43	8	3	4	3	4	5	SRR3096406	B. abortus	28	82	1	B10-0948	Cattle		USA: Idaho
4	5	4	12	2	2	3	3	6	43	8	3	4	3	4	5	SRR3096433	B. abortus	28	82	1	B11-0256	Bison		USA: Wyoming
4	5	4	12	2	2	3	3	6	43	8	3	4	3	4	5	SRR3096440	B. abortus	28	82	1	B11-0268	Deer/El.		USA: Wyoming
4	5	4	12	2	2	3	3	6	43	8	3	4	3	4	5	SRR3096444	B. abortus	28	82	1	B11-0277	Deer/El.		USA: Wyoming
4	5	4	12	2	2	3	3	6	43	8	3	4	3	4	5	SRR3096458	B. abortus	28	82	1	B12-0359	Cattle		USA: Idaho
4	5	4	12	2	2	3	3	6	43	8	3	4	3	4	5	SRR3096459	B. abortus	28	82	1	B12-0360	Cattle		USA: Idaho
4	5	4	12	2	2	3	3	6	43	8	3	4	3	4	5	SRR3096461	B. abortus	28	82	1	B12-0389	Cattle		USA: Idaho
4	5	4	12	2	2	3	3	6	43	8	3	4	3	4	5	SRR3096462	B. abortus	28	82	1	B12-0390	Cattle		USA: Idaho
4	5	4	12	2	2	3	3	6	43	8	3	4	3	4	5	SRR3096465	B. abortus	28	82		B12-0399	Deer/El.		USA: Wyoming
4	5	4	12	2	2	3	3	6	43	8	3	4	3	4	5	SRR3096476	B. abortus	28	82	1	B12-0411	Deer/El.		USA: Wyoming
4	5	4	12	2	2	3	3	6	43	8	3	4	3	4	5	SRR3096486	B. abortus	28	82	1	B12-0570	Cattle		USA: Idaho
4	5	4	12	2	2	3	3	6	43	8	3	4	3	4	5	SRR3096491	B. abortus	28	82	1	B12-0599	Cattle		USA: Idaho
4	5	4	12	2	2	3	3	6	43	8	3	4	3	4	5	SRR3096502	B. abortus	28	82	1	B13-0118	Cattle		USA: Idaho
4	5	4	12	2	2	3	3	6	43	8	3	4	3	3	5	2006LeFleche#105	B. abortus bv1	28	82		BCCN#95-55	Cattle	1995	Costa Rica
4	5	4	12	2	2	3	3	6	43	8	3	4	3	3	5	2017Hernández-M.	B. abortus	28	82		baboCR31	Cattle	2004	Costa Rica
4	5	4	12	2	2	3	3	6	43	8	3	4	3	3	5	2017Hernández-M.	B. abortus	28	82		baboCR44	Cattle	2004	Costa Rica
4	5	4	12	2	2	3	3	6	43	8	3	4	3	3	5	2017Hernández-M.	B. abortus	28	82		baboCR72	Cattle	2011	Costa Rica
4	5	4	12	2	2	3	3	6	43	8	3	4	3	6	5	2006LeFleche#104	B. abortus bv1	28	82		BCCN#95-51	Cattle	1995	Argentina
4	5	4	12	2	2	3	3	6	43	8	3	4	3	6	5	2012Ferreira#267	B. abortus bv1	28	82		LNIV-413Ba1-07	Cattle	2007	S. Miguel, Azores, Port.

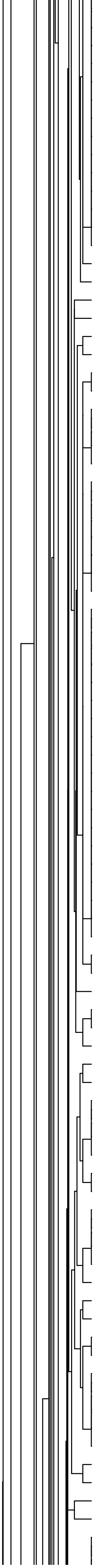

4	5	4	12	2	2	3	3	6	43	8	3	4	3	6	5	2012Ferreira#274	B. abortus bv1	28	82		LNIV-423Ba1-07	Cattle	2007	S. Miguel, Azores, Port.
4	5	4	12	2	2	3	3	6	43	8	3	4	3	6	5	2012Ferreira#277	B. abortus bv1	28	82		LNIV-426Ba1-07	Cattle	2007	S. Miguel, Azores, Port.
4	5	4	12	2	2	3	3	6	43	8	3	4	3	6	5	2012Ferreira#284	B. abortus bv1	28	82		LNIV-434Ba1-07	Cattle	2007	S. Miguel, Azores, Port.
4	5	4	12	2	2	3	3	6	43	8	3	4	3	6	5	2012Ferreira#287	B. abortus bv1	28	82		LNIV-436Ba1-07	Cattle	2007	S. Miguel, Azores, Port.
4	5	4	12	2	2	3	3	6	43	8	3	4	3	6	5	2012Ferreira#288	B. abortus bv1	28	82		LNIV-437Ba1-07	Cattle	2007	S. Miguel, Azores, Port.
4	5	4	12	2	2	3	3	6	43	8	3	4	3	6	5	2013Minharro#109	B. abortus bv1	28	82		SP 4	Cattle		Sao Paulo, 01036970, .
4	5	4	12	2	2	3	3	6	43	8	3	4	3	6	5	SRR3096347	B. abortus	28	82		B04-0060	Cattle		USA: Wyoming
4	5	4	12	2	2	3	3	6	43	8	3	4	3	6	5	SRR3096348	B. abortus	28	82		B04-0067	Cattle		USA: Wyoming
4	5	4	12	2	2	3	3	6	43	8	3	4	3	6	5	SRR3096349	B. abortus	28	82		B04-0083	Cattle		USA: Wyoming
4	5	4	12	2	2	3	3	6	43	8	3	4	3	6	5	SRR3096358	B. abortus	28	82	1	B05-0908	Deer/El.		USA: Montana
4	5	4	12	2	2	3	3	6	43	8	3	4	3	6	5	SRR3096393	B. abortus	28	82		B-10021001	Deer/El.		USA: Wyoming
4	5	4	12	2	2	3	3	6	43	8	3	4	3	6	5	SRR3096400	B. abortus	28	82	1	B10-0517	Deer/El.		USA: Montana
4	5	4	12	2	2	3	3	6	43	8	3	4	3	6	5	SRR3096426	B. abortus	28	82	1	B11-0249	Bison		USA: Wyoming
4	5	4	12	2	2	3	3	6	43	8	3	4	3	6	5	SRR3096445	B. abortus	28	82	1	B-11031001	Deer/El.		USA: Wyoming
4	5	4	12	2	2	3	3	6	43	8	3	4	3	10	5	SRR3096434	B. abortus	28	82	1	B11-0257	Bison		USA: Wyoming
4	5	4	12	2	2	3	3	6	43	8	3	4	9	4	5	2013Minharro#078	B. abortus bv1	28	82		02_jun	Cattle	2006	Porto Alegre, 90570020.
4	5	4	12	2	2	3	3	6	43	8	5	6	3	3	5	2013Minharro#029	B. abortus bv1	28	82		96	Cattle	2007	Guarai, 77700000, Brazil
4	5	4	12	2	2	3	3	6	45	8	3	6	3	3	5	2013Minharro#034	B. abortus bv1	28	75		147	Cattle	2007	Esmeraldas, 35740000,.
4	5	4	12	2	2	3	3	6	43	8	3	6	3	3	6	SRR017413	B. abortus	28	82	5	S19 (NCTC 80.	Cattle		
4	5	4	12	2	2	3	3	6	43	8	3	6	3	6	6	SRR3096360	B. abortus	28	82	1	B05-1282	Bison		USA: Montana
4	5	4	12	2	2	3	3	6	43	8	3	6	3	8	5	SRR3096332	B. abortus	28	82	1	B02-0137	Bison		USA: Montana
4	5	4	12	2	2	3	3	6	43	8	3	6	3	8	5	SRR3096501	B. abortus	28	82	1	B13-0101	Bison		USA: Montana
4	5	4	12	2	2	3	3	6	43	8	3	6	3	3	5	2006LeFleche#135	B. abortus bv1	28	82		BfR 95	Mouse		
4	5	4	12	2	2	3	3	6	43	8	3	6	3	3	5	2009Her#011	B. abortus bv1	28	82		KRef15	Cattle		USA
4	5	4	12	2	2	3	3	6	43	8	3	6	3	3	5	2013Minharro#031	B. abortus bv1	28	82		137	Cattle	2007	Esmeraldas, 35740000,.
4	5	4	12	2	2	3	3	6	43	8	3	6	3	3	5	2016Shevtsova#028	B. abortus	28	82		68Al	Cattle	1968	Karachingil, Zhambyl, .
4	5	4	12	2	2	3	3	6	43	8	3	6	3	7	5	SRR1371361	B. abortus	28	82		B13-0215	Cattle		United States - MT
4	5	4	12	2	2	3	3	6	43	8	3	6	3	7	5	SRR3096328	B. abortus	28	82	1	B01-0314	Bison		USA: Wyoming
4	5	4	12	2	2	3	3	6	43	8	3	6	3	7	5	SRR3096368	B. abortus	28	82	1	B08-0404	Bison		USA: Montana
4	5	4	12	2	2	3	3	6	43	8	3	6	3	7	5	SRR3096377	B. abortus	28	82	1	B08-1080	Bison		USA: Montana
4	5	4	12	2	2	3	3	6	43	8	3	6	3	7	5	SRR3096386	B. abortus	28	82	1	B09-0553	Bison		USA: Montana
4	5	4	12	2	2	3	3	6	43	8	3	6	3	7	5	SRR3096390	B. abortus	28	82	1	B10-0118	Deer/El.		USA: Montana
4	5	4	12	2	2	3	3	6	43	8	3	6	3	7	5	SRR3096401	B. abortus	28	82	1	B10-0529	Deer/El.		USA: Montana
4	5	4	12	2	2	3	3	6	43	8	3	6	3	6	5	SRR1371318	B. abortus	28	82		B07-0266	Cattle		United States - MT
4	5	4	12	2	2	3	3	6	43	8	3	6	3	6	5	SRR1371319	B. abortus	28	82	1	B08-0416	Cattle		United States - MT
4	5	4	12	2	2	3	3	6	43	8	3	6	3	6	5	SRR1371331	B. abortus	28	82	1	B11-0604	Cattle		United States - MT
4	5	4	12	2	2	3	3	6	43	8	3	6	3	6	5	SRR3096323	B. abortus	28	82	1	B01-0154	Bison		USA: Wyoming
4	5	4	12	2	2	3	3	6	43	8	3	6	3	6	5	SRR3096324	B. abortus	28	82	1	B01-0174	Bison		USA: Wyoming
4	5	4	12	2	2	3	3	6	43	8	3	6	3	6	5	SRR3096344	B. abortus	28	82	1	B03-0136	Bison		USA: Montana
4	5	4	12	2	2	3	3	6	43	8	3	6	3	6	5	SRR3096359	B. abortus	28	82	1	B05-0999	Bison		USA: Wyoming
4	5	4	12	2	2	3	3	6	43	8	3	6	3	6	5	SRR3096366	B. abortus	28	82	1	B08-0151	Deer/El.		USA: Montana
4	5	4	12	2	2	3	3	6	43	8	3	6	3	6	5	SRR3096373	B. abortus	28	82	1	B08-0795	Bison		USA: Montana
4	5	4	12	2	2	3	3	6	43	8	3	6	3	6	5	SRR3096374	B. abortus	28	82	1	B08-0921	Bison		USA: Montana
4	5	4	12	2	2	3	3	6	43	8	3	6	3	6	5	SRR3096375	B. abortus	28	82	1	B08-0951	Bison		USA: Montana
4	5	4	12	2	2	3	3	6	43	8	3	6	3	6	5	SRR3096376	B. abortus	28	82	1	B08-1019	Bison		USA: Montana
4	5	4	12	2	2	3	3	6	43	8	3	6	3	6	5	SRR3096395	B. abortus	28	82	1	B10-0273	Deer/El.		USA: Montana
4	5	4	12	2	2	3	3	6	43	8	3	6	3	6	5	SRR3096408	B. abortus	28	82	1	B10-0975	Bison		USA: Montana
4	5	4	12	2	2	3	3	6	43	8	3	6	3	6	5	SRR3096449	B. abortus	28	82	1	B11-0480	Bison		USA: Montana
4	5	4	12	2	2	3	3	6	43	8	3	6	3	6	5	SRR3096452	B. abortus	28	82	1	B11-0611	Cattle		USA: Montana
4	5	4	12	2	2	3	3	6	43	8	3	6	3	6	5	SRR3096453	B. abortus	28	82	1	B11-0612	Cattle		USA: Montana
4	5	4	12	2	2	3	3	6	43	8	3	6	3	6	5	SRR3096454	B. abortus	28	82	1	B11-0613	Cattle		USA: Montana
4	5	4	12	2	2	3	3	6	43	8	3	6	3	6	5	SRR3096455	B. abortus	28	82	1	B11-0616	Cattle		USA: Montana
4	5	4	12	2	2	3	3	6	43	8	3	6	3	4	5	2012Ferreira#295	B. abortus bv1	28	82		LNIV-P15Ba1/03	Cattle	2003	Tras-os-Montes, Portugal
4	5	4	12	2	2	3	3	6	43	8	3	6	3	4	5	SRR1371373	B. abortus	28	82	1	B94-0885	Cattle		United States - MS
4	5	4	12	2	2	3	3	6	43	8	3	6	3	7	7	SRR1371387	B. abortus	28	82	1	B99-0096	Cattle		United States - TX
4	5	4	12	2	2	3	3	6	43	8	3	7	3	8	5	SRR3096326	B. abortus	28	82	1	B01-0305	Bison		USA: Wyoming
4	5	4	12	2	2	3	3	6	43	8	3	7	3	8	5	SRR3096327	B. abortus	28	82	1	B01-0308	Bison		USA: Wyoming
4	5	4	12	2	2	3	3	6	43	8	3	7	3	6	5	SRR3096369	B. abortus	28	82	1	B08-0423	Bison		USA: Montana
4	5	4	12	2	2	3	3	6	43	8	4	4	3	4	6	2013Minharro#085	B. abortus bv1	28	82		13b/02	Cattle	2002	Porto Alegre, 90570020.
4	5	4	12	2	2	3	3	6	43	8	4	4	3	4	7	2013Minharro#095	B. abortus bv1	28	82		Ba 56	Cattle	1996	Porto Alegre, 90570020.
4	5	4	12	2	2	3	3	6	43	8	4	4	3	4	5	2013Minharro#011	B. abortus bv2	28	82		38	Cattle	2006	Governador Valadares,.
4	5	4	12	2	2	3	3	6	43	8	4	4	3	4	5	2016Shevtsova#022	B. abortus	28	82		57al2	Cattle	1957	Eskeldi, Almaty, Kazakh.
4	5	4	12	2	2	3	3	6	43	8	4	4	3	4	5	2016Shevtsova#024	B. abortus	28	82		85Ak	Cattle	1985	Razdol'nyy, Esil, Akmola.
4	5	4	12	2	2	3	3	6	43	8	4	4	3	4	5	2016Shevtsova#035	B. abortus	28	82		43Al	Cattle	1943	Almaty, Kazakhstan
4	5	4	12	2	2	3	3	6	45	8	4	4	3	4	5	2013Minharro#008	B. abortus bv2	28	75		35	Cattle	2006	Governador Valadares,.
4	5	4	12	2	2	3	3	6	45	8	4	4	3	4	5	2013Minharro#009	B. abortus bv2	28	75		36	Cattle	2006	Governador Valadares,.
4	5	4	12	2	2	3	3	6	43	8	5	4	3	4	5	2013Jiang#103	B. abortus bv1	28	82		NM1058	Cattle	1984	Inner Mongolia, China
4	5	4	12	2	2	3	3	6	43	8	5	4	3	4	5	2013Jiang#114	B. abortus bv1	28	82		NM1076	Ovine	1985	Inner Mongolia, China
4	5	4	12	2	2	3	3	6	43	8	5	4	3	4	5	2013Jiang#115	B. abortus bv1	28	82		NM1078	Ovine	1985	Inner Mongolia, China
4	5	4	12	2	2	3	3	6	43	8	5	4	3	4	5	2013Jiang#116	B. abortus bv1	28	82		NM1079	Ovine	1985	Inner Mongolia, China
4	5	4	12	2	2	3	1	6	43	8	5	4	3	4	5	2013Jiang#102	B. abortus bv1	37	77		NM1057	Cattle	1984	Inner Mongolia, China
4	5	4	12	2	2	3	3	6	43	8	4	7	3	4	5	2006LeFleche#116	B. abortus bv4	28	82		BCCN#95-31	Cattle	1995	Sicilia, Italia
4	5	4	12	2	2	3	3	6	43	8	4	6	3	4	5	SRR3096460	B. abortus	28	82	1	B12-0385	Deer/El.		USA: Montana
4	5	4	12	2	2	3	3	6	43	9	4	5	3	4	5	2013Minharro#092	B. abortus bv1	28	235		89/04	Cattle	2004	Porto Alegre, 90570020.
4	5	4	12	2	2	3	3	6	43	9	4	5	3	4	5	2013Minharro#134	B. abortus bv1	28	235		Jr 20	Cattle	2008	Sao Paulo, 01036970, .
4	5	4	12	2	2	3	3	6	43	8	4	5	3	4	5	2013Minharro#091	B. abortus bv1	28	82		80/04	Cattle	2004	Porto Alegre, 90570020.
4	5	4	12	2	2	3	3	6	43	8	4	5	3	4	5	2013Minharro#099	B. abortus bv1	28	82		RS 4	Cattle		Porto Alegre, 90570020.
4	5	4	12	2	2	3	3	6	43	8	4	5	3	4	5	SRR3096392	B. abortus	28	82	1	B10-0203	Deer/El.		USA: Montana
4	5	4	12	2	2	3	3	6	43	8	4	5	3	4	5	SRR3096396	B. abortus	28	82	1	B10-0325	Deer/El.		USA: Montana
4	5	4	12	2	2	3	3	6	43	8	4	5	3	4	5	SRR3096463	B. abortus	28	82	1	B12-0393	Deer/El.		USA: Montana
3	5	4	12	2	2	3	3	6	43	8	3	4	3	4	5	2012Ferreira#145	B. abortus bv1	33	76		LNIV-444Ba1-07	Cattle	2007	S. Miguel, Azores, Port.
3	5	4	12	2	2	3	3	6	43	8	4	4	3	4	5	GCA_001296965	B. abortus	33	76		104M			
4	5	4	12	2	2	3	3	6	43	8	3	3	3	9	7	SRR1371382	B. abortus	28	82	1	B98-0370	Cattle		United States - TX
4	5	4	12	2	2	3	3	6	43	8	3	8	3	9	5	SRR3096367	B. abortus	28	82	1	B08-0187	Deer/El.		USA: Montana
4	5	4	12	2	2	3	2	6	43	8	3	4	3	3	5	2006LeFleche#005	B. abortus bv4	30	78		REF 292	Cattle		England
4	5	4	12	2	2	3	2	6	43	8	3	4	3	3	5	2009Her#004	B. abortus bv4	30	78		KRef04	Cattle		England

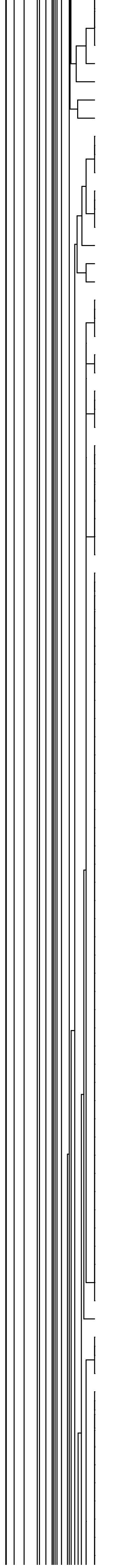

4	5	4	12	2	2	3	2	6	43	8	3	4	3	3	5	2012Ferreira#213	B. abortus bv4	30	78		REF 292			
4	5	4	12	2	2	3	2	6	43	8	3	4	3	3	5	GCA_000740135	B. abortus	30	78	1	BDW			
4	5	4	12	2	2	3	2	6	43	8	3	4	3	3	5	SRR1371297	B. abortus	30	78	1	292			
4	5	4	12	2	2	3	2	6	43	8	3	5	3	3	5	GCA_000018725	B. abortus	30	78	5	S19 (NCTC 80.	Cattle	2008	USA
4	5	4	12	2	2	3	1	6	43	8	3	4	3	3	6	2006LeFleche#137	B. abortus bv1	37	77		BfR 97	Cattle		Zimbabwe
4	5	4	12	2	2	3	3	7	43	8	3	4	5	4	5	2013Minharro#005	B. abortus bv1	28	227		32	Cattle	2006	Governador Valadares,.
4	5	4	12	2	2	3	3	7	43	8	3	4	3	7	5	SRR1371376	B. abortus	28	227	1	B95-0236	Cattle		United States - MS
4	5	4	12	2	2	3	3	6	43	8	3	5	3	3	6	2017Hernández-M.	B. abortus	28	82		baboCR141	Cattle	2016	Costa Rica
4	5	4	12	2	2	3	3	6	43	8	3	5	3	3	6	2017Hernández-M.	B. abortus	28	82		baboCR142	Cattle	2016	Costa Rica
4	5	4	12	2	2	3	3	6	43	8	3	5	3	3	6	2017Hernández-M.	B. abortus	28	82		baboCR166	Cattle	2015	Costa Rica
4	5	4	12	2	2	3	3	6	43	8	6	5	3	3	6	2017Hernández-M.	B. abortus	28	82		baboCR140	Cattle	2016	Costa Rica
4	5	4	12	2	2	3	3	6	43	8	6	5	3	3	6	2017Hernández-M.	B. abortus	28	82		baboCR160	Cattle	2016	Costa Rica
4	5	4	12	2	2	3	3	6	43	8	6	5	3	3	6	2017Hernández-M.	B. abortus	28	82		baboCR165	Cattle	2016	Costa Rica
4	5	4	12	2	2	3	3	6	45	8	3	5	3	3	6	2013Minharro#033	B. abortus bv1	28	75		144	Cattle	2007	Esmeraldas, 35740000,.
4	5	4	12	2	2	3	3	6	43	8	3	5	3	6	6	SRR1371324	B. abortus	28	82		B11-0037	Cattle		United States - WY
4	5	4	12	2	2	3	3	6	43	8	3	5	3	6	11	SRR3096403	B. abortus	28	82	1	B10-0584	Deer/El.		USA: Montana
4	5	4	12	2	2	3	3	6	43	8	7	5	3	3	5	2017Hernández-M.	B. abortus	28	82		baboCR117	Cattle	2015	Costa Rica
4	5	4	12	2	2	3	3	6	43	8	7	5	3	3	5	2017Hernández-M.	B. abortus	28	82		baboCR161	Cattle	2016	Costa Rica
4	5	4	12	2	2	3	3	6	43	8	7	5	3	3	5	2017Hernández-M.	B. abortus	28	82		baboCR107	Cattle	2015	Costa Rica
4	5	4	12	2	2	3	3	6	43	8	8	5	3	3	5	2017Hernández-M.	B. abortus	28	82		baboCR113	Cattle	2015	Costa Rica
4	5	4	12	2	2	3	3	6	43	8	8	5	3	3	5	2017Hernández-M.	B. abortus	28	82		baboCR125	Cattle	2015	Costa Rica
4	5	4	12	2	2	3	3	6	43	8	5	5	3	3	5	2017Hernández-M.	B. abortus	28	82		baboCR112	Cattle	2015	Costa Rica
4	5	4	12	2	2	3	3	6	43	8	5	5	3	3	5	2017Hernández-M.	B. abortus	28	82		baboCR106	Cattle	2015	Costa Rica
4	5	4	12	2	2	3	3	6	43	8	5	5	3	3	5	2017Hernández-M.	B. abortus	28	82		baboCR108	Cattle	2015	Costa Rica
4	5	4	12	2	2	3	3	6	43	8	4	5	3	3	5	2013Minharro#030	B. abortus bv1	28	82		136	Cattle	2007	Esmeraldas, 35740000,.
4	5	4	12	2	2	3	3	6	43	8	4	5	3	3	5	2017Hernández-M.	B. abortus	28	82		baboCR48	Cattle	2005	Costa Rica
4	5	4	12	2	2	3	3	6	43	8	4	5	3	3	5	2017Hernández-M.	B. abortus	28	82		baboCR49	Cattle	2005	Costa Rica
4	5	4	12	2	2	3	3	6	43	8	4	5	3	3	5	2017Hernández-M.	B. abortus	28	82		baboCR50	Cattle	2005	Costa Rica
4	5	4	12	2	2	3	3	6	43	8	4	5	3	3	5	2017Hernández-M.	B. abortus	28	82		baboCR51	Cattle	2005	Costa Rica
4	5	4	12	2	2	3	3	6	43	8	4	5	3	3	5	2017Hernández-M.	B. abortus	28	82		baboCR24	Cattle	2003	Costa Rica
4	5	4	12	2	2	3	3	6	43	8	4	5	3	3	5	2017Hernández-M.	B. abortus	28	82		baboCR68	Cattle	2003	Costa Rica
4	5	4	12	2	2	3	3	6	43	8	3	5	3	3	5	2006LeFleche#107	B. abortus bv1	28	82		BCCN#V1	Cattle		USA
4	5	4	12	2	2	3	3	6	43	8	3	5	3	3	5	2009Her#010	B. abortus bv1	28	82		KRef13	Cattle		USA
4	5	4	12	2	2	3	3	6	43	8	3	5	3	3	5	2012Ferreira#223	B. abortus bv1	28	82		LNIV-18Ba1-01	Cattle	2001	Azores, Portugal
4	5	4	12	2	2	3	3	6	43	8	3	5	3	3	5	2012Ferreira#224	B. abortus bv1	28	82		LNIV-20Ba1-01	Cattle	2001	Azores, Portugal
4	5	4	12	2	2	3	3	6	43	8	3	5	3	3	5	2012Ferreira#297	B. abortus bv1	28	82		S19	Cattle		USA
4	5	4	12	2	2	3	3	6	43	8	3	5	3	3	5	2016Shevtsova#091	B. abortus	28	82		S19	vaccine		
4	5	4	12	2	2	3	3	6	43	8	3	5	3	3	5	2017Hernández-M.	B. abortus	28	82		baboCR40	Cattle	2004	Costa Rica
4	5	4	12	2	2	3	3	6	43	8	3	5	3	3	5	2017Hernández-M.	B. abortus	28	82		baboCR41	Cattle	2004	Costa Rica
4	5	4	12	2	2	3	3	6	43	8	3	5	3	3	5	2017Hernández-M.	B. abortus	28	82		baboCR42	Cattle	2004	Costa Rica
4	5	4	12	2	2	3	3	6	43	8	3	5	3	3	5	2017Hernández-M.	B. abortus	28	82		baboCR43	Cattle	2004	Costa Rica
4	5	4	12	2	2	3	3	6	43	8	3	5	3	3	5	2017Hernández-M.	B. abortus	28	82		baboCR45	Cattle	2004	Costa Rica
4	5	4	12	2	2	3	3	6	43	8	3	5	3	3	5	2017Hernández-M.	B. abortus	28	82		baboCR46	Cattle	2004	Costa Rica
4	5	4	12	2	2	3	3	6	43	8	3	5	3	3	5	2017Hernández-M.	B. abortus	28	82		baboCR47	Cattle	2005	Costa Rica
4	5	4	12	2	2	3	3	6	43	8	3	5	3	3	5	2017Hernández-M.	B. abortus	28	82		baboCR32	Cattle	2004	Costa Rica
4	5	4	12	2	2	3	3	6	43	8	3	5	3	3	5	2017Hernández-M.	B. abortus	28	82		baboCR53	Cattle	2005	Costa Rica
4	5	4	12	2	2	3	3	6	43	8	3	5	3	3	5	2017Hernández-M.	B. abortus	28	82		baboCR54	Cattle	2005	Costa Rica
4	5	4	12	2	2	3	3	6	43	8	3	5	3	3	5	2017Hernández-M.	B. abortus	28	82		baboCR55	Cattle	2005	Costa Rica
4	5	4	12	2	2	3	3	6	43	8	3	5	3	3	5	2017Hernández-M.	B. abortus	28	82		baboCR56	Cattle	2005	Costa Rica
4	5	4	12	2	2	3	3	6	43	8	3	5	3	3	5	2017Hernández-M.	B. abortus	28	82		baboCR33	Cattle	2004	Costa Rica
4	5	4	12	2	2	3	3	6	43	8	3	5	3	3	5	2017Hernández-M.	B. abortus	28	82		baboCR26	Cattle	2003	Costa Rica
4	5	4	12	2	2	3	3	6	43	8	3	5	3	3	5	2017Hernández-M.	B. abortus	28	82		baboCR34	Cattle	2004	Costa Rica
4	5	4	12	2	2	3	3	6	43	8	3	5	3	3	5	2017Hernández-M.	B. abortus	28	82		baboCR35	Cattle	2004	Costa Rica
4	5	4	12	2	2	3	3	6	43	8	3	5	3	3	5	2017Hernández-M.	B. abortus	28	82		baboCR36	Cattle	2004	Costa Rica
4	5	4	12	2	2	3	3	6	43	8	3	5	3	3	5	2017Hernández-M.	B. abortus	28	82		baboCR37	Cattle	2004	Costa Rica
4	5	4	12	2	2	3	3	6	43	8	3	5	3	3	5	2017Hernández-M.	B. abortus	28	82		baboCR38	Cattle	2004	Costa Rica
4	5	4	12	2	2	3	3	6	43	8	3	5	3	3	5	2017Hernández-M.	B. abortus	28	82		baboCR39	Cattle	2004	Costa Rica
4	5	4	12	2	2	3	3	6	43	8	3	5	3	3	5	2017Hernández-M.	B. abortus	28	82		baboCR115	Cattle	2015	Costa Rica
4	5	4	12	2	2	3	3	6	43	8	3	5	3	3	5	2017Hernández-M.	B. abortus	28	82		baboCR124	Cattle	2015	Costa Rica
4	5	4	12	2	2	3	3	6	43	8	3	5	3	3	5	2017Hernández-M.	B. abortus	28	82		baboCR127	Cattle	2016	Costa Rica
4	5	4	12	2	2	3	3	6	43	8	3	5	3	3	5	2017Hernández-M.	B. abortus	28	82		baboCR128	Cattle	2016	Costa Rica
4	5	4	12	2	2	3	3	6	43	8	3	5	3	3	5	2017Hernández-M.	B. abortus	28	82		baboCR129	Cattle	2016	Costa Rica
4	5	4	12	2	2	3	3	6	43	8	3	5	3	3	5	2017Hernández-M.	B. abortus	28	82		baboCR130	Cattle	2016	Costa Rica
4	5	4	12	2	2	3	3	6	43	8	3	5	3	3	5	2017Hernández-M.	B. abortus	28	82		baboCR131	Cattle	2016	Costa Rica
4	5	4	12	2	2	3	3	6	43	8	3	5	3	3	5	2017Hernández-M.	B. abortus	28	82		baboCR132	Cattle	2016	Costa Rica
4	5	4	12	2	2	3	3	6	43	8	3	5	3	3	5	2017Hernández-M.	B. abortus	28	82		baboCR134	Cattle	2016	Costa Rica
4	5	4	12	2	2	3	3	6	43	8	3	5	3	3	5	2017Hernández-M.	B. abortus	28	82		baboCR135	Cattle	2016	Costa Rica
4	5	4	12	2	2	3	3	6	43	8	3	5	3	3	5	2017Hernández-M.	B. abortus	28	82		baboCR116	Cattle	2015	Costa Rica
4	5	4	12	2	2	3	3	6	43	8	3	5	3	3	5	2017Hernández-M.	B. abortus	28	82		baboCR136	Cattle	2016	Costa Rica
4	5	4	12	2	2	3	3	6	43	8	3	5	3	3	5	2018Vergnaud#0218	B. abortus bv1	28	82		BCCN#V1B	Cattle		USA
4	5	4	12	2	2	3	3	6	43	8	3	5	3	3	5	SRR1371299	B. abortus	28	82	5	S19			
4	5	4	12	2	2	3	3	6	43	8	3	5	3	3	5	SRR1371379	B. abortus	28	82	5	19	Cattle		United States - TX
3	5	4	12	2	2	3	3	6	43	8	3	5	3	3	5	2013Minharro#098	B. abortus bv1	33	76		RS 3	Cattle	2004	Porto Alegre, 90570020.
4	5	4	12	2	2	3	3	6	43	8	3	5	3	6	5	2013Minharro#107	B. abortus bv1	28	82		SP 2	Cattle		Sao Paulo, 01036970, .
4	5	4	12	2	2	3	3	6	43	8	3	5	3	6	5	SRR3096333	B. abortus	28	82	1	B02-0186	Bison		USA: Montana
4	5	4	12	2	2	3	3	6	43	8	3	5	3	6	5	SRR3096483	B. abortus	28	82	1	B12-0418	Deer/El.		USA: Wyoming
4	5	4	12	2	2	3	3	6	43	8	3	5	3	4	5	2012Ferreira#268	B. abortus bv1	28	82		LNIV-416Ba1-07	Cattle	2007	S. Miguel, Azores, Port.
4	5	4	12	2	2	3	3	6	43	8	3	5	3	4	5	2012Ferreira#273	B. abortus bv1	28	82		LNIV-422Ba1-07	Cattle	2007	S. Miguel, Azores, Port.
4	5	4	12	2	2	3	3	6	43	8	3	5	3	4	5	2012Ferreira#278	B. abortus bv1	28	82		LNIV-427Ba1-07	Cattle	2007	S. Miguel, Azores, Port.
4	5	4	12	2	2	3	3	6	43	8	3	5	3	4	5	2012Ferreira#282	B. abortus bv1	28	82		LNIV-432Ba1-07	Cattle	2007	S. Miguel, Azores, Port.
4	5	4	12	2	2	3	3	6	43	8	3	5	3	4	5	2012Ferreira#294	B. abortus bv1	28	82		LNIV-446Ba1-07	Cattle	2007	S. Miguel, Azores, Port.
4	5	4	12	2	2	3	3	6	43	8	3	5	3	4	5	2013Minharro#079	B. abortus bv1	28	82		03_jun	Cattle	2006	Porto Alegre, 90570020.
4	5	4	12	2	2	3	3	6	43	8	3	5	3	4	5	2013Minharro#080	B. abortus bv1	28	82		07_jun	Cattle	2006	Porto Alegre, 90570020.
4	5	4	12	2	2	3	3	6	43	8	3	5	3	4	5	2013Minharro#086	B. abortus bv1	28	82		14_mar	Cattle	2003	Porto Alegre, 90570020.
4	5	4	12	2	2	3	3	6	43	8	3	5	3	4	5	SRR1371368	B. abortus	28	82	1	B93-0623	Cattle		United States - MS
4	5	4	12	2	2	3	3	6	43	8	3	5	3	4	5	SRR1371378	B. abortus	28	82	1	B96-0217	Cattle		United States - TX

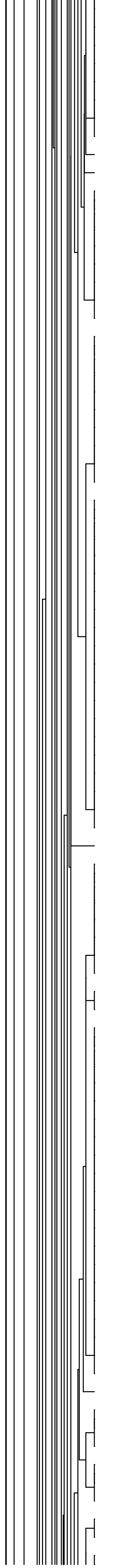

4	5	4	12	2	2	3	3	6	43	8	3	5	3	4	5	SRR3096353	B. abortus	28	82	1	B05-0014	Deer/El.		USA: Wyoming
4	5	4	12	2	2	3	3	6	43	8	3	5	3	4	5	SRR3096370	B. abortus	28	82	1	B08-0440	Bison		USA: Montana
4	5	4	12	2	2	3	3	6	43	8	3	5	3	4	5	SRR3096413	B. abortus	28	82	1	B11-0142	Deer/El.		USA: Wyoming
4	5	4	12	2	2	3	3	6	43	8	3	5	3	4	5	SRR3096415	B. abortus	28	82	1	B11-0144	Deer/El.		USA: Wyoming
4	5	4	12	2	2	3	3	6	43	8	3	5	3	4	5	SRR3096438	B. abortus	28	82	1	B11-0261	Bison		USA: Wyoming
4	5	4	12	2	2	3	3	6	43	8	3	5	3	4	5	SRR3096477	B. abortus	28	82	1	B12-0412	Deer/El.		USA: Wyoming
4	5	4	12	2	2	3	3	6	43	8	3	5	3	4	5	SRR3096479	B. abortus	28	82	1	B12-0414	Deer/El.		USA: Wyoming
4	5	4	12	2	2	3	3	6	43	8	3	5	3	4	5	SRR3096480	B. abortus	28	82	1	B12-0415	Deer/El.		USA: Wyoming
4	5	4	12	2	2	3	3	6	43	8	3	5	3	7	5	SRR3096446	B. abortus	28	82	1	B11-0442	Bison		USA: Montana
4	5	4	12	2	2	3	3	6	43	8	3	5	2	4	5	SRR3096474	B. abortus	28	82		B12-0409	Deer/El.		USA: Wyoming
4	5	4	12	2	2	3	3	5	43	8	3	5	3	4	5	2006LeFleche#002	B. abortus bv1	28	83		REF 544	Cattle		England
4	5	4	12	2	2	3	3	5	43	8	3	5	3	4	5	2009Her#001	B. abortus bv1	28	83		KRef01	Cattle		England
4	5	4	12	2	2	3	3	5	43	8	3	5	3	4	5	2012Ferreira#214	B. abortus bv1	28	83		REF 544	Cattle		
4	5	4	12	2	2	3	3	5	43	8	3	5	3	4	5	2013Minharro#100	B. abortus bv1	28	83		RS 5	Cattle	2004	Porto Alegre, 90570020.
4	5	4	12	2	2	3	3	5	43	8	3	5	3	4	5	2016Shevtsova#093	B. abortus	28	83		REF 544	Cattle		
4	5	4	12	2	2	3	3	5	43	8	3	5	3	4	5	2018Vergnaud#0216	B. abortus bv1	28	83		BCCN#R4C	Cattle		United Kingdom
4	5	4	12	2	2	3	3	5	43	8	3	5	3	4	5	2018Vergnaud#0217	B. abortus bv1	28	83		BCCN#R4D	Cattle		United Kingdom
4	5	4	12	2	2	3	3	5	43	8	3	5	3	4	5	SRR1371296	B. abortus	28	83	1	544			
4	5	3	12	2	3	3	3	6	43	8	3	5	3	3	5	2017Hernández-M.	B. abortus	191	383		baboCR105	Cattle	2015	Costa Rica
4	5	3	12	2	3	3	3	6	43	8	3	5	3	3	5	2017Hernández-M.	B. abortus	191	383		baboCR163	Cattle	2016	Costa Rica
4	5	3	12	2	3	3	3	6	43	8	3	5	3	3	5	2017Hernández-M.	B. abortus	191	383		baboCR84	Cattle	2014	Costa Rica
4	5	3	12	2	3	3	3	6	43	8	3	5	3	3	5	2017Hernández-M.	B. abortus	191	383		baboCR94	Cattle	2015	Costa Rica
4	5	3	12	2	3	3	3	6	43	8	3	5	3	3	5	2017Hernández-M.	B. abortus	191	383		baboCR101	Cattle	2015	Costa Rica
4	5	3	12	2	3	3	3	6	43	8	3	5	3	3	5	2017Hernández-M.	B. abortus	191	383		baboCR103	Cattle	2015	Costa Rica
4	5	3	12	2	3	3	3	6	43	8	3	5	3	3	5	2017Hernández-M.	B. abortus	191	383		baboCR154	Cattle	2016	Costa Rica
4	5	3	12	2	3	3	3	6	43	8	3	5	3	3	5	2017Hernández-M.	B. abortus	191	383		baboCR155	Cattle	2016	Costa Rica
4	5	3	12	2	3	3	3	6	43	8	3	5	3	3	5	2017Hernández-M.	B. abortus	191	383		baboCR156	Cattle	2016	Costa Rica
4	5	3	12	2	2	3	3	6	43	8	3	5	3	3	5	2017Hernández-M.	B. abortus	112	210		baboCR96	Cattle	2015	Costa Rica
4	5	3	12	2	2	3	3	6	43	8	3	5	3	3	5	2017Hernández-M.	B. abortus	112	210		baboCR100	Cattle	2015	Costa Rica
4	5	3	12	2	2	3	3	6	43	8	3	5	3	3	5	2017Hernández-M.	B. abortus	112	210		baboCR118	Cattle	2015	Costa Rica
4	5	3	12	2	2	3	3	6	43	8	3	5	3	3	5	2017Hernández-M.	B. abortus	112	210		baboCR119	Cattle	2015	Costa Rica
4	5	3	12	2	2	3	3	6	43	8	3	5	3	3	5	2017Hernández-M.	B. abortus	112	210		baboCR121	Cattle	2015	Costa Rica
4	5	3	12	2	2	3	3	6	43	8	3	5	3	3	5	2017Hernández-M.	B. abortus	112	210		baboCR122	Cattle	2015	Costa Rica
4	5	3	12	2	2	3	3	6	43	8	3	5	3	3	5	2017Hernández-M.	B. abortus	112	210		baboCR123	Cattle	2015	Costa Rica
4	5	3	12	2	2	3	3	6	43	8	3	5	3	3	5	2017Hernández-M.	B. abortus	112	210		baboCR144	Cattle	2016	Costa Rica
4	5	3	12	2	2	3	3	6	43	8	3	5	3	3	5	2017Hernández-M.	B. abortus	112	210		baboCR77B	Cattle	2014	Costa Rica
4	5	3	12	2	2	3	3	6	43	8	3	5	3	3	5	2017Hernández-M.	B. abortus	112	210		baboCR78B	Cattle	2014	Costa Rica
4	5	3	12	2	2	3	3	6	43	8	3	5	3	3	5	2017Hernández-M.	B. abortus	112	210		baboCR79B	Cattle	2014	Costa Rica
4	5	3	12	2	2	3	3	6	43	8	3	5	3	3	5	2017Hernández-M.	B. abortus	112	210		baboCR80B	Cattle	2014	Costa Rica
4	5	3	12	2	2	3	3	6	43	8	3	5	3	3	5	2017Hernández-M.	B. abortus	112	210		baboCR81	Cattle	2014	Costa Rica
4	5	3	12	2	2	3	3	6	43	8	3	5	3	3	5	2017Hernández-M.	B. abortus	112	210		baboCR82	Cattle	2014	Costa Rica
4	5	3	12	2	2	3	3	6	43	8	3	5	3	3	5	2017Hernández-M.	B. abortus	112	210		baboCR83	Cattle	2014	Costa Rica
4	5	3	12	2	2	3	3	6	43	8	3	5	3	3	5	2017Hernández-M.	B. abortus	112	210		baboCR86	Cattle	2014	Costa Rica
4	5	3	12	2	2	3	3	6	43	8	3	5	3	3	5	2017Hernández-M.	B. abortus	112	210		baboCR97	Cattle	2015	Costa Rica
4	5	3	12	2	2	3	3	6	43	8	3	5	3	3	5	2017Hernández-M.	B. abortus	112	210		baboCR98	Cattle	2015	Costa Rica
4	5	3	12	2	2	3	3	6	43	8	3	5	3	3	5	2017Hernández-M.	B. abortus	112	210		baboCR104	Cattle	2015	Costa Rica
4	5	4	12	2	2	3	3	6	43	8	3	5	7	3	7	SRR1371295	B. abortus	28	82	1	45/20			
4	5	4	12	2	3	3	3	6	42	8	3	7	3	3	5	2017Hernández-M.	B. abortus	27	371		baboCR23	Cattle	2003	Costa Rica
4	5	4	12	2	3	3	3	6	42	8	3	7	3	3	5	2017Hernández-M.	B. abortus	27	371		baboCR25	Cattle	2003	Costa Rica
4	5	4	12	2	3	3	3	6	42	8	3	7	3	3	5	2017Hernández-M.	B. abortus	27	371		baboCR29	Cattle	2003	Costa Rica
4	5	4	12	2	3	3	3	6	42	8	3	7	3	3	5	2017Hernández-M.	B. abortus	27	371		baboCR16	Cattle	2003	Costa Rica
4	5	4	12	2	3	3	3	6	42	8	3	7	3	3	5	2017Hernández-M.	B. abortus	27	371		baboCR8	Cattle	2003	Costa Rica
4	5	4	12	2	3	3	3	6	42	8	3	7	3	3	5	2017Hernández-M.	B. abortus	27	371		baboCR6	Cattle	2003	Costa Rica
4	5	4	12	2	3	3	3	6	42	8	3	7	3	3	5	2017Hernández-M.	B. abortus	27	371		baboCR19	Cattle	2003	Costa Rica
4	5	4	12	2	3	3	3	6	41	8	3	7	3	3	5	2017Hernández-M.	B. abortus	27	372		baboCR58	Cattle	2010	Costa Rica
4	5	4	12	2	3	3	3	6	41	8	3	7	3	3	5	2017Hernández-M.	B. abortus	27	372		baboCR57	Cattle	2010	Costa Rica
4	5	4	12	2	3	3	3	6	43	8	3	7	3	3	5	2006LeFleche#120	B. abortus	27	79			Cattle		USA
4	5	4	12	2	3	3	3	6	43	8	3	7	3	3	5	2009Her#008	B. abortus bv1	27	79		2308_KRef09	Cattle		USA
4	5	4	12	2	3	3	3	6	43	8	3	7	3	3	5	2009Her#009	B. abortus Rough	27	79		RB51_KRef10	Cattle		USA
4	5	4	12	2	3	3	3	6	43	8	3	7	3	3	5	2009Her#112	B. abortus bv1	27	79		KBa101	Cattle	1999	Chungbuk, South Korea
4	5	4	12	2	3	3	3	6	43	8	3	7	3	3	5	2012Ferreira#298	B. abortus Rough	27	79		RB51_LNIV-3.	Cattle	2007	S. Miguel, Azores, Port.
4	5	4	12	2	3	3	3	6	43	8	3	7	3	3	5	2012Ferreira#299	B. abortus Rough	27	79		RB51_LNIV-2.	Cattle	2005	Tras-os-Montes, Portugal
4	5	4	12	2	3	3	3	6	43	8	3	7	3	3	5	2012Ferreira#300	B. abortus bv1	27	79		REF 2308	Cattle		USA
4	5	4	12	2	3	3	3	6	43	8	3	7	3	3	5	2012Ferreira#301	B. abortus Rough	27	79		RB51	Cattle		USA
4	5	4	12	2	3	3	3	6	43	8	3	7	3	3	5	2013Garofolo_12.	B. abortus Rough	27	79		RB51_12177	Buffalo	2011	Altavilla Silentina,Italy
4	5	4	12	2	3	3	3	6	43	8	3	7	3	3	5	2015Shevtsov#030	B. abortus Rough	27	79		RB51			USA
4	5	4	12	2	3	3	3	6	43	8	3	7	3	3	5	2016Shevtsova#092	B. abortus Rough	27	79		RB51	vaccine		
4	5	4	12	2	3	3	3	6	43	8	3	7	3	3	5	2017Hernández-M.	B. abortus	27	79		baboCR63	Cattle	2012	Costa Rica
4	5	4	12	2	3	3	3	6	43	8	3	7	3	3	5	2017Hernández-M.	B. abortus	27	79		baboCR64	Cattle	2012	Costa Rica
4	5	4	12	2	3	3	3	6	43	8	3	7	3	3	5	2018Vergnaud#0006	B. abortus bv1	27	79		BCCN#R23			
4	5	4	12	2	3	3	3	6	43	8	3	7	3	3	5	GCA_000054005	B. abortus bv1	27	79	5	REF 2308	Human	2005	India
4	5	4	12	2	3	3	3	6	43	8	3	7	3	3	5	GCA_001715385	B. abortus	27	79	5	2002734562	Rodent	2005	San Diego, California
4	5	4	12	2	3	3	3	6	43	8	3	7	3	3	5	SRR1371294	B. abortus	27	79	5	2308			
4	5	4	12	2	3	3	3	6	43	8	3	7	3	3	5	SRR1371298	B. abortus Rough	27	79	5	RB51			
4	5	4	12	2	3	3	3	6	43	8	3	7	3	3	5	SRR1371362	B. abortus Rough	27	79	5	RB51	Human		United States - IA
4	5	4	12	2	3	3	3	6	43	8	3	7	3	3	5	SRR3096342	B. abortus	27	79		B02-0268	Deer/El.		USA: Idaho
4	5	4	12	2	3	3	3	6	43	8	3	6	3	3	5	2017Hernández-M.	B. abortus	27	79		baboCR76	Cattle	2014	Costa Rica
4	5	4	12	2	3	3	3	6	43	8	3	3	3	3	5	2017Hernández-M.	B. abortus	27	79		baboCR87	Cattle	2014	Costa Rica
4	5	4	12	2	3	3	3	6	43	8	3	3	3	3	5	2017Hernández-M.	B. abortus	27	79		baboCR92	Cattle	2015	Costa Rica
4	5	4	12	2	3	3	3	6	43	8	3	3	3	3	5	SRR1371363	B. abortus Rough	27	79	5	RB51	Cattle		United States - MT
4	5	4	12	2	2	3	3	6	43	8	3	3	3	3	5	2017Hernández-M.	B. abortus	28	82		baboCR126	Cattle	2015	Costa Rica
4	5	4	12	2	2	3	3	6	43	8	3	3	3	3	5	2017Hernández-M.	B. abortus	28	82		baboCR157	Cattle	2015	Costa Rica
4	5	4	12	2	2	3	3	6	43	8	3	3	3	3	5	2018Vergnaud#0226	B. abortus bv1	28	82		BCCN#02-29	Buffalo	2002	India
4	5	4	12	2	3	3	3	6	43	8	5	5	3	3	5	SRR1371304	B. abortus	27	79	5	B01-0352	Cattle		United States - KS
4	5	4	12	2	3	3	3	6	43	8	5	5	3	3	5	SRR1371374	B. abortus	27	79	5	B94-1564	Cattle		United States - MO
4	5	4	12	2	3	3	3	6	43	8	3	5	3	3	5	2017Hernández-M.	B. abortus	27	79		baboCR88	Cattle	2014	Costa Rica

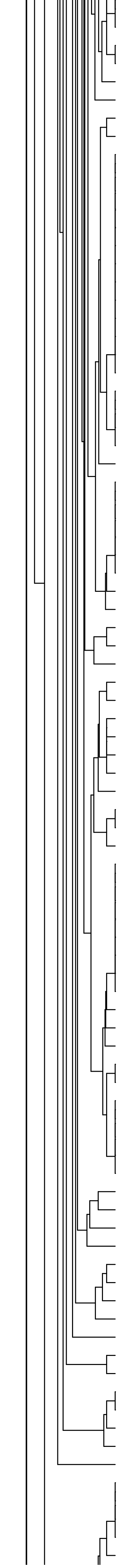

4	5	4	12	2	3	3	3	6	43	8	3	5	3	3	5	2017Hernández-M.	B. abortus	27	79		baboCR89	Cattle	2015	Costa Rica
4	5	4	12	2	3	3	3	6	43	8	3	5	3	3	5	SRR1371385	B. abortus	27	79	5	B98-0862	Cattle		United States - TX
4	5	4	12	2	3	3	3	6	43	8	7	5	3	3	5	2017Hernández-M.	B. abortus	27	79		baboCR69A	Cattle	2014	Costa Rica
4	5	4	12	2	3	3	3	6	43	8	7	5	3	3	5	2017Hernández-M.	B. abortus	27	79		baboCR70A	Cattle	2014	Costa Rica
4	5	4	12	2	3	3	3	6	43	8	3	5	3	3	8	SRR1371327	B. abortus	27	79	5	B11-0202	Cattle		United States - TX
4	5	4	12	1	3	3	3	6	43	8	3	4	3	3	5	2006LeFleche#136	B. abortus bv1	31	74		BfR 96	Cattle		Zimbabwe
4	5	4	12	2	1	3	3	6	42	8	3	5	3	3	5	2017Hernández-M.	B. abortus	29	370		baboCR10	Cattle	2003	Costa Rica
4	5	4	12	2	2	3	3	6	43	8	3	5	3	3	5	2017Hernández-M.	B. abortus	28	82		baboCR133	Cattle	2016	Costa Rica
4	5	4	12	2	1	3	3	6	42	8	3	4	3	3	5	2017Hernández-M.	B. abortus	29	370		baboCR11	Cattle	2003	Costa Rica
4	5	4	12	2	1	3	3	6	42	8	3	4	3	3	5	2017Hernández-M.	B. abortus	29	370		baboCR4	Cattle	2003	Costa Rica
4	5	4	12	2	1	3	3	6	42	8	3	4	3	3	5	2017Hernández-M.	B. abortus	29	370		baboCR22	Cattle	2003	Costa Rica
4	5	4	12	2	1	3	3	6	42	8	3	4	3	3	5	2017Hernández-M.	B. abortus	29	370		baboCR27	Cattle	2003	Costa Rica
4	5	4	12	2	1	3	3	6	42	8	3	4	3	3	5	2017Hernández-M.	B. abortus	29	370		baboCR30	Cattle	2003	Costa Rica
4	5	4	12	2	1	3	3	6	42	8	3	4	3	3	5	2017Hernández-M.	B. abortus	29	370		baboCR18	Cattle	2003	Costa Rica
4	5	4	12	2	1	3	3	6	42	8	3	4	3	3	5	2017Hernández-M.	B. abortus	29	370		baboCR7	Cattle	2003	Costa Rica
4	5	4	12	2	1	3	3	6	42	8	3	4	3	3	5	2017Hernández-M.	B. abortus	29	370		baboCR12	Cattle	2003	Costa Rica
4	5	4	12	2	1	3	3	6	42	8	3	4	3	3	5	2017Hernández-M.	B. abortus	29	370		baboCR5	Cattle	2003	Costa Rica
4	5	4	12	2	1	3	3	6	42	8	3	4	3	3	5	2017Hernández-M.	B. abortus	29	370		baboCR15	Cattle	2003	Costa Rica
4	5	4	12	2	1	3	3	6	42	8	3	4	3	3	5	2017Hernández-M.	B. abortus	29	370		baboCR13	Cattle	2003	Costa Rica
4	5	4	12	2	1	3	3	6	42	8	3	4	3	3	5	2017Hernández-M.	B. abortus	29	370		baboCR14	Cattle	2003	Costa Rica
4	5	4	12	2	1	3	3	6	42	8	3	4	3	3	5	2017Hernández-M.	B. abortus	29	370		baboCR17	Cattle	2003	Costa Rica
4	5	4	12	2	1	3	3	6	43	8	3	4	3	3	5	2006LeFleche#003	B. abortus bv2	29	80		REF 86/8/59	Cattle		England
4	5	4	12	2	1	3	3	6	43	8	3	4	3	3	5	2009Her#002	B. abortus bv2	29	80		KRef02	Cattle		England
4	5	4	12	2	1	3	3	6	43	8	3	4	3	3	5	2012Ferreira#212	B. abortus bv2	29	80		REF 86/8/59	Cattle		
4	5	4	12	2	1	3	3	6	43	8	3	4	3	3	5	GCA_000740375	B. abortus bv2	29	80	1	REF 86/8/59			
3	5	4	12	2	1	3	3	6	43	8	3	4	3	3	5	SRR032623	B. abortus bv2			1	86/8/59			
4	5	4	12	2	2	3	3	6	41	8	3	4	3	3	5	2012Ferreira#215	B. abortus bv1	28	181		LNIV-21Ba1-01	Cattle	2001	Azores, Portugal
4	5	4	12	2	2	3	3	6	41	8	3	4	3	3	5	2012Ferreira#217	B. abortus bv1	28	181		LNIV-T243Ba1.	Cattle	2002	Terceira, Azores, Portu.
4	5	4	12	2	2	3	3	6	41	8	3	4	3	3	5	2012Ferreira#218	B. abortus bv1	28	181		LNIV-T244Ba1.	Cattle	2002	Terceira, Azores, Portu.
4	5	4	12	2	2	3	3	6	41	8	3	4	3	3	5	2012Ferreira#219	B. abortus bv1	28	181		LNIV-T245Ba1.	Cattle	2002	Terceira, Azores, Portu.
4	5	4	12	2	2	3	3	6	41	8	3	4	3	3	5	2012Ferreira#220	B. abortus bv1	28	181		LNIV-T246Ba3.	Cattle	2002	Terceira, Azores, Portu.
4	5	4	12	2	2	3	3	6	41	8	3	4	3	3	5	2012Ferreira#222	B. abortus bv1	28	181		LNIV-T248Ba1.	Cattle	2002	Terceira, Azores, Portu.
4	5	4	12	2	2	3	3	6	41	8	3	5	3	3	5	2012Ferreira#221	B. abortus bv1	28	181		LNIV-T247Ba1.	Cattle	2002	Terceira, Azores, Portu.
4	5	4	12	2	2	3	3	6	41	8	3	4	3	3	6	2012Ferreira#216	B. abortus bv1	28	181		LNIV-T242Ba1.	Cattle	2002	Terceira, Azores, Portu.
4	5	4	13	2	2	3	3	6	43	8	3	4	3	6	6	2013Minharro#114	B. abortus bv1	159	336		SP 9	Cattle		Sao Paulo, 01036970, .
4	5	4	13	2	2	3	3	6	43	8	3	4	3	6	5	2013Minharro#115	B. abortus bv1	159	336		SP 10	Cattle		Sao Paulo, 01036970, .
4	5	4	13	1	2	3	3	6	43	8	3	4	3	3	6	2012Ferreira#302	B. abortus bv1	99	184		LNIV-399Ba1-06	Cattle	2006	Terceira, Azores, Portu.
4	5	3	12	2	2	3	3	6	43	8	3	4	3	10	4	SRR1371301	B. abortus	112	210	1	B00-0220	Cattle		United States - TX
4	5	3	12	2	2	3	3	6	43	8	3	4	3	5	4	SRR1371377	B. abortus	112	210	1	B95-1140	Cattle		United States - MO
4	5	3	12	2	2	3	3	6	43	8	3	6	3	6	4	SRR1371300	B. abortus	112	210		B00-0092	Cattle		United States - TX
4	5	3	12	2	2	3	3	6	43	8	3	6	3	4	4	SRR1371303	B. abortus	112	210		B00-0751	Cattle		United States - TX
4	5	3	12	2	2	3	3	6	43	8	3	6	3	5	4	SRR1371375	B. abortus	112	210	1	B95-0116	Cattle		United States - MS
4	5	3	12	2	2	3	3	6	43	8	3	6	3	3	4	SRR1371381	B. abortus	112	210		B98-0001	Cattle		United States - TX
4	5	3	12	2	2	3	3	6	43	8	3	3	3	9	4	SRR1371302	B. abortus	112	210	1	B00-0624	Cattle		United States - TX
4	5	3	12	2	2	3	3	6	43	8	3	5	3	5	6	SRR3096481	B. abortus	112	210		B12-0416	Deer/El.		USA: Wyoming
4	5	3	12	2	2	3	3	6	43	8	3	5	3	5	6	SRR3096510	B. abortus	112	210	1	B-GF032709	Deer/El.		USA: Wyoming
4	5	3	12	2	2	3	3	6	43	8	3	6	3	5	6	SRR3096419	B. abortus	112	210		B11-0148	Deer/El.		USA: Wyoming
4	5	3	12	2	2	3	3	6	43	8	3	5	3	4	7	SRR1371312	B. abortus	112	210	1	B04-0031	Cattle		United States - TX
4	5	3	12	2	2	3	3	6	43	8	3	5	3	4	7	SRR1371320	B. abortus	112	210	1	B08-0665	Cattle		United States - WY
4	5	3	12	2	2	3	3	6	43	8	3	5	3	4	7	SRR3096330	B. abortus	112	210	1	B01-0638	Deer/El.		USA: Idaho
4	5	3	12	2	2	3	3	6	43	8	3	5	3	4	7	SRR3096331	B. abortus	112	210	1	B01-0642	Deer/El.		USA: Idaho
4	5	3	12	2	2	3	3	6	43	8	3	5	3	4	7	SRR3096350	B. abortus	112	210	1	B04-0196	Deer/El.		USA: Idaho
4	5	3	12	2	2	3	3	6	43	8	3	5	3	4	7	SRR3096371	B. abortus	112	210	1	B08-0666	Cattle		USA: Wyoming
4	5	3	12	2	2	3	3	6	43	8	3	5	3	4	7	SRR3096381	B. abortus	112	210	1	B09-0009	Cattle		USA: Wyoming
4	5	3	12	2	2	3	3	6	43	8	3	5	3	4	7	SRR3096417	B. abortus	112	210	1	B11-0146	Deer/El.		USA: Wyoming
4	5	4	12	2	2	3	3	6	43	8	3	5	3	4	7	2013Minharro#097	B. abortus bv1	28	82		RS 1	Cattle	2004	Porto Alegre, 90570020.
4	5	3	12	2	2	3	3	6	43	8	3	4	3	4	7	SRR3096341	B. abortus	112	210	1	B02-0246	Deer/El.		USA: Idaho
4	5	3	12	2	2	3	3	6	43	8	3	5	3	4	5	SRR3096471	B. abortus	112	210	1	B12-0406	Deer/El.		USA: Wyoming
4	5	3	12	2	2	3	3	6	43	8	4	5	3	4	8	SRR1371357	B. abortus	112	210	1	B12-0332	Bison		United States - ID
4	5	3	12	2	2	3	3	6	43	8	4	5	3	4	8	SRR3096499	B. abortus	112	210	1	B13-0093	Bison		USA: Idaho
4	5	3	12	2	2	3	3	6	43	8	3	5	3	4	8	SRR1371316	B. abortus	112	210	1	B05-0022	Cattle		United States - WY
4	5	3	12	2	2	3	3	6	43	8	3	5	3	4	8	SRR3096356	B. abortus	112	210	1	B05-0338	Cattle		USA: Wyoming
4	5	3	12	2	2	3	3	6	43	8	3	5	3	4	8	SRR3096357	B. abortus	112	210	1	B05-0756	Deer/El.		USA: Idaho
4	5	3	12	2	2	3	3	6	43	8	3	5	3	4	8	SRR3096423	B. abortus	112	210	1	B11-0159	Deer/El.		USA: Wyoming
4	5	3	12	2	2	3	3	6	43	8	3	5	3	4	8	SRR3096457	B. abortus	112	210	1	B12-0333	Bison		USA: Idaho
4	5	4	12	2	2	4	3	6	43	9	4	5	3	3	5	2013Minharro#081	B. abortus bv1	152	257		10_jun	Cattle	2006	Porto Alegre, 90570020.
4	5	4	12	2	2	3	3	6	43	9	4	5	3	5	5	2013Minharro#105	B. abortus bv1	28	235		RS 10	Cattle		Porto Alegre, 90570020.
4	5	4	12	2	2	3	3	7	43	8	4	5	3	5	5	2013Minharro#106	B. abortus bv1	28	227		SP 1	Cattle		Sao Paulo, 01036970, .
4	5	4	12	2	2	3	3	6	43	8	4	5	4	6	5	2013Minharro#103	B. abortus bv1	28	82		RS 8	Cattle	2007	Porto Alegre, 90570020.
4	5	4	12	2	2	3	3	6	45	7	3	4	3	6	5	2013Minharro#020	B. abortus bv2	28	334		49	Cattle	2006	Governador Valadares,.
4	5	4	12	2	2	3	3	6	45	7	3	4	3	5	5	2013Minharro#022	B. abortus bv2	28	334		54	Cattle	2006	Porto Alegre, 90570020.
4	5	4	12	2	2	3	3	6	45	7	3	4	3	5	4	2013Minharro#021	B. abortus bv2	28	334		50	Cattle	2006	Governador Valadares,.
4	5	4	12	2	2	3	3	6	45	7	3	5	3	4	5	2013Minharro#023	B. abortus bv2	28	334		55	Cattle	2006	Porto Alegre, 90570020.
4	6	3	12	2	2	3	3	6	43	8	5	4	3	5	5	2017Hanot-Mamb.	B. abortus bv1	185	363		L3/173	Human	2010	Belgium
4	5	4	12	2	2	3	3	6	43	8	3	7	2	2	4	2017Liu#117	B. abortus bv1	28	82		NM-25	Ovine	2016	
4	5	4	12	2	2	3	3	6	43	8	3	7	2	2	3	2017Liu#118	B. abortus bv1	28	82		NM-26	Ovine	2016	
4	5	4	11	2	3	3	3	5	42	8	3	6	3	3	6	2017Hernández-M.	B. abortus	192	381		baboCR28	Cattle	2003	Costa Rica
4	5	4	11	2	3	3	3	5	42	8	3	6	3	3	6	2017Hernández-M.	B. abortus	192	381		baboCR9	Cattle	2003	Costa Rica
4	5	4	11	2	3	3	3	5	40	8	3	6	3	3	6	2017Hernández-M.	B. abortus	192	382		baboCR1	Cattle	2003	Costa Rica
4	5	4	12	2	3	3	3	5	42	8	3	6	3	3	6	2017Hernández-M.	B. abortus	27	373		baboCR20	Cattle	2003	Costa Rica
4	5	3	12	2	2	2	3	6	36	8	6	6	3	3	2	2013Garofolo_1265	B. abortus bv3	111	209		1265	Cattle	2011	Apricena,Italy
3	5	4	11	2	2	3	3	7	41	8	5	4	3	7	6	2018Vergnaud#0201	B. abortus bv3	34	317		BCCN#78-47	Cattle	1978	Oussouye ,Ziguinchor, .
3	5	4	11	2	2	3	3	7	41	8	5	4	3	7	6	2018Vergnaud#1061	B. abortus bv3	34	317		BCCN#78-46	Cattle	1978	Oussouye ,Ziguinchor, .
3	5	4	11	2	2	3	3	7	41	8	5	4	3	7	6	2018Vergnaud#1358	B. abortus bv3	34	317		BCCN#78-34	Cattle	1978	Oussouye ,Ziguinchor, .
3	5	4	11	2	2	3	3	7	41	8	5	4	3	7	6	2018Vergnaud#1360	B. abortus bv3	34	317		BCCN#78-44	Cattle	1978	Oussouye ,Ziguinchor, .
3	5	4	11	2	2	3	3	7	41	8	5	4	3	5	6	2018Vergnaud#1059	B. abortus bv3	34	317		BCCN#78-136	Cattle	1978	Sare Yoro Soudy ,Velin.

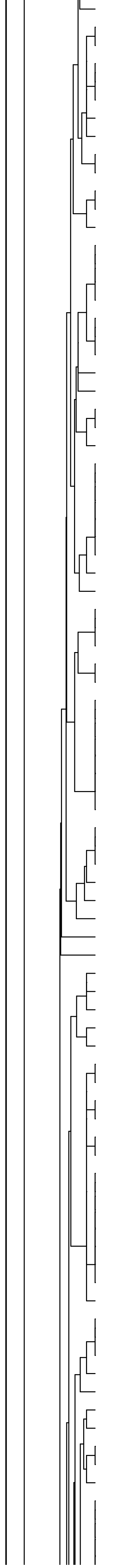

3	5	4	11	2	2	3	3	7	41	8	5	4	8	5	6	2018Vergnaud#0185	B. abortus bv3	34	317	BCCN#77-116	Cattle	1977	Tankon ,Senegal-Gamb.
3	5	4	11	2	2	3	3	7	41	8	5	4	3	5	7	2018Vergnaud#0759	B. abortus bv3	34	317	BCCN#78-49	Cattle	1978	Oussouye ,Ziguinchor, .
3	5	4	11	2	2	3	3	7	41	8	5	4	3	5	7	2018Vergnaud#1229	B. abortus bv3	34	317	BCCN#77-34	Cattle	1977	Oudoucar ,Sedhiou Nor.
3	5	4	11	2	2	3	3	7	41	8	5	4	3	8	7	2018Vergnaud#0239	B. abortus bv3	34	317	BCCN#82-39	Cattle	1982	Guinea-Bissau
3	5	4	11	2	2	3	3	7	41	8	5	4	3	8	7	2018Vergnaud#0240	B. abortus bv3	34	317	BCCN#82-40	Cattle	1982	Guinea-Bissau
3	5	4	11	2	2	3	3	7	41	8	5	4	3	8	7	2018Vergnaud#0241	B. abortus bv3	34	317	BCCN#82-41	Cattle	1982	Guinea-Bissau
3	5	4	11	2	2	3	3	7	41	8	5	4	3	7	7	2018Vergnaud#0243	B. abortus bv3	34	317	BCCN#82-43	Cattle	1982	Guinea-Bissau
3	5	4	11	2	2	3	3	7	41	8	5	4	3	6	7	2018Vergnaud#0244	B. abortus bv3	34	317	BCCN#82-44	Cattle	1982	Guinea-Bissau
3	5	4	11	2	2	3	3	7	41	8	5	4	3	8	8	2018Vergnaud#0242	B. abortus bv3	34	317	BCCN#82-42	Cattle	1982	Guinea-Bissau
3	5	4	11	2	2	3	3	7	41	8	5	4	3	8	8	2018Vergnaud#0333	B. abortus bv3	34	317	BCCN#78-141	Cattle	1978	Dialakha ,Velingara, S.
3	5	4	11	2	2	3	3	7	41	8	5	3	3	6	6	2018Vergnaud#0340	B. abortus bv3	34	317	BCCN#79-179	Cattle	1979	Senegal
3	5	4	11	2	2	3	3	7	41	8	5	3	3	6	6	2018Vergnaud#0797	B. abortus bv3	34	317	BCCN#79-165	Cattle	1979	Senegal
3	5	4	11	2	2	3	3	7	41	8	5	3	3	6	7	2018Vergnaud#0795	B. abortus bv3	34	317	BCCN#79-158	Cattle	1979	Senegal
3	5	4	11	2	2	3	3	7	41	8	5	6	3	4	4	2018Vergnaud#0275	B. abortus bv3	34	317	BCCN#83-228	Cattle	1983	Rwanda
3	5	4	11	2	2	3	3	7	41	8	5	6	3	4	4	2018Vergnaud#0276	B. abortus bv3	34	317	BCCN#83-230	Cattle	1983	Rwanda
3	5	4	11	2	2	3	3	7	41	8	5	6	3	4	4	2018Vergnaud#0309	B. abortus bv3	34	317	BCCN#83-231	Cattle	1983	Rwanda
3	5	4	11	2	2	3	3	7	41	8	5	6	3	4	4	2018Vergnaud#0312	B. abortus bv3	34	317	BCCN#83-229	Cattle	1983	Rwanda
3	5	4	11	2	2	3	3	7	41	8	5	6	3	5	4	2018Vergnaud#0271	B. abortus bv3	34	317	BCCN#83-17	Cattle	1983	Rwanda
3	5	4	11	2	2	3	3	7	41	8	5	6	3	5	4	2018Vergnaud#0272	B. abortus bv3	34	317	BCCN#83-18	Cattle	1983	Rwanda
3	5	4	11	2	2	3	3	7	41	8	5	6	3	5	4	2018Vergnaud#0273	B. abortus bv3	34	317	BCCN#83-19	Cattle	1983	Rwanda
3	5	4	11	2	2	3	3	7	41	8	5	8	3	9	4	2018Vergnaud#0277	B. abortus bv3	34	317	BCCN#83-232	Cattle	1983	Rwanda
3	5	4	11	2	2	3	3	7	41	8	5	7	3	6	4	2018Vergnaud#0270	B. abortus bv3	34	317	BCCN#83-16	Cattle	1983	Rwanda
3	5	4	11	2	2	3	3	7	41	8	5	7	3	4	6	2018Vergnaud#0753	B. abortus bv3	34	317	BCCN#77-201	Cattle	1977	Keur Sacoumba ,Kaola.
3	5	4	11	2	2	3	3	7	41	8	5	7	3	4	6	2018Vergnaud#1055	B. abortus bv3	34	317	BCCN#77-185	Cattle	1977	Ndoumbouthie ,Kaolack.
3	5	4	11	2	2	3	3	7	41	8	5	6	3	4	6	2018Vergnaud#0331	B. abortus bv3	34	317	BCCN#77-208	Cattle	1977	Ouakngouna ,Kaolack,.
3	5	4	11	2	2	3	3	7	41	8	5	6	3	3	5	2018Vergnaud#0193	B. abortus bv3	34	317	BCCN#78-129	Cattle	1978	Pilapitiance ,Velingara,.
3	5	4	11	2	2	3	3	7	41	8	5	6	3	3	5	2018Vergnaud#0195	B. abortus bv3	34	317	BCCN#78-134	Cattle	1978	Temento Madia ,Veling.
3	5	4	11	2	2	3	3	7	41	8	5	6	3	3	5	2018Vergnaud#0324	B. abortus bv3	34	317	BCCN#77-118	Cattle	1977	Senoba ,Senegal-Gamb.
3	5	4	11	2	2	3	3	7	41	8	5	6	3	3	5	2018Vergnaud#0332	B. abortus bv3	34	317	BCCN#78-128	Cattle	1978	Pilapitiance ,Velingara,.
3	5	4	11	2	2	3	3	7	41	8	5	6	3	3	5	2018Vergnaud#0757	B. abortus bv3	34	317	BCCN#78-130	Cattle	1978	Pilapitiance ,Velingara,.
3	5	4	11	2	2	3	3	7	41	8	5	6	3	3	5	2018Vergnaud#0758	B. abortus bv3	34	317	BCCN#78-131	Cattle	1978	Pilapitiance ,Velingara,.
3	5	4	11	2	2	3	3	7	41	8	5	6	3	3	7	2018Vergnaud#1363	B. abortus bv3	34	317	BCCN#79-156	Cattle	1979	Senegal
3	5	4	11	2	2	3	3	7	41	8	5	5	3	3	7	2018Vergnaud#1053	B. abortus bv3	34	317	BCCN#76-425	Cattle	1976	Sénégal Oriental, Sene.
3	5	4	11	2	2	3	3	7	41	8	6	4	3	6	6	2018Vergnaud#0329	B. abortus bv3	34	317	BCCN#79-152	Cattle	1979	Senegal
3	5	4	11	2	2	3	3	7	41	8	6	4	3	6	6	2018Vergnaud#0388	B. abortus bv3	34	317	BCCN#79-153	Cattle	1979	Senegal
3	5	4	11	2	2	3	3	7	41	8	6	4	3	6	6	2018Vergnaud#0752	B. abortus bv3	34	317	BCCN#77-183	Cattle	1977	Ndoumbouthie ,Kaolack.
3	5	4	11	2	2	3	3	7	41	8	6	9	3	6	7	2018Vergnaud#0205	B. abortus bv3	34	317	BCCN#92-104	Cattle	1992	Guinea
3	5	4	11	2	2	3	3	7	41	8	6	9	3	6	7	2018Vergnaud#0245	B. abortus bv3	34	317	BCCN#92-105	Cattle	1992	Guinea
3	5	4	11	2	2	3	3	7	41	8	6	6	3	11	6	2011Muendo#06	B. abortus bv3	34	317	6-KEBa1	Cattle	2009	Eastern, Kenya
3	5	4	11	2	2	3	3	7	41	8	6	6	3	11	6	2011Muendo#07	B. abortus bv3	34	317	7-KEBa1	Cattle	2009	Eastern, Kenya
3	5	4	11	2	2	3	3	7	41	8	6	6	3	11	6	2011Muendo#08	B. abortus bv3	34	317	8-KEBa1	Cattle	2009	Eastern, Kenya
3	5	4	11	2	2	3	3	7	41	8	6	6	3	11	6	2011Muendo#09	B. abortus bv3	34	317	9-KEBa1	Cattle	2009	Eastern, Kenya
3	5	4	11	2	2	3	3	7	41	8	6	6	3	11	6	2011Muendo#10	B. abortus bv3	34	317	10-KEBa1	Cattle	2009	Eastern, Kenya
3	5	4	11	2	2	3	3	7	41	8	6	6	3	11	6	2011Muendo#12	B. abortus bv3	34	317	12-KEBa1	Cattle	2009	Central, Kenya
3	5	4	11	2	2	3	3	7	41	8	6	6	3	11	6	2011Muendo#13	B. abortus bv3	34	317	13-KEBa1	Cattle	2009	Central, Kenya
3	5	4	11	2	2	3	3	10	41	8	5	6	3	8	6	2018Vergnaud#0790	B. abortus bv3	34	315	BCCN#78-38	Cattle	1978	Loudia Wolof ,Ziguincho.
3	5	4	11	2	2	3	3	10	41	8	5	6	3	8	6	2018Vergnaud#0791	B. abortus bv3	34	315	BCCN#78-39	Cattle	1978	Effisao ,Ziguinchor, Se.
3	5	4	11	2	2	3	3	10	41	8	5	6	3	8	6	2018Vergnaud#1359	B. abortus bv3	34	315	BCCN#78-41	Cattle	1978	Effisao ,Ziguinchor, Se.
3	5	4	11	2	2	3	3	10	41	8	5	6	3	9	6	2018Vergnaud#0199	B. abortus bv3	34	315	BCCN#78-37	Cattle	1978	Loudia Wolof ,Ziguincho.
3	5	4	11	2	2	3	3	9	41	8	5	6	3	8	6	2018Vergnaud#0200	B. abortus bv3	34	316	BCCN#78-42	Cattle	1978	Santiaba Wolof ,Ziguin.
3	5	4	11	2	2	3	3	10	41	8	7	6	3	6	6	2018Vergnaud#1057	B. abortus bv3	34	315	BCCN#77-36	Cattle	1977	Marandan ,Sedhiou Nor.
2	5	4	11	2	2	3	3	7	41	8	5	7	3	5	7	2018Vergnaud#0180	B. abortus bv3	139	314	BCCN#77-192	Cattle	1977	Nioro du Rip ,Kaolack, .
3	5	4	11	2	2	3	3	7	41	8	5	8	4	4	4	2018Vergnaud#0269	B. abortus bv3	34	317	BCCN#83-15	Cattle	1983	Rwanda
3	5	4	11	2	2	3	3	8	41	8	4	6	3	5	6	2018Vergnaud#0190	B. abortus bv3	34	64	BCCN#77-58	Cattle	1977	Bounkiling ,Senegal-G.
3	5	4	11	2	2	3	3	7	41	8	4	6	3	5	6	2018Vergnaud#1349	B. abortus bv3	34	317	BCCN#77-57	Cattle	1977	Kenieto ,Senegal-Gamb.
3	5	4	11	2	2	3	3	9	41	8	4	6	3	5	6	2018Vergnaud#1345	B. abortus bv3	34	316	BCCN#77-29	Cattle	1977	Ndiama ,Sedhiou Nord,.
3	5	4	11	2	2	3	3	9	41	8	4	6	8	5	7	2018Vergnaud#0184	B. abortus bv3	34	316	BCCN#76-411	Cattle	1976	Bignona, Senegal
3	5	4	11	2	2	3	3	9	41	8	4	6	3	5	7	2018Vergnaud#0750	B. abortus bv3	34	316	BCCN#76-318	Cattle	1976	Senegal
3	5	4	11	2	2	3	3	8	41	8	4	6	3	5	5	2018Vergnaud#0782	B. abortus bv3	34	64	BCCN#77-12	Cattle	1977	Dianayel ,Kolda, Senegal
3	5	4	11	2	2	3	3	8	41	8	4	6	3	5	5	2018Vergnaud#1054	B. abortus bv3	34	64	BCCN#77-16	Cattle	1977	Oumoulkoure ,Kolda, S.
3	5	4	11	2	2	3	3	8	41	8	4	6	3	6	5	2018Vergnaud#0756	B. abortus bv3	34	64	BCCN#77-42	Cattle	1977	Dandon ,Sedhiou Nord,.
3	5	4	11	2	2	3	3	8	41	8	4	6	3	6	5	2018Vergnaud#0788	B. abortus bv3	34	64	BCCN#78-21	Cattle	1978	Fanghotte ,Ziguinchor, .
3	5	4	11	2	2	3	3	8	41	8	4	6	3	4	5	2018Vergnaud#0327	B. abortus bv3	34	64	BCCN#77-207	Cattle	1977	Mbayene ,Kaolack, Se.
3	5	4	11	2	2	3	3	8	41	8	4	6	3	4	5	2018Vergnaud#1342	B. abortus bv3	34	64	BCCN#77-189	Cattle	1977	Djilor ,Kaolack, Senegal
3	5	4	11	2	2	3	3	8	41	8	4	6	3	7	5	2018Vergnaud#0202	B. abortus bv3	34	64	BCCN#78-55	Cattle	1978	Diatene ,Ziguinchor, S.
3	5	4	11	2	2	3	3	8	41	8	4	6	3	7	5	2018Vergnaud#0760	B. abortus bv3	34	64	BCCN#78-50	Cattle	1978	Diakene Wolof ,Ziguinc.
3	5	4	11	2	2	3	3	8	41	8	4	6	3	7	5	2018Vergnaud#0792	B. abortus bv3	34	64	BCCN#78-51	Cattle	1978	Diakene Wolof ,Ziguinc.
3	5	4	11	2	2	3	3	8	41	8	4	6	3	7	5	2018Vergnaud#1232	B. abortus bv3	34	64	BCCN#78-53	Cattle	1978	Diakene Wolof ,Ziguinc.
3	5	4	11	2	2	3	3	8	41	8	4	6	3	7	5	2018Vergnaud#1233	B. abortus bv3	34	64	BCCN#78-54	Cattle	1978	Diatene ,Ziguinchor, S.
3	5	4	11	2	2	3	3	8	41	8	4	6	3	7	5	2018Vergnaud#1361	B. abortus bv3	34	64	BCCN#78-56	Cattle	1978	Diatene ,Ziguinchor, S.
3	5	4	11	2	2	3	3	8	41	8	4	6	3	7	5	2018Vergnaud#1362	B. abortus bv3	34	64	BCCN#78-57	Cattle	1978	Diatene ,Ziguinchor, S.
3	5	4	11	2	2	3	3	8	41	8	4	6	3	8	5	2018Vergnaud#0793	B. abortus bv3	34	64	BCCN#78-52	Cattle	1978	Diakene Wolof ,Ziguinc.
3	5	4	11	2	2	3	3	8	41	8	5	5	3	4	7	2018Vergnaud#0192	B. abortus bv3	34	64	BCCN#77-70	Cattle	1977	Kaour ,Sedhiou Sud, S.
3	5	4	11	2	2	3	3	8	41	8	5	5	3	4	7	2018Vergnaud#1351	B. abortus bv3	34	64	BCCN#77-69	Cattle	1977	Kaour ,Sedhiou Sud, S.
3	5	4	11	2	2	3	3	8	41	8	5	5	3	4	7	2018Vergnaud#1352	B. abortus bv3	34	64	BCCN#77-71	Cattle	1977	Kaour ,Sedhiou Sud, S.
3	5	4	11	2	2	3	3	8	41	8	5	5	3	5	7	2018Vergnaud#1348	B. abortus bv3	34	64	BCCN#77-56	Cattle	1977	Sinthiou Diae ,Sedhiou.
3	5	4	11	2	2	3	3	8	41	8	5	5	3	5	6	2018Vergnaud#1228	B. abortus bv3	34	64	BCCN#77-33	Cattle	1977	Brosso ,Senegal-Gambi.
3	5	4	11	2	2	3	3	9	41	8	4	5	3	4	6	2018Vergnaud#1234	B. abortus bv3	34	316	BCCN#79-168	Cattle	1979	Senegal
3	5	4	11	2	2	3	3	9	41	8	4	5	3	4	5	2018Vergnaud#1350	B. abortus bv3	34	316	BCCN#77-67	Cattle	1977	Diattacounda ,Sedhiou.
3	5	4	11	2	2	3	3	9	41	8	4	5	3	4	7	2018Vergnaud#1058	B. abortus bv3	34	316	BCCN#77-62	Cattle	1977	Simbandi Ballante ,Sed.
3	5	4	11	2	2	3	3	9	41	8	4	5	3	4	7	2018Vergnaud#1353	B. abortus bv3	34	316	BCCN#77-72	Cattle	1977	Kaour ,Sedhiou Sud, S.
3	5	4	11	2	2	3	3	8	41	8	4	5	3	4	7	2018Vergnaud#1063	B. abortus bv3	34	64	BCCN#79-162	Cattle	1979	Senegal
3	5	4	11	2	2	3	3	8	41	8	4	5	3	4	6	2018Vergnaud#0183	B. abortus bv3	34	64	BCCN#76-402	Cattle	1976	Bignona, Senegal
3	5	4	11	2	2	3	3	8	41	8	4	5	3	4	6	2018Vergnaud#0198	B. abortus bv3	34	64	BCCN#78-32	Cattle	1978	Toubacouta ,Ziguinchor.
3	5	4	11	2	2	3	3	8	41	8	4	5	3	4	6	2018Vergnaud#0780	B. abortus bv3	34	64	BCCN#76-394	Cattle	1976	Bignona, Senegal
3	5	4	11	2	2	3	3	8	41	8	4	5	3	4	6	2018Vergnaud#0781	B. abortus bv3	34	64	BCCN#76-395	Cattle	1976	Bignona, Senegal

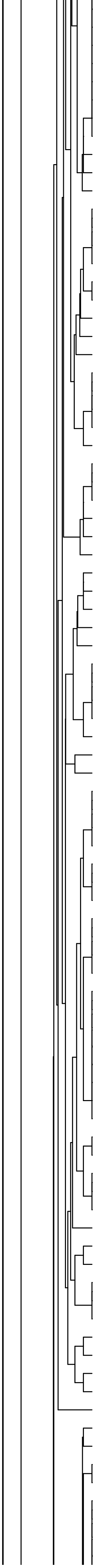

3	5	4	11	2	2	3	3	8	41	8	4	5	3	4	6	2018Vergnaud#0796	B. abortus bv3	34	64		BCCN#79-164	Cattle	1979	Senegal
3	5	4	11	2	2	3	3	8	41	8	4	5	3	4	6	2018Vergnaud#0799	B. abortus bv3	34	64		BCCN#79-172	Cattle	1979	Senegal
3	5	4	11	2	2	3	3	8	41	8	4	5	3	4	6	2018Vergnaud#1049	B. abortus bv3	34	64		BCCN#76-396	Cattle	1976	Bignona, Senegal
3	5	4	11	2	2	3	3	8	41	8	4	5	3	4	6	2018Vergnaud#1050	B. abortus bv3	34	64		BCCN#76-397	Cattle	1976	Bignona, Senegal
3	5	4	11	2	2	3	3	8	41	8	4	5	3	4	6	2018Vergnaud#1052	B. abortus bv3	34	64		BCCN#76-401	Cattle	1976	Bignona, Senegal
3	5	4	11	2	2	3	3	8	41	8	4	5	3	4	6	2018Vergnaud#1236	B. abortus bv3	34	64		BCCN#79-173	Cattle	1979	Senegal
3	5	4	11	2	2	3	3	8	41	8	4	5	3	4	6	2018Vergnaud#1356	B. abortus bv3	34	64		BCCN#78-31	Cattle	1978	Toubacouta ,Ziguinchor.
3	5	4	11	2	2	3	3	8	41	8	4	5	3	4	6	2018Vergnaud#1357	B. abortus bv3	34	64		BCCN#78-33	Cattle	1978	Toubacouta ,Ziguinchor.
3	5	4	11	2	2	3	3	8	41	8	4	5	3	5	6	2018Vergnaud#0321	B. abortus bv3	34	64		BCCN#76-315	Cattle	1976	Senegal
3	5	4	11	2	2	3	3	8	41	8	4	5	3	6	6	2018Vergnaud#0783	B. abortus bv3	34	64		BCCN#77-13	Cattle	1977	Inor-Diola ,Senegal-G.
3	5	4	11	2	2	3	3	8	41	8	4	5	3	4	3	2018Vergnaud#1227	B. abortus bv3	34	64		BCCN#77-32	Cattle	1977	Tambanaba ,Sedhiou N.
3	5	4	11	2	2	3	3	8	41	8	4	5	3	5	5	2018Vergnaud#0322	B. abortus bv3	34	64		BCCN#76-392	Cattle	1976	Bignona, Senegal
3	5	4	11	2	2	3	3	8	41	8	4	5	3	5	5	2018Vergnaud#0336	B. abortus bv3	34	64		BCCN#78-18	Cattle	1978	Adeane ,Ziguinchor, S.
3	5	4	11	2	2	3	3	8	41	8	4	5	3	5	5	2018Vergnaud#0794	B. abortus bv3	34	64		BCCN#79-157	Cattle	1979	Senegal
3	5	4	11	2	2	3	3	8	41	8	4	5	3	5	5	2018Vergnaud#1051	B. abortus bv3	34	64		BCCN#76-400	Cattle	1976	Bignona, Senegal
3	5	4	11	2	2	3	3	9	41	8	4	5	3	5	5	2018Vergnaud#0182	B. abortus bv3	34	316		BCCN#76-393	Cattle	1976	Bignona, Senegal
3	5	4	11	2	2	3	3	9	41	8	4	5	3	5	5	2018Vergnaud#0747	B. abortus bv3	34	316		BCCN#76-317	Cattle	1976	Senegal
3	5	4	11	2	2	3	3	8	41	8	4	5	3	5	8	2018Vergnaud#1235	B. abortus bv3	34	64		BCCN#79-169	Cattle	1979	Senegal
3	5	4	11	2	2	3	3	8	41	8	4	5	3	6	5	2018Vergnaud#1230	B. abortus bv3	34	64		BCCN#78-28	Cattle	1978	Bourofaye Baimouk ,Zig.
3	5	4	11	2	2	3	3	9	41	8	4	5	3	8	5	2018Vergnaud#1355	B. abortus bv3	34	316		BCCN#78-27	Cattle	1978	Bourofaye Baimouk ,Zig.
3	5	4	11	2	2	3	3	6	41	8	4	5	3	5	7	2018Vergnaud#0178	B. abortus bv1	34	63		BCCN#77-179	Cattle	1977	Ndoumbouthie ,Kaolack.
3	5	4	11	2	2	3	3	6	41	8	4	5	3	5	7	2018Vergnaud#0751	B. abortus bv3	34	63		BCCN#77-182	Cattle	1977	Ndoumbouthie ,Kaolack.
3	5	4	11	2	2	3	3	6	41	8	4	5	3	5	7	2018Vergnaud#1220	B. abortus bv3	34	63		BCCN#77-177	Cattle	1977	Ndoumbouthie ,Kaolack.
3	5	4	11	2	2	3	3	6	41	8	4	5	3	5	7	2018Vergnaud#1221	B. abortus bv3	34	63		BCCN#77-178	Cattle	1977	Ndoumbouthie ,Kaolack.
3	5	4	11	2	2	3	3	8	41	8	4	5	3	5	7	2018Vergnaud#0334	B. abortus bv3	34	64		BCCN#78-142	Cattle	1978	Sare Demba Assett ,Vel.
3	5	4	11	2	2	3	3	8	41	8	6	5	3	11	5	2006LeFleche#004	B. abortus bv3	34	64		REF Tulya	Human		Uganda
3	5	4	11	2	2	3	3	8	41	8	6	5	3	11	5	GCA_000740315	B. abortus	34	64	6	BFY			
3	5	4	11	2	2	3	3	8	41	8	6	5	3	11	5	SRR032625	B. abortus bv3	34	64	6	REF Tulya			
3	5	4	11	2	2	3	3	8	41	8	5	5	3	11	5	2009Her#003	B. abortus bv3	34	64		KRef03	Human		Uganda
3	5	4	11	2	2	3	3	8	41	8	7	5	3	11	5	GCA_000740155	B. abortus	34	64	6	BER			
3	5	4	11	2	2	3	3	8	43	8	6	5	3	11	5	2012Ferreira#136	B. abortus bv3	34	185		REF Tulya			
3	5	4	11	2	2	3	3	7	41	8	4	4	3	5	5	2018Vergnaud#0188	B. abortus bv3	34	317		BCCN#77-50	Cattle	1977	Tankon ,Senegal-Gamb.
3	5	4	11	2	2	3	3	7	41	8	4	4	3	5	7	2018Vergnaud#0191	B. abortus bv3	34	317		BCCN#77-65	Cattle	1977	Mandina ,Sedhiou Sud,.
3	5	4	11	2	2	3	3	7	41	8	4	4	3	5	6	2018Vergnaud#0335	B. abortus bv3	34	317		BCCN#78-16	Cattle	1978	Fanda ,Ziguinchor, Sen.
3	5	4	11	2	2	3	3	7	41	8	9	4	3	5	5	2018Vergnaud#0755	B. abortus bv3	34	317		BCCN#77-41	Cattle	1977	Sedhiou-Ville ,Sedhiou.
3	5	4	11	2	2	3	3	7	41	8	7	4	3	5	7	2018Vergnaud#0789	B. abortus bv3	34	317		BCCN#78-22	Cattle	1978	Fanghotte ,Ziguinchor, .
3	5	4	11	2	2	3	3	7	41	8	4	4	3	4	8	2018Vergnaud#0786	B. abortus bv3	34	317		BCCN#77-43	Cattle	1977	Dandon ,Sedhiou Nord,.
3	5	4	11	2	2	3	3	7	41	8	4	4	3	4	8	2018Vergnaud#1224	B. abortus bv3	34	317		BCCN#77-22	Cattle	1977	Dandon ,Sedhiou Nord,.
3	5	4	11	2	2	3	3	7	41	8	4	4	3	4	8	2018Vergnaud#1225	B. abortus bv3	34	317		BCCN#77-23	Cattle	1977	Dandon ,Sedhiou Nord,.
3	5	4	11	2	2	3	3	7	41	8	4	4	3	4	8	2018Vergnaud#1226	B. abortus bv3	34	317		BCCN#77-24	Cattle	1977	Dandon ,Sedhiou Nord,.
3	5	4	11	2	2	3	3	7	41	8	4	4	3	5	8	2018Vergnaud#0761	B. abortus bv3	34	317		BCCN#79-176	Cattle	1979	Senegal
3	5	4	11	2	2	3	3	7	41	8	4	3	3	7	9	2018Vergnaud#0189	B. abortus bv3	34	317		BCCN#77-53	Cattle	1977	Kenewal ,Sedhiou Sud,.
3	5	4	11	2	2	3	3	7	41	8	4	3	3	10	7	2018Vergnaud#0323	B. abortus bv3	34	317		BCCN#77-11	Cattle	1977	Bantancouyel ,Kolda, S.
3	5	4	11	2	2	3	3	7	41	8	4	5	3	3	7	2018Vergnaud#0203	B. abortus bv3	34	317		BCCN#78-68	Cattle	1978	Kedougou ,Tambacoun.
3	5	4	11	2	2	3	3	7	41	8	4	5	3	3	7	2018Vergnaud#0204	B. abortus bv3	34	317		BCCN#78-71	Cattle	1978	Siling ,Tambacounda, .
3	5	4	11	2	2	3	3	7	41	8	4	5	3	3	7	2018Vergnaud#0338	B. abortus bv3	34	317		BCCN#78-70	Cattle	1978	Kedougou ,Tambacoun.
3	5	4	11	2	2	3	3	7	41	8	4	5	3	3	7	2018Vergnaud#0339	B. abortus bv3	34	317		BCCN#78-72	Cattle	1978	Nianghe ,Tambacounda.
3	5	4	11	2	2	3	3	7	41	8	4	5	3	3	4	2018Vergnaud#0194	B. abortus bv3	34	317		BCCN#78-132	Cattle	1978	Sare Konco I ,Velingara.
3	5	4	11	2	2	3	3	7	41	8	4	5	3	3	4	2018Vergnaud#0196	B. abortus bv3	34	317		BCCN#78-138	Cattle	1978	Sare Boukagano ,Velin.
3	5	4	11	2	2	3	3	7	41	8	4	5	3	3	4	2018Vergnaud#1060	B. abortus bv3	34	317		BCCN#78-137	Cattle	1978	Sare Boukagano ,Velin.
3	5	4	11	2	2	3	3	7	41	8	4	5	3	5	5	2018Vergnaud#0330	B. abortus bv3	34	317		BCCN#79-180	Cattle	1979	Senegal
3	5	4	11	2	2	3	3	7	41	8	4	5	3	5	5	2018Vergnaud#1062	B. abortus bv3	34	317		BCCN#79-161	Cattle	1979	Senegal
3	5	4	11	2	2	3	3	7	41	8	4	5	3	5	5	2018Vergnaud#1231	B. abortus bv3	34	317		BCCN#78-29	Cattle	1978	Bourofaye Baimouk ,Zig.
3	5	4	11	2	2	3	3	7	41	8	4	5	3	5	5	2018Vergnaud#1354	B. abortus bv3	34	317		BCCN#78-26	Cattle	1978	Bourofaye Baimouk ,Zig.
3	5	4	11	2	2	3	3	7	41	8	4	5	3	3	5	2018Vergnaud#0325	B. abortus bv3	34	317		BCCN#77-197	Cattle	1977	Keur Ilo ,Kaolack, Sen.
3	5	4	11	2	2	3	3	7	41	8	4	5	3	3	5	2018Vergnaud#0326	B. abortus bv3	34	317		BCCN#77-198	Cattle	1977	Medina Sabakh ,Kaolac.
3	5	4	11	2	2	3	3	7	41	8	4	5	3	3	5	2018Vergnaud#0341	B. abortus bv3	34	317		BCCN#79-99	Cattle	1979	Senegal
3	5	4	11	2	2	3	3	7	41	8	4	5	3	3	5	2018Vergnaud#0784	B. abortus bv3	34	317		BCCN#77-204	Cattle	1977	Keur Ilo ,Kaolack, Sen.
3	5	4	11	2	2	3	3	7	41	8	4	5	3	3	5	2018Vergnaud#1222	B. abortus bv3	34	317		BCCN#77-193	Cattle	1977	Kantorali ,Kaolack, Sen.
3	5	4	11	2	2	3	3	7	41	8	4	5	3	3	5	2018Vergnaud#1223	B. abortus bv3	34	317		BCCN#77-194	Cattle	1977	Kantorali ,Kaolack, Sen.
3	5	4	11	2	2	3	3	7	41	8	4	5	3	3	5	2018Vergnaud#1237	B. abortus bv3	34	317		BCCN#79-174	Cattle	1979	Senegal
3	5	4	11	2	2	3	3	7	41	8	4	5	3	3	5	2018Vergnaud#1364	B. abortus bv3	34	317		BCCN#79-159	Cattle	1979	Senegal
3	5	4	11	2	2	3	3	7	41	8	4	7	3	5	5	2018Vergnaud#1346	B. abortus bv3	34	317		BCCN#77-38	Cattle	1977	Marandan ,Sedhiou Nor.
3	5	4	11	2	2	3	3	7	41	8	4	7	3	5	5	2018Vergnaud#1347	B. abortus bv3	34	317		BCCN#77-39	Cattle	1977	Marandan ,Sedhiou Nor.
3	5	4	11	2	2	3	3	7	41	8	4	7	3	3	5	2018Vergnaud#0787	B. abortus bv3	34	317		BCCN#77-44	Cattle	1977	Sitaba ,Sedhiou Nord, .
3	5	4	11	2	2	3	3	7	41	8	4	7	3	3	5	2018Vergnaud#0798	B. abortus bv3	34	317		BCCN#79-171	Cattle	1979	Senegal
3	5	4	11	2	2	3	3	7	41	8	4	7	3	3	5	2018Vergnaud#1343	B. abortus bv3	34	317		BCCN#77-26	Cattle	1977	Sitaba ,Sedhiou Nord, .
3	5	4	11	2	2	2	3	7	41	8	4	5	3	3	7	2018Vergnaud#0337	B. abortus bv3	137	310		BCCN#78-69	Cattle	1978	Kedougou ,Tambacoun.
3	5	4	11	2	2	3	3	7	41	8	5	5	3	4	5	2018Vergnaud#0328	B. abortus bv3	34	317		BCCN#78-17	Cattle	1978	Sindone ,Ziguinchor, S.
3	5	4	11	2	2	3	3	7	41	8	5	5	3	5	5	2018Vergnaud#0762	B. abortus bv3	34	317		BCCN#79-178	Cattle	1979	Senegal
3	5	4	11	2	2	3	3	7	41	8	6	5	3	12	5	2011Muendo#11	B. abortus bv3	34	317		11-KEBa2	Cattle	2009	Central, Kenya
3	5	4	11	2	2	3	3	7	41	8	6	5	3	12	5	2011Muendo#14	B. abortus bv3	34	317		14-KEBa2	Cattle	2009	Central, Kenya
3	5	4	11	2	2	3	3	7	41	8	6	5	3	12	5	2011Muendo#15	B. abortus bv3	34	317		15-KEBa2	Cattle	2009	Central, Kenya
3	5	4	11	2	2	3	3	7	41	8	6	5	3	9	4	2018Vergnaud#0274	B. abortus bv3	34	317		BCCN#83-227	Cattle	1983	Rwanda
3	5	4	11	2	2	3	3	7	41	8	6	5	3	10	4	2018Vergnaud#0307	B. abortus bv3	34	317		BCCN#82-21	Cattle	1982	Butare ,Bugesera, Rwa.
3	5	4	11	2	2	3	3	7	41	8	4	5	3	6	4	2018Vergnaud#0268	B. abortus bv3	34	317		BCCN#83-14	Cattle	1983	Rwanda
3	5	4	11	2	2	3	3	7	41	8	7	5	3	6	4	2018Vergnaud#0308	B. abortus bv3	34	317		BCCN#82-27	Human	1982	Rwanda
3	5	4	11	2	2	3	3	6	41	8	4	5	6	5	6	2018Vergnaud#0389	B. abortus bv3	34	63		BCCN#79-98	Cattle	1979	Senegal
3	5	4	11	2	2	3	3	8	41	8	7	9	3	5	3	2018Vergnaud#0265	B. abortus bv3	34	64		BCCN#80-211	Cattle	1980	Togo
3	5	4	11	2	2	3	3	8	41	8	7	4	3	5	3	2018Vergnaud#1332	B. abortus bv3	34	64		BCCN#80-208	Cattle	1980	Togo
3	5	4	11	2	2	3	3	8	41	8	8	10	3	5	3	2018Vergnaud#0763	B. abortus bv3	34	64		BCCN#80-194	Cattle	1980	Togo
3	5	4	11	2	2	3	3	8	41	8	8	10	3	5	3	2018Vergnaud#1336	B. abortus bv3	34	64		BCCN#79-145	Cattle	1979	Togo
3	5	4	11	2	2	3	3	8	41	8	7	10	3	5	3	2018Vergnaud#0263	B. abortus bv3	34	64		BCCN#80-192	Cattle	1980	Togo
3	5	4	11	2	2	3	3	8	41	8	7	10	3	5	3	2018Vergnaud#0264	B. abortus bv3	34	64		BCCN#80-193	Cattle	1980	Togo
3	5	4	11	2	2	3	3	8	41	8	7	10	3	5	3	2018Vergnaud#0266	B. abortus bv3	34	64		BCCN#79-142	Cattle	1979	Togo
3	5	4	11	2	2	3	3	8	41	8	7	10	3	5	3	2018Vergnaud#0267	B. abortus bv3	34	64		BCCN#79-151	Cattle	1979	Togo

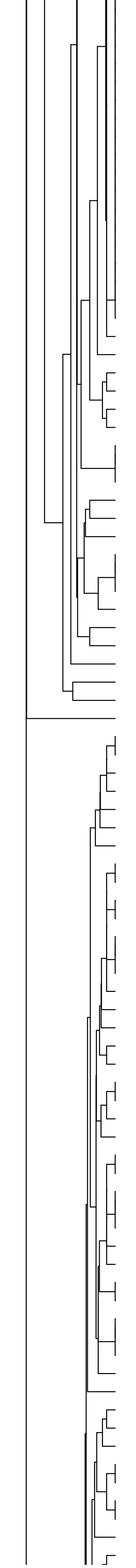

3	5	4	11	2	2	3	3	8	41	8	7	10	3	5	3	2018Vergnaud#0764	B. abortus bv3	34	64	BCCN#80-195	Cattle	1980	Togo
3	5	4	11	2	2	3	3	8	41	8	7	10	3	5	3	2018Vergnaud#1064	B. abortus bv3	34	64	BCCN#80-196	Cattle	1980	Togo
3	5	4	11	2	2	3	3	8	41	8	7	10	3	5	3	2018Vergnaud#1065	B. abortus bv3	34	64	BCCN#80-197	Cattle	1980	Togo
3	5	4	11	2	2	3	3	8	41	8	7	10	3	5	3	2018Vergnaud#1238	B. abortus bv3	34	64	BCCN#80-198	Cattle	1980	Togo
3	5	4	11	2	2	3	3	8	41	8	7	10	3	5	3	2018Vergnaud#1239	B. abortus bv3	34	64	BCCN#80-199	Cattle	1980	Togo
3	5	4	11	2	2	3	3	8	41	8	7	10	3	5	3	2018Vergnaud#1325	B. abortus bv3	34	64	BCCN#80-200	Cattle	1980	Togo
3	5	4	11	2	2	3	3	8	41	8	7	10	3	5	3	2018Vergnaud#1326	B. abortus bv3	34	64	BCCN#80-201	Cattle	1980	Togo
3	5	4	11	2	2	3	3	8	41	8	7	10	3	5	3	2018Vergnaud#1327	B. abortus bv3	34	64	BCCN#80-202	Cattle	1980	Togo
3	5	4	11	2	2	3	3	8	41	8	7	10	3	5	3	2018Vergnaud#1328	B. abortus bv3	34	64	BCCN#80-203	Cattle	1980	Togo
3	5	4	11	2	2	3	3	8	41	8	7	10	3	5	3	2018Vergnaud#1329	B. abortus bv3	34	64	BCCN#80-205	Cattle	1980	Togo
3	5	4	11	2	2	3	3	8	41	8	7	10	3	5	3	2018Vergnaud#1330	B. abortus bv3	34	64	BCCN#80-206	Cattle	1980	Togo
3	5	4	11	2	2	3	3	8	41	8	7	10	3	5	3	2018Vergnaud#1331	B. abortus bv3	34	64	BCCN#80-207	Cattle	1980	Togo
3	5	4	11	2	2	3	3	8	41	8	7	10	3	5	3	2018Vergnaud#1333	B. abortus bv3	34	64	BCCN#80-209	Cattle	1980	Togo
3	5	4	11	2	2	3	3	8	41	8	7	10	3	5	3	2018Vergnaud#1334	B. abortus bv3	34	64	BCCN#80-210	Cattle	1980	Togo
3	5	4	11	2	2	3	3	8	41	8	7	10	3	5	3	2018Vergnaud#1337	B. abortus bv3	34	64	BCCN#79-146	Cattle	1979	Togo
3	5	4	11	2	2	3	3	8	41	8	7	10	3	5	3	2018Vergnaud#1338	B. abortus bv3	34	64	BCCN#79-147	Cattle	1979	Togo
3	5	4	11	2	2	3	3	8	41	8	7	10	3	5	3	2018Vergnaud#1339	B. abortus bv3	34	64	BCCN#79-148	Cattle	1979	Togo
3	5	4	11	2	2	3	3	8	41	8	7	10	3	5	3	2018Vergnaud#1340	B. abortus bv3	34	64	BCCN#79-149	Cattle	1979	Togo
3	5	4	11	2	2	3	3	8	41	8	6	10	3	5	3	2018Vergnaud#1335	B. abortus bv3	34	64	BCCN#79-144	Cattle	1979	Togo
3	5	4	11	2	2	3	3	8	41	8	7	5	7	5	3	2018Vergnaud#0311	B. abortus bv3	34	64	BCCN#79-150	Cattle	1979	Togo
3	5	4	11	2	2	3	3	8	41	8	6	7	3	8	3	2018Vergnaud#1056	B. abortus bv3	34	64	BCCN#77-187	Cattle	1977	Djilor ,Kaolack, Senegal
3	5	4	11	2	2	3	3	8	41	8	6	7	3	6	3	2018Vergnaud#1341	B. abortus bv3	34	64	BCCN#77-188	Cattle	1977	Djilor ,Kaolack, Senegal
3	5	4	11	2	2	3	3	8	41	8	6	7	3	7	3	2018Vergnaud#0754	B. abortus bv3	34	64	BCCN#77-203	Cattle	1977	Djilor ,Kaolack, Senegal
3	5	4	11	2	2	3	3	8	41	8	5	7	3	7	3	2018Vergnaud#0785	B. abortus bv3	34	64	BCCN#77-205	Cattle	1977	Keur Dengre ,Kaolack,.
3	5	3	11	2	2	3	3	8	41	8	6	2	3	5	5	2018Vergnaud#0246	B. abortus bv3	134	313	BCCN#83-20	Cattle	1983	Niger
3	5	3	11	2	2	3	3	8	41	8	6	2	3	5	5	2018Vergnaud#0247	B. abortus bv3	134	313	BCCN#83-233	Cattle	1983	Niger
3	5	3	11	2	2	3	3	8	41	8	6	2	3	5	5	2018Vergnaud#0248	B. abortus bv3	134	313	BCCN#83-234	Cattle	1983	Niger
3	5	4	11	2	2	3	3	6	41	8	6	4	3	8	4	2006LeFleche#124	B. abortus bv6	34	63	BfR 7	Cattle		Tchad
3	5	4	11	2	2	3	3	6	41	8	5	3	3	11	4	2018Vergnaud#0237	B. abortus bv3	34	63	BCCN#90-45	Zebu	1990	Cameroon
3	5	4	11	2	2	3	3	6	41	8	7	4	3	7	9	2018Vergnaud#0236	B. abortus bv3	34	63	BCCN#89-116	Cattle	1989	Cameroon
3	5	4	11	2	2	3	3	6	41	8	6	8	3	7	7	2006LeFleche#111	B. abortus bv3	34	63	BCCN#93-26	Dromed.	1993	Sudan
3	5	4	11	2	2	3	3	6	41	8	6	8	3	7	7	2018Vergnaud#0345	B. abortus bv3	34	63	BCCN#93-25	Dromed.	1993	Sudan
3	5	4	11	2	2	3	3	6	41	8	6	8	3	7	7	2018Vergnaud#0346	B. abortus bv3	34	63	BCCN#93-27	Dromed.	1993	Sudan
3	5	4	11	2	2	3	3	8	41	8	6	8	3	5	7	2018Vergnaud#0179	B. abortus bv3	34	64	BCCN#03-33	Human	2003	Mauritania
3	5	4	11	2	1	3	3	6	41	8	5	4	3	6	5	2006LeFleche#125	B. abortus bv6	35	62	BfR 8	Cattle		Tchad
3	5	4	11	2	2	3	3	6	41	8	4	13	3	6	5	2018Vergnaud#0238	B. abortus bv3	34	63	BCCN#90-46	Zebu	1990	Cameroon
3	5	4	11	2	2	3	3	7	41	8	6	3	9	6	8	2018Vergnaud#1344	B. abortus bv3	34	317	BCCN#77-28	Cattle	1977	Brosso ,Senegal-Gambi.
3	5	4	11	3	2	2	3	7	41	8	4	7	8	3	5	2018Vergnaud#0186	B. abortus bv3	142	312	BCCN#77-19	Cattle	1977	Diatouma ,Sedhiou Nord.
3	5	4	11	3	2	2	3	9	41	8	4	6	3	5	6	2018Vergnaud#0187	B. abortus bv3	142	311	BCCN#77-27	Cattle	1977	Ndiama ,Sedhiou Nord,.
4	5	3	11	2	2	6	3	4	41	10	8	5	4	8	4	2013Minharro#028	B. abortus bv6	166	309	95	Cattle	2007	Presidente Kennedy, 7.
3	6	3	14	1	1	3	3	7	43	8	5	4	7	6	3	2018Vergnaud#0725	B. melitensis bv3	49	87	BCCN#99-79	Ovine	1999	Sicile, Italy
3	6	3	14	1	1	3	3	7	43	8	5	4	7	6	3	2018Vergnaud#1252	B. melitensis bv3	49	87	BCCN#99-72	Ovine	1999	Sicile, Italy
3	6	3	14	1	1	3	3	7	43	8	5	4	10	6	3	2018Vergnaud#1016	B. melitensis bv3	49	87	BCCN#96-91	Ovine	1996	Sicile, Italy
3	6	3	14	1	1	3	3	7	43	8	5	4	12	6	3	2018Vergnaud#0696	B. melitensis bv3	49	87	BCCN#99-9	Ovine	1999	Sicile, Italy
3	6	3	14	1	1	3	3	7	43	8	5	6	10	6	3	2018Vergnaud#0653	B. melitensis bv3	49	87	BCCN#96-85	Ovine	1996	Sicile, Italy
3	6	3	14	1	1	3	3	7	43	8	5	7	12	6	3	2017Hanot-Mamb.	B. melitensis bv3	49	87	L3/147	Human	2005	Belgium
3	6	3	14	1	1	3	3	7	43	8	5	4	9	4	3	2018Vergnaud#0984	B. melitensis bv3	49	87	BCCN#99-12	Ovine	1999	Sicile, Italy
3	6	3	14	1	1	3	3	7	43	8	6	4	10	6	3	2018Vergnaud#1246	B. melitensis bv3	49	87	BCCN#97-22	Ovine	1997	Sicile, Italy
3	6	3	14	1	1	3	3	7	43	8	6	4	10	6	3	2018Vergnaud#1247	B. melitensis bv3	49	87	BCCN#97-23	Ovine	1997	Sicile, Italy
3	6	3	14	1	1	3	3	7	43	8	6	4	7	6	3	2018Vergnaud#0687	B. melitensis bv3	49	87	BCCN#99-61	Cattle	1999	Sicile, Italy
3	6	3	14	1	1	3	3	7	43	8	6	4	7	6	3	2018Vergnaud#0746	B. melitensis bv3	49	87	BCCN#99-59	Cattle	1999	Sicile, Italy
3	6	3	14	1	1	3	3	7	43	8	6	4	11	6	3	2018Vergnaud#0685	B. melitensis bv3	49	87	BCCN#99-6	Ovine	1999	Sicile, Italy
3	6	3	14	1	1	3	3	7	43	8	6	4	11	6	3	2018Vergnaud#0693	B. melitensis bv3	49	87	BCCN#99-8	Ovine	1999	Sicile, Italy
3	6	3	14	1	1	3	3	7	43	8	6	4	11	6	3	2018Vergnaud#1216	B. melitensis bv3	49	87	BCCN#99-47	Ovine	1999	Sicile, Italy
3	6	3	14	1	1	3	3	7	43	8	6	4	6	6	3	2018Vergnaud#0857	B. melitensis bv3	49	87	BCCN#99-58	Ovine	1999	Sicile, Italy
3	6	3	14	1	1	3	3	7	43	8	6	9	11	6	3	2018Vergnaud#0658	B. melitensis bv3	49	87	BCCN#97-17	Ovine	1997	Sicile, Italy
3	6	3	14	1	1	3	3	7	43	8	10	4	11	6	3	2013Garofolo_7556	B. melitensis bv3	49	87	7556	Ovine	2011	Taviano,Italy
3	6	3	13	1	1	3	3	7	43	8	6	4	11	6	3	2007Marianelli#015	B. melitensis bv3	88	154	GA8	Human	2005	Catania, Italy
3	6	3	13	1	1	3	3	7	43	8	6	4	10	6	3	2018Vergnaud#0655	B. melitensis bv3	88	154	BCCN#96-87	Ovine	1996	Sicile, Italy
3	6	3	14	1	1	3	3	7	43	8	6	4	7	8	3	2007AlDahouk#080	B. melitensis bv2	49	87	BfR 56	Human	2002	Bern, Switzerland
3	6	3	14	1	1	3	3	7	43	8	6	4	7	8	3	2013Garofolo_11.	B. melitensis bv3	49	87	11793	Caprine	2011	Caltanissetta,Italy
3	6	3	14	1	1	3	3	7	43	8	6	4	11	8	3	2018Vergnaud#0678	B. melitensis bv3	49	87	BCCN#99-35	Ovine	1999	Sicile, Italy
3	6	3	14	1	1	3	3	7	43	8	6	5	11	8	3	2018Vergnaud#0689	B. melitensis bv3	49	87	BCCN#99-67	Ovine	1999	Sicile, Italy
3	6	3	14	1	1	3	3	7	43	8	6	4	10	7	3	2018Vergnaud#0684	B. melitensis bv3	49	87	BCCN#99-54	Ovine	1999	Sicile, Italy
3	6	3	14	1	1	3	3	7	43	8	6	4	10	7	3	2018Vergnaud#0856	B. melitensis bv3	49	87	BCCN#99-57	Cattle	1999	Sicile, Italy
3	6	3	14	1	1	3	3	7	43	8	6	4	6	7	3	2007Marianelli#006	B. melitensis bv3	49	87	BL9	Human	2005	Catania, Italy
3	6	3	14	1	1	3	3	7	43	8	6	4	6	7	3	2013Garofolo_13.	B. melitensis bv3	49	87	13537	Ovine	2011	Sperlingua,Italy
3	6	3	14	1	1	3	3	7	43	8	6	4	6	7	3	2018Vergnaud#1214	B. melitensis bv3	49	87	BCCN#97-89	Ovine	1997	Sicile, Italy
3	6	3	14	1	1	3	3	7	43	8	6	4	4	7	3	2018Vergnaud#0423	B. melitensis bv3	49	87	BCCN#97-62	Ovine	1997	Sicile, Italy
3	6	3	14	1	1	3	3	7	43	8	6	4	5	7	3	2018Vergnaud#1245	B. melitensis bv3	49	87	BCCN#96-54	Ovine	1996	Sicile, Italy
3	6	3	14	1	1	3	3	7	43	8	6	4	4	4	3	2013Garofolo_585	B. melitensis bv3	49	87	585	Cattle	2011	Apricena,Italy
3	6	3	14	1	1	3	3	7	43	8	6	4	4	4	3	2013Garofolo_6842	B. melitensis bv3	49	87	6842	Caprine	2011	Massafra,Italy
3	6	3	14	1	1	3	3	7	43	8	6	4	8	11	3	2013Garofolo_11.	B. melitensis bv3	49	87	11782	Caprine	2011	Aci bonaccorsi,Italy
3	6	3	14	1	1	3	3	7	43	8	6	4	8	11	3	2013Garofolo_13.	B. melitensis bv3	49	87	13535	Ovine	2011	Tremestieri Etneo,Italy
3	6	3	14	1	1	3	3	7	43	8	6	4	8	11	3	2013Garofolo_19.	B. melitensis bv3	49	87	19428	Ovine	2011	Tremestieri Etneo,Italy
3	6	3	14	1	1	3	3	7	43	8	6	4	9	10	3	2007AlDahouk#081	B. melitensis bv2	49	87	BfR 57	Human	2002	Bern, Switzerland
3	6	3	14	1	1	3	3	7	43	8	6	8	16	2	3	2013Garofolo_6840	B. melitensis bv3	49	87	6840	Ovine	2011	Massafra,Italy
3	6	3	14	1	1	3	3	7	43	8	8	4	6	5	3	2018Vergnaud#0424	B. melitensis bv3	49	87	BCCN#97-63	Cattle	1997	Sicile, Italy
3	6	3	14	1	1	3	3	7	43	8	5	4	6	5	3	2018Vergnaud#1286	B. melitensis bv3	49	87	BCCN#97-66	Ovine	1997	Sicile, Italy
3	6	3	14	1	1	3	3	7	43	8	5	4	7	5	3	2018Vergnaud#0426	B. melitensis bv3	49	87	BCCN#98-50	Human	1998	Buenos Aires ,Argentina
3	6	3	14	1	1	3	3	7	43	8	7	4	7	5	3	2018Vergnaud#0652	B. melitensis bv3	49	87	BCCN#96-84	Cattle	1996	Sicile, Italy
3	6	3	14	1	1	3	3	7	43	8	7	4	7	5	3	2018Vergnaud#0654	B. melitensis bv3	49	87	BCCN#96-86	Cattle	1996	Sicile, Italy
3	6	3	13	1	1	3	3	7	43	8	7	4	7	5	3	2013Garofolo_11.	B. melitensis bv3	88	154	11802	Caprine	2011	Barcellona Pozzo di Go.
3	6	3	13	1	1	3	3	7	43	8	7	4	7	5	3	2013Garofolo_11.	B. melitensis bv3	88	154	11842	Ovine	2011	Barcellona Pozzo di Go.
3	6	3	14	1	1	3	3	7	43	8	8	4	7	9	3	2007Marianelli#011	B. melitensis bv3	49	87	CV5	Human	2005	Catania, Italy
3	6	3	14	1	1	3	3	7	43	8	9	7	7	5	3	2013Garofolo_1164	B. melitensis bv3	49	87	1164	Ovine	2011	Chiaravalle Centrale,Italy

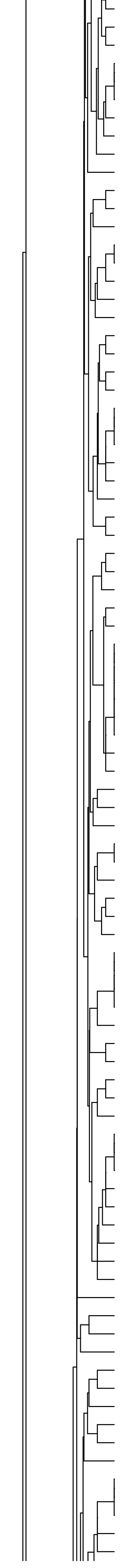

3	6	3	14	1	1	3	3	7	43	8	9	6	7	5	3	2018Vergnaud#0657	B. melitensis bv3	49	87		BCCN#97-15	Ovine	1997	Sicile, Italy
3	6	3	14	1	1	3	3	7	43	8	6	6	7	5	3	2007AlDahouk#093	B. melitensis bv3	49	87		BfR 70	Human	1999	Italy
3	6	3	14	1	1	3	3	7	43	8	6	7	7	5	3	2007Marianelli#004	B. melitensis bv3	49	87		CM10	Human	2005	Catania, Italy
3	6	3	14	1	1	3	3	7	43	8	8	7	7	5	3	2013Garofolo_1169	B. melitensis bv3	49	87		1169	Caprine	2011	Pizzoni,Italy
3	6	3	14	1	1	3	3	7	43	8	8	7	7	5	3	2013Garofolo_1171	B. melitensis bv3	49	87		1171	Ovine	2011	Briatico,Italy
3	6	3	14	1	1	3	3	7	43	8	8	7	7	5	3	2013Garofolo_3271	B. melitensis bv3	49	87		3271	Ovine	2011	Briatico,Italy
3	6	3	14	1	1	3	3	7	43	8	8	7	10	5	3	2007AlDahouk#082	B. melitensis bv2	49	87		BfR 58	Human	2004	Italy
3	6	3	14	1	1	3	3	7	43	8	8	7	7	8	3	2013Garofolo_15.	B. melitensis bv3	49	87		15272	Cattle	2012	Foggia,Italy
3	6	3	14	1	1	3	3	7	43	8	6	7	7	7	3	2018Vergnaud#0651	B. melitensis bv3	49	87		BCCN#96-75	Cattle	1996	Sicile, Italy
3	6	3	14	1	1	3	3	7	43	8	5	9	5	5	3	2018Vergnaud#0663	B. melitensis bv3	49	87		BCCN#97-64	Ovine	1997	Sicile, Italy
3	6	3	14	1	1	3	3	7	43	8	9	7	8	5	3	2013Garofolo_18.	B. melitensis bv3	49	87		18942	Ovine	2011	Chiaravalle Centrale,Italy
3	6	3	14	1	1	3	3	7	43	8	9	5	8	5	3	2018Vergnaud#0686	B. melitensis bv3	49	87		BCCN#99-60	Cattle	1999	Sicile, Italy
3	6	3	14	1	1	3	3	7	43	8	9	5	6	3	3	2018Vergnaud#1020	B. melitensis bv3	49	87		BCCN#99-64	Ovine	1999	Sicile, Italy
3	6	3	14	1	1	3	3	7	43	8	10	5	11	5	3	2018Vergnaud#1096	B. melitensis bv3	49	87		BCCN#99-5	Ovine	1999	Sicile, Italy
3	6	3	14	1	1	3	3	7	43	8	10	5	11	5	3	2018Vergnaud#1100	B. melitensis bv3	49	87		BCCN#99-7	Ovine	1999	Sicile, Italy
3	6	3	14	1	1	3	3	7	43	8	8	5	11	5	3	2018Vergnaud#1089	B. melitensis bv3	49	87		BCCN#96-52	Ovine	1996	Sicile, Italy
3	6	3	14	1	1	3	3	7	43	8	5	5	11	4	3	2013Garofolo_13.	B. melitensis bv3	49	87		13552	Caprine	2011	Mandanici,Italy
3	6	3	14	1	1	3	3	7	43	8	11	5	12	5	3	2018Vergnaud#0683	B. melitensis bv3	49	87		BCCN#99-53	Cattle	1999	Sicile, Italy
3	6	3	14	1	1	3	3	7	43	8	10	5	10	6	3	2018Vergnaud#0692	B. melitensis bv3	49	87		BCCN#99-69	Cattle	1999	Sicile, Italy
3	6	3	14	1	1	3	3	7	43	8	6	5	10	6	3	2018Vergnaud#0854	B. melitensis bv3	49	87		BCCN#96-94	Ovine	1996	Sicile, Italy
3	6	3	14	1	1	3	3	7	43	8	5	5	8	6	3	2013Garofolo_4500	B. melitensis bv3	49	87		4500	Ovine	2011	Messina,Italy
3	6	3	14	1	1	3	3	7	43	8	6	5	8	6	3	2018Vergnaud#0853	B. melitensis bv3	49	87		BCCN#96-93	Cattle	1996	Sicile, Italy
3	6	3	14	1	1	3	3	7	43	8	7	5	6	6	3	2018Vergnaud#0676	B. melitensis bv3	49	87		BCCN#99-26	Ovine	1999	Sicile, Italy
3	6	3	14	1	1	3	3	7	43	8	7	5	6	6	3	2018Vergnaud#0677	B. melitensis bv3	49	87		BCCN#99-27	Cattle	1999	Sicile, Italy
3	6	3	14	1	1	3	3	7	43	8	7	5	6	6	3	2018Vergnaud#1090	B. melitensis bv3	49	87		BCCN#96-96	Cattle	1996	Sicile, Italy
3	6	3	14	1	1	3	3	7	43	8	7	5	8	6	3	2013Garofolo_11.	B. melitensis bv3	49	87		11798	Ovine	2011	Messina,Italy
3	6	3	14	1	1	3	3	7	43	8	7	5	9	6	3	2013Garofolo_4497	B. melitensis bv3	49	87		4497	Ovine	2011	Messina,Italy
3	6	3	14	1	1	3	3	7	43	8	4	5	4	6	3	2018Vergnaud#0985	B. melitensis bv3	49	87		BCCN#99-13	Ovine	1999	Sicile, Italy
3	6	3	14	1	1	3	3	7	36	8	8	5	7	6	3	2018Vergnaud#0415	B. melitensis bv3	49	306		BCCN#95-32	Ovine	1995	Sicile, Italy
3	6	3	14	1	1	3	3	7	43	8	8	5	7	6	3	2018Vergnaud#0646	B. melitensis bv3	49	87		BCCN#96-47	Cattle	1996	Sicile, Italy
3	6	3	14	1	1	3	3	7	43	8	5	5	8	8	3	2018Vergnaud#0434	B. melitensis bv3	49	87		BCCN#95-35	Cattle	1995	Sicile, Italy
3	6	3	14	1	1	3	3	7	43	8	5	6	8	8	3	2018Vergnaud#0849	B. melitensis bv3	49	87		BCCN#96-55	Ovine	1996	Sicile, Italy
3	6	3	14	1	1	3	3	7	43	8	5	6	3	8	3	2018Vergnaud#0644	B. melitensis bv3	49	87		BCCN#96-42	Cattle	1996	Sicile, Italy
3	6	3	14	1	1	3	3	7	43	8	4	6	10	8	3	2013Garofolo_18.	B. melitensis bv3	49	87		18945	Caprine	2011	Cleto,Italy
3	6	3	14	1	1	3	3	7	43	8	4	6	12	8	3	2018Vergnaud#1217	B. melitensis bv3	49	87		BCCN#99-48	Ovine	1999	Sicile, Italy
3	6	3	14	1	1	3	3	7	43	8	4	6	11	8	3	2013Garofolo_12.	B. melitensis bv3	49	87		12372	Ovine	2011	Zungri,Italy
3	6	3	14	1	1	3	3	7	43	8	4	6	11	8	3	2013Garofolo_12.	B. melitensis bv3	49	87		12373	Ovine	2011	Rombiolo,Italy
3	6	3	14	1	1	3	3	7	43	8	4	6	11	8	3	2013Garofolo_12.	B. melitensis bv3	49	87		12849	Ovine	2011	Mileto,Italy
3	6	3	14	1	1	3	3	7	43	8	4	6	11	8	3	2013Garofolo_17.	B. melitensis bv3	49	87		17475	Ovine	2011	Zungri,Italy
3	6	3	14	1	1	3	3	7	43	8	4	6	11	8	3	2013Garofolo_6061	B. melitensis bv3	49	87		6061	Ovine	2011	Rombiolo,Italy
3	6	3	14	1	1	3	3	7	43	8	4	6	11	8	3	2013Garofolo_9065	B. melitensis bv3	49	87		9065	Ovine	2011	Lamezia Terme,Italy
3	6	3	14	1	1	3	3	7	43	8	4	6	11	9	3	2013Garofolo_12.	B. melitensis bv3	49	87		12376	Cattle	2011	Zungri,Italy
3	6	3	14	1	1	3	3	7	43	8	4	6	11	7	3	2013Garofolo_12.	B. melitensis bv3	49	87		12841	Ovine	2011	Gerocarne,Italy
3	6	3	14	1	1	3	3	7	43	8	8	6	9	8	3	2013Garofolo_2299	B. melitensis bv3	49	87		2299	Ovine	2011	Ugento,Italy
3	6	3	14	1	1	3	3	7	43	8	9	13	9	8	3	2018Vergnaud#0679	B. melitensis bv3	49	87		BCCN#99-37	Caprine	1999	Sicile, Italy
3	6	3	14	1	1	3	3	7	43	8	9	11	7	8	3	2018Vergnaud#0680	B. melitensis bv3	49	87		BCCN#99-38	Ovine	1999	Sicile, Italy
3	6	3	14	1	1	3	3	7	43	8	8	6	5	6	3	2018Vergnaud#1240	B. melitensis bv3	49	87		BCCN#90-180	Human	1990	Moselle, France
3	6	3	14	1	1	3	3	7	43	8	8	6	5	6	3	SRR4436621	B. melitensis	49	87	88	BwIM_ITA_45	Human	2017	Italy
3	6	3	14	1	1	3	3	7	45	8	4	6	5	6	3	2007AlDahouk#097	B. melitensis bv3	49	89		BfR 74	Human	2000	Italy
3	6	3	14	1	1	3	3	7	43	8	4	6	7	6	3	2013Garofolo_13.	B. melitensis bv3	49	87		13541	Caprine	2011	Caltagirone,Italy
3	6	3	14	1	1	3	3	7	43	8	4	6	9	6	3	2018Vergnaud#0675	B. melitensis bv3	49	87		BCCN#99-21	Cattle	1999	Sicile, Italy
3	6	3	14	1	1	3	3	7	43	8	4	4	9	6	3	2018Vergnaud#0681	B. melitensis bv3	49	87		BCCN#99-39	Caprine	1999	Sicile, Italy
3	6	3	14	1	1	3	3	7	43	8	10	6	13	5	3	2018Vergnaud#0660	B. melitensis bv3	49	87		BCCN#97-20	Ovine	1997	Sicile, Italy
3	6	3	14	1	1	3	3	7	43	8	10	6	13	5	3	2018Vergnaud#1248	B. melitensis bv3	49	87		BCCN#97-24	Ovine	1997	Sicile, Italy
3	6	3	14	1	1	3	3	7	43	8	10	6	13	5	3	2018Vergnaud#1289	B. melitensis bv3	49	87		BCCN#97-74	Ovine	1997	Sicile, Italy
3	6	3	14	1	1	3	3	7	43	8	10	6	13	5	3	2018Vergnaud#1404	B. melitensis bv3	49	87		BCCN#97-78	Ovine	1997	Sicile, Italy
3	6	3	14	1	1	3	3	7	43	8	7	6	13	6	3	2018Vergnaud#1290	B. melitensis bv3	49	87		BCCN#97-65	Ovine	1997	Sicile, Italy
3	6	3	14	1	1	3	3	7	43	8	7	6	8	4	3	2006LeFleche#092	B. melitensis bv3	49	87		BCCN#96-56	Ovine	1996	Sicilia, Italia
3	6	3	14	1	1	3	3	7	43	8	8	6	8	4	3	2018Vergnaud#1285	B. melitensis bv3	49	87		BCCN#97-32	Ovine	1997	Sicile, Italy
3	6	3	14	1	1	3	3	7	43	8	7	6	9	7	3	2007Marianelli#009	B. melitensis bv3	49	87		BC6	Human	2005	Catania, Italy
3	6	3	14	1	1	3	3	7	43	8	7	6	10	7	3	2018Vergnaud#1284	B. melitensis bv3	49	87		BCCN#97-31	Ovine	1997	Sicile, Italy
3	6	3	14	1	1	3	3	8	43	8	7	6	3	7	3	2018Vergnaud#1398	B. melitensis bv3	49	162		BCCN#97-79	Ovine	1997	Sicile, Italy
3	6	3	14	1	1	3	3	7	43	8	6	6	4	7	3	2018Vergnaud#0664	B. melitensis bv3	49	87		BCCN#97-70	Ovine	1997	Sicile, Italy
3	6	3	14	1	1	3	3	7	43	8	6	6	4	7	3	2018Vergnaud#1288	B. melitensis bv3	49	87		BCCN#97-71	Ovine	1997	Sicile, Italy
3	6	3	14	1	1	3	3	7	43	8	6	6	4	7	3	2018Vergnaud#1400	B. melitensis bv3	49	87		BCCN#99-45	Ovine	1999	Sicile, Italy
3	6	3	14	1	1	3	3	7	43	8	6	6	9	7	3	SRR4436617	B. melitensis	49	87	87	BwIM_ITA_55	Human	2017	Italy
3	6	3	14	1	1	3	3	7	43	8	6	6	10	7	3	2018Vergnaud#1097	B. melitensis bv3	49	87		BCCN#99-50	Ovine	1999	Sicile, Italy
3	6	3	14	1	1	3	3	7	43	8	6	6	4	8	3	2018Vergnaud#1215	B. melitensis bv3	49	87		BCCN#99-46	Ovine	1999	Sicile, Italy
3	6	3	14	1	1	3	3	7	43	8	6	6	9	5	3	2013Garofolo_13.	B. melitensis bv3	49	87		13543	Ovine	2011	Mineo,Italy
3	6	3	14	1	1	3	3	7	43	8	6	6	12	2	3	2018Vergnaud#0850	B. melitensis bv3	49	87		BCCN#96-58	Ovine	1996	Sicile, Italy
3	6	3	14	1	1	3	3	7	43	8	6	6	6	6	3	2018Vergnaud#1244	B. melitensis bv3	49	87		BCCN#96-53	Ovine	1996	Sicile, Italy
3	6	3	14	1	1	3	3	7	43	8	5	6	13	7	5	2018Vergnaud#0436	B. melitensis bv3	49	87		BCCN#97-37	Ovine	1997	Sicile, Italy
3	5	3	14	1	1	3	3	7	43	8	7	4	9	8	3	2006LeFleche#093	B. melitensis bv3	50	86		BCCN#96-97	Cattle	1996	Sicilia, Italia
3	5	3	14	1	1	3	3	7	43	8	8	4	8	12	3	2018Vergnaud#0411	B. melitensis bv3	50	86		BCCN#93-38	Human	1993	Tunisia
3	7	3	14	1	1	3	3	7	43	8	5	4	16	7	3	2013Garofolo_11.	B. melitensis bv3	106	204		11791	Ovine	2011	Noto,Italy
3	6	3	14	1	1	3	3	8	43	8	6	6	7	6	3	2018Vergnaud#0981	B. melitensis bv3	49	162		BCCN#97-91	Ovine	1997	Sicile, Italy
3	6	3	14	1	1	3	3	8	43	8	6	4	7	12	3	2018Vergnaud#1287	B. melitensis bv3	49	162		BCCN#97-67	Ovine	1997	Sicile, Italy
3	6	3	14	1	1	3	3	8	43	8	6	9	12	9	3	2018Vergnaud#0659	B. melitensis bv3	49	162		BCCN#97-18	Caprine	1997	Sicile, Italy
3	6	3	14	1	1	3	3	8	43	8	4	10	8	6	3	2007Marianelli#002	B. melitensis bv3	49	162		DG1	Human	2005	Catania, Italy
3	6	3	14	1	1	3	3	8	43	8	6	8	8	6	3	2018Vergnaud#0647	B. melitensis bv3	49	162		BCCN#96-49	Cattle	1996	Sicile, Italy
3	6	3	14	1	1	3	3	8	43	8	4	4	5	8	3	2018Vergnaud#0673	B. melitensis bv3	49	162		BCCN#99-10	Ovine	1999	Sicile, Italy
3	6	3	14	1	1	3	3	8	43	8	7	7	10	7	3	2018Vergnaud#0650	B. melitensis bv3	49	162		BCCN#96-74	Ovine	1996	Sicile, Italy
3	6	3	14	1	1	3	3	8	43	8	7	7	10	7	3	2018Vergnaud#1017	B. melitensis bv3	49	162		BCCN#96-92	Ovine	1996	Sicile, Italy
3	6	3	14	1	1	3	3	8	43	8	7	7	10	7	3	2018Vergnaud#1211	B. melitensis bv3	49	162		BCCN#97-76	Ovine	1997	Sicile, Italy
3	6	3	14	1	1	3	3	8	43	8	7	7	7	5	3	2013Garofolo_13.	B. melitensis bv3	49	162		13553	Ovine	2011	Marsala,Italy
3	6	3	14	1	1	3	3	8	43	8	7	7	11	8	3	2018Vergnaud#0661	B. melitensis bv3	49	162		BCCN#97-33	Ovine	1997	Sicile, Italy

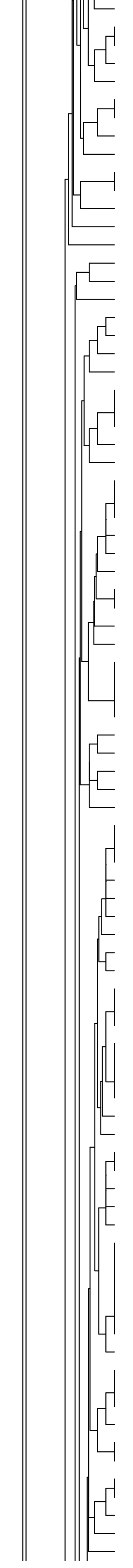

3	6	3	14	1	1	3	3	8	43	8	5	7	9	7	3	2018Vergnaud#0690	B. melitensis bv3	49	162		BCCN#99-68	Ovine	1999	Sicile, Italy
3	6	3	14	1	1	3	3	8	43	8	6	7	13	8	3	2018Vergnaud#1249	B. melitensis bv3	49	162		BCCN#97-82	Ovine	1997	Sicile, Italy
3	6	3	14	1	1	3	3	8	43	8	6	7	13	8	3	2018Vergnaud#1255	B. melitensis bv3	49	162		BCCN#97-81	Ovine	1997	Sicile, Italy
3	6	3	14	1	1	3	3	8	43	8	6	7	13	12	3	2018Vergnaud#1250	B. melitensis bv3	49	162		BCCN#97-83	Ovine	1997	Sicile, Italy
3	6	3	14	1	1	3	3	6	43	8	6	7	15	8	3	2013Garofolo_21.	B. melitensis bv3	49	203		21687	Ovine	2011	Petrizzi,Italy
3	6	3	14	1	1	3	3	8	43	8	9	6	11	7	3	2018Vergnaud#1213	B. melitensis bv3	49	162		BCCN#97-86	Ovine	1997	Sicile, Italy
3	6	3	14	1	1	3	3	8	43	8	9	6	11	7	3	2018Vergnaud#1256	B. melitensis bv3	49	162		BCCN#97-84	Ovine	1997	Sicile, Italy
3	6	3	14	1	1	3	3	8	43	8	9	5	11	5	3	2018Vergnaud#0656	B. melitensis bv3	49	162		BCCN#97-12	Cattle	1997	Sicile, Italy
3	6	3	14	1	1	3	3	8	43	8	8	6	14	7	6	2018Vergnaud#0370	B. melitensis bv3	49	162		BCCN#97-34	Caprine	1997	Sicile, Italy
3	6	3	14	1	1	3	3	8	45	8	5	4	8	7	3	2006LeFleche#089	B. melitensis bv3	49	88		BCCN#95-36	Caprine	1995	Sicilia, Italia
3	6	3	14	1	1	3	3	8	45	8	5	4	8	7	3	2018Vergnaud#0645	B. melitensis bv3	49	88		BCCN#96-44	Caprine	1996	Sicile, Italy
3	6	3	14	1	1	3	3	7	45	8	7	4	6	6	3	2006LeFleche#088	B. melitensis bv3	49	89		BCCN#95-30	Ovine	1995	Sicilia, Italia
3	6	3	14	1	1	3	3	7	43	8	4	3	12	11	6	2018Vergnaud#0378	B. melitensis bv3	49	87		BCCN#04-8	Cattle	2004	Sicile, Italy
4	6	3	14	2	1	3	3	7	43	8	6	4	4	4	3	2013Garofolo_744	B. melitensis bv3	107	206		744	Ovine	2011	Apricena,Italy
3	5	3	13	1	1	3	3	7	43	8	8	4	6	12	5	2018Vergnaud#0484	B. melitensis bv2	51	96		BCCN#76-360	Human	1976	Grenoble ,Haute-Savoie.
3	5	3	13	1	1	3	3	7	43	8	6	4	5	5	5	2018Vergnaud#0505	B. melitensis bv3	51	96		BCCN#78-83	Human	1978	Hérault, France
3	5	3	13	1	1	3	3	7	43	8	6	6	7	9	4	2018Vergnaud#1207	B. melitensis bv3	51	96		BCCN#93-52	Human	1993	Bouches-du-Rhone, Fr.
3	5	3	13	1	1	3	3	6	43	8	5	5	11	6	3	2018Vergnaud#0127	B. melitensis bv3	51	207		BCCN#80-240	Human	1980	Paris, France
3	5	3	13	1	1	3	3	6	43	8	5	5	12	6	3	2018Vergnaud#1378	B. melitensis bv3	51	207		BCCN#80-241	Human	1980	Vienne, France
3	5	3	13	1	1	3	3	9	43	8	5	5	9	6	3	2013Garofolo_1995	B. melitensis bv3	51	92		1995	Caprine	2011	Ravello,Italy
3	5	3	13	1	1	3	3	6	43	8	5	7	12	5	3	2018Vergnaud#0016	B. melitensis bv2	51	207		BCCN#74-247	Human	1974	Corse, France
3	5	3	13	1	1	3	3	6	43	8	8	5	7	8	3	2018Vergnaud#0017	B. melitensis bv2	51	207		BCCN#74-249	Human	1974	Paris, France
3	5	3	13	1	1	3	3	6	43	8	8	5	7	8	3	2018Vergnaud#0020	B. melitensis bv2	51	207		BCCN#74-251	Human	1974	Paris, France
3	5	3	13	1	1	3	3	6	43	8	8	5	7	8	3	2018Vergnaud#0130	B. melitensis bv3	51	207		BCCN#77-108	Human	1977	France
3	5	3	13	1	1	3	3	6	43	8	8	5	5	10	3	2018Vergnaud#0554	B. melitensis bv3	51	207		BCCN#83-32	Human	1983	Bastia ,Haute-Corse, F.
3	5	3	13	1	1	3	3	6	49	8	8	5	9	5	3	2018Vergnaud#0563	B. melitensis bv3	51	307		BCCN#81-122	Ovine	1981	Corse, France
3	5	3	13	1	1	3	3	6	43	8	6	5	8	6	3	2018Vergnaud#0844	B. melitensis bv3	51	207		BCCN#92-60	Cattle	1992	Haute-Savoie, France
3	5	3	13	1	1	3	3	6	43	8	6	5	8	6	3	2018Vergnaud#1178	B. melitensis bv3	51	207		BCCN#92-7	Cattle	1992	Savoie, France
3	5	3	13	1	1	3	3	6	43	8	6	5	8	6	3	2018Vergnaud#1179	B. melitensis bv3	51	207		BCCN#92-8	Cattle	1992	Savoie, France
3	5	3	13	1	1	3	3	6	43	8	6	5	8	14	3	2018Vergnaud#0830	B. melitensis bv3	51	207		BCCN#90-52	Caprine	1990	Savoie, France
3	5	3	13	1	1	3	3	6	43	8	6	5	8	12	3	2018Vergnaud#1130	B. melitensis bv3	51	207		BCCN#88-19	Ovine	1988	Savoie, France
3	5	3	13	1	1	3	3	6	43	8	6	5	9	21	3	2018Vergnaud#0584	B. melitensis bv3	51	207		BCCN#90-31	Ovine	1990	Vaucluse, France
3	5	3	13	1	1	3	3	6	43	8	6	6	8	13	3	2018Vergnaud#0511	B. melitensis bv3	51	207		BCCN#80-371	Cattle	1980	Savoie, France
3	5	3	13	1	1	3	3	6	43	8	6	6	8	13	3	2018Vergnaud#0512	B. melitensis bv3	51	207		BCCN#80-376	Cattle	1980	Savoie, France
3	5	3	13	1	1	3	3	5	43	8	6	5	8	9	3	2018Vergnaud#0487	B. melitensis bv2	51	93		BCCN#76-361	Human	1976	Nice ,Alpes-Maritimes, .
3	5	3	13	1	1	3	3	6	43	8	7	5	8	10	3	2018Vergnaud#0514	B. melitensis bv3	51	207		BCCN#80-40	Ovine	1980	Corse, France
2	5	3	13	1	1	3	3	7	43	8	6	5	8	6	3	2013Garofolo_20.	B. melitensis bv3	110	205		20020	Ovine	2011	San Marco Argentano,It.
2	5	3	13	1	1	3	3	7	43	8	6	5	8	6	3	2013Garofolo_6057	B. melitensis bv3	110	205		6057	Ovine	2011	San Lucido,Italy
2	5	3	13	1	1	3	3	7	43	8	6	5	8	6	3	2013Garofolo_6059	B. melitensis bv3	110	205		6059	Ovine	2011	Reggio Emilia,Italy
2	5	3	13	1	1	3	3	7	43	8	6	5	8	6	3	2013Garofolo_6076	B. melitensis bv3	110	205		6076	Cattle	2011	Mongrassano,Italy
3	5	3	13	1	1	3	3	6	43	8	9	4	8	7	3	2018Vergnaud#0482	B. melitensis bv2	51	207		BCCN#74-254	Human	1974	Corse, France
3	5	3	13	1	1	3	3	6	43	8	6	4	9	7	3	2018Vergnaud#0542	B. melitensis bv3	51	207		BCCN#83-22	Human	1983	France
3	5	3	13	1	1	3	3	6	43	8	5	4	7	11	3	2018Vergnaud#0133	B. melitensis bv3	51	207		BCCN#78-154	Human	1978	Nord, France
3	5	3	13	1	1	3	3	6	43	8	10	4	7	5	3	2018Vergnaud#0938	B. melitensis bv2	51	207		BCCN#76-359	Human	1976	Grenoble ,Haute-Savoie.
3	5	3	13	1	1	3	3	6	43	8	7	4	12	12	3	2018Vergnaud#0224	B. melitensis bv3	51	207		BCCN#01-18	Chamois	2001	Savoie, France
3	5	3	13	1	1	3	3	7	43	8	7	5	7	5	3	2018Vergnaud#0103	B. melitensis bv3	51	96		BCCN#83-2	Cattle	1983	Thones ,Haute-Savoie, .
3	5	3	13	1	1	3	3	7	43	8	7	5	7	5	3	2018Vergnaud#0531	B. melitensis bv3	51	96		BCCN#83-1	Cattle	1983	Thones ,Haute-Savoie, .
3	5	3	13	1	1	3	3	7	43	8	7	5	7	5	3	2018Vergnaud#0555	B. melitensis bv3	51	96		BCCN#83-4b	Cattle	1983	Vimines ,Savoie, France
3	5	3	13	1	1	3	3	7	43	8	7	5	7	10	3	2018Vergnaud#1203	B. melitensis bv3	51	96		BCCN#91-171	Ovine	1991	Var, France
3	5	3	13	1	1	3	3	7	43	8	7	5	7	11	3	2018Vergnaud#0529	B. melitensis bv3	51	96		BCCN#82-97	Cattle	1982	Tessens ,Savoie, France
3	5	3	13	1	1	3	3	7	43	8	7	5	7	6	3	2018Vergnaud#0821	B. melitensis bv3	51	96		BCCN#81-135	Human	1981	Bouches-du-Rhone, Fr.
3	5	3	13	1	1	3	3	7	43	8	7	5	8	5	3	2018Vergnaud#0622	B. melitensis bv3	51	96		BCCN#93-55	Human	1993	Hautes-Alpes, France
3	5	3	13	1	1	3	3	7	43	8	7	5	13	10	3	2018Vergnaud#0007	B. melitensis bv3	51	96		BCCN#R3	Caprine		Italy
3	5	3	13	1	1	3	3	7	43	8	7	5	14	10	3	GCA_000740355	B. melitensis bv3	51	96	9	REF Ether	unknown		
3	5	3	13	1	1	3	3	7	43	8	7	5	10	7	3	2018Vergnaud#0513	B. melitensis bv3	51	96		BCCN#80-377	Ovine	1980	Savoie, France
3	5	3	13	1	1	3	3	7	43	8	7	5	10	7	3	2018Vergnaud#1202	B. melitensis bv3	51	96		BCCN#91-169	Ovine	1991	Vaucluse, France
3	5	3	13	1	1	3	3	7	43	8	7	5	10	7	3	2018Vergnaud#1391	B. melitensis bv3	51	96		BCCN#91-170	Ovine	1991	Var, France
3	5	3	13	1	1	3	3	7	43	8	7	5	10	9	3	2018Vergnaud#0013	B. melitensis bv2	51	96		BCCN#74-233	Human	1974	Rhone, France
3	5	3	13	1	1	3	3	7	43	8	7	5	10	9	3	2018Vergnaud#0480	B. melitensis bv2	51	96		BCCN#74-231	Human	1974	Rhone, France
3	5	3	13	1	1	3	3	7	43	8	7	5	10	9	3	2018Vergnaud#0809	B. melitensis bv2	51	96		BCCN#74-234	Human	1974	Rhone, France
3	5	3	13	1	1	3	3	7	43	8	7	5	10	9	3	2018Vergnaud#0932	B. melitensis bv3	51	96		BCCN#86-19	Cattle	1986	Hautes-Alpes, France
3	5	3	13	1	1	3	3	7	43	8	7	5	12	9	3	2012Ferreira#003	B. melitensis bv3	51	96		REF Ether	unknown		
3	5	3	13	1	1	3	3	7	43	8	7	5	11	7	3	2018Vergnaud#0548	B. melitensis bv3	51	96		BCCN#78-149	Human	1978	Ansouis ,Vaucluse, Fra.
3	5	3	13	1	1	3	3	7	43	8	7	5	9	12	3	2018Vergnaud#0090	B. melitensis bv2	51	96		BCCN#74-252	Ovine	1974	Nice ,Alpes-Maritimes, .
3	5	3	13	1	1	3	3	7	43	8	7	5	9	12	3	2018Vergnaud#0124	B. melitensis bv3	51	96		BCCN#79-50	Caprine	1979	Alpes-Maritimes, France
3	5	3	13	1	1	3	3	7	43	8	7	5	9	6	3	2018Vergnaud#0898	B. melitensis bv3	51	96		BCCN#81-138	Human	1981	Pertuis ,Vaucluse, France
3	5	3	13	1	1	3	3	7	43	8	7	5	9	8	3	2018Vergnaud#0358	B. melitensis R.	51	96		BCCN#92-2	Human	1992	Hautes-Alpes, France
3	5	3	13	1	1	3	3	7	43	8	7	5	9	4	3	2018Vergnaud#0883	B. melitensis bv2	51	96		BCCN#74-257	Human	1974	France
3	5	3	13	1	1	3	3	7	43	8	7	4	9	9	3	2018Vergnaud#0361	B. melitensis R.	51	96		BCCN#93-33	Caprine	1993	Alpes-de-Haute-Proven.
3	5	3	13	1	1	3	3	7	43	8	7	4	9	9	3	2018Vergnaud#0624	B. melitensis bv3	51	96		BCCN#93-58	Cattle	1993	Vaucluse, France
3	5	3	13	1	1	3	3	7	43	8	7	4	9	9	3	2018Vergnaud#0625	B. melitensis bv3	51	96		BCCN#93-59	Cattle	1993	Vaucluse, France
3	5	3	13	1	1	3	3	7	43	8	7	4	9	9	3	2018Vergnaud#0843	B. melitensis bv3	51	96		BCCN#92-54	Caprine	1992	Alpes-de-Haute-Proven.
3	5	3	13	1	1	3	3	7	43	8	7	4	9	9	3	2018Vergnaud#0891	B. melitensis bv3	51	96		BCCN#92-61	Caprine	1992	Alpes-de-Haute-Proven.
3	5	3	13	1	1	3	3	7	43	8	7	4	9	9	3	2018Vergnaud#1180	B. melitensis bv3	51	96		BCCN#93-46	Human	1993	Bouches-du-Rhone, Fr.
3	5	3	13	1	1	3	3	7	43	8	7	5	9	9	3	2018Vergnaud#0018	B. melitensis bv2	51	96		BCCN#74-230	Human	1974	Rhone, France
3	5	3	13	1	1	3	3	7	43	8	7	9	6	11	3	2013Garofolo_15.	B. melitensis bv3	51	96		15078	Ovine	2011	Rignano Garganico,Italy
3	5	3	13	1	1	3	3	7	43	8	7	9	6	11	3	2013Garofolo_6837	B. melitensis bv3	51	96		6837	Ovine	2011	Vieste,Italy
3	5	3	13	1	1	3	3	7	43	8	7	9	6	11	3	2013Garofolo_6838	B. melitensis bv3	51	96		6838	Ovine	2011	Rignano Graganico,Italy
3	5	3	13	1	1	3	3	7	43	8	7	9	6	9	3	2013Garofolo_6839	B. melitensis bv3	51	96		6839	Ovine	2011	San Severo,Italy
3	5	3	13	1	1	3	3	7	43	8	7	7	6	8	3	2013Garofolo_11.	B. melitensis bv3	51	96		11825	Ovine	2011	Aci Catena,Italy
3	5	3	13	1	1	3	3	7	43	8	7	7	6	8	3	2013Garofolo_11.	B. melitensis bv3	51	96		11826	Ovine	2011	Aci Catena,Italy
3	5	3	13	1	1	3	3	7	43	8	7	6	10	10	3	2018Vergnaud#0381	B. melitensis bv3	51	96		BCCN#82-82	Human	1982	Belgium
3	5	3	13	1	1	3	3	7	43	8	7	6	10	10	3	2018Vergnaud#0408	B. melitensis bv3	51	96		BCCN#82-38	Human	1982	Belgium
3	5	3	13	1	1	3	3	7	43	8	8	6	10	10	3	2018Vergnaud#0383	B. melitensis bv3	51	96		BCCN#82-96	Human	1982	Belgium
2	5	3	13	1	1	3	3	7	43	8	7	5	10	10	3	SRR138272	B. melitensis bv3	110	205	9	REF Ether	unknown		
3	5	3	13	1	1	3	3	7	43	8	7	5	10	8	5	2018Vergnaud#0422	B. melitensis bv3	51	96		BCCN#97-6	Human	1997	Bouches-du-Rhone, Fr.

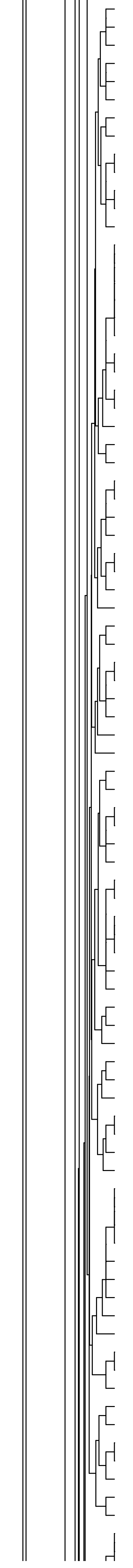

3	5	3	13	1	1	3	3	7	43	8	7	5	8	11	3	2018Vergnaud#0520	B. melitensis bv3	51	96		BCCN#82-103	Cattle	1982	Les Chapelles ,Savoie, .
3	5	3	13	1	1	3	3	7	43	8	10	5	8	11	3	2018Vergnaud#1281	B. melitensis bv3	51	96		BCCN#94-32	Human	1994	Bouches-du-Rhone, Fr.
3	5	3	13	1	1	3	3	7	43	8	6	5	8	11	3	2018Vergnaud#0910	B. melitensis bv3	51	96		BCCN#87-98	Cattle	1987	Hautes-Alpes, France
3	5	3	13	1	1	3	3	7	43	8	8	5	8	4	3	2007AlDahouk#065	B. melitensis bv2	51	96		BfR 31	Human	2001	Munchen, Germany
3	5	3	13	1	1	3	3	7	43	8	8	5	8	8	3	2018Vergnaud#0420	B. melitensis bv3	51	96		BCCN#97-50	Human	1997	France
3	5	3	13	1	1	3	3	7	43	8	8	5	8	11	3	2018Vergnaud#0571	B. melitensis bv3	51	96		BCCN#87-49	Ovine	1987	Hérault, France
3	5	3	13	1	1	3	3	7	43	8	8	5	13	7	3	2018Vergnaud#0015	B. melitensis bv2	51	96		BCCN#74-245	Ovine	1974	Nice ,Alpes-Maritimes, .
3	5	3	13	1	1	3	3	7	43	8	8	5	4	7	3	2018Vergnaud#0948	B. melitensis bv3	51	96		BCCN#89-76	Human	1989	Moselle, France
3	5	3	13	1	1	3	3	7	43	8	8	5	8	7	3	2018Vergnaud#0544	B. melitensis bv2	51	96		BCCN#74-246	Human	1974	Maine-et-Loire, France
3	5	3	13	1	1	3	3	7	43	8	8	5	8	7	3	2018Vergnaud#0662	B. melitensis bv3	51	96		BCCN#97-52	Human	1997	Pyrénées-Orientales, F.
3	5	3	13	1	1	3	3	7	43	8	6	5	8	7	3	2018Vergnaud#0541	B. melitensis bv3	51	96		BCCN#83-217	Cattle	1983	Thones ,Haute-Savoie, .
3	5	3	13	1	1	3	3	7	43	8	6	5	8	7	3	2018Vergnaud#0944	B. melitensis bv3	51	96		BCCN#78-91	Human	1978	Bouches-du-Rhone, Fr.
3	5	3	13	1	1	3	3	7	43	8	7	5	8	7	3	2018Vergnaud#0096	B. melitensis bv3	51	96		BCCN#79-136	Cattle	1979	Haute-Savoie, France
3	5	3	13	1	1	3	3	7	43	8	5	5	8	6	3	2018Vergnaud#0551	B. melitensis bv3	51	96		BCCN#83-26	Ovine	1983	Rambaud ,Hautes-Alpes.
3	5	3	13	1	1	3	3	7	43	8	5	5	8	6	3	2018Vergnaud#0846	B. melitensis bv3	51	96		BCCN#92-96	Cattle	1992	Savoie, France
3	5	3	13	1	1	3	3	7	43	8	5	5	8	6	3	2018Vergnaud#1004	B. melitensis bv3	51	96		BCCN#91-302	Cattle	1991	Savoie, France
3	5	3	13	1	1	3	3	7	43	8	5	5	8	6	3	2018Vergnaud#1010	B. melitensis bv3	51	96		BCCN#92-97	Cattle	1992	Savoie, France
3	5	3	13	1	1	3	3	7	43	8	5	5	8	6	3	2018Vergnaud#1011	B. melitensis bv3	51	96		BCCN#92-98	Cattle	1992	Savoie, France
3	5	3	13	1	1	3	3	7	43	8	5	5	8	6	3	2018Vergnaud#1012	B. melitensis bv3	51	96		BCCN#92-99	Cattle	1992	Savoie, France
3	5	3	13	1	1	3	3	7	43	8	5	5	8	7	3	2018Vergnaud#0354	B. melitensis R.	51	96		BCCN#83-208	Human	1983	France
3	5	3	13	1	1	3	3	7	43	8	5	5	8	7	3	2018Vergnaud#0591	B. melitensis bv3	51	96		BCCN#90-95	Caprine	1990	Hautes-Alpes, France
3	5	3	13	1	1	3	3	7	43	8	5	5	8	8	3	2018Vergnaud#0105	B. melitensis bv3	51	96		BCCN#85-3	Cattle	1985	Loiret, France
3	5	3	13	1	1	3	3	7	43	8	5	5	8	8	3	2018Vergnaud#0567	B. melitensis bv3	51	96		BCCN#85-4	Cattle	1985	Loiret, France
3	5	3	13	1	1	3	3	7	43	8	5	5	10	6	3	2018Vergnaud#1390	B. melitensis bv3	51	96		BCCN#90-18	Human	1990	Val-de-Marne, France
3	5	3	13	1	1	3	3	7	43	8	5	5	6	8	3	2007Marianelli#005	B. melitensis bv3	51	96		CS13	Human	2005	Catania, Italy
3	5	3	13	1	1	3	3	7	43	8	5	5	6	10	3	2018Vergnaud#0945	B. melitensis bv3	51	96		BCCN#78-93	Human	1978	Lyon ,Rhone, France
3	5	3	13	1	1	3	3	7	43	8	6	5	8	8	3	2018Vergnaud#0011	B. melitensis bv2	51	96		BCCN#76-362	Human	1976	Montpellier ,Hérault, Fr.
3	5	3	13	1	1	3	3	7	43	8	6	5	8	8	3	2018Vergnaud#0104	B. melitensis bv3	51	96		BCCN#84-2	Cattle	1984	Thones ,Haute-Savoie, .
3	5	3	13	1	1	3	3	7	43	8	6	5	4	8	3	2018Vergnaud#1069	B. melitensis bv3	51	96		BCCN#88-66	Cattle	1988	Hautes-Alpes, France
3	5	3	13	1	1	3	3	7	43	8	6	5	9	8	3	2018Vergnaud#0931	B. melitensis bv3	51	96		BCCN#86-18	Cattle	1986	Hautes-Alpes, France
3	5	3	13	1	1	3	3	7	43	8	9	5	8	8	3	2018Vergnaud#0125	B. melitensis bv3	51	96		BCCN#80-36	Ovine	1980	Alpes-Maritimes, France
3	5	3	13	1	1	3	3	7	43	8	9	5	8	8	3	2018Vergnaud#1040	B. melitensis bv3	51	96		BCCN#80-51	Ovine	1980	Cher, France
3	5	3	13	1	1	3	3	7	43	8	9	5	9	8	3	2018Vergnaud#0939	B. melitensis bv2	51	96		BCCN#76-363	Human	1976	Carcassonne ,Aude, Fr.
3	5	3	13	1	1	3	3	7	43	8	8	5	11	8	3	2018Vergnaud#0940	B. melitensis bv3	51	96		BCCN#00-22	Human	2000	France
3	5	3	13	1	1	3	3	7	43	8	8	5	7	10	3	2018Vergnaud#0019	B. melitensis bv2	51	96		BCCN#74-244	Ovine	1974	Nice ,Alpes-Maritimes, .
3	5	3	13	1	1	3	3	7	43	8	8	5	7	9	3	2018Vergnaud#1307	B. melitensis bv3	51	96		BCCN#91-229	Ovine	1991	Saragosse ,Spain
3	5	3	13	1	1	3	3	7	43	8	9	5	7	7	3	2018Vergnaud#0975	B. melitensis bv3	51	96		BCCN#91-120	Cattle	1991	Pyrénées-Orientales, F.
3	5	3	13	1	1	3	3	7	43	8	9	5	7	7	3	2018Vergnaud#0989	B. melitensis bv3	51	96		BCCN#91-121	Cattle	1991	Pyrénées-Orientales, F.
3	5	3	13	1	1	3	3	7	43	8	5	5	7	7	3	2006LeFleche#086	B. melitensis bv2	51	96		BCCN#95-33	Ovine	1995	Sicilia, Italia
3	5	3	13	1	1	3	3	7	43	8	8	5	7	7	3	2018Vergnaud#1021	B. melitensis bv3	51	96		BCCN#99-94	Human	1999	Seine-et-Marne, France
3	5	3	13	1	1	3	3	7	43	8	4	5	7	6	3	2007AlDahouk#050	B. melitensis bv2	51	96		BfR 2	Human	2000	Bern, Switzerland
3	5	3	13	1	1	3	3	7	45	8	5	5	7	7	3	2007AlDahouk#078	B. melitensis bv2	51	94		BfR 53	Human	2001	Bern, Switzerland
3	5	3	13	1	1	3	3	7	43	8	6	5	4	5	3	2018Vergnaud#0674	B. melitensis bv3	51	96		BCCN#99-20	Ovine	1999	Sicile, Italy
3	5	3	13	1	1	3	3	7	43	8	6	5	5	5	3	2018Vergnaud#0688	B. melitensis bv3	51	96		BCCN#99-62	Ovine	1999	Sicile, Italy
3	5	3	13	1	1	3	3	7	43	8	6	5	7	11	3	2018Vergnaud#0519	B. melitensis bv3	51	96		BCCN#82-102	Cattle	1982	Les Chapelles ,Savoie, .
3	5	3	13	1	1	3	3	7	43	8	6	5	7	11	3	2018Vergnaud#0588	B. melitensis bv3	51	96		BCCN#90-44	Cattle	1990	Hautes-Alpes, France
3	5	3	13	1	1	3	3	7	43	8	6	5	7	5	3	2018Vergnaud#0114	B. melitensis bv3	51	96		BCCN#83-5	Cattle	1983	Marthod ,Savoie, France
3	5	3	13	1	1	3	3	7	43	8	6	5	7	12	3	2018Vergnaud#1387	B. melitensis bv3	51	96		BCCN#89-48	Cattle	1989	Hautes-Alpes, France
3	5	3	13	1	1	3	3	7	43	8	6	5	6	9	3	2018Vergnaud#0633	B. melitensis bv3	51	96		BCCN#95-21	Cattle	1995	La Tourrette ,Loire, Fra.
3	5	3	13	1	1	3	3	7	43	8	6	5	6	9	3	2018Vergnaud#0847	B. melitensis bv3	51	96		BCCN#94-5	Human	1994	Drome, France
3	5	3	13	1	1	3	3	7	43	8	6	5	4	9	3	2018Vergnaud#0102	B. melitensis bv3	51	96		BCCN#82-100	Ovine	1982	Freissinieres ,Hautes-A.
3	5	3	13	1	1	3	3	7	43	8	6	5	4	9	3	2018Vergnaud#0549	B. melitensis bv3	51	96		BCCN#83-24	Ovine	1983	Chateauroux ,Hautes-A.
3	5	3	13	1	1	3	3	7	43	8	6	5	4	9	3	2018Vergnaud#0550	B. melitensis bv3	51	96		BCCN#83-25	Ovine	1983	Freissinieres ,Hautes-A.
3	5	3	13	1	1	3	3	7	43	8	6	5	16	9	3	2018Vergnaud#0225	B. melitensis bv3	51	96		BCCN#94-59	Deer/El.	1994	Allier, France
3	5	3	13	1	1	3	3	7	43	8	6	5	12	9	3	2018Vergnaud#0779	B. melitensis bv3	51	96		BCCN#90-50	Ovine	1990	Hautes-Alpes, France
3	5	3	13	1	1	3	3	7	43	8	6	5	9	10	3	2018Vergnaud#0517	B. melitensis bv3	51	96		BCCN#81-130	Human	1981	Dijon,Cote d'Or, France
3	5	3	13	1	1	3	3	7	43	8	6	5	8	10	3	2018Vergnaud#0988	B. melitensis bv3	51	96		BCCN#86-1	Ovine	1986	Saint-Sebastien ,Isère, .
3	5	3	13	1	1	3	3	8	43	8	6	5	9	10	3	2018Vergnaud#0598	B. melitensis bv3	51	91		BCCN#91-186	Caprine	1991	Alpes-Maritimes, France
3	5	3	13	1	1	3	3	7	43	8	6	5	9	13	3	2018Vergnaud#0810	B. melitensis bv2	51	96		BCCN#74-236	Human	1974	Paris, France
3	5	3	13	1	1	3	3	7	43	8	9	5	9	13	3	2018Vergnaud#1135	B. melitensis bv3	51	96		BCCN#90-20	Ovine	1990	Var, France
3	5	3	13	1	1	3	3	7	43	8	9	5	9	11	3	2018Vergnaud#0901	B. melitensis bv3	51	96		BCCN#86-12	Ovine	1986	Alpes-de-Haute-Proven.
3	5	3	13	1	1	3	3	7	43	8	8	5	9	5	3	2018Vergnaud#0355	B. melitensis R.	51	96		BCCN#88-8	Human	1988	Ille-et-Vilaine, France
3	5	3	13	1	1	3	3	7	43	8	8	5	9	5	3	2018Vergnaud#0527	B. melitensis bv3	51	96		BCCN#78-59	Caprine	1978	Isère, France
3	5	3	13	1	1	3	3	7	43	8	5	5	9	5	3	2018Vergnaud#0120	B. melitensis bv3	51	96		BCCN#75-199	Human	1975	Rhone, France
3	5	3	13	1	1	3	3	7	43	8	8	5	9	4	3	2018Vergnaud#0515	B. melitensis bv3	51	96		BCCN#81-100	Ovine	1981	Aime ,Savoie, France
3	5	3	13	1	1	3	3	7	43	8	9	5	6	6	3	2007AlDahouk#008	B. melitensis bv2	51	96		BCCN#74-235	Human	1974	Lyon, France
3	5	3	13	1	1	3	3	7	43	8	9	5	6	6	3	2018Vergnaud#0044	B. melitensis bv3	51	96		BCCN#82-11	Ovine	1982	Saint Julien en Bochene.
3	5	3	13	1	1	3	3	7	43	8	9	5	6	6	3	2018Vergnaud#0616	B. melitensis bv3	51	96		BCCN#93-42	Human	1993	France
3	5	3	13	1	1	3	3	7	43	8	9	5	6	6	3	2018Vergnaud#0632	B. melitensis bv3	51	96		BCCN#95-20	Human	1995	Haute-Garonne, France
3	5	3	13	1	1	3	3	7	43	8	3	5	6	6	3	2007Marianelli#014	B. melitensis bv3	51	96		DZ7	Human	2005	Catania, Italy
3	5	3	13	1	1	3	3	7	43	8	5	5	6	6	3	2018Vergnaud#0570	B. melitensis bv3	51	96		BCCN#87-44	Ovine	1987	Hautes-Alpes, France
3	5	3	13	1	1	3	3	7	43	8	6	5	6	6	3	SRR1925727	B. melitensis	51	96	11	Human/CT/US.	Human		USA: Connecticut
3	5	3	13	1	1	3	3	7	49	8	9	5	6	6	3	2018Vergnaud#0902	B. melitensis bv3	51	95		BCCN#86-15	Cattle	1986	Pouyastruc ,Hautes-Py.
3	5	3	13	1	1	3	3	7	43	8	8	5	5	6	3	2018Vergnaud#0412	B. melitensis bv3	51	96		BCCN#93-39	Human	1993	Tunisia
3	5	3	14	1	1	3	3	7	43	8	6	5	5	6	3	2018Vergnaud#1046	B. melitensis bv3	50	86		BCCN#97-92	Ovine	1997	Sicile, Italy
3	5	3	14	1	1	3	3	7	43	8	6	5	5	6	3	2018Vergnaud#1212	B. melitensis bv3	50	86		BCCN#97-77	Ovine	1997	Sicile, Italy
3	5	3	14	1	1	3	3	7	43	8	6	5	6	6	3	2018Vergnaud#1088	B. melitensis bv3	50	86		BCCN#96-50	Ovine	1996	Sicile, Italy
3	5	3	13	1	1	3	3	7	43	8	9	5	14	4	3	2018Vergnaud#0543	B. melitensis bv3	51	96		BCCN#83-23	Ovine	1983	Upaix ,Hautes-Alpes, F.
3	5	3	13	1	1	3	3	7	43	8	9	5	10	4	3	2018Vergnaud#0968	B. melitensis bv3	51	96		BCCN#85-5	Human	1985	France
3	5	3	13	1	1	3	3	7	43	8	9	5	10	12	3	2018Vergnaud#0233	B. melitensis bv3	51	96		BCCN#91-203	Caprine	1991	France
3	5	3	13	1	1	3	3	7	43	8	9	5	10	12	3	2018Vergnaud#0234	B. melitensis bv3	51	96		BCCN#91-204	Caprine	1991	France
3	5	3	13	1	1	3	3	7	49	8	9	5	10	12	3	2018Vergnaud#0207	B. melitensis R.	51	95		BCCN#76-347	Human	1976	Toulouse ,Haute-Garo.
3	5	3	13	1	1	3	3	7	43	8	9	5	11	13	3	2018Vergnaud#0357	B. melitensis R.	51	96		BCCN#91-287	Caprine	1991	France
3	5	3	13	1	1	3	3	7	43	8	9	5	11	5	3	2018Vergnaud#0564	B. melitensis bv3	51	96		BCCN#81-123	Ovine	1981	Vaucluse, France
3	5	3	13	1	1	3	3	7	43	8	5	5	12	4	3	2006LeFleche#082	B. melitensis bv2	51	96		BCCN#77-6	Caprine	1977	Nice, France
3	5	3	13	1	1	3	3	7	43	8	5	5	12	4	3	2018Vergnaud#0908	B. melitensis bv3	51	96		BCCN#87-96	Ovine	1987	Hautes-Alpes, France

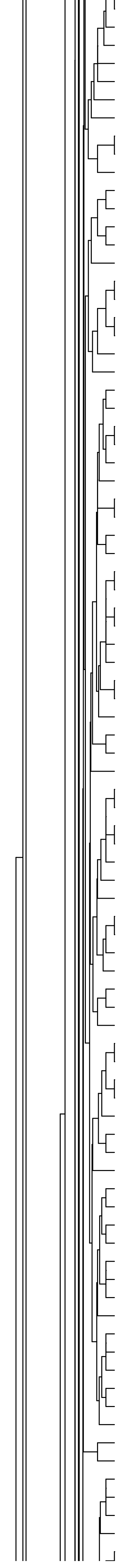

3	5	3	13	1	1	3	3	7	43	8	5	5	12	4	3	2018Vergnaud#0909	B. melitensis bv3	51	96		BCCN#87-97	Ovine	1987	Hautes-Alpes, France
3	5	3	13	1	1	3	3	7	43	8	5	5	12	9	3	2007Marianelli#012	B. melitensis bv3	51	96		MA5	Human	2005	Catania, Italy
3	5	3	13	1	1	3	3	7	43	8	5	5	12	4	5	2018Vergnaud#0503	B. melitensis bv3	51	96		BCCN#78-65	Ovine	1978	Alpes-Maritimes, France
3	5	3	13	1	1	3	3	7	43	8	5	5	13	9	3	2007Marianelli#018	B. melitensis bv3	51	96		ST4	Human	2005	Catania, Italy
3	5	3	13	1	1	3	3	8	43	8	5	5	12	9	3	2018Vergnaud#0741	B. melitensis bv3	51	91		BCCN#88-48	Ovine	1988	Hautes-Alpes, France
3	5	3	13	1	1	3	3	7	43	8	5	5	13	4	6	2018Vergnaud#0504	B. melitensis bv3	51	96		BCCN#78-66	Ovine	1978	Alpes-Maritimes, France
3	5	3	13	1	1	3	3	7	43	8	10	5	4	4	3	2018Vergnaud#0516	B. melitensis bv3	51	96		BCCN#81-114	Ovine	1981	Var, France
3	5	3	13	1	1	3	3	7	43	8	5	3	7	4	3	2006LeFleche#097	B. melitensis bv3	51	96		BCCN#94-16	Cattle	1994	Rodez, France
3	5	3	13	1	1	3	3	7	43	8	5	3	7	4	3	2018Vergnaud#0628	B. melitensis bv3	51	96		BCCN#94-17	Cattle	1994	Aveyron, France
3	5	3	13	1	1	3	3	7	43	7	5	3	5	4	3	2010Valdezate#084	B. melitensis	51	159		#20070849	Human	2007	Las Palmas, Spain
3	5	3	13	1	1	3	3	7	43	8	6	8	10	7	3	2018Vergnaud#0928	B. melitensis bv3	51	96		BCCN#80-41	Ovine	1980	Alpes-de-Haute-Proven.
3	5	3	13	1	1	3	3	7	43	8	6	8	8	7	3	SRR3106339	B. melitensis	51	96	89	BRUC048	Human		Egypt
3	5	3	13	1	1	3	3	7	43	8	6	5	9	7	3	2008Marianelli#1	B. melitensis bv3	51	96		PZ2	Human	2006	Roma, Italy
3	5	3	13	1	1	3	3	7	43	8	6	6	9	7	3	2018Vergnaud#1388	B. melitensis bv3	51	96		BCCN#90-103	Ovine	1990	Hautes-Alpes, France
3	5	3	13	1	1	3	3	7	43	8	6	4	8	7	6	2018Vergnaud#0489	B. melitensis bv2	51	96		BCCN#76-368	Human	1976	Montpellier ,Hérault, Fr.
3	5	3	13	1	1	3	3	7	43	8	6	7	8	10	3	2018Vergnaud#0221	B. melitensis bv3	51	96		BCCN#91-11	Chamois	1991	Hautes-Alpes, France
3	5	3	13	1	1	3	3	7	43	8	6	7	8	10	3	2018Vergnaud#0222	B. melitensis bv3	51	96		BCCN#91-118	Chamois	1991	Hautes-Alpes, France
3	5	3	13	1	1	3	3	7	43	8	6	7	8	9	3	2018Vergnaud#0219	B. melitensis bv3	51	96		BCCN#89-8	Chamois	1989	Rhone, France
3	5	3	13	1	1	3	3	7	43	8	6	7	8	9	3	2018Vergnaud#0220	B. melitensis bv3	51	96		BCCN#89-32	Chamois	1989	Rhone, France
3	5	3	13	1	1	3	3	7	43	8	6	7	4	6	3	2007Marianelli#007	B. melitensis bv3	51	96		MM14	Human	2005	Catania, Italy
3	5	3	13	1	1	3	3	7	43	8	5	7	5	9	3	2018Vergnaud#0561	B. melitensis bv3	51	96		BCCN#85-19	Ovine	1985	Chalet,Massiac ,Cantal,.
3	5	3	13	1	1	3	3	7	43	8	6	4	10	6	3	2018Vergnaud#0606	B. melitensis bv3	51	96		BCCN#92-103	Ovine	1992	Aveyron, France
3	5	3	13	1	1	3	3	7	43	8	6	4	6	6	3	2018Vergnaud#0997	B. melitensis bv3	51	96		BCCN#88-51	Ovine	1988	Hérault, France
3	5	3	13	1	1	3	3	7	43	8	6	4	8	6	3	2018Vergnaud#0014	B. melitensis bv2	51	96		BCCN#74-241	Human	1974	Paris, France
3	5	3	13	1	1	3	3	7	43	8	6	4	8	6	3	2018Vergnaud#0648	B. melitensis bv3	51	96		BCCN#96-64	Ovine	1996	Sicile, Italy
3	5	3	13	1	1	3	3	7	43	8	6	4	8	7	3	2018Vergnaud#0118	B. melitensis bv3	51	96		BCCN#75-67	Human	1975	France
3	5	3	13	1	1	3	3	7	43	8	6	4	6	12	3	2018Vergnaud#1168	B. melitensis bv3	51	96		BCCN#91-184	Ovine	1991	Gard, France
3	5	3	13	1	1	3	3	7	43	8	6	4	9	5	3	2006LeFleche#099	B. melitensis	51	96		BCCN#92-117	Ovine	1992	Rodez, France
3	5	3	13	1	1	3	3	7	43	8	6	4	9	5	3	2018Vergnaud#0213	B. melitensis R.	51	96		BCCN#92-116	Ovine	1992	Aveyron, France
3	5	3	13	1	1	3	3	7	43	8	6	4	3	11	3	2018Vergnaud#0842	B. melitensis bv3	51	96		BCCN#92-52	Ovine	1992	Vaucluse, France
3	5	3	13	1	1	3	3	7	43	8	6	4	14	11	3	2018Vergnaud#1080	B. melitensis bv3	51	96		BCCN#93-3	Human	1993	Hautes-Alpes, France
3	5	3	13	1	1	3	3	7	43	8	6	4	7	9	3	2018Vergnaud#1071	B. melitensis bv3	51	96		BCCN#89-51	Cattle	1989	Hautes-Alpes, France
3	5	3	13	1	1	3	3	7	43	8	6	4	7	9	3	2018Vergnaud#1072	B. melitensis bv3	51	96		BCCN#89-52	Cattle	1989	Hautes-Alpes, France
3	5	3	13	1	1	3	3	7	43	8	6	4	7	6	3	2018Vergnaud#0435	B. melitensis bv3	51	96		BCCN#97-36	Ovine	1997	Sicile, Italy
3	5	3	13	1	1	3	3	7	43	8	6	4	7	6	3	2018Vergnaud#0488	B. melitensis bv2	51	96		BCCN#76-366	Human	1976	Ales ,Gard, France
3	5	3	13	1	1	3	3	7	43	8	6	4	7	10	3	2018Vergnaud#0618	B. melitensis bv3	51	96		BCCN#92-43	Human	1992	Sfax ,Tunisia
3	5	3	13	1	1	3	3	7	43	8	6	4	7	5	3	2018Vergnaud#1068	B. melitensis bv3	51	96		BCCN#88-65	Ovine	1988	Hérault, France
3	5	3	13	1	1	3	3	7	43	8	8	4	7	6	3	2018Vergnaud#0562	B. melitensis bv3	51	96		BCCN#85-30	Cattle	1985	Valbonnais ,Isère, France
3	5	3	13	1	1	3	3	7	43	8	8	4	7	6	3	2018Vergnaud#0566	B. melitensis bv3	51	96		BCCN#85-35	Cattle	1985	St Georges d'Esperanc.
3	5	3	13	1	1	3	3	7	43	8	7	4	7	10	3	2018Vergnaud#0613	B. melitensis bv3	51	96		BCCN#92-45	Human	1992	Sfax ,Tunisia
3	5	3	13	1	1	3	3	7	43	8	4	4	6	5	3	2018Vergnaud#0380	B. melitensis bv3	51	96		BCCN#81-141	Hare	1981	Yvelines, France
3	5	3	13	1	1	3	3	7	43	8	4	4	6	6	3	2018Vergnaud#0612	B. melitensis bv3	51	96		BCCN#92-44	Human	1992	Sfax ,Tunisia
3	5	3	13	1	1	3	3	5	43	8	6	4	7	3	3	2007AlDahouk#013	B. melitensis bv3	51	93		BCCN#92-42	Human	1992	Tunisia
3	5	3	13	1	1	3	3	7	43	8	8	4	10	12	3	2018Vergnaud#1170	B. melitensis bv3	51	96		BCCN#91-250	Cattle	1991	France
3	5	3	13	1	1	3	3	7	43	8	8	4	10	12	3	2018Vergnaud#1171	B. melitensis bv3	51	96		BCCN#91-251	Cattle	1991	Isère, France
3	5	3	13	1	1	3	3	7	43	8	8	4	10	10	3	2018Vergnaud#0559	B. melitensis bv3	51	96		BCCN#85-15	Cattle	1985	Hautes-Alpes, France
3	5	3	13	1	1	3	3	7	43	8	8	4	10	10	3	2018Vergnaud#0560	B. melitensis bv3	51	96		BCCN#85-16	Cattle	1985	Hautes-Alpes, France
3	5	3	13	1	1	3	3	7	43	8	8	4	10	11	3	2018Vergnaud#0507	B. melitensis bv3	51	96		BCCN#79-47	Ovine	1979	Alpes-Maritimes, France
3	5	3	13	1	1	3	3	7	43	8	3	4	10	12	3	2018Vergnaud#0521	B. melitensis bv3	51	96		BCCN#82-104	Cattle	1982	Beaufort ,Savoie, France
3	5	3	13	1	1	3	3	7	43	8	8	4	5	11	3	2018Vergnaud#0530	B. melitensis bv3	51	96		BCCN#82-99	Ovine	1982	Puy-Saint-Vincent ,Hau.
3	5	3	13	1	1	3	3	7	43	8	8	4	4	9	3	2018Vergnaud#0126	B. melitensis bv3	51	96		BCCN#80-164	Human	1980	Vendome ,Indre-et-Loire.
3	5	3	13	1	1	3	3	7	43	8	8	4	4	9	3	2018Vergnaud#0508	B. melitensis bv3	51	96		BCCN#80-165	Human	1980	Vendome ,Indre-et-Loire.
3	5	3	13	1	1	3	3	7	43	8	8	4	4	10	3	2018Vergnaud#0964	B. melitensis bv3	51	96		BCCN#80-239	Human	1980	Var, France
3	5	3	13	1	1	3	3	7	43	8	8	4	11	9	3	2018Vergnaud#1324	B. melitensis bv3	51	96		BCCN#92-28	Cattle	1992	Hautes-Alpes, France
3	5	3	13	1	1	3	3	7	43	8	9	4	10	11	3	2018Vergnaud#0230	B. melitensis bv3	51	96		BCCN#89-111	Dog	1989	Isère, France
3	5	3	13	1	1	3	3	7	43	8	9	4	11	11	3	2018Vergnaud#0597	B. melitensis bv3	51	96		BCCN#91-153	Ovine	1991	Vaucluse, France
3	5	3	13	1	1	3	3	7	43	8	9	4	5	12	3	2018Vergnaud#0523	B. melitensis bv3	51	96		BCCN#82-3	Ovine	1982	Bourg d'Oisans ,Isère, .
3	5	3	13	1	1	3	3	7	43	8	10	4	9	7	3	2018Vergnaud#0418	B. melitensis bv3	51	96		BCCN#97-43	Human	1997	Sud Tunisia, Tunisia
3	5	3	13	1	1	3	3	7	43	8	10	4	9	7	3	2018Vergnaud#0419	B. melitensis bv3	51	96		BCCN#97-44	Human	1997	Sud Tunisia, Tunisia
3	5	3	13	1	1	3	3	7	43	8	10	4	9	10	3	2018Vergnaud#0396	B. melitensis bv3	51	96		BCCN#90-165	Dog	1990	Isère, France
3	5	3	13	1	1	3	3	7	43	8	10	4	9	10	3	2018Vergnaud#1074	B. melitensis bv3	51	96		BCCN#90-66	Cattle	1990	Isère, France
3	5	3	13	1	1	3	3	7	43	8	10	4	10	10	3	2018Vergnaud#0947	B. melitensis bv3	51	96		BCCN#89-124	Ovine	1989	Isère, France
3	5	3	13	1	1	3	3	7	43	8	9	4	9	6	3	2007AlDahouk#017	B. melitensis bv3	51	96		BCCN#02-4	Human	2002	Tunisia
3	5	3	13	1	1	3	3	7	43	8	9	4	9	10	3	2018Vergnaud#0590	B. melitensis bv3	51	96		BCCN#90-94	Cattle	1990	Isère, France
3	5	3	13	2	1	3	3	7	43	8	5	4	9	10	3	2017Hanot-Mamb.	B. melitensis bv3	188	366		L3/251	Human	2012	Belgium
3	5	3	13	1	1	3	3	7	43	8	5	4	8	8	3	2018Vergnaud#0181	B. melitensis bv3	51	96		BCCN#91-244	Ovine	1991	Tunisia
3	5	3	13	1	1	3	3	7	43	8	5	4	8	6	3	2018Vergnaud#0911	B. melitensis bv3	51	96		BCCN#90-51	Caprine	1990	Alpes-de-Haute-Proven.
3	5	3	13	1	1	3	3	7	43	8	5	4	4	7	3	2018Vergnaud#0132	B. melitensis bv3	51	96		BCCN#77-167	Ovine	1977	Alpes-Maritimes, France
3	5	3	13	1	1	3	3	7	43	8	5	4	8	7	3	2018Vergnaud#1001	B. melitensis bv3	51	96		BCCN#90-85	Ovine	1990	Var, France
3	5	3	13	1	1	3	3	7	43	8	5	4	7	8	3	2007Marianelli#010	B. melitensis bv3	51	96		PD11	Human	2005	Catania, Italy
3	5	3	13	1	1	3	3	7	43	8	5	4	7	7	3	2018Vergnaud#0502	B. melitensis bv3	51	96		BCCN#78-64	Ovine	1978	Alpes-Maritimes, France
3	5	3	13	1	1	3	3	7	43	8	5	4	7	9	3	2018Vergnaud#0362	B. melitensis R.	51	96		BCCN#93-34	Caprine	1993	Var, France
3	5	3	13	1	1	3	3	7	43	8	5	4	11	12	3	2018Vergnaud#0223	B. melitensis bv3	51	96		BCCN#96-102	Chamois	1996	Savoie, France
3	5	3	13	1	1	3	3	7	43	8	5	4	9	8	3	2018Vergnaud#0064	B. melitensis bv3	51	96		BCCN#91-247	Human	1991	Tunisia
3	5	3	13	1	1	3	3	7	43	8	5	4	9	6	3	2018Vergnaud#1396	B. melitensis bv3	51	96		BCCN#96-66	Ovine	1996	Sicile, Italy
3	5	3	13	1	1	3	3	7	43	8	5	4	9	11	3	2018Vergnaud#0229	B. melitensis bv3	51	96		BCCN#81-111	Deer/El.	1981	Jura, France
3	5	3	13	1	1	3	3	7	43	8	5	5	9	7	3	2007AlDahouk#068	B. melitensis bv2	51	96		BfR 35	Human	2000	Munchen, Germany
3	5	3	13	1	1	3	3	7	43	8	5	4	9	7	3	2018Vergnaud#1268	B. melitensis bv3	51	96		BCCN#90-56	Ovine	1990	Var, France
3	5	3	13	1	1	3	3	7	43	8	5	7	9	8	3	2018Vergnaud#0691	B. melitensis bv3	51	96		BCCN#97-69	Cattle	1997	Sicile, Italy
3	5	3	13	1	1	3	3	7	43	8	9	8	6	11	3	2013Garofolo_15.	B. melitensis bv3	51	96		15082	Ovine	2011	San Giovanni rotondo,It.
3	5	3	13	1	1	3	3	7	43	8	9	8	7	10	3	2013Garofolo_6844	B. melitensis bv3	51	96		6844	Ovine	2011	San Severo,Italy
3	5	3	13	1	1	3	3	7	43	8	6	6	11	8	3	2018Vergnaud#0500	B. melitensis bv3	51	96		BCCN#78-13	Caprine	1978	Haute-Savoie, France
3	5	3	13	1	1	3	3	7	43	8	6	6	5	8	3	2018Vergnaud#1251	B. melitensis bv3	51	96		BCCN#99-71	Ovine	1999	Sicile, Italy
3	5	3	13	1	1	3	3	7	43	8	6	6	10	8	3	2018Vergnaud#0518	B. melitensis bv3	51	96		BCCN#81-44	Ovine	1981	Hautes-Alpes, France
3	5	3	13	1	1	3	3	7	43	8	6	6	11	10	3	2018Vergnaud#0501	B. melitensis bv3	51	96		BCCN#78-14	Caprine	1978	Haute-Savoie, France
3	5	3	13	1	1	3	3	7	43	8	6	6	11	6	3	2018Vergnaud#1209	B. melitensis bv3	51	96		BCCN#96-72	Ovine	1996	Sicile, Italy

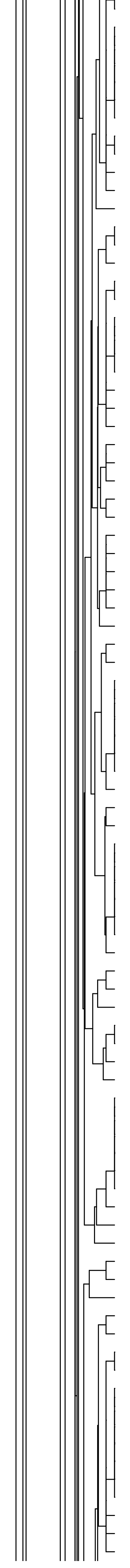

3	5	3	13	1	1	3	3	7	43	8	6	6	11	6	3	2018Vergnaud#1210	B. melitensis bv3	51	96	BCCN#96-73	Ovine	1996	Sicile, Italy
3	5	3	13	1	1	3	3	7	43	8	6	6	5	6	3	2018Vergnaud#0629	B. melitensis bv3	51	96	BCCN#94-33	Human	1994	Rhone, France
3	5	3	13	1	1	3	3	7	43	8	6	6	5	6	3	2018Vergnaud#1000	B. melitensis bv3	51	96	BCCN#89-90	Human	1989	Isère, France
3	5	3	13	1	1	3	3	7	43	8	6	6	5	6	3	2018Vergnaud#1006	B. melitensis bv3	51	96	BCCN#92-21	Cattle	1992	Hautes-Alpes, France
3	5	3	13	1	1	3	3	7	43	8	6	6	5	6	3	2018Vergnaud#1143	B. melitensis bv3	51	96	BCCN#91-276	Cattle	1991	Hautes-Alpes, France
3	5	3	13	1	1	3	3	7	43	8	6	6	5	6	3	2018Vergnaud#1144	B. melitensis bv3	51	96	BCCN#91-277	Cattle	1991	Hautes-Alpes, France
3	5	3	13	1	1	3	3	7	43	8	6	6	5	6	3	2018Vergnaud#1322	B. melitensis bv3	51	96	BCCN#91-282	Cattle	1991	Hautes-Alpes, France
3	5	3	13	1	1	3	3	7	43	8	6	6	6	6	3	2018Vergnaud#0469	B. melitensis bv3	51	96	BCCN#97-45	Human	1997	Ain, France
3	5	3	13	1	1	3	3	7	43	8	6	6	6	6	3	2018Vergnaud#1395	B. melitensis bv3	51	96	BCCN#95-8	Human	1995	Ain, France
3	5	3	13	1	1	3	3	7	43	8	6	6	9	6	3	2018Vergnaud#0509	B. melitensis bv3	51	96	BCCN#80-289	Ovine	1980	Hautes-Alpes, France
3	5	3	13	1	1	3	3	7	43	8	6	6	3	6	3	2018Vergnaud#1321	B. melitensis bv3	51	96	BCCN#91-281	Cattle	1991	Hautes-Alpes, France
3	5	3	13	1	1	3	3	7	49	8	6	6	10	6	3	2018Vergnaud#0483	B. melitensis bv2	51	95	BCCN#76-354	Human	1976	Montpellier ,Hérault, Fr.
3	5	3	13	1	1	3	3	7	43	8	5	6	5	7	3	2018Vergnaud#1156	B. melitensis bv3	51	96	BCCN#89-46	Cattle	1989	Hautes-Alpes, France
3	5	3	13	1	1	3	3	7	43	8	5	6	5	7	3	2018Vergnaud#1157	B. melitensis bv3	51	96	BCCN#89-47	Cattle	1989	Hautes-Alpes, France
3	5	3	13	1	1	3	3	7	43	8	5	6	8	7	3	2018Vergnaud#1092	B. melitensis bv3	51	96	BCCN#97-11	Human	1997	Hautes-Alpes, France
3	5	3	13	1	1	3	3	7	43	8	5	6	8	8	3	2018Vergnaud#1169	B. melitensis bv3	51	96	BCCN#91-249	Cattle	1991	Hautes-Alpes, France
3	5	3	13	1	1	3	3	7	43	8	5	6	8	8	3	2018Vergnaud#1201	B. melitensis bv3	51	96	BCCN#91-168	Cattle	1991	Hautes-Alpes, France
3	5	3	13	1	1	3	3	7	43	8	5	6	11	8	3	2018Vergnaud#0600	B. melitensis bv3	51	96	BCCN#91-189	Cattle	1991	Hautes-Alpes, France
3	5	3	13	1	1	3	3	7	43	8	5	6	11	8	3	2018Vergnaud#0605	B. melitensis bv3	51	96	BCCN#91-9	Cattle	1991	Hautes-Alpes, France
3	5	3	13	1	1	3	3	7	43	8	5	6	11	8	3	2018Vergnaud#1176	B. melitensis bv3	51	96	BCCN#91-6	Cattle	1991	Hautes-Alpes, France
3	5	3	13	1	1	3	3	7	43	8	5	6	11	8	3	2018Vergnaud#1177	B. melitensis bv3	51	96	BCCN#91-7	Cattle	1991	Hautes-Alpes, France
3	5	3	13	1	1	3	3	7	43	8	5	6	7	8	3	2007AlDahouk#104	B. melitensis bv3	51	96	BfR 83	Human	1996	Italy
3	5	3	13	1	1	3	3	7	43	8	5	6	10	8	3	2018Vergnaud#1167	B. melitensis bv3	51	96	BCCN#91-183	Ovine	1991	Alpes-de-Haute-Proven.
3	5	3	13	1	1	3	3	7	43	8	5	6	6	8	3	2018Vergnaud#1397	B. melitensis bv3	51	96	BCCN#96-67	Ovine	1996	Sicile, Italy
3	5	3	13	1	1	3	3	7	43	8	5	6	10	5	3	2017Hanot-Mamb.	B. melitensis R.	51	96	L3/140	Human	2004	Belgium
3	5	3	13	1	1	3	3	7	43	8	5	6	12	5	3	2018Vergnaud#0522	B. melitensis bv3	51	96	BCCN#82-140	Cattle	1982	Thones ,Haute-Savoie, .
3	5	3	13	1	1	3	3	7	43	8	5	6	3	5	3	2018Vergnaud#1098	B. melitensis bv3	51	96	BCCN#99-65	Ovine	1999	Sicile, Italy
3	5	3	13	1	1	3	3	7	43	8	5	6	12	9	3	2007Marianelli#003	B. melitensis bv3	51	96	GV2	Human	2005	Catania, Italy
3	5	3	13	1	1	3	3	7	43	8	5	6	3	9	3	2018Vergnaud#0599	B. melitensis bv3	51	96	BCCN#91-188	Cattle	1991	Hautes-Alpes, France
3	5	3	13	1	1	3	3	7	43	8	5	6	11	6	3	2018Vergnaud#0021	B. melitensis bv2	51	96	BCCN#74-256	Ovine	1974	Nice ,Alpes-Maritimes, .
3	5	3	13	1	1	3	3	7	43	8	5	6	12	6	3	2018Vergnaud#0022	B. melitensis bv2	51	96	BCCN#75-9	Ovine	1975	Alpes-Maritimes, France
3	5	3	13	1	1	3	3	7	43	8	5	6	9	6	3	2018Vergnaud#0510	B. melitensis bv3	51	96	BCCN#80-291	Ovine	1980	Gap ,Hautes-Alpes, Fr.
3	5	3	13	1	1	3	3	7	43	8	5	6	6	6	3	2018Vergnaud#0649	B. melitensis bv3	51	96	BCCN#96-65	Cattle a.	1996	Sicile, Italy
3	5	3	13	1	1	3	3	7	43	8	5	6	8	6	3	2018Vergnaud#1402	B. melitensis R.	51	96	BCCN#99-78	Ovine	1999	Sicile, Italy
3	5	3	13	1	1	3	3	7	43	8	8	6	11	6	3	2018Vergnaud#0097	B. melitensis bv3	51	96	BCCN#79-141	Cattle	1979	Mieussy ,Haute-Savoie,.
3	5	3	13	1	1	3	3	7	43	8	7	6	5	5	3	2018Vergnaud#0586	B. melitensis bv3	51	96	BCCN#90-36	Ovine	1990	Alpes-de-Haute-Proven.
3	5	3	13	1	1	3	3	7	43	8	7	6	4	5	3	2018Vergnaud#1070	B. melitensis bv3	51	96	BCCN#89-50	Ovine	1989	Hautes-Alpes, France
3	5	3	13	1	1	3	3	7	43	8	8	6	4	5	3	2018Vergnaud#0098	B. melitensis bv3	51	96	BCCN#80-375	Cattle	1980	Savoie, France
3	5	3	13	1	1	3	3	7	43	8	8	6	4	5	3	2018Vergnaud#0553	B. melitensis bv3	51	96	BCCN#83-30	Caprine	1983	Montaimont ,Savoie, Fr.
3	5	3	13	1	1	3	3	7	43	8	8	6	4	5	3	2018Vergnaud#0574	B. melitensis bv3	51	96	BCCN#87-6	Cattle	1987	Saint Jean d'Arves ,Sav.
3	5	3	13	1	1	3	3	7	43	8	8	6	4	5	3	2018Vergnaud#0828	B. melitensis bv3	51	96	BCCN#87-13	Cattle	1987	Savoie, France
3	5	3	13	1	1	3	3	7	43	8	8	6	4	5	3	2018Vergnaud#0829	B. melitensis bv3	51	96	BCCN#87-14	Cattle	1987	Savoie, France
3	5	3	13	1	1	3	3	7	43	8	8	6	4	5	3	2018Vergnaud#1073	B. melitensis bv3	51	96	BCCN#90-65	Cattle	1990	Isère, France
3	5	3	13	1	1	3	3	7	43	8	8	6	9	5	3	2018Vergnaud#0575	B. melitensis bv3	51	96	BCCN#87-7	Cattle	1987	Saint Jean d'Arves ,Sav.
3	5	3	13	1	1	3	3	7	43	8	8	6	10	8	3	2018Vergnaud#0532	B. melitensis bv3	51	96	BCCN#83-12	Cattle	1983	Sousville ,Isère, France
3	5	3	13	1	1	3	3	7	43	8	8	6	8	8	3	2018Vergnaud#1196	B. melitensis bv2	51	96	BCCN#74-258	Human	1974	France
3	5	3	13	1	1	3	3	7	43	8	8	6	6	8	3	2007Marianelli#017	B. melitensis bv3	51	96	DN5	Human	2005	Catania, Italy
3	5	3	13	1	1	3	3	7	43	8	8	6	6	8	3	2013Garofolo_4471	B. melitensis bv3	51	96	4471	Ovine	2011	Casteldaccia,Italy
3	5	3	13	1	1	3	3	7	43	8	8	6	6	8	3	2013Garofolo_4474	B. melitensis bv3	51	96	4474	Ovine	2011	Acicatena,Italy
3	5	3	13	1	1	3	3	7	43	8	8	6	6	8	3	2013Garofolo_4478	B. melitensis bv3	51	96	4478	Ovine	2011	Novara di Sicilia,Italy
3	5	3	13	1	1	3	3	7	43	8	8	6	6	8	3	2013Garofolo_4479	B. melitensis bv3	51	96	4479	Ovine	2011	Niscemi,Italy
3	5	3	13	1	1	3	3	7	43	8	8	6	6	8	3	2013Garofolo_4486	B. melitensis bv3	51	96	4486	Ovine	2011	Prizzi,Italy
3	5	3	13	1	1	3	3	7	41	8	8	6	6	8	3	2013Garofolo_4467	B. melitensis bv3	51	212	4467	Ovine	2011	Noto,Italy
3	5	3	13	1	1	3	3	6	43	8	6	6	10	9	3	2018Vergnaud#0601	B. melitensis bv3	51	207	BCCN#91-197	Cattle	1991	Hautes-Alpes, France
3	5	3	13	1	1	3	3	6	43	8	7	6	10	9	3	2018Vergnaud#0969	B. melitensis bv3	51	207	BCCN#85-9	Ovine	1985	Picolard les Chapelles,.
3	5	3	13	1	1	3	3	6	43	8	8	6	10	11	3	2018Vergnaud#1132	B. melitensis bv3	51	207	BCCN#89-40	Ovine	1989	Savoie, France
3	5	3	13	1	1	3	3	6	43	8	6	6	11	11	3	2018Vergnaud#0231	B. melitensis bv3	51	207	BCCN#91-201	Dog	1991	Hautes-Alpes, France
3	5	3	13	1	1	3	3	6	43	8	6	6	11	11	3	2018Vergnaud#0232	B. melitensis bv3	51	207	BCCN#91-202	Dog	1991	Hautes-Alpes, France
3	5	3	13	1	1	3	3	7	43	8	6	6	11	11	3	2018Vergnaud#0023	B. melitensis bv2	51	96	BCCN#76-328	Human	1976	Grenoble ,Haute-Savoie.
3	5	3	13	1	1	3	3	6	43	8	7	6	11	11	3	2018Vergnaud#1133	B. melitensis bv3	51	207	BCCN#89-41	Cattle	1989	Savoie, France
3	5	3	13	1	1	3	3	7	43	8	6	3	11	5	3	2013Garofolo_1994	B. melitensis bv3	51	96	1994	Ovine	2011	Montecorvino Pugliano,.
3	5	3	13	1	1	3	3	7	43	8	6	3	11	5	3	2013Garofolo_2461	B. melitensis bv3	51	96	2461	Ovine	2011	Montecorvino Pugliano,.
3	5	3	13	1	1	3	3	7	43	8	6	3	11	5	3	2013Garofolo_6299	B. melitensis bv3	51	96	6299	Human	2011	Salerno,Italy
3	5	3	13	1	1	3	3	7	43	8	6	3	11	5	3	2017Hanot-Mamb.	B. melitensis bv3	51	96	L3/295	Human	2015	Belgium
3	5	3	13	1	1	3	3	7	43	8	6	3	11	5	3	2017Hanot-Mamb.	B. melitensis bv3	51	96	L3/296	Human	2015	Belgium
3	5	3	13	1	1	3	3	7	43	8	6	3	11	5	3	2017Hanot-Mamb.	B. melitensis bv3	51	96	L3/297	Human	2015	Belgium
3	5	3	13	1	1	3	3	7	43	8	5	3	11	5	3	2013Garofolo_22.	B. melitensis bv3	51	96	22840	Ovine	2011	Montecorvino Pugliano,.
3	5	3	13	1	1	3	3	7	43	8	6	3	10	5	2	2013Garofolo_3922	B. melitensis bv3	51	96	3922	Caprine	2011	Montecorvino Pugliano,.
3	5	3	13	1	1	3	3	6	43	8	6	9	11	5	3	2013Garofolo_11.	B. melitensis bv3	51	207	11789	Ovine	2011	Santa Croce Camerina,.
3	5	3	13	1	1	3	3	7	49	8	5	6	9	7	3	2018Vergnaud#0101	B. melitensis bv3	51	95	BCCN#82-73	Human	1982	Toulouse ,Haute-Garo.
3	5	3	13	1	1	3	3	7	49	8	8	6	9	7	3	2018Vergnaud#1043	B. melitensis bv3	51	95	BCCN#86-61	Ovine	1986	Tarn-et-Garonne, France
3	5	3	13	1	1	3	3	7	49	8	10	8	14	7	3	2018Vergnaud#0528	B. melitensis bv3	51	95	BCCN#79-133	Ovine	1979	Haute-Vienne, France
3	5	3	13	1	1	3	3	7	49	8	9	5	10	5	3	2018Vergnaud#0379	B. melitensis bv3	51	95	BCCN#81-109	Human	1981	Landen ,Province Du L.
3	5	3	13	1	1	3	3	7	49	8	8	5	10	5	3	2018Vergnaud#0927	B. melitensis bv3	51	95	BCCN#77-47	Human	1977	Bouches-du-Rhone, Fr.
3	5	3	13	1	1	3	3	7	49	8	6	5	13	5	3	2018Vergnaud#1385	B. melitensis bv3	51	95	BCCN#89-10	Cattle	1989	Savoie, France
3	5	3	13	1	1	3	3	7	49	8	6	5	13	5	3	2018Vergnaud#1386	B. melitensis bv3	51	95	BCCN#89-102	Ovine	1989	Haute-Savoie, France
3	5	3	13	1	1	3	3	7	49	8	6	5	12	5	3	2018Vergnaud#0603	B. melitensis bv3	51	95	BCCN#91-253	Cattle	1991	Savoie, France
3	5	3	13	1	1	3	3	7	49	8	6	5	12	5	3	2018Vergnaud#0970	B. melitensis bv3	51	95	BCCN#89-117	Cattle	1989	Savoie, France
3	5	3	13	1	1	3	3	7	49	8	6	5	12	5	3	2018Vergnaud#0971	B. melitensis bv3	51	95	BCCN#89-118	Cattle	1989	Savoie, France
3	5	3	13	1	1	3	3	7	49	8	6	5	12	5	3	2018Vergnaud#0972	B. melitensis bv3	51	95	BCCN#89-119	Cattle	1989	Savoie, France
3	5	3	13	1	1	3	3	7	49	8	6	5	12	5	3	2018Vergnaud#1134	B. melitensis bv3	51	95	BCCN#90-19	Cattle	1990	Savoie, France
3	5	3	13	1	1	3	3	7	49	8	6	5	12	5	3	2018Vergnaud#1136	B. melitensis bv3	51	95	BCCN#90-22	Cattle	1990	Savoie, France
3	5	3	13	1	1	3	3	7	49	8	6	5	12	5	3	2018Vergnaud#1204	B. melitensis bv3	51	95	BCCN#91-173	Cattle	1991	Savoie, France
3	5	3	13	1	1	3	3	7	49	8	6	5	10	5	3	2018Vergnaud#0996	B. melitensis bv3	51	95	BCCN#88-50	Cattle	1988	Savoie, France
3	5	3	13	1	1	3	3	7	49	8	6	5	8	5	3	2018Vergnaud#1124	B. melitensis bv3	51	95	BCCN#93-1	Human	1993	Bouches-du-Rhone, Fr.
3	5	3	13	1	1	3	3	7	49	8	6	5	11	5	3	2018Vergnaud#1131	B. melitensis bv3	51	95	BCCN#88-20	Cattle	1988	Savoie, France

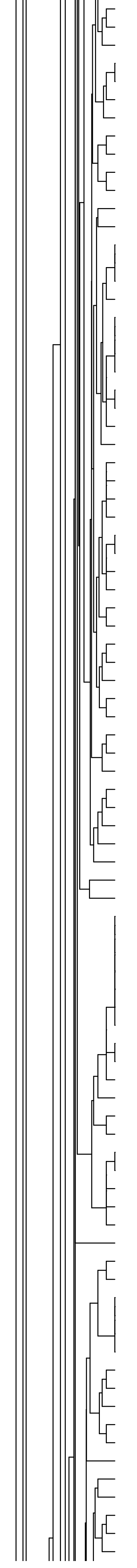

3	5	3	13	1	1	3	3	7	49	8	7	5	7	5	3	2018Vergnaud#0123	B. melitensis bv3	51	95		BCCN#79-49	Ovine	1979	Alpes-de-Haute-Proven.
3	5	3	13	1	1	3	3	7	49	8	7	5	14	5	3	2018Vergnaud#1159	B. melitensis bv3	51	95		BCCN#90-62	Ovine	1990	Petit-Bornand ,Haute-S.
3	5	3	13	1	1	3	3	7	49	8	5	5	14	5	3	2018Vergnaud#1160	B. melitensis bv3	51	95		BCCN#90-63	Ovine	1990	Petit-Bornand ,Haute-S.
3	5	3	13	1	1	3	3	7	49	8	6	5	9	4	3	2018Vergnaud#0099	B. melitensis bv3	51	95		BCCN#81-253	Cattle	1981	Notre Dame de Bellec.
3	5	3	13	1	1	3	3	7	49	8	6	5	9	4	3	2018Vergnaud#0552	B. melitensis bv3	51	95		BCCN#83-3	Ovine	1983	Saint-Offenge Dessous.
3	5	3	13	1	1	3	3	7	49	8	6	5	8	4	3	2018Vergnaud#0556	B. melitensis bv3	51	95		BCCN#84-5	Cattle	1984	Corse-du-Sud, France
3	5	3	13	1	1	3	3	7	49	8	6	5	9	8	3	2018Vergnaud#0998	B. melitensis bv3	51	95		BCCN#89-86	Ovine	1989	Saragosse, Spain
3	5	3	13	1	1	3	3	7	49	8	12	5	10	10	3	2018Vergnaud#0558	B. melitensis bv3	51	95		BCCN#85-11	Ovine	1985	Charente, France
3	5	3	13	1	1	3	3	7	49	8	6	5	10	10	3	2018Vergnaud#0942	B. melitensis bv3	51	95		BCCN#76-320	Human	1976	Haute-Garonne, France
3	5	3	13	1	1	3	3	7	49	8	8	5	9	10	3	2018Vergnaud#0524	B. melitensis bv3	51	95		BCCN#82-48	Human	1982	Toulouse ,Haute-Garo.
3	5	3	13	1	1	3	3	7	49	8	9	5	9	10	3	2018Vergnaud#0943	B. melitensis bv3	51	95		BCCN#76-326	Human	1976	Toulouse ,Haute-Garo.
3	5	3	13	1	1	3	3	7	49	8	11	5	7	8	3	2018Vergnaud#0533	B. melitensis bv3	51	95		BCCN#83-139	Cattle	1983	Indre, France
3	5	3	13	1	1	3	3	7	49	8	11	5	8	11	3	2018Vergnaud#1075	B. melitensis bv3	51	95		BCCN#91-57	Ovine	1991	Huesca, Spain
3	5	3	13	1	1	3	3	7	49	8	10	5	8	8	3	2018Vergnaud#0067	B. melitensis bv3	51	95		BCCN#96-101	Swine	1996	Indre, France
3	5	3	13	1	1	3	3	7	49	8	10	5	8	8	3	2018Vergnaud#0820	B. melitensis bv3	51	95		BCCN#81-134	Human	1981	Haute-Garonne, France
3	5	3	13	1	1	3	3	7	49	8	10	5	8	8	3	2018Vergnaud#1085	B. melitensis bv3	51	95		BCCN#96-111	Human	1996	Indre, France
3	5	3	13	1	1	3	3	7	49	8	6	5	8	8	3	2018Vergnaud#0999	B. melitensis bv3	51	95		BCCN#89-87	Ovine	1989	Saragosse, Spain
3	5	3	13	1	1	3	3	7	49	8	7	5	8	8	3	2018Vergnaud#1076	B. melitensis bv3	51	95		BCCN#91-58	Ovine	1991	Huesca, Spain
3	5	3	13	1	1	3	3	7	49	8	7	5	8	8	3	2018Vergnaud#1077	B. melitensis bv3	51	95		BCCN#91-59	Ovine	1991	Huesca, Spain
3	5	3	13	1	1	3	3	7	49	8	7	5	8	8	3	2018Vergnaud#1304	B. melitensis bv3	51	95		BCCN#91-176	Cattle	1991	Haute-Garonne, France
3	5	3	13	1	1	3	3	7	49	8	7	5	8	8	3	2018Vergnaud#1305	B. melitensis bv3	51	95		BCCN#91-177	Cattle	1991	Haute-Garonne, France
3	5	3	13	1	1	3	3	7	49	8	7	5	10	8	3	2018Vergnaud#0585	B. melitensis bv3	51	95		BCCN#90-33	Cattle	1990	Hautes-Pyrénées, France
3	5	3	13	1	1	3	3	7	49	8	7	5	10	8	3	2018Vergnaud#1002	B. melitensis bv3	51	95		BCCN#90-86	Cattle	1990	Hautes-Pyrénées, France
3	5	3	13	1	1	3	3	7	49	8	7	5	9	8	3	2018Vergnaud#1384	B. melitensis bv3	51	95		BCCN#88-24	Cattle	1988	Mens ,Isère, France
3	5	3	13	1	1	3	3	7	49	8	7	5	8	10	3	2018Vergnaud#0557	B. melitensis bv3	51	95		BCCN#85-1	Cattle	1985	Hautes-Pyrénées,France
3	5	3	13	1	1	3	3	7	49	8	7	5	14	7	3	2006LeFleche#083	B. melitensis bv2	51	95		BCCN#80-39	Ovine	1980	Digne-les-Bains, France
3	5	3	13	1	1	3	3	7	49	8	7	5	9	7	3	2018Vergnaud#0049	B. melitensis bv3	51	95		BCCN#88-25	Cattle	1988	Mens ,Isère, France
3	5	3	13	1	1	3	3	7	49	8	7	5	6	7	3	2018Vergnaud#0498	B. melitensis bv3	51	95		BCCN#76-418	Ovine	1976	Alpes-Maritimes, France
3	5	3	13	1	1	3	3	7	49	8	7	5	11	7	3	2018Vergnaud#0739	B. melitensis bv3	51	95		BCCN#88-32	Cattle	1988	Hautes-Alpes, France
3	5	3	13	1	1	3	3	7	49	8	7	5	10	7	3	2018Vergnaud#0623	B. melitensis bv3	51	95		BCCN#93-56	Human	1993	Marte Tolosanne ,Haut.
3	5	3	13	1	1	3	3	7	49	8	7	5	10	7	3	2018Vergnaud#1279	B. melitensis bv3	51	95		BCCN#93-69	Human	1993	Haute-Garonne, France
3	5	3	13	1	1	3	3	7	49	8	7	5	10	5	3	2018Vergnaud#1389	B. melitensis bv3	51	95		BCCN#90-167	Ovine	1990	Pyrénées-Orientales, F.
3	5	3	13	1	1	3	3	7	49	8	7	5	10	10	3	2018Vergnaud#1195	B. melitensis bv3	51	95		BCCN#89-138	Ovine	1989	Saragosse, Spain
3	5	3	13	1	1	3	3	7	49	8	7	5	6	9	3	2018Vergnaud#1009	B. melitensis bv3	51	95		BCCN#92-79	Ovine	1992	Aragon, Spain
3	5	3	13	1	1	3	3	7	49	8	7	5	9	9	3	2018Vergnaud#1269	B. melitensis bv3	51	95		BCCN#90-59	Cattle	1990	Hautes-Pyrénées, France
3	5	3	13	1	1	3	3	7	49	8	10	5	6	7	3	2018Vergnaud#0506	B. melitensis bv3	51	95		BCCN#79-134	Ovine	1979	Haute-Vienne, France
3	5	3	13	1	1	3	3	7	49	8	8	5	6	7	3	2018Vergnaud#0635	B. melitensis bv3	51	95		BCCN#96-125	Human	1996	Hérault, France
3	5	3	13	1	1	3	3	7	49	8	10	5	10	7	3	2018Vergnaud#0634	B. melitensis bv3	51	95		BCCN#96-124	Cattle	1996	Indre, France
3	5	3	13	1	1	3	3	7	49	8	9	5	10	7	3	2018Vergnaud#0409	B. melitensis bv3	51	95		BCCN#91-14	Ovine	1991	Hautes-Pyrénées, France
3	5	3	13	1	1	3	3	7	49	8	9	5	7	7	3	2018Vergnaud#1005	B. melitensis bv3	51	95		BCCN#91-304	Cattle	1991	Hautes-Pyrénées, France
3	5	3	13	1	1	3	3	7	49	8	7	5	11	6	3	2018Vergnaud#0963	B. melitensis bv3	51	95		BCCN#77-149	Human	1977	Paris, France
3	5	3	13	1	1	3	3	7	49	8	8	5	11	6	3	2018Vergnaud#1181	B. melitensis bv3	51	95		BCCN#93-47	Human	1993	Cap Vernes Les Bains .
3	5	3	13	1	1	3	3	7	49	8	8	5	12	6	3	2018Vergnaud#1044	B. melitensis bv3	51	95		BCCN#86-8	Cattle	1986	Hautes-Pyrénées, France
3	5	3	13	1	1	3	3	7	49	8	10	5	7	9	3	2018Vergnaud#0391	B. melitensis R.	51	95		BCCN#82-66	Human	1982	San Lazaro ,Spain
3	5	3	13	1	1	3	3	7	49	8	6	5	7	9	3	2018Vergnaud#0949	B. melitensis bv3	51	95		BCCN#89-83	Ovine	1989	Saragosse, Spain
3	5	3	13	1	1	3	3	7	49	8	6	5	11	9	3	2018Vergnaud#0569	B. melitensis bv3	51	95		BCCN#87-39	Human	1987	Vienne, France
3	5	3	13	1	1	3	3	7	49	8	13	5	10	9	3	2018Vergnaud#0907	B. melitensis bv3	51	95		BCCN#87-38	Human	1987	Vienne, France
3	5	3	13	1	1	3	3	7	49	7	5	5	7	9	3	2010Valdezate#083	B. melitensis	51	161		#20071672	Human	2007	Girona, Spain
3	5	3	13	1	1	3	3	7	49	8	9	5	7	6	5	2018Vergnaud#0414	B. melitensis bv3	51	95		BCCN#94-61	Human	1994	Gers ,Haute-Garonne, .
3	5	3	13	1	1	3	3	7	49	8	6	4	8	6	5	2018Vergnaud#0481	B. melitensis bv2	51	95		BCCN#74-243	Ovine	1974	Nice ,Alpes-Maritimes, .
3	5	3	13	1	1	3	3	9	43	8	5	5	3	7	3	2018Vergnaud#0407	B. melitensis bv3	51	92		BCCN#03-19	Human	2003	Algeria
3	5	3	13	1	1	3	3	9	43	8	5	5	3	7	3	2018Vergnaud#0639	B. melitensis bv3	51	92		BCCN#96-151	Human	1996	Algeria
3	5	3	13	1	1	3	3	9	43	8	5	5	3	7	3	2018Vergnaud#0640	B. melitensis bv3	51	92		BCCN#96-153	Human	1996	Algeria
3	5	3	13	1	1	3	3	9	43	8	5	5	3	7	3	2018Vergnaud#0912	B. melitensis bv3	51	92		BCCN#96-154	Human	1996	Algeria
3	5	3	13	1	1	3	3	9	43	8	5	5	3	7	3	2018Vergnaud#1047	B. melitensis bv3	51	92		BCCN#91-242	Human	1991	Moselle, France
3	5	3	13	1	1	3	3	9	43	8	5	5	3	7	3	2018Vergnaud#1066	B. melitensis bv3	51	92		BCCN#00-25	Human	2000	Doubs, France
3	5	3	13	1	1	3	3	9	43	8	5	5	3	7	3	2018Vergnaud#1393	B. melitensis bv3	51	92		BCCN#93-49	Human	1993	Val-d'Oise, France
3	5	3	13	1	1	3	3	9	43	8	5	5	3	10	3	2007AlDahouk#019	B. melitensis bv3	51	92		BCCN#02-6	Human	2002	Tunisia
3	5	3	13	1	1	3	3	9	43	8	5	5	3	10	3	2018Vergnaud#0068	B. melitensis bv3	51	92		BCCN#04-31	Human	2004	Sfax,Tunisia
3	5	3	13	1	1	3	3	9	43	8	5	5	3	9	3	2007AlDahouk#018	B. melitensis bv3	51	92		BCCN#02-5	Human	2002	Tunisia
3	5	3	13	1	1	3	3	9	43	8	4	5	3	6	3	2018Vergnaud#0619	B. melitensis bv3	51	92		BCCN#93-16	Human	1993	Tunisia
3	5	3	13	1	1	3	3	9	43	8	6	6	3	5	3	2007AlDahouk#025	B. melitensis	51	92		BCCN#96-147	Human	1996	Algeria
3	5	3	13	1	1	3	3	9	43	8	6	5	3	5	3	2018Vergnaud#0614	B. melitensis bv3	51	92		BCCN#92-46	Human	1992	Sfax ,Tunisia
3	5	3	13	1	1	3	3	9	43	8	7	4	3	7	3	2018Vergnaud#0620	B. melitensis bv3	51	92		BCCN#93-50	Human	1993	Tunisia
3	5	3	13	1	1	3	3	9	43	8	7	4	3	7	3	2018Vergnaud#0630	B. melitensis bv3	51	92		BCCN#94-4	Human	1994	Tunisia
3	5	3	13	1	1	3	3	9	43	8	7	4	3	10	3	2007AlDahouk#016	B. melitensis bv3	51	92		BCCN#02-3	Human	2002	Tunisia
3	5	3	13	1	1	3	3	9	43	8	7	4	3	9	3	2018Vergnaud#0058	B. melitensis bv3	51	92		BCCN#04-32	Human	2004	Sfax,Tunisia
3	5	3	13	1	1	3	3	9	43	8	7	4	3	8	3	2018Vergnaud#0621	B. melitensis bv3	51	92		BCCN#93-51	Human	1993	Tunisia
3	5	3	13	1	1	3	2	7	43	8	5	4	8	11	3	2018Vergnaud#0208	B. melitensis R.	124	94		BCCN#76-416	Human	1976	France
3	5	3	13	1	1	3	3	8	43	8	7	6	12	4	3	2013Garofolo_4498	B. melitensis bv3	51	91		4498	Ovine	2011	Roccafiorita,Italy
3	5	3	13	1	1	3	3	8	43	8	7	6	11	4	3	2013Garofolo_6058	B. melitensis bv3	51	91		6058	Caprine	2011	San Luca,Italy
3	5	3	13	1	1	3	3	8	43	8	7	6	10	5	3	2013Garofolo_11.	B. melitensis bv3	51	91		11810	Caprine	2011	Scicli,Italy
3	5	3	13	1	1	3	3	8	43	8	7	6	10	5	3	2013Garofolo_11.	B. melitensis bv3	51	91		11814	Ovine	2011	Scicli,Italy
3	5	3	13	1	1	3	3	8	43	8	7	6	10	5	3	2013Garofolo_4480	B. melitensis bv3	51	91		4480	Ovine	2011	Montalbano Elicona,Italy
3	5	3	13	1	1	3	3	8	43	8	7	6	10	5	3	2013Garofolo_4488	B. melitensis bv3	51	91		4488	Caprine	2011	Montalbano Elicona,Italy
3	5	3	13	1	1	3	3	6	43	8	7	5	3	5	3	2018Vergnaud#0913	B. melitensis bv3	51	207		BCCN#81-140	Human	1981	Corse-du-Sud, France
3	5	3	13	1	1	3	3	8	43	8	7	5	3	5	3	2018Vergnaud#1099	B. melitensis bv3	51	91		BCCN#99-66	Ovine	1999	Sicile, Italy
3	5	3	13	1	1	3	3	8	43	8	7	5	3	8	3	2018Vergnaud#1283	B. melitensis bv3	51	91		BCCN#94-68	Human	1994	Vaucluse, France
3	5	3	13	1	1	3	3	8	43	8	7	5	3	6	3	2018Vergnaud#0642	B. melitensis bv3	51	91		BCCN#96-33	Human	1996	Mougins ,Alpes-Mariti.
3	5	3	13	1	1	3	3	8	43	8	7	5	5	6	3	2018Vergnaud#1086	B. melitensis bv3	51	91		BCCN#96-12	Human	1996	Alpes-Maritimes, France
3	5	3	13	1	1	3	3	8	43	8	9	6	8	6	3	2018Vergnaud#0235	B. melitensis bv3	51	91		BCCN#92-65	Cat	1992	Vaucluse, France
3	5	3	13	1	1	3	3	8	43	8	5	7	8	5	3	2007AlDahouk#049	B. melitensis bv2	51	91		BfR 1	Human	1999	Bern, Switzerland
3	5	3	13	1	1	3	3	8	43	8	5	9	5	5	3	GCA_001715485	B. melitensis	51	91	89	2008724259	unknown		
3	5	3	13	1	1	3	3	8	43	8	4	8	10	5	3	2007Marianelli#013	B. melitensis bv3	51	91		SP4	Human	2005	Catania, Italy
3	5	3	13	1	1	3	3	8	43	8	5	8	10	5	3	2018Vergnaud#0611	B. melitensis bv3	51	91		BCCN#92-41	Human	1992	Moselle, France
3	5	3	13	1	1	3	3	8	43	8	5	8	9	5	3	2013Garofolo_13.	B. melitensis bv3	51	91		13545	Cattle	2011	Barcellona Pozzo di Go.

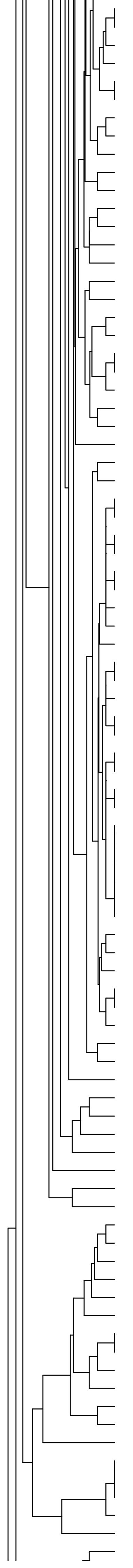

3	5	3	13	1	1	3	3	8	43	8	6	8	8	5	3	2017Hanot-Mamb.	B. melitensis bv3	51	91		L3/15	Human	1997	Belgium
3	5	3	13	1	1	3	3	8	43	8	6	8	8	5	3	2018Vergnaud#0682	B. melitensis bv3	51	91		BCCN#99-52	Ovine	1999	Sicile, Italy
3	5	3	13	1	1	3	3	8	43	8	6	6	8	5	3	2013Garofolo_11.	B. melitensis bv3	51	91		11803	Caprine	2011	Montalbano Elicona,Italy
3	5	3	13	1	1	3	3	8	43	8	6	8	13	5	3	2013Garofolo_4491	B. melitensis bv3	51	91		4491	Ovine	2011	San Pier Niceto,Italy
3	5	3	13	1	1	3	3	8	43	8	6	6	9	5	3	2013Garofolo_11.	B. melitensis bv3	51	91		11805	Cattle	2011	Floresta,Italy
3	5	3	13	1	1	3	3	8	43	8	6	6	9	5	3	2013Garofolo_11.	B. melitensis bv3	51	91		11844	Ovine	2011	Montalbano Elicona,Italy
3	5	3	13	1	1	3	3	8	43	8	4	13	9	5	3	2013Garofolo_11.	B. melitensis bv3	51	91		11828	Cattle	2011	Montalbano Elicona,Italy
3	5	3	13	1	1	3	3	8	43	8	4	14	9	5	3	2013Garofolo_13.	B. melitensis bv3	51	91		13517	Ovine	2011	Castroreale,Italy
3	5	3	13	1	1	3	3	6	43	8	4	6	9	5	3	2018Vergnaud#0134	B. melitensis bv3	51	207		BCCN#78-267	Human	1978	Bouches-du-Rhone, Fr.
3	5	3	13	1	1	3	3	8	43	8	6	13	6	4	3	2018Vergnaud#0375	B. melitensis bv3	51	91		BCCN#03-20	Human	2003	Egypt
3	5	3	13	1	1	3	3	8	43	8	6	6	5	4	3	SRR4436588	B. melitensis	51	91	89	BwIM_XXX_57	Human	2017	
3	5	3	13	1	1	3	3	8	43	8	4	7	11	9	3	2007Marianelli#008	B. melitensis bv3	51	91		AM12	Human	2005	Catania, Italy
3	5	3	13	1	1	3	3	8	43	8	7	7	11	5	3	2018Vergnaud#1019	B. melitensis bv3	51	91		BCCN#99-63	Ovine	1999	Sicile, Italy
3	5	3	13	1	1	3	3	8	43	8	8	5	11	7	3	2018Vergnaud#1291	B. melitensis bv3	51	91		BCCN#97-72	Ovine	1997	Sicile, Italy
3	5	3	13	1	1	3	3	8	43	8	5	7	14	7	3	2013Garofolo_13.	B. melitensis bv3	51	91		13548	Ovine	2011	San Pier Niceto,Italy
3	6	3	13	1	1	3	3	8	43	8	7	4	6	10	3	2007Marianelli#016	B. melitensis bv3	88	155		GR6	Human	2005	Catania, Italy
3	5	3	13	1	1	3	3	8	43	8	4	4	3	10	3	2018Vergnaud#1401	B. melitensis bv3	51	91		BCCN#99-76	Ovine	1999	Sicile, Italy
3	5	3	13	1	1	3	3	8	43	8	6	4	10	6	3	2007AlDahouk#083	B. melitensis bv2	51	91		BfR 59	Human	2004	Italy
3	5	3	13	1	1	3	3	8	43	8	6	4	10	8	3	2018Vergnaud#0593	B. melitensis bv3	51	91		BCCN#91-13	Human	1991	Paris, France
3	6	3	13	1	1	3	3	8	43	8	6	4	9	8	3	2013Garofolo_11.	B. melitensis bv3	88	155		11815	Ovine	2011	San Pier Niceto,Italy
3	6	3	13	1	1	3	3	8	43	8	6	4	9	8	3	2013Garofolo_11.	B. melitensis bv3	88	155		11821	Ovine	2011	Santa Lucia del Mela,Italy
3	6	3	13	1	1	3	3	8	43	8	6	4	9	9	3	2013Garofolo_4496	B. melitensis bv3	88	155		4496	Ovine	2011	Scicli,Italy
3	6	3	13	1	1	3	3	8	43	8	9	4	7	8	3	2013Garofolo_4484	B. melitensis bv3	88	155		4484	Ovine	2011	Ravanusa,Italy
3	5	3	13	1	1	3	3	8	43	8	9	4	8	8	3	2018Vergnaud#0416	B. melitensis bv3	51	91		BCCN#96-146	Human	1996	Algeria
3	6	3	13	1	1	3	3	8	43	8	5	5	12	12	3	2013Garofolo_13.	B. melitensis bv3	88	155		13540	Ovine	2011	Mirabella Imbaccari,Italy
3	5	3	13	1	1	3	3	7	45	8	6	5	6	4	3	2018Vergnaud#0397	B. melitensis bv3	51	94		BCCN#90-27	Human	1990	Algeria
3	5	3	13	1	1	3	3	7	45	8	6	2	6	6	3	SRR4436596	B. melitensis	51	94		BwIM_MAR_25	Human	2017	Morocco
3	5	3	13	1	1	3	3	7	45	8	7	2	10	4	3	2018Vergnaud#1078	B. melitensis bv3	51	94		BCCN#93-13	Human	1993	Paris, France
3	5	3	13	1	1	3	3	7	45	8	7	2	10	4	3	2018Vergnaud#1182	B. melitensis bv3	51	94		BCCN#93-61	Human	1993	Paris, France
3	5	3	13	1	1	3	3	7	45	8	7	2	7	4	3	2018Vergnaud#0742	B. melitensis bv3	51	94		BCCN#90-24	Human	1990	Algeria
3	5	3	13	1	1	3	3	7	45	8	7	2	7	4	3	2018Vergnaud#0749	B. melitensis bv3	51	94		BCCN#90-23	Human	1990	Algeria
3	5	3	13	1	1	3	3	7	45	8	7	2	9	4	3	2018Vergnaud#0417	B. melitensis bv3	51	94		BCCN#96-149	Human	1996	Algeria
3	5	3	13	1	1	3	3	7	45	8	7	2	9	4	3	2018Vergnaud#0638	B. melitensis bv3	51	94		BCCN#96-150	Human	1996	Algeria
3	5	3	13	1	1	3	3	7	45	8	7	2	13	4	3	2007AlDahouk#021	B. melitensis bv3	51	94		BCCN#03-13	Human	2003	Nice, France
3	5	3	13	1	1	3	3	7	45	8	7	2	12	4	3	2018Vergnaud#0607	B. melitensis bv3	51	94		BCCN#92-118	Human	1992	Sfax ,Tunisia
3	5	3	13	1	1	3	3	8	45	8	7	2	7	4	3	2007AlDahouk#024	B. melitensis	51	90		BCCN#96-144	Human	1996	Algeria
3	5	3	13	1	1	3	3	7	45	8	6	2	5	4	3	2018Vergnaud#0365	B. melitensis R.	51	94		BCCN#96-145	Human	1996	Algeria
3	5	3	13	1	1	3	3	7	45	8	6	2	5	4	3	2018Vergnaud#0609	B. melitensis bv3	51	94		BCCN#92-121	Human	1992	Sfax ,Tunisia
3	5	3	13	1	1	3	3	7	45	8	6	2	10	4	3	2018Vergnaud#0065	B. melitensis bv3	51	94		BCCN#91-248	Ovine	1991	Tunisia
3	5	3	13	1	1	3	3	7	45	8	6	2	9	4	3	2006LeFleche#087	B. melitensis bv3	51	94		BCCN#91-243	Ovine	1991	Tunisia
3	5	3	13	1	1	3	3	7	45	8	6	2	9	4	3	2018Vergnaud#0643	B. melitensis bv3	51	94		BCCN#96-40	Human	1996	France
3	5	3	13	1	1	3	3	7	45	8	5	2	7	4	3	2018Vergnaud#0410	B. melitensis bv3	51	94		BCCN#93-37	Human	1993	Tunisia
3	5	3	13	1	1	3	3	7	45	8	5	2	7	4	3	2018Vergnaud#0608	B. melitensis bv3	51	94		BCCN#92-120	Human	1992	Sfax ,Tunisia
3	5	3	13	1	1	3	3	7	45	8	8	2	7	4	3	2018Vergnaud#0398	B. melitensis bv3	51	94		BCCN#90-28	Human	1990	Algeria
3	5	3	13	1	1	3	3	7	45	8	8	2	7	4	3	2018Vergnaud#0399	B. melitensis bv3	51	94		BCCN#90-29	Ovine	1990	Algeria
3	5	3	13	1	1	3	3	7	45	8	6	2	7	4	3	2006LeFleche#096	B. melitensis bv3	51	94		BCCN#92-106a	unknown	1992	Algeria
3	5	3	13	1	1	3	3	7	45	8	6	2	7	4	3	2018Vergnaud#0100	B. melitensis bv3	51	94		BCCN#82-22	Human	1982	Paris, France
3	5	3	13	1	1	3	3	7	45	8	6	2	7	4	3	2018Vergnaud#0582	B. melitensis bv3	51	94		BCCN#90-26	Human	1990	Algeria
3	5	3	13	1	1	3	3	7	45	8	6	2	7	4	3	2018Vergnaud#0583	B. melitensis bv3	51	94		BCCN#90-30	Caprine	1990	Algeria
3	5	3	13	1	1	3	3	7	45	8	6	2	7	4	3	2018Vergnaud#0604	B. melitensis bv3	51	94		BCCN#91-254	Human	1991	Paris, France
3	5	3	13	1	1	3	3	7	45	8	6	2	7	4	3	2018Vergnaud#0743	B. melitensis bv3	51	94		BCCN#90-25	Human	1990	Algeria
3	5	3	13	1	1	3	3	7	45	8	8	2	9	4	3	2007AlDahouk#022	B. melitensis bv3	51	94		BCCN#96-148	Human	1996	Algeria
3	5	3	13	1	1	3	3	7	45	8	8	2	8	4	3	SRR4436604	B. melitensis	51	94		BwIM_DZA_50	Human	2017	Algeria
3	5	3	13	1	1	3	3	7	45	8	10	2	8	4	3	2018Vergnaud#0360	B. melitensis R.	51	94		BCCN#92-119	Human	1992	Sfax ,Tunisia
3	5	3	13	1	1	3	3	7	45	8	5	2	6	4	3	2018Vergnaud#0063	B. melitensis bv3	51	94		BCCN#91-246	Ovine	1991	Tunisia
3	5	3	13	1	1	3	3	7	45	8	5	2	6	4	3	2018Vergnaud#1280	B. melitensis bv3	51	94		BCCN#94-31	Human	1994	Rhone, France
3	5	3	13	1	1	3	3	7	45	8	5	2	8	4	3	2018Vergnaud#1079	B. melitensis bv3	51	94		BCCN#93-14	Human	1993	Hautes-Alpes, France
3	5	3	13	1	1	3	3	9	45	8	5	2	6	5	3	2010Valdezate#093	B. melitensis	51	153		#20091040	Human	2009	Leon, Spain
3	5	3	13	1	1	3	3	7	45	8	5	2	10	5	3	2018Vergnaud#1242	B. melitensis bv3	51	94		BCCN#94-27	Human	1994	Paris, France
2	5	3	13	1	1	3	3	5	43	8	5	5	13	9	3	2018Vergnaud#0121	B. melitensis bv3	110	308		BCCN#75-216	Human	1975	France
3	5	3	13	1	1	3	3	5	41	7	6	4	13	4	3	2010Valdezate#068	B. melitensis	51	158		#20040082	Human	2004	Barcelona, Spain
3	5	3	13	1	1	3	3	5	49	7	6	4	5	8	3	2010Valdezate#073	B. melitensis	51	160		#20000326	Human	2000	Cordoba, Spain
3	5	3	13	1	1	3	3	7	41	7	4	4	3	4	3	2010Valdezate#075	B. melitensis	51	157		#20040020	Human	2004	Barcelona, Spain
3	5	3	13	1	1	3	3	7	41	7	3	6	8	7	3	2010Valdezate#077	B. melitensis	51	157		#20050707	Human	2005	Madrid, Spain
3	5	3	13	1	1	3	3	7	45	9	3	3	4	6	1	2010Valdezate#100	B. melitensis	51	156		#20091419	Human	2009	Alava, Spain
1	5	3	13	1	1	1	3	7	9	8	8	4	10	4	4	2018Vergnaud#0576	B. melitensis bv3	136	305		BCCN#87-75	Cattle	1987	Cervieres ,Hautes-Alpes.
1	5	3	13	1	1	3	3	8	9	8	8	5	6	6	4	2018Vergnaud#0577	B. melitensis bv3	125	304		BCCN#89-27	Caprine	1989	Vaucluse, France
1	2	3	12	1	2	3	3	7	44	8	10	4	3	9	3	2018Vergnaud#1313	Brucella spp. (	144	271		BCCN#10-4	Rodent	2010	Australia
1	2	3	12	1	2	3	3	7	44	8	9	4	3	9	3	2018Vergnaud#1315	Brucella spp. (	144	271		BCCN#10-6	Rodent	2010	Australia
1	2	3	12	1	2	5	3	7	44	8	12	4	3	9	3	2018Vergnaud#1311	Brucella spp. (	141	274		BCCN#10-2	Rodent	2010	Australia
1	2	3	12	2	2	3	3	7	44	8	11	4	3	9	3	2018Vergnaud#1314	Brucella spp. (	148	270		BCCN#10-5	Rodent	2010	Australia
1	2	3	12	1	2	5	3	7	44	8	10	6	3	9	3	2018Vergnaud#1316	Brucella spp. (	141	274		BCCN#10-8	Rodent	2010	Australia
1	2	3	12	1	2	5	3	7	44	8	10	4	3	10	5	2018Vergnaud#1308	Brucella spp. (	141	274		BCCN#83-210	Rodent	1983	Australia
1	2	3	12	1	2	5	3	10	44	8	4	6	3	7	3	2018Vergnaud#1309	Brucella spp. (	141	273		BCCN#83-211	Rodent	1983	Australia
1	2	3	12	1	2	5	3	10	44	8	4	6	3	7	3	2018Vergnaud#1318	Brucella spp. (	141	273		BCCN#10-10	Rodent	2010	Australia
1	2	1	12	1	2	5	3	9	44	8	4	6	3	7	3	2018Vergnaud#1319	Brucella spp. (	147	269		BCCN#10-11	Rodent	2010	Australia
1	2	3	12	1	2	5	3	9	44	8	11	6	3	6	3	2018Vergnaud#1317	Brucella spp. (	141	272		BCCN#10-9	Rodent	2010	Australia
1	2	3	11	1	2	5	3	6	44	8	7	4	3	11	3	2018Vergnaud#1310	Brucella spp. (	130	268		BCCN#10-1	Rodent	2010	Australia
1	2	3	11	1	2	3	3	6	44	8	12	4	3	11	3	2018Vergnaud#1312	Brucella spp. (	138	267		BCCN#10-3	Rodent	2010	Australia
1	5	3	13	1	2	5	3	12	37	8	19	5	3	5	10	2018Vergnaud#1320	Brucella sp. BO2	126	266		BCCN#10-12	Human	2010	USA
1	2	9	14	1	2	5	5	7	43	9	9	5	3	9	5	2006LeFleche#016	B. suis bv5	21	2		REF 513	Rodent		Former USSR
1	2	9	14	1	2	5	5	7	43	9	9	5	3	9	5	2017Ferreira#181	B. suis bv5	21	2		REF 513 [biova.	Rodent		Former USSR
1	2	9	14	1	2	5	5	7	43	9	9	5	3	9	5	GCA_000740235	B. suis bv5	21	2	19	REF 513UK	Rodent		Former USSR
1	2	7	14	1	2	5	5	7	43	9	9	5	3	9	5	SRR011163	B. suis bv5	182	349	19	REF 513	Rodent		Former USSR
1	2	9	14	1	2	5	6	8	43	9	7	5	9	5	6	2006LeFleche#188	B. suis bv5	22	1		ELT80	unknown		
3	4	3	13	4	2	3	3	7	36	6	2	4	9	6	5	2018Vergnaud#0060	B. melitensis bv1	55	265		BCCN#74-261	Human	1974	Africa

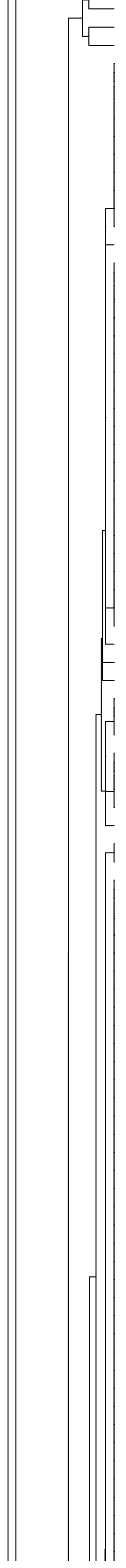

3	4	3	13	4	2	3	3	7	36	6	2	5	3	7	5	2018Vergnaud#0772	B. melitensis bv1	55	265	BCCN#87-40	Human	1987	Paris, France
3	4	3	13	5	2	3	3	7	36	6	2	3	5	4	5	2010Valdezate#097	B. melitensis	48	141	#20050708	Human	2005	Caceres, Spain
3	4	3	13	6	2	3	3	7	36	6	2	3	9	9	5	2018Vergnaud#0209	B. melitensis R.	93	139	BCCN#82-67	Human	1982	Barcelone ,Spain
3	4	2	13	5	2	3	3	7	36	6	2	5	8	4	4	2009Nockler#83	B. melitensis bv1	54	134	S59 1	Human	2006	Callao, Peru
3	4	2	13	5	2	3	3	7	36	6	2	5	8	4	4	2009Nockler#84	B. melitensis bv1	54	134	S59 2	Human	2006	Callao, Peru
3	4	2	13	5	2	3	3	7	36	6	2	5	8	4	4	2009Nockler#85	B. melitensis bv1	54	134	S59 3	Human	2006	Callao, Peru
3	4	2	13	5	2	3	3	7	36	6	2	5	8	4	4	2009Nockler#86	B. melitensis bv1	54	134	S59 4	Human	2006	Callao, Peru
3	4	2	13	5	2	3	3	7	36	6	2	5	8	4	4	2009Nockler#87	B. melitensis bv1	54	134	S59 5	Human	2006	Callao, Peru
3	4	2	13	5	2	3	3	7	36	6	2	5	8	4	4	2009Nockler#88	B. melitensis bv1	54	134	S59 6	Human	2007	Callao, Peru
3	4	2	13	5	2	3	3	7	36	6	2	5	8	4	4	2009Smits#006	B. melitensis bv1	54	134	#6	Human	2000	Peru
3	4	2	13	5	2	3	3	7	36	6	2	5	8	4	4	2009Smits#007	B. melitensis bv1	54	134	#7	Human	2000	Peru
3	4	2	13	5	2	3	3	7	36	6	2	5	8	4	4	2009Smits#008	B. melitensis bv1	54	134	#8	Human	2000	Peru
3	4	2	13	5	2	3	3	7	36	6	2	5	8	4	4	2009Smits#009	B. melitensis bv1	54	134	#9	Human	2000	Peru
3	4	2	13	5	2	3	3	7	36	6	2	5	5	4	4	2009Nockler#90	B. melitensis bv1	54	134	S87 1	Human	2007	Callao, Peru
3	4	2	13	5	2	3	3	7	36	6	2	5	7	4	4	2009Nockler#01	B. melitensis bv1	54	134	S1 1	Human	2003	Callao, Peru
3	4	2	13	5	2	3	3	7	36	6	2	5	7	4	4	2009Nockler#02	B. melitensis bv1	54	134	S1 2	Human	2005	Callao, Peru
3	4	2	13	5	2	3	3	7	36	6	2	5	7	4	4	2009Nockler#03	B. melitensis bv1	54	134	S1 3	Human	2005	Callao, Peru
3	4	2	13	5	2	3	3	7	36	6	2	5	7	4	4	2009Nockler#04	B. melitensis bv1	54	134	S1 4	Human	2005	Callao, Peru
3	4	2	13	5	2	3	3	7	36	6	2	5	7	4	4	2009Nockler#05	B. melitensis bv1	54	134	S1 5	Human	2005	Callao, Peru
3	4	2	13	5	2	3	3	7	36	6	2	5	7	4	4	2009Nockler#06	B. melitensis bv1	54	134	S1 6	Human	2005	Callao, Peru
3	4	2	13	5	2	3	3	7	36	6	2	5	7	4	4	2009Nockler#07	B. melitensis bv1	54	134	S1 7	Human	2005	Callao, Peru
3	4	2	13	5	2	3	3	7	36	6	2	5	7	4	4	2009Nockler#08	B. melitensis bv1	54	134	S1 8	Human	2005	Callao, Peru
3	4	2	13	5	2	3	3	7	36	6	2	5	7	4	4	2009Nockler#09	B. melitensis bv1	54	134	S1 9	Human	2006	Callao, Peru
3	4	2	13	5	2	3	3	7	36	6	2	5	7	4	4	2009Nockler#10	B. melitensis bv1	54	134	S1 10	Human	2006	Callao, Peru
3	4	2	13	5	2	3	3	7	36	6	2	5	7	4	4	2009Nockler#11	B. melitensis bv1	54	134	S1 11	Human	2006	Callao, Peru
3	4	2	13	5	2	3	3	7	36	6	2	5	7	4	4	2009Nockler#12	B. melitensis bv1	54	134	S1 12	Human	2006	Callao, Peru
3	4	2	13	5	2	3	3	7	36	6	2	5	7	4	4	2009Nockler#13	B. melitensis bv1	54	134	S1 13	Human	2006	Callao, Peru
3	4	2	13	5	2	3	3	7	36	6	2	5	7	4	4	2009Nockler#14	B. melitensis bv1	54	134	S1 14	Human	2007	Callao, Peru
3	4	2	13	5	2	3	3	7	36	6	2	5	7	4	4	2009Nockler#15	B. melitensis bv1	54	134	S1 15	Human	2007	Callao, Peru
3	4	2	13	5	2	3	3	7	36	6	2	5	7	4	4	2009Nockler#16	B. melitensis bv1	54	134	S1 16	Human	2007	Callao, Peru
3	4	2	13	5	2	3	3	7	36	6	2	5	7	4	4	2009Nockler#17	B. melitensis bv1	54	134	S1 17	Human	2007	Callao, Peru
3	4	2	13	5	2	3	3	7	36	6	2	5	7	4	4	2009Nockler#18	B. melitensis bv1	54	134	S1 18	Human	2007	Callao, Peru
3	4	2	13	5	2	3	3	7	36	6	2	5	7	4	4	2009Nockler#19	B. melitensis bv1	54	134	S1 19	Human	2007	Callao, Peru
3	4	2	13	5	2	3	3	7	36	6	2	5	7	4	4	2009Smits#001	B. melitensis bv1	54	134	#1	Human	2000	Peru
3	4	2	13	5	2	3	3	7	36	6	2	5	7	4	4	2009Smits#011	B. melitensis bv1	54	134	#11	Human	2000	Peru
3	4	2	13	5	2	3	3	7	36	6	2	5	4	4	4	2009Nockler#89	B. melitensis bv1	54	134	S90 1	Human	2007	Callao, Peru
3	4	2	13	6	2	3	3	7	36	6	2	5	7	4	4	2009Nockler#82	B. melitensis bv1	87	150	S35 1	Human	2005	Callao, Peru
3	4	2	13	5	2	3	3	7	36	6	2	5	7	3	4	2009Smits#015	B. melitensis bv1	54	134	#15	Human	2000	Peru
3	4	2	13	5	2	3	3	7	36	6	2	5	6	3	4	2009Smits#014	B. melitensis bv1	54	134	#14	Human	2001	Peru
3	4	2	13	5	2	3	3	7	36	6	2	5	6	3	4	2009Smits#017	B. melitensis bv1	54	134	#17	Human	2000	Peru
3	4	2	13	5	2	3	3	7	36	6	2	5	6	3	4	2009Smits#022	B. melitensis bv1	54	134	#22	Human	2000	Peru
3	4	2	13	5	2	3	3	7	36	6	2	5	6	4	4	2009Nockler#76	B. melitensis bv1	54	134	S8 1	Human	2004	Callao, Peru
3	4	2	13	5	2	3	3	7	36	6	2	5	6	4	4	2009Nockler#77	B. melitensis bv1	54	134	S8 2	Human	2006	Callao, Peru
3	4	2	13	5	2	3	3	7	36	6	2	5	6	4	4	2009Smits#005	B. melitensis bv1	54	134	#5	Human	2000	Peru
3	4	2	13	5	2	3	3	7	36	6	2	5	6	4	4	2009Smits#024	B. melitensis bv1	54	134	#24	Human	2001	Peru
3	4	2	13	5	2	3	3	7	36	6	2	5	6	2	4	2009Smits#023	B. melitensis bv1	54	134	#23	Human	2000	Peru
3	4	2	13	3	2	3	3	7	36	6	2	6	4	4	4	2009Nockler#80	B. melitensis bv1	80	133	S28 1	Human	2004	Callao, Peru
3	4	2	13	3	2	3	3	7	36	6	2	6	4	4	4	2009Nockler#81	B. melitensis bv1	80	133	S28 2	Human	2005	Callao, Peru
3	4	2	13	3	2	3	3	7	36	6	2	5	4	4	4	2009Nockler#20	B. melitensis bv1	80	133	S2 1	Human	2003	Callao, Peru
3	4	2	13	3	2	3	3	7	36	6	2	5	4	4	4	2009Nockler#21	B. melitensis bv1	80	133	S2 2	Human	2003	Callao, Peru
3	4	2	13	3	2	3	3	7	36	6	2	5	4	4	4	2009Nockler#22	B. melitensis bv1	80	133	S2 3	Human	2003	Callao, Peru
3	4	2	13	3	2	3	3	7	36	6	2	5	4	4	4	2009Nockler#23	B. melitensis bv1	80	133	S2 4	Human	2003	Callao, Peru
3	4	2	13	3	2	3	3	7	36	6	2	5	4	4	4	2009Nockler#24	B. melitensis bv1	80	133	S2 5	Human	2004	Callao, Peru
3	4	2	13	3	2	3	3	7	36	6	2	5	4	4	4	2009Nockler#25	B. melitensis bv1	80	133	S2 6	Human	2004	Callao, Peru
3	4	2	13	3	2	3	3	7	36	6	2	5	4	4	4	2009Nockler#26	B. melitensis bv1	80	133	S2 7	Human	2004	Callao, Peru
3	4	2	13	3	2	3	3	7	36	6	2	5	4	4	4	2009Nockler#27	B. melitensis bv1	80	133	S2 8	Human	2004	Callao, Peru
3	4	2	13	3	2	3	3	7	36	6	2	5	4	4	4	2009Nockler#28	B. melitensis bv1	80	133	S2 9	Human	2004	Callao, Peru
3	4	2	13	3	2	3	3	7	36	6	2	5	4	4	4	2009Nockler#29	B. melitensis bv1	80	133	S2 10	Human	2004	Callao, Peru
3	4	2	13	3	2	3	3	7	36	6	2	5	4	4	4	2009Nockler#30	B. melitensis bv1	80	133	S2 11	Human	2004	Callao, Peru
3	4	2	13	3	2	3	3	7	36	6	2	5	4	4	4	2009Nockler#31	B. melitensis bv1	80	133	S2 12	Human	2004	Callao, Peru
3	4	2	13	3	2	3	3	7	36	6	2	5	4	4	4	2009Nockler#32	B. melitensis bv1	80	133	S2 13	Human	2004	Callao, Peru
3	4	2	13	3	2	3	3	7	36	6	2	5	4	4	4	2009Nockler#33	B. melitensis bv1	80	133	S2 14	Human	2004	Callao, Peru
3	4	2	13	3	2	3	3	7	36	6	2	5	4	4	4	2009Nockler#34	B. melitensis bv1	80	133	S2 15	Human	2004	Callao, Peru
3	4	2	13	3	2	3	3	7	36	6	2	5	4	4	4	2009Nockler#35	B. melitensis bv1	80	133	S2 16	Human	2004	Callao, Peru
3	4	2	13	3	2	3	3	7	36	6	2	5	4	4	4	2009Nockler#36	B. melitensis bv1	80	133	S2 17	Human	2004	Callao, Peru
3	4	2	13	3	2	3	3	7	36	6	2	5	4	4	4	2009Nockler#37	B. melitensis bv1	80	133	S2 18	Human	2004	Callao, Peru
3	4	2	13	3	2	3	3	7	36	6	2	5	4	4	4	2009Nockler#38	B. melitensis bv1	80	133	S2 19	Human	2004	Callao, Peru
3	4	2	13	3	2	3	3	7	36	6	2	5	4	4	4	2009Nockler#39	B. melitensis bv1	80	133	S2 20	Human	2004	Callao, Peru
3	4	2	13	3	2	3	3	7	36	6	2	5	4	4	4	2009Nockler#40	B. melitensis bv1	80	133	S2 21	Human	2004	Callao, Peru
3	4	2	13	3	2	3	3	7	36	6	2	5	4	4	4	2009Nockler#41	B. melitensis bv1	80	133	S2 22	Human	2004	Callao, Peru
3	4	2	13	3	2	3	3	7	36	6	2	5	4	4	4	2009Nockler#42	B. melitensis bv1	80	133	S2 23	Human	2005	Callao, Peru
3	4	2	13	3	2	3	3	7	36	6	2	5	4	4	4	2009Nockler#43	B. melitensis bv1	80	133	S2 24	Human	2005	Callao, Peru
3	4	2	13	3	2	3	3	7	36	6	2	5	4	4	4	2009Nockler#44	B. melitensis bv1	80	133	S2 25	Human	2005	Callao, Peru
3	4	2	13	3	2	3	3	7	36	6	2	5	4	4	4	2009Nockler#45	B. melitensis bv1	80	133	S2 26	Human	2005	Callao, Peru
3	4	2	13	3	2	3	3	7	36	6	2	5	4	4	4	2009Nockler#46	B. melitensis bv1	80	133	S2 27	Human	2005	Callao, Peru
3	4	2	13	3	2	3	3	7	36	6	2	5	4	4	4	2009Nockler#47	B. melitensis bv1	80	133	S2 28	Human	2005	Callao, Peru
3	4	2	13	3	2	3	3	7	36	6	2	5	4	4	4	2009Nockler#48	B. melitensis bv1	80	133	S2 29	Human	2005	Callao, Peru
3	4	2	13	3	2	3	3	7	36	6	2	5	4	4	4	2009Nockler#49	B. melitensis bv1	80	133	S2 30	Human	2005	Callao, Peru
3	4	2	13	3	2	3	3	7	36	6	2	5	4	4	4	2009Nockler#50	B. melitensis bv1	80	133	S2 31	Human	2005	Callao, Peru
3	4	2	13	3	2	3	3	7	36	6	2	5	4	4	4	2009Nockler#51	B. melitensis bv1	80	133	S2 32	Human	2005	Callao, Peru
3	4	2	13	3	2	3	3	7	36	6	2	5	4	4	4	2009Nockler#52	B. melitensis bv1	80	133	S2 33	Human	2005	Callao, Peru
3	4	2	13	3	2	3	3	7	36	6	2	5	4	4	4	2009Nockler#53	B. melitensis bv1	80	133	S2 34	Human	2005	Callao, Peru
3	4	2	13	3	2	3	3	7	36	6	2	5	4	4	4	2009Nockler#54	B. melitensis bv1	80	133	S2 35	Human	2005	Callao, Peru
3	4	2	13	3	2	3	3	7	36	6	2	5	4	4	4	2009Nockler#55	B. melitensis bv1	80	133	S2 36	Human	2005	Callao, Peru
3	4	2	13	3	2	3	3	7	36	6	2	5	4	4	4	2009Nockler#56	B. melitensis bv1	80	133	S2 37	Human	2005	Callao, Peru
3	4	2	13	3	2	3	3	7	36	6	2	5	4	4	4	2009Nockler#57	B. melitensis bv1	80	133	S2 38	Human	2005	Callao, Peru

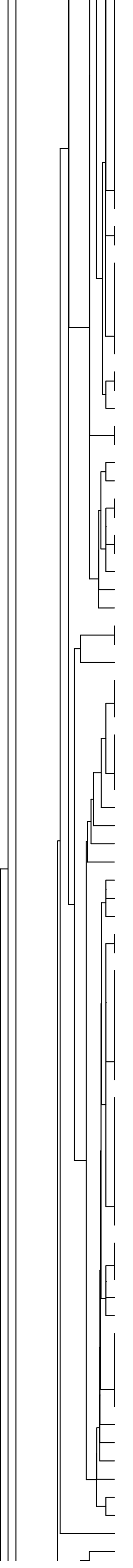

3	4	2	13	3	2	3	3	7	36	6	2	5	4	4	4	2009Nockler#58	B. melitensis bv1	80	133		S2 39	Human	2006	Callao, Peru
3	4	2	13	3	2	3	3	7	36	6	2	5	4	4	4	2009Nockler#59	B. melitensis bv1	80	133		S2 40	Human	2006	Callao, Peru
3	4	2	13	3	2	3	3	7	36	6	2	5	4	4	4	2009Nockler#60	B. melitensis bv1	80	133		S2 41	Human	2006	Callao, Peru
3	4	2	13	3	2	3	3	7	36	6	2	5	4	4	4	2009Nockler#61	B. melitensis bv1	80	133		S2 42	Human	2006	Callao, Peru
3	4	2	13	3	2	3	3	7	36	6	2	5	4	4	4	2009Nockler#62	B. melitensis bv1	80	133		S2 43	Human	2006	Callao, Peru
3	4	2	13	3	2	3	3	7	36	6	2	5	4	4	4	2009Nockler#63	B. melitensis bv1	80	133		S2 44	Human	2006	Callao, Peru
3	4	2	13	3	2	3	3	7	36	6	2	5	4	4	4	2009Nockler#64	B. melitensis bv1	80	133		S2 45	Human	2006	Callao, Peru
3	4	2	13	3	2	3	3	7	36	6	2	5	4	4	4	2009Nockler#65	B. melitensis bv1	80	133		S2 46	Human	2006	Callao, Peru
3	4	2	13	3	2	3	3	7	36	6	2	5	4	4	4	2009Nockler#66	B. melitensis bv1	80	133		S2 47	Human	2006	Callao, Peru
3	4	2	13	3	2	3	3	7	36	6	2	5	4	4	4	2009Nockler#67	B. melitensis bv1	80	133		S2 48	Human	2007	Callao, Peru
3	4	2	13	3	2	3	3	7	36	6	2	5	4	4	4	2009Nockler#68	B. melitensis bv1	80	133		S2 49	Human	2007	Callao, Peru
3	4	2	13	3	2	3	3	7	36	6	2	5	4	4	4	2009Nockler#69	B. melitensis bv1	80	133		S2 50	Human	2007	Callao, Peru
3	4	2	13	3	2	3	3	8	36	6	2	5	4	4	4	2009Nockler#78	B. melitensis bv1	80	151		S20 1	Human	2004	Callao, Peru
3	4	2	13	3	2	3	3	8	36	6	2	5	4	4	4	2009Nockler#79	B. melitensis bv1	80	151		S20 2	Human	2004	Callao, Peru
3	4	2	13	3	2	3	3	7	36	6	2	5	5	4	4	2009Nockler#70	B. melitensis bv1	80	133		S7 1	Human	2004	Callao, Peru
3	4	2	13	3	2	3	3	7	36	6	2	5	5	4	4	2009Nockler#71	B. melitensis bv1	80	133		S7 2	Human	2006	Callao, Peru
3	4	2	13	3	2	3	3	7	36	6	2	5	5	4	4	2009Nockler#72	B. melitensis bv1	80	133		S7 3	Human	2006	Callao, Peru
3	4	2	13	3	2	3	3	7	36	6	2	5	5	4	4	2009Nockler#73	B. melitensis bv1	80	133		S7 4	Human	2006	Callao, Peru
3	4	2	13	3	2	3	3	7	36	6	2	5	5	4	4	2009Nockler#74	B. melitensis bv1	80	133		S7 5	Human	2006	Callao, Peru
3	4	2	13	3	2	3	3	7	36	6	2	5	5	4	4	2009Nockler#75	B. melitensis bv1	80	133		S7 6	Human	2006	Callao, Peru
3	4	2	13	3	2	3	3	7	36	6	2	5	6	4	4	2009Smits#003	B. melitensis bv1	80	133		#3	Human	2000	Peru
3	4	2	13	3	2	3	3	7	36	6	2	5	6	4	4	2009Smits#010	B. melitensis bv1	80	133		#10	Human	2000	Peru
3	4	2	13	3	2	3	3	7	36	6	2	5	6	2	4	2009Smits#019	B. melitensis bv1	80	133		#19	Human	2000	Peru
3	4	2	13	4	2	3	3	7	36	6	2	5	6	8	4	2007AlDahouk#001	B. melitensis bv1	47	135		BCCN#V3	Human		Mexico
3	4	2	13	4	2	3	3	7	36	6	2	5	6	8	4	2018Vergnaud#0206	B. melitensis R.	47	135		BCCN#74-242	Human	1974	Paris, France
3	4	2	13	3	2	3	3	7	36	6	2	4	8	3	4	2009Smits#020	B. melitensis bv1	80	133		#20	Human	2000	Peru
3	4	2	13	3	2	3	3	7	36	6	2	4	8	4	4	2017Hanot-Mamb.	B. melitensis bv1	80	133		L3/107	Human	1999	Belgium
3	4	2	13	5	2	3	3	7	36	6	2	4	8	3	4	2009Smits#013	B. melitensis bv1	54	134		#13	Human	2000	Peru
3	4	2	13	5	2	3	3	7	36	6	2	4	8	3	4	2009Smits#018	B. melitensis bv1	54	134		#18	Human	2000	Peru
3	4	2	13	5	2	3	3	7	36	6	2	4	8	4	4	2009Smits#002	B. melitensis bv1	54	134		#2	Human	2000	Peru
3	4	2	13	5	2	3	3	7	36	6	2	4	8	4	4	2009Smits#004	B. melitensis bv1	54	134		#4	Human	2000	Peru
3	4	2	13	5	2	3	3	7	36	6	2	4	8	2	4	2009Smits#016	B. melitensis bv1	54	134		#16	Human	2000	Peru
3	4	2	13	5	2	3	3	7	36	6	2	4	4	4	4	2009Smits#012	B. melitensis bv1	54	134		#12	Human	2000	Peru
3	4	2	13	5	2	3	3	7	36	6	2	4	6	3	4	2009Smits#021	B. melitensis bv1	54	134		#21	Human	2000	Peru
3	4	2	13	5	2	3	3	9	36	6	2	3	8	9	6	2007AlDahouk#007	B. melitensis bv1	54	132		BCCN#96-137b	Human	1996	Argentina
3	4	2	13	5	2	3	3	9	36	6	2	3	8	9	6	2018Vergnaud#0113	B. melitensis aty.	54	132		BCCN#96-137b	Human	1996	Argentina
3	4	2	13	4	2	3	3	7	36	6	2	7	8	5	6	2018Vergnaud#0286	B. melitensis bv1	47	135		BCCN#98-51	Human	1998	Buenos Aires ,Argentina
3	4	2	13	4	2	3	3	5	36	6	2	5	7	3	6	2007Marianelli#001	B. melitensis bv1	47	136		REF 16M	unknown		
3	4	2	13	4	2	3	3	5	36	6	2	5	7	3	6	2012Ferreira#001	B. melitensis bv1	47	136		REF 16M	unknown		
3	4	2	13	4	2	3	3	5	36	6	2	5	7	3	6	GCA_000007125	B. melitensis bv1	47	136	73	REF 16M	Caprine	2001	USA
3	4	2	13	4	2	3	3	5	36	6	2	5	8	3	6	2006LeFleche#001	B. melitensis bv1	47	136		REF 16M	Caprine		USA
3	4	2	13	4	2	3	3	5	36	6	2	5	8	3	6	2018Vergnaud#0001	B. melitensis bv1	47	136		BCCN#R1	Caprine		USA
3	4	2	13	4	2	3	3	5	36	6	2	5	8	3	6	2018Vergnaud#0094	B. melitensis bv1	47	136		BCCN#79-86	Human	1979	Nouzilly ,Indre-et-Loire, .
3	4	2	13	4	2	3	3	5	36	6	2	5	8	3	6	2018Vergnaud#0228	B. melitensis bv1	47	136		BCCN#00-17	Deer/El.	2000	Essonne, France
3	4	2	13	4	2	3	3	5	36	6	2	6	7	3	6	2007Garcia_Yoldi.	B. melitensis bv1	47	136		R1Sp	unknown		Spain
3	4	2	13	4	2	3	3	5	36	6	2	4	7	2	6	2017Liu#175	B. melitensis bv1	47	136		M1	Ovine	2016	
3	4	2	13	3	2	3	3	5	36	6	2	5	7	3	7	GCA_000740415	B. melitensis bv1	80	350	73	REF 16M	unknown		
3	4	2	13	4	2	3	3	5	36	6	2	3	5	3	5	2010Valdezate#087	B. melitensis	47	136		#20030213	Human	2003	Teruel, Spain
3	4	2	13	4	2	3	3	8	36	6	2	4	6	3	7	2018Vergnaud#0440	B. melitensis bv1	47	138		BCCN#81-136	Ovine	1981	Domaine du Merle ,Bo.
3	4	2	13	4	2	3	3	8	36	6	2	4	6	3	8	2018Vergnaud#0442	B. melitensis bv1	47	138		BCCN#82-92	Ovine	1982	France
3	4	2	13	4	2	3	3	8	36	6	2	4	6	3	5	2018Vergnaud#0441	B. melitensis bv1	47	138		BCCN#81-137	Ovine	1981	Domaine du Merle ,Bo.
3	4	2	13	4	2	3	3	8	36	6	2	8	6	3	6	2018Vergnaud#0215	B. melitensis R.	47	138		BCCN#V4B	Caprine		Mexico
3	4	2	13	4	2	3	3	8	36	6	2	8	6	3	6	2018Vergnaud#0478	B. melitensis bv1	47	138		BCCN#95-6	Human	1995	Die ,Drome, France
3	4	2	13	4	2	3	3	8	36	6	2	5	6	3	6	2007Garcia_Yoldi.	B. melitensis bv1	47	138		R1	Ovine	1986	Spain
3	4	2	13	4	2	3	3	8	36	6	2	5	6	3	6	2018Vergnaud#0048	B. melitensis bv1	47	138		BCCN#87-23	Ovine	1987	Saragosse, Spain
3	4	2	13	4	2	3	3	8	36	6	2	5	6	3	6	2018Vergnaud#0282	B. melitensis bv1	47	138		BCCN#91-78	Cattle	1991	Salonique ,Greece
3	4	2	13	4	2	3	3	8	36	6	2	5	6	3	6	2018Vergnaud#1145	B. melitensis bv1	47	138		BCCN#91-55	Ovine	1991	Saragosse, Spain
3	4	2	13	4	2	3	3	8	36	6	2	5	6	3	6	2018Vergnaud#1146	B. melitensis bv1	47	138		BCCN#91-56	Ovine	1991	Saragosse, Spain
3	4	2	13	4	2	3	3	8	36	6	2	5	6	3	6	2018Vergnaud#1292	B. melitensis bv1	47	138		BCCN#86-25	Ovine	1986	Spain
3	4	2	13	4	2	3	3	8	36	6	2	5	6	3	6	2018Vergnaud#1293	B. melitensis bv1	47	138		BCCN#86-54	Ovine	1986	Saragosse,Spain
3	4	2	13	4	2	3	3	8	36	6	2	4	6	3	6	2006LeFleche#079	B. melitensis bv1	47	138		BCCN#96-22	Ovine	1996	Israel
3	4	2	13	4	2	3	3	8	36	6	2	4	6	3	6	2012Ferreira#058	B. melitensis bv1	47	138		LNIV-523REV1.	unknown	2006	Tras-os-Montes, Portugal
3	4	2	13	4	2	3	3	8	36	6	2	4	6	3	6	2012Ferreira#059	B. melitensis bv1	47	138		LNIV-527REV1.	unknown	2006	Tras-os-Montes, Portugal
3	4	2	13	4	2	3	3	8	36	6	2	4	6	3	6	2013Garofolo_13.	B. melitensis bv1	47	138		13550	Caprine	2011	Centuripe,Italy
3	4	2	13	4	2	3	3	8	36	6	2	4	6	3	6	2018Vergnaud#0283	B. melitensis bv1	47	138		BCCN#96-19	Human	1996	Israel
3	4	2	13	4	2	3	3	8	36	6	2	4	6	3	6	2018Vergnaud#0364	B. melitensis R.	47	138		BCCN#96-26	Ovine	1996	Israel
3	4	2	13	4	2	3	3	8	36	6	2	4	6	3	6	2018Vergnaud#0889	B. melitensis bv1	47	138		BCCN#93-43	Human	1993	Haute-Garonne, France
3	4	2	13	4	2	3	3	8	36	6	2	4	6	3	6	2018Vergnaud#1033	B. melitensis bv1	47	138		BCCN#89-88	Ovine	1989	Savoie, France
3	4	2	13	4	2	3	3	8	36	6	2	4	7	3	6	2007Garcia_Yoldi.	B. melitensis bv1	47	138		R16	Ovine	2000	Spain
3	4	2	13	4	2	3	3	8	36	6	2	4	7	3	6	2013Garofolo_21.	B. melitensis bv1	47	138		21031	Caprine	2011	,Italy
3	4	2	13	4	2	3	3	8	36	6	2	4	7	3	6	2018Vergnaud#0767	B. melitensis bv1	47	138		BCCN#82-16	Ovine	1982	Myans ,Savoie, France
3	4	2	13	4	2	3	3	8	36	6	2	4	3	3	6	2012Ferreira#116	B. melitensis bv1	47	138		LNIV-764REV1.	unknown	2005	Entre Douro e Minho, P.
3	4	2	13	4	2	3	3	8	36	6	2	4	5	3	6	2007Garcia_Yoldi.	B. melitensis bv1	47	138		R15	Ovine	1995	Spain
3	4	3	13	4	2	3	3	8	36	6	2	4	6	3	6	2011Muendo#01	B. melitensis bv1	55	195		1-KEBm1	Cattle	2009	Central, Kenya
3	4	3	13	4	2	3	3	8	36	6	2	4	6	3	6	2011Muendo#02	B. melitensis bv1	55	195		2-KEBm1	Cattle	2009	Central, Kenya
3	4	3	13	4	2	3	3	8	36	6	2	4	6	3	6	2011Muendo#03	B. melitensis bv1	55	195		3-KEBm1	Cattle	2009	Central, Kenya
3	4	3	13	4	2	3	3	8	36	6	2	4	6	3	6	2011Muendo#04	B. melitensis bv1	55	195		4-KEBm1	Cattle	2009	Central, Kenya
3	4	3	13	4	2	3	3	8	36	6	2	4	6	3	6	2011Muendo#05	B. melitensis bv1	55	195		5-KEBm1	Cattle	2009	Central, Kenya
3	4	2	13	4	2	3	3	9	36	6	2	4	6	3	6	2007Garcia_Yoldi.	B. melitensis bv1	47	137		R32	unknown	1997	Spain
3	4	2	13	4	2	3	3	8	36	6	4	4	6	3	6	2018Vergnaud#0284	B. melitensis bv1	47	138		BCCN#96-20	Ovine	1996	Israel
3	4	2	13	4	2	3	3	8	36	6	2	4	6	3	6	SRR032601	B. melitensis bv1	80	151	73	REV1	unknown		
3	4	2	13	4	2	3	3	8	36	6	2	5	5	3	6	2007Garcia_Yoldi.	B. melitensis bv1	47	138		R26	unknown	1996	Spain
3	4	2	13	4	2	3	3	8	36	6	2	4	7	4	6	2006LeFleche#081	B. melitensis bv1	47	138		BCCN#90-61	unknown	1990	South Africa
3	4	2	13	4	2	3	3	8	36	6	2	4	6	4	6	2007Garcia_Yoldi.	B. melitensis bv1	47	138		R5	Ovine	1990	South Africa
3	4	3	13	5	3	3	3	7	40	6	2	5	5	7	4	2010Valdezate#091	B. melitensis	53	149		#20090777	Human	2009	Madrid, Spain
3	4	3	13	6	2	3	3	6	36	6	2	3	3	5	3	2010Valdezate#085	B. melitensis	93	140		#20071186	Human	2007	Alicante, Spain

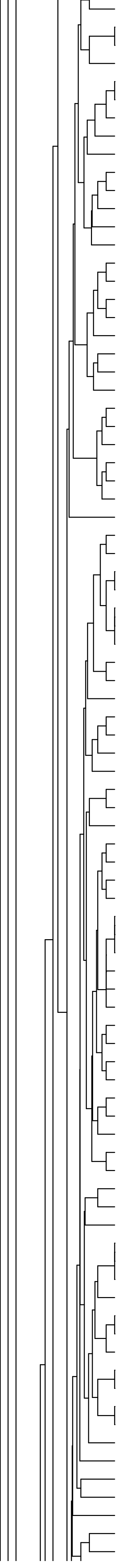

3	4	3	13	5	2	3	3	6	36	6	2	3	8	5	7	2010Valdezate#096	B. melitensis	48	146	#20080634	Human	2008	Caceres, Spain
3	4	3	13	5	3	3	3	6	36	6	2	4	6	5	3	2010Valdezate#102	B. melitensis	53	145	#20040143	Human	2004	Valencia, Spain
3	4	3	13	5	3	3	3	6	36	6	2	4	6	5	3	2010Valdezate#104	B. melitensis	53	145	#20040170	Human	2004	Valencia, Spain
3	4	3	13	4	2	3	3	6	36	6	2	4	6	5	4	2012Ferreira#040	B. melitensis bv1	55	147	AFSSA-02/6501	unknown	2002	Portugal
3	4	3	13	6	2	3	3	6	36	6	2	3	6	7	4	2018Vergnaud#0028	B. melitensis bv1	93	140	BCCN#83-199	Human	1983	Spain
3	4	3	13	6	2	3	3	6	36	6	2	3	6	7	4	2018Vergnaud#1261	B. melitensis bv1	93	140	BCCN#83-200	Human	1983	Spain
3	4	3	13	6	2	3	3	6	36	6	2	3	6	6	4	2018Vergnaud#0770	B. melitensis bv1	93	140	BCCN#82-64	Human	1982	San Lazaro ,Spain
3	4	3	13	6	2	3	3	7	36	6	2	2	6	7	4	2010Valdezate#082	B. melitensis	93	139	#20030176	Human	2003	Valencia, Spain
3	4	3	13	4	2	3	3	9	36	6	2	3	6	3	4	2010Valdezate#080	B. melitensis	55	148	#20081168	Human	2008	Guadalajara, Spain
3	4	3	13	5	2	3	3	6	36	6	2	3	3	3	4	2010Valdezate#072	B. melitensis	48	146	#9800059	Human	1998	Madrid, Spain
3	4	3	13	5	2	3	3	6	36	6	2	3	6	3	4	2010Valdezate#078	B. melitensis	48	146	#20070523	Human	2007	Ciudad Real, Spain
3	4	3	13	5	2	3	3	6	36	6	2	2	4	3	4	2010Valdezate#074	B. melitensis	48	146	#20081715	Human	2008	Sevilla, Spain
3	4	3	13	5	2	3	3	6	36	5	2	5	6	3	4	2018Vergnaud#0082	B. melitensis bv1	48	130	BCCN#75-76	Human	1975	Navarre, Spain
3	4	3	13	5	2	3	3	6	36	6	2	4	6	3	6	2010Valdezate#092	B. melitensis	48	146	#9800053	Human	1998	Almeria, Spain
3	4	3	13	5	3	3	3	5	36	6	2	5	3	3	4	2010Valdezate#070	B. melitensis	53	143	#20040440	Human	2004	Malaga, Spain
3	4	3	13	5	3	3	3	5	36	6	2	4	3	3	4	2010Valdezate#071	B. melitensis	53	143	#20010190	Human	2010	Malaga, Spain
3	4	3	13	5	2	3	3	5	36	6	2	4	3	3	4	2010Valdezate#067	B. melitensis	48	144	#20000167	Human	2000	Cordoba, Spain
3	4	3	13	5	2	3	3	5	36	6	2	4	3	4	4	2010Valdezate#069	B. melitensis	48	144	#20030179	Human	2003	Madrid, Spain
3	4	3	13	5	3	3	3	5	36	6	2	4	5	4	4	2010Valdezate#088	B. melitensis	53	143	#20040233	Human	2004	Caceres, Spain
3	4	3	13	5	2	3	3	7	36	6	2	6	3	4	4	2010Valdezate#076	B. melitensis	48	141	#20060684	Human	2006	Ciudad Real, Spain
3	4	3	13	5	2	3	3	6	36	6	2	8	3	4	4	2010Valdezate#079	B. melitensis	48	146	#20071290	Human	2007	Ciudad Real, Spain
3	4	3	13	5	3	3	3	6	36	6	2	5	3	4	4	2010Valdezate#089	B. melitensis	53	145	#20040163	Human	2004	Badajoz, Spain
3	4	3	13	5	3	3	3	6	36	6	2	4	4	6	4	2010Valdezate#094	B. melitensis	53	145	#20000193	Human	2000	Huelva, Spain
3	4	3	13	5	3	3	3	6	36	6	2	3	4	6	4	2010Valdezate#098	B. melitensis	53	145	#20000232	Human	2000	Huelva, Spain
3	4	3	13	5	3	3	3	6	36	6	2	3	4	6	3	2010Valdezate#105	B. melitensis	53	145	#20000233	Human	2000	Huelva, Spain
3	4	3	13	5	3	3	3	6	36	6	2	6	4	6	5	2010Valdezate#101	B. melitensis	53	145	#9900109	Human	1999	Cordoba, Spain
3	4	3	13	5	3	3	3	6	36	6	2	4	4	6	5	2010Valdezate#103	B. melitensis	53	145	#9900108	Human	1999	Cordoba, Spain
3	4	3	13	5	3	3	3	6	36	5	2	4	4	6	5	2018Vergnaud#0004	B. melitensis R.	53	263	BCCN#95-15	Human	1995	Spain
3	4	3	13	4	2	3	3	6	36	6	9	5	3	5	4	2010Valdezate#081	B. melitensis	55	147	#20020475	Human	2002	Valencia, Spain
3	4	3	13	4	2	3	3	6	36	5	2	5	8	3	9	2018Vergnaud#1110	B. melitensis bv1	55	128	BCCN#97-120	Human	1997	Isère, France
3	4	3	13	4	2	3	3	6	36	5	2	7	8	3	9	2018Vergnaud#1111	B. melitensis bv1	55	128	BCCN#97-122	Human	1997	Paris, France
3	4	3	13	4	2	3	3	6	36	5	2	4	8	3	5	2018Vergnaud#0832	B. melitensis bv1	55	128	BCCN#76-422	Human	1976	Navarre, Spain
3	4	3	13	4	2	3	3	6	36	5	2	4	8	3	5	2018Vergnaud#0887	B. melitensis bv1	55	128	BCCN#76-421	Human	1976	Navarre, Spain
3	4	3	13	4	2	3	3	6	36	5	2	5	8	3	5	2012Ferreira#088	B. melitensis bv1	55	128	LNIV-669Bm1-07	unknown	2007	Ribatejo e Oeste, Portu.
3	4	3	13	4	2	3	3	6	36	5	2	5	8	3	5	2012Ferreira#089	B. melitensis bv1	55	128	LNIV-670Bm1-07	unknown	2007	Ribatejo e Oeste, Portu.
3	4	3	13	4	2	3	3	6	36	5	2	5	8	3	5	2012Ferreira#090	B. melitensis bv1	55	128	LNIV-677Bm1-07	unknown	2007	Ribatejo e Oeste, Portu.
3	4	3	13	4	2	3	3	6	36	5	2	4	8	5	5	2012Ferreira#109	B. melitensis bv3	55	128	LNIV-284Bm3-04	unknown	2004	Algarve, Portugal
3	4	3	14	4	2	3	3	6	36	5	2	4	8	5	5	2012Ferreira#122	B. melitensis bv1	96	186	LNIV-770Bm1-07	unknown	2007	Entre Douro e Minho, P.
3	4	3	13	4	2	3	3	6	36	5	2	7	10	3	7	2018Vergnaud#0808	B. melitensis bv1	55	128	BCCN#96-166	Human	1996	Portugal
3	4	3	11	4	2	3	3	6	36	5	2	5	5	3	5	2012Ferreira#117	B. melitensis bv1	97	187	LNIV-765Bm1-06	unknown	2006	Entre Douro e Minho, P.
3	4	3	11	4	2	3	3	6	36	5	2	5	7	3	5	2018Vergnaud#0032	B. melitensis bv1	97	187	BCCN#84-9	Human	1984	France
3	4	3	11	4	2	3	3	6	36	5	2	6	9	3	5	2018Vergnaud#0085	B. melitensis bv1	97	187	BCCN#76-409	Human	1976	France
3	4	3	11	4	2	3	3	6	36	5	2	7	7	7	5	2018Vergnaud#0476	B. melitensis bv1	97	187	BCCN#94-20	Human	1994	Portugal
3	4	3	13	5	2	3	3	7	36	5	2	5	8	3	5	2012Ferreira#065	B. melitensis bv3	48	142	LNIV-312Bm3-04	unknown	2004	Beira Litoral, Portugal
3	4	3	13	5	2	3	3	5	36	5	2	5	8	3	5	2012Ferreira#084	B. melitensis bv3	48	188	LNIV-158Bm3-03	unknown	2003	Beira Litoral, Portugal
3	4	3	13	5	2	3	3	8	36	5	2	5	5	4	5	2007Garcia_Yoldi.	B. melitensis bv1	48	131	R22	Human	2002	Spain
3	4	3	13	5	2	3	3	6	36	5	2	7	12	3	5	2018Vergnaud#1037	B. melitensis bv1	48	130	BCCN#98-11	Human	1998	Paris, France
3	4	3	13	5	2	3	3	6	36	5	2	7	11	3	5	2018Vergnaud#1377	B. melitensis bv1	48	130	BCCN#98-5	Human	1998	Paris, France
3	4	3	13	5	2	3	3	6	36	5	2	9	8	3	5	2012Ferreira#041	B. melitensis bv1	48	130	AFSSA-05/0716	unknown	2005	Portugal
3	4	3	13	5	2	3	3	6	36	5	2	7	8	3	5	2018Vergnaud#1109	B. melitensis bv1	48	130	BCCN#94-60	Human	1994	Val-de-Marne, France
3	4	3	13	5	2	3	3	6	36	5	2	6	9	3	5	2018Vergnaud#1263	B. melitensis bv1	48	130	BCCN#83-214	Human	1983	France
3	4	3	13	5	2	3	3	6	36	5	2	6	9	3	5	2018Vergnaud#1264	B. melitensis bv1	48	130	BCCN#83-216	Human	1983	France
3	4	3	13	5	2	3	3	6	36	5	2	6	9	3	5	2018Vergnaud#1369	B. melitensis bv1	48	130	BCCN#93-62	Human	1993	Rhone, France
3	4	3	13	5	2	3	3	6	36	5	2	6	6	3	5	2012Ferreira#113	B. melitensis bv1	48	130	LNIV-760Bm1-04	unknown	2004	Entre Douro e Minho, P.
3	4	3	13	5	2	3	3	6	36	5	2	6	7	3	5	2018Vergnaud#0486	B. melitensis bv1	48	130	BCCN#98-96	Human	1998	Correze, France
3	4	3	13	5	2	3	3	6	36	5	2	6	3	3	5	2018Vergnaud#0852	B. melitensis bv1	48	130	BCCN#95-53	Human	1995	Portugal
3	4	3	13	5	2	3	3	6	36	5	2	3	7	3	5	2018Vergnaud#0080	B. melitensis bv1	48	130	BCCN#74-270	Human	1974	Navarre, Spain
3	4	3	13	5	2	3	3	6	36	5	2	8	7	3	5	2018Vergnaud#0095	B. melitensis bv1	48	130	BCCN#79-129	Human	1979	Loiret, France
3	4	3	13	5	2	3	3	6	36	5	2	5	7	8	5	2018Vergnaud#0072	B. melitensis bv1	48	130	BCCN#74-238	Human	1974	Paris, France
3	4	3	13	5	2	3	3	6	36	5	2	5	7	3	5	2018Vergnaud#0092	B. melitensis bv1	48	130	BCCN#78-8	Human	1978	France
3	4	3	13	5	2	3	3	6	36	5	2	6	5	3	6	2018Vergnaud#0074	B. melitensis bv1	48	130	BCCN#74-248	Human	1974	Paris, France
3	4	3	13	5	2	3	3	6	36	5	2	6	8	3	6	2018Vergnaud#0748	B. melitensis bv1	48	130	BCCN#85-6	Human	1985	Portugal
3	4	3	13	5	2	3	3	6	36	5	2	4	7	3	6	2018Vergnaud#1036	B. melitensis bv1	48	130	BCCN#93-57	Human	1993	Haute-Garonne, France
3	4	3	13	3	2	3	3	6	36	5	2	6	5	3	5	2012Ferreira#078	B. melitensis bv3	95	190	LNIV-509Bm3-05	unknown	2005	Beira Interior, Portugal
3	4	3	13	4	2	3	3	6	36	5	2	6	5	3	5	2012Ferreira#114	B. melitensis bv1	55	128	LNIV-761Bm1-04	unknown	2004	Entre Douro e Minho, P.
3	4	3	13	5	2	3	3	6	36	6	2	5	3	6	6	2010Valdezate#086	B. melitensis	48	146	#20081102	Human	2008	Caceres, Spain
3	4	3	13	5	2	3	3	6	36	6	2	5	9	6	7	2010Valdezate#095	B. melitensis	48	146	#20080232	Human	2008	Madrid, Spain
3	4	3	13	5	2	3	3	6	36	5	2	5	7	8	7	2018Vergnaud#1029	B. melitensis bv1	48	130	BCCN#80-87	Human	1980	Loiret, France
3	4	3	13	5	2	3	3	6	36	5	2	5	8	6	8	2012Ferreira#042	B. melitensis bv1	48	130	LNIV-860Bm3-09	unknown	2009	Ribatejo e Oeste, Portu.
3	4	3	13	5	2	3	3	6	36	5	2	5	8	6	8	2012Ferreira#043	B. melitensis bv1	48	130	LNIV-861Bm3-09	unknown	2009	Ribatejo e Oeste, Portu.
3	4	3	13	5	2	3	3	6	36	5	2	5	8	6	8	2012Ferreira#044	B. melitensis bv1	48	130	LNIV-862Bm3-09	unknown	2009	Ribatejo e Oeste, Portu.
3	4	3	13	5	2	3	3	6	36	5	2	5	10	6	5	2018Vergnaud#0107	B. melitensis bv1	48	130	BCCN#93-89	Ovine	1993	Pays Basque Espagnol.
3	4	3	13	5	2	3	3	6	36	5	2	4	8	6	5	2006LeFleche#098	B. melitensis	48	130	BCCN#R19	Caprine		Malta
3	4	3	13	5	2	3	3	6	36	5	2	4	8	6	5	2018Vergnaud#0109	B. melitensis bv1	48	130	BCCN#97-87	Human	1997	Sicile, Italy
3	4	3	14	5	2	3	3	6	36	5	2	4	8	6	5	2012Ferreira#091	B. melitensis bv3	52	129	LNIV-79Bm3-01	unknown	2001	Alentejo, Portugal
3	4	3	13	5	2	3	3	6	36	5	2	3	3	6	5	2018Vergnaud#0054	B. melitensis bv1	48	130	BCCN#94-15	Human	1994	Paris, France
3	4	3	13	5	2	3	3	6	36	5	2	3	3	6	5	2018Vergnaud#1372	B. melitensis bv1	48	130	BCCN#95-4	Human	1995	Paris, France
3	4	3	13	5	2	3	3	6	36	5	2	4	3	6	4	2018Vergnaud#0033	B. melitensis bv1	48	130	BCCN#84-10	Human	1984	Belgium
3	4	3	13	5	2	3	3	6	36	5	2	4	3	6	4	2018Vergnaud#0729	B. melitensis bv1	48	130	BCCN#84-1	Human	1984	Lommel ,Belgium
3	4	3	13	5	2	3	3	6	36	5	2	7	6	6	6	2018Vergnaud#1367	B. melitensis bv1	48	130	BCCN#82-69	Human	1982	Barcelone,Spain
3	4	3	13	3	2	3	3	6	36	5	2	4	3	5	5	2018Vergnaud#0031	B. melitensis bv1	95	190	BCCN#84-7	Human	1984	Spain
3	4	3	13	4	2	3	3	6	36	5	2	5	4	4	6	2007AlDahouk#009	B. melitensis bv2	55	128	BCCN#82-59	Human	1982	Madrid, Spain
3	4	3	13	3	2	3	3	6	36	5	2	8	4	3	4	2018Vergnaud#1278	B. melitensis bv1	95	190	BCCN#93-68	Human	1993	Tarn-et-Garonne, France
3	4	3	13	5	2	3	3	7	36	5	2	5	3	10	3	2010Valdezate#090	B. melitensis	48	142	#20090402	Human	2009	Ciudad Real, Spain
3	4	3	13	5	3	3	3	4	36	5	2	5	3	4	5	2007AlDahouk#057	B. melitensis bv1	53	127	BfR 17	Human	2000	Berlin, Germany
3	4	3	13	4	3	3	3	6	36	5	2	5	3	6	5	2018Vergnaud#0053	B. melitensis bv1	145	264	BCCN#93-4	Human	1993	Ille-et-Vilaine, France

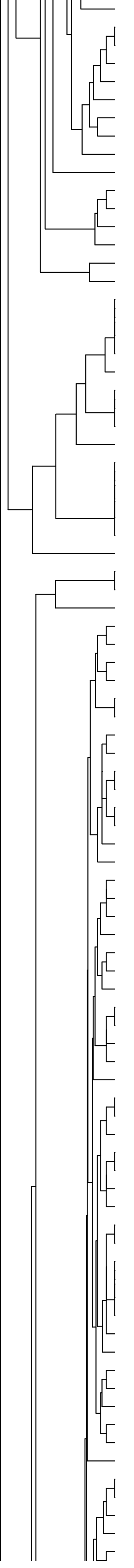

3	4	3	13	4	3	3	3	6	36	5	2	6	3	5	6	2018Vergnaud#0888	B. melitensis bv1	145	264		BCCN#82-56	Human	1982	Madrid ,Spain
3	4	3	14	5	2	3	3	6	36	5	2	4	6	5	4	2007AlDahouk#003	B. melitensis bv1	52	129		BCCN#03-14	Human	2003	Essonne, France
3	4	3	14	5	2	3	3	6	36	5	2	4	6	5	4	2018Vergnaud#0430	B. melitensis bv1	52	129		BCCN#02-8	Human	2002	France
3	4	3	14	4	2	3	3	6	36	5	2	4	6	5	4	2012Ferreira#046	B. melitensis bv1	96	186		AFSSA-04/2179	unknown	2004	Portugal
3	4	3	14	4	2	3	3	6	36	5	2	4	7	5	4	2012Ferreira#045	B. melitensis bv1	96	186		AFSSA-02/6907	unknown	2002	Portugal
3	4	3	14	5	2	3	3	6	36	5	2	4	5	5	5	2012Ferreira#102	B. melitensis bv3	52	129		LNIV-109Bm3-03	unknown	2003	Algarve, Portugal
3	4	3	13	4	2	3	3	6	36	5	2	4	7	7	4	2012Ferreira#119	B. melitensis bv1	55	128		LNIV-767Bm1-07	unknown	2004	Entre Douro e Minho, P.
3	4	3	14	5	2	3	3	6	36	5	2	4	7	7	4	2018Vergnaud#0730	B. melitensis bv1	52	129		BCCN#84-19	Human	1984	France
3	4	3	14	4	2	3	3	6	40	5	2	4	8	6	4	2012Ferreira#105	B. melitensis bv3	96	189		LNIV-214Bm3-03	unknown	2003	Algarve, Portugal
3	4	2	13	3	2	3	3	7	36	5	1	7	3	5	3	2010Valdezate#099	B. melitensis	80	152		#20091322	Human	2009	Madrid, Spain
2	4	2	12	4	2	3	3	9	36	7	2	4	3	3	5	2007AlDahouk#002	B. melitensis bv1	56	126		BCCN#87-92	Human	1987	USA
2	4	2	12	4	2	3	3	5	36	7	2	4	3	3	5	2018Vergnaud#0136	B. melitensis bv1	56	261		BCCN#87-92	Human	1987	Texas, USA
2	4	2	12	3	2	3	3	8	36	7	2	4	3	3	5	SRR857211	B. melitensis	181	351		BA 4837	Human		USA: New Mexico USA
2	4	2	12	3	2	3	3	9	36	7	2	4	3	3	6	SRR857223	B. suis	181	352		2483	Swine		USA: South Carolina
3	4	2	13	4	2	3	3	4	40	8	4	4	1	7	8	2013Jiang#028	B. melitensis bv1	47	262		QH87-029	Bharal	1986	Qinghai, China
3	4	2	13	4	2	3	3	4	40	8	4	4	3	6	10	2013Jiang#029	B. melitensis bv1	47	262		QH86-030	Bharal	1986	Qinghai, China
1	6	23	13	3	2	5	7	10	40	8	14		14		10	2017Al_Dahouk#09	Brucella spp. (.	104	199		10RB9205	African .	2009	Germany
1	6	23	13	3	2	5	7	10	40	8	14		14		10	2017Al_Dahouk#11	Brucella spp. (.	104	199		10RB9207	African .	2009	Germany
1	6	23	13	3	2	5	7	10	40	8	14		14		10	2017Al_Dahouk#12	Brucella spp. (.	104	199		10RB9208	African .	2009	Germany
1	6	23	13	3	2	5	7	10	40	8	14		14		10	2017Al_Dahouk#13	Brucella spp. (.	104	199		10RB9209	African .	2009	Germany
1	6	23	13	3	2	5	7	10	40	8	14		11		10	2017Al_Dahouk#20	Brucella spp. (.	104	199		10RB9216	African .	2009	Germany
1	6	23	13	3	2	5	7	10	40	8	13		12		8	2017Al_Dahouk#01	Brucella spp. (.	104	199		09RB8471	African .	2009	Germany
1	6	23	13	3	2	5	7	10	40	8	13		12		8	2017Al_Dahouk#10	Brucella spp. (.	104	199		10RB9206	African .	2009	Germany
1	6	23	13	3	2	5	7	10	40	8	13		12		8	2017Al_Dahouk#21	Brucella spp. (.	104	199		10RB9217	African .	2009	Germany
1	6	23	13	3	2	5	7	9	40	8	10		16		7	2017Al_Dahouk#14	Brucella spp. (.	104	200		10RB9210	African .	2009	Germany
3	6	26	13	4	2	5	7	10	41	8	10	13		8	8	2017Al_Dahouk#02	Brucella spp. (.	103	197		09RB8908	African .	2009	Germany
3	6	26	13	4	2	5	7	10	41	8	10	13		8	8	2017Al_Dahouk#03	Brucella spp. (.	103	197		09RB8909	African .	2009	Germany
3	6	26	13	4	2	5	7	10	41	8	10	13		8	8	2017Al_Dahouk#04	Brucella spp. (.	103	197		09RB8910	African .	2009	Germany
3	6	26	13	4	2	5	7	10	41	8	10	13		8	8	2017Al_Dahouk#15	Brucella spp. (.	103	197		10RB9211	African .	2009	Germany
3	6	26	13	4	2	5	7	10	41	8	10	13		8	8	2017Al_Dahouk#16	Brucella spp. (.	103	197		10RB9212	African .	2009	Germany
1	5	22	14	3	2	5	5	30	40	8	13	6	3	14		2017Al_Dahouk#17	Brucella spp. (.	101	202		10RB9213	African .	2009	Germany
3	5	8	8	2	2	5	3	4	9	9	5	4	8	6	3	2009Maquart#093	Brucella sp. ST27	78	9		02/611	Human	2002	New Zealand
3	5	8	8	2	2	5	3	4	9	9	5	4	8	6	3	SRR013402	Brucella sp. ST27	78	9	27	02/611	Human	2002	New Zealand
2	5	8	8	2	2	5	3	5	9	9	4	3	6	5	6	SRR011171	Brucella sp. ST27	193	377		F5/99	Bottlen.		
2	5	8	7	3	2	5	1	4	45	9	6	5	3	8	6	2009Maquart#058	B. ceti	23	12		M78/05/2	Porpoise	2005	Scotland
2	5	8	7	3	2	5	1	4	45	9	6	5	3	12	6	2009Maquart#083	B. ceti	23	12		M116/03/2	Porpoise	2003	Scotland
2	5	8	7	3	2	5	1	4	45	9	5	6	3	8	6	2009Maquart#037	B. ceti	23	12		M2788/97/1	AWSD	1997	Scotland
2	5	8	7	3	2	5	1	4	45	9	6	6	3	8	6	2009Maquart#069	B. ceti	23	12		M2788/97/2	AWSD	1997	Scotland
2	5	8	7	3	2	5	1	4	45	9	6	4	3	5	6	2006LeFleche#017	B. ceti	23	12		B1/94	Porpoise	1994	Scotland
2	5	8	7	3	2	5	1	4	45	9	6	4	3	5	6	SRR032594	B. ceti	23	12	23	B1/94	Porpoise	1994	Scotland
2	5	8	7	3	2	5	1	4	45	9	5	6	3	7	4	2009Maquart#028	B. ceti	23	12		M39/94/1	Porpoise	1994	Scotland
2	5	8	7	3	2	5	1	4	45	9	5	5	3	7	4	2009Maquart#056	B. ceti	23	12		M89/03/5	Porpoise	2003	Scotland
2	5	8	7	3	2	5	1	4	45	9	5	6	3	7	6	2009Maquart#059	B. ceti	23	12		M61/05/1	AWSD	2005	Scotland
2	5	8	7	3	2	5	1	4	45	9	5	6	3	7	6	2009Maquart#129	B. ceti	23	12		M199/04/3	Porpoise	2004	Scotland
2	5	8	7	3	2	5	1	4	45	9	5	5	3	7	6	2009Maquart#049	B. ceti	23	12		M499/99/5	Porpoise	1999	Scotland
2	5	8	7	3	2	5	1	4	45	9	5	5	3	7	6	2009Maquart#073	B. ceti	23	12		M499/99/10	Porpoise	1999	Scotland
2	5	8	7	3	2	5	1	4	45	9	5	5	3	7	8	2009Maquart#096	B. ceti	23	12		M83/01/4	Porpoise	2001	Scotland
2	5	8	7	3	2	5	1	4	45	9	3	6	3	7	6	2009Maquart#060	B. ceti	23	12		M199/04/2	Porpoise	2004	Scotland
2	5	8	7	3	2	5	1	4	45	9	5	6	3	7	5	2009Maquart#026	B. ceti	23	12		B202R	Minke .	1995	Norway
2	5	8	7	3	2	5	1	4	45	9	5	6	3	6	5	2009Maquart#107	B. ceti	23	12		M217/03/4	Porpoise	2003	Scotland
2	5	8	7	3	2	5	1	4	45	9	5	6	3	5	5	2009Maquart#061	B. ceti	23	12		M38/04/3	Porpoise	2004	Scotland
2	5	8	7	3	2	5	1	4	45	9	7	6	3	7	5	2009Maquart#057	B. ceti	23	12		M74/05/2	Porpoise	2005	Scotland
2	5	8	7	3	2	5	1	4	45	9	8	6	3	4	5	2009Maquart#041	B. ceti	23	12		M493/99/1	Porpoise	1999	Scotland
2	5	8	7	3	2	5	1	4	45	9	6	6	3	4	5	2009Maquart#121	B. ceti	23	12		M54/07/11	Porpoise	2007	Scotland
2	5	8	7	3	2	5	1	4	45	9	6	6	3	12	5	2009Maquart#063	B. ceti	23	12		M93/04/3	Porpoise	2004	Scotland
2	5	8	7	3	2	5	1	4	45	9	7	7	3	4	5	2009Maquart#065	B. ceti	23	12		M234/05/	Porpoise	2005	Scotland
2	5	8	7	3	2	5	1	4	45	9	7	7	3	4	5	2009Maquart#123	B. ceti	23	12		M234/05/2	Porpoise	2005	Scotland
2	5	8	7	3	2	5	1	4	45	9	7	5	3	4	5	2009Maquart#055	B. ceti	23	12		M23/03/4	Porpoise	2003	Scotland
2	5	8	7	3	2	5	1	4	45	9	7	6	3	4	5	2009Maquart#094	B. ceti	23	12		M90/06/4	Porpoise	2006	Scotland
2	5	8	7	3	2	5	1	4	45	9	8	7	3	7	5	2009Maquart#101	B. ceti	23	12		M52/01/2	AWSD	2001	Scotland
2	5	8	7	3	2	5	1	4	45	9	7	5	3	5	5	2009Maquart#007	B. ceti	23	12		M181/97/1	AWSD	1997	Scotland
2	5	8	7	3	2	5	1	4	45	9	7	5	3	5	5	2009Maquart#090	B. ceti	23	12		M120/05/1	Porpoise	2005	Scotland
2	5	8	7	3	2	5	1	4	45	9	7	5	3	7	5	2009Maquart#008	B. ceti	23	12		M870/97/1	White-b.	1997	Scotland
2	5	8	7	3	2	5	1	4	45	9	6	5	3	7	5	2009Maquart#089	B. ceti	23	12		M100/04/6	Porpoise	2004	Scotland
2	5	8	7	3	2	5	1	4	45	9	6	5	3	7	5	2009Maquart#117	B. ceti	23	12		M100/04/7	Porpoise	2004	Scotland
2	5	8	7	3	2	5	1	4	45	9	6	5	3	9	5	2009Maquart#052	B. ceti	23	12		M187/00/1	AWSD	2000	Scotland
2	5	8	7	3	2	5	1	4	45	9	6	5	3	5	5	2009Maquart#036	B. ceti	23	12		M452/97/2	Commo.	1997	Scotland
2	5	8	7	3	2	5	1	4	45	9	5	5	3	8	5	2009Maquart#111	B. ceti	23	12		M245/05/8	Porpoise	2005	Scotland
2	5	8	7	3	2	5	1	4	45	9	5	5	3	8	5	2009Maquart#131	B. ceti	23	12		M245/05/4	Porpoise	2005	Scotland
2	5	8	7	3	2	5	1	4	45	9	5	5	3	6	5	2009Maquart#070	B. ceti	23	12		M103/99/2	Porpoise	1999	Scotland
2	5	8	7	3	2	5	1	4	45	9	5	5	3	6	5	2009Maquart#099	B. ceti	23	12		M39/06/6	Porpoise	2006	Scotland
2	5	8	7	3	2	5	1	4	45	9	5	5	3	6	5	2009Maquart#115	B. ceti	23	12		M99/07/3	Porpoise	2007	Scotland
2	5	8	7	3	2	5	1	4	45	9	5	5	3	6	5	2009Maquart#133	B. ceti	23	12		M99/07/2	Porpoise	2007	Scotland
2	5	8	7	3	2	5	1	4	45	9	5	5	3	7	5	2009Maquart#006	B. ceti	23	12		M1661/94/2	Porpoise	1994	Scotland
2	5	8	7	3	2	5	1	4	45	9	5	5	3	6	7	2009Maquart#103	B. ceti	23	12		M181/07/7	Porpoise	2007	Scotland
2	5	8	7	3	2	5	1	4	45	9	4	4	3	5	5	2009Maquart#039	B. ceti	23	12		M1747/98/3	Porpoise	1998	Scotland
2	5	8	7	3	2	5	1	4	45	9	4	5	3	5	5	2009Maquart#118	B. ceti	23	12		M186/07/12	Porpoise	2007	Scotland
2	5	8	7	3	2	5	1	4	45	9	4	5	3	8	5	2009Maquart#062	B. ceti	23	12		M51/04/2	Porpoise	2004	Scotland
2	5	8	7	3	2	5	1	4	45	9	4	6	3	6	5	2009Maquart#033	B. ceti	23	12		M2438/95/1	AWSD	1995	Scotland
2	5	8	7	3	2	5	1	4	45	9	4	5	3	6	5	2009Maquart#108	B. ceti	23	12		M99/07/1	Porpoise	2007	Scotland
2	5	8	7	3	2	5	1	4	45	9	9	5	3	5	8	2009Maquart#085	B. ceti	23	12		M150/03/3	Porpoise	2003	Scotland
2	5	8	7	3	2	5	1	4	45	9	6	6	3	6	4	2009Maquart#048	B. ceti	23	12		M2/00/1	AWSD	2000	Scotland
2	5	8	7	3	2	5	1	4	45	9	6	6	3	6	4	2009Maquart#116	B. ceti	23	12		M2/00/4	AWSD	2000	Scotland
2	5	8	7	3	2	5	1	4	45	9	5	6	3	6	4	2009Maquart#084	B. ceti	23	12		M130/03/2	Porpoise	2003	Scotland
2	5	8	7	3	2	5	1	4	45	9	6	5	3	6	4	2009Maquart#127	B. ceti	23	12		M85/06/3	Porpoise	2006	Scotland
2	5	8	7	3	2	5	1	4	45	9	6	5	3	7	4	2009Maquart#042	B. ceti	23	12		M610/99/1	Porpoise	1999	Scotland

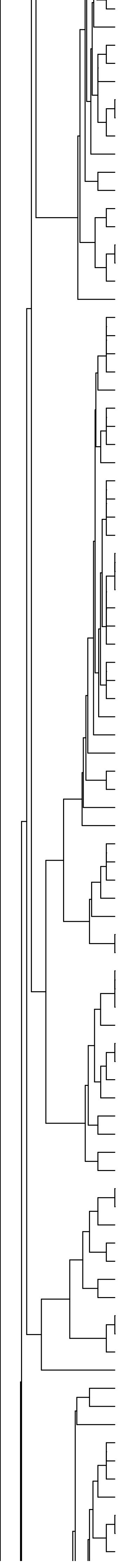

2	5	8	7	3	2	5	1	4	45	9	6	7	3	7	4	2009Maquart#047	B. ceti	23	12		M575/99/4	Porpoise	1999	Scotland
2	5	8	7	3	2	5	1	4	45	9	8	6	3	3	4	2009Maquart#074	B. ceti	23	12		M12/00/3	Porpoise	2000	Scotland
2	5	8	7	3	2	5	1	4	45	9	4	8	3	5	4	2009Maquart#038	B. ceti	23	12		M854/98/8	Porpoise	1998	Scotland
2	5	8	7	3	2	5	1	4	45	9	4	5	3	5	4	2009Maquart#077	B. ceti	23	12		M130/02/2	Porpoise	2002	Scotland
2	5	8	7	3	2	5	1	4	45	9	4	6	5	5	4	2009Maquart#035	B. ceti	23	12		M515/96/2	Porpoise	1996	Scotland
2	5	8	7	3	2	5	1	4	45	9	4	6	3	8	4	2009Maquart#095	B. ceti	23	12		M55/07/1	Porpoise	2007	Netherlands
2	5	8	7	3	2	5	1	4	45	9	4	6	3	8	4	2009Maquart#114	B. ceti	23	12		M67/07/3	Porpoise	2007	Scotland
2	5	8	7	3	2	5	1	4	45	9	4	6	3	7	4	2009Maquart#043	B. ceti	23	12		M563/99/2	Porpoise	1999	Scotland
2	5	8	7	3	2	5	1	4	45	9	7	7	3	8	4	2009Maquart#120	B. ceti	23	12		M311/03/5	Porpoise	2003	Scotland
2	5	8	7	3	2	5	1	4	45	9	4	5	4	4	4	2009Maquart#076	B. ceti	23	12		M117/01/1	Porpoise	2001	Scotland
2	5	8	7	3	2	5	1	4	45	9	4	5	3	4	6	2009Maquart#109	B. ceti	23	12		M49/01/2	Porpoise	2001	Scotland
2	5	8	7	2	2	5	1	4	45	9	7	4	3	7	6	2009Maquart#030	B. ceti	69	11		M997/94/2	AWSD	1994	Scotland
2	5	8	7	2	2	5	1	4	45	9	7	5	3	7	6	2009Maquart#064	B. ceti	69	11		M89/05/1	Porpoise	2005	Scotland
2	5	8	7	2	2	5	1	4	45	9	7	8	3	5	6	SRR032591	B. ceti	69	11	23	M490/95/1	Commo.	1995	Scotland
2	5	8	7	2	2	5	1	4	45	9	7	8	3	5	6	SRR032592	B. ceti	69	11	23	M490/95/1	Commo.	1995	Scotland
2	5	8	7	3	2	5	1	4	45	9	7	8	3	5	6	2009Maquart#032	B. ceti	23	12		M490/95/1	Commo.	1995	Scotland
1	5	8	7	3	2	5	1	4	45	9	7	4	3	6	4	2009Maquart#087	B. ceti	70	10		M195/03/10	Porpoise	2003	Scotland
3	5	6	13	3	2	5	4	7	43	9	5	6	6	3	3	2009Maquart#018	B. pinnipedialis	25	22		#11232	Commo.	2002	Germany
3	5	6	13	3	2	5	4	7	43	9	5	6	8	3	3	2009Maquart#022	B. pinnipedialis	25	22		#11256	Commo.	2002	Germany
3	5	6	13	3	2	5	4	7	43	9	5	6	7	3	3	2009Maquart#019	B. pinnipedialis	25	22		#11234	Commo.	2002	Germany
3	5	6	13	3	2	5	4	7	43	9	5	6	4	3	3	2009Maquart#021	B. pinnipedialis	25	22		#11255	Commo.	2002	Germany
3	5	6	13	3	2	5	4	7	43	9	6	6	5	3	3	2009Maquart#029	B. pinnipedialis	25	22		M339/94/1	Commo.	1994	Scotland
3	5	6	13	3	2	5	4	7	43	9	5	4	5	3	3	2009Maquart#013	B. pinnipedialis	25	22		#8155	Commo.	1999	Germany
3	5	6	13	3	2	5	4	7	43	9	5	4	7	3	3	2009Maquart#016	B. pinnipedialis	25	22		#8241	Commo.	2000	Germany
3	5	6	13	3	2	5	4	7	43	9	5	4	6	3	3	2009Maquart#015	B. pinnipedialis	25	22		#8172	Commo.	2000	Germany
3	5	6	13	3	2	5	4	7	43	9	5	4	5	4	3	2009Maquart#014	B. pinnipedialis	25	22		#8170	Commo.	2000	Germany
3	5	6	13	3	2	5	4	7	43	9	5	5	7	3	3	2009Maquart#009	B. pinnipedialis	25	22		#8105	Commo.	1998	Germany
3	5	6	13	3	2	5	4	7	43	9	5	5	8	3	3	2009Maquart#023	B. pinnipedialis	25	22		#11265	Commo.	2003	Germany
3	5	6	13	3	2	5	4	7	43	9	5	5	4	3	3	2009Maquart#010	B. pinnipedialis	25	22		#8150	Commo.	1998	Germany
3	5	6	13	3	2	5	4	7	43	9	5	5	9	3	3	2009Maquart#020	B. pinnipedialis	25	22		#11235	Commo.	2002	Germany
3	5	6	13	3	2	5	4	7	43	9	5	5	5	3	3	2006LeFleche#019	B. pinnipedialis	25	22		B2/94	Commo.	1994	Scotland
3	5	6	13	3	2	5	4	7	43	9	5	5	5	3	3	GCA_000221005	B. pinnipedialis	25	22	25	B2/94	Commo.	2011	Scotland
3	5	6	13	3	2	5	4	7	43	9	5	5	5	3	3	SRR032867	B. pinnipedialis	25	22	25	B2/94	Commo.	2011	Scotland
3	5	6	13	3	2	5	4	7	43	9	7	5	5	3	3	2009Maquart#003	B. pinnipedialis	25	22		M972/94/1	Commo.	1994	Scotland
3	5	6	13	3	2	5	4	7	43	9	6	5	5	3	3	2009Maquart#005	B. pinnipedialis	25	22		M514/96/4	Commo.	1996	Scotland
3	5	6	13	3	2	5	4	7	43	9	2	5	5	3	3	2009Maquart#025	B. pinnipedialis	25	22		#11267	Commo.	2004	Germany
3	5	6	13	3	2	5	4	7	43	9	5	5	6	3	3	2009Maquart#004	B. pinnipedialis	25	22		M1771/94/1	Otter	1994	Scotland
3	5	6	13	3	2	5	4	7	43	9	7	5	6	3	3	2009Maquart#075	B. pinnipedialis	25	22		M13/01/1	Commo.	2001	Scotland
3	5	6	13	3	2	5	4	7	43	9	6	5	6	3	3	2009Maquart#024	B. pinnipedialis	25	22		#11266	Commo.	2003	Germany
3	5	6	13	3	2	5	4	6	43	9	5	5	5	3	3	2009Maquart#051	B. pinnipedialis	25	18		M194/00/1	Gray seal	2000	Scotland
3	5	6	13	3	2	5	4	7	43	9	5	5	7	4	3	2009Maquart#017	B. pinnipedialis	25	22		#8244	Commo.	2000	Germany
3	5	3	13	3	2	5	4	7	43	9	5	4	3	3	3	2009Maquart#011	B. pinnipedialis	73	21		#8169	Commo.	1998	Germany
3	5	6	13	3	2	5	2	7	43	9	4	5	6	3	3	2009Maquart#027	B. pinnipedialis	72	20		M2466/93/4	Commo.	1993	Scotland
3	5	6	13	3	2	5	2	7	43	9	4	5	9	3	3	2009Maquart#124	B. pinnipedialis	72	20		M374/02/2	Commo.	2002	Scotland
2	5	6	13	3	2	5	4	7	43	9	4	4	6	3	3	GCA_000740275	B. pinnipedialis	174	359	52	6/566	unknown	unk.	unknown
3	5	6	13	3	2	5	4	7	45	9	6	6	4	3	3	2009Maquart#045	B. pinnipedialis	25	19		M621/99/2	Gray seal	1999	Scotland
2	5	6	12	3	2	5	3	7	43	9	6	4	6	5	3	2009Maquart#001	B. pinnipedialis	71	17		M2533/93/1	Commo.	1993	Scotland
2	5	6	12	3	2	5	3	7	43	9	6	4	5	5	3	2009Maquart#012	B. pinnipedialis	71	17		#8151	Gray seal	1998	Germany
2	5	6	12	3	2	5	3	7	43	9	6	4	7	5	3	2009Maquart#054	B. pinnipedialis	71	17		M305/02/1	Commo.	2002	Scotland
2	5	6	13	3	2	5	3	7	43	9	6	4	7	5	3	2009Maquart#130	B. pinnipedialis	79	16		M4/06/4	Gray seal	2006	Scotland
2	5	6	12	3	2	5	3	7	43	9	5	5	7	5	3	2009Maquart#050	B. pinnipedialis	71	17		M192/00/1	Minke .	2000	Scotland
2	5	6	12	3	2	5	3	7	43	9	5	4	6	6	3	2009Maquart#002	B. pinnipedialis	71	17		M292/94/1	Commo.	1994	Scotland
2	5	6	12	3	2	5	3	7	43	9	5	4	6	6	3	SRR032866	B. pinnipedialis	71	17	24	M292/94/1	Commo.	1994	Scotland
3	5	15	12	3	2	5	7	7	45	9	4	4	6	7	4	2009Maquart#067	B. pinnipedialis	76	15		37f-1	Hooded.	2002	Norway
3	5	15	12	3	2	5	7	7	45	9	4	4		7	4	2009Maquart#078	B. pinnipedialis	76	15		9a-1	Hooded.	2002	Norway
3	5	15	12	3	2	5	7	7	45	9	4	4	6	7	4	2009Maquart#091	B. pinnipedialis	76	15		M603/99/7	Hooded.	1999	Scotland
3	5	15	12	3	2	5	7	7	45	9	4	4	5	5	4	2009Maquart#068	B. pinnipedialis	76	15			Hooded.	2002	Norway
3	5	15	12	3	2	5	7	7	45	9	4	4	6	4	6	2009Maquart#066	B. pinnipedialis	76	15		22f-1	Hooded.	2002	Norway
3	5	15	12	3	2	5	7	7	45	9	4	4	6	4	6	2009Maquart#079	B. pinnipedialis	76	15		22a-2	Hooded.	2002	Norway
3	5	15	12	3	2	5	7	7	45	9	4	4	6	6	6	2009Maquart#031	B. pinnipedialis	76	15		M2006/94/6	Hooded.	1994	Scotland
3	5	15	12	3	2	5	7	7	45	9	4	4	7	6	6	2009Maquart#086	B. pinnipedialis	76	15		53c-1	Hooded.	2002	Norway
3	5	15	12	3	2	5	7	7	45	9	5	4	6	5	4	2009Maquart#071	B. pinnipedialis	76	15		M163/99/10	Hooded.	1999	Scotland
3	5	14	12	3	2	5	7	7	45	9	6	4	6	5	4	2009Maquart#080	B. pinnipedialis	75	14		23a-1	Hooded.	2002	Norway
3	5	15	12	3	2	5	7	7	45	9	3	4	5	6	4	2009Maquart#081	B. pinnipedialis	76	15		25a-1	Hooded.	2002	Norway
3	5	12	12	3	2	5	7	7	45	9	5	4	5	6	4	2009Maquart#082	B. pinnipedialis	74	13		30a-1	Hooded.	2002	Norway
4	5	4	13	4	3	5	1	6	41	9	3	4	3	6	3	2017aSuárez-Esqu.	B. neotomae	26	6		bneohCR01	Human	2011	Costa Rica
4	5	4	13	4	3	5	1	6	41	9	3	4	3	6	3	2017aSuárez-Esqu.	B. neotomae	26	6		bneohCR02	Human		Costa Rica
4	5	4	13	4	3	5	1	6	41	9	3	6	3	6	3	2006LeFleche#020	B. neotomae	26	6	22	REF 5K33	Rodent	1957	Utah desert,USA
3	5	4	13	4	3	5	1	6	41	9	3	4	3	5	3	2018Vergnaud#0138	B. neotomae	143	231	22	BCCN#R34	Rodent		Utah desert,USA
1	5	4	13	4	3	5	1	6	41	9	3	4	3	5	3	2018Vergnaud#0144	B. neotomae	123	230	22	BCCN#R40	Rodent		Utah desert,USA
3	5	4	13	4	3	5	1	6	41	9	4	6	3	10	3	2018Vergnaud#0139	B. neotomae	143	231	22	BCCN#R35	Rodent		Utah desert,USA
4	5	4	13	4	3	5	1	6	41	9	4	6	3	11	3	2018Vergnaud#0143	B. neotomae	26	6	22	BCCN#R39	Rodent		Utah desert,USA
3	5	4	13	4	2	5	1	6	41	9	4	7	6	5	3	2018Vergnaud#0140	B. neotomae	150	229	120	BCCN#R36	Rodent		Utah desert,USA
3	5	4	13	4	2	5	1	6	41	9	4	7	6	5	3	2018Vergnaud#0142	B. neotomae	150	229	120	BCCN#R38	Rodent		Utah desert,USA
4	5	4	13	4	2	5	1	6	41	9	4	7	6	5	3	2018Vergnaud#0141	B. neotomae	129	228	120	BCCN#R37	Rodent		Utah desert,USA
4	5	20	12	1	2	5	6	6	41	9	6	5	3	7	3	2018Vergnaud#1405	Brucella sp. ST66	183	360	66	97-3288	Human		Thailand
2	5	8	13	2	2	4	2	6	12	9	8	12	6	18	3	2014Isidoro-Ayza.	B. ceti	24	379		bmarMR24	Striped .	2012	Spain
2	5	5	13	2	2	4	2	6	12	9	8	5	6	11	3	2017bSuárez-Esqu.	B. ceti	189	384		bmarCR5	Striped .	2006	Costa Rica
2	5	8	13	2	2	4	2	6	12	9	12	6	15	19	3	2014Isidoro-Ayza.	B. ceti	24	379		bmarMR25	Bottlen.	2012	Spain
2	5	8	13	2	2	4	2	6	11	9	8	6	8	8	3	2009Maquart#034	B. ceti	24	8		M18/96/1	AWSD	1996	Scotland
2	5	8	13	2	2	4	2	6	11	9	8	6	6	8	3	2009Maquart#110	B. ceti	24	8		M57/07/1	AWSD	2007	Scotland
2	5	8	13	2	2	4	2	6	11	9	8	6	7	8	3	2009Maquart#134	B. ceti	24	8		M260/03/1	AWSD	2003	Scotland
2	5	8	13	2	2	4	2	6	11	9	8	6	11	14	3	2009Maquart#097	B. ceti	24	8		M42/07/1	AWSD	2007	Scotland
2	5	8	13	2	2	4	2	6	11	9	8	7	8	14	3	2009Maquart#053	B. ceti	24	8		M9/02/01	Striped .	2002	Scotland
2	5	8	13	2	2	4	2	6	11	9	8	7	8	14	3	SRR032593	B. ceti	24	8	26	M644/93/1			
2	5	8	13	2	2	4	2	6	11	9	8	7	9	14	3	2009Maquart#088	B. ceti	24	8		M13/05/1	Striped .	2005	Scotland

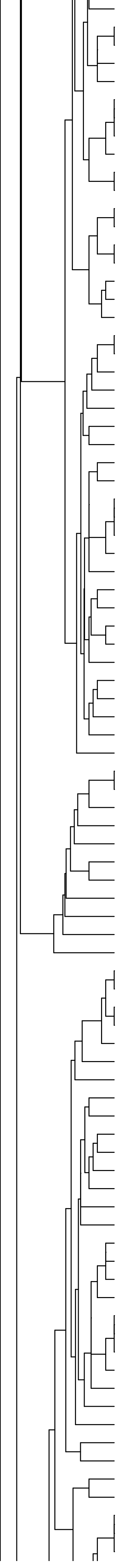

2	5	8	13	2	2	4	2	6	11	9	8	11	10	18	3	2009Maquart#102	B. ceti	24	8		M222/07/1	AWSD	2007	Scotland
2	5	8	13	2	2	4	2	6	11	9	8	8	8	15	3	2009Maquart#072	B. ceti	24	8		M656/99/2	Striped .	1999	Scotland
2	5	8	13	2	2	4	2	6	11	9	8	8	8	15	3	2009Maquart#092	B. ceti	24	8		M656/99/1	Striped .	1999	Scotland
2	5	8	13	2	2	4	2	6	11	9	9	8	7	15	3	2009Maquart#100	B. ceti	24	8		M158/06/1	AWSD	2006	Scotland
2	5	8	13	2	2	4	2	6	11	9	9	5	8	15	3	2009Maquart#040	B. ceti	24	8		M2194/94/1	Striped .	1994	Scotland
2	5	8	13	2	2	4	2	6	11	9	9	5	9	16	3	2009Maquart#106	B. ceti	24	8		M52/07/9	AWSD	2007	Scotland
2	5	8	13	2	2	4	2	6	11	9	9	5	9	16	3	2009Maquart#112	B. ceti	24	8		M52/07/1	AWSD	2007	Scotland
2	5	8	13	2	2	4	2	6	11	9	9	5	9	16	3	2009Maquart#119	B. ceti	24	8		M52/07/7	AWSD	2007	Scotland
2	5	8	13	2	2	4	2	6	11	9	9	8	9	16	3	2009Maquart#125	B. ceti	24	8		M35/08/2	Minke .	2008	Scotland
2	5	8	13	2	2	4	2	6	11	9	9	14	6	7	3	2013Garofolo_10.	B. ceti	24	8		10759	Striped .	2012	Lido Pizzo, Gallipoli, Italy
2	5	8	13	2	2	4	2	6	11	9	9	14	6	7	3	2013Garofolo_28.	B. ceti	24	8		28753	Striped .	2012	Porto Cesareo Bacino .
2	5	8	13	1	2	4	2	6	11	9	11	11	7	5	3	2009Maquart#105	B. ceti	77	7		M267/05/4	AWSD	2005	Scotland
2	5	8	13	1	2	4	2	6	11	9	11	11	7	5	3	2009Maquart#122	B. ceti	77	7		M267/05/1	AWSD	2005	Scotland
2	5	8	13	1	2	4	2	6	11	9	11	10	7	9	3	2009Maquart#098	B. ceti	77	7		M83/07/1	AWSD	2007	Scotland
2	5	8	13	1	2	4	2	6	11	9	11	10	7	9	3	2009Maquart#113	B. ceti	77	7		M83/07/3	AWSD	2007	Scotland
2	5	8	13	1	2	4	2	6	11	9	12	8	7	12	3	2009Maquart#044	B. ceti	77	7		M654/99/1	Striped .	1999	Scotland
2	5	8	13	1	2	4	2	6	11	9	13	8	7	12	3	2009Maquart#126	B. ceti	77	7		M231/07/2	Bottlen.	2007	Scotland
2	5	8	13	1	2	4	2	6	11	9	13	8	7	13	3	2009Maquart#104	B. ceti	77	7		M231/07/3	Bottlen.	2007	Scotland
2	5	7	13	2	2	4	2	6	12	9	7	11	7	6	3	2017bSuárez-Esqu.	B. ceti	176	378		bmarCR8	Striped .	2009	Costa Rica
2	5	7	13	2	2	4	2	6	12	9	7	11	7	6	3	2017bSuárez-Esqu.	B. ceti	176	378		bmarCR8w alia.	Striped .	2009	Costa Rica
2	5	7	13	2	2	4	2	6	12	9	7	11	13	8	3	2017bSuárez-Esqu.	B. ceti	176	378		bmarCR4	Striped .	2007	Costa Rica
2	5	7	13	2	2	4	2	6	12	9	7	10	8	8	3	2017bSuárez-Esqu.	B. ceti	176	378		bmarCR1	Striped .	2006	Costa Rica
2	5	8	13	2	2	4	2	6	12	9	7	11	6	21	3	2014Isidoro-Ayza.	B. ceti	24	379		bmarMR26	Striped .	2009	Spain
2	5	7	13	2	2	4	2	6	12	9	8	11	15	13	3	2017bSuárez-Esqu.	B. ceti	176	378		bmarCR18	Striped .	2013	Costa Rica
2	5	7	13	2	2	4	2	6	12	9	8	10	16	9	3	2017bSuárez-Esqu.	B. ceti	176	378		bmarCR19	Striped .	2013	Costa Rica
2	5	7	13	2	2	4	2	6	12	9	6	7	9	7	3	2017bSuárez-Esqu.	B. ceti	176	378		bmarCR7	Striped .	2008	Costa Rica
2	5	7	13	2	2	4	2	6	12	9	6	7	11	12	3	2017bSuárez-Esqu.	B. ceti	176	378		bmarCR16	Striped .	2012	Costa Rica
2	5	7	13	2	2	4	2	6	12	9	6	14	12	10	3	2017bSuárez-Esqu.	B. ceti	176	378		bmarCR20	Striped .	2013	Costa Rica
2	5	7	13	2	2	4	2	6	12	9	6	14	12	10	3	2017bSuárez-Esqu.	B. ceti	176	378		bmarCR20w	Striped .	2013	Costa Rica
2	5	7	13	2	2	4	2	6	12	9	6	14	12	10	3	2017bSuárez-Esqu.	B. ceti	176	378		bmarCR20lung	Striped .	2013	Costa Rica
2	5	7	13	2	2	4	2	6	12	9	6	14	13	10	3	2017bSuárez-Esqu.	B. ceti	176	378		bmarCR13	Striped .	2011	Costa Rica
2	5	7	13	2	2	4	2	6	12	9	6	12	14	9	3	2017bSuárez-Esqu.	B. ceti	176	378		bmarCR9	Striped .	2009	Costa Rica
2	5	7	13	2	2	4	2	6	12	9	7	14	9	11	3	2017bSuárez-Esqu.	B. ceti	176	378		bmarCR11	Striped .	2011	Costa Rica
2	5	7	13	2	2	4	2	6	12	9	10	9	9	11	3	2017bSuárez-Esqu.	B. ceti	176	378		bmarCR14	Striped .	2011	Costa Rica
2	5	7	13	2	2	4	2	6	12	9	7	13	9	10	3	2017bSuárez-Esqu.	B. ceti	176	378		bmarCR2	Striped .	2006	Costa Rica
2	5	7	13	2	2	4	2	6	12	9	8	13	9	10	3	2017bSuárez-Esqu.	B. ceti	176	378		bmarCR12	Striped .	2011	Costa Rica
2	5	7	13	2	2	4	2	6	12	9	5	13	8	11	3	2017bSuárez-Esqu.	B. ceti	176	378		bmarCR6	Striped .	2007	Costa Rica
2	5	7	13	2	2	4	2	6	12	9	5	6	13	8	3	2017bSuárez-Esqu.	B. ceti	176	378		bmarCR15	Striped .	2012	Costa Rica
2	5	7	13	2	2	4	2	6	12	9	5	6	15	10	3	2017bSuárez-Esqu.	B. ceti	176	378		bmarCR17	Striped .	2012	Costa Rica
2	5	7	13	2	2	4	2	6	12	9	5	16	11	10	3	2017bSuárez-Esqu.	B. ceti	176	378		bmarCR21	Striped .	2014	Costa Rica
2	5	7	13	2	2	4	2	6	12	9	5	9	5	12	3	2017bSuárez-Esqu.	B. ceti	176	378		bmarCR10	Striped .	2010	Costa Rica
2	5	7	11	2	2	4	2	6	12	9	14	11	9	11	3	2017bSuárez-Esqu.	B. ceti	190	385		bmarCR3	Striped .	2007	Costa Rica
4	5	12	13	5	2	5	6	9	7	9	8	5	7	13	5	2008Scholz#001	B. microti	67	5		CCM4915 ((IMB.	Rodent		Czech Republic
4	5	12	13	5	2	5	6	9	7	9	8	5	7	13	5	GCA_000022745	B. microti	67	5	67	CCM4915	Rodent	2009	Czech Republic
4	5	12	13	5	2	5	6	10	7	9	8	6	7	11	5	2012AlDahouk#002	B. microti	67	192		FK 21908	Red fox		Austria
4	5	12	13	5	2	5	6	9	7	9	6	7	10	12	5	2012AlDahouk#005	B. microti	67	5		BMS 10	soil	2008	South Moravia, Czech .
4	5	12	13	5	2	5	6	9	7	9	9	5	11	10	6	2012AlDahouk#004	B. microti	67	5		F303M	Red fox		Austria
4	5	12	13	5	2	5	6	8	7	9	7	6	6	7	5	2008Scholz#002	B. microti	67	4		CCM4916 ((IMB.	Rodent		Czech Republic
4	5	12	13	5	2	5	6	8	7	9	7	7	10	7	6	2008Scholz#005	B. microti	67	4		(IMB) 257	Red fox		Austria
4	5	12	13	5	2	5	6	7	7	9	10	13	12	4	6	2015Kreizinger#001	B. microti	67	232		HUN-Bmi-01	Wild boar	2014	Rajka, Hungary
4	5	12	13	5	2	5	10	6	7	9	10	6	9	11	5	2012AlDahouk#003	B. microti	81	194		FW 70608	Red fox		Austria
4	5	12	13	5	2	5	6	8	7	6	9	8	7	12	6	2012AlDahouk#001	B. microti	67	193		FH 2208	Red fox		Austria
4	5	12	13	4	2	5	10	6	7	9	8	7	3	13	4	2008Scholz#006	B. microti	68	3		(IMB) 284	Red fox		Austria
2	3	9	11	3	1	5	2	5	36	9	4	5	8	6	3	GCA_000740435	B. suis	3	27	78	BSP			
2	3	9	11	3	1	5	2	5	36	9	4	5	8	6	3	SRR015442	B. suis bv4	3	27	78	REF 40	Deer/El.		
2	3	9	11	3	1	5	2	5	36	9	4	5	9	6	3	2006LeFleche#015	B. suis bv4	3	27		REF 40	Deer/El.		Former USSR
2	3	9	11	3	1	5	2	5	36	9	4	5	9	6	3	2017Ferreira#016	B. suis bv4	3	27		REF 40 [biovar .	Deer/El.		Former USSR
2	3	9	10	3	1	5	2	5	36	9	4	5	9	6	3	2017Ferreira#017	B. suis bv4	5	260		VLA-63/202	Deer/El.	1963	Poland
2	3	9	10	3	1	5	2	5	36	9	8	5	10	9	3	2017Ferreira#018	B. suis bv4	5	260		VLA-63/219	Deer/El.	1963	Poland
2	3	9	10	3	1	5	2	5	36	9	12	8	16	5	3	2017Ferreira#019	B. suis bv4	5	260		VLA-63/198	Deer/El.	1963	Poland
2	3	9	11	3	1	5	2	4	40	9	5	5	10	10	3	2006LeFleche#189	B. suis bv4	3	29		BCCN#87-57	Human	1987	Canada
2	3	9	11	3	1	5	2	4	40	9	6	9	6	10	3	2006LeFleche#190	B. suis bv4	3	29		BCCN#87-58	Human	1987	Canada
2	3	9	11	3	1	5	2	5	40	9	6	5	14	8	3	2006LeFleche#185	B. canis	3	26		BCCN#96-121	Dog	1996	
2	3	9	11	3	1	5	2	5	40	9	5	5	7	8	3	GCA_000238195	B. canis	3	26	21	HSK A52141	Dog	2011	Hwasung, Geyonggi, R.
2	3	9	11	3	1	5	2	5	40	9	6	5	9	10	3	GCA_001715365	B. canis	3	26	77	2009004498			
2	3	9	11	3	1	5	2	4	40	9	6	5	7	9	3	2006LeFleche#192	B. suis bv4	3	29		BCCN#87-60	Musk ox	1987	Canada
2	3	9	11	3	1	5	2	8	40	9	10	5	8	9	3	GCA_000691585	B. canis	3	355	77	SVA13	Dog	2013	Sweden
2	3	9	11	3	1	5	2	6	40	9	7	5	9	7	3	2006LeFleche#147	B. canis	3	28		BfR 132	Dog	1976	
2	3	9	11	3	1	5	2	5	40	9	8	6	7	5	3	2006LeFleche#018	B. canis	3	26		REF RM6/66	Dog		USA
2	3	9	11	3	1	5	2	5	40	9	8	6	10	5	3	2006LeFleche#182	B. canis	3	26		BCCN#87-66	Dog	1987	Canada
2	3	9	11	3	1	5	2	5	40	9	8	6	8	5	3	2018Vergnaud#0008	B. canis	3	26		BCCN#R18	Dog		USA
2	3	9	11	3	1	5	2	5	40	9	8	6	6	6	3	2006LeFleche#184	B. canis	3	26		BCCN#96-107	Dog	1996	Romania
2	3	9	11	3	1	5	2	5	40	9	7	6	10	7	3	2006LeFleche#186	B. canis	3	26		BCCN#97-3	Dog	1997	France
2	3	9	11	3	1	5	2	5	40	9	7	6	10	7	3	GCA_000018525	B. canis	3	26	20	REF RM6/66 (.	Dog	2007	USA
2	3	9	11	3	1	5	2	5	40	9	7	6	10	7	3	GCA_000740335	B. canis	3	26	20	REF RM6/66 (.	Dog		
2	3	9	11	3	1	5	2	5	40	9	7	6	7	7	3	2006LeFleche#183	B. canis	3	26		BCCN#96-105	Dog	1996	Romania
2	3	9	11	3	1	5	2	5	40	9	4	6	7	10	3	GCA_001715405	B. canis	3	26	21	2010009751			
2	3	9	11	3	1	5	2	5	40	9	6	7	8	5	3	2006LeFleche#146	B. canis	3	26		BfR 130	Dog	2001	Greece
2	3	9	11	3	1	5	2	4	40	9	8	4	5	12	3	2006LeFleche#191	B. suis bv4	3	29		BCCN#87-59	Caribou	1987	Canada
2	3	9	10	3	1	5	2	4	40	9	6	6	7	7	3	2006LeFleche#187	B. suis bv4	5	30		BCCN#3609(.	Caribou		
2	3	9	10	3	1	5	2	4	40	9	5	6	11	9	5	2017Ferreira#015	B. suis bv4	5	30		VLA-63/252	Caribou	1963	Alaska
2	3	4	11	3	1	5	2	4	38	9	9	7	7	8	6	2017Liu#179	B. suis bv3	4	31		NM-10		2016	
2	3	4	11	3	1	5	2	4	38	9	8	7	7	7	3	2017Liu#180	B. suis bv3	4	31		NM-19		2016	
2	3	4	11	3	1	5	2	4	38	9	7	5	10	4	5	2006LeFleche#014	B. suis bv3	4	31		REF 686	Swine		USA
2	3	4	11	3	1	5	2	4	38	9	7	5	10	4	5	2018Vergnaud#0173	B. suis bv2	4	31		BCCN#06-63		2006	United Kingdom
2	3	4	11	3	1	5	2	4	38	9	7	5	10	4	5	GCA_000740255	B. suis bv3	4	31	17	REF 686			

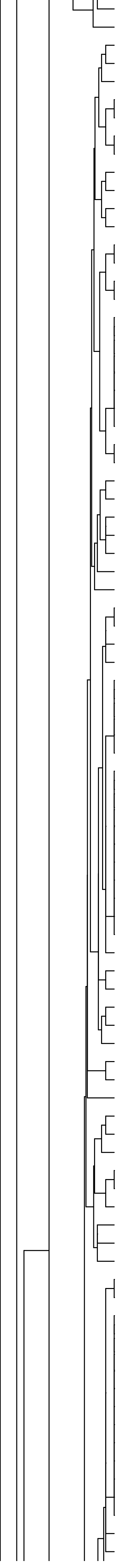

2	3	4	11	3	1	5	2	4	38	9	8	6	10	4	5	2017Ferreira#013	B. suis bv3	4	31		REF 686 [biova.	Swine		USA
2	3	4	11	3	1	5	2	4	38	9	7	5	12	4	7	2017Ferreira#014	B. suis bv3	4	31		VLA-UK6/04	Human	2004	India
2	3	6	10	4	1	5	2	4	38	9	5	7	8	5	3	SRR2058957	B. suis bv1	6	33	75	B11-0525	Cattle		USA:HI
2	3	6	10	4	1	5	2	4	38	9	5	4	8	5	3	SRR2058980	B. suis bv1	6	33	14	B95-0512	Dog		USA:OH
2	3	6	10	4	1	5	2	4	38	9	9	4	8	5	3	SRR2058983	B. suis bv1	6	33	14	B13-0260	Dog		USA:OK
2	3	6	10	4	1	5	2	4	38	9	7	6	8	5	3	SRR2058968	B. suis bv1	6	33	14	B13-0195	Swine		USA:TX
2	3	6	10	4	1	5	2	4	38	9	7	6	8	5	3	SRR2058969	B. suis bv1	6	33	14	B13-0198	Swine		USA:TX
2	3	6	10	4	1	5	2	4	38	9	7	7	8	5	3	2006LeFleche#026	B. suis bv1	6	33		BCCN#87-87	unknown	1987	Poland
2	3	6	10	4	1	5	2	4	38	9	7	7	8	5	3	2018Vergnaud#0260	B. suis bv1	6	33		BCCN#04-21	Wild boar	2004	Croatia
2	3	6	10	4	1	5	2	4	38	9	2	8	7	5	3	2007Garcia_Yoldi.	B. suis bv1	6	33		S-99	Hare	1980	Croatia
2	3	6	10	4	1	5	2	4	38	9	2	7	7	5	3	2007Garcia_Yoldi.	B. suis bv1	6	33		S-100	Hare	1980	Croatia
2	3	6	10	4	1	5	2	4	38	9	2	8	8	5	3	2007Garcia_Yoldi.	B. suis bv1	6	33		S-90	Wild boar	1992	Croatia
2	3	6	10	4	1	5	2	4	38	9	2	7	8	5	3	2007Garcia_Yoldi.	B. suis bv1	6	33		S-97	Swine	1994	Croatia
2	3	6	10	4	1	5	2	5	38	9	7	7	7	5	3	GCA_000875695	B. suis	6	32	14	Human/AR/US.	Swine	1981	Arkansas, USA
2	3	6	10	4	1	5	2	5	38	9	7	7	7	5	3	SRR1786972	B. suis	6	32	14	Human/AR/US.	Swine	1981	Arkansas, USA
2	3	6	10	4	1	5	2	4	38	9	7	7	7	5	3	2007Garcia_Yoldi.	B. suis bv1	6	33		S-102	Hare	1980	Croatia
2	3	6	10	4	1	5	2	4	38	9	7	7	7	5	3	2018Vergnaud#0259	B. suis bv1	6	33		BCCN#04-20	Wild boar	2004	Croatia
2	3	6	10	4	1	5	2	4	38	9	7	4	7	5	3	SRR2058930	B. suis bv1	6	33	14	B05-1320	Swine		USA:IA
2	3	6	10	4	1	5	2	4	38	9	7	4	7	5	3	SRR2058931	B. suis bv1	6	33	14	B05-1324	Swine		USA:IA
2	3	6	10	4	1	5	2	4	38	9	7	4	7	5	3	SRR2058932	B. suis bv1	6	33	14	B05-1325	Swine		USA:IA
2	3	6	10	4	1	5	2	4	38	9	7	4	7	5	3	SRR2058933	B. suis bv1	6	33	14	B05-1326	Swine		USA:IA
2	3	6	10	4	1	5	2	4	38	9	7	4	7	5	3	SRR2058934	B. suis bv1	6	33		B05-1333	Swine		USA:IA
2	3	6	10	4	1	5	2	4	38	9	7	4	7	5	3	SRR2058935	B. suis bv1	6	33	14	B05-1335	Swine		USA:IA
2	3	6	10	4	1	5	2	4	38	9	7	4	7	5	3	SRR2058936	B. suis bv1	6	33	14	B05-1336	Swine		USA:IA
2	3	6	10	4	1	5	2	4	38	9	4	4	7	5	3	SRR2058925	B. suis bv1	6	33	14	B00-0234	Swine	2000	Ohio, USA
2	3	6	10	4	1	5	2	4	38	9	4	4	7	5	3	SRR2058954	B. suis bv1	6	33	14	B11-0452	Swine		USA:FL
2	3	6	10	4	1	5	2	4	38	9	6	7	9	5	3	2018Vergnaud#0262	B. suis bv1	6	33		BCCN#04-26	Wild boar	2004	Croatia
2	3	6	10	4	1	5	2	4	38	9	5	7	9	5	3	SRR2058946	B. suis bv1	6	33	14	B11-0193	Cattle		USA:TX
2	3	6	10	4	1	5	2	4	38	9	5	7	6	5	3	2007Garcia_Yoldi.	B. suis bv1	6	33		S-104	Hare	1980	Croatia
2	3	6	10	4	1	5	2	4	38	9	7	7	6	5	3	2018Vergnaud#0146	B. suis bv1	6	33		BCCN#02-28	Swine	2002	Tahiti, French Polynesia
2	3	6	10	4	1	5	2	4	38	9	6	7	6	5	3	2018Vergnaud#0701	B. suis bv1	6	33		BCCN#98-32	Swine	1998	Buenos Aires ,Argentina
2	3	6	10	4	1	5	2	4	38	9	6	8	6	5	3	2018Vergnaud#0147	B. suis bv1	6	33		BCCN#03-34	Human	2003	French Polynesia
2	3	6	10	4	1	5	2	4	38	9	7	7	6	6	3	SRR2058926	B. suis bv1	6	33	75	B00-0468	Cattle	2000	Hawaii, USA
2	3	6	10	4	1	5	2	4	38	9	6	6	6	5	3	2006LeFleche#027	B. suis bv1	6	33		BCCN#87-88	unknown	1987	Poland
2	3	6	10	4	1	5	2	4	38	9	6	6	6	5	3	2013Jiang#165	B. suis bv1	6	33		208	Human	1957	Inner Mongolia, China
2	3	6	10	4	1	5	2	4	38	9	6	6	7	5	3	SRR2058974	B. suis bv1	6	33	75	B93-0748	Cattle		USA:HI
2	3	6	10	4	1	5	2	4	38	9	6	6	9	5	3	SRR2058928	B. suis bv1	6	33	14	B00-0729	Swine		USA:TX
2	3	6	10	4	1	5	2	4	38	9	5	6	5	5	3	2006LeFleche#193	B. suis bv3	6	33		BCCN#03-27	Horse	2003	Croatia
2	3	6	10	4	1	5	2	4	38	9	5	6	5	5	3	2017Ferreira#001	B. suis bv1	6	33		INRA03-26	Swine	2003	France
2	3	6	10	4	1	5	2	4	38	9	5	6	5	5	3	2017Ferreira#002	B. suis bv1	6	33		AFSSA-03/308.	Caprine	2003	Croatia
2	3	6	10	4	1	5	2	4	38	9	5	6	5	5	3	2017Ferreira#003	B. suis bv1	6	33		AFSSA-04/115	Hare	2004	France
2	3	6	10	4	1	5	2	4	38	9	5	6	5	5	3	2017Ferreira#004	B. suis bv1	6	33		AFSSA-04/302.	Swine	2004	Croatia
2	3	6	10	4	1	5	2	4	38	9	6	6	5	5	3	2006LeFleche#012	B. suis bv1	6	33		REF 1330	Swine		USA
2	3	6	10	4	1	5	2	4	38	9	6	6	5	5	3	2013Jiang#163	B. suis bv1	6	33		71	Ovine	1974	Shandong, China
2	3	6	10	4	1	5	2	4	38	9	6	6	5	5	3	2013Jiang#164	B. suis bv1	6	33		72	Ovine	1975	Shandong, China
2	3	6	10	4	1	5	2	4	38	9	6	6	5	5	3	2017Ferreira#005	B. suis bv1	6	33		REF 1330 [biov.	Swine		USA
2	3	6	10	4	1	5	2	4	38	9	6	6	5	5	3	2017Ferreira#006	B. suis bv1	6	33		AFSSA-04/2987	Human	2004	France
2	3	6	10	4	1	5	2	4	38	9	6	6	5	5	3	2018Vergnaud#0005	B. suis bv1	6	33		BCCN#R12	Swine		USA
2	3	6	10	4	1	5	2	4	38	9	6	6	5	5	3	2018Vergnaud#0705	B. suis bv1	6	33		BCCN#98-37	Swine	1998	Buenos Aires ,Argentina
2	3	6	10	4	1	5	2	4	38	9	6	6	5	5	3	GCA_000007505	B. suis bv1	6	33	75	REF 1330	Swine		USA
2	3	6	10	4	1	5	2	4	38	9	6	6	5	5	3	GCA_000223195	B. suis bv1	6	33	75	REF 1330	Swine		USA
2	3	6	10	4	1	5	2	4	38	9	6	6	5	5	3	SRR2058923	B. suis bv1	6	33	75	REF 1330	Swine		USA
2	3	6	10	4	1	5	2	4	38	9	2	6	5	5	3	2007Garcia_Yoldi.	B. suis bv1	6	33		S-94	Wild boar	2003	Croatia
2	3	6	10	4	1	5	2	4	38	9	2	6	6	5	3	2007Garcia_Yoldi.	B. suis bv1	6	33		S-88	Horse	2003	Croatia
2	3	6	10	4	1	5	2	4	38	9	2	6	9	5	3	2007Garcia_Yoldi.	B. suis bv1	6	33		S-105	Hare	1980	Croatia
2	3	6	10	4	1	5	2	4	38	9	4	6	7	5	3	2006LeFleche#022	B. suis bv1	6	33		BCCN#87-68	unknown	1987	Portugal
2	3	6	10	4	1	5	2	4	38	9	5	6	7	5	3	SRR2058977	B. suis bv1	6	33	14	B94-0444	Swine		USA:TX
2	3	6	10	4	1	5	2	4	38	9	4	6	4	5	3	SRR2058950	B. suis bv1	6	33	14	B11-0281	Cattle		USA:FL
2	3	6	10	4	1	5	2	4	38	9	7	8	11	5	3	2018Vergnaud#0145	B. suis bv1	6	33		BCCN#74-378	Hare	1974	
2	3	6	10	4	1	5	2	4	38	9	7	8	11	9	3	2018Vergnaud#0254	B. suis bv1	6	33		BCCN#98-22	Swine	1998	Buenos Aires ,Argentina
2	3	6	10	4	1	5	2	4	38	9	7	7	5	5	4	2017Ferreira#008	B. suis bv1	6	33		AFSSA-01/5744	Swine	2001	French Polinesia
2	3	6	10	4	1	5	2	5	38	9	5	6	8	5	3	2006LeFleche#024	B. suis bv1	6	32		BCCN#87-72	unknown	1987	Denmark
2	3	6	10	4	1	5	2	5	38	9	5	6	4	5	3	SRR2058985	B. suis bv1	6	32	14	B13-0235	Human		Tonga: New_Zealand
2	3	6	10	4	1	5	2	5	38	9	5	7	4	5	3	SRR2058988	B. suis bv1	6	32	14	B13-0238	Human		Tonga: New_Zealand
2	3	6	10	4	1	5	2	5	38	9	6	5	4	5	3	SRR2058971	B. suis bv1	6	32	14	B13-0219	Human		USA:OR
2	3	6	10	4	1	5	2	5	38	9	6	5	4	5	3	SRR2058989	B. suis bv1	6	32	14	B13-0239	Human		Tonga: New_Zealand
2	3	6	10	4	1	5	2	5	38	9	7	5	4	5	3	SRR2058984	B. suis bv1	6	32	14	B13-0234	Human		Tonga: New_Zealand
2	3	6	10	4	1	5	2	5	38	9	6	6	6	5	3	2017Ferreira#007	B. suis bv1	6	32		VLA-92/29	Human	1992	Mexico
2	3	6	10	4	1	5	2	5	38	9	6	7	5	5	3	SRR2058990	B. suis bv1	6	32	14	B13-0240	Human		Tonga: New_Zealand
2	3	6	10	4	1	5	2	5	38	9	7	6	5	5	3	2018Vergnaud#0258	B. suis bv1	6	32		BCCN#03-17	Human	2003	Tahiti, French Polynesia
2	3	6	10	4	1	5	2	4	38	9	5	5	7	6	3	2013Jiang#161	B. suis bv1	6	33		33	Human	1965	Guangdong, China
2	3	6	10	4	1	5	2	4	38	9	5	5	7	6	3	2013Jiang#162	B. suis bv1	6	33		34	Swine	1965	Guangdong, China
2	3	6	10	4	1	5	2	4	38	9	5	5	8	6	3	2006LeFleche#021	B. suis bv1	6	33		BCCN#87-67a	unknown	1987	China
2	3	6	10	4	1	5	2	4	38	9	5	5	8	6	3	2013Jiang#153	B. suis bv1	6	33		N2	Cattle	2009	Inner Mongolia, China
2	3	6	10	4	1	5	2	4	38	9	5	5	8	6	3	2013Jiang#154	B. suis bv1	6	33		N6	Cattle	2009	Inner Mongolia, China
2	3	6	10	4	1	5	2	4	38	9	5	5	8	6	3	2013Jiang#155	B. suis bv1	6	33		N7	Cattle	2009	Inner Mongolia, China
2	3	6	10	4	1	5	2	4	38	9	5	5	8	6	3	2013Jiang#156	B. suis bv1	6	33		N8	Cattle	2009	Inner Mongolia, China
2	3	6	10	4	1	5	2	4	38	9	5	5	8	6	3	2013Jiang#157	B. suis bv1	6	33		N9	Cattle	2009	Inner Mongolia, China
2	3	6	10	4	1	5	2	4	38	9	5	5	8	6	3	2013Jiang#158	B. suis bv1	6	33		N11	Cattle	2009	Inner Mongolia, China
2	3	6	10	4	1	5	2	4	38	9	5	5	8	6	3	2013Jiang#159	B. suis bv1	6	33		S2	Cattle	2009	Inner Mongolia, China
2	3	6	10	4	1	5	2	4	38	9	5	5	8	6	3	2013Jiang#160	B. suis bv1	6	33		9	Ovine	2006	Inner Mongolia, China
2	3	6	10	4	1	5	2	4	38	9	5	5	8	6	3	2013Jiang#166	B. suis bv1	6	33		215	Human	1978	Inner Mongolia, China
2	3	6	10	4	1	5	2	4	38	9	5	5	8	6	3	2013Jiang#167	B. suis bv1	6	33		210	Deer/El.	1985	JiLin, China
2	3	6	10	4	1	5	2	4	38	9	5	5	8	6	3	2013Jiang#169	B. suis bv1	6	33		218	Ovine	1980	Tibet
2	3	6	10	4	1	5	2	4	38	9	5	5	9	6	3	2013Jiang#168	B. suis bv1	6	33		217	Human	1979	NingXia, China
2	3	6	10	4	1	5	2	4	38	9	5	5	5	6	3	SRR2058947	B. suis bv1	6	33	14	B11-0200	Cattle		USA:TX

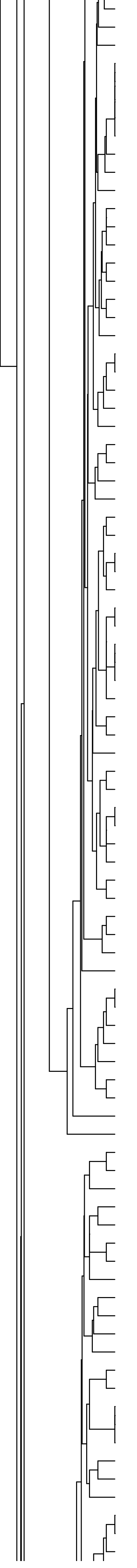

2	3	6	10	4	1	5	2	4	38	9	5	5	8	8	3	SRR2058975	B. suis bv1	6	33	75	B93-0820	Swine		USA:SC
2	3	6	10	4	1	5	2	4	38	9	5	5	5	3	3	SRR2058929	B. suis bv1	6	33	14	B00-0771	Swine		USA:AL
2	3	6	10	4	1	5	2	4	38	9	5	4	10	6	3	2017Ferreira#011	B. suis bv1	6	33		VLA-F1/04	Human	2004	Netherlands
2	3	6	10	4	1	5	2	4	38	9	5	5	6	4	3	2006LeFleche#030	B. suis bv1	6	33		BCCN#98-31	Swine	1998	Argentina
2	3	6	10	4	1	5	2	4	38	9	5	5	6	4	3	2018Vergnaud#0257	B. suis bv1	6	33		BCCN#98-25	Swine	1998	Buenos Aires ,Argentina
2	3	6	10	4	1	5	2	4	38	9	5	5	6	4	3	2018Vergnaud#0699	B. suis bv1	6	33		BCCN#98-27	Swine	1998	Buenos Aires ,Argentina
2	3	6	10	4	1	5	2	4	38	9	5	5	6	4	3	2018Vergnaud#0702	B. suis bv1	6	33		BCCN#98-33	Swine	1998	Buenos Aires ,Argentina
2	3	6	10	4	1	5	2	4	38	9	5	5	6	4	3	SRR2058955	B. suis bv1	6	33	14	B11-0460	Swine	2011	Mississippi, USA
2	3	6	10	4	1	5	2	4	38	9	5	6	6	4	3	SRR2058979	B. suis bv1	6	33	14	B95-0322	Swine		USA:AR
2	3	6	10	4	1	5	2	4	38	9	5	5	9	4	3	2018Vergnaud#0253	B. suis bv1	6	33		BCCN#96-138B	Human	1996	Argentina
2	3	6	10	4	1	5	2	4	38	9	5	6	6	6	3	SRR2058949	B. suis bv1	6	33	14	B11-0234	Swine		USA:FL
2	3	6	10	4	1	5	2	4	38	9	5	5	11	5	3	GCA_001715425	B. suis	6	33		2004000578	Human	2003	Massachusetts, USA
2	3	6	10	4	1	5	2	4	38	9	5	5	6	5	3	SRR2058970	B. suis bv1	6	33	14	B13-0216	Dog		USA:TX
2	3	6	10	4	1	5	2	4	38	9	5	5	8	5	3	SRR2058951	B. suis bv1	6	33	14	B11-0438	Cattle		USA:FL
2	3	6	10	4	1	5	2	4	38	9	4	5	5	5	3	2006LeFleche#078	B. suis	6	33		BCCN#96-36	Swine	1996	Argentina
2	3	6	10	4	1	5	2	4	38	9	5	5	5	5	3	GCA_000600055	B. suis bv1	6	33	14	S2	Swine	1960	China
2	3	6	10	4	1	5	2	4	38	9	9	5	7	5	3	2006LeFleche#025	B. suis bv1	6	33		BCCN#87-85	unknown	1987	Denmark
2	3	6	10	4	1	5	2	4	38	9	5	5	7	5	3	SRR2058972	B. suis bv1	6	33	14	B93-0078	Cattle		USA:FL
2	3	6	10	4	1	5	2	4	38	9	6	5	8	5	3	SRR2058982	B. suis bv1	6	33	14	B13-0257	Swine		USA:TX
2	3	6	10	4	1	5	2	4	38	9	5	7	7	4	3	2018Vergnaud#0860	B. suis bv1	6	33		BCCN#98-29	Swine	1998	Buenos Aires ,Argentina
2	3	6	10	4	1	5	2	4	38	9	5	7	7	4	3	2018Vergnaud#0861	B. suis bv1	6	33		BCCN#98-30	Swine	1998	Buenos Aires ,Argentina
2	3	6	10	4	1	5	2	4	38	9	5	5	7	4	3	2017Ferreira#010	B. suis bv1	6	33		VLA-64/24	Swine	1964	USA
2	3	6	10	4	1	5	2	4	38	9	5	7	7	6	3	2018Vergnaud#0197	B. abortus bv3	6	33		BCCN#78-143	Cattle	1978	Batata Demba ,Velingar.
2	3	6	10	4	1	5	2	4	38	9	5	8	7	7	3	SRR2058961	B. suis bv1	6	33	14	B11-0566	Cattle		USA:TX
2	3	6	10	4	1	5	2	4	38	9	6	5	7	4	3	2006LeFleche#023	B. suis bv1	6	33		BCCN#87-71	unknown	1987	USA
2	3	6	10	4	1	5	2	4	38	9	6	5	7	3	3	2006LeFleche#028	B. suis bv1	6	33		BCCN#87-89	Swine	1987	USA
2	3	6	10	4	1	5	2	5	38	9	6	5	7	6	3	SRR2058986	B. suis bv1	6	32	14	B13-0236	Human		Tonga: New_Zealand
2	3	6	10	4	1	5	2	4	38	9	6	8	7	9	3	2018Vergnaud#0255	B. suis bv1	6	33		BCCN#98-23	Swine	1998	Buenos Aires ,Argentina
2	3	6	10	4	1	5	2	4	38	9	4	6	7	4	3	SRR2058943	B. suis bv1	6	33	14	B11-0093	Swine		USA:TX
2	3	6	10	4	1	5	2	4	38	9	4	6	8	4	3	SRR2058978	B. suis bv1	6	33		B94-0564	Horse		USA:OK
2	3	6	10	4	1	5	2	4	38	9	4	6	6	4	3	2006LeFleche#029	B. suis bv1	6	33		BCCN#98-26	Swine	1998	Argentina
2	3	6	10	4	1	5	2	4	38	9	4	6	6	4	3	SRR2058945	B. suis bv1	6	33		B11-0172	Swine		USA:TX
2	3	6	10	4	1	5	2	4	38	9	4	7	6	4	3	2018Vergnaud#0700	B. suis bv1	6	33		BCCN#98-28	Swine	1998	Buenos Aires ,Argentina
2	3	6	10	4	1	5	2	4	38	9	4	5	8	4	3	2018Vergnaud#0704	B. suis bv1	6	33		BCCN#98-35	Swine	1998	Buenos Aires ,Argentina
2	3	6	10	4	1	5	2	4	38	9	4	5	8	4	3	2018Vergnaud#0707	B. suis bv1	6	33		BCCN#98-44	Swine	1998	Buenos Aires ,Argentina
2	3	6	10	4	1	5	2	4	38	9	4	5	9	4	3	2018Vergnaud#0256	B. suis bv1	6	33		BCCN#98-24	Swine	1998	Buenos Aires ,Argentina
2	3	6	10	4	1	5	2	4	38	9	4	5	9	4	3	2018Vergnaud#0706	B. suis bv1	6	33		BCCN#98-39	Swine	1998	Buenos Aires ,Argentina
2	3	6	10	4	1	5	2	4	38	9	4	5	9	4	3	2018Vergnaud#1104	B. suis bv1	6	33		BCCN#98-36	Swine	1998	Buenos Aires, Argentina
2	3	6	10	4	1	5	2	4	38	9	4	5	3	4	3	SRR2058952	B. suis bv1	6	33	14	B11-0440	Cattle		USA:FL
2	3	6	10	4	1	5	2	4	38	9	5	4	8	4	3	SRR2058953	B. suis bv1	6	33	14	B11-0443	Cattle		USA:TX
2	3	6	10	4	1	5	2	4	38	9	4	4	8	4	3	SRR2058966	B. suis bv1	6	33	14	B12-0604	Cattle		USA:TX
2	3	6	10	4	1	5	2	4	38	9	4	5	10	8	3	SRR2058927	B. suis bv1	6	33	14	B00-0489	Cattle	2000	Florida, USA
2	3	6	10	4	1	5	2	4	38	9	4	4	6	6	3	SRR2058962	B. suis bv1	6	33	14	B11-0586	Swine		USA:FL
2	3	6	10	4	1	5	2	4	38	9	4	4	9	6	3	SRR2058967	B. suis bv1	6	33	14	B12-0608	Cattle		USA:FL
2	3	6	10	4	1	5	2	4	38	9	4	4	8	5	3	2006LeFleche#031	B. suis bv1	6	33		BCCN#98-43	Swine	1998	Argentina
2	3	6	10	4	1	5	2	4	38	9	4	4	8	5	3	2018Vergnaud#0863	B. suis bv1	6	33		BCCN#98-42	Swine	1998	Buenos Aires ,Argentina
2	3	6	10	4	1	5	2	4	38	9	4	4	6	5	3	SRR2058924	B. suis bv1	6	33	14	B00-0133	Swine	2000	Ohio, USA
2	3	6	10	4	1	5	2	4	38	9	4	4	9	5	3	SRR2058944	B. suis bv1	6	33	14	B11-0171	Cattle		USA:FL
2	3	6	10	4	1	5	2	4	38	9	4	4	6	7	3	SRR2058948	B. suis bv1	6	33	14	B11-0203	Human		USA:WI
2	3	6	10	4	1	5	2	4	38	9	4	6	6	7	3	SRR2058976	B. suis bv1	6	33	14	B94-0322	Swine		USA:LA
2	3	6	10	4	1	5	2	3	38	9	4	5	7	5	3	2018Vergnaud#0703	B. suis bv1	6	258		BCCN#98-34		1998	Buenos Aires ,Argentina
2	3	6	10	4	1	5	2	3	38	9	4	5	6	5	3	2018Vergnaud#0862	B. suis bv1	6	258		BCCN#98-40	Swine	1998	Buenos Aires ,Argentina
2	3	6	10	4	1	5	2	6	38	9	4	5	6	5	3	SRR2058987	B. suis bv1	6	353	14	B13-0237	Human		Tonga: New_Zealand
2	3	6	10	4	1	5	2	4	38	9	6	9	12	8	3	2018Vergnaud#0261	B. suis bv1	6	33		BCCN#04-25	Wild boar	2004	Croatia
2	3	6	9	4	1	5	2	4	38	9	4	5	7	6	3	GCA_000236255	B. suis	169	354	14	VBI22	Cattle	2011	Texas, USA
2	3	6	9	4	1	5	2	4	38	9	4	5	7	6	3	SRR2058960	B. suis bv1	169	354	14	B11-0552	Cattle	2011	Texas, USA
2	3	6	9	4	1	5	2	4	38	9	4	5	8	6	3	SRR2058963	B. suis bv1	169	354	14	B11-0603	Cattle	2011	Texas, USA
2	3	6	9	4	1	5	2	4	38	9	4	6	7	6	3	SRR2058964	B. suis bv1	169	354	14	B12-0577	Cattle	2012	Texas, USA
2	3	6	9	4	1	5	2	4	38	9	4	4	5	6	3	SRR2058958	B. suis bv1	169	354	14	B11-0527	Cattle	2011	Texas, USA
2	3	6	9	4	1	5	2	4	38	9	4	5	6	5	3	SRR2058965	B. suis bv1	169	354	14	B12-0598	Cattle	2012	Texas, USA
2	3	6	9	4	1	5	2	4	38	9	4	5	5	5	3	SRR2058973	B. suis bv1	169	354	14	B93-0364	Dog	1993	Georgia, USA
2	4	6	10	4	1	5	2	6	38	9	7	7	6	5	3	2017Ferreira#012	B. suis bv1	153	255		AFSSA-96/164.	Swine	1996	France
2	3	6	10	4	1	5	2	6	43	9	5	6	5	5	4	2017Ferreira#009	B. suis bv1	6	256		AFSSA-03/206.	Swine	2003	France
3	5	2	10	1	1	5	2	3	7	9	11	10	10	8	2	2006LeFleche#194	B. ovis	1	25		BCCN#74-308	Ovine	1974	Pau, France
3	5	2	10	1	1	5	2	3	7	9	11	10	12	8	2	2006LeFleche#195	B. ovis	1	25		BCCN#74-345	Ovine	1974	Pau, France
3	5	2	10	1	1	5	2	3	7	9	8	7	14	8	2	2006LeFleche#196	B. ovis	1	25		BCCN#76-247	Ovine	1976	Avignon, France
3	5	2	10	1	1	5	2	3	7	9	9	9	9	14	2	2006LeFleche#121	B. ovis	1	25		BCCN#R22	Ovine		USA
3	5	2	10	1	1	5	2	3	7	9	9	9	8	7	2	2006LeFleche#198	B. ovis	1	25		BCCN#76-258	Ovine	1976	Pau, France
3	5	2	10	1	1	5	2	3	7	9	9	5	6	8	2	2006LeFleche#178	B. ovis	1	25		BfR 176	Ovine	1998	
3	5	2	10	1	1	5	2	3	7	9	9	5	5	8	2	2006LeFleche#181	B. ovis	1	25		BfR 181	Ovine	1998	
3	5	2	10	1	1	5	2	3	7	9	9	4	7	15	2	2006LeFleche#197	B. ovis	1	25		BCCN#76-250	Ovine	1976	Pau, France
3	5	2	10	1	1	5	2	3	7	9	10	5	12	9	2	2006LeFleche#176	B. ovis	1	25		BfR 173	Ovine	1998	
3	5	2	10	1	1	5	2	3	7	9	9	6	12	9	2	2006LeFleche#179	B. ovis	1	25		BfR 179	Ovine	1998	
3	5	2	10	1	1	5	2	3	7	9	8	6	13	9	2	2006LeFleche#199	B. ovis	1	25		BCCN#77-7	Ovine	1977	Nice, France
3	5	2	10	1	1	5	2	3	7	9	10	7	15	9	2	2006LeFleche#201	B. ovis	1	25		BCCN#82-90	Ovine	1982	Pau, France
3	5	2	10	1	1	5	2	3	7	9	6	5	16	10	2	2006LeFleche#177	B. ovis	1	25		BfR 175	Ovine	1998	
3	5	2	10	1	1	5	2	3	7	9	6	5	17	10	2	2006LeFleche#180	B. ovis	1	25		BfR 180	Ovine	1998	
3	5	2	10	1	1	5	2	3	7	9	6	7	7	6	2	2006LeFleche#011	B. ovis	1	25		REF BOW 63/.	Ovine		Australia
3	5	2	10	1	1	5	2	3	7	9	6	7	7	6	2	2018Vergnaud#0003	B. ovis	1	25		BCCN#R17	Ovine		Africa
3	5	2	10	1	1	5	2	3	7	9	6	7	7	6	2	GCA_000016845	B. ovis	1	25	13	REF BOW 63/.	Ovine	2007	Australia
3	5	2	10	1	1	5	2	3	7	9	6	12	12	14	2	2006LeFleche#123	B. ovis	1	25		BCCN#98-46	Ovine	1998	Argentina
3	5	2	10	1	1	5	2	3	7	9	6	6	12	12	2	2014Dorneles#006	B. ovis	1	25		Bo241E	Ovine	1982	Porto Alegre, 90570020.
3	5	2	10	1	1	5	2	3	7	9	6	12	11	12	7	2018Vergnaud#0062	B. ovis	1	25		BCCN#91-143	Ovine	1991	Salonique ,Greece
3	5	2	10	1	1	5	2	3	7	9	5	4	9	8	2	2014Dorneles#005	B. ovis	1	25		Bo203L	Ovine	1995	Santana do Livramento,.
3	5	2	10	1	1	5	2	3	7	9	5	4	9	8	2	2014Dorneles#007	B. ovis	1	25		Bo252L	Ovine	1995	Santana do Livramento,.
3	5	2	10	1	1	5	2	3	7	9	5	4	9	9	2	2014Dorneles#004	B. ovis	1	25		Bo201L	Ovine	1995	Santana do Livramento,.

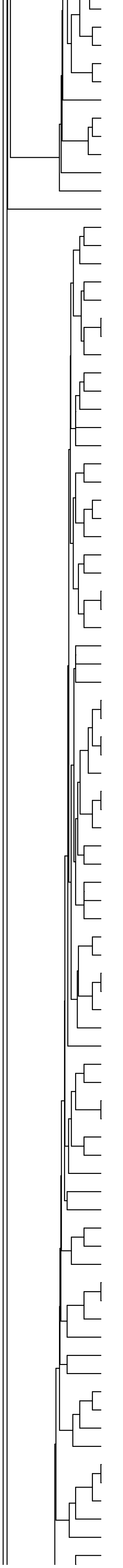

3	5	2	10	1	1	5	2	3	7	9	6	4	9	8	2	2014Dorneles#014	B. ovis	1	25	BoS/NL	Ovine	1995	Santana do Livramento,.
3	4	2	10	1	1	5	2	2	7	9	6	4	9	8	2	2014Dorneles#008	B. ovis	164	252	Bo266L	Ovine	1995	Santana do Livramento,.
3	4	2	10	1	1	5	2	3	7	9	6	4	9	8	2	2014Dorneles#009	B. ovis	164	251	Bo286L	Ovine	1995	Santana do Livramento,.
3	5	2	10	1	1	5	2	3	7	9	0	4	13	13	2	2014Dorneles#002	B. ovis	1	25	Bo0236	Ovine	1995	Uruguaina, 97501974, .
3	5	2	10	1	1	5	2	3	7	9	8	4	13	13	2	2014Dorneles#003	B. ovis	1	25	Bo100V	Ovine	1995	Santana do Livramento,.
3	4	2	10	1	1	5	2	3	7	9	6	8	3	10	2	2014Dorneles#011	B. ovis	164	251	Bo5013	Ovine	1985	Porto Alegre, 90570020.
3	4	2	9	1	1	5	2	3	7	9	7	4	14	14	2	2014Dorneles#010	B. ovis	165	250	Bo31V	Ovine	1995	Santana do Livramento,.
3	4	2	10	1	1	5	2	3	7	9	7	4	14	14	2	2014Dorneles#012	B. ovis	164	251	Bo91AV	Ovine	1995	Santana do Livramento,.
3	5	2	10	1	1	5	2	3	7	9	7	4	14	14	2	2014Dorneles#013	B. ovis	1	25	Bo94AV	Ovine	1995	Santana do Livramento,.
3	5	2	10	1	1	5	2	3	7	7	7	6	10	8	2	2006LeFleche#200	B. ovis	1	24	BCCN#78-256	Ovine	1978	Rennes, France
2	5	2	10	1	1	5	2	3	7	9	4	6	9	5	2	2006LeFleche#202	B. ovis	2	23	BCCN#91-68	Ovine	1991	Spain
2	5	3	12	3	1	3	2	5	36	9	9	6	7	10	4	2006LeFleche#084	B. dog Costa Rica	46	97	BCCN#84-3	Dog	1984	Costa Rica
2	4	8	14	6	1	5	6	6	43	9	9	10	11	2	9	2006LeFleche#071	B. suis bv2	7	44	BCCN#02-50	Swine	2002	Le Mans, France
2	4	8	14	6	1	5	6	6	43	9	9	5	9	2	9	2017Ferreira#045	B. suis bv2	7	44	C9B4	Wild boar		Belgium
2	4	8	14	6	1	5	6	6	43	9	5	4	11	2	9	2018Vergnaud#0164	B. suis bv2	7	44	BCCN#01-14	Swine	2001	Charente, France
2	4	8	14	6	1	5	6	6	43	9	7	5	15	2	9	2017Ferreira#044	B. suis bv2	7	44	S275	Wild boar		Belgium
2	4	8	14	6	1	5	6	6	43	9	16	5	16	2	9	2018Vergnaud#0163	B. suis bv2	7	44	BCCN#96-123	Swine	1996	Cote d'Or, France
2	4	8	14	6	1	5	6	6	43	9	7	5	16	2	8	2018Vergnaud#0252	B. suis Rough	7	44	BCCN#03-31	Swine	2003	Haute-Vienne,France
2	4	8	14	6	1	5	6	6	43	9	7	5	16	2	8	2018Vergnaud#0717	B. suis bv2	7	44	BCCN#03-32	Swine	2003	Haute-Vienne, France
2	4	8	14	6	1	5	6	6	43	9	15	5	17	2	8	2018Vergnaud#0301	B. suis bv2	7	44	BCCN#03-16	Hare	2003	Haute-Vienne, France
2	4	8	14	6	1	5	6	4	43	9	9	7	9	2	8	2017Ferreira#055	B. suis bv2	7	41	AFSSA-03/148.	Wild boar	2003	France
2	4	8	14	6	1	5	6	6	43	9	15	7	9	2	8	2018Vergnaud#0320	B. suis bv2	7	44	BCCN#04-9	Wild boar	2004	Ille-et-Vilaine, France
2	4	8	14	6	1	5	6	6	43	9	13	7	17	2	8	2018Vergnaud#0177	B. suis bv2	7	44	BCCN#06-73	Swine	2006	France
2	4	8	14	6	1	5	6	6	43	9	9	7	16	2	7	2017Ferreira#064	B. suis bv2	7	44	RATES5-11	Wild boar		Belgium
2	4	8	14	6	1	5	6	6	43	9	9	6	12	2	8	2018Vergnaud#0302	B. suis bv2	7	44	BCCN#03-21	Wild boar	2003	Lot, France
2	4	8	14	6	1	5	6	6	43	9	15	6	7	2	5	2017Ferreira#052	B. suis bv2	7	44	AFSSA-97/9757	Swine	1997	France
2	4	8	14	6	1	5	6	6	43	9	15	11	19	2	5	2018Vergnaud#0718	B. suis bv2	7	44	BCCN#03-4	Wild boar	2003	Indre-et-Loire, France
2	4	8	14	6	1	5	6	6	43	9	15	9	17	2	9	2017Ferreira#037	B. suis bv2	7	44	C2B11	Wild boar		Belgium
2	4	8	14	6	1	5	6	6	43	9	15	9	18	2	9	2017Ferreira#038	B. suis bv2	7	44	C9B3	Wild boar		Belgium
2	4	8	14	6	1	5	6	6	43	9	15	9	13	2	7	2006LeFleche#045	B. suis bv2	7	44	BCCN#93-63	Swine	1993	Rouen, France
2	4	8	14	6	1	5	6	6	43	9	20	7	12	2	9	2017Ferreira#035	B. suis bv2	7	44	AFSSA-04/191.	Wild boar	2004	Switzerland
2	4	8	14	6	1	5	6	6	43	9	22	7	17	2	9	2017Ferreira#036	B. suis bv2	7	44	C13B4	Wild boar		Belgium
2	4	8	14	6	1	5	6	4	43	9	15	7	7	2	9	2017Ferreira#040	B. suis bv2	7	41	0111602/4+9	Wild boar		Belgium
2	4	8	14	6	1	5	6	4	43	9	15	7	7	2	9	2017Ferreira#041	B. suis bv2	7	41	0111602/3+8	Wild boar		Belgium
2	4	8	14	6	1	5	6	6	43	9	15	7	15	2	9	2017Ferreira#039	B. suis bv2	7	44	PY69	Wild boar		Belgium
2	4	8	14	6	1	5	6	6	43	9	4	6	24	2	6	2006LeFleche#052	B. suis bv2	7	44	BCCN#00-23	Swine	2000	Limoges, France
2	4	8	14	6	1	5	6	6	43	9	11	6	17	2	12	2017Ferreira#053	B. suis bv2	7	44	MASA07	Wild boar		Belgium
2	4	8	14	6	1	5	6	6	43	9	14	6	14	2	7	2006LeFleche#163	B. suis bv2	7	44	BfR 157	Wild boar	2001	Bern, Switzerland
2	4	8	14	6	1	5	6	6	43	9	6	6	16	2	10	2017Ferreira#049	B. suis bv2	7	44	AFSSA-04/770	Wild boar	2004	Italy
2	4	8	14	6	1	5	6	6	43	9	6	6	16	2	10	2018Vergnaud#0305	B. suis bv2	7	44	BCCN#04-16	Wild boar	2004	Italy
2	4	8	14	6	1	5	6	6	43	9	7	6	16	2	10	2017Ferreira#047	B. suis bv2	7	44	It2	Wild boar		Italy
2	4	8	14	6	1	5	6	6	43	9	7	6	16	2	10	2017Ferreira#048	B. suis bv2	7	44	It3	Wild boar		Italy
2	4	8	14	6	1	5	6	6	43	9	6	9	16	2	10	2006LeFleche#050	B. suis bv2	7	44	BCCN#98-95	Swine	1998	Limoges, France
2	4	8	14	6	1	5	6	6	43	9	7	6	18	2	9	2017Ferreira#042	B. suis bv2	7	44	It4	Wild boar		Italy
2	4	8	14	6	1	5	6	6	43	9	7	6	18	2	9	2017Ferreira#043	B. suis bv2	7	44	It5	Wild boar		Italy
2	4	8	14	6	1	5	6	6	43	9	7	6	18	2	10	2018Vergnaud#0356	B. suis bv2	7	44	BCCN#04-19	Wild boar	2004	Italy
2	4	8	14	6	1	5	6	6	43	9	8	6	19	2	10	2017Ferreira#050	B. suis bv2	7	44	It6	Wild boar		Italy
2	4	8	14	6	1	5	6	6	43	9	8	6	10	2	11	2017Ferreira#051	B. suis bv2	7	44	It1	Wild boar		Italy
2	4	8	14	6	1	5	6	6	43	9	11	12	7	2	10	2006LeFleche#068	B. suis bv2	7	44	BCCN#02-36	Swine	2002	Le Mans, France
2	4	8	14	6	1	5	6	6	43	9	9	8	7	2	10	2006LeFleche#076	B. suis bv2	7	44	BCCN#03-11	Boar	2003	Rennes, France
2	4	8	14	6	1	5	6	6	43	9	10	6	7	2	10	2018Vergnaud#0170	B. suis bv2	7	44	BCCN#06-58	Swine	2006	France
2	4	8	14	6	1	5	6	6	43	9	12	4	14	2	9	2006LeFleche#075	B. suis bv2	7	44	BCCN#03-6	Boar	2003	Chambery, France
2	4	8	14	6	1	5	6	6	43	9	12	4	14	2	5	2018Vergnaud#0715	B. suis bv2	7	44	BCCN#03-10	Wild boar	2003	Savoie, France
2	4	8	14	6	1	5	6	6	43	9	12	6	8	2	5	2018Vergnaud#0720	B. suis bv2	7	44	BCCN#03-8	Wild boar	2003	Savoie, France
2	4	8	14	6	1	5	6	6	43	9	12	6	8	2	5	2018Vergnaud#0721	B. suis bv2	7	44	BCCN#03-9	Wild boar	2003	Savoie, France
2	4	8	14	6	1	5	6	6	43	9	12	6	8	2	8	2006LeFleche#067	B. suis bv2	7	44	BCCN#02-23	Boar	2002	Chambery, France
2	4	8	14	6	1	5	6	6	43	9	12	9	15	2	8	2017Ferreira#046	B. suis bv2	7	44	C8B3	Wild boar		Belgium
2	4	8	14	6	1	5	6	6	43	9	11	10	22	2	7	2017Ferreira#065	B. suis bv2	7	44	C6B1	Wild boar		Belgium
2	4	8	14	6	1	5	6	5	43	9	13	8	17	2	10	2017Ferreira#056	B. suis bv2	7	246	C13B1	Wild boar		Belgium
2	4	8	14	6	1	5	6	5	43	9	15	8	7	2	10	2017Ferreira#057	B. suis bv2	7	246	C3B3	Wild boar		Belgium
2	4	8	14	6	1	5	6	5	43	9	14	8	16	2	9	2017Ferreira#058	B. suis bv2	7	246	C13B6	Wild boar		Belgium
2	4	8	14	6	1	5	6	5	43	9	14	8	16	2	9	2017Ferreira#059	B. suis bv2	7	246	C11B4	Wild boar		Belgium
2	4	8	14	6	1	5	6	6	43	9	14	8	11	2	10	2017Ferreira#063	B. suis bv2	7	44	COSA13	Wild boar		Belgium
2	4	8	14	6	1	5	6	6	43	9	10	8	11	2	5	2018Vergnaud#0716	B. suis bv2	7	44	BCCN#03-29	Swine	2003	Haute-Vienne, France
2	4	8	14	6	1	5	6	5	43	9	10	5	19	2	9	2017Ferreira#060	B. suis bv2	7	246	C4B6	Wild boar		Belgium
2	4	8	14	6	1	5	6	6	43	9	13	13	6	2	11	2006LeFleche#065	B. suis bv2	7	44	BCCN#02-20	Swine	2002	Niort, France
2	4	8	14	6	1	5	6	7	43	9	13	7	20	2	10	2006LeFleche#126	B. suis bv2	7	42	BfR 9	Wild boar	2002	Halle, Germany
2	5	8	14	6	1	5	6	6	43	9	16	7	18	2	11	2017Ferreira#032	B. suis bv2	157	244	Bs364CITA	Wild boar	2008	Añón,Spain
2	5	8	14	6	1	5	6	6	43	9	14	7	18	2	11	2017Ferreira#033	B. suis bv2	157	244	Bs365CITA	Wild boar	2008	Añón,Spain
2	5	8	14	6	1	5	6	6	43	9	23	9	7	2	10	2017Ferreira#034	B. suis bv2	157	244	Bs396CITA	Wild boar	2009	Navarra,Spain
2	4	8	14	6	1	5	6	5	43	9	15	7	10	2	11	2017Ferreira#061	B. suis bv2	7	246	S120	Wild boar		Belgium
2	4	8	14	6	1	5	6	5	43	9	15	7	10	2	11	2018Vergnaud#0291	B. suis bv2	7	246	BCCN#94-2	Wild boar	1994	Belgium
2	4	8	14	6	1	5	6	5	43	9	9	7	7	2	11	2017Ferreira#062	B. suis bv2	7	246	C5B5	Wild boar		Belgium
2	4	8	9	6	1	5	6	5	43	9	8	7	9	2	7	2018Vergnaud#0719	B. suis bv2	122	243	BCCN#03-7	Wild boar	2003	Savoie, France
2	4	8	14	7	1	5	6	6	43	9	6	4	18	2	5	2006LeFleche#074	B. suis bv2	12	43	BCCN#03-5	Swine	2003	Moulins, France
2	4	8	14	6	1	5	6	8	43	9	6	8	18	2	12	2018Vergnaud#0171	B. suis bv2	7	247	BCCN#06-61	Swine	2006	France
2	4	8	14	6	1	5	6	4	43	9	14	4	11	2	7	2018Vergnaud#0167	B. suis bv2	7	41	BCCN#02-24	Wild boar	2002	Creuse, France
2	4	8	14	6	1	5	6	4	43	9	14	4	8	2	7	2018Vergnaud#0299	B. suis bv2	7	41	BCCN#01-56	Wild boar	2001	Haute-Vienne, France
2	4	8	14	6	1	5	6	4	43	9	8	4	8	2	8	2006LeFleche#053	B. suis bv2	7	41	BCCN#00-24	Boar	2000	Limoges, France
2	4	8	14	4	1	5	6	4	43	9	15	4	7	2	7	2006LeFleche#066	B. suis bv2	11	40	BCCN#02-21	Swine	2002	Gueret, France
2	4	8	14	6	1	5	7	6	43	9	4	7	3	2	7	2006LeFleche#051	B. suis bv2	8	45	BCCN#00-8	Cattle	2000	Clermont-Ferrand, Fra.
2	4	8	14	6	1	5	7	6	43	9	4	7	3	2	7	2017Ferreira#066	B. suis bv2	8	45	AFSSA-00/4898	Caprine	2000	France
2	4	8	14	6	1	5	7	6	43	9	4	8	3	2	7	2018Vergnaud#0319	B. suis bv2	8	45	BCCN#04-30	Human	2004	Gard, France
2	4	8	14	6	1	5	7	6	43	9	14	4	10	2	7	2006LeFleche#033	B. suis bv2	8	45	BCCN#81-89	Hare	1981	Poland
2	4	8	14	5	1	5	7	7	43	9	18	7	3	2	7	2006LeFleche#038	B. suis bv2	17	37	BCCN#84-22	Hare	1984	Gap, France
2	4	8	14	6	1	5	2	5	43	9	5	7	20	2	4	2018Vergnaud#0161	B. suis bv2	9	47	BCCN#94-9	Hare	1994	Haut-Rhin, France

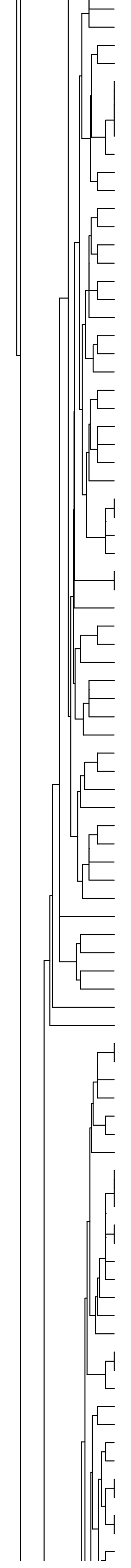

2	4	8	14	6	1	5	2	5	43	9	8	12	18	2	4	2018Vergnaud#0304	B. suis bv2	9	47		BCCN#04-15	Hare	2004	Bas-Rhin, France
2	4	8	14	6	1	5	2	2	43	9	8	7	14	2	4	2018Vergnaud#0162	B. suis bv2	9	248		BCCN#96-122	Wild boar	1996	Moselle, France
2	4	8	14	6	1	5	2	6	43	9	20	9	10	2	4	2017Ferreira#030	B. suis bv2	9	50		VLA-74/11	Unknown	1974	Denmark
2	4	8	14	6	1	5	2	6	43	9	19	8	10	2	4	2018Vergnaud#0176	B. suis bv2	9	50		BCCN#06-69		2006	United Kingdom
2	4	8	14	6	1	5	2	6	43	9	9	9	18	2	4	2014Kreizinger#069	B. suis bv2	9	50		REF Thomsen .	Swine	1952	Denmark
2	4	8	14	6	1	5	2	6	43	9	9	9	18	2	4	2017Ferreira#028	B. suis bv2	9	50		REF Thomsen [.	Swine	1952	Denmark
2	4	8	14	6	1	5	2	6	43	9	9	9	18	2	4	2018Vergnaud#0174	B. suis bv2	9	50		BCCN#06-66		2006	United Kingdom
2	4	8	14	6	1	5	2	6	43	9	9	9	18	2	4	GCA_000018905	B. suis bv2	9	50	15	REF Thomsen .	Swine	2007	Denmark
2	4	8	14	6	1	5	2	6	45	9	9	9	18	2	4	2006LeFleche#013	B. suis bv2	9	51		REF Thomsen .	Swine		Denmark
2	4	8	14	6	1	5	2	6	43	9	23	12	13	2	4	2006LeFleche#156	B. suis bv2	9	50		BfR 150	Swine	1998	Aulendorf, Germany
2	4	8	14	6	1	5	2	6	43	9	9	12	7	2	4	2018Vergnaud#0009	B. suis bv2	9	50		BCCN#R13	Swine		Denmark
2	4	8	14	6	1	5	2	6	43	9	15	5	12	2	5	2018Vergnaud#0155	B. suis bv2	9	50		BCCN#74-379	Hare	1974	
2	4	8	14	6	1	5	2	6	43	9	5	5	11	2	5	2018Vergnaud#0156	B. suis bv2	9	50		BCCN#74-380	Hare	1974	
2	4	8	14	6	1	5	2	6	43	9	13	9	17	2	5	2006LeFleche#034	B. suis bv2	9	50		BCCN#81-287	Swine	1981	Strasbourg, France
2	4	8	14	6	1	5	2	6	43	9	28	9	11	2	5	2006LeFleche#148	B. suis bv2	9	50		BfR 138	Hare	1999	Nurnberg, Germany
2	4	8	14	6	1	5	2	6	43	9	16	7	11	2	6	2006LeFleche#042	B. suis bv2	9	50		BCCN#89-155	Hare	1989	Metz, France
2	4	8	14	6	1	5	2	6	43	9	20	7	11	2	5	2018Vergnaud#0165	B. suis bv2	9	50		BCCN#02-19	Hare	2002	Maine-et-Loire, France
2	4	8	14	6	1	5	2	6	43	9	20	13	9	2	5	2006LeFleche#151	B. suis bv2	9	50		BfR 142	Hare	1998	Bern, Switzerland
2	4	8	14	6	1	5	2	5	43	9	15	12	5	2	5	2006LeFleche#040	B. suis bv2	9	47		BCCN#87-17	Hare	1987	Avignon, France
2	4	8	14	6	1	5	2	6	43	9	14	12	5	2	5	2017Ferreira#029	B. suis bv2	9	50		AFSSA-92/13000	Hare	1992	France
2	4	8	14	6	1	5	2	6	43	9	12	10	5	2	5	2018Vergnaud#0157	B. suis bv2	9	50		BCCN#93-22	Hare	1993	Vaucluse, France
2	4	8	14	6	1	5	2	6	43	9	13	5	9	2	4	2017Ferreira#031	B. suis bv2	9	50		AFSSA-00/9182	Hare	2000	France
2	4	8	14	6	1	5	2	6	43	9	11	5	3	2	4	2018Vergnaud#0712	B. suis bv2	9	50		BCCN#93-21	Hare	1993	Haut-Rhin, France
2	4	8	14	6	1	5	2	6	43	9	7	5	16	2	6	2006LeFleche#064	B. suis bv2	9	50		BCCN#02-18	Hare	2002	Angers, France
2	4	8	14	6	1	5	2	6	43	9	9	5	8	2	6	2006LeFleche#070	B. suis bv2	9	50		BCCN#02-48	Hare	2002	Angers, France
2	4	8	14	6	1	5	2	6	43	9	13	5	15	2	6	2018Vergnaud#0300	B. suis bv2	9	50		BCCN#02-49	Hare	2002	Maine-et-Loire, France
2	4	8	14	6	1	5	2	6	43	9	12	5	13	2	11	2006LeFleche#032	B. suis bv2	9	50		BCCN#78-6	Hare	1978	Metz, France
2	4	8	14	6	1	5	2	6	43	9	11	4	12	2	6	2014Kreizinger#003	B. suis bv2	9	50		BH14	Hare	2008	3051,Hungary
2	4	8	14	6	1	5	2	6	43	9	11	4	12	2	6	2014Kreizinger#005	B. suis bv2	9	50		BH16	Hare	2010	6445,Hungary
2	4	8	14	6	1	5	2	6	43	9	11	4	13	2	6	2014Kreizinger#001	B. suis bv2	9	50		BH12	Hare	2008	5126,Hungary
2	4	8	14	6	1	5	2	6	43	9	11	4	11	2	6	2014Kreizinger#002	B. suis bv2	9	50		BH13	Hare	2008	5123,Hungary
2	4	8	14	6	1	5	2	6	39	9	14	8	10	2	5	2006LeFleche#043	B. suis bv2	9	49		BCCN#93-20	Hare	1993	Colmar, France
2	4	8	14	6	1	5	2	6	39	9	14	8	10	2	5	2018Vergnaud#0711	B. suis bv2	9	49		BCCN#93-19	Hare	1993	Haut-Rhin, France
2	4	8	9	6	1	5	2	6	43	9	10	8	13	2	6	2006LeFleche#155	B. suis bv2	14	46		BfR 148	Hare		Bern, Switzerland
2	4	8	14	6	1	5	2	5	43	9	12	4	14	2	7	2014Kreizinger#004	B. suis bv2	9	47		BH15	Hare	2010	6445,Hungary
2	4	8	14	6	1	5	2	5	43	9	9	4	17	2	7	GCA_000698325	B. suis bv2	9	47	40	Bs364CITA			
2	4	8	14	6	1	5	2	5	43	9	16	11	11	2	8	2006LeFleche#044	B. suis bv2	9	47		BCCN#93-24	Hare	1993	Moulins, France
2	4	8	14	6	1	5	2	7	43	9	14	7	13	2	8	2006LeFleche#035	B. suis bv2	9	48		BCCN#81-288	Hare	1981	Metz, France
2	4	8	14	6	1	5	2	7	43	9	4	7	3	2	4	2017Ferreira#068	B. suis bv2	9	48		AFSSA-98/6335	Swine	1998	France
2	4	8	14	6	1	5	2	7	43	9	6	7	17	2	7	2018Vergnaud#0175	B. suis bv2	9	48		BCCN#06-67		2006	United Kingdom
2	4	8	14	6	1	5	2	7	43	9	6	6	23	2	12	2006LeFleche#039	B. suis bv2	9	48		BCCN#87-11	Hare	1987	Metz, France
2	4	8	14	5	1	5	2	7	43	9	19	6	10	2	5	2018Vergnaud#0722	B. suis bv2	10	242		BCCN#04-11	Hare	2004	Lozère, France
2	4	8	14	5	1	5	2	7	43	9	13	6	13	2	5	2018Vergnaud#0726	B. suis bv2	10	242		BCCN#81-279	Hare	1981	Moselle, France
2	4	8	14	5	1	5	2	7	43	9	13	10	14	2	6	2018Vergnaud#0306	B. suis bv2	10	242		BCCN#04-17	Wild boar	2004	Italy
2	4	8	14	5	1	5	2	5	43	9	11	11	14	2	5	2006LeFleche#069	B. suis bv2	10	38		BCCN#02-47	Hare	2002	Saint-Etienne, France
2	4	8	14	5	1	5	2	6	43	9	15	4	17	2	5	2006LeFleche#072	B. suis bv2	10	39		BCCN#03-2	Hare	2003	Grenoble, France
2	4	8	14	5	1	5	2	6	43	9	14	4	15	2	5	2018Vergnaud#0724	B. suis bv2	10	39		BCCN#04-14	Hare	2004	Cher, France
2	4	8	14	5	1	5	2	6	43	9	10	9	8	2	5	2006LeFleche#037	B. suis bv2	10	39		BCCN#82-19	Hare	1982	Poland
2	4	8	14	5	1	5	2	6	43	9	5	8	13	2	5	2018Vergnaud#0723	B. suis bv2	10	39		BCCN#04-13	Hare	2004	Aveyron, France
2	4	8	14	5	1	5	2	6	43	9	8	5	8	2	8	2018Vergnaud#0709	B. suis bv2	10	39		BCCN#87-73	Hare	1987	Moselle, France
2	4	7	14	5	1	5	2	6	43	9	6	5	9	2	6	2018Vergnaud#0710	B. suis bv2	146	241		BCCN#89-156	Hare	1989	Moselle, France
2	4	9	14	6	1	5	7	6	43	9	14	10	12	2	11	2017Ferreira#067	B. suis bv2	15	239		AFSSA-98/729.	Hare	1998	France
2	4	9	14	6	1	5	7	5	43	9	18	7	11	2	11	2018Vergnaud#0160	B. suis bv2	15	240		BCCN#94-10	Hare	1994	Haut-Rhin, France
2	4	9	14	6	1	5	7	8	43	9	5	12	13	2	9	2006LeFleche#036	B. suis bv2	15	36		BCCN#82-4	Hare	1982	Epinal, France
2	4	9	14	6	1	5	7	8	43	9	14	8	11	2	5	2018Vergnaud#0708	B. suis bv2	15	36		BCCN#83-223	Hare	1983	Meuse, France
2	3	8	14	6	1	5	2	4	38	9	11	5	21	2	4	2017Ferreira#020	B. suis bv2	162	254		AFSSA-92/115.	Hare	1992	France
2	4	8	9	6	1	5	7	7	41	9	19	8	7	2	6	2006LeFleche#041	B. suis bv2	13	34		BCCN#88-57	Hare	1988	Metz, France
2	5	8	14	5	1	5	7	4	43	9	9	7	3	2	7	2014Kreizinger#007	B. suis bv2	16	57		BH19	Swine	2011	7475,Hungary
2	5	8	14	5	1	5	7	4	43	9	9	7	3	2	7	2014Kreizinger#008	B. suis bv2	16	57		BH20	Swine	2011	7475,Hungary
2	5	8	14	5	1	5	7	4	43	9	22	8	3	2	7	2014Kreizinger#006	B. suis bv2	16	57		BH11	Wild boar	2008	2370,Hungary
2	5	8	14	5	1	5	7	4	43	9	16	6	3	2	7	2014Kreizinger#046	B. suis bv2	16	57		BH9	Swine	2005	3293,Hungary
2	5	8	14	5	1	5	7	4	43	9	18	9	3	2	8	2014Kreizinger#019	B. suis bv2	16	57		BH40	Wild boar	2012	3515,Hungary
2	5	8	14	5	1	5	7	4	43	9	18	9	3	2	7	2014Kreizinger#037	B. suis bv2	16	57		BH60	Wild boar	2012	4097,Hungary
2	5	8	14	5	1	5	7	4	43	9	11	7	3	2	10	2014Kreizinger#038	B. suis bv2	16	57		BH61	Wild boar	2012	7551,Hungary
2	5	8	14	5	1	5	7	4	43	9	4	5	3	2	8	2014Kreizinger#021	B. suis bv2	16	57		BH42	Wild boar	2012	8725,Hungary
2	5	8	14	5	1	5	7	4	43	9	4	5	3	2	8	2014Kreizinger#024	B. suis bv2	16	57		BH45	Wild boar	2012	8725,Hungary
2	5	8	14	5	1	5	7	4	43	9	4	5	3	2	8	2014Kreizinger#025	B. suis bv2	16	57		BH46	Wild boar	2012	8725,Hungary
2	5	8	14	5	1	5	7	4	43	9	10	5	3	2	8	2006LeFleche#077	B. suis bv2	16	57		BCCN#02-42	Swine	2002	Croatia
2	5	8	14	5	1	5	7	4	43	9	10	5	3	2	8	2014Kreizinger#020	B. suis bv2	16	57		BH41	Wild boar	2012	7562,Hungary
2	5	8	14	5	1	5	7	4	43	9	2	5	3	2	8	2007Garcia_Yoldi.	B. suis bv2	16	57		S-86	Swine	2000	Croatia
2	5	8	14	5	1	5	7	4	43	9	11	5	3	2	8	2018Vergnaud#0303	B. suis bv2	16	57		BCCN#03-28	Wild boar	2003	Croatia
2	5	8	14	5	1	5	7	4	43	9	10	4	3	2	8	2014Kreizinger#033	B. suis bv2	16	57		BH54	Wild boar	2012	7473,Hungary
2	5	8	14	5	1	5	7	4	43	9	7	7	3	2	8	2014Kreizinger#043	B. suis bv2	16	57		BH80	Wild boar	2013	8622,Hungary
2	5	8	14	6	1	5	7	4	43	9	12	5	3	2	8	2017Ferreira#026	B. suis bv2	158	237		AFSSA-04/302.	Swine	2004	Croatia
2	5	8	14	5	1	5	7	4	43	9	11	6	12	2	8	2014Kreizinger#013	B. suis bv2	16	57		BH31	Wild boar	2011	8725,Hungary
2	5	8	14	5	1	5	7	4	43	9	11	6	12	2	8	2014Kreizinger#016	B. suis bv2	16	57		BH35	Wild boar	2011	8725,Hungary
2	5	8	14	5	1	5	7	4	43	9	11	6	11	2	8	2014Kreizinger#039	B. suis bv2	16	57		BH65	Wild boar	2012	7588,Hungary
2	5	8	14	5	1	5	7	5	43	9	16	7	3	2	8	2014Kreizinger#010	B. suis bv2	16	56		BH22	Wild boar	2011	8676,Hungary
2	5	8	14	5	1	5	7	5	43	9	8	8	3	2	8	2014Kreizinger#036	B. suis bv2	16	56		BH59	Wild boar	2012	7475,Hungary
2	5	8	14	5	1	5	7	5	43	9	13	8	3	2	9	2014Kreizinger#012	B. suis bv2	16	56		BH30	Wild boar	2011	8725,Hungary
2	5	8	14	5	1	5	7	5	43	9	9	8	3	2	9	2014Kreizinger#044	B. suis bv2	16	56		BH84	Wild boar	2013	7475,Hungary
2	5	8	14	5	1	5	7	5	43	9	14	7	3	2	9	2014Kreizinger#022	B. suis bv2	16	56		BH43	Wild boar	2012	8725,Hungary
2	5	8	14	5	1	5	7	5	43	9	14	7	3	2	9	2014Kreizinger#026	B. suis bv2	16	56		BH47	Wild boar	2012	8725,Hungary
2	5	8	14	5	1	5	7	5	43	9	14	8	3	2	9	2014Kreizinger#014	B. suis bv2	16	56		BH32	Wild boar	2011	8725,Hungary
2	5	8	14	5	1	5	7	5	43	9	14	8	3	2	9	2014Kreizinger#034	B. suis bv2	16	56		BH56	Wild boar	2012	7563,Hungary
2	5	8	14	5	1	5	7	5	43	9	15	7	3	2	9	2014Kreizinger#032	B. suis bv2	16	56		BH53	Wild boar	2012	8725,Hungary

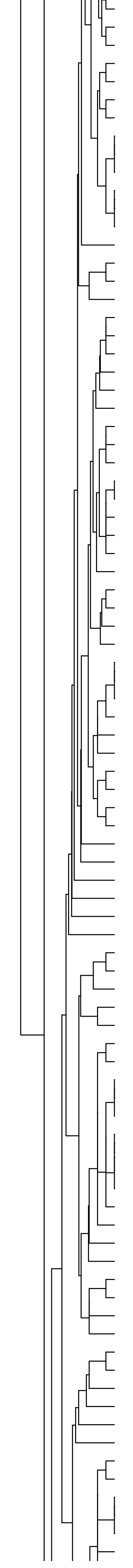

2	5	8	14	5	1	5	7	5	43	9	9	7	3	2	9	2014Kreizinger#035	B. suis bv2	16	56	BH57	Wild boar	2012	7563,Hungary
2	5	8	14	5	1	5	7	4	43	9	12	7	3	2	9	2014Kreizinger#018	B. suis bv2	16	57	BH39	Wild boar	2012	8618,Hungary
2	5	8	14	5	1	5	7	5	43	9	12	7	3	2	9	2014Kreizinger#027	B. suis bv2	16	56	BH48	Wild boar	2012	8725,Hungary
2	5	8	14	5	1	5	7	4	43	9	20	12	3	2	9	2014Kreizinger#040	B. suis bv2	16	57	BH75	Wild boar	2012	7191,Hungary
2	5	8	14	5	1	5	7	4	43	9	19	12	3	2	9	2014Kreizinger#041	B. suis bv2	16	57	BH76	Wild boar	2012	7191,Hungary
2	5	8	14	5	1	5	7	4	43	9	18	10	3	2	9	2014Kreizinger#023	B. suis bv2	16	57	BH44	Wild boar	2012	8725,Hungary
2	5	8	14	5	1	5	7	4	43	9	19	10	3	2	9	2014Kreizinger#031	B. suis bv2	16	57	BH52	Wild boar	2012	8725,Hungary
2	5	8	14	5	1	5	7	4	43	9	12	8	3	2	9	2014Kreizinger#029	B. suis bv2	16	57	BH50	Wild boar	2012	8725,Hungary
2	5	8	14	5	1	5	7	4	43	9	12	8	3	2	9	2014Kreizinger#030	B. suis bv2	16	57	BH51	Wild boar	2012	8725,Hungary
2	5	8	14	5	1	5	7	4	43	9	12	8	3	2	9	2014Kreizinger#045	B. suis bv2	16	57	BH87	Wild boar	2013	7562,Hungary
2	5	8	14	5	1	5	7	4	43	9	10	8	3	2	9	2014Kreizinger#011	B. suis bv2	16	57	BH29	Wild boar	2011	8725,Hungary
2	5	8	14	5	1	5	7	4	43	9	10	8	3	2	9	2014Kreizinger#015	B. suis bv2	16	57	BH33	Wild boar	2011	8725,Hungary
2	5	8	14	5	1	5	7	4	43	9	10	8	3	2	9	2014Kreizinger#017	B. suis bv2	16	57	BH36	Wild boar	2011	8725,Hungary
2	5	8	9	5	1	5	7	4	43	9	7	8	3	2	7	2014Kreizinger#047	B. suis bv2	20	55	BH17	Swine	2011	7822,Hungary
2	5	8	14	5	1	5	6	4	43	9	16	8	13	2	8	2014Kreizinger#067	B. suis bv2	154	214	BH10	Swine	2008	6133,Hungary
2	5	8	14	5	1	5	6	4	43	9	17	8	13	2	8	2014Kreizinger#068	B. suis bv2	154	214	BH7	Swine	1978	8124,Hungary
2	5	8	14	5	1	5	8	4	43	9	14	8	3	2	8	2014Kreizinger#054	B. suis bv2	155	213	BH93	Swine	2013	5537,Hungary
2	5	8	14	5	1	5	7	4	43	9	6	13	19	2	6	2006LeFleche#162	B. suis bv2	16	57	BfR 156	Wild boar	2002	Halle, Germany
2	5	8	14	5	1	5	7	4	43	9	6	13	21	2	6	2006LeFleche#171	B. suis bv2	16	57	BfR 167	Wild boar	2003	Halle, Germany
2	5	8	14	5	1	5	7	4	43	9	6	13	20	2	6	2006LeFleche#167	B. suis bv2	16	57	BfR 163	Wild boar	2003	Halle, Germany
2	5	8	14	5	1	5	7	4	43	9	6	16	20	2	6	2006LeFleche#173	B. suis bv2	16	57	BfR 169	Wild boar	2004	Halle, Germany
2	5	8	14	5	1	5	7	4	43	9	5	13	20	2	6	2006LeFleche#174	B. suis bv2	16	57	BfR 170	Wild boar	2004	Halle, Germany
2	5	8	14	5	1	5	7	4	43	9	15	13	14	2	6	2006LeFleche#172	B. suis bv2	16	57	BfR 168	Wild boar	2003	Halle, Germany
2	5	8	14	6	1	5	7	4	43	9	6	14	20	2	6	2017Ferreira#022	B. suis bv2	158	237	A183	Wild boar		Germany
2	5	8	14	6	1	5	7	4	43	9	6	14	18	2	6	2017Ferreira#024	B. suis bv2	158	237	04RB0377	Wild boar		Germany
2	5	8	14	6	1	5	7	4	43	9	6	14	19	2	6	2017Ferreira#023	B. suis bv2	158	237	A196	Wild boar		Germany
2	5	8	14	5	1	5	7	4	43	9	6	14	19	2	6	2006LeFleche#166	B. suis bv2	16	57	BfR 161	Wild boar	2003	Halle, Germany
2	5	8	14	5	1	5	7	4	43	9	6	14	19	2	6	2018Vergnaud#0168	B. suis bv2	16	57	BCCN#02-25	Wild boar	2002	Germany
2	5	8	14	5	1	5	7	4	43	9	6	14	11	2	6	2006LeFleche#157	B. suis bv2	16	57	BfR 151	Wild boar	2001	Halle, Germany
2	5	8	14	5	1	5	7	4	43	9	6	14	20	2	6	2006LeFleche#165	B. suis bv2	16	57	BfR 160	Wild boar	2003	Halle, Germany
2	5	8	14	5	1	5	7	4	43	9	6	14	14	2	6	2018Vergnaud#0166	B. suis bv2	16	57	BCCN#02-22	Swine	2002	Nièvre, France
2	5	8	14	5	1	5	7	5	43	9	6	14	20	2	6	2006LeFleche#150	B. suis bv2	16	56	BfR 140	Wild boar	2000	Halle, Germany
2	5	8	14	5	1	5	7	4	43	9	15	14	13	2	6	2006LeFleche#152	B. suis bv2	16	57	BfR 143	Boar	2000	Halle, Germany
2	5	8	14	5	1	5	7	4	43	9	14	14	13	2	6	2006LeFleche#158	B. suis bv2	16	57	BfR 152	Wild boar	2001	Halle, Germany
2	5	8	14	5	1	5	7	4	43	9	14	13	13	2	6	2006LeFleche#153	B. suis bv2	16	57	BfR 144	Boar	2001	Halle, Germany
2	5	8	14	5	1	5	7	4	43	9	15	12	13	2	6	2006LeFleche#175	B. suis bv2	16	57	BfR 171	Wild boar	2004	Halle, Germany
2	5	8	14	5	1	5	7	4	43	9	12	10	18	2	6	2018Vergnaud#0150	B. suis bv2	16	57	BCCN#74-372	Swine	1974	Belgium
2	5	8	14	5	1	5	7	4	43	9	12	10	18	2	6	2018Vergnaud#0152	B. suis bv2	16	57	BCCN#74-375	Swine	1974	Belgium
2	5	8	14	5	1	5	7	4	43	9	12	10	18	2	6	2018Vergnaud#0287	B. suis bv2	16	57	BCCN#74-370	Swine	1974	Belgium
2	5	8	14	5	1	5	7	4	43	9	12	10	6	2	6	2018Vergnaud#0151	B. suis bv2	16	57	BCCN#74-374	Swine	1974	Belgium
2	5	8	14	5	1	5	7	4	43	9	13	10	19	2	6	2018Vergnaud#0154	B. suis bv2	16	57	BCCN#74-377	Swine	1974	Belgium
2	5	8	14	5	1	5	7	4	43	9	12	11	19	2	6	2018Vergnaud#0148	B. suis bv2	16	57	BCCN#74-367	Swine	1974	Belgium
2	5	8	14	5	1	5	7	4	43	9	11	10	23	2	6	2014Kreizinger#028	B. suis bv2	16	57	BH49	Wild boar	2012	8725,Hungary
2	5	8	14	5	1	5	7	4	43	9	11	10	20	2	6	2018Vergnaud#0153	B. suis bv2	16	57	BCCN#74-376	Swine	1974	Belgium
2	5	8	14	5	1	5	7	4	43	9	11	11	3	2	6	2014Kreizinger#009	B. suis bv2	16	57	BH21	Wild boar	2011	8676,Hungary
2	5	8	14	5	1	5	7	4	43	9	11	11	21	2	6	2018Vergnaud#0149	B. suis bv2	16	57	BCCN#74-369	Swine	1974	Belgium
2	5	8	14	6	1	5	7	4	43	9	8	9	10	2	6	2017Ferreira#025	B. suis bv2	158	237	05RB0007	Wild boar		Germany
2	5	8	14	5	1	5	7	5	43	9	16	11	7	2	6	2014Kreizinger#042	B. suis bv2	16	56	BH77	Wild boar	2013	4834,Hungary
2	5	8	9	5	1	5	7	4	43	9	10	7	17	2	8	2006LeFleche#160	B. suis bv2	20	55	BfR 154	Swine	2002	Leipzig, Germany
2	5	8	14	5	1	5	7	4	45	9	14	6	17	2	7	2018Vergnaud#0172	B. suis bv2	16	238	BCCN#06-62		2006	United Kingdom
2	5	8	14	4	1	5	7	5	43	9	9	8	3	2	10	2014Kreizinger#053	B. suis bv2	156	215	BH55	Wild boar	2012	7563,Hungary
2	5	8	14	6	1	5	7	6	43	9	12	12	16	2	6	2017Ferreira#027	B. suis bv2	158	236	05RB1442	Hare		Germany
2	5	8	14	5	1	5	7	5	41	9	4	12	18	2	5	2006LeFleche#054	B. suis bv2	16	53	BCCN#01-25	Boar	2001	Germany
2	5	8	14	5	1	5	7	5	41	9	7	12	18	2	5	2006LeFleche#169	B. suis bv2	16	53	BfR 165	Wild boar	1999	Halle, Germany
2	5	8	14	5	1	5	7	5	41	9	7	11	17	2	5	2006LeFleche#168	B. suis bv2	16	53	BfR 164	Wild boar	2003	Halle, Germany
2	5	8	14	5	1	5	7	7	41	9	12	9	10	2	5	2006LeFleche#161	B. suis bv2	16	52	BfR 155	Wild boar	2002	Halle, Germany
2	5	8	14	5	1	5	7	7	41	9	11	9	11	2	5	2006LeFleche#164	B. suis bv2	16	52	BfR 158	Wild boar	2003	Halle, Germany
2	5	8	14	5	1	5	7	6	41	9	8	7	9	2	5	2006LeFleche#049	B. suis bv2	16	54	BCCN#98-9	Boar	1998	Limoges, France
2	5	8	14	5	1	5	7	6	41	9	8	7	11	2	5	2018Vergnaud#1253	B. suis bv2	16	54	BCCN#97-59	Swine	1997	Vienne, France
2	5	8	14	5	1	5	7	6	41	9	22	7	3	2	5	2018Vergnaud#0866	B. suis bv2	16	54	BCCN#97-101	Swine	1997	Dordogne, France
2	5	8	14	5	1	5	7	6	41	9	22	7	3	2	5	2018Vergnaud#0867	B. suis bv2	16	54	BCCN#97-102	Swine	1997	Dordogne, France
2	5	8	14	5	1	5	7	6	41	9	22	7	3	2	5	2018Vergnaud#0869	B. suis bv2	16	54	BCCN#97-107	Swine	1997	Dordogne, France
2	5	8	14	5	1	5	7	6	41	9	22	7	13	2	5	2006LeFleche#048	B. suis bv2	16	54	BCCN#97-108	Swine	1997	Perigueux, France
2	5	8	14	5	1	5	7	6	41	9	22	7	13	2	5	2018Vergnaud#0868	B. suis bv2	16	54	BCCN#97-106	Swine	1997	Dordogne, France
2	5	8	14	5	1	5	7	6	41	9	22	7	13	2	5	2018Vergnaud#1022	B. suis bv2	16	54	BCCN#97-103	Swine	1997	Dordogne, France
2	5	8	14	5	1	5	7	6	41	9	22	7	13	2	5	2018Vergnaud#1024	B. suis bv2	16	54	BCCN#97-105	Swine	1997	Dordogne, France
2	5	8	14	5	1	5	7	6	41	9	22	7	14	2	5	2018Vergnaud#1023	B. suis bv2	16	54	BCCN#97-104	Swine	1997	Dordogne, France
2	5	8	14	5	1	5	7	6	41	9	20	7	15	2	5	2006LeFleche#047	B. suis bv2	16	54	BCCN#96-140	Swine	1996	Angouleme, France
2	5	8	14	5	1	5	7	6	41	9	15	8	17	2	5	2006LeFleche#073	B. suis bv2	16	54	BCCN#03-3	Boar	2003	Tours, France
2	5	8	14	5	1	5	7	6	41	9	9	7	8	2	8	2006LeFleche#170	B. suis bv2	16	54	BfR 166	Wild boar	1999	Bad Langensalza, Ger.
2	5	8	14	5	1	5	7	6	41	9	17	14	11	2	6	2006LeFleche#149	B. suis bv2	16	54	BfR 139	Wild boar	2000	Frankfurt/O., Germany
2	5	8	14	5	1	5	7	6	41	9	13	14	11	2	6	2018Vergnaud#0292	B. suis bv2	16	54	BCCN#01-27	Wild boar	2001	Berlin ,Germany
2	5	8	14	5	1	5	7	6	41	9	16	11	22	2	6	2006LeFleche#154	B. suis bv2	16	54	BfR 147	Swine	2000	Potsdam, Germany
2	5	8	14	5	1	5	7	6	41	9	18	13	16	2	6	2006LeFleche#159	B. suis bv2	16	54	BfR 153	Boar	2002	Bad Langensalza, Ger.
2	5	7	14	5	1	5	8	5	43	9	18	8	11	2	4	2014Kreizinger#050	B. suis bv2	160	220	BH72	Wild boar	2012	4552,Hungary
2	5	7	14	5	1	5	8	5	43	9	18	8	10	2	4	2014Kreizinger#051	B. suis bv2	160	220	BH73	Wild boar	2012	4552,Hungary
2	5	7	14	5	1	5	8	6	43	9	8	8	3	2	4	2014Kreizinger#052	B. suis bv2	160	219	BH74	Wild boar	2012	3515,Hungary
2	5	8	14	5	1	5	8	5	43	9	7	8	9	2	4	2014Kreizinger#055	B. suis bv2	155	216	BH34	Wild boar	2011	8725,Hungary
2	5	7	9	5	1	5	8	5	43	9	7	18	12	2	4	2014Kreizinger#048	B. suis bv2	161	221	BH6	Swine	2003	5400,Hungary
2	5	7	14	5	1	5	8	5	43	9	14	7	6	2	3	2014Kreizinger#049	B. suis bv2	160	220	BH71	Wild boar	2012	4937,Hungary
2	5	8	14	5	1	5	8	6	43	9	10	5	4	2	4	2014Kreizinger#062	B. suis bv2	155	218	BH37	Wild boar	2012	8618,Hungary
2	5	8	14	5	1	5	8	6	43	9	11	5	4	2	4	2014Kreizinger#063	B. suis bv2	155	218	BH38	Wild boar	2012	8618,Hungary
2	5	8	14	5	1	5	8	6	43	9	19	5	6	2	4	2014Kreizinger#058	B. suis bv2	155	218	BH25	Wild boar	2011	7475,Hungary
2	5	8	14	5	1	5	8	6	43	9	19	5	6	2	4	2014Kreizinger#060	B. suis bv2	155	218	BH27	Wild boar	2011	7475,Hungary
2	5	8	14	5	1	5	8	6	43	9	19	5	6	2	4	2014Kreizinger#061	B. suis bv2	155	218	BH28	Wild boar	2011	7475,Hungary
2	5	8	14	5	1	5	8	6	43	9	21	5	7	2	4	2014Kreizinger#059	B. suis bv2	155	218	BH26	Wild boar	2011	7475,Hungary

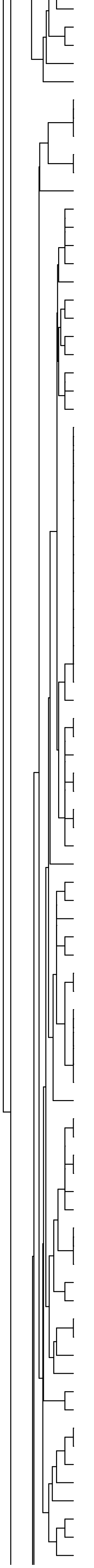

2	5	8	14	5	1	5	8	6	43	9	14	5	13	2	4	2014Kreizinger#065	B. suis bv2	155	218	BH91	Wild boar	2013	7551,Hungary
2	5	8	14	5	1	5	8	6	43	9	6	6	5	2	4	2014Kreizinger#056	B. suis bv2	155	218	BH23	Wild boar	2011	8622,Hungary
2	5	8	14	5	1	5	8	6	43	9	6	6	7	2	4	2014Kreizinger#057	B. suis bv2	155	218	BH24	Wild boar	2011	8622,Hungary
2	5	8	14	5	1	5	8	7	43	9	9	5	10	2	4	2014Kreizinger#066	B. suis bv2	155	217	BH63	Wild boar	2012	7477,Hungary
2	5	8	14	5	1	5	8	6	43	9	8	6	6	2	5	2014Kreizinger#064	B. suis bv2	155	218	BH58	Wild boar	2012	7475,Hungary
2	5	8	9	5	1	5	5	7	38	9	15	5	18	2	7	2017Ferreira#177	B. suis bv2	19	233	LNIV-4193(1)/09	Wild boar	2009	Alentejo,Portugal
2	5	8	9	5	1	5	5	7	38	9	15	5	18	2	7	2017Ferreira#178	B. suis bv2	19	233	LNIV-4193(2)/09	Wild boar	2009	Alentejo,Portugal
2	5	8	9	5	1	5	5	7	38	9	15	5	18	2	7	2017Ferreira#179	B. suis bv2	19	233	LNIV-4193(9)/09	Wild boar	2009	Alentejo,Portugal
2	5	8	9	5	1	5	5	7	38	9	15	6	14	2	8	2017Ferreira#175	B. suis bv2	19	233	LNIV-22497(47.	Ovine	2008	Alentejo,Portugal
2	5	8	9	5	1	5	5	7	38	9	15	6	14	2	8	2017Ferreira#176	B. suis bv2	19	233	LNIV-22498(69.	Ovine	2008	Alentejo,Portugal
2	5	8	9	5	1	5	5	7	38	9	12	4	19	2	6	2017Ferreira#180	B. suis bv2	19	233	LNIV-9H	Swine	2011	Trás-os-Montes,Portugal
2	5	8	9	5	1	5	5	6	38	9	14	5	7	2	6	2006LeFleche#061	B. suis bv2	19	58	BCCN#01-45	Swine	2001	Salamanca, Spain
2	5	8	9	5	1	5	5	6	38	9	2	5	7	2	6	2007Garcia_Yoldi.	B. suis bv2	19	58	S-30	Swine	2000	Salamanca, Spain
2	5	8	9	5	1	5	5	6	38	9	10	5	7	2	6	2017Ferreira#137	B. suis bv2	19	58	LNIV-3115(13).	Wild boar	2009	Alentejo,Portugal
2	5	8	9	5	1	5	5	6	38	9	21	5	7	2	6	2017Ferreira#138	B. suis bv2	19	58	LNIV-4478(4)/09	Wild boar	2009	Algarve,Portugal
2	5	8	9	5	1	5	5	6	38	9	21	5	8	2	6	2017Ferreira#139	B. suis bv2	19	58	LNIV-4187(7)/09	Wild boar	2009	Alentejo,Portugal
2	5	8	9	5	1	5	5	6	38	9	13	5	16	2	6	2017Ferreira#140	B. suis bv2	19	58	LNIV-3478(3)/09	Wild boar	2009	Beira Interior,Portugal
2	5	8	9	5	1	5	5	6	38	9	13	5	6	2	6	2018Vergnaud#1025	B. suis bv2	19	58	BCCN#01-46	Swine	2001	Salamanca, Spain
2	5	8	9	5	1	5	5	6	38	9	13	5	19	2	6	2006LeFleche#059	B. suis bv2	19	58	BCCN#01-41	Swine	2001	Badajoz, Spain
2	5	8	9	5	1	5	5	6	38	9	2	5	19	2	6	2007Garcia_Yoldi.	B. suis bv2	19	58	S-25	Swine	2000	Badajoz, Spain
2	5	8	9	5	1	5	5	6	38	9	2	5	13	2	6	2007Garcia_Yoldi.	B. suis bv2	19	58	S-45	Swine	2002	Soria, Spain
2	5	8	9	5	1	5	5	6	38	9	2	5	9	2	6	2007Garcia_Yoldi.	B. suis bv2	19	58	S-59	Swine	2003	Soria, Spain
2	5	8	9	5	1	5	5	6	38	9	2	5	17	2	6	2007Garcia_Yoldi.	B. suis bv2	19	58	S-49	Swine	2002	Rocio, Spain
2	5	8	9	5	1	5	5	6	38	9	8	5	15	2	6	2006LeFleche#055	B. suis bv2	19	58	BCCN#01-30	Swine	2001	Portugal
2	5	8	9	5	1	5	5	6	38	9	8	5	15	2	6	2017Ferreira#141	B. suis bv2	19	58	LNIV-438/00	Swine	2000	Beira Interior,Portugal
2	5	8	9	5	1	5	5	6	38	9	8	5	15	2	6	2017Ferreira#142	B. suis bv2	19	58	LNIV-5346(1)/00	Swine	2000	Ribatejo e Oeste,Portugal
2	5	8	9	5	1	5	5	6	38	9	8	5	15	2	6	2017Ferreira#143	B. suis bv2	19	58	LNIV-5346(16).	Swine	2000	Ribatejo e Oeste,Portugal
2	5	8	9	5	1	5	5	6	38	9	8	5	15	2	6	2017Ferreira#144	B. suis bv2	19	58	LNIV-5346(3)/00	Swine	2000	Ribatejo e Oeste,Portugal
2	5	8	9	5	1	5	5	6	38	9	8	5	15	2	6	2017Ferreira#145	B. suis bv2	19	58	LNIV-6552(3)/00	Swine	2000	Ribatejo e Oeste,Portugal
2	5	8	9	5	1	5	5	6	38	9	8	5	15	2	6	2017Ferreira#146	B. suis bv2	19	58	LNIV-6552(8)/00	Swine	2000	Ribatejo e Oeste,Portugal
2	5	8	9	5	1	5	5	6	38	9	8	5	15	2	6	2017Ferreira#147	B. suis bv2	19	58	LNIV-6552(2)/00	Swine	2000	Ribatejo e Oeste,Portugal
2	5	8	9	5	1	5	5	6	38	9	8	5	15	2	6	2017Ferreira#148	B. suis bv2	19	58	LNIV-2739(18).	Swine	2002	Alentejo,Portugal
2	5	8	9	5	1	5	5	6	38	9	8	5	15	2	6	2017Ferreira#149	B. suis bv2	19	58	LNIV-2739(9)/02	Swine	2002	Alentejo,Portugal
2	5	8	9	5	1	5	5	6	38	9	8	5	15	2	6	2017Ferreira#150	B. suis bv2	19	58	AFSSA-PT-655.	Swine	2000	Ribatejo e Oeste,Portugal
2	5	8	9	5	1	5	5	6	38	9	8	5	15	2	6	2017Ferreira#151	B. suis bv2	19	58	LNIV-4215(20).	Swine	2001	Ribatejo e Oeste,Portugal
2	5	8	9	5	1	5	5	6	38	9	8	5	15	2	6	2018Vergnaud#0296	B. suis bv2	19	58	BCCN#01-52	Swine	2001	Badajoz, Portugal
2	5	8	9	5	1	5	5	6	38	9	8	5	15	2	6	2018Vergnaud#0297	B. suis bv2	19	58	BCCN#01-53	Swine	2001	Badajoz, Portugal
2	5	8	9	5	1	5	5	6	38	9	8	5	15	2	6	2018Vergnaud#0298	B. suis bv2	19	58	BCCN#01-54	Swine	2001	Badajoz, Portugal
2	5	8	9	5	1	5	5	6	38	9	2	5	15	2	6	2007Garcia_Yoldi.	B. suis bv2	19	58	S-56	Swine	2002	Soria, Spain
2	5	8	9	5	1	5	5	6	38	9	9	5	17	2	6	2017Ferreira#160	B. suis bv2	19	58	LNIV-Soc35/00	Swine	2000	Trás-os-Montes,Portugal
2	5	8	9	5	1	5	5	6	38	9	9	5	17	2	6	2017Ferreira#161	B. suis bv2	19	58	LNIV-Soc50/00	Swine	2000	Trás-os-Montes,Portugal
2	5	8	9	5	1	5	5	6	38	9	9	5	14	2	6	2017Ferreira#157	B. suis bv2	19	58	LNIV-7676/00	Cattle	2000	Algarve,Portugal
2	5	8	9	5	1	5	5	6	38	9	9	5	19	2	6	2017Ferreira#154	B. suis bv2	19	58	LNIV-468/00	Swine	2000	Beira Interior,Portugal
2	5	8	9	5	1	5	5	6	38	9	9	5	19	2	6	2017Ferreira#155	B. suis bv2	19	58	LNIV-Soc29/00	Swine	2000	Trás-os-Montes,Portugal
2	5	8	9	5	1	5	5	6	38	9	9	5	15	2	6	2017Ferreira#152	B. suis bv2	19	58	LNIV-6552(10).	Swine	2000	Ribatejo e Oeste,Portugal
2	5	8	9	5	1	5	5	6	38	9	9	5	15	2	6	2017Ferreira#153	B. suis bv2	19	58	LNIV-2739(3)/02	Swine	2002	Alentejo,Portugal
2	5	8	9	5	1	5	5	6	38	9	9	5	18	2	6	2017Ferreira#156	B. suis bv2	19	58	LNIV-Soc43/00	Swine	2000	Trás-os-Montes,Portugal
2	5	8	9	5	1	5	5	7	38	9	9	5	21	2	6	2017Ferreira#163	B. suis bv2	19	233	LNIV-46685(15.	Wild boar	2008	Beira Interior,Portugal
2	5	8	9	5	1	5	5	6	38	9	13	5	7	2	8	2017Ferreira#164	B. suis bv2	19	58	LNIV-9789(J10).	Wild boar	2008	Entre Douro e Minho,Po.
2	5	8	9	5	1	5	5	6	38	9	12	5	7	2	8	2017Ferreira#165	B. suis bv2	19	58	LNIV-9789(Feto.	Wild boar	2008	Trás-os-Montes,Portugal
2	5	8	9	5	1	5	5	6	38	9	17	5	17	2	8	2017Ferreira#166	B. suis bv2	19	58	LNIV-44406(3).	Wild boar	2009	Beira Interior,Portugal
2	5	8	9	5	1	5	5	6	38	9	14	5	18	2	8	2006LeFleche#060	B. suis bv2	19	58	BCCN#01-43	Swine	2001	Badajoz, Spain
2	5	8	9	5	1	5	5	6	38	9	2	5	18	2	8	2007Garcia_Yoldi.	B. suis bv2	19	58	S-28	Swine	2000	Badajoz, Spain
2	5	8	9	5	1	5	5	6	38	9	15	5	12	2	8	2017Ferreira#172	B. suis bv2	19	58	S34	Swine	2000	Toledo,Spain
2	5	8	9	5	1	5	5	6	38	9	15	5	12	2	8	2018Vergnaud#0745	B. suis bv2	19	58	BCCN#01-44	Swine	2001	Badajoz, Portugal
2	5	8	9	5	1	5	5	6	38	9	15	5	20	2	8	2017Ferreira#167	B. suis bv2	19	58	S32	Swine	2000	Toledo,Spain
2	5	8	9	5	1	5	5	6	38	9	15	5	20	2	8	2017Ferreira#168	B. suis bv2	19	58	LNIV-5414(12).	Swine	2003	Alentejo,Portugal
2	5	8	9	5	1	5	5	6	38	9	15	5	20	2	8	2017Ferreira#169	B. suis bv2	19	58	LNIV-5414(13).	Swine	2003	Alentejo,Portugal
2	5	8	9	5	1	5	5	6	38	9	15	5	20	2	8	2017Ferreira#170	B. suis bv2	19	58	LNIV-5414(2)/03	Swine	2003	Alentejo,Portugal
2	5	8	9	5	1	5	5	6	38	9	15	5	20	2	8	2017Ferreira#171	B. suis bv2	19	58	LNIV-5414(13).	Swine	2003	Alentejo,Portugal
2	5	8	9	5	1	5	5	6	38	9	15	5	19	2	5	2018Vergnaud#0744	B. suis bv2	19	58	BCCN#01-42	Swine	2001	Badajoz, Portugal
2	5	8	9	5	1	5	5	6	38	9	9	5	15	2	7	2017Ferreira#162	B. suis bv2	19	58	LNIV-1967/00	Swine	2000	Beira Interior,Portugal
2	5	8	9	5	1	5	5	6	38	9	9	5	15	2	7	2018Vergnaud#0169	B. suis bv2	19	58	BCCN#02-27	Swine	2002	Portugal
2	5	8	9	5	1	5	5	6	38	9	9	5	17	2	7	2017Ferreira#158	B. suis bv2	19	58	LNIV-1262/00	Swine	2000	Beira Interior,Portugal
2	5	8	9	5	1	5	5	6	38	9	9	5	17	2	7	2017Ferreira#159	B. suis bv2	19	58	LNIV-Soc39/00	Swine	2000	Trás-os-Montes,Portugal
2	5	8	9	5	1	5	5	6	38	9	9	5	14	2	7	2006LeFleche#063	B. suis bv2	19	58	BCCN#01-51	Swine	2001	Badajoz, Spain
2	5	8	9	5	1	5	5	6	38	9	9	5	18	2	7	2017Ferreira#131	B. suis bv2	19	58	LNIV-Soc41/00	Swine	2000	Trás-os-Montes,Portugal
2	5	8	9	5	1	5	5	6	38	9	9	6	18	2	7	2017Ferreira#128	B. suis bv2	19	58	LNIV-Soc57/00	Swine	2000	Trás-os-Montes,Portugal
2	5	8	9	5	1	5	5	6	38	9	9	6	18	2	7	2017Ferreira#129	B. suis bv2	19	58	LNIV-Soc64/00	Swine	2000	Trás-os-Montes,Portugal
2	5	8	9	5	1	5	5	6	38	9	9	6	18	2	7	2017Ferreira#130	B. suis bv2	19	58	LNIV-Soc73/00	Swine	2000	Trás-os-Montes,Portugal
2	5	8	9	5	1	5	5	6	38	9	10	5	20	2	7	2018Vergnaud#0295	B. suis bv2	19	58	BCCN#01-37	Swine	2001	Salamanque, Spain
2	5	8	9	5	1	5	5	6	38	9	10	5	11	2	7	2018Vergnaud#1254	B. suis bv2	19	58	BCCN#01-39	Swine	2001	Salamanque, Spain
2	5	8	9	5	1	5	5	6	38	9	21	6	15	2	7	2017Ferreira#133	B. suis bv2	19	58	LNIV-14256(72.	Swine	2009	Ribatejo e Oeste,Portugal
2	5	8	9	5	1	5	5	6	38	9	21	6	15	2	7	2017Ferreira#134	B. suis bv2	19	58	LNIV-231(1)/09	Swine	2009	Ribatejo e Oeste,Portugal
2	5	8	9	5	1	5	5	5	38	9	21	6	16	2	7	2017Ferreira#135	B. suis bv2	19	60	LNIV-14256(40.	Swine	2009	Ribatejo e Oeste,Portugal
2	5	8	9	5	1	5	5	6	38	9	2	6	13	2	7	2007Garcia_Yoldi.	B. suis bv2	19	58	S-64	Swine	2003	Badajoz, Spain
2	5	8	9	5	1	5	5	6	38	9	15	7	15	2	9	2017Ferreira#173	B. suis bv2	19	58	LNIV-21566(94.	Wild boar	2009	Trás-os-Montes,Portugal
2	5	8	9	5	1	5	5	6	38	9	15	8	15	2	9	2017Ferreira#174	B. suis bv2	19	58	LNIV-S-384(679)	Swine	2009	Piensos del segre, Spain
2	5	8	9	5	1	5	5	6	38	9	13	6	10	2	5	2017Ferreira#121	B. suis bv2	19	58	LNIV-44821(81.	Wild boar	2009	Ribatejo e Oeste,Portugal
2	5	8	9	5	1	5	5	6	38	9	13	6	10	2	5	2017Ferreira#122	B. suis bv2	19	58	LNIV-44821(86.	Wild boar	2009	Ribatejo e Oeste,Portugal
2	5	8	9	5	1	5	5	6	38	9	13	6	17	2	5	2017Ferreira#123	B. suis bv2	19	58	LNIV-44821(79.	Wild boar	2009	Ribatejo e Oeste,Portugal
2	5	8	9	5	1	5	5	6	38	9	10	6	7	2	5	2017Ferreira#124	B. suis bv2	19	58	LNIV-3115(9)/09	Wild boar	2009	Alentejo,Portugal
2	5	8	9	5	1	5	5	6	38	9	9	6	10	2	6	2017Ferreira#132	B. suis bv2	19	58	LNIV-1344(3)/09	Wild boar	2009	Beira Interior,Portugal
2	5	8	9	5	1	5	5	6	38	9	11	6	19	2	9	2017Ferreira#125	B. suis bv2	19	58	LNIV-3514(2)/10	Wild boar	2010	Alentejo,Portugal
2	5	8	9	5	1	5	5	6	38	9	12	6	19	2	9	2017Ferreira#126	B. suis bv2	19	58	LNIV-3515(6)/10	Wild boar	2010	Alentejo,Portugal
2	5	8	9	5	1	5	5	6	38	9	20	6	19	2	6	2017Ferreira#127	B. suis bv2	19	58	LNIV-44401(3).	Wild boar	2009	Beira Interior,Portugal

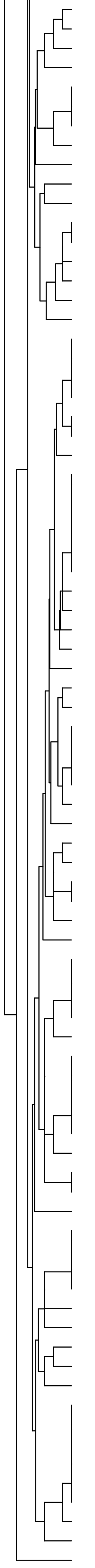

2	5	8	9	5	1	5	4	6	38	9	2	5	9	2	8	2007Garcia_Yoldi.	B. suis bv2	18	59		S-143	Wild boar	2005	Spain
2	5	8	9	5	1	5	4	6	38	9	2	5	17	2	8	2007Garcia_Yoldi.	B. suis bv2	18	59		S-145	Wild boar	2005	Spain
2	5	8	9	5	1	5	4	6	38	9	2	7	10	2	8	2007Garcia_Yoldi.	B. suis bv2	18	59		S-147	Wild boar	2005	Spain
2	5	8	9	5	1	5	4	6	38	9	9	14	15	2	8	2017Ferreira#117	B. suis bv2	18	59		LNIV-4477(4)/09	Wild boar	2009	Algarve,Portugal
2	5	8	9	5	1	5	4	6	38	9	20	9	13	2	5	2006LeFleche#057	B. suis bv2	18	59		BCCN#01-36	Swine	2001	Huelva, Spain
2	5	8	9	5	1	5	4	6	38	9	20	9	13	2	5	2017Ferreira#119	B. suis bv2	18	59		S27	Swine	2000	Badajoz,Spain
2	5	8	9	5	1	5	4	6	38	9	20	9	13	2	5	2017Ferreira#120	B. suis bv2	18	59		S13	Swine	1992	Salamanca,Spain
2	5	8	9	5	1	5	4	6	38	9	2	8	13	2	5	2007Garcia_Yoldi.	B. suis bv2	18	59		S-21	Swine	1999	Huelva, Spain
2	5	8	9	5	1	5	4	6	38	9	13	15	8	2	4	2017Ferreira#118	B. suis bv2	18	59		LNIV-J2A/08	Wild boar	2009	Beira Interior,Portugal
2	5	8	9	5	1	5	4	6	38	9	2	8	5	2	7	2007Garcia_Yoldi.	B. suis bv2	18	59		S-148	Wild boar	2005	Spain
2	5	8	9	5	1	5	5	6	38	9	3	8	12	2	7	2017Ferreira#136	B. suis bv2	19	58		LNIV-1989(112.	Wild boar	2010	Trás-os-Montes,Portugal
2	5	8	9	5	1	5	4	6	38	9	7	7	12	2	7	2007Garcia_Yoldi.	B. suis bv2	18	59		S-13	Swine	1998	Extremadura, Spain
2	5	8	9	5	1	5	4	6	38	9	7	7	12	2	7	2018Vergnaud#0294	B. suis bv2	18	59		BCCN#01-35	Swine	2001	Badajoz, Portugal
2	5	8	9	5	1	5	4	6	38	9	7	7	11	2	7	2006LeFleche#056	B. suis bv2	18	59		BCCN#01-34	Swine	2001	Badajoz, Spain
2	5	8	9	5	1	5	4	6	38	9	7	7	7	2	7	2017Ferreira#114	B. suis bv2	18	59		S12	Swine	1992	Salamanca,Spain
2	5	8	9	5	1	5	4	7	38	9	7	7	11	2	7	2018Vergnaud#0293	B. suis bv2	18	234		BCCN#01-33	Swine	2001	Badajoz, Portugal
2	5	8	9	5	1	5	4	6	38	9	9	12	11	2	7	2017Ferreira#116	B. suis bv2	18	59		LNIV-45014(4).	Wild boar	2009	Alentejo,Portugal
2	5	8	9	5	1	5	5	5	38	9	20	9	14	2	7	2017Ferreira#094	B. suis bv2	19	60		LNIV-4498(J1)/08	Wild boar	2008	Trás-os-Montes,Portugal
2	5	8	9	5	1	5	5	5	38	9	20	9	14	2	7	2017Ferreira#095	B. suis bv2	19	60		LNIV-4498(J9)/08	Wild boar	2008	Trás-os-Montes,Portugal
2	5	8	9	5	1	5	5	5	38	9	20	9	14	2	7	2017Ferreira#096	B. suis bv2	19	60		LNIV-4498(J4)/08	Wild boar	2008	Trás-os-Montes,Portugal
2	5	8	9	5	1	5	5	5	38	9	20	9	14	2	7	2017Ferreira#097	B. suis bv2	19	60		LNIV-4498(J6)/08	Wild boar	2008	Trás-os-Montes,Portugal
2	5	8	9	5	1	5	5	5	38	9	15	9	14	2	7	2017Ferreira#093	B. suis bv2	19	60		S3	Swine	1992	Salamanca,Spain
2	5	8	9	5	1	5	5	5	38	9	15	9	14	2	7	2018Vergnaud#0713	B. suis bv2	19	60		BCCN#93-74	Swine	1993	Saragosse, Spain
2	5	8	9	5	1	5	5	5	38	9	15	7	14	2	7	2017Ferreira#088	B. suis bv2	19	60		S4	Swine	1992	Salamanca,Spain
2	5	8	9	5	1	5	5	5	38	9	15	9	13	2	7	2017Ferreira#091	B. suis bv2	19	60		S1	Swine	1992	Spain
2	5	8	9	5	1	5	5	5	38	9	15	9	13	2	7	2017Ferreira#092	B. suis bv2	19	60		S2	Swine	1992	Salamanca,Spain
2	5	8	9	5	1	5	5	5	38	9	15	9	13	2	7	2018Vergnaud#0159	B. suis bv2	19	60		BCCN#93-80	Swine	1993	Saragosse, Spain
2	5	8	9	5	1	5	5	5	38	9	15	9	13	2	7	2018Vergnaud#0288	B. suis bv2	19	60		BCCN#93-72	Swine	1993	Saragosse, Spain
2	5	8	9	5	1	5	5	5	38	9	15	9	13	2	7	2018Vergnaud#0865	B. suis bv2	19	60		BCCN#93-79	Swine	1993	Saragosse, Spain
2	5	8	9	5	1	5	5	5	38	9	15	9	13	2	7	2018Vergnaud#1218	B. suis bv2	19	60		BCCN#93-77	Swine	1993	Saragosse, Spain
2	5	8	9	5	1	5	5	5	38	9	12	9	13	2	7	2017Ferreira#089	B. suis bv2	19	60		S21	Swine	1999	Huelva,Spain
2	5	8	9	5	1	5	5	5	38	9	14	9	13	2	7	2018Vergnaud#0289	B. suis bv2	19	60		BCCN#93-73	Swine	1993	Saragosse, Spain
2	5	8	9	5	1	5	5	5	38	9	15	9	13	2	5	2018Vergnaud#0714	B. suis bv2	19	60		BCCN#93-76	Swine	1993	Saragosse, Spain
2	5	8	9	5	1	5	5	5	38	9	15	9	3	2	7	2018Vergnaud#0864	B. suis bv2	19	60		BCCN#93-78	Swine	1993	Saragosse, Spain
2	5	8	9	5	1	5	5	5	38	9	15	9	17	2	8	2017Ferreira#098	B. suis bv2	19	60		VLA-63/311	Swine	1963	
2	5	8	9	5	1	5	4	5	38	9	12	9	7	2	7	2017Ferreira#112	B. suis bv2	18	61		LNIV-8605(2)/10	Wild boar	2010	Alentejo,Portugal
2	5	8	9	5	1	5	4	5	38	9	13	9	7	2	7	2017Ferreira#113	B. suis bv2	18	61		LNIV-8605(9)/10	Wild boar	2010	Alentejo,Portugal
2	5	8	9	5	1	5	5	5	38	9	12	9	7	2	7	2006LeFleche#062	B. suis bv2	19	60		BCCN#01-47	Swine	2001	Toledo, Spain
2	5	8	9	5	1	5	5	5	38	9	12	9	7	2	7	2017Ferreira#090	B. suis bv2	19	60		S6	Swine	1998	Badajoz,Spain
2	5	8	9	5	1	5	5	5	38	9	12	9	7	2	7	2018Vergnaud#0871	B. suis bv2	19	60		BCCN#01-50	Swine	2001	Toledo, Spain
2	5	8	9	5	1	5	5	5	38	9	12	9	7	2	7	2018Vergnaud#1026	B. suis bv2	19	60		BCCN#01-48	Swine	2001	Toledo, Spain
2	5	8	9	5	1	5	5	5	38	9	13	9	7	2	7	2018Vergnaud#0870	B. suis bv2	19	60		BCCN#01-49	Swine	2001	Toledo, Spain
2	5	8	9	5	1	5	5	5	38	9	2	5	7	2	7	2007Garcia_Yoldi.	B. suis bv2	19	60		S-32	Swine	2000	Toledo, Spain
2	5	8	9	5	1	5	5	5	38	9	2	7	10	2	7	2007Garcia_Yoldi.	B. suis bv2	19	60		S-144	Wild boar	2005	Spain
2	5	8	9	5	1	5	5	5	38	9	20	7	10	2	7	2017Ferreira#087	B. suis bv2	19	60		Bs146	Swine		Spain
2	5	8	9	5	1	5	5	5	38	9	9	7	13	2	7	2017Ferreira#085	B. suis bv2	19	60		S22	Swine	2000	Salamanca,Spain
2	5	8	9	5	1	5	5	5	38	9	9	7	13	2	7	2018Vergnaud#0158	B. suis bv2	19	60		BCCN#93-75	Swine	1993	Saragosse, Spain
2	5	8	9	5	1	5	5	5	38	9	21	7	11	2	7	2017Ferreira#086	B. suis bv2	19	60		Bs147	Swine		Spain
2	5	8	9	5	1	5	5	5	38	9	2	9	11	2	6	2007Garcia_Yoldi.	B. suis bv2	19	60		S-149	Wild boar	2005	Spain
2	5	8	9	5	1	5	5	5	38	9	13	10	17	2	7	2017Ferreira#099	B. suis bv2	19	60		LNIV-9789(J9)/08	Wild boar	2008	Entre Douro e Minho,Po.
2	5	8	9	5	1	5	5	5	38	9	13	10	17	2	7	2017Ferreira#100	B. suis bv2	19	60		LNIV-19122(J1).	Wild boar	2008	Entre Douro e Minho,Po.
2	5	8	9	5	1	5	5	5	38	9	13	10	17	2	7	2017Ferreira#101	B. suis bv2	19	60		LNIV-17888(J3).	Wild boar	2008	Entre Douro e Minho,Po.
2	5	8	9	5	1	5	5	5	38	9	13	10	17	2	7	2017Ferreira#102	B. suis bv2	19	60		LNIV-21346(J2).	Wild boar	2008	Entre Douro e Minho,Po.
2	5	8	9	5	1	5	5	5	38	9	13	10	9	2	6	2017Ferreira#103	B. suis bv2	19	60		LNIV-2454(J10).	Wild boar	2008	Trás-os-Montes,Portugal
2	5	8	9	5	1	5	4	5	38	9	11	10	9	2	7	2017Ferreira#105	B. suis bv2	18	61		LNIV-2948(1)/09	Wild boar	2009	Alentejo,Portugal
2	5	8	9	5	1	5	4	5	38	9	11	10	9	2	7	2017Ferreira#106	B. suis bv2	18	61		LNIV-2948(5)/09	Wild boar	2009	Alentejo,Portugal
2	5	8	9	5	1	5	4	5	38	9	11	10	9	2	7	2017Ferreira#107	B. suis bv2	18	61		PT09143	Wild boar	2009	Alentejo,Portugal
2	5	8	9	5	1	5	4	5	38	9	11	10	9	2	7	2017Ferreira#108	B. suis bv2	18	61		LNIV-2948(8)/09	Wild boar	2009	Alentejo,Portugal
2	5	8	9	5	1	5	4	5	38	9	11	10	9	2	7	2017Ferreira#109	B. suis bv2	18	61		LNIV-2948(9)/09	Wild boar	2009	Alentejo,Portugal
2	5	8	9	5	1	5	5	5	38	9	11	6	9	2	7	2017Ferreira#104	B. suis bv2	19	60		LNIV-1344(2)/09	Wild boar	2009	Beira Interior,Portugal
2	5	8	9	5	1	5	5	5	38	9	11	10	7	2	4	2017Ferreira#110	B. suis bv2	19	60		LNIV-4189(1)/09	Wild boar	2009	Alentejo,Portugal
2	5	8	9	5	1	5	5	5	38	9	11	10	7	2	4	2017Ferreira#111	B. suis bv2	19	60		LNIV-4189(2)/09	Wild boar	2009	Alentejo,Portugal
2	5	8	9	5	1	5	5	5	38	9	9	15	16	2	10	2017Ferreira#115	B. suis bv2	19	60		LNIV-4647(1)/09	Wild boar	2009	Beira Interior,Portugal
2	5	8	9	5	1	5	5	5	38	9	5	7	12	2	8	2017Ferreira#080	B. suis bv2	19	60		PT09172	Wild boar	2009	Trás-os-Montes,Portugal
2	5	8	9	5	1	5	5	5	38	9	5	7	12	2	8	2017Ferreira#081	B. suis bv2	19	60		LNIV-44821(12.	Wild boar	2009	Trás-os-Montes,Portugal
2	5	8	9	5	1	5	5	5	38	9	5	7	12	2	8	2017Ferreira#082	B. suis bv2	19	60		LNIV-44821(12.	Wild boar	2009	Trás-os-Montes,Portugal
2	5	8	9	5	1	5	5	5	38	9	5	7	12	2	8	2017Ferreira#083	B. suis bv2	19	60		LNIV-44821(12.	Wild boar	2009	Trás-os-Montes,Portugal
2	5	8	9	5	1	5	5	5	38	9	4	10	13	2	8	2007Garcia_Yoldi.	B. suis bv2	19	60		S-62	Swine	2003	Oropesa, Spain
2	5	8	9	5	1	5	5	5	38	9	14	8	14	2	8	2017Ferreira#084	B. suis bv2	19	60		LNIV-46685(20.	Wild boar	2008	Beira Interior,Portugal
2	5	8	9	5	1	5	5	6	38	9	7	11	10	2	8	2017Ferreira#077	B. suis bv2	19	58		S-145(PN-II)	Wild boar	2005	Asturias,Spain
2	5	8	9	5	1	5	4	5	38	9	7	11	10	2	8	2017Ferreira#078	B. suis bv2	18	61		Bs144	Swine		Spain
2	5	8	9	5	1	5	5	5	38	9	7	12	9	2	8	2017Ferreira#079	B. suis bv2	19	60		Bs143CITA	Wild boar	2005	Asturias,Spain
2	5	8	9	5	1	5	4	5	38	9	13	13	11	2	8	2017Ferreira#069	B. suis bv2	18	61		LNIV-2948(3)/09	Wild boar	2009	Alentejo,Portugal
2	5	8	9	5	1	5	4	5	38	9	13	13	11	2	8	2017Ferreira#070	B. suis bv2	18	61		LNIV-2948(10).	Wild boar	2009	Alentejo,Portugal
2	5	8	9	5	1	5	4	5	38	9	13	13	11	2	8	2017Ferreira#071	B. suis bv2	18	61		LNIV-2948(12).	Wild boar	2009	Alentejo,Portugal
2	5	8	9	5	1	5	4	5	38	9	13	13	11	2	8	2017Ferreira#072	B. suis bv2	18	61		LNIV-2948(20).	Wild boar	2009	Alentejo,Portugal
2	5	8	9	5	1	5	4	5	38	9	13	13	11	2	8	2017Ferreira#073	B. suis bv2	18	61		LNIV-2948(27).	Wild boar	2009	Alentejo,Portugal
2	5	8	9	5	1	5	4	5	38	9	13	13	11	2	8	2017Ferreira#074	B. suis bv2	18	61		LNIV-2948(29).	Wild boar	2009	Alentejo,Portugal
2	5	8	9	5	1	5	4	5	38	9	13	10	11	2	8	2017Ferreira#075	B. suis bv2	18	61		LNIV-2948(34).	Wild boar	2009	Alentejo,Portugal
2	5	8	9	5	1	5	4	5	38	9	12	5	17	2	8	2017Ferreira#076	B. suis bv2	18	61		Bs145	Swine		Spain
2	5	8	9	6	1	5	4	5	38	9	5	9	18	2	4	GCA_000698285	B. suis bv2	172	357	39	PT09172			
